# miRNAs in the alga *Chlamydomonas reinhardtii* are not phylogenetically conserved and play a limited role in responses to nutrient deprivation

**DOI:** 10.1038/s41598-017-05561-0

**Published:** 2017-07-14

**Authors:** Adam Voshall, Eun-Jeong Kim, Xinrong Ma, Tomohito Yamasaki, Etsuko N. Moriyama, Heriberto Cerutti

**Affiliations:** 1 School of Biological Sciences, University of Nebraska-Lincoln, Lincoln, Nebraska USA; 2 Center for Plant Science Innovation, University of Nebraska-Lincoln, Lincoln, Nebraska USA; 3 Division of Environmental Photobiology, National Institute for Basic Biology, Okazaki Aichi Prefecture, Japan

## Abstract

The unicellular alga *Chlamydomonas reinhardtii* contains many types of small RNAs (sRNAs) but the biological role(s) of bona fide microRNAs (miRNAs) remains unclear. To address their possible function(s) in responses to nutrient availability, we examined miRNA expression in cells cultured under different trophic conditions (mixotrophic in the presence of acetate or photoautotrophic in the presence or absence of nitrogen). We also reanalyzed miRNA expression data in Chlamydomonas subject to sulfur or phosphate deprivation. Several miRNAs were differentially expressed under the various trophic conditions. However, in transcriptome analyses, the majority of their predicted targets did not show expected changes in transcript abundance, suggesting that they are not subject to miRNA-mediated RNA degradation. Mutant strains, defective in sRNAs or in *ARGONAUTE3* (a key component of sRNA-mediated gene silencing), did not display major phenotypic defects when grown under multiple nutritional regimes. Additionally, Chlamydomonas miRNAs were not conserved, even in algae of the closely related Volvocaceae family, and many showed features resembling those of recently evolved, species-specific miRNAs in the genus *Arabidopsis*. Our results suggest that, in *C. reinhardtii*, miRNAs might be subject to relatively fast evolution and have only a minor, largely modulatory role in gene regulation under diverse trophic states.

## Introduction

MicroRNAs are short RNA molecules (~20–24 nt in length) that generally function as negative regulators of gene expression, by binding complementary sequences in target transcripts and leading to translation repression and/or mRNA degradation^1–6^. In higher eukaryotes, miRNAs have been implicated in the control of many biological processes such as development, metabolism or stress responses^1–3, 7–9^. In contrast, the biological role(s) of miRNAs in unicellular organisms such as the alga *Chlamydomonas reinhardtii* ^4, 5^ remains unclear, although they have been recently proposed to modulate adaptation to abiotic stress^10^. In land plants, a subset of miRNAs is differentially or uniquely expressed under nutrient deprivation^8, 9, 11–13^. However, these condition-specific miRNAs often have very few, if any, targets within the pathways directly involved in the response to nutrient limitation, making it difficult to assess their overall significance^8, 9, 11–13^.

Sulfur deprivation has been reported to induce differential miRNA expression in *C. reinhardtii* but, similarly to observations in higher plants, very few predicted targets appeared to be involved in mechanisms responding directly to sulfur deficiency^6^. In addition, it is often difficult to identify genuine miRNA-regulated transcripts in Chlamydomonas^14^. For instance, several putative targets showed mRNA up-regulation when the miRNAs predicted to target them also increased in abundance^6, 15^. This expression pattern makes it unlikely that the predicted targets, if genuine, are regulated via miRNA-mediated transcript degradation although they could still be translationally modulated^14, 16, 17^. A further challenge to characterize miRNA function in *C. reinhardtii* is posed by the apparent lack of conservation among algal miRNAs and those identified in higher plants and animals^14, 18–20^.

Plasticity in miRNA populations has been reported in both animal and plant lineages, as reflected by the number of miRNAs that are either species-specific or limited to closely related species^2, 21–26^. Within the genus *Arabidopsis*, there is evidence that miRNA genes may arise from inverted duplication of sequences or from spontaneous mutations in sequences capable of forming hairpin structures^2, 20, 23, 24^. This generation of novel miRNA genes from random sequences may account for the large number of miRNAs corresponding uniquely to *A. thaliana* or *A. lyrata* (~13% of their total miRNA populations) despite their relatively recent evolutionary divergence^24, 26^. These species-specific miRNAs appear to be lowly expressed, whereas miRNAs conserved among plant species generally show higher expression levels^25, 26^. Furthermore, many novel miRNAs have no experimentally confirmed targets and their function(s) is largely unknown^24–26^ although some could conceivably play a role in lineage-specific processes such as responses to environmental conditions unique to the habitat of individual species^7, 9, 12, 27^.

In order to characterize the possible roles of miRNAs in *C. reinhardtii*, particularly in response to nutrient availability, we investigated changes in miRNA populations and their putative targets in Chlamydomonas cells grown under mixotrophic conditions (in the presence of acetate) and under photoautotrophic conditions with or without a source of nitrogen. We also examined the phenotypes of mutant strains, lacking sRNAs or defective in a core component of the RNA interference (RNAi) machinery, under nutrient deprived conditions. Subsets of differentially expressed miRNAs were identified under the various trophic conditions but very few of their predicted targets displayed expected changes in transcript abundance (assuming regulation by miRNA-triggered RNA degradation) or coded for proteins involved in direct responses to nutrient deficiency. The identified Chlamydomonas miRNAs were not conserved even in related green algal lineages and many showed relatively low expression levels, similarly to the recently evolved miRNAs characterized in higher plants. Interestingly, the RNAi defective mutants displayed slight phenotypic defects, suggesting that miRNAs might not play an essential role in endogenous gene regulation under the conditions examined.

## Results

### Changes in *C. reinhardtii* miRNA populations under various trophic conditions

**Figure 1:**
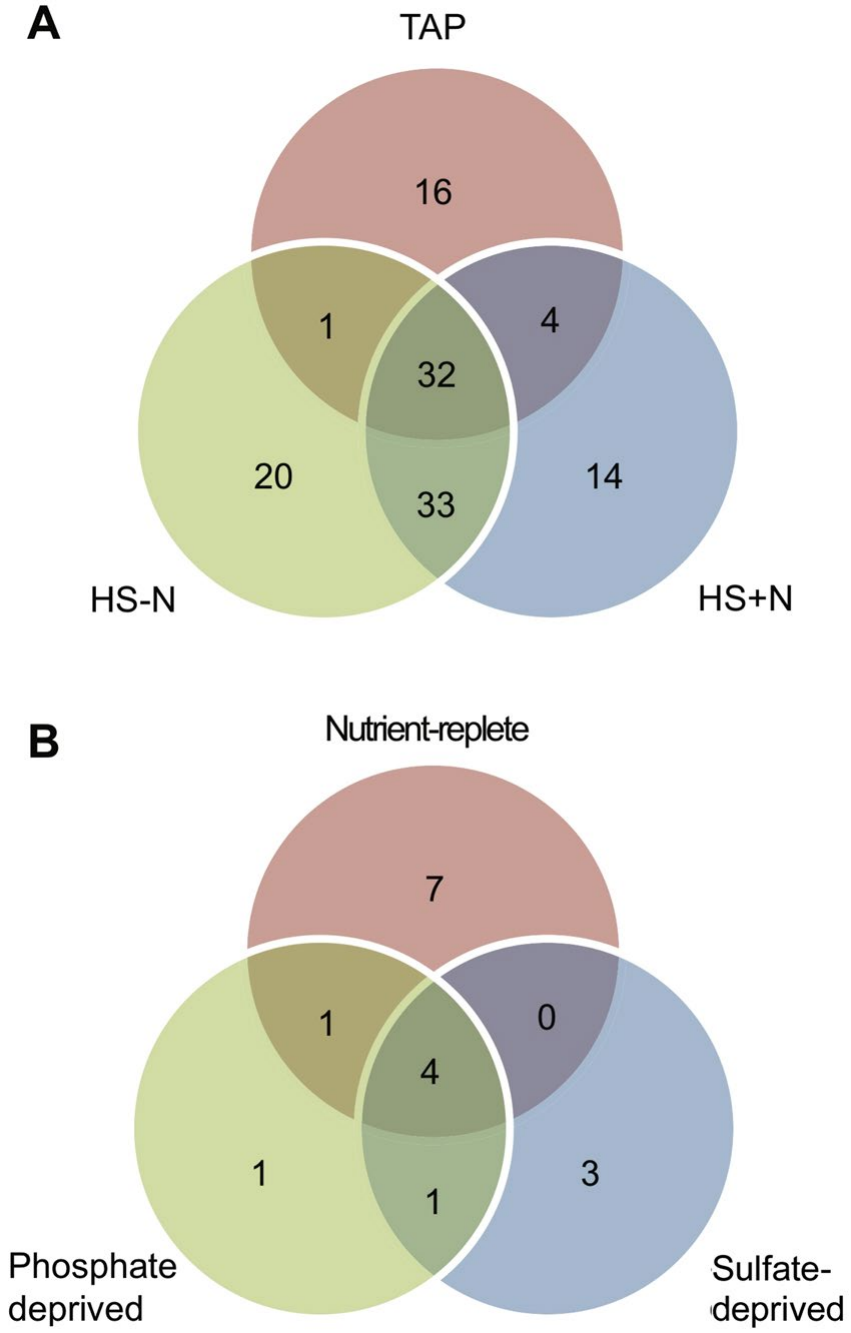
Comparison of miRNAs identified in Chlamydomonas cells grown under various nutritional deprivation conditions. Venn diagrams show the numbers of unique and shared candidate miRNAs in cells grown under the different trophic regimes. The data was obtained from the AGO3-associated sRNA libraries in this study (**A**) and from the total sRNA libraries prepared by Chávez Montes *et al*.^25^ (**B**).

Small RNAs associated with ARGONAUTE3 (AGO3), a key component of the RNAi machinery in *C. reinhardtii* ^14, 28^, were isolated by co-immunoprecipitation with FLAG-tagged AGO3, sRNA libraries constructed and then analyzed by deep sequencing (see Methods). From these sequences, miRNAs were predicted based on the criteria outlined by Tarver *et al*.^29^. To differentiate miRNAs from other sRNAs, all genome mapped reads (see Methods) were clustered by genomic location such that within each cluster adjacent reads were no more than 200 nt apart, regardless of strand^14^. Genomic sequences for each strand of each cluster were then folded using RNAfold to determine their secondary structure^14^. In order to be classified as a miRNA precursor, a cluster was required to fold into a hairpin and have no more than two predominant 5′ processing sites^14, 29^. In addition, the main reads (i.e., the greatest abundance reads in each cluster; usually representing ≥90% of the locally mapped reads) were required to have no more than four mismatches in the complementary arm of the hairpin^14, 29^. By using these criteria, we identified 120 candidate miRNAs, across three growth conditions, co-immunoprecipitating with FLAG-tagged AGO3 (Figs 1A and S1). These sequences included the 45 miRNAs previously identified in cells grown under mixotrophic conditions in TAP (Tris-Acetate-Phosphate) medium^14^ as well as 75 additional candidate miRNAs (Table S1A). Most miRNAs (83 of 120) were detected in photoautotrophically grown cells in nutrient replete high salt medium (HS + N), but only 14 were identified primarily in these cells whereas the majority (69 of 83) was shared between at least two trophic conditions (Fig. 1A and Table S1A). On the other hand, Chlamydomonas grown photoautotrophically in nitrogen deprived medium (HS − N) had 20 condition-prevalent miRNAs (Fig. 1A).

The population of AGO3-associated miRNAs clearly varies among cells grown under different trophic conditions (Table S1A). However, virtually none of the mature miRNA sequences is completely missing from libraries from any condition (Fig. S1 and Table S1A). Most precursor miRNAs appear to be transcribed and processed under all growth conditions but the generated sRNA sequences may only meet the criteria to be classified as miRNAs under one or two of the examined nutritional regimes. Often, this occurs because the mature miRNA sequence may not represent at least 90% of the local reads matching to the precursor miRNA hairpin (Fig. S1), one of the criteria for prediction of canonical miRNAs^14, 29^. When considering read abundance, most miRNAs identified as such primarily in cells grown under a specific trophic condition (*i.e*., the 16 miRNAs in TAP, the 14 miRNAs in HS + N and the 20 miRNAs in HS − N) were also present at their highest levels in the libraries from that same condition (Table S1A). Yet, there were also some inconsistencies. For instance, miR_t35 and miR_t79 were classified as miRNAs in libraries from cells grown in HS − N but they seemed to be more prevalent in cells cultured in TAP (Table S1A).

**Figure 2:**
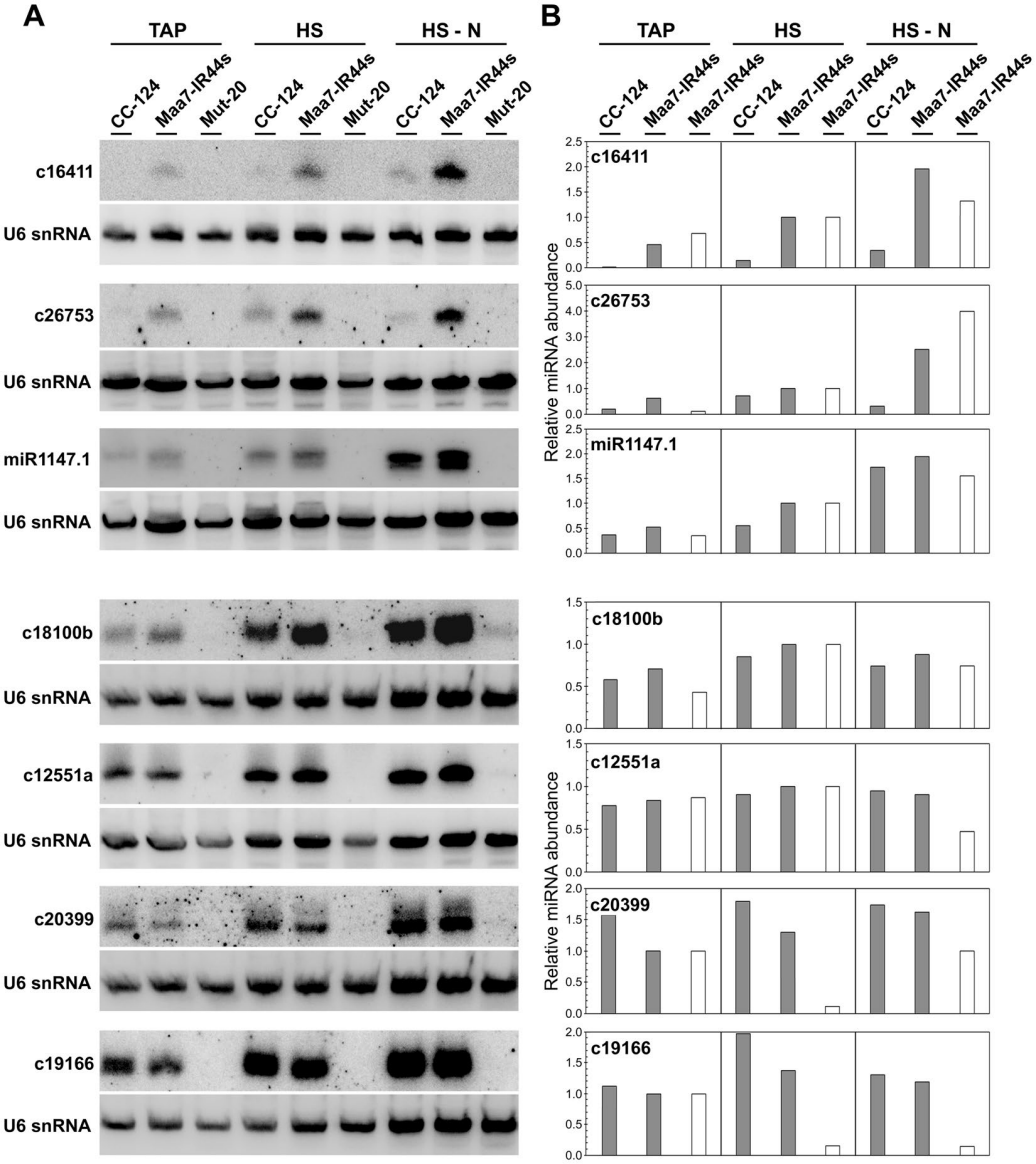
Northern blot analysis of miRNA expression in Chlamydomonas cells grown under the denoted trophic conditions. (**A**) Small RNAs were detected with probes specific for the indicated miRNAs. The same filters were reprobed with the U6 small nuclear RNA sequence as a control for lane loading. CC-124 wild type strain; Maa7-IR44s, CC-124 containing a transgene expressing FLAG-tagged AGO3; Mut-20, *TSN1* deletion mutant, in the Maa7-IR44s background, defective in sRNA biogenesis^14^. (**B**) Relative miRNA levels in the indicated strains under the different trophic conditions. Values shown are the average of two independent experiments and are normalized to those of the Maa7-IR44s strain grown photoautotrophically in nutrient replete minimal medium (HS). For c20399 and c19166, values are normalized to those of Maa7-IR44s grown mixotrophically in acetate containing medium (TAP). The relative standard deviation, as percentage of the mean, was in no case higher than 28.3%. Data corresponds to phosphorimager measurements of sRNA signals on northern blots (gray bars) or normalized read counts from the AGO3-associated sRNA libraries (white bars).

The abundance of AGO3-associated miRNAs identified in Chlamydomonas grown under multiple trophic conditions (*i.e*., the 32 miRNAs common to all three conditions examined, Fig. 1A) remained relatively constant or differed depending on the nutritional regime (Table S1A). The expression of a subset of these miRNAs was validated by northern blot analyses of the Maa7-IR44s strain (containing the FLAG-tagged AGO3 protein), the parental strain CC-124, and a previously described mutant strain, Mut-20^14^, virtually devoid of small RNAs (Fig. 2A). The U6 snRNA, whose abundance remains fairly stable under the examined conditions (Fig. S2), was used as a loading control. Even though the RNA blots measure total cellular miRNA abundance whereas the libraries reflect AGO3-associated miRNA abundance, there was reasonable agreement between the two techniques for most miRNAs examined (Fig. 2B). The main exceptions were c20399 (miR_t20) and c19166 (miR_t124) which showed decreased abundance in the libraries from one or both photoautotrophic conditions relative to that from the mixotrophic condition whereas in the northern blots their steady-state levels remained relatively constant (Fig. 2B). Additionally, the Maa7-IR44s and CC-124 strains generally showed comparable miRNA levels, although unexpected differences were also observed for a few miRNAs (Fig. 2, c16411 and c26753).

To extend the pool of potentially functional miRNAs related to nutritional stress responses in *C. reinhardtii*, we also re-analyzed the sRNA data published by Chávez Montes *et al*.^25^. However, the libraries in their study were generated from total cellular small RNAs (under nutrient-replete, phosphate-deprived, or sulfur-deprived conditions), rather than from AGO3-associated sRNAs, and only 17 sequences met the criteria^14, 29^ to be classified as canonical miRNAs in our analyses (Fig. 1B and Table S1B). This limited dataset nonetheless suggests that the *C. reinhardtii* miRNA population also varies among cells grown under phosphate or sulfur starvation although, as discussed above, when considering read abundance virtually none of the identified miRNAs is truly condition-specific (Table S1B). The expression of a subset of miRNAs in cells grown under phosphate or sulfur deprivation was also examined by RNA blotting and hybridization, but there was relatively poor agreement between the northern blotting signals (Fig. S3A) and the total library read counts (Fig. S3B). The studied cells did experience the expected nutritional deficiency, as indicated by the upregulation of diagnostic genes such as *PHO5*, encoding a phosphate-repressible alkaline phosphatase, and *SLT1* (*SAC1-LIKE TRANSPORTER1*), encoding a sodium/sulfate cotransporter (Fig. S4). Thus, the poor correlation in miRNA abundance between northern blotting and library read counts might be due to the fact that different Chlamydomonas strains were used for the analyses and/or technical issues (see Discussion). Nevertheless, our observations, taken together, indicate that several miRNAs are differentially expressed in response to nutrient depletion in Chlamydomonas, although very few (if any) appear to be strictly condition-specific.

### Predicted miRNA targets in *C*. *reinhardtii* under various trophic conditions

**Figure 3:**
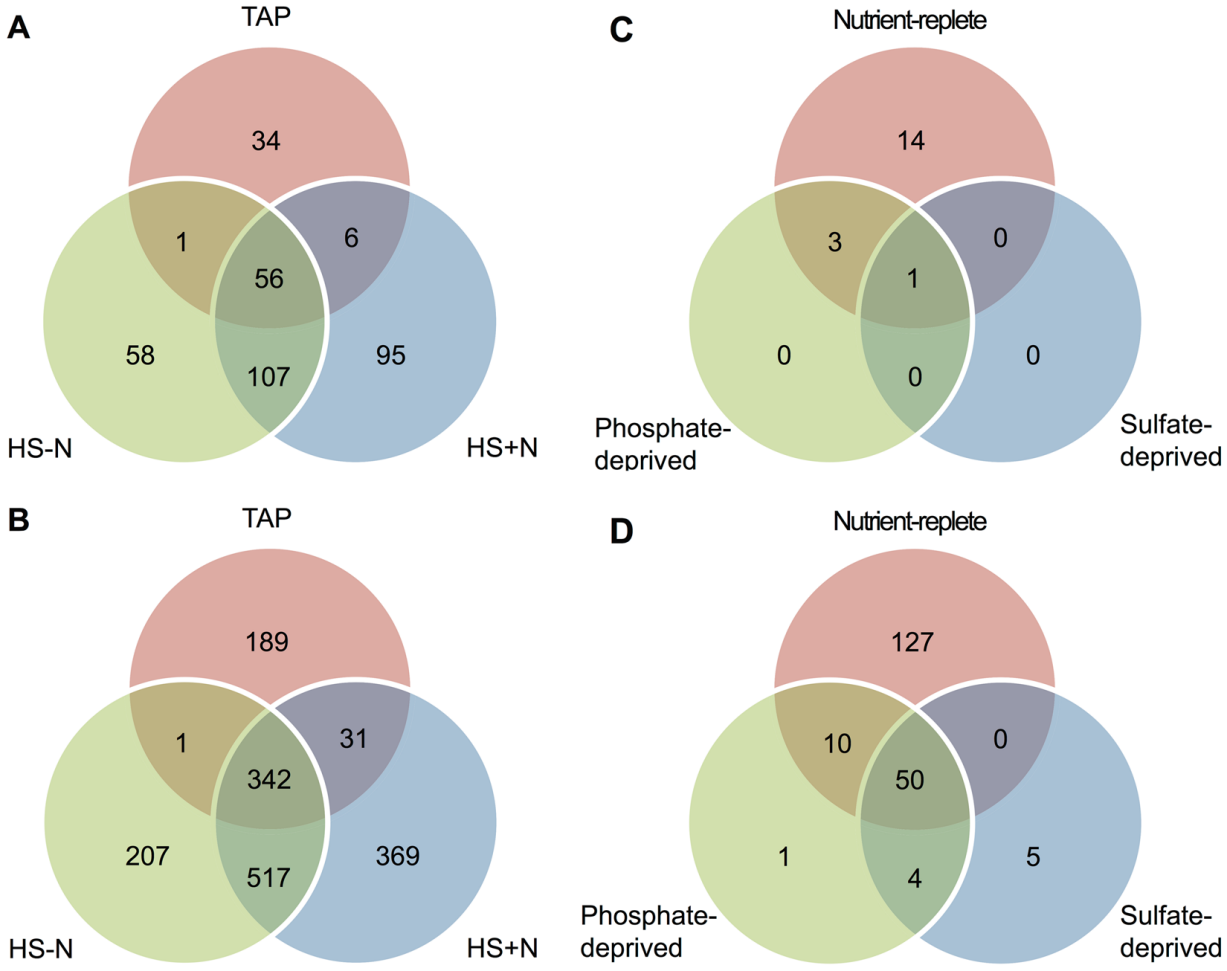
Comparison of predicted miRNA targets in Chlamydomonas cells grown under various nutritional deprivation conditions. Venn diagrams show the numbers of unique and shared putative miRNA targets in cells grown under the different trophic regimes. (**A**) and (**C**), Predicted cleavage targets. (**B**) and (**D**), Predicted translation repression targets. MicroRNA targets were computationally predicted (see Methods) based on the miRNAs identified from the AGO3-associated sRNA libraries in this study (**A** and **B**) and from the total sRNA libraries prepared by Chávez Montes *et al*.^25^ (**C** and **D**).

As previously described^14^, potential miRNA targets were predicted based on sequence complementarity between miRNAs and binding sites on transcripts. In addition, predicted targets were classified, depending on the extent of complementarity to a miRNA, as likely to be regulated via transcript cleavage or translation repression^14^. As expected, changes in miRNA populations associated with each growth condition resulted in the prediction of distinct target gene populations (Fig. 3).

To begin assessing whether miRNAs may play a regulatory role in the response to nitrogen starvation, we examined in more detail the predicted targets of the 20 miRNAs that were more prevalent under nitrogen-deprived photoautotrophic conditions (Fig. 1A, HS − N) as well as those of the 14 miRNAs that were identified primarily in nutrient replete photoautotrophic conditions (Fig. 1A, HS + N). The miRNAs characteristic of HS − N had 58 putative cleavage targets and 207 translation repression targets whereas the miRNAs typical of HS + N potentially regulated 95 cleavage targets and 369 translational repression targets (Fig. 3A and B, and Table S1A). However, the vast majority of the predicted targets corresponds to genes with unknown function and very few of those with an annotated function(s) code for proteins presumably involved in direct responses to nutrient deficiency (Table S2). Instead they appear to have a wide variety of cellular roles, including flagellar associated proteins, molecular chaperones, protein kinases, post-translational modification proteins, predicted extracellular polypeptides and a few transcription factors (Table S2).

Transcriptome profiling revealed that of the 58 predicted cleavage targets for miRNAs more abundant in HS − N grown cells, only two showed at least a 2-fold decrease in steady-state mRNA levels under nitrogen starvation and a concomitant up-regulation in the miRNA-deficient Mut-20, as expected for true cleavage targets (Table S2, *Cre18.g749747* and *Cre06.g303200* highlighted in yellow). Likewise, of the 95 predicted cleavage targets for the 14 HS + N prevalent miRNAs, two displayed at least a 2-fold increase in expression in Mut-20 (Table S2, *Cre12.g552950* and *Cre16.g674291* highlighted in yellow). However, only one of these potential targets, *Cre12.g552950*, was differentially expressed during nitrogen starvation. Additionally, of the 207 potential translational repression targets for miRNAs more abundant in HS − N, only three showed a ≥2-fold change in transcript levels under nitrogen starvation as well as in the miRNA deficient Mut-20 strain (Table S2, *Cre14.g627411*, *Cre07.g338000* and *Cre10.g464100* highlighted in yellow). Similarly, of the 369 predicted translational repression targets for the 14 miRNAs identified in HS + N, only 8 showed at least a 2-fold increase in transcript abundance in the miRNA deficient Mut-20 (Table S2, targets highlighted in yellow). Nonetheless, because miRNA regulation by translation inhibition does not necessarily alter the steady-state level of target transcripts^14, 16, 17^, further analyses of protein abundance would be necessary to verify potential translation repression targets.

The transcript abundance of three predicted miRNA cleavage targets, up-regulated in the RNA-seq experiments with Mut-20, was also verified by qRT-PCR analyses in Mut-20 and in a strain defective in AGO3, ago3-1^28^ (see below), in comparison with their parental strains (Fig. S5). We examined the putative targets of two miRNAs expressed at high levels (*Cre04.g227600* target of c12364 and *Cre06.g249550* target of c18100a) as well as the predicted target of one miRNAs expressed at low levels (*Cre12.g552950* target of miR_t70). *Cre12.g552950* behaved as a genuine cleavage target, with increased transcript abundance in both RNAi defective strains (Fig. S5A). However, the *Cre12.g552950* mRNA is perfectly complementary to several sRNAs present in the libraries (including putative endogenous small interfering RNAs) (Fig. S5B) and its steady state level may be modulated by the combined action of multiple sRNAs rather than solely by the lowly expressed miR_t70. *Cre04.g227600* and *Cre06.g249550* showed increased transcript levels only in the Mut-20 strain (Fig. S5A). Interestingly, the c12364 and c18100a miRNAs are moderately reduced in abundance in ago3-1 (30–40% of wild type levels) and they may still be able to suppress *Cre04.g227600* and *Cre06.g249550* expression in this strain, in conjunction with Chlamydomonas AGO1 or AGO2^28^. Of note, transcript abundance of all three target genes appears to be only modestly affected by the action of miRNAs/sRNAs (Fig. S5A).

For the five miRNAs that were recognized preferentially during phosphate- and/or sulfur-deprivation (Fig. 1B), we identified no potential cleavage targets and only 10 potential translational repression targets (Fig. 3C and D, Table S1B), which were not differentially expressed under the conditions examined. Conversely, for the 7 miRNAs that were preferentially identified in total sRNA libraries from the nutrient-replete condition (Fig. 1B), we predicted 14 potential cleavage targets and 127 potential translational repression targets (Fig. 3C and D, Table S1B). Of these putative targets, only one predicted cleavage target and three predicted translational repression targets were differentially expressed during phosphate- and/or sulfur-deprivation (Table S3). However, none of these genes codes for a protein involved in nutrient assimilation/metabolism and their putative role(s) in response to phosphate- and/or sulfur-deficiency is not clear. Moreover, in most cases, the changes in transcript abundance observed under nutrient deprivation (Table S3) were not in the expected direction based on the changes in abundance of the corresponding targeting miRNAs (Table S1B).

In summary, it remains uncertain how many of the predicted miRNA targets (under any of the examined trophic conditions) are genuine. However, even if some (many) predictions correspond to false positives, our observations strongly suggest that most targets in *C. reinhardtii* do not appear to be subject to miRNA-triggered transcript degradation, as reflected by the lack of changes in mRNA abundance in response to changes in miRNA abundance (in an sRNA-defective mutant strain or in cells exposed to various nutritional regimes inducing differential miRNA expression). As discussed below, we hypothesize that many Chlamydomonas miRNAs may be expressed at too low levels, under any trophic condition, to be functionally effective and those expressed at higher levels appear to have few, if any, targets.

### Cell growth and survival of RNAi-defective *C. reinhardtii* strains under nutrient deprived conditions

**Figure 4:**
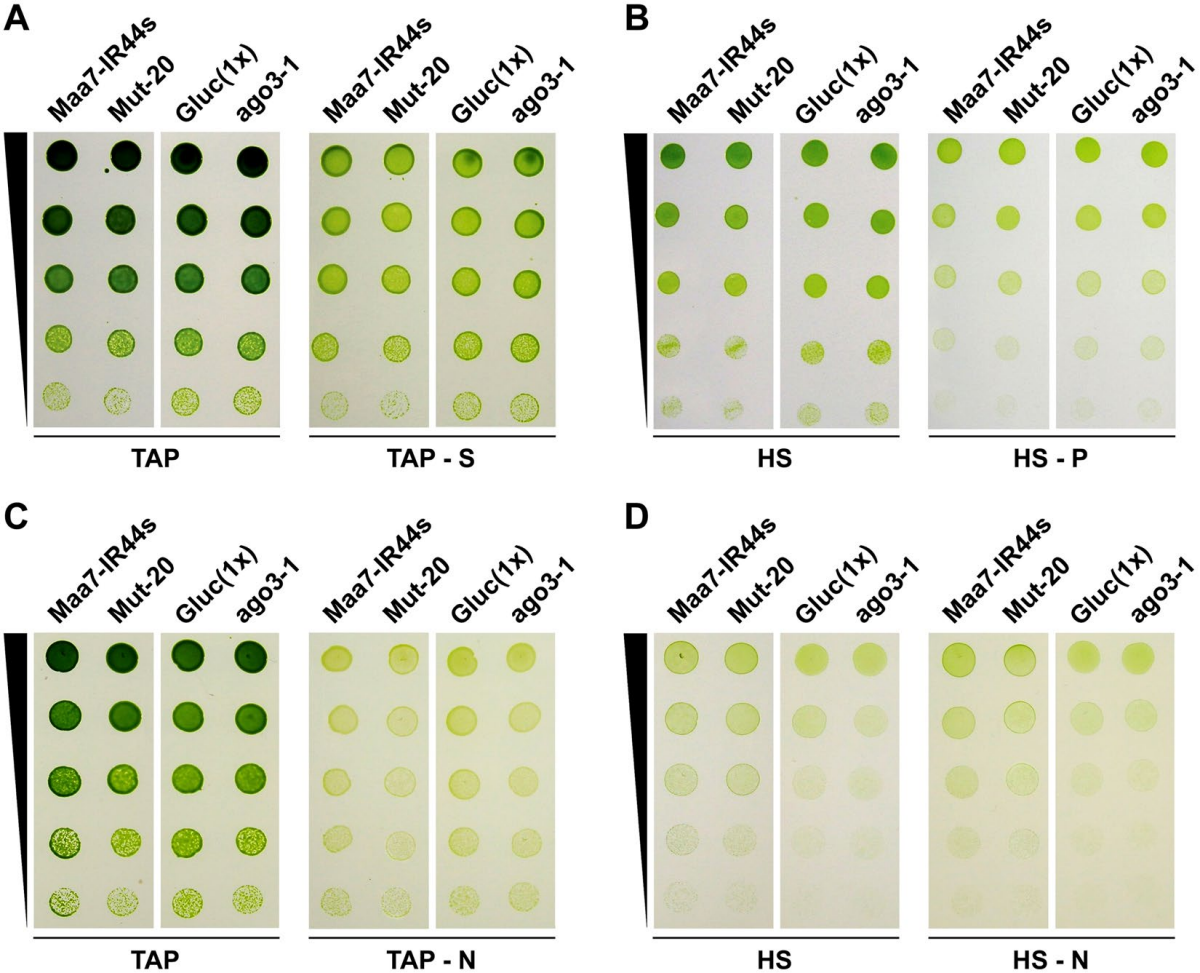
Growth and survival of Chlamydomonas cells subjected to various nutritional deprivation conditions. Cells grown to logarithmic phase in TAP medium were serially diluted in water, 5 μl-aliquots spotted on plates of the appropriate media and incubated for 7 to 15 days under continuous illumination. Maa7-IR44s, CC-124 strain containing a transgene expressing FLAG-tagged AGO3; Mut-20, *TSN1* deletion mutant, in the Maa7-IR44s background, defective in sRNA biogenesis^14^; Gluc(1x), wild type strain derived from CC-124; ago3-1, *AGO3* disrupted mutant, in the Gluc(1x) background, defective in RNA interference^28^. (**A**) Cells grown under mixotrophic conditions in the presence or absence of sulfur. (**B**) Cells grown under photoautotrophic conditions in the presence or absence of phosphorus. (**C**) Cells grown under mixotrophic conditions in the presence or absence of nitrogen. (**D**) Cells grown under photoautotrophic conditions in the presence or absence of nitrogen.

To examine further whether miRNAs may play a role in responses to nutrient depletion (and by inference in endogenous gene regulation), we tested the growth and survival of several RNAi defective strains under different trophic conditions. We assayed Mut-20, which contains a deletion of the gene coding for TUDOR STAPHYLOCOCCAL NUCLEASE1 (TSN1), implicated in sRNA biogenesis^14^, and its parental strain Maa7-IR44s. We also analyzed ago3-1, which contains a disrupted *AGO3* gene, and its parental strain Gluc(1x)^28^. Mut-20 is virtually devoid of small RNAs^14^ whereas ago3-1 has reduced levels of a subset of miRNAs and defects in sRNA mediated post-transcriptional gene silencing^28^. Nonetheless, the growth of the mutant strains, under a variety of nutrient depletion conditions, was very similar to that of the parental strains (Fig. 4).

**Figure 5:**
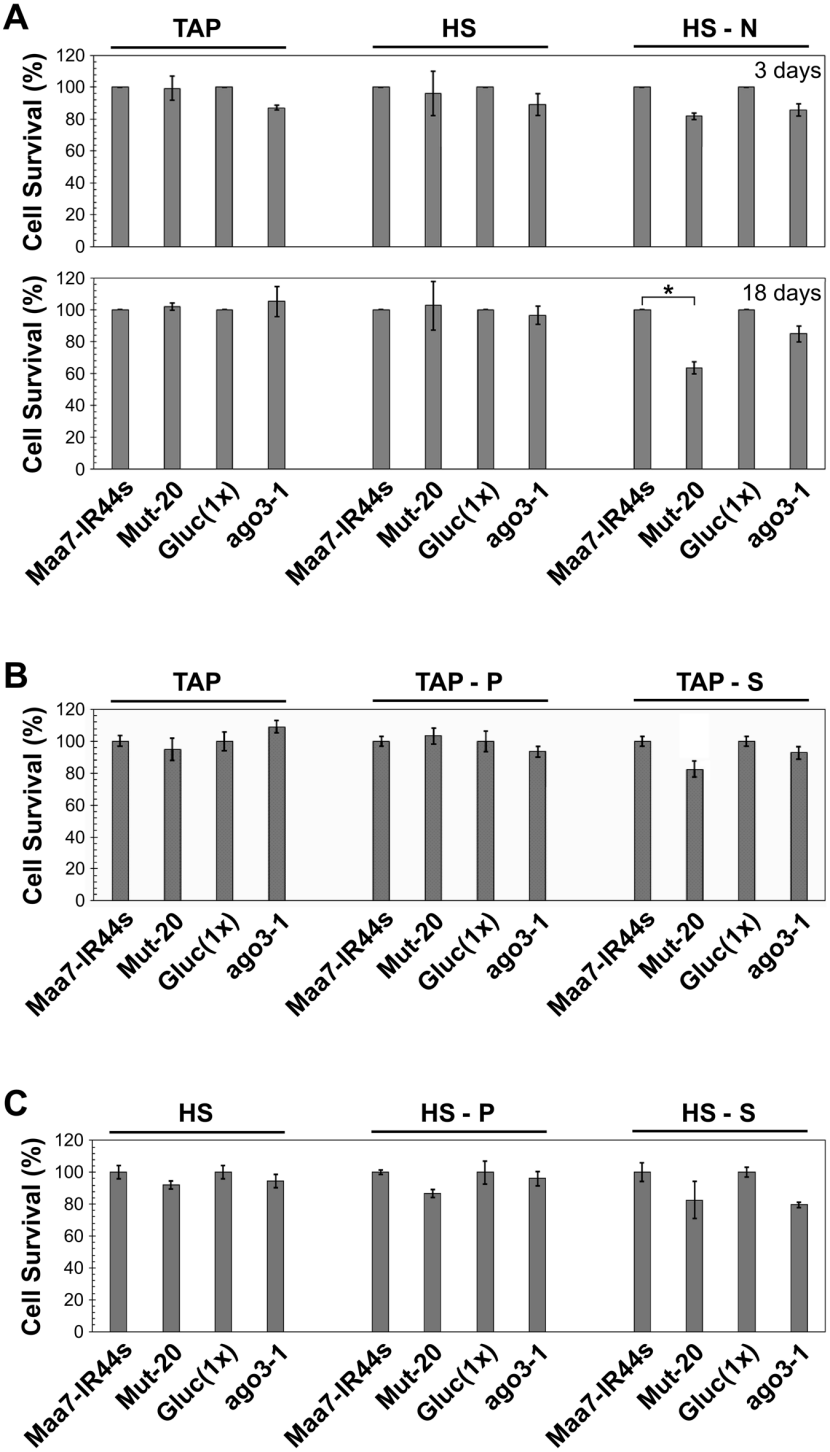
Viability of wild-type and RNA interference defective strains subjected to nutrient deprivation conditions. Cells were cultured in liquid medium, either replete or lacking a specific nutrient, for certain number of days and then spread on TAP-agar plates to assess survival as colony forming units. Values shown are the average of three independent experiments ± SD and are normalized to those of the control strains under each trophic condition. Maa7-IR44s, CC-124 strain containing a transgene expressing FLAG-tagged AGO3; Mut-20, *TSN1* deletion mutant, in the Maa7-IR44s background, defective in sRNA biogenesis^14^; Gluc(1x), wild type strain derived from CC-124; ago3-1, *AGO3* disrupted mutant, in the Gluc(1x) background, defective in RNA interference^28^. (**A**) Cell survival of the indicated strains grown mixotrophically (TAP) or photoautotrophically in the presence (HS) or absence (HS − N) of nitrogen for 3 or 18 days. Samples marked with an asterisk are significantly different (*p* < 0.05) in a two tailed Student’s t-test. (**B**) Cell survival of the indicated strains grown mixotrophically for 18 days in nutrient replete medium (TAP) or lacking phosphorus (TAP-P) or sulfur (TAP-S). (**C**) Cell survival of the indicated strains grown photoautotrophically for 18 days in nutrient replete medium (HS) or lacking phosphorus (HS-P) or sulfur (HS-S).

We also examined cell survival after subjecting the strains to prolonged nitrogen-, phosphate- or sulfur-deprivation. Yet, for the most part, the mutants behaved like the wild type strains (Fig. 5). The only significant difference was a moderate decrease in the survival of Mut-20, relative to Maa7-IR44s, upon exposure to nitrogen-depleted medium under photoautotrophic conditions (Fig. 5A). However, it is not certain whether this reduced survival is due to a defect in miRNA-mediated gene regulation or caused by a deficiency in other pleotropic functions of the deleted TSN1 protein^14^, particularly since ago3-1 survival was not meaningfully compromised by nitrogen deprivation (Fig. 5A). Thus, the lack of major phenotypic defects associated with disruption of the RNAi machinery (see also Valli *et al*.^30^) suggests a rather limited, modulatory role of miRNA-mediated gene regulation in Chlamydomonas cells cultured under nutrient deprived conditions.

### Features of AGO3-associated *C. reinhardtii* miRNAs expressed under various trophic conditions

**Figure 6:**
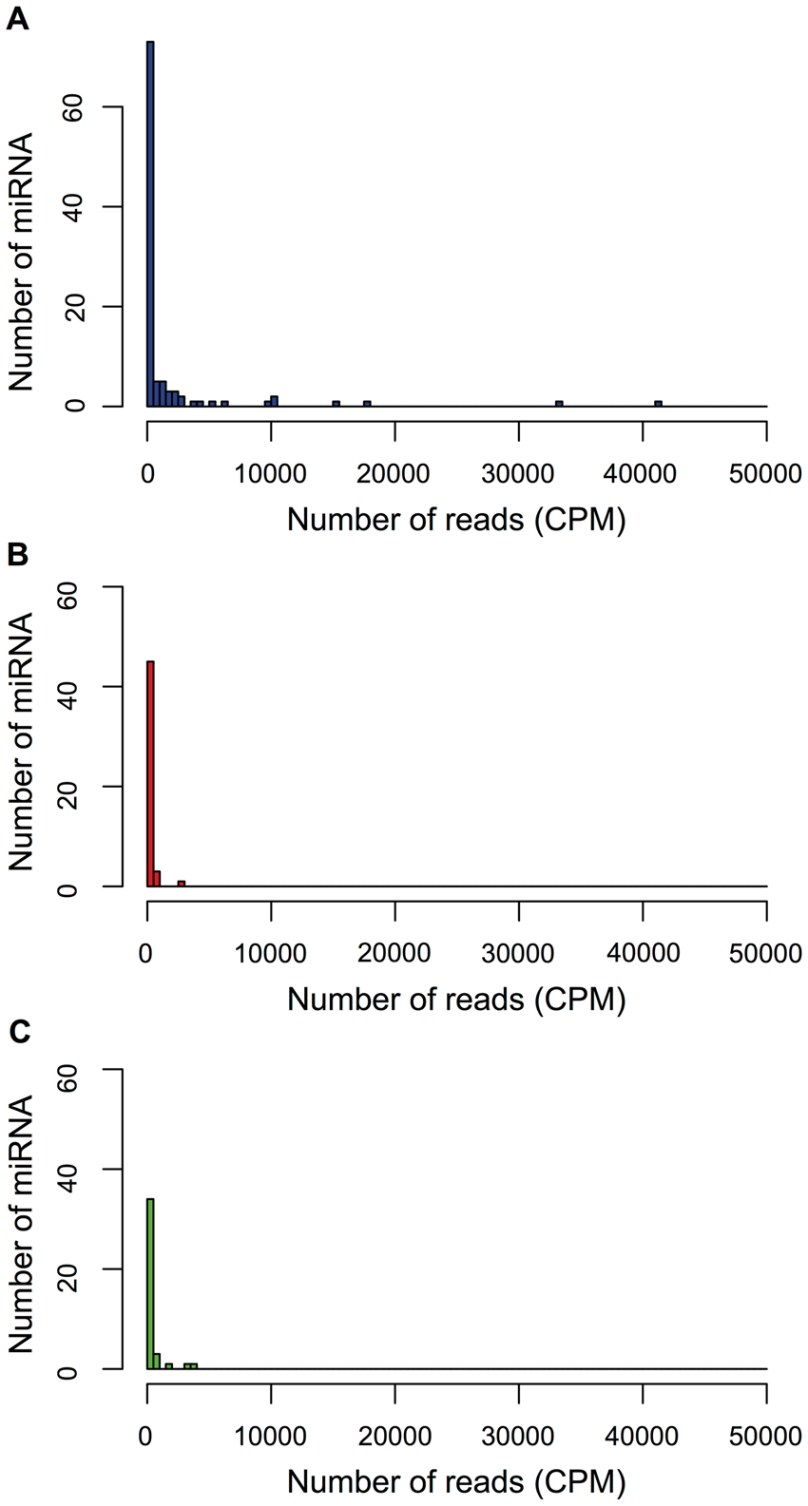
Distribution of miRNAs based on their average expression levels for *C. reinhardtii* (**A**) and for the species-specific miRNAs in *Arabidopsis thaliana* (**B**) and *Arabidopsis lyrata* (**C**). Binning was done with 500 CPM (Counts Per Million mapped reads) intervals.

**Figure 7:**
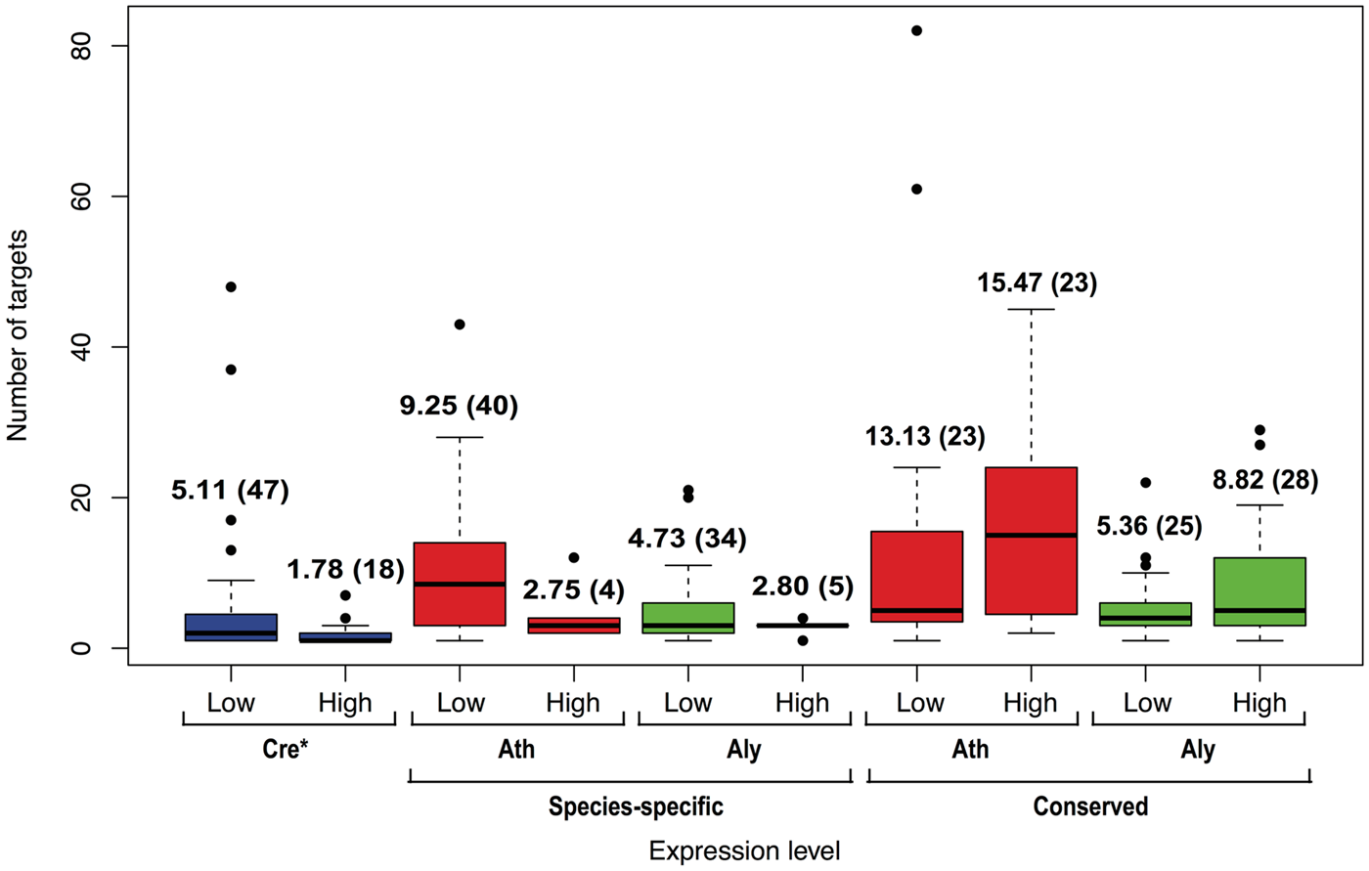
Comparison of the number of predicted cleavage targets for lowly expressed and highly expressed miRNAs. Boxplots show the number of predicted cleavage targets for miRNAs with low expression (<500 CPM) and high expression (≥500 CPM) identified in the AGO3-pulldown libraries from *C. reinhardtii* (blue) and for species-specific or conserved miRNAs from *Arabidopsis thaliana* (red) or *Arabidopsis lyrata* (green). Conserved miRNAs are shared between *A. thaliana* and *A. lyrata* and many have experimentally validated roles. MicroRNAs that had no predicted targets were excluded from this plot. The average number of targets (including outliers) and the sample size for each category are shown above the boxes. The target numbers were compared between the two expression-level groups for each species and an asterisk next to the species abbreviation indicates a significant difference (*p* < 0.05 by Wilcoxon rank sum test).

Many of the identified miRNAs have relatively low levels of expression (on average <500 Counts Per Million mapped reads or CPM) (Fig. 6A, Table S1A). These miRNAs also tend to have a larger number of predicted targets than those with higher expression (Fig. S6). In the most extreme case, a lowly expressed miRNA (miR_t69) was predicted to have 48 cleavage targets and 242 translational repression targets (Table S1A). When comparing low expression miRNAs (average expression <500 CPM) classified as miRNAs only in cells grown under a certain nutritional regime (TAP, HS + N or HS − N) with high expression miRNAs (average expression ≥500 CPM) shared under all nutritional conditions, the average number of predicted targets per miRNA was significantly different (Fig. 7). The lowly expressed miRNAs have an average of 5.11 (n = 47) predicted cleavage targets per miRNA, whereas highly expressed miRNAs have an average of 1.78 (n = 18) predicted cleavage targets per miRNA (*p* = 0.0156 by Wilcoxon rank sum test, Cohen’s *d* = 0.538) (Fig. 7). This trend was also observed for predicted translational repression targets, but with smaller (non-significant) differences.

**Figure 8:**
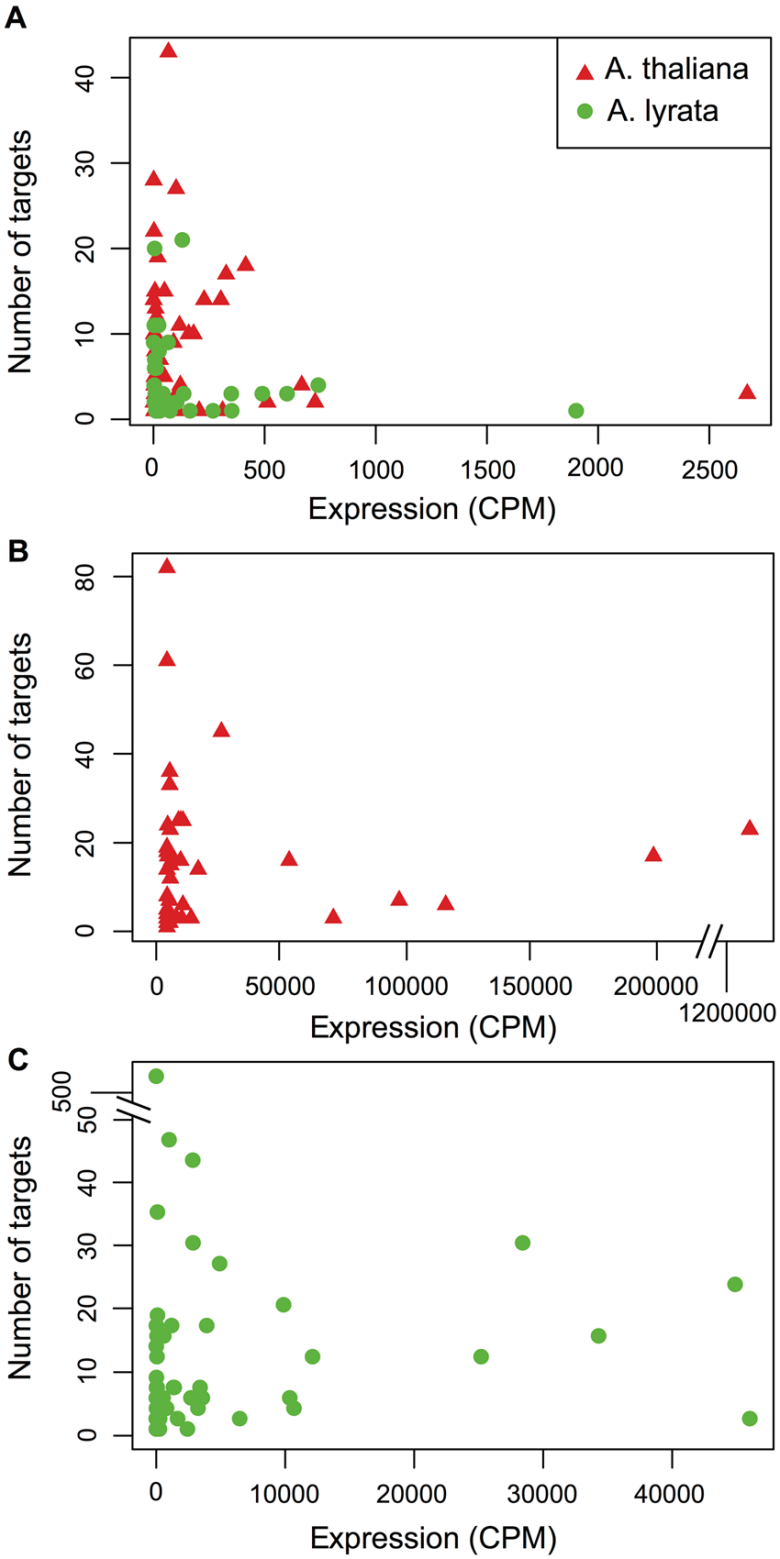
Relationship between miRNA expression level and number of predicted targets in *Arabidopsis thaliana* and *Arabidopsis lyrata*. Scatter plots show the expression level and the number of predicted targets for each miRNA in *A. thaliana* (red) and *A. lyrata* (green). (**A**) Species-specific miRNAs in both Arabidopsis species. (**B**) Conserved miRNAs in *A. thaliana*. (**C**) Conserved miRNAs in *A. lyrata*.

Because non-conserved, recently evolved miRNAs in higher plants tend to have low expression levels^24–26, 31, 32^, similarly to many Chlamydomonas miRNAs, we performed equivalent analyses on miRNAs specific to either *A. thaliana* or *A. lyrata*. Most species-specific miRNAs in each *Arabidopsis* species are lowly expressed (<500 CPM) (Fig. 6B and C). They also tend to show an inverse relationship between miRNA expression level and number of predicted targets (Figs 7 and 8A). The difference was more prominent in *A. thaliana*, with an average of 9.25 (n = 40) predicted cleavage targets per lowly expressed miRNA and only 2.75 (n = 4) predicted cleavage targets per highly expressed miRNA (Fig. 7). However, likely due to the small number of highly expressed miRNAs, this difference was not statistically significant (*p* = 0.075 by Wilcoxon rank sum test). A similar trend was observed in *A. lyrata*, with an average of 4.73 (n = 34) predicted cleavage targets per lowly expressed miRNA and 2.80 (n = 5) predicted cleavage targets per highly expressed miRNA (Fig. 7) although, as in *A. thaliana*, the difference was not statistically significant (*p* = 0.915 by Wilcoxon rank sum test). In contrast, miRNAs that are conserved between *A. thaliana* and *A. lyrata* (many with experimentally validated roles) are expressed at higher levels^26, 32^ and have on average a greater number of predicted cleavage targets per miRNA (Figs 7 and 8B,C). Moreover, by target degradome sequencing, nearly all verified cleavage targets in *A. thaliana* and *A. lyrata* were those corresponding to conserved miRNAs^26^.

We also examined conservation of the Chlamydomonas miRNAs (Table S1) against all miRNAs (either mature or stem-loop sequences) deposited in miRBase^33^. The search revealed no significant hits to miRNAs from any organism aside from *C. reinhardtii* (see Methods). Previous studies also showed that the miRNAs identified in Chlamydomonas had no meaningful similarity to mature miRNA sequences even from the alga *Volvox carteri* ^14, 18, 19, 34^, the closest relative to *C. reinhardtii* for which sRNAs and miRNAs have been extensively profiled. Thus, Chlamydomonas miRNAs are not evolutionarily conserved and many show relatively low expression levels (particularly those identified primarily under certain nutritional conditions) as well as negative correlation between miRNA abundance and number of predicted targets. These features largely resemble those of the species-specific, recently evolved miRNAs characterized in higher plants^24–26, 31, 32^.

## Discussion

Comparison of AGO3-associated sRNA populations from Chlamydomonas cells grown under various trophic conditions revealed that some miRNAs are differentially expressed in response to nutritional changes, but none of the identified miRNAs appears to be strictly condition specific (Table S1A). The re-analysis of total sRNA libraries prepared by Chávez Montes *et al*.^25^ from *C. reinhardtii* cultured under nutrient replete, phosphate-deprived or sulfur-deprived conditions also supported the existence of differentially expressed miRNAs as a result of nutrient depletion (Table S1B). Changes in steady-state levels for a subset of the miRNAs were corroborated by northern blot analyses (Figs 2 and S3), although there was better agreement, with some exceptions, between northern blot signals and normalized read counts from the AGO3-associated sRNA libraries than from the total sRNA libraries. This may be explained by experimental variation since different Chlamydomonas strains were used in the latter comparison (see Methods and Chávez Montes *et al*.^25^).

As previously demonstrated, the representation of miRNA sequences relative to each other within a sRNA library may not be consistent with their input concentrations, because of biases in ligation-based small RNA library construction due to adaptors, RNA structure, and RNA ligase activity^35–38^. On the other hand, this problem is expected to be less relevant (*i.e*., a systematic bias) for relative abundance comparisons of the same miRNA across libraries prepared in the same way. Nonetheless, for some miRNAs this may still be a problem since their relative levels in sRNA libraries from various trophic conditions differed substantially from their detection by northern blotting. From a technical perspective, it seems clear that library construction (either from total or AGO-associated sRNAs) does affect the identification of potentially functional miRNAs. However, our combined observations, based on the analyses of multiple sRNA libraries as well as northern blotting, strongly support that changing nutritional conditions induces the differential expression of a subset of miRNAs in *C. reinhardtii*.

Since AGO3 is the main effector of sRNA-mediated post-transcriptional gene silencing in Chlamydomonas^28^, changes in AGO3-associated miRNAs may potentially be of functional relevance in responses to nutritional stress. Thus, we surveyed the putative role(s) of computationally predicted targets for the 20 miRNAs that were more prevalent under nitrogen-deprived photoautotrophic conditions (Fig. 1A, HS − N) and for the 14 miRNAs that were identified preferentially in nutrient replete photoautotrophic conditions (Fig. 1A, HS + N). However, most predicted targets corresponded to genes with unknown function, and virtually none of those with an annotated function(s) encoded a protein directly involved in nitrogen metabolism/assimilation (Table S2). Additionally, transcriptome profiling of cells cultured in nutrient-replete or in nitrogen-deprived media as well as of a mutant strain, Mut-20, virtually devoid of sRNAs revealed that very few of the putative miRNA targets showed changes in transcript abundance consistent with their regulation by miRNA-mediated RNA degradation (Table S2). Similar observations were made for cells grown under phosphate- or sulfur-deprived conditions (Table S3).

The Chlamydomonas RNAi machinery has the capability to operate by target transcript cleavage, as demonstrated with artificial miRNA transgenes^16, 28, 39, 40^. However, with the caveat that some predictions may represent false positives, most endogenous miRNA targets do not appear to be subject to transcript cleavage and degradation in cells cultured under multiple trophic conditions. A similar conclusion was reached by Valli *et al*.^30^ through the analysis of a Chlamydomonas mutant defective in DICER LIKE3 (DCL3), which failed to produce both miRNAs and siRNAs. Nonetheless, the Chlamydomonas RNAi machinery also has the capability to cause translation repression of target transcripts, as demonstrated with transgenic constructs^16, 17, 28^. MicroRNAs could also exert regulatory roles on host transcripts in *cis*, simply by being processed by Dicer, since several Chlamydomonas miRNAs are derived from mRNAs of hypothetical protein coding genes^14, 30^.

Conceivably, miRNAs differentially expressed under various trophic conditions could play an important role in responses to nutritional stress, via modulation of target translation efficiency, the stability of some host transcripts or even some unconventional mechanisms^7, 41–43^. However, this interpretation is not supported by phenotypic analyses of strains defective in components of the RNAi machinery. Mut-20 is virtually devoid of sRNAs^14^ whereas ago3-1 has reduced levels of a subset of miRNAs and defects in sRNA mediated post-transcriptional gene silencing^28^. Despite these major alterations to the RNAi machinery both mutants grew as well as the parental strains under mixotrophic or photoautotrophic conditions, in liquid or solid medium, and under various nutrient deprived conditions (Fig. 4). As already mentioned, even the moderate decrease in Mut-20 survival after prolonged exposure to nitrogen depleted minimal medium (Fig. 5A) cannot be unequivocally ascribed to a defect in miRNA-mediated gene regulation. Likewise, the Chlamydomonas *dcl3* mutant did not show obvious alterations in growth or morphological abnormalities under normal laboratory conditions^30, 44^. Moreover, ribosome profiling and proteomic analyses in the wild type and the *dcl3* mutant indicated that miRNAs have little effect on translation efficiency and largely fine tune target gene expression^44^. Thus, while a recent report proposed that certain miRNAs play a key role in abiotic stress responses in *C. reinhardtii* ^10^, accumulating evidence suggests that most miRNAs mainly have a modulatory, rather modest function in the regulation of biological processes in this alga (at least under normal and nutrient deprived growth conditions). This hypothesis is very difficult to demonstrate conclusively since it can only be supported by negative data (*i.e*., the lack of a verified miRNA function) but it seems the most parsimonious explanation for the collective results of us and others^14, 28, 30, 44^.

In addition, Chlamydomonas miRNAs are not evolutionarily conserved even within the order Volvocales, which includes the related alga *Volvox carteri* ^14, 18, 19, 34^. Chlamydomonas and Volvox lineages diverged ~200 million years ago^45^ and extensive sequence divergence over this length of evolutionary time may have obscured miRNA homologies. However, this seems unlikely to be the case for all miRNA loci, since subsets of both animal and land plant miRNAs have been strongly conserved over a similar period of time^20, 25, 29, 32, 46, 47^. Thus, Chlamydomonas miRNAs appear to have evolved relatively recently, since the divergence from the lineage leading to the family Volvocaceae. Moreover, many Chlamydomonas miRNAs, particularly those identified preferentially under certain nutritional conditions, are expressed at relatively low levels (Table S1A and Fig. 6A) and show negative correlation between miRNA abundance and number of predicted targets (Figs 7 and S6). Many condition-prevalent miRNAs also seem to show imprecise processing from fairly long hairpin precursors (Fig. S1), which is reflected in lower predominance of the reads corresponding exactly to the mature miRNAs, representing <90% of all the reads mapping locally to the precursor hairpins. These features resemble those of the species-specific, newly evolved miRNAs characterized in land plants^24–26, 31, 32, 47, 48^.

Recent findings in metazoans suggest that only strongly expressed miRNAs, above a certain threshold level, may lead to functionally significant target suppression. By using a sensor library to monitor miRNA activity in human monocytes, only miRNAs expressed above 100–1000 reads per million showed suppressive activity^49^. High miRNA abundance might be necessary to facilitate miRNA interaction with target transcripts, through diffusion and sampling within a cell, although the extent of suppression also depends, among other variables, on target site concentration^49–51^. Assuming a similar expression threshold for functional miRNAs in Chlamydomonas, we hypothesize that over 60% of the AGO3-associated miRNAs (detected at <500 CPM under any condition, Table S1A) would not be expected to have discernable activity. This is consistent with expectations for young miRNAs since an initial weak expression and negligible fitness effects would allow their progressive integration into gene regulatory networks^32, 48, 52^.

The RNAi machinery presumably arose as an ancestral defense mechanism against selfish genetic elements such as viruses and transposons^53–55^ and was later co-opted to miRNA pathways that evolved independently in several eukaryotic lineages^2, 3, 47, 54, 55^. As proposed in a number of organisms^2, 20, 32, 47, 52^, low level transcription of inverted repeats or mutationally engendered hairpin structures could give rise to a diversity of RNAs recognized as substrates by the sRNA biogenesis machinery. However, most young miRNAs would likely be neutral^32, 47, 48, 52^, either by not being expressed at a high enough level or by not having enough sequence identity to regulate any meaningful target. Random mutations and genetic drift would lead to the relatively rapid evolutionary turnover of these miRNA precursor genes. In contrast, miRNAs that acquire a target with functional relevance would be maintained under purifying selection and could increase, over time, their expression and even acquire additional targets to enable more efficient gene regulation^52^. Accordingly, in both land plants and animals, conserved, older miRNAs are generally expressed at higher levels and have more targets than young ones^23–26, 32, 47, 48, 52^.

The eukaryotic groups that exhibit the highest level of multicellular complexity (animals and land plants) all possess miRNAs^2, 20, 25, 29, 46, 47, 55^ that regulate important biological processes, including cell differentiation and development^1, 3, 7, 32, 46, 47^. This correlation has led several authors to propose that miRNAs may have played a role in the evolution of complex multicellularity^55–57^. In contrast, in the unicellular alga *C. reinhardtii* the miRNA system appears to consist largely of recently evolved miRNAs that, based on the RNAi-defective mutant phenotypes, do not seem to play a substantial role in cell growth and survival (at least under the trophic conditions examined). Indeed, Chlamydomonas miRNAs do not appear to have been meaningfully integrated yet into the organism’s gene regulatory network. Even in eukaryotes at an early transition towards a multicellular stage, such as *Volvox carteri* and *Dictyostelium discoideum*, the role of miRNAs in controlling gene expression is elusive^19, 58^. For instance, *D. discoideum drnB* ^*−*^ mutant cells, lacking a Dicer-like protein required for miRNA biogenesis, grow and develop normally^58^. Thus, it is tempting to speculate that in unicellular eukaryotes, miRNAs arising as accidental products of random genome evolution may provide no major selective advantage for the regulation of essential cellular functions, ancestrally controlled by other components such as transcription factors. Most miRNAs in these organisms may be transient, without a main biological utility, but may provide a pool from which new miRNA-target regulatory interactions could eventually be recruited leading to evolutionary innovations.

## Methods

### Strains, mutants, and culture conditions

Chlamydomonas cells were grown mixotrophically in TAP medium^17^ or photoautotrophically in high salt (HS) medium^59^. For nitrogen deprivation analyses, cells initially grown photoautotrophically in nutrient replete medium to the middle of the logarithmic phase were collected by centrifugation and resuspended at a density of ~1.0 × 10^6^ cells mL^−1^ in the same medium with (HS + N) or without nitrogen (HS − N). After 72 h of incubation under continuous illumination (180 μmol m^−2^ s^−1^ photosynthetically active radiation), cells were harvested and immediately frozen in liquid nitrogen for subsequent RNA isolation or FLAG-tagged AGO3 purification. A similar protocol was used for the analysis of phosphate or sulfur deprived cells, following prior specifications^15, 25^. The wild type strain, CC-124, and a transgenic strain, Maa7-IR44s, containing an inverted repeat construct targeting the 3′ UTR of the *MAA7* gene (encoding tryptophan synthase β subunit) and the FLAG-tagged AGO3, have been previously described^14, 17^. Mut-20, deleted for the *TSN1* gene, was obtained in an insertional mutagenesis screen designed to isolate mutants defective in RNAi-mediated translation repression^14^. Likewise, ago3-1, containing a disrupted *AGO3* gene, was isolated in a forward mutagenesis screen in the Gluc(1x) background^28^. For estimating strain survival under nutritional stress, cells were grown in TAP or HS medium to the middle of the logarithmic phase, washed three times in the desired medium, and resuspended to a density of ~1.0 × 10^6^ cells mL^−1^ in nutrient replete TAP or HS medium or in the same medium lacking N, P or S. After incubation in liquid medium, under standard culture conditions, for 3 or 18 d, aliquots of cells were spread on TAP-agar plates (5 replicates per treatment and strain) to assess survival as colony forming units.

### Isolation of AGO3-associated sRNAs, library preparation, and sequencing

FLAG-tagged AGO3 was affinity purified from cell lysates as previously described for a TAP-tagged protein^60^. RNAs associated with AGO3 were purified with TRI reagent (Molecular Research Center) and contaminant DNA was removed by DNase I treatment (Ambion)^17^. Construction of cDNA libraries and Illumina sequencing were then carried out as previously reported^61^. AGO3-associated sRNAs were characterized from cells grown mixotrophically in TAP or photoautotrophically in HS + N or HS − N media (NCBI accession numbers SRR1747077, SRR2959984 and SRR2959993, respectively).

### sRNA mapping and profiling

Sequenced reads were first mapped to the *C. reinhardtii* genome^62^, by using version 3.02 of Novoalign (www.novocraft.com) with the miRNA flag and with a score threshold of 15. Mapped reads were filtered to remove those showing alignments with gaps or mismatches as well as those that mapped to more than five locations in the genome. Reads mapping to the chloroplast or mitochondrial genomes or to functional non-coding RNAs were also removed, as previously described^14^. The expression level in counts per million (CPM) for each mapped sRNA was determined by the formula:

$$\mathrm{CPM}=[({{\rm{10}}}^{{\rm{6}}}{\rm{C}})/N]$$

where *C* is the number of mapped reads corresponding to an individual sRNA sequence in the library and *N* is the total number of mapped reads in the library. We also re-analyzed, in the same manner, the sRNA libraries generated by Chávez Montes *et al*.^25^ (accession number GSM803103) to identify miRNAs related to phosphate or sulfur deprivation.

### Genomic clustering of sRNAs and miRNA identification

Clusters of reads were identified as previously described^14^ and the genomic sequence for each strand of a cluster was folded using version 2.1.5 of RNAfold from the Vienna RNA package^63^. Clusters containing sequence gaps (*i.e*., unsequenced genomic regions) were excluded from further analyses since the secondary structure of these regions cannot be unambiguously predicted. The obtained secondary structures were then parsed to determine if they fold into a hairpin. Clusters remaining after this filtering were manually curated based on the processing accuracy of the 5′ end of the predominant read(s), the frequency of the predominant read(s), and the extent of complementarity between the two arms of the hairpins, according to the criteria for canonical miRNA prediction^14, 29^.

### RNA analyses

Total RNA was isolated with TRI reagent (Molecular Research Center, Inc.)^17, 61^, in accordance with the manufacturer’s instructions, from *C. reinhardtii* cells grown under the different trophic conditions. The same RNA samples were used for northern blotting and for transcriptome analyses (see below). For sRNA northern analyses, total RNA samples were resolved in 15% polyacrylamide/7-M urea gels and electroblotted to Hybond-XL membranes (GE Healthcare)^61^. Blots were hybridized with ^32^P-labeled DNA probes using the High Efficiency Hybridization System at 40 °C for 72 h^17, 61^. Specific miRNAs were detected by hybridization with DNA oligonucleotides labeled at their 5′ termini with [γ-^32^P]ATP and T4 Polynucleotide Kinase (New England Biolabs)^17, 61^. For quantitative RT-PCR analyses, DNase I-treated RNA samples were used as template for first-strand cDNA synthesis, using an oligo(dT)_18_ primer and SuperScript III reverse transcriptase (Life Technologies). Primer pairs for the quantitative PCR amplifications were as follows: for *Cre04.g227600*, LRR-F (5′-ACCCATGCTCTAAGGACTGGA-3′) and LRR-R (5′-GTCGGAGAAGCAGGTGAGTGT-3′); for *Cre06.g249550*, 249550-F (5′-GGGAAAGAGTGGATGATGTGG-3′) and 249550-R (5′-ACATCAACGTTGTGCCTCACT-3′); and for *Cre12.g552950*, 552950-F (5′-AACTGGATAGGCTGAGCAGGA-3′) and 552950-R (5′-TTGTGGGGACAGCTTCTTCTT-3′). The *ACTIN1* transcript^17, 61^ was amplified for normalization purposes. DNA fragments were amplified and quantified with the RT^2^ SYBR Green/Fluorescein qPCR mastermix (Qiagen), using the iCycler Real Time PCR Detection System (Bio-Rad). For semi-quantitative RT-PCR, the number of cycles showing a linear relationship between input cDNA and the final product were determined in preliminary experiments^17^. Aliquots of each RT-PCR were resolved on 1.2% agarose gels and visualized by ethidium bromide staining. The primer sequences were as follows: for *PHO5* (Cre04.g216700), PHO5-5F (5′-TTCCGTTTCCGTTCTCTGAC-3′) and PHO5-3R (5′-CCCTGCATCTTGTTCTCCAG-3′); for *SLT1* (Cre12.g502600), SLT1-5F (5′-ACGGGTTCTTCGAGCGAATTGC-3′) and SLT1-3R (5′-CGACTGCTTACGCAACAATCTTGG-3′); for *CBLP* (Cre06.g278222), CBLP-5F (5′-CTTCTCGCCCATGACCAC-3′) and CBLP-3R (5′-CCCACCAGGTTGTTCTTCAG-3′); and for the *U6 snRNA*, U6-F (5′-TGCTTCGGCACAACTGTTAAA-3′) and U6-R (5′-AAAATTTGGAACCATTTCTCGATT-3′).

### MicroRNA target prediction

Potential miRNA-binding sites in transcripts were determined as previously described^14^, by searching v11 of the Phytozome *C. reinhardtii* transcriptome using version 2.1 of RNAhybrid^64^. For cleavage targets, this search required perfect matching for nucleotides 2–8 (the miRNA seed region) and nucleotides 9–12 (the miRNA catalytic center), and no more than three G:U wobbles and three mismatches or a gap of >1 nt in the remaining sequence. For translational repression targets, the constraints for the catalytic region were relaxed to allow up to three mismatches or wobbles. Additionally, translational-repression targets needed at least one mismatch or wobble in the catalytic region to keep the two sets of predicted targets non-overlapping^14^. Putative functions of the predicted targets were evaluated by using the annotations of Chlamydomonas genes (if available) as well as conserved protein domains. Functional annotations were obtained with the Algal Functional Annotation Tool^65^ and are mostly based on those in Phytozome v11^66^.

### Differential gene expression analyses

Transcriptome sequencing was performed on RNA samples isolated from Maa7-IR44s and Mut-20 grown photoautotrophically in HS + N or HS − N media or mixotrophically in TAP medium (NCBI accession numbers SRX1451698, SRX1451708, and SRR1747017, respectively). Experiments were performed twice, independently, and libraries were sequenced with the Illumina GAIIx analyzer, as previously described^14^. Illumina reads were mapped to the Augustus v5.0 transcript models for *C. reinhardtii* (available from http://genome.jgi-psf.org/Chlre4/Chlre4.download.ftp.html), by using Burrows-Wheeler Aligner (BWA; v0.5.7)^67^ with a seed length of 25 and allowing 2 mismatches. An in-house Perl script was used to ensure that only reads that matched uniquely to a single transcript were counted. Raw gene counts were determined by adding the number of reads aligned to each transcript. RNA-Seq data for sulfur- or phosphate-deprived samples were taken from Gonzalez-Ballester *et al*.^15^ and Schmollinger *et al*.^68^, respectively (accession numbers GSE17970 and GSE56505). Transcript abundance was analyzed as Reads Per Kilobase of transcript per Million mapped reads (RPKM), which normalizes read counts based on both transcript length and total number of reads, using the formula:

$$\mathrm{RPKM}=[({{\rm{10}}}^{{\rm{9}}}{\rm{C}})/({\rm{NL}})]$$

where *C* is the number of reads mapped to each transcript, *N* is the total number of mapped reads in the library, and *L* is the transcript length in nucleotides^69^. To assess changes in gene expression, transcript abundance was compared between Mut-20 and its parental strain Maa7-IR44s, under each trophic condition, or between different nutritional conditions for the same strain. Differences in gene expression were examined as *log*_2_(FC), where FC (Fold Change) refers to the ratio of RPKM values between compared strains or treatments. Statistical analysis of the data was performed using the DESeq package (version 1.18)^70^. Genes with a *q*-value ≤ 0.05 and ≥2-fold change in transcript abundance under at least one of the pairwise comparisons were considered differentially expressed. The Augustus v5.0 transcript IDs were converted to the Phytozome v11 transcript IDs using the name conversion file on the Phytozome website (https://phytozome.jgi.doe.gov/pz/portal.html#!bulk?org=Org_Creinhardtii).

### Arabidopsis miRNAs and target prediction

Predicted miRNAs and their expression information for *A. thaliana* and *A. lyrata* were taken from Ma *et al*.^26^. Expression levels, given in raw read counts, were converted to CPM as described above. Species-specific miRNAs were determined by comparing the miRNA datasets for *A. thaliana* and *A. lyrata* and cross-referencing with in-text results^26^. Targets for the miRNAs were predicted using version 1.6 of Target Finder^31^, searching against the *A. thaliana* and the *A. lyrata* transcriptomes taken from Phytozome v11^66^.

### Comparisons of miRNA expression and number of predicted targets

All statistical analyses were performed in R using standard libraries. The histograms of miRNA expression were generated by binning miRNAs, based on their average CPM levels, at 500 CPM intervals. MicroRNAs that had no identifiable target or only targeted their precursor transcript were excluded from further analyses, since these miRNAs would not have constraints on their expression level. A cutoff of 500 CPM, chosen based on the findings of Mullokandov *et al*.^49^ for functionally effective miRNAs in metazoans, was used to classify lowly expressed (presumably non-functional) and highly expressed (potentially functional) miRNAs. The numbers of targets predicted for highly expressed and lowly expressed miRNAs were compared using Wilcoxon rank sum test, and Cohen’s *d* was used to determine the effect size for the two groups.

### Analysis of conservation of Chlamydomonas miRNAs

The identified Chlamydomonas mature miRNA sequences were compared against both the mature miRNA and the pre-miRNA hairpin sequences in release 21 of miRBase^33^. Both sequence similarity searches were performed using version 2.2.30+ of blastn with an e-value cutoff of 10^71^. To increase the chances of finding conserved miRNAs, the search was performed against the entire database rather than limiting it to the high confidence miRNAs.

## Electronic supplementary material

Supplementary Figures S1 to S6

Supplementary Table S1

Supplementary Table S2

Supplementary Table S3

## References

[1] Ameres SL, Zamore PD (2013). Diversifying microRNA sequence and function. Nat Rev Mol Cell Biol.

[2] Cui J, You C, Chen X (2016). The evolution of microRNAs in plants. Curr Opin Plant Biol.

[3] Bartel DP (2009). MicroRNAs: target recognition and regulatory functions. Cell.

[4] Zhao T (2007). A complex system of small RNAs in the unicellular green alga Chlamydomonas reinhardtii. Genes Dev.

[5] Molnar A, Schwach F, Studholme DJ, Thuenemann EC, Baulcombe DC (2007). miRNAs control gene expression in the single-cell alga Chlamydomonas reinhardtii. Nature.

[6] Shu L, Hu Z (2012). Characterization and differential expression of microRNAs elicited by sulfur deprivation in Chlamydomonas reinhardtii. BMC Genomics.

[7] Wang HL, Chekanova JA (2016). Small RNAs: essential regulators of gene expression and defenses against environmental stresses in plants. Wiley Interdiscip Rev RNA.

[8] Nguyen GN, Rothstein SJ, Spangenberg G, Kant S (2015). Role of microRNAs involved in plant response to nitrogen and phosphorous limiting conditions. Front Plant Sci.

[9] Khraiwesh B, Zhu JK, Zhu J (2012). Role of miRNAs and siRNAs in biotic and abiotic stress responses of plants. Biochim Biophys Acta.

[10] Gao X (2016). MicroRNAs modulate adaption to multiple abiotic stresses in Chlamydomonas reinhardtii. Sci Rep.

[11] Liang G, He H, Yu D (2012). Identification of nitrogen starvation-responsive microRNAs in Arabidopsis thaliana. PLoS One.

[12] Shukla LI, Chinnusamy V, Sunkar R (2008). The role of microRNAs and other endogenous small RNAs in plant stress responses. Biochim Biophys Acta.

[13] Ding YF, Zhu C (2009). The role of microRNAs in copper and cadmium homeostasis. Biochem Biophys Res Commun.

[14] Voshall A, Kim EJ, Ma X, Moriyama EN, Cerutti H (2015). Identification of AGO3-Associated miRNAs and Computational Prediction of Their Targets in the Green Alga Chlamydomonas reinhardtii. Genetics.

[15] Gonzalez-Ballester D (2010). RNA-seq analysis of sulfur-deprived Chlamydomonas cells reveals aspects of acclimation critical for cell survival. Plant Cell.

[16] Yamasaki T (2013). Complementarity to an miRNA seed region is sufficient to induce moderate repression of a target transcript in the unicellular green alga Chlamydomonas reinhardtii. Plant J.

[17] Ma X (2013). Small interfering RNA-mediated translation repression alters ribosome sensitivity to inhibition by cycloheximide in Chlamydomonas reinhardtii. Plant Cell.

[18] Evers M, Huttner M, Dueck A, Meister G, Engelmann JC (2015). miRA: adaptable novel miRNA identification in plants using small RNA sequencing data. BMC Bioinformatics.

[19] Li J, Wu Y, Qi Y (2014). MicroRNAs in a multicellular green alga Volvox carteri. Sci China Life Sci.

[20] Nozawa M, Miura S, Nei M (2012). Origins and evolution of microRNA genes in plant species. Genome Biol Evol.

[21] Loh YH, Yi SV, Streelman JT (2011). Evolution of microRNAs and the diversification of species. Genome Biol Evol.

[22] Barakat A, Wall PK, Diloreto S, Depamphilis CW, Carlson JE (2007). Conservation and divergence of microRNAs in Populus. BMC Genomics.

[23] Felippes FF, Schneeberger K, Dezulian T, Huson DH, Weigel D (2008). Evolution of Arabidopsis thaliana microRNAs from random sequences. RNA.

[24] Fahlgren N (2010). MicroRNA gene evolution in Arabidopsis lyrata and Arabidopsis thaliana. Plant Cell.

[25] Chávez Montes RA (2014). Sample sequencing of vascular plants demonstrates widespread conservation and divergence of microRNAs. Nat Commun.

[26] Ma Z, Coruh C, Axtell MJ (2010). Arabidopsis lyrata small RNAs: transient MIRNA and small interfering RNA loci within the Arabidopsis genus. Plant Cell.

[27] Sharma N, Tripathi A, Sanan-Mishra N (2015). Profiling the expression domains of a rice-specific microRNA under stress. Front Plant Sci.

[28] Yamasaki T, Kim EJ, Cerutti H, Ohama T (2016). Argonaute3 is a key player in miRNA-mediated target cleavage and translational repression in Chlamydomonas. Plant J.

[29] Tarver JE, Donoghue PC, Peterson KJ (2012). Do miRNAs have a deep evolutionary history?. BioEssays.

[30] Valli AA (2016). Most microRNAs in the single-cell alga Chlamydomonas reinhardtii are produced by Dicer-like 3-mediated cleavage of introns and untranslated regions of coding RNAs. Genome Res.

[31] Fahlgren N (2007). High-throughput sequencing of Arabidopsis microRNAs: evidence for frequent birth and death of MIRNA genes. PLoS One.

[32] Cuperus JT, Fahlgren N, Carrington JC (2011). Evolution and functional diversification of MIRNA genes. Plant Cell.

[33] Kozomara A, Griffiths-Jones S (2014). miRBase: annotating high confidence microRNAs using deep sequencing data. Nucleic Acids Res.

[34] Dueck A (2016). Gene silencing pathways found in the green alga Volvox carteri reveal insights into evolution and origins of small RNA systems in plants. BMC Genomics.

[35] Fuchs RT, Sun Z, Zhuang F, Robb GB (2015). Bias in ligation-based small RNA sequencing library construction is determined by adaptor and RNA structure. PLoS One.

[36] Jackson TJ, Spriggs RV, Burgoyne NJ, Jones C, Willis AE (2014). Evaluating bias-reducing protocols for RNA sequencing library preparation. BMC Genomics.

[37] Alon S (2011). Barcoding bias in high-throughput multiplex sequencing of miRNA. Genome Res.

[38] Hafner M (2011). RNA-ligase-dependent biases in miRNA representation in deep-sequenced small RNA cDNA libraries. RNA.

[39] Molnar A (2009). Highly specific gene silencing by artificial microRNAs in the unicellular alga Chlamydomonas reinhardtii. Plant J.

[40] Zhao T, Wang W, Bai X, Qi Y (2009). Gene silencing by artificial microRNAs in Chlamydomonas. Plant J.

[41] Hausser J, Zavolan M (2014). Identification and consequences of miRNA-target interactions-beyond repression of gene expression. Nat Rev Genet.

[42] Liang H, Zhang J, Zen K, Zhang CY, Chen X (2013). Nuclear microRNAs and their unconventional role in regulating non-coding RNAs. Protein Cell.

[43] Carroll AP, Tran N, Tooney PA, Cairns MJ (2012). Alternative mRNA fates identified in microRNA-associated transcriptome analysis. BMC Genomics.

[44] Chung, B. Y. W., Deery, M. J., Groen, A. J., Howard, J. & Baulcombe, D. C. mRNA turnover through CDS-targeting is the primary role of miRNA in the green alga Chlamydomonas. *bioRxiv* doi:10.1101/088807 (2016). 10.1038/s41477-017-0024-6 PMC5662147 28970560

[45] Herron MD, Hackett JD, Aylward FO, Michod RE (2009). Triassic origin and early radiation of multicellular volvocine algae. Proc Natl Acad Sci USA.

[46] Hertel J, Stadler PF (2015). The Expansion of Animal MicroRNA Families Revisited. Life (Basel).

[47] Axtell MJ, Westholm JO, Lai EC (2011). Vive la différence: biogenesis and evolution of microRNAs in plants and animals. Genome Biol.

[48] Guo W (2016). High-throughput sequencing and degradome analysis reveal neutral evolution of Cercis gigantea microRNAs and their targets. Planta.

[49] Mullokandov G (2012). High-throughput assessment of microRNA activity and function using microRNA sensor and decoy libraries. Nature Meth.

[50] Bosson AD, Zamudio JR, Sharp PA (2014). Endogenous miRNA and target concentrations determine susceptibility to potential ceRNA competition. Mol Cell.

[51] Denzler R (2016). Impact of MicroRNA Levels, Target-Site Complementarity, and Cooperativity on Competing Endogenous RNA-Regulated Gene Expression. Mol Cell.

[52] Nozawa M (2016). Evolutionary Transitions of MicroRNA-Target Pairs. Genome Biol Evol.

[53] Casas-Mollano JA (2008). Diversification of the core RNA interference machinery in Chlamydomonas reinhardtii and the role of DCL1 in transposon silencing. Genetics.

[54] Burroughs AM, Ando Y, Aravind L (2014). New perspectives on the diversification of the RNA interference system: insights from comparative genomics and small RNA sequencing. Wiley Interdisciplinary Reviews RNA.

[55] Tarver JE (2015). microRNAs and the evolution of complex multicellularity: identification of a large, diverse complement of microRNAs in the brown alga Ectocarpus. Nucleic Acids Res.

[56] Mattick JS (2007). A new paradigm for developmental biology. J Exp Biol.

[57] Peterson KJ, Dietrich MR, McPeek MA (2009). MicroRNAs and metazoan macroevolution: insights into canalization, complexity, and the Cambrian explosion. Bioessays.

[58] Avesson L, Reimegård J, Wagner EG, Söderbom F (2012). MicroRNAs in Amoebozoa: deep sequencing of the small RNA population in the social amoeba Dictyostelium discoideum reveals developmentally regulated microRNAs. RNA.

[59] Sueoka N (1960). Mitotic Replication of Deoxyribonucleic Acid in Chlamydomonas Reinhardi. Proc Natl Acad Sci USA.

[60] van Dijk K (2005). Monomethyl histone H3 lysine 4 as an epigenetic mark for silenced euchromatin in Chlamydomonas. Plant Cell.

[61] Ibrahim F (2010). Uridylation of mature miRNAs and siRNAs by the MUT68 nucleotidyltransferase promotes their degradation in Chlamydomonas. Proc Natl Acad Sci USA.

[62] Merchant SS (2007). The Chlamydomonas genome reveals the evolution of key animal and plant functions. Science.

[63] Lorenz R (2011). Vienna RNA Package 2.0. Algorithms Mol Biol.

[64] Kruger, J. & Rehmsmeier, M. RNAhybrid: microRNA target prediction easy, fast and flexible. *Nucleic Acids Res* **34** (Web Server issue), W451–454 (2006). 10.1093/nar/gkl243 PMC1538877 16845047

[65] Lopez D, Casero D, Cokus SJ, Merchant SS, Pellegrini M (2011). Algal Functional Annotation Tool: a web-based analysis suite to functionally interpret large gene lists using integrated annotation and expression data. BMC Bioinformatics.

[66] Goodstein DM (2012). Phytozome: a comparative platform for green plant genomics. Nucleic Acids Res.

[67] Li H, Durbin R (2009). Fast and accurate short read alignment with Burrows-Wheeler transform. Bioinformatics.

[68] Schmollinger S (2014). Nitrogen-Sparing Mechanisms in Chlamydomonas Affect the Transcriptome, the Proteome, and Photosynthetic Metabolism. Plant Cell.

[69] Mortazavi A, Williams BA, McCue K, Schaeffer L, Wold B (2008). Mapping and quantifying mammalian transcriptomes by RNA-Seq. Nature Meth.

[70] Anders S, Huber W (2010). Differential expression analysis for sequence count data. Genome Biol.

[71] Altschul SF, Gish W, Miller W, Myers EW, Lipman DJ (1990). Basic local alignment search tool. J Mol Biol.

