# Supplementary material for: miRNAs in the alga Chlamydomonas reinhardtii are not phylogenetically conserved and play a limited role in responses to nutrient deprivation
Source: Sci Rep. 2017 Jul 14;7:5462. doi: 10.1038/s41598-017-05561-0 (PMC5511227; doi:10.1038/s41598-017-05561-0)
Supplement: Supplementary file 1 — Supplementary Figures S1 to S6 [file 41598_2017_5561_MOESM1_ESM.pdf]

miRNAs in the alga *Chlamydomonas reinhardtii* are not phylogenetically conserved and play a limited role in responses to nutrient deprivation

Adam Voshall[1,2,*], Eun-Jeong Kim[1,*], Xinrong Ma[1,2], Tomohito Yamasaki[3], Etsuko N. Moriyama[1,2] & Heriberto Cerutti[1,2]

[1]School of Biological Sciences, University of Nebraska-Lincoln, Lincoln, Nebraska, USA

[2]Center for Plant Science Innovation, University of Nebraska-Lincoln, Lincoln, Nebraska, USA

[3]Division of Environmental Photobiology, National Institute for Basic Biology, Okazaki, Aichi Prefecture, Japan

*These authors contributed equally to this work.

Supplemental Figure 1: miRNA clusters for all conditions examined

miRNAs identified in TAP only :

miRNA: miR_t8 chromosome_4 Start: 3694773 End: 3694881 Condition: TAP

```
---------------------------------------------TATCACCCGTGTTACAGG--      0.097      0.000      0.000
--------------------------TTAGTCGTCACAAGGCGTG--------------------      0.146      0.076      0.076
------------------------ACTTAGTCGTCACAAGGCG----------------------      0.146      0.000      0.000
-----------------------AACTTAGTCGTCACAAGGCG----------------------      1.117      0.530      0.075
----------------------TAACTTAGTCGTCACAAGGCGTG--------------------      0.534      0.152      0.152
----------------------TAACTTAGTCGTCACAAGGC-----------------------      0.049      0.341      0.075
----------------------TAACTTAGTCGTCACAAGGCGT---------------------      0.049      0.038      0.038
----------------------TAACTTAGTCGTCACAAGGCG----------------------     77.126     51.108      2.259 miR_t8
-------------------TGGTAACTTAGTCGTCACAAGG------------------------      0.049      0.000      0.000
-------------------TGGTAACTTAGTCGTCACAAG-------------------------      0.340      0.000      0.000
----TGTAACGAGGGAAAATGGTAA----------------------------------------      0.097      0.000      0.000
----TGTAACGAGGGAAAATGGTA-----------------------------------------      1.651      0.076      0.339

GTCCTGTAACGAGGGAAAATG              A                        A  T
                     GTAACTTAGTCGTC CAAGGCGTGTATCACCCGTGTTAC GG C
                     CATTGAATCAGTAG GTTCCGCACATAGTGGGTACAATG CC C
---------------------              G                        -  C

-------------------------GAATCAGTAGGGTTCCGCACAT------------------      0.049      0.000      0.000
```

miRNA: miR_t10,miR_t90 chromosome_16 Start: 184972 End: 185187 Condition: TAP

```
---------------------------------------------------------------------------------------------------------------TATACGGTATCCAGCAAGCAACATC-------------------------      0.049      0.000      0.000
---------------------------------------------------------------------------------------------------------------TATACGGTATCCAGCAAGC-------------------------------      0.049      0.000      0.000
----------------------------------------------------------------------------------------------------------------ATACGGTATCCAGCAA---------------------------------      0.049      0.000      0.000
-----------------------------------------------------------------------------------------------------------------TACGGTATCCAGCAAGCAACATCC------------------------      0.049      0.000      0.000
-----------------------------------------------------------------------------------------------------------------TACGGTATCCAGCAAGCAAC----------------------------      0.049      0.038      0.038
----------------------------------------------------------------------------------------------------------------------TATCCAGCAAGCAACATCCACAT--------------------      0.049      0.075      0.075
----------------------------------------------------------------------------------------------------------------------TATCCAGCAAGCAACATCCACA---------------------      4.420      1.478      2.937
----------------------------------------------------------------------------------------------------------------------TATCCAGCAAGCAACATCCAC----------------------      0.291      0.189      0.377
-----------------------------------------------------------------------------------------------------------------------ATCCAGCAAGCAACATCCACA---------------------      0.097      0.000      0.000 miR_t90*
------------------------------------------------------------------------------------------------------------------------TCCAGCAAGCAACATCCACAT--------------------      1.457      0.530      0.377
------------------------------------------------------------------------------------------------------------------------TCCAGCAAGCAACATCCAC----------------------      0.194      0.000      0.000
------------------------------------------------------------------------------------------------------------------------TCCAGCAAGCAACATCCACATA-------------------      0.777      0.341      0.226
------------------------------------------------------------------------------------------------------------------------TCCAGCAAGCAACATCCACA---------------------      5.003      0.985      1.242
--------------------------------------------------------------------------------------------------------------------------CAGCAAGCAACATCCACA---------------------      0.049      0.000      0.000
--------------------------------------------------------------------------------------------------------------------------CAGCAAGCAACATCCACATA-------------------      0.049      0.000      0.000
--------------------------------------------------------------------------------------------------------------------------------------TCCACATAAGCCGGGGTTGG-------      1.020      0.114      0.188
--------------------------------------------------------------------------------------------------------------------------------------------TAAGCCGGGGTTGGTAGC---     68.287     77.629     71.273
--------------------------------------------------------------------------------------------------------------------------------------------TAAGCCGGGGTTGGTAG----     14.328      4.167      1.017
--------------------------------------------------------------------------------------------------------------------------------------------TAAGCCGGGGTTGGT------      0.049      0.000      0.000
--------------------------------------------------------------------------------------------------------------------------------------------TAAGCCGGGGTTGG-------      0.049      0.000      0.000
--------------------------------------------------------------------------------------------------------------------------------------------TAAGCCGGGGTTGGTAGCA--      9.179      4.281      7.568
--------------------------------------------------------------------------------------------------------------------------------------------TAAGCCGGGGTTGGTA-----      0.146      0.000      0.000
--------------------------------------------------------------------------------------------------------------------------------------------TAAGCCGGGGTTGGTAGCAA-    101.362     22.353     42.432
---------------------------------------------------------------------------------------------------------------------------------------------AAGCCGGGGTTGGTAGCAA-      0.243      0.114      0.038
----------------------------------------------------------------------------------------------------------------------------------------------AGCCGGGGTTGGTAGCAA-      0.049      0.000      0.000
--------------------------------------------------------------------------------------------------------------------------------------------TAAGCCGGGGTTGGTAGCAAC   1534.900    181.891    339.422 miR_t10

-----------------------------------------------------------------------------------------------------------GGGCTA                                            CAA
                                                                                                                 TACGGTATCCAGCAAGCAACATCCACATAAGCCGGGGTTGGTAG   C
                                                                                                                 GTGCCATAGGTCGTTCGTTGTAGGTGTATTCGGCCCCAACCATC   T
ACACGAAAGTCACTTACCGGCGTGTCTTACCGTCGTTCAGGTCGGCGAGTGTTTTTTTGGTAGGTGGCGGGGTTGTGCCCGGGTGGCCGGTTCCGGCGGGGTTGGTTGGTGTA                                            TTT

------------------------------------------------------------------------------------------------------------------------------------------GTATTCGGCCCCAACCATCT---      0.194      0.038      0.038
------------------------------------------------------------------------------------------------------------------------------------------GTATTCGGCCCCAACCATC----      0.049      0.000      0.000
--------------------------------------------------------------------------------------------------------------------------GTCGTTCGTTGTAGGTGTATT------------------      0.049      0.038      0.038
------------------------------------------------------------------------------------------------------------------------AGGTCGTTCGTTGTAGGTGTAT-------------------      0.389      0.113      0.113
-----------------------------------------------------------------------------------------------------------------------TAGGTCGTTCGTTGTAGGTGTAT-------------------      0.097      0.000      0.000
-------------------------------------------------------------------------------------------------------------------------GGTCGTTCGTTGTAGGTGTAT-------------------      6.751      1.364      1.092
---------------------------------------------------------------------------------------------------------------------CATAGGTCGTTCGTTGTAGGTGT---------------------      1.166      3.599      0.075
-----------------------------------------------------------------------------------------------------------------------TAGGTCGTTCGTTGTAGGTGT---------------------   3653.784    285.320    373.007
-------------------------------------------------------------------------------------------------------------------GCCATAGGTCGTTCGTTGTAGGTGT---------------------      0.146      0.114      0.114
------------------------------------------------------------------------------------------------------------------------AGGTCGTTCGTTGTAGGTGT---------------------    415.355     51.070     72.478
--------------------------------------------------------------------------------------------------------------------CCATAGGTCGTTCGTTGTAGGTGT---------------------      0.486      1.288      1.288
----------------------------------------------------------------------------------------------------------------------------CGTTCGTTGTAGGTGT---------------------      0.243      0.000      0.000
----------------------------------------------------------------------------------------------------------------------ATAGGTCGTTCGTTGTAGGTGT---------------------    302.385     72.817      4.104
---------------------------------------------------------------------------------------------------------------------------TCGTTCGTTGTAGGTGT---------------------      0.389      0.076      0.113
-------------------------------------------------------------------------------------------------------------------------GGTCGTTCGTTGTAGGTGT---------------------     13.890      5.683      7.831
---------------------------------------------------------------------------------------------------------------TAGTGCCATAGGTCGTTCGTTGTAGGTGT---------------------      0.097      0.038      0.038
--------------------------------------------------------------------------------------------------------------------------GTCGTTCGTTGTAGGTGT---------------------      0.971      0.227      0.527
-----------------------------------------------------------------------------------------------------------------------TAGGTCGTTCGTTGTAGGTG----------------------     22.536      1.288      1.581
---------------------------------------------------------------------------------------------------------------------CATAGGTCGTTCGTTGTAGGTG----------------------      0.049      0.000      0.000
----------------------------------------------------------------------------------------------------------------------ATAGGTCGTTCGTTGTAGGTG----------------------      1.748      0.152      0.152
------------------------------------------------------------------------------------------------------------------------AGGTCGTTCGTTGTAGGTG----------------------      3.594      0.152      0.678
------------------------------------------------------------------------------------------------------------------TGCCATAGGTCGTTCGTTGTAGGT-----------------------      0.486      0.568      0.188
----------------------------------------------------------------------------------------------------------------------ATAGGTCGTTCGTTGTAGGT-----------------------   4629.810    846.944   1017.513
--------------------------------------------------------------------------------------------------------------------CCATAGGTCGTTCGTTGTAGGT-----------------------   1537.231    334.913    189.685
---------------------------------------------------------------------------------------------------------------------CATAGGTCGTTCGTTGTAGGT-----------------------   8917.991   7059.466  13144.240 miR_t90
-------------------------------------------------------------------------------------------------------------------------GGTCGTTCGTTGTAGGT-----------------------      6.362      5.153      4.330
----------------------------------------------------------------------------------------------------------------------------CGTTCGTTGTAGGT-----------------------      0.049      0.000      0.000
------------------------------------------------------------------------------------------------------------GTGTAGTGCCATAGGTCGTTCGTTGTAGGT-----------------------      0.049      0.682      0.075
---------------------------------------------------------------------------------------------------------------------------TCGTTCGTTGTAGGT-----------------------      0.049      0.000      0.000
-----------------------------------------------------------------------------------------------------------------------TAGGTCGTTCGTTGTAGGT-----------------------     64.304     42.660     57.756
---------------------------------------------------------------------------------------------------------------TAGTGCCATAGGTCGTTCGTTGTAGGT-----------------------      0.097      1.781      1.781
-------------------------------------------------------------------------------------------------------------------GCCATAGGTCGTTCGTTGTAGGT-----------------------      2.380      1.061      0.527
-----------------------------------------------------------------------------------------------------------------GTGCCATAGGTCGTTCGTTGTAGGT-----------------------      0.291      0.720      0.075
--------------------------------------------------------------------------------------------------------------------------GTCGTTCGTTGTAGGT-----------------------      0.389      0.000      0.000
----------------------------------------------------------------------------------------------------------------AGTGCCATAGGTCGTTCGTTGTAGGT-----------------------      0.680      4.395      0.151
------------------------------------------------------------------------------------------------------------------------AGGTCGTTCGTTGTAGGT-----------------------      5.245      5.001      3.389
--------------------------------------------------------------------------------------------------------------GTAGTGCCATAGGTCGTTCGTTGTAGGT-----------------------      0.146      1.326      1.326
----------------------------------------------------------------------------------------------------------------------ATAGGTCGTTCGTTGTAGG------------------------     30.501      4.357      8.283
-------------------------------------------------------------------------------------------------------------------------GGTCGTTCGTTGTAGG------------------------      0.097      0.000      0.000
-----------------------------------------------------------------------------------------------------------------------TAGGTCGTTCGTTGTAGG------------------------      0.486      0.568      0.602
------------------------------------------------------------------------------------------------------------------------AGGTCGTTCGTTGTAGG------------------------      0.049      0.076      0.038
---------------------------------------------------------------------------------------------------------------------CATAGGTCGTTCGTTGTAGG------------------------    102.770     58.648    186.560
--------------------------------------------------------------------------------------------------------------------CCATAGGTCGTTCGTTGTAGG------------------------     11.705      2.917      1.995
-------------------------------------------------------------------------------------------------------------------GCCATAGGTCGTTCGTTGT---------------------------      0.097      0.000      0.000
----------------------------------------------------------------------------------------------------------------AGTGCCATAGGTCGTTCGTTGT---------------------------      0.097      0.000      0.000
----------------------------------------------------------------------------------------------------------------------ATAGGTCGTTCGTTGT---------------------------      0.049      0.000      0.000
---------------------------------------------------------------------------------------------------------------TAGTGCCATAGGTCGTTCGTT-----------------------------      0.049      0.038      0.038
--------------------------------------------------------------------------------------------------------------GTAGTGCCATAGGTCGTTCGTT-----------------------------      0.243      0.000      0.000
----------------------------------------------------------------------------------------------------------------AGTGCCATAGGTCGTTCGTT-----------------------------      0.097      0.189      0.189
----------------------------------------------------------------------------------------------------------------AGTGCCATAGGTCGTTCGT------------------------------      0.049      0.000      0.000
----GAAAGTCACTTACCGGCGTGTCT--------------------------------------------------------------------------------------------------------------------------------------      0.097      0.000      0.000
```

miRNA: miR_t36 chromosome_12 Start: 3183047 End: 3183300 Condition: TAP

```
----------------------------------------------------------------------TAACGGACCTGGAGGACAGGCGGCG-----------------------------------------------------------      0.049      0.000      0.000
----------------------------------------------------------------------TAACGGACCTGGAGGACAGGC---------------------------------------------------------------      0.049      1.212      0.527
----------------------------------------------------------------------TAACGGACCTGGAGGACAGG----------------------------------------------------------------      1.894      3.220      1.883
--------------------------------------------------------------------AATAACGGACCTGGAGGACA------------------------------------------------------------------      0.097      0.038      0.038
--------------------------------------------------------------------AATAACGGACCTGGAGGAC-------------------------------------------------------------------      0.146      0.000      0.000
-------------------------------------------------------------------CAATAACGGACCTGGAGGACA------------------------------------------------------------------      0.194      0.000      0.000
-------------------------------------------------------------------CAATAACGGACCTGGAGGAC-------------------------------------------------------------------      0.291      0.000      0.000
------------------------------------------------------------------TCAATAACGGACCTGGAGGA--------------------------------------------------------------------      0.340      0.114      0.151
------------------------------------------------------------------TCAATAACGGACCTGGAG----------------------------------------------------------------------      0.049      0.000      0.000
------------------------------------------------------------------TCAATAACGGACCTGGAGGACA------------------------------------------------------------------     18.310     13.904      1.920
------------------------------------------------------------------TCAATAACGGACCTGG------------------------------------------------------------------------      0.049      0.000      0.000
------------------------------------------------------------------TCAATAACGGACCTGGA-----------------------------------------------------------------------      0.097      0.000      0.000
------------------------------------------------------------------TCAATAACGGACCTGGAGGAC-------------------------------------------------------------------     57.359     22.656     12.538 miR_t36
-----------------------------------------------------------------TTCAATAACGGACCTGGAGG---------------------------------------------------------------------      0.389      1.970      0.452
-----------------------------------------------------------------TTCAATAACGGACCTGGAGGA--------------------------------------------------------------------      1.748      0.227      0.075
-----------------------------------------------------------------TTCAATAACGGACCTGGAGGAC-------------------------------------------------------------------      0.826      0.493      0.301
-----------------------------------------------------------------TTCAATAACGGACCTGGAGGACA------------------------------------------------------------------      0.097      0.152      0.152
--------------------------------------------------------------AACTTCAATAACGGACCTGG------------------------------------------------------------------------      0.049      0.038      0.038
------------------------------------------------------------TCAACTTCAATAACGGACCTGG------------------------------------------------------------------------      1.263      0.265      0.452
------------------------------------------------------------TCAACTTCAATAACGGACC---------------------------------------------------------------------------      0.437      0.758      0.527
------------------------------------------------------------TCAACTTCAATAACGGACCTG-------------------------------------------------------------------------      0.146      0.038      0.151
--------------------------------------------------------TGCATCAACTTCAATAACGGA-----------------------------------------------------------------------------      0.631      0.113      0.113
--------------------------------------------------------TGCATCAACTTCAATAACGGACC---------------------------------------------------------------------------      0.291      0.265      0.265
--------------------------------------------------------TGCATCAACTTCAATAACGG------------------------------------------------------------------------------      0.049      0.189      0.189
-------------------------------------------------------TTGCATCAACTTCAATAACGG------------------------------------------------------------------------------      0.049      0.152      0.152
------------------------------------------------------TTTGCATCAACTTCAATAACGG------------------------------------------------------------------------------      0.146      0.000      0.000
-----------------------------------------------------TTTTGCATCAACTTCAATAACGG------------------------------------------------------------------------------      0.049      0.000      0.000
---------------------TCCAAGGCCGGAAGCGGTGCC----------------------------------------------------------------------------------------------------------------      0.049      0.000      0.000

-----------------                                                                              ATGACGGAGATGCCATAACTCATCAGGTCCTCGTCCCCA     CA  CCACA-  CT
                 GGCGTCCAAGGCCGGAAGCGGTGCCAGCATACGTGGTTTTGCATCAACTTCAATAACGGACCTGGAGGACAGGCGGCG                                       GCTGC  CT       TC  A
                 CCGCAGGTTCCGGCCTTCGCCACGGTCGTATGCACCAAAACGTAGTTGAAGTTATTGCCTGGACCTCCTGTCCGCCGC                                       CGACG  GA       AG  /
TTCTCCCGGGGCCGTCG                                                                              GAA------------------------------------     AC  CCGCAC  AA

----CCCGGGGCCGTCGCCGCAGGT---------------------------------------------------------------------------------------------------------------------------------      0.243      0.000      0.000
------CGGGGCCGTCGCCGCAGGT---------------------------------------------------------------------------------------------------------------------------------      0.049      0.000      0.000
-----CCGGGGCCGTCGCCGCAGGTT--------------------------------------------------------------------------------------------------------------------------------      0.243      0.076      0.076
------CGGGGCCGTCGCCGCAGGTT--------------------------------------------------------------------------------------------------------------------------------      0.097      0.227      0.301
----------------------GGTTCCGGCCTTCGCCACGG----------------------------------------------------------------------------------------------------------------      0.049      0.076      0.076
----------------------GGTTCCGGCCTTCGCCACGGT---------------------------------------------------------------------------------------------------------------      2.331      0.644      0.414
---------------------------CGGCCTTCGCCACGGTCGTAT----------------------------------------------------------------------------------------------------------      0.049      0.076      0.038
----------------------------GGCCTTCGCCACGGTCGTAT----------------------------------------------------------------------------------------------------------      0.097      0.076      0.076
---------------------------------------CGGTCGTATGCACCAAAACGT----------------------------------------------------------------------------------------------      0.194      0.000      0.000
------------------------------------------TCGTATGCACCAAAACGTAG--------------------------------------------------------------------------------------------      0.049      0.000      0.000
----------------------------------------GGTCGTATGCACCAAAACGTAGT-------------------------------------------------------------------------------------------      0.049      0.000      0.000
-------------------------------------------CGTATGCACCAAAACGTAGTT------------------------------------------------------------------------------------------      0.049      0.682      0.264
---------------------------------------------------CCAAAACGTAGTTGAAGTTAT----------------------------------------------------------------------------------      0.049      0.000      0.000
----------------------------------------------------CAAAACGTAGTTGAAGTTATT---------------------------------------------------------------------------------      0.049      0.038      0.038
--------------------------------------------------------ACGTAGTTGAAGTTATTGCCT-----------------------------------------------------------------------------      0.097      0.114      0.114
---------------------------------------------------------CGTAGTTGAAGTTATTGCCT-----------------------------------------------------------------------------      0.243      0.038      0.038
---------------------------------------------------------------TGAAGTTATTGCCTGGACCT-----------------------------------------------------------------------      0.097      0.152      0.152
-------------------------------------------------------------GTTGAAGTTATTGCCTGGACCT-----------------------------------------------------------------------      0.340      0.189      0.226
----------------------------------------------------------------GAAGTTATTGCCTGGACCTC----------------------------------------------------------------------      0.194      0.000      0.000
----------------------------------------------------------------GAAGTTATTGCCTGGACCTCCT--------------------------------------------------------------------      6.654      2.879      0.226
```

```
------------------------------------------------------------------AGTTATTGCCTGGACCTCCT--------------------------------------------------------------------      0.049      0.000      0.000
---------------------------------------------------------------TGAAGTTATTGCCTGGACCTCCT--------------------------------------------------------------------      0.049      0.114      0.114
------------------------------------------------------------------AGTTATTGCCTGGACCTCCTGT------------------------------------------------------------------      0.049      0.000      0.000
-------------------------------------------------------------------GTTATTGCCTGGACCTCCTGT------------------------------------------------------------------      0.097      0.038      0.038

miRNA: miR_t40,miR_t42 chromosome_14 Start: 1950047 End: 1950359 Condition: TAP
------------------------------------------------------------------------------------------------------------------------------------------------AGGCGCGGAGCTCATCAAGC----------      0.049      0.076      0.075
-----------------------------------------------------------------------------------------------------------------------------------------------TAGGCGCGGAGCTCATCAAGC----------      1.554      7.426      7.530
-----------------------------------------------------------------------------------------------------------------------------------------------TAGGCGCGGAGCTCATCAAG-----------      0.146      0.188      0.188
-----------------------------------------------------------------------------------------------------------------------------------------------TAGGCGCGGAGCTCATCAAGCG---------      0.049      0.000      0.000
-----------------------------------------------------------------------------------------------------------------TGCAGGGGCATCTCGATGTCC----------------------------------------      0.049      0.000      0.000
--------------------------------------------------------------------------------------------------------------TACTGCAGGGGCATCTC-----------------------------------------------      0.146      0.152      0.414
--------------------------------------------------------------------------------------------------------------TACTGCAGGGGCATCT------------------------------------------------      0.049      0.000      0.000
--------------------------------------------------------------------------------------------------TCGCCGCGCAGGTACTGCAGG-------------------------------------------------------      0.874      0.114      0.114
-----------------------------------------------------------------------------------------------TTCTCGCCGCGCAGGTACTGC----------------------------------------------------------      0.534      0.227      0.339
--------------------------------------------------------------------------------------------ACGTTCTCGCCGCGCAGG----------------------------------------------------------------      0.049      0.000      0.000
-------------------------------------------------------------------------------------------CACGTTCTCGCCGCGCAGG----------------------------------------------------------------      9.811      1.364      1.920
-----------------------------------------------------------------------------------------TCCACGTTCTCGCCGCGCAGG----------------------------------------------------------------    339.443     84.221     61.672 miR_t40
-----------------------------------------------------------------------------------------TCCACGTTCTCGCCGCGCAGGT---------------------------------------------------------------      0.729      0.076      0.113
-----------------------------------------------------------------------------------------TCCACGTTCTCGCCGCGCAGGTACT------------------------------------------------------------      0.049      0.000      0.000
-----------------------------------------------------------------------------------------TCCACGTTCTCGCCGC---------------------------------------------------------------------      0.097      0.000      0.000
-----------------------------------------------------------------------------------------TCCACGTTCTCGCCGCG--------------------------------------------------------------------      0.049      0.000      0.000
-----------------------------------------------------------------------------------------TCCACGTTCTCGCCGCGCAG-----------------------------------------------------------------      0.971      0.720      0.941
----------------------------------------------------------------------------------------CTCCACGTTCTCGCCGCGCAGG----------------------------------------------------------------      0.243      0.152      0.152
--------------------------------------------------------------------------------------TTCTCCACGTTCTCGCCGCGC-------------------------------------------------------------------      0.243      0.038      0.038
--------------------------------------------------------------------------------------TTCTCCACGTTCTCGCCGCGCA------------------------------------------------------------------      0.146      0.038      0.038
--------------------------------------------------------------------------TTCTCCGTCACCTTCTCCACG-------------------------------------------------------------------------------      0.340      0.227      0.452
------------------------------------------------------------------------CATTCTCCGTCACCTTCTCC----------------------------------------------------------------------------------      0.049      0.000      0.000
------------------------------------------------------------------------CATTCTCCGTCACCTTCT------------------------------------------------------------------------------------      0.049      0.000      0.000
-----------------------------------------------------------------------ACATTCTCCGTCACCTTCTCC----------------------------------------------------------------------------------      0.146      0.038      0.226
----------------------------------------------------------------------TACATTCTCCGTCACCTTCT------------------------------------------------------------------------------------      2.477      1.364      0.904
----------------------------------------------------------------------TACATTCTCCGTCACCTTC-------------------------------------------------------------------------------------      2.574      5.380      3.238
----------------------------------------------------------------------TACATTCTCCGTCACCTTCTCC----------------------------------------------------------------------------------      4.808      2.538      3.276
----------------------------------------------------------------------TACATTCTCCGTCACCTTCTC-----------------------------------------------------------------------------------      0.291      0.189      0.075
----------------------------------------------------------------------TACATTCTCCGTCACCTTCTCCA---------------------------------------------------------------------------------      0.437      0.114      0.114
----------------------------------------------------------------------TACATTCTCCGTCACC----------------------------------------------------------------------------------------      0.049      0.000      0.000
---------------------------------------------------------------------TTACATTCTCCGTCACCTTC-------------------------------------------------------------------------------------      0.874      0.265      1.017
---------------------------------------------------------------------TTACATTCTCCGTCACCTTCTCC----------------------------------------------------------------------------------      0.049      0.038      0.038
---------------------------------------------------------------------TTACATTCTCCGTCACC----------------------------------------------------------------------------------------      0.049      0.038      0.038
---------------------------------------------------------------------TTACATTCTCCGTCACCTTCTC-----------------------------------------------------------------------------------      0.146      0.113      0.113
---------------------------------------------------------------------TTACATTCTCCGTCACCTTCT------------------------------------------------------------------------------------      0.146      0.301      0.301
----------------------------------------------------------------TTACCTTACATTCTCCGTCACCTTCT------------------------------------------------------------------------------------      0.049      0.000      0.000
----------------------------------------------------------------TTACCTTACATTCTCCGTCACCTTC-------------------------------------------------------------------------------------      0.049      0.152      0.152
---------------------------------------------------------TACCCACTTACCTTACATTCTCC----------------------------------------------------------------------------------------------      0.971      1.174      0.113
---------------------------------------------------------TACCCACTTACCTTACATTCTC-----------------------------------------------------------------------------------------------      0.243      0.113      0.113
-----------------------------------CGAAGTTATCAGGAACGGGAAG---------------------------------------------------------------------------------------------------------------------      0.049      0.000      0.000
----TTTTTGTAACGACGGGAAAAG-----------------------------------------------------------------------------------------------------------------------------------------------------      0.049      0.038      0.038
----TTTTTGTAACGACGGGAAAAGG----------------------------------------------------------------------------------------------------------------------------------------------------      0.049      0.000      0.000
----TTTTTGTAACGACGGGAAAA------------------------------------------------------------------------------------------------------------------------------------------------------      0.049      0.000      0.000

GGGGTTTTTGTAACGACGGGAAAAGGCCCGCCCTTC      AT                  A  TACC                       A                                                                            C TC
                                    GAAGTT  CAGGAACGGGAAGTACCC CT    TTACATTCTCCGTCACCTTCTCC CGTTCTCGCCGCGCAGGTACTGCAGGGGCATCTCGATGTCCATCGCCTCGTAGGCGCGGAGCTCATCAAGCGCCTG G  T
                                    CTTCAA  GTCCTTGCCCTTCATGGG GG    AATGTAAGAGGCGGTGGAAGAGG GCAAGAGCGGCGCGTCCATGACGTCCCCGTAGAGCTACAGGTAGCGGAGCATCCGCGCCTCGAGTAGTTCGCGGAC C  T
----------------------------------AT      CG                  C  CGC-                       C                                                                            - TT

--------------------------------------TCAACGGTCCTTGCCCTTCAT-------------------------------------------------------------------------------------------------------------------      0.049      0.000      0.000
-----------------------------------------------------------------CGC-AATGTAAGAGGCGGTGGA---------------------------------------------------------------------------------------      0.049      0.682      0.866
---------------------------------------------------------------------AATGTAAGAGGCGGTGGAAGA-------------------------------------------------------------------------------------      0.049      1.894      1.995
------------------------------------------------------------------------------------GGAAGAGGCGCAAGAGCGGCGC--------------------------------------------------------------------      0.049      0.265      0.113
------------------------------------------------------------------------------------GGAAGAGGCGCAAGAGCGGCGCGT------------------------------------------------------------------      0.097      0.265      0.188
-----------------------------------------------------------------------------------TGGAAGAGGCGCAAGAGCGGCGCGT------------------------------------------------------------------      0.097      0.038      0.038
------------------------------------------------------------------------------------------------AGAGCGGCGCGTCCATGACGT---------------------------------------------------------      0.146      0.076      0.038
-------------------------------------------------------------------------------------------------GAGCGGCGCGTCCATGACGT---------------------------------------------------------      0.049      0.076      0.038
-----------------------------------------------------------------------------------------------------GGCGCGTCCATGACGTCCCCGT---------------------------------------------------      0.146      0.000      0.000
------------------------------------------------------------------------------------------------------GCGCGTCCATGACGTCCCCGT---------------------------------------------------      0.049      0.038      0.113
------------------------------------------------------------------------------------------------------------CCATGACGTCCCCGTAGA------------------------------------------------      0.049      0.075      0.075
-------------------------------------------------------------------------------------------------------------CATGACGTCCCCGTAGAG-----------------------------------------------      0.049      0.114      0.075
----------------------------------------------------------------------------------------------------------GTCCATGACGTCCCCGTAGAG-----------------------------------------------      0.097      0.265      0.188
------------------------------------------------------------------------------------------------------------CCATGACGTCCCCGTAGAG-----------------------------------------------      0.097      1.326      1.468
------------------------------------------------------------------------------------------------------------CCATGACGTCCCCGTAGAGCT---------------------------------------------     38.175     30.385     33.133 miR_t42
----------------------------------------------------------------------------------------------------------GTCCATGACGTCCCCGTAGAGCT---------------------------------------------      0.291      0.379      0.226
-----------------------------------------------------------------------------------------------------------TCCATGACGTCCCCGTAGAGCT---------------------------------------------      0.729      0.076      0.151
--------------------------------------------------------------------------------------------------------------ATGACGTCCCCGTAGAGCTAC-------------------------------------------      0.049      0.339      0.339
-------------------------------------------------------------------------------------------------------------------GTCCCCGTAGAGCTACAGGT---------------------------------------      0.049      0.038      0.038
----------------------------------------------------------------------------------------------------------------GACGTCCCCGTAGAGCTACAGGT---------------------------------------      0.049      0.000      0.000
-----------------------------------------------------------------------------------------------------------------ACGTCCCCGTAGAGCTACAGGT---------------------------------------      0.049      0.076      0.151
------------------------------------------------------------------------------------------------------------------CGTCCCCGTAGAGCTACAGGT---------------------------------------     10.296      2.046      2.297
---------------------------------------------------------------------------------------------------------------------------------------------GCATCCGCGCCTCGAGTAGTT------------      0.243      0.000      0.000

miRNA: miR_t54,miR_t50,miR_t15 chromosome_5 Start: 935240 End: 935510 Condition: TAP
-----TACGAGACGTCTGCACCGCAC---------------------------------------------------------------------------------------------------------------      0.049      0.000      0.000
---------AGACGTCTGCACCGCACATG------------------------------------------------------------------------------------------------------------      0.049      0.189      0.075
--------------TCTGCACCGCACATGTCTCAG------------------------------------------------------------------------------------------------------      3.011      0.075      0.075
--------------TCTGCACCGCACATGTCTCAGG-----------------------------------------------------------------------------------------------------      0.923      0.000      0.000
--------------TCTGCACCGCACATGTCTCA-------------------------------------------------------------------------------------------------------      0.534      0.000      0.000
--------------TCTGCACCGCACATGTCTC--------------------------------------------------------------------------------------------------------      0.049      0.000      0.000
----------------TGCACCGCACATGTCTCAGG-----------------------------------------------------------------------------------------------------      2.186      0.303      0.452
----------------TGCACCGCACATGTCTCAG------------------------------------------------------------------------------------------------------      0.049      0.000      0.000
---------------------------TGTCTCAGGAAGAGATCGTC------------------------------------------------------------------------------------------      0.631      0.076      0.113
-----------------------------TCTCAGGAAGAGATCGTC-CG---------------------------------------------------------------------------------------      0.146      0.303      0.151
-----------------------------TCTCAGGAAGAGATCGTC-CGTAGT-----------------------------------------------------------------------------------      2.380      3.789      0.527
-----------------------------TCTCAGGAAGAGATCGTC-CGTA-------------------------------------------------------------------------------------      0.437      1.099      1.092
-----------------------------TCTCAGGAAGAGATCGTC-CGTAG------------------------------------------------------------------------------------     21.321     30.385     23.720 miR_t54
-----------------------------TCTCAGGAAGAGATCGTC-C----------------------------------------------------------------------------------------      0.243      0.720      0.264
-----------------------------TCTCAGGAAGAGATCGTC-CGT--------------------------------------------------------------------------------------      2.477      1.781      1.506
------------------------------CTCAGGAAGAGATCGTC-CGTAG------------------------------------------------------------------------------------      0.049      0.152      0.151
-------------------------------TCAGGAAGAGATCGTC-CGTAG------------------------------------------------------------------------------------      0.486      0.682      0.791
-------------------------------TCAGGAAGAGATCGTC-CGTAGT-----------------------------------------------------------------------------------      0.146      0.113      0.113
-----------------------------------------------------TAGCAAGCTGCTGGAGGGTA----------------------------------------------------------------      0.097      0.076      0.113
---------------------------------------------------------AAGCTGCTGGAGGGT-----------------------------------------------------------------      0.631      0.000      0.000
----------------------------------------------------------------TGGAGGGTATCTCGGTCAGC-----------------------------------------------------      0.097      0.000      0.000
---------------------------------------------------------------------------TCGGTCAGCATCTCGATTG-------------------------------------------      0.097      0.000      0.000
---------------------------------------------------------------------------TCGGTCAGCATCTCGATTGGC-----------------------------------------   1009.635      0.000      0.000 miR_t50
---------------------------------------------------------------------------TCGGTCAGCATCTCGATTGGCA----------------------------------------      0.243      0.000      0.000
---------------------------------------------------------------------------TCGGTCAGCATCTCGATTGG------------------------------------------      7.868      0.000      0.000
----------------------------------------------------------------------------CGGTCAGCATCTCGATTGGC-----------------------------------------      6.071      0.000      0.000
----------------------------------------------------------------------------CGGTCAGCATCTCGATTGG------------------------------------------      0.097      0.000      0.000
-----------------------------------------------------------------------------GGTCAGCATCTCGATTGG------------------------------------------      0.049      0.000      0.000
-----------------------------------------------------------------------------GGTCAGCATCTCGATTGGC-----------------------------------------      1.846      0.000      0.000
-----------------------------------------------------------------------------GGTCAGCATCTCGATT--------------------------------------------      0.049      0.000      0.000
-------------------------------------------------------------------------------TCAGCATCTCGATTGGCAAG--------------------------------------      9.811      0.000      0.000
-------------------------------------------------------------------------------TCAGCATCTCGATTGGCAAGA-------------------------------------      0.194      0.000      0.000
-------------------------------------------------------------------------------TCAGCATCTCGATTGGCAAGACG-----------------------------------      0.097      0.000      0.000
---------------------------------------------------------------------------------------TCGATTGGCAAGACGTCCACT-----------------------------      0.097      0.038      0.038
-------------------------------------------------------------------------------------------TTGGCAAGACGTCCACTCCGG-------------------------      0.194      0.076      0.075
-------------------------------------------------------------------------------------------TTGGCAAGACGTCCACTCCGGG------------------------      1.408      0.871      1.092
--------------------------------------------------------------------------------------------TGGCAAGACGTCCACTCCGG-------------------------      0.049      0.000      0.000
--------------------------------------------------------------------------------------------TGGCAAGACGTCCACTCCGGGT-----------------------      0.049      0.000      0.000
--------------------------------------------------------------------------------------------TGGCAAGACGTCCACTCCGGG------------------------      0.631      0.265      0.753
-----------------------------------------------------------------------------------------------CAAGACGTCCACTCCGGGTAC---------------------      0.243      0.038      0.264
-----------------------------------------------------------------------------------------------CAAGACGTCCACTCCGGGTACG--------------------      0.049      0.000      0.000
------------------------------------------------------------------------------------------------AAGACGTCCACTCCGGGTACG--------------------      0.097      0.038      0.151
------------------------------------------------------------------------------------------------------TCCACTCCGGGTACGCGCCGC--------------      0.194      0.038      0.151
------------------------------------------------------------------------------------------------------TCCACTCCGGGTACGCGCCGCT-------------      0.097      0.000      0.000

-     A  A         A                 A  G      - GTAG            GG                   C   A         C    A  C                         CG
 CCAAT CG GACGTCTGC CCGCACATGTCTCAGGA GA ATCGTC C    TAGCAAGCTGCT  AGGGTATCTCGGTCAGCAT TCG TTGGCAAGA GTCC CT CGGGTACGCGCCGCTCATGCGATGA  C
 GGTTA GC CTGCAGACG GGCGTGTACAGAGTCCT CT TAGCGG G    ATCGTTCGATGA  TCCCATAGAGCCAGTCGTA AGC AACCGTTCT CAGG GA GTCCATGTGCGGCGAGTGCGCTACT  /
T     C  C         C                 C  G      T ATA-            AG                   A   A         A    C  A                         TT

----------------------------------------------------------------------------------------------------------GAAGTCCATGTGCGGCGAGT-----------      0.777      0.076      0.113
---------------------------------------------------------------------------------------------------------CGAAGTCCATGTGCGGCGAGT-----------      0.194      0.227      0.151
------------------------------------------------------------------------------------------------TTCTACAGGCGAAGTCCAT----------------------      0.049      0.076      0.226
----------------------------------------------------------------------------------------------CGTTCTACAGGCGAAGTCCAT----------------------    225.308    278.652    484.491 miR_t15
---------------------------------------------------------------------------------------------CCGTTCTACAGGCGAAGTCCAT----------------------     20.399      7.577      1.920
--------------------------------------------------------------------------------------------ACCGTTCTACAGGCGAAGTCCAT----------------------      0.097      0.114      0.114
-----------------------------------------------------------------------------------------------GTTCTACAGGCGAAGTCCAT----------------------      6.411      2.576      3.125
----------------------------------------------------------------------------------------------CGTTCTACAGGCGAAGTCCA-----------------------      1.797      2.046      6.062
---------------------------------------------------------------------------------------------CCGTTCTACAGGCGAAGTCCA-----------------------      0.097      0.076      0.075
----------------------------------------------------------------------------------------------CGTTCTACAGGCGAAGTCC------------------------      0.437      0.152      0.602
-----------------------------------------------------------------------------------------CAAACCGTTCTACAGGCGAAGT--------------------------      0.146      0.000      0.000
----------------------------------------------------------------------------------------GCAAACCGTTCTACAGGCGAAGT--------------------------      0.049      0.114      0.114
--------------------------------------------------------------------------------GTCGTAAAGCAAACCGTTCT-------------------------------------      0.097      0.000      0.000
-------------------------------------------------------------------------------AGTCGTAAAGCAAACCGTTCT-------------------------------------      0.826      0.075      0.075
--------------------------------------------------------------------------GAGCCAGTCGTAAAGCAAACCGT----------------------------------------      0.049      0.000      0.000
----------------------------------------------------------------------------GCCAGTCGTAAAGCAAACCGT----------------------------------------      2.526      0.265      0.339
----------------------------------------------------------------------------GCCAGTCGTAAAGCAAACCG-----------------------------------------      0.049      0.038      0.038
-------------------------------------------------------------------------AGAGCCAGTCGTAAAGCAAAC-------------------------------------------      0.194      0.000      0.000 miR_t50*
------------------------------------------------------------------------TAGAGCCAGTCGTAAAGCAAA--------------------------------------------      0.049      0.000      0.000
-------------------------------------------------------------------------AGAGCCAGTCGTAAAGCAAA--------------------------------------------      0.340      0.565      0.565
-----------------------------------------------------------------------ATAGAGCCAGTCGTAAAGCAA---------------------------------------------      0.097      0.000      0.000
--------------------------------------------------------------------CCCATAGAGCCAGTCGTAAAG------------------------------------------------      0.049      0.000      0.000
------------------------------------------------------------------GTCCCATAGAGCCAGTCGTAAA-------------------------------------------------      0.049      0.000      0.000
-----------------------------------------------------------------AGTCCCATAGAGCCAGTCGTAA--------------------------------------------------      0.971      1.743      3.502
---------------------------------------------------------------GAAGTCCCATAGAGCCAGTCGT----------------------------------------------------      0.777      2.008      2.008
-----------------------------------------------------------------AGTCCCATAGAGCCAGTCGT----------------------------------------------------      1.554      0.796      0.602
----------------------------------------------------------------AAGTCCCATAGAGCCAGTCGT----------------------------------------------------      0.680      0.379      0.379
----------------------------------------------------------------AAGTCCCATAGAGCCAGTCG-----------------------------------------------------      0.049      0.000      0.000
-------------------------------------------------------------ATGAAGTCCCATAGAGCCAGT-------------------------------------------------------      0.777      0.644      1.092
-----------------------------------------------------------CGATGAAGTCCCATAGAGCCAGT-------------------------------------------------------      0.049      0.152      0.152
-------------------------------------------------------CGTTCGATGAAGTCCCATAGA-------------------------------------------------------------      0.049      0.000      0.000
---------------------------------------------------A-ATCGTTCGATGAAGTCCCAT----------------------------------------------------------------      1.748      0.113      0.113
--------------------------------------------------TA-ATCGTTCGATGAAGTCCCAT----------------------------------------------------------------      0.049      0.000      0.000
------------------------------------------------------TCGTTCGATGAAGTCCCAT----------------------------------------------------------------      0.049      0.038      0.038
--------------------------TACAGAGTCCTCCTGTAGCGGTGAT--------------------------------------------------------------------------------------      0.049      0.000      0.000
----------------------------CAGAGTCCTCCTGTAGCGGTGAT--------------------------------------------------------------------------------------      0.049      0.227      0.227
```

```
----------------------------AGAGTCCTCCTGTAGCGGTGAT--------------------------------------------------------------------------------    0.340    0.038    0.038
--------------------------ACAGAGTCCTCCTGTAGCGGT-----------------------------------------------------------------------------------    0.097    0.000    0.000
-------------------GGCGTGTACAGAGTCCTCCTGT-----------------------------------------------------------------------------------------    3.691    0.530    0.301
----------------CGCGGCGTGTACAGAGTCCTCCT-------------------------------------------------------------------------------------------    0.291    0.114    0.114
-------------------GGCGTGTACAGAGTCCTCCT-------------------------------------------------------------------------------------------    0.340    0.000    0.000
-----------------GCGGCGTGTACAGAGTCCTCCT-------------------------------------------------------------------------------------------    0.097    0.000    0.000
------------------CGGCGTGTACAGAGTCCTCCT-------------------------------------------------------------------------------------------    0.146    0.226    0.226
------------------CGGCGTGTACAGAGTCCTCC--------------------------------------------------------------------------------------------    0.049    0.000    0.000
----------------CGCGGCGTGTACAGAGTCCTC---------------------------------------------------------------------------------------------    0.291    0.114    0.038
---------------ACGCGGCGTGTACAGAGTCCT----------------------------------------------------------------------------------------------    4.614    0.644    1.318
--------------GACGCGGCGTGTACAGAGT-------------------------------------------------------------------------------------------------    0.049    0.038    0.038
-----------GCAGACGCGGCGTGTACAGAGT-------------------------------------------------------------------------------------------------   21.370   14.889   16.152
-------------AGACGCGGCGTGTACAGAGT-------------------------------------------------------------------------------------------------    1.554    0.379    1.092
---------------ACGCGGCGTGTACAGAGT-------------------------------------------------------------------------------------------------    0.097    0.000    0.000
-----------GCAGACGCGGCGTGTACAGAG--------------------------------------------------------------------------------------------------    0.049    0.038    0.113
----TACGCCCTGCAGACGCGGCGTGT-------------------------------------------------------------------------------------------------------    0.049    0.000    0.000
-------GCCCTGCAGACGCGGCGTGT-------------------------------------------------------------------------------------------------------    8.985    0.114    0.151
--------CCCTGCAGACGCGGCGTGT-------------------------------------------------------------------------------------------------------    0.389    0.038    0.038
```

miRNA: miR_t55 chromosome_12 Start: 9542432 End: 9542883 Condition: TAP

```
---------------------------------------------------------------------------------------------------------------------------------------------------------------------------------------TGTGCCAGGTGCCCCGTCGCAGC------------------------    0.049    0.075    0.075
---------------------------------------------------------------------------------------------------------------------------------------------------------------------------------------TGTGCCAGGTGCCCCGTCGC---------------------------    0.049    0.000    0.000
-----------------------------------------------------------------------------------------------------------------------------------------------------------------------------TGCTGCCGTGTGCCAGGTGC-------------------------------------    0.049    0.000    0.000
--------------------------------------------------------------------------------------------------------------------------------------------------------------------TGACCGGTCGGTGCTGCCGTG---------------------------------------------    0.049    0.038    0.038
--------------------------------------------------------------------------------------------------------------------------------------------------------------------TGACCGGTCGGTGCTGCCG-----------------------------------------------    0.291    0.076    0.188
-------------------------------------------------------------------------------------------------------------------------------------------------------------------CTGACCGGTCGGTGCTGCCG-----------------------------------------------    0.097    0.038    0.414
------------------------------------------------------------------------------------------------------------------------------------------------------------------TCTGACCGGTCGGTGCTGCCGT----------------------------------------------    0.194    0.038    0.038
------------------------------------------------------------------------------------------------------------------------------------------------------------------TCTGACCGGTCGGTGC----------------------------------------------------    0.049    0.000    0.000
------------------------------------------------------------------------------------------------------------------------------------------------------------------TCTGACCGGTCGGTGCTGCCG-----------------------------------------------  231.087   39.326  126.356 miR_t55
------------------------------------------------------------------------------------------------------------------------------------------------------------------TCTGACCGGTCGGTGCTGC-------------------------------------------------    0.097    0.000    0.000
------------------------------------------------------------------------------------------------------------------------------------------------------------------TCTGACCGGTCGGTGCTGCC------------------------------------------------    0.049    0.038    0.151
------------------------------------------------------------------------------------------------------------------------------------------------------------------TCTGACCGGTCGGT------------------------------------------------------    0.049    0.000    0.000
------------------------------------------------------------------------------------------------------------------------------TCA-GTTGCAGACAGGTCGGAG--------------------------------------------------------------------------------    0.340    0.339    0.339
------------------------------------------------------------------------------------------------------------------------ATGGGCTCA-GTTGCAGACAGG--------------------------------------------------------------------------------------    0.146    0.038    0.038
-------------------------------------------------------------------------------------------------------GGGATGGGAGCGGCAGCATG---------------------------------------------------------------------------------------------------------    0.049    0.000    0.000
------------------------------------------------------------------------------------------------------TGGGATGGGAGCGGCAGCATG---------------------------------------------------------------------------------------------------------    0.389    0.303    0.791
-------------------------------------------------------------------------------------------------CAGCTTGGGATGGGAGCGGCA--------------------------------------------------------------------------------------------------------------    0.049    0.000    0.000
------------TCAGGGGCCTTGACGCCAGC------------------------------------------------------------------------------------------------------------------------------------------------------------------------------------------------    0.049    0.114    0.113
------------TCAGGGGCCTTGACGCCAGCT-----------------------------------------------------------------------------------------------------------------------------------------------------------------------------------------------    0.049    0.000    0.000
------TGGACATCAGGGGCCTTGACGC----------------------------------------------------------------------------------------------------------------------------------------------------------------------------------------------------    2.186    5.342    1.431
------TGGACATCAGGGGCCTTGAC------------------------------------------------------------------------------------------------------------------------------------------------------------------------------------------------------    0.146    0.188    0.188
------TGGACATCAGGGGCCTTGACG-----------------------------------------------------------------------------------------------------------------------------------------------------------------------------------------------------    0.340    0.152    0.188

--           A      T       A                                      G                              CA                             A-          G                        C                               TC  A CC    CA    A   ACCCA  T
  GGCCTGGACATC GGGGCC TGACGCC GCTGCGCTGCCGCCAGCGGTGCGGCGCCTGTGGCGCCA CGGCGCAGGCCCTCGTCCCTCGCCACAA  GCTTGGGATGGGAGCGGCAGCATGGGCTC  GTTGCAGACA GTCGGAGCGGTCCGAGCGATCTGA CGGTCGGTGCTGCCGTGTGCCAGGTGCCCCG  GC G  CTCG  AGCC TGG     GC C
  CTGGATCTGTAG CCCCGG ACTGCGG CGACGCGACGGCGGTTGCCACGCCGCGGACACCGCGGT GCCGCGTCCGGGAGCAGGGAGCGGTGTT  CGAACCCTACCCTCGCCGTCGTGCCCGAG  CAACGTCTGT CAGCCTCGTCAGGCTCGCTAGGCT GTCAGCCACGACGGCACACGGTCCACGGGGT  CG C  GAGT  TCGG GCC      CG G
CG           C      C       A                                      A                              AC                             CC          G                        T                               --  A AA    AG    C   GCCCG  G

----GGATCTGTAGCCCCCGGCACT-------------------------------------------------------------------------------------------------------------------------------------------------------------------------------------------    0.291    0.038    0.151
-------------------------------------------------------------------------CGTCCGGGAGCAGGGAGCGG---------------------------------------------------------------------------------------------------------------------------------------    0.049    1.017    1.017
--------------------------------------------------------------------------GTCCGGGAGCAGGGAGCGGT--------------------------------------------------------------------------------------------------------------------------------------    0.680    0.377    0.377
-------------------------------------------------------------------------CGTCCGGGAGCAGGGAGCGGT--------------------------------------------------------------------------------------------------------------------------------------   13.016    5.493   39.232
---------------------------------------------------------------------------TCCGGGAGCAGGGAGCGGTG-------------------------------------------------------------------------------------------------------------------------------------    0.049    0.038    0.038
----------------------------------------------------------------------------CCGGGAGCAGGGAGCGGTGT------------------------------------------------------------------------------------------------------------------------------------    0.049    0.075    0.075
---------------------------------------------------------------------------TCCGGGAGCAGGGAGCGGTGT------------------------------------------------------------------------------------------------------------------------------------    0.049    0.075    0.075
-----------------------------------------------------------------------------CGGGAGCAGGGAGCGGTGTT-----------------------------------------------------------------------------------------------------------------------------------    0.049    0.038    0.038
----------------------------------------------------------------------------CCGGGAGCAGGGAGCGGTGTT-----------------------------------------------------------------------------------------------------------------------------------    0.049    0.038    0.038
----------------------------------------------------------------------------------------------------GAACCCTACCCTCGCCGTCGT-----------------------------------------------------------------------------------------------------------    0.049    0.038    0.188
-------------------------------------------------------------------------------------------------------------------CGTCGTGCCCGAGCCCAACGT--------------------------------------------------------------------------------------------    0.194    0.000    0.000
------------------------------------------------------------------------------------------------------------------CCGTCGTGCCCGAGCCCAACGT--------------------------------------------------------------------------------------------    0.049    0.000    0.000
-------------------------------------------------------------------------------------------------------------------CGTCGTGCCCGAGCCCAACGTCT------------------------------------------------------------------------------------------    0.146    0.000    0.000
--------------------------------------------------------------------------------------------------------------------GTCGTGCCCGAGCCCAACGTCT------------------------------------------------------------------------------------------    0.049    0.000    0.000
---------------------------------------------------------------------------------------------------------------------TCGTGCCCGAGCCCAACGTCT------------------------------------------------------------------------------------------    0.049    0.000    0.000
----------------------------------------------------------------------------------------------------------------------CGTGCCCGAGCCCAACGTCTGT----------------------------------------------------------------------------------------    0.437    0.227    0.414
----------------------------------------------------------------------------------------------------------------------------------CAACGTCTGTGCAGCCTCGT------------------------------------------------------------------------------    0.049    0.000    0.000
-----------------------------------------------------------------------------------------------------------------------------------------------GCCTCGTCAGGCTCGCTAGGC----------------------------------------------------------------    0.049    0.000    0.000
-----------------------------------------------------------------------------------------------------------------------------------------------GCCTCGTCAGGCTCGCTAGGCT---------------------------------------------------------------    1.603    1.819    1.431
------------------------------------------------------------------------------------------------------------------------------------------------CCTCGTCAGGCTCGCTAGGCTT--------------------------------------------------------------    0.049    0.000    0.000
--------------------------------------------------------------------------------------------------------------------------------------------------TCGTCAGGCTCGCTAGGCTT--------------------------------------------------------------    0.049    0.000    0.000
--------------------------------------------------------------------------------------------------------------------------------------------------------------------TTGTCAGCCACGACGGCACACGG-----------------------------------------    0.049    0.000    0.000
--------------------------------------------------------------------------------------------------------------------------------------------------------------------TTGTCAGCCACGACGGCACACGGT----------------------------------------    0.097    0.152    0.152
----------------------------------------------------------------------------------------------------------------------------------------------------------------------GTCAGCCACGACGGCACACGGT----------------------------------------    0.049    0.000    0.000
---------------------------------------------------------------------------------------------------------------------------------------------------------------------TGTCAGCCACGACGGCACACGGT----------------------------------------    0.194    0.076    0.075
```

miRNA: miR_t57 chromosome_2 Start: 9121113 End: 9121295 Condition: TAP

```
-----------------------------------------------GCTCGCTGGGTTTATCATC----------------------------------------------    0.049    0.000    0.000
----------------------------------------------TGCTCGCTGGGTTTATCATCC---------------------------------------------    0.291    0.038    0.226
----------------------------------------------TGCTCGCTGGGTTTATCATC----------------------------------------------    0.194    0.226    0.226
-------------------------------------------TGATGCTCGCTGGGTTTATCA------------------------------------------------    0.049    0.000    0.000
----TGGCTGGAGTGCAGCGCAGAGC--------------------------------------------------------------------------------------    0.389    1.061    2.485

GGGCTGGCTGGAGTGCAGCGCAGAGCTGGGCG     C                 G       AT     -     CCA    T G  A-    G    A-- - G    G
                                GCCGT TCCGCTGATGCTCGCTG GTTTATC  CCGCT TTGGC   TGAT G TC  CAAC CCGG   G G CTGC T
                                CGGCA AGGCGACTACGAGCGGC CAAGTAG  GGCGA AGCCG   ATTA C AG  GTTG GGCC   C C GACG C
--------------------------------     A                 A       CG     T     ---    - G  AC    -    GTA G G    C

---------------------------------------GGCGACTACGAGCGGCACAAGT---------------------------------------------------   59.787    2.160    9.488 miR_t57
----------------------------------------GCGACTACGAGCGGCACAAGT---------------------------------------------------   38.077    0.114    0.753
--------------------------------------AGGCGACTACGAGCGGCACAAGT---------------------------------------------------    0.340    0.000    0.000
------------------------------------AAAGGCGACTACGAGCGGCACAAGT---------------------------------------------------    0.049    0.000    0.000
-----------------------------------------CGACTACGAGCGGCACAAGT---------------------------------------------------    0.923    0.227    0.188
--------------------------------------------------GCGGCACAAGTAGCGGGCGAT-----------------------------------------    0.097    0.417    0.226
```

miRNA: miR_t59 chromosome_4 Start: 1804230 End: 1804569 Condition: TAP

```
----------------TGCCATCCCACCCTACGCTGT---------------------------------------------------------------------------------------------------------------------------------------------    0.049    0.000    0.000
---------------------TCCCACCCTACGCTGTCGGCT----------------------------------------------------------------------------------------------------------------------------------------    0.049    0.000    0.000
-----------------------------TACGCTGTCGGCTGAGCTCACC-------------------------------------------------------------------------------------------------------------------------------    0.049    0.189    0.038
------------------------------------TCGGCTGAGCTCACCGCGGG--------------------------------------------------------------------------------------------------------------------------    0.826    0.189    0.527
------------------------------------TCGGCTGAGCTCACCGCGGGT-------------------------------------------------------------------------------------------------------------------------    0.146    0.038    0.038
-------------------------------------CGGCTGAGCTCACCGCGGGTC------------------------------------------------------------------------------------------------------------------------    0.049    0.000    0.000
-----------------------------------------------------------------TCATGTTCTCGATCAGCTGCG--------------------------------------------------------------------------------------------    0.680    0.265    0.377
-----------------------------------------------------------------TCATGTTCTCGATCAGCTGCGT-------------------------------------------------------------------------------------------    0.534    0.265    0.377
----------------------------------------------------------------------TTCTCGATCAGCTGCGTAGC----------------------------------------------------------------------------------------    0.194    0.720    0.678
----------------------------------------------------------------------TTCTCGATCAGCTGCGTAGCC---------------------------------------------------------------------------------------    8.888    3.561    1.958
----------------------------------------------------------------------TTCTCGATCAGCTGCGTAGCCT--------------------------------------------------------------------------------------    0.534    0.189    0.075
----------------------------------------------------------------------TTCTCGATCAGCTGCGTAGCCTT-------------------------------------------------------------------------------------    0.194    0.076    0.113
-----------------------------------------------------------------------TCTCGATCAGCTGCGTAGCC---------------------------------------------------------------------------------------    0.097    0.000    0.000
-------------------------------------------------------------------------TCGATCAGCTGCGTAGCCTTG------------------------------------------------------------------------------------    0.049    0.038    0.038
-------------------------------------------------------------------------TCGATCAGCTGCGTAGCCTTGG-----------------------------------------------------------------------------------    0.194    0.075    0.075
-----------------------------------------------------------------------------TCAGCTGCGTAGCCTTGGGAGC-------------------------------------------------------------------------------    0.534    0.758    0.758
----------------------------------------------------------------------------------TGCGTAGCCTTGGGAGCCGGCA--------------------------------------------------------------------------    0.194    0.227    0.264
----------------------------------------------------------------------------------TGCGTAGCCTTGGGAGCCGGC---------------------------------------------------------------------------    2.720    5.569   22.214
-----------------------------------------------------------------------------------GCGTAGCCTTGGGAGCCGGCA--------------------------------------------------------------------------    0.049    0.000    0.000
-----------------------------------------------------------------------------------GCGTAGCCTTGGGAGCCGGC---------------------------------------------------------------------------    0.097    0.038    0.527
--------------------------------------------------------------------------------------TAGCCTTGGGAGCCGGCAGGG-----------------------------------------------------------------------    0.291    0.227    0.452
--------------------------------------------------------------------------------------TAGCCTTGGGAGCCGGCAGGGC----------------------------------------------------------------------    0.049    0.075    0.075
--------------------------------------------------------------------------------------TAGCCTTGGGAGCCGGCAGG------------------------------------------------------------------------    0.049    0.000    0.000
-------------------------------------------------------------------------------------------TTGGGAGCCGGCAGGGCCAGC------------------------------------------------------------------    2.477    0.417    2.749
--------------------------------------------------------------------------------------------TGGGAGCCGGCAGGGCCAGCG-----------------------------------------------------------------    0.097    0.075    0.075
-----------------------------------------------------------------------------------------------------------------TGAGCTGCAGGGTGGTCATGT--------------------------------------------    0.194    0.038    0.038
-----------------------------------------------------------------------------------------------------------------TGAGCTGCAGGGTGGTCATGTGG------------------------------------------    0.146    0.114    0.114
-----------------------------------------------------------------------------------------------------------------TGAGCTGCAGGGTGGTCATG---------------------------------------------    0.874    0.189    0.414
------------------------------------------------------------------------------------------------------------------GAGCTGCAGGGTGGTCATGTGG------------------------------------------    0.049    0.000    0.000
-------------------------------------------------------------------------------------------------------------------AGCTGCAGGGTGGTCATGTGG------------------------------------------    0.049    0.038    0.038
----------------------------------------------------------------------------------------------------------------------TGCAGGGTGGTCATGTGGGACA--------------------------------------    0.243    0.000    0.000
----------------------------------------------------------------------------------------------------------------------TGCAGGGTGGTCATGTGGG-----------------------------------------    0.097    0.076    0.038
----------------------------------------------------------------------------------------------------------------------TGCAGGGTGGTCATGTGGGA----------------------------------------    0.194    0.000    0.000
----------------------------------------------------------------------------------------------------------------------TGCAGGGTGGTCATGTGGGAC---------------------------------------   16.562    2.766    7.041
-----------------------------------------------------------------------------------------------------------------------GCAGGGTGGTCATGTGGGAC---------------------------------------    0.146    0.000    0.000
------------------------------------------------------------------------------------------------------------------------CAGGGTGGTCATGTGGGACAG-------------------------------------    0.049    0.151    0.151
------------------------------------------------------------------------------------------------------------------------CAGGGTGGTCATGTGGGACA--------------------------------------    0.049    0.000    0.000
-----------------------------------------------------------------------------------------------------------------------------TGGTCATGTGGGACAGATCCCC-------------------------------    0.097    0.038    0.038
-----------------------------------------------------------------------------------------------------------------------------TGGTCATGTGGGACAGATCCC--------------------------------    1.020    0.227    0.264
------------------------------------------------------------------------------------------------------------------------------GGTCATGTGGGACAGATCCC--------------------------------    0.049    0.000    0.000
-----------------------------------------------------------------------------------------------------------------------------------TGTGGGACAGATCCCCAACGG--------------------------    0.194    0.114    0.151
-------------------------------------------------------------------------------------------------------------------------------------TGGGACAGATCCCCAACGGCT-G----------------------    0.049    0.000    0.000
-------------------------------------------------------------------------------------------------------------------------------------TGGGACAGATCCCCAACGGCT------------------------    0.729    0.455    1.581
-------------------------------------------------------------------------------------------------------------------------------------TGGGACAGATCCCCAACGGC-------------------------    0.097    0.114    0.339
--------------------------------------------------------------------------------------------------------------------------------------GGGACAGATCCCCAACGGCT------------------------    0.049    0.000    0.000

------------  C      C      TAC     C     A                     T    TTC           C       T                      G                                  C    -    T    ATT     A  CG
            CCA TGCCAT CCACCC   GCTGT GGCTG GCTCACCGCGGGTCAGGCGGG CATG   TCGATCAGCTG GTAGCCT GGGAGCCGGCAGGGCCAGCGT AGCTGCAGGGTGGTCATGTGGGACAGATCCCCAA GGCT GCTG CGCG   GCCCT GG  G
            GGT ACGGTA GGTGGG   CGACA CCGAC CGAGTGGCGCCCAGTCCGCCC GTAC   AGTTAGTCGAC CATCGGA CCCTCGGCCGTCCCGGTCGCA TCGACGTCCCACCAGTACACCCTGTCTAGGGGTT CCGG CGGC GCGC   CGGGG CC  /
ACGCACGGCGCG  A      A      CGT     A     G                     T    CTA           C       C                      G                                  A    T    -    AC-     -  AA

---------------------------------------------------------------------------------------------------------------------GACGTCCCACCAGTACACCCT----------------------------------------    0.146    0.000    0.000
--------------------------------------------------------------------------------------------------------------------CGACGTCCCACCAGTACACCCT----------------------------------------    0.049    0.000    0.000
-------------------------------------------------------------------------------------------------------------TCGCAGTCGACGTCCCACCAGT-----------------------------------------------    0.777    0.000    0.000
---------------------------------------------------------------------------------------------------------------GCAGTCGACGTCCCACCAGT-----------------------------------------------    2.866    0.303    0.226
--------------------------------------------------------------------------------------------------------------CGCAGTCGACGTCCCACCAGT-----------------------------------------------  117.292    2.804    9.752 miR_t59
------------------------------------------------------------------------------------------------------------GTCGCAGTCGACGTCCCACCAGT-----------------------------------------------    0.049    0.000    0.000
--------------------------------------------------------------------------------------------------------------CGCAGTCGACGTCCCACCAG------------------------------------------------    0.097    0.075    0.075
--------------------------------------------------------------------------------------------------------------CGCAGTCGACGTCCCACCA-------------------------------------------------    0.049    0.000    0.000
-----------------------------------------------------------CCGCCCTGTACCTAAGTTAGT--------------------------------------------------------------------------------------------------    0.243    0.152    0.075
-----------------------------------------------------------CCGCCCTGTACCTAAGTTAG---------------------------------------------------------------------------------------------------    0.049    0.000    0.000
------------------------------------------------------CCAGTCCGCCCTGTACCTAAGT------------------------------------------------------------------------------------------------------    0.049    0.000    0.000
-----------------------------------CAACCGACGCGAGTGGCGCCCAGT-----------------------------------------------------------------------------------------------------------------------    0.049    0.000    0.000
---------------------------------------CGACGCGAGTGGCGCCCAGT-----------------------------------------------------------------------------------------------------------------------    0.049    0.000    0.000
----ACGGCGCGGGTAACGGTAAGGT--------------------------------------------------------------------------------------------------------------------------------------------------------    0.049    0.038    0.038
-----CGGCGCGGGTAACGGTAAGGT--------------------------------------------------------------------------------------------------------------------------------------------------------    0.049    0.152    0.377
```

miRNA: miR_t62 chromosome_17 Start: 6673966 End: 6674281 Condition: TAP

```
-------------------------------------------------------------------------------------------------------TCAAGACCGGGGGATGGAAGG-------------------------------------------------------------    1.700    1.932    1.920
-------------------------------------------------------------------------------------------------------TCAAGACCGGGGGATGGAAG--------------------------------------------------------------    0.291    0.265    0.377
------------------------------------------------------------------------------------------------------ATCAAGACCGGGGGATGGAAG--------------------------------------------------------------    0.049    0.000    0.000
-------------------------------------------------------------------------------------------------GCAAAATCAAGACCGGGGGA--------------------------------------------------------------------    1.457    0.151    0.151
```

```
------------------------------------------------------------------------------------------------TGCAAAATCAAGACCGGGG----------------------------------------------------------------------     0.049     0.264     0.264
------------------------------------------------------------------------------------------------TGCAAAATCAAGACCGGGGG---------------------------------------------------------------------     0.486     0.152     0.753
------------------------------------------------------------------------------------------------TGCAAAATCAAGACCGGGGGAT-------------------------------------------------------------------     0.146     0.000     0.000
------------------------------------------------------------------------------------------------TGCAAAATCAAGACCGGGGGA--------------------------------------------------------------------     60.370    6.213     15.700 miR_t62
-----------------------------------------------------------------------------------------------TTGCAAAATCAAGACCGGGGGA--------------------------------------------------------------------     0.146     0.038     0.075
-----------------------------------------------------------------------------------------------TTGCAAAATCAAGACCGGGG----------------------------------------------------------------------     0.097     0.038     0.038
-----------------------------------------------------------------------------------------------TTGCAAAATCAAGACCGGGGG---------------------------------------------------------------------     0.874     0.227     0.339
----------------------------------------------------------------------------------------------TTTGCAAAATCAAGACCGGG-----------------------------------------------------------------------     0.194     0.114     0.075
----------------------------------------------------------------------------------------------TTTGCAAAATCAAGACCGG------------------------------------------------------------------------     0.146     0.076     0.113
----------------------------------------------------------------------------------------------TTTGCAAAATCAAGACCGGGG----------------------------------------------------------------------     4.614     0.455     0.640
----------------------------------------------------------------------------------------------TTTGCAAAATCAAGACCGGGGG---------------------------------------------------------------------     1.068     0.076     0.076
-----------------------------------------------------------------------TAAGGCGTGTTGATGCATTC----------------------------------------------------------------------------------------------     0.049     0.000     0.000
-----------------------------------------------------------------TGACAGTAAGGCGTGTTGATG---------------------------------------------------------------------------------------------------     0.971     1.099     1.017
-----------------------------------------------------------------TGACAGTAAGGCGTGTTGATGC--------------------------------------------------------------------------------------------------     0.729     0.152     0.188
---------------------------------------------------------------AATGACAGTAAGGCGTGTTGA-----------------------------------------------------------------------------------------------------     0.049     0.000     0.000
-------------------------------------------------------------CAAATGACAGTAAGGCGTGT--------------------------------------------------------------------------------------------------------     0.097     0.000     0.000
---------------------------------------TGGGACCCGCACATGCATG-------------------------------------------------------------------------------------------------------------------------------     0.049     0.000     0.000
--------------------------------------TTGGGACCCGCACATGCATG-------------------------------------------------------------------------------------------------------------------------------     0.291     0.114     0.188
--------------------------------------TTGGGACCCGCACATGCATGT------------------------------------------------------------------------------------------------------------------------------     0.049     0.000     0.000
----------------------------------TTGCTTGGGACCCGCACATG-----------------------------------------------------------------------------------------------------------------------------------     0.389     0.038     0.339
----------------------------------TTGCTTGGGACCCGCACATGC----------------------------------------------------------------------------------------------------------------------------------     0.146     0.114     0.113
------------------------------TGACTTGCTTGGGACCCGC----------------------------------------------------------------------------------------------------------------------------------------     0.583     7.009     7.191
------------------------------TGACTTGCTTGGGACCCGCA---------------------------------------------------------------------------------------------------------------------------------------     0.097     0.076     0.151
------------------------------TGACTTGCTTGGGACCCGCAC--------------------------------------------------------------------------------------------------------------------------------------     0.049     0.076     0.038
------------------------------TGACTTGCTTGGGACCCGCACA-------------------------------------------------------------------------------------------------------------------------------------     0.194     0.000     0.000
-----------------------TGAACAATGACTTGCTTGGGACC-------------------------------------------------------------------------------------------------------------------------------------------     0.971     0.833     0.833
-----------------------TGAACAATGACTTGCTTGGGA---------------------------------------------------------------------------------------------------------------------------------------------     0.729     0.152     0.038
-----------------------TGAACAATGACTTGCTTGG-----------------------------------------------------------------------------------------------------------------------------------------------     0.097     0.000     0.000
-----------------------TGAACAATGACTTGCTTGGGAC--------------------------------------------------------------------------------------------------------------------------------------------     2.283     0.076     0.076
-----------------------TGAACAATGACTTGCTTGGG----------------------------------------------------------------------------------------------------------------------------------------------     2.963     0.189     0.151
----------------TGGGACGTGAACAATGACTTGCT--------------------------------------------------------------------------------------------------------------------------------------------------     0.049     0.000     0.000
----------------TGGGACGTGAACAATGACTT-----------------------------------------------------------------------------------------------------------------------------------------------------     0.049     0.000     0.000
----------------TGGGACGTGAACAATGACTTG----------------------------------------------------------------------------------------------------------------------------------------------------     0.340     0.455     0.151
----------------TGGGACGTGAACAATGACTTGC---------------------------------------------------------------------------------------------------------------------------------------------------     0.146     0.530     0.377
----------------TGGGACGTGAACAATGAC-------------------------------------------------------------------------------------------------------------------------------------------------------     0.049     0.114     0.038
---------------TTGGGACGTGAACAATGAC-------------------------------------------------------------------------------------------------------------------------------------------------------     13.405    2.463     2.485
---------------TTGGGACGTGAACAATGACT------------------------------------------------------------------------------------------------------------------------------------------------------     0.340     0.076     0.188
-----------TGCGTTGGGACGTGAACAATG---------------------------------------------------------------------------------------------------------------------------------------------------------     0.097     0.000     0.000
----------TTGCGTTGGGACGTGAACAATG---------------------------------------------------------------------------------------------------------------------------------------------------------     0.146     0.038     0.038

------                                                     C                                                               TA   TGGTGGAGGGGAGAAGGCCACCCGTCGGCACGCGCGCCAACCCATACATCC     G
      CGGCTTGCGTTGGGACGTGAACAATGACTTGCTTGGGACCCGCACATGCATGT ACAAATGACAGTAAGGCGTGTTGATGCATTCAGATTTGCAAAATCAAGACCGGGGGATGGAAGG  TG                                                   ACGC T
      GCCGAACGCAACCCTGCACTTGTTACTGAACGAACCCTGGGCGTGTACGTACA TGTTTACTGTCATTCCGCACAACTACGTAAGTCTAAACGTTTTAGTTCTGGCCCCCTACCTTCC  AC                                                   TGCG A
CCGTGG                                                     A                                                               CC   TC------------------------------------------------     A

----GGGCCGAACGCAACCCTGCACT---------------------------------------------------------------------------------------------------------------------------------------------------------------     0.049     0.075     0.075
-------CCGAACGCAACCCTGCACTT--------------------------------------------------------------------------------------------------------------------------------------------------------------     0.049     0.000     0.000
-----GGCCGAACGCAACCCTGCACTT--------------------------------------------------------------------------------------------------------------------------------------------------------------     0.049     0.227     0.227
--------CGAACGCAACCCTGCACTTGT------------------------------------------------------------------------------------------------------------------------------------------------------------     0.049     0.000     0.000
---------GAACGCAACCCTGCACTTGTT-----------------------------------------------------------------------------------------------------------------------------------------------------------     0.971     0.038     0.038
-----------ACGCAACCCTGCACTTGTT-----------------------------------------------------------------------------------------------------------------------------------------------------------     0.049     0.000     0.000
--------CGAACGCAACCCTGCACTTGTT-----------------------------------------------------------------------------------------------------------------------------------------------------------     0.049     0.038     0.038
-------------GCAACCCTGCACTTGTTACT--------------------------------------------------------------------------------------------------------------------------------------------------------     0.049     0.000     0.000
---------------------------GTTACTGAACGAACCCTGGGCGT---------------------------------------------------------------------------------------------------------------------------------------     0.049     0.000     0.000
------------------------------ACTGAACGAACCCTGGGCGTGT-------------------------------------------------------------------------------------------------------------------------------------     0.049     0.076     0.076
-------------------------------CTGAACGAACCCTGGGCGTGT-------------------------------------------------------------------------------------------------------------------------------------     0.437     0.114     0.301
------------------------------------CGAACCCTGGGCGTGTACGT---------------------------------------------------------------------------------------------------------------------------------     0.049     0.038     0.038
--------------------------------------------GGGCGTGTACGTACAATGTTT------------------------------------------------------------------------------------------------------------------------     0.049     0.000     0.000
-------------------------------------------------------------GTTTACTGTCATTCCGCACAA-------------------------------------------------------------------------------------------------------     0.097     0.000     0.000
---------------------------------------------------------------TTACTGTCATTCCGCACAACT-----------------------------------------------------------------------------------------------------     0.049     0.152     0.038
-------------------------------------------------------------------TGTCATTCCGCACAACTACGT-------------------------------------------------------------------------------------------------     0.777     0.114     0.114
-----------------------------------------------------------------------ATTCCGCACAACTACGTAAGT---------------------------------------------------------------------------------------------     0.049     0.000     0.000
----------------------------------------------------------------------CATTCCGCACAACTACGTAAGT---------------------------------------------------------------------------------------------     0.146     0.000     0.000
--------------------------------------------------------------------------CCGCACAACTACGTAAGTCT-------------------------------------------------------------------------------------------     0.437     0.038     0.038
------------------------------------------------------------------------TTCCGCACAACTACGTAAGTCT-------------------------------------------------------------------------------------------     0.049     0.000     0.000
-------------------------------------------------------------------------TCCGCACAACTACGTAAGTCT-------------------------------------------------------------------------------------------     3.011     0.189     0.038
---------------------------------------------------------------------------CGCACAACTACGTAAGTCTAAA----------------------------------------------------------------------------------------     0.049     0.000     0.000
----------------------------------------------------------------------------------CTACGTAAGTCTAAACGTT------------------------------------------------------------------------------------     0.049     0.000     0.000
-------------------------------------------------------------------------------------CGTAAGTCTAAACGTTTTAG--------------------------------------------------------------------------------     0.049     0.038     0.038
------------------------------------------------------------------------------------ACGTAAGTCTAAACGTTTTAGT-------------------------------------------------------------------------------     0.243     0.379     0.379
-------------------------------------------------------------------------------------CGTAAGTCTAAACGTTTTAGT-------------------------------------------------------------------------------     3.400     4.622     1.280
------------------------------------------------------------------------------------------------ACGTTTTAGTTCTGGCCCCCT--------------------------------------------------------------------     0.049     0.000     0.000
```

miRNA: miR_t67 chromosome_16 Start: 2231046 End: 2231431 Condition: TAP

```
---------------------------------------------------------------------------------------------TCCTACGCAAGCGATC-GCCAT-----------     0.049     0.000     0.000
---------------------------------------------------------------------------------------------TCCTACGCAAGCGATC-GCCATG----------     0.243     0.076     0.075
--------------------------------------------------------------------------------------TACCACGTCCTACGCAAGCGA-------------------     0.291     0.000     0.000
--------------------------------------------------------------------------------TGCGCCTACCACGTCCTACGCA------------------------     0.389     0.000     0.000
--------------------------------------------------------------------------------TGCGCCTACCACGTCCTACGC-------------------------     0.291     0.076     0.076
--------------------------------------------------------------------------------TGCGCCTACCACGTCCTACGCAAG----------------------     0.049     0.076     0.076
----------------------------------------------------------------------TCCTTGCTGTGCGCCTACCACG----------------------------------     0.923     0.265     0.301
--------------------------------------------------------------------TGTCCTTGCTGTGCGCCTACC-------------------------------------     0.049     0.038     0.038
--------------------------------------------------------------------TGTCCTTGCTGTGCGCCTACCA------------------------------------     0.049     0.000     0.000
----------------------------------------------------------------CGACTGTCCTTGCTGTGCG-------------------------------------------     0.049     0.000     0.000
-------------------------------------------------------------TGGCGACTGTCCTTGCTGTGCG-------------------------------------------     3.400     2.917     0.866
-------------------------------------------------------------TGGCGACTGTCCTTGCTGTGC--------------------------------------------     3.448     14.472    3.276
------------------------------------------------------------TTGGCGACTGTCCTTGCTGTG---------------------------------------------     0.243     0.114     0.038
------------------------------------------------------------TTGGCGACTGTCCTTGCTGTGCG-------------------------------------------     0.146     0.379     0.379
------------------------------------------------------------TTGGCGACTGTCCTTGCTGTGC--------------------------------------------     5.537     16.821    3.238
-------------------------------------CCGCCACCCCGCAGCCCGCAC--------------------------------------------------------------------     0.146     0.038     0.038
---------------------------------TCCGCCGCCACCCCGCAGCCCG-----------------------------------------------------------------------     0.146     0.076     0.113
---------------------------------TCCGCCGCCACCCCGCAGCCC------------------------------------------------------------------------     0.194     0.000     0.000
------------------TGCTGCGCAGGCACGTCCGC----------------------------------------------------------------------------------------     0.777     0.038     0.038
------------------TGCTGCGCAGGCACGTCCG-----------------------------------------------------------------------------------------     0.291     0.000     0.000

--------------                       C     CC       C     A         T     T         C        C           ATC-        A   CTC
              ATCATGCTGCGCAGGCACGTCCG CGCCA  CCGCAGC CGCAC CTTGGCGAC GTCCT GCTGTGCGC TACCACGT CTACGCAAGCG    GCCATGTA TGC   G
              TGGTACGACGCGTCCGTGCAGGC GCGGT  GGCGTCG GCGTG GAGCTGTTG CAGGA CGACACGCG ATGGTGCA GATGCGTTCGC    CGGTACAT ACG   /
CGCAACGCACGGCT                       A     AA       A     C         C     -         C        C           CAAC        -   CCC

---------CGGCTTGGTACGACGCGTCCGT-----------------------------------------------------------------------------------------------     0.049     0.000     0.000
----------GGCTTGGTACGACGCGTCCGT-----------------------------------------------------------------------------------------------     0.826     0.076     0.076
-----------------TACGACGCGTCCGTGCAGGC-----------------------------------------------------------------------------------------     0.049     0.038     0.038
-----------------TACGACGCGTCCGTGCAGGCA----------------------------------------------------------------------------------------     0.049     0.152     0.301
----------------------CGCGTCCGTGCAGGCAGCGGT-----------------------------------------------------------------------------------     9.859     12.805    13.856
-------------------------GTCCGTGCAGGCAGCGGT-----------------------------------------------------------------------------------     0.049     0.000     0.000
-----------------------GCGTCCGTGCAGGCAGCGGT-----------------------------------------------------------------------------------     0.194     0.606     0.339
------------------------CGTCCGTGCAGGCAGCGGTAA---------------------------------------------------------------------------------     0.097     1.856     0.640
----------------------------CGTGCAGGCAGCGGTAAGGCG-----------------------------------------------------------------------------     0.049     0.493     0.941
-----------------------------GTGCAGGCAGCGGTAAGGCGT----------------------------------------------------------------------------     0.194     0.606     0.075
---------------------------CCGTGCAGGCAGCGGTAAGGCGT----------------------------------------------------------------------------     0.097     1.894     1.355
----------------------------CGTGCAGGCAGCGGTAAGGCGT----------------------------------------------------------------------------     5.925     105.361   95.897
-------------------------------GCAGGCAGCGGTAAGGCGTCG--------------------------------------------------------------------------     0.049     0.000     0.000
------------------------------------CAGCGGTAAGGCGTCGAGCG----------------------------------------------------------------------     0.826     0.455     1.130
-----------------------------------GCAGCGGTAAGGCGTCGAGCG----------------------------------------------------------------------     0.437     3.561     2.146
----------------------------------------GGTAAGGCGTCGAGCGT---------------------------------------------------------------------     0.097     0.568     1.242
---------------------------------------CGGTAAGGCGTCGAGCGT---------------------------------------------------------------------     0.097     2.917     14.834
------------------------------------CAGCGGTAAGGCGTCGAGCGT---------------------------------------------------------------------     85.140    164.539   227.449 miR_t67
-----------------------------------GCAGCGGTAAGGCGTCGAGCGT---------------------------------------------------------------------     45.217    557.191   212.727
-------------------------------------AGCGGTAAGGCGTCGAGCGT---------------------------------------------------------------------     3.108     25.194    15.211
----------------------------------GGCAGCGGTAAGGCGTCGAGCGT---------------------------------------------------------------------     0.049     1.326     0.301
--------------------------------------GCGGTAAGGCGTCGAGCGTGC-------------------------------------------------------------------     0.340     1.932     0.753
-------------------------------------------AAGGCGTCGAGCGTGCGAGCT--------------------------------------------------------------     0.049     0.038     0.038
---------------------------------------------GGCGTCGAGCGTGCGAGCTGT------------------------------------------------------------     0.437     1.174     0.377
-----------------------------------------------------GCGTGCGAGCTGTT-GCCAGGA---------------------------------------------------     0.097     0.265     0.265
--------------------------------------------------------TGCGAGCTGTT-GCCAGGACGA------------------------------------------------     0.097     0.076     0.076
----------------------------------------------------------------GTT-GCCAGGACGACACGCGCA----------------------------------------     0.146     0.038     0.264
------------------------------------------------------------------T-GCCAGGACGACACGCGCAT---------------------------------------     0.291     0.152     0.075
-----------------------------------------------------------------TT-GCCAGGACGACACGCGCAT---------------------------------------     0.049     0.076     0.113
----------------------------------------------------------------GTT-GCCAGGACGACACGCGCAT---------------------------------------     6.605     5.721     3.238
---------------------------------------------------------------TGTT-GCCAGGACGACACGCGCAT---------------------------------------     0.291     0.038     0.038
-------------------------------------------------------------GCTGTT-GCCAGGACGACACGCGCAT---------------------------------------     0.049     0.189     0.189
--------------------------------------------------------------------GCCAGGACGACACGCGCATG--------------------------------------     0.049     0.000     0.000
------------------------------------------------------------------------GGACGACACGCGCATGGT------------------------------------     0.049     0.000     0.000
---------------------------------------------------------------------CCAGGACGACACGCGCATGGT------------------------------------     1.360     0.114     0.075
----------------------------------------------------------------------CAGGACGACACGCGCATGGT------------------------------------     3.983     0.720     0.188
--------------------------------------------------------------------------------CGCGCATGGTGCACGATGCGT-------------------------     0.243     0.303     0.113
```

miRNA: miR_t76 chromosome_14 Start: 3218718 End: 3218895 Condition: TAP

```
-----------------------------------------------TGCGGGGCCCGCAGGTAGCTGC-------------------------------------------     34.192    1.061     2.146 miR_t76
--------------------------------------------------------------TAGCTGCCGTTGAGCCCGACG-----------------------------     0.097     0.038     0.113
----------------------------------------------------------------GCTGCCGTTGAGCCCGACGC----------------------------     0.000     0.038     0.038
-----------------------------------------------------------------------TTGAGCCCGACGCTGCTGC----------------------     0.291     0.379     0.075

-------------------------------------------                    A     C                    C    C - C         G
                                           GGGTTGCGGGGCCCGCAGGT GCTGC GTTGAGCCCGACGCTGCTGC GCCG G C GGTGCTGCT C
                                           CCCGACGCCCTGGGCGTCCA CGACG CAGCTCGGGCTGCGACGACG CGGC C G CCACGACGG C
TCGGCCAGTCCACGTCGGCCAGTCCACGTCAGCCATCGACGAC                    C     C                    T    - A T         C
---------------------------------------------------------------------CCAGCTCGGGCTGCGACGACGT-------------------- 0.146 0.076 0.301
----------------------------------------------------------------------CAGCTCGGGCTGCGACGACGT-------------------- 0.049 1.023 0.678
----------------------------------------------------------------------CAGCTCGGGCTGCGACGACG--------------------- 0.049 0.0376508192987708 0.038
-------------------------------------------------------------------CGCCAGCTCGGGCTGCGACGAC---------------------- 0.631 0.379 0.075
--------------------------------------------------------------------GCCAGCTCGGGCTGCGACGAC---------------------- 0.049 0.038 0.113
---------------------------------------------------------------------CCAGCTCGGGCTGCGACGA----------------------- 0.049 0.0753016385975416 0.075
-------------------------------------------------------------------CGCCAGCTCGGGCTGCGACGA----------------------- 30.355 54.367 39.420
--------------------------------------------------------------------GCCAGCTCGGGCTGCGACGA----------------------- 0.049 0.076 0.113
-------------------------------------------------------------------CGCCAGCTCGGGCTGCGACG------------------------ 0.437 0.796 0.565
----------CACGTCGGCCAGTCCACGTCAGCCATC-------------------------------------------------------------------------- 0 0.0376508192987708 0.038
----CCAGTCCACGTCGGCCAGTCC-------------------------------------------------------------------------------------- 0 0.0753016385975416 0.075
```

miRNA: miR_t78 chromosome_16 Start: 2231045 End: 2231429 Condition: TAP

```
----------TGCTGCGCAGGCACGTCCGTC-----------------------------------------------------------------------------------------     0.049     0.000     0.000
----------TGCTGCGCAGGCACGTCCG-------------------------------------------------------------------------------------------     0.291     0.000     0.000
----------------------------------------------------T---CGACAACGGTCCT-GCTGTGC-------------------------------------------     0.049     0.000     0.000
---------------------------------------------------------------------------------------TGCTACGCAAGCGGTTGGCCATG----------     0.097     0.000     0.000
```

```
TGCCGAAC                                             ---    AACG     -         G        G               G       TGCG  C
        CATGCTGCGCAGGCACGTCCGTCGCCATTCCGCAGCTCGCACGCT   CGAC    GTCCT GCTGTGCGC TACCACGT CTACGCAAGCGGTTG CCATGTA    GC G
        GTACGACGCGTCCGTGCAGGCGGCGGTGGGGCGTCGGGCGTGTGA   GCTG    CAGGA CGACACGCG ATGGTGCA GATGCGTTCGCTAGC GGTACAT    CG G
-----GTA                                             ACC    A---     A         G        G               -       TA--  G

----------------------------------------------------------------------------GCGGATGGTGCAGGATGCGT------------------------      0.389      0.188      0.188
---------------------------------------------------------------------------CGCGGATGGTGCAGGATGCGT------------------------     92.668     31.635     16.303 miR_t78
----------------------------------------------------------------------------GCGGATGGTGCAGGATGCG-------------------------      0.049      0.000      0.000
---------------------------------------------------------------------------CGCGGATGGTGCAGGATGCG-------------------------      0.146      0.076      0.075
-----------------------------------------------------------------------GACACGCGGATGGTGCAGGAT----------------------------      0.049      0.000      0.000
----------------------------------------------------------------------CGACACGCGGATGGTGCAGGAT----------------------------      0.049      0.000      0.000
-------------------------------------------------------------------------CACGCGGATGGTGCAGGAT----------------------------      0.437      0.038      0.038
---------------------------------------------------------------------------CGCGGATGGTGCAGGAT----------------------------      0.049      0.000      0.000
------------------------------------------------------------------------ACACGCGGATGGTGCAGGAT----------------------------      0.049      0.000      0.000
----------------------------------------------------------------CAGGAACGACACGCGGATGGT-----------------------------------      0.049      0.000      0.000
---------------------------------------------------------------ACAGGAACGACACGCGGATGGT-----------------------------------      0.194      0.189      0.189
-----------------------------GGCGGTGGGGCGTCGGGCGTGT---------------------------------------------------------------------      0.097      0.000      0.000
------------------------------GCGGTGGGGCGTCGGGCGT-----------------------------------------------------------------------      0.049      0.000      0.000
-------------------------------CGGTGGGGCGTCGGGCGT-----------------------------------------------------------------------      0.097      0.000      0.000
-----------------------GCAGGCGGCGGTGGGGCGTCGGGCGT-----------------------------------------------------------------------      0.049      0.000      0.000
----------------------------CGGCGGTGGGGCGTCGGGCGT-----------------------------------------------------------------------      6.557      0.379      0.828
-----------------------------GGCGGTGGGGCGTCGGGCGT-----------------------------------------------------------------------      0.729      0.038      0.188
---------------------------GCGGCGGTGGGGCGTCGGGCGT-----------------------------------------------------------------------      0.243      0.038      0.038
---------TACGACGCGTCCGTGCAGGC-------------------------------------------------------------------------------------------      0.049      0.038      0.038

miRNA: miR_t89 chromosome_11 Start: 2529187 End: 2529546 Condition: TAP
---------------------------------------------------------------------------------------------------------------------------------------------------------------------------TCTGGATGGTCGAAACCGCG---------------------------------------------------------------------------------      1.117      2.425      2.033
---------------------------------------------------------------------------------------------------------------------------------------------------------------------------TCTGGATGGTCGAAACCGC----------------------------------------------------------------------------------      0.437      0.644      0.113
---------------------------------------------------------------------------------------------------------------------------------------------------------------------------TCTGGATGGTCGAAACCGCGT--------------------------------------------------------------------------------      0.097      0.038      0.038
---------------------------------------------------------------------------------------------------------------------------------------------------------------------------TCTGGATGGTCGAAACCGCGTAC------------------------------------------------------------------------------      1.506      1.023      0.113
-----------------------------------------------------------------------------------------------------------------------------------------------------------------------------TGGATGGTCGAAACCGCGTAC------------------------------------------------------------------------------      6.071      4.016      2.786
-----------------------------------------------------------------------------------------------------------------------------------------------------------------------------TGGATGGTCGAAACCGCGTACA-----------------------------------------------------------------------------     77.466     21.444     14.571 miR_t89
-----------------------------------------------------------------------------------------------------------------------------------------------------------------------------TGGATGGTCGAAACCGCGTACAGC---------------------------------------------------------------------------      0.146      0.076      0.076
-----------------------------------------------------------------------------------------------------------------------------------------------------------------------------TGGATGGTCGAAACCGCGTACAG----------------------------------------------------------------------------      0.049      0.000      0.000
------------------------------------------------------------------------------------------------------------------------------------------------------------------------------GGATGGTCGAAACCGCGTAC------------------------------------------------------------------------------      0.049      0.000      0.000
------------------------------------------------------------------------------------------------------------------------------------------------------------------------------GGATGGTCGAAACCGCGTACA-----------------------------------------------------------------------------      0.534      0.076      0.076
-------------------------------------------------------------------------------------------------------------------------------------------------------------------------------GATGGTCGAAACCGCGTACA-----------------------------------------------------------------------------      0.049      0.000      0.000
-----------------------------------------------------------------------------------------------------------------------------------------------------------------------------------------------TACAGCACTCCCGCCGTCGCTC-----------------------------------------------------------      0.680      0.038      0.226
------------------------------------------------------------------------------------------------------------------------------------------------------------------------------------------------ACAGCACTCCCGCCGTCGCTC-----------------------------------------------------------      0.049      0.000      0.000
-------------------------------------------------------------------------------------------------------------------------------------------------------------------------------------------------------TCCCGCCGTCGCTCTGCGCCTT---------------------------------------------------      0.049      0.000      0.000
---------------------------------------------------------------------------------------------------------------------------------------------------------------------------------------------------------------------------TTCCTGGCGGGAACCTGGGCC--------------------------------      0.049      0.000      0.000
-------------------------------------------------------------------------------------------------------------------------------------------------------------------------------------------------------------------------------TGGCGGGAACCTGGGCCTTTG----------------------------      0.049      0.000      0.000

-----------------------------------------------------------------------------------------------------------------------------------------------------------------------                     G                    G            C GG                      GTCCCCGCCCTTCCTGGGC  GC
                                                                                                                                                                       AAGGTCTGGATGGTCGAAACC CGTACAGCACTCCCGCCGTC CTCTGCGCCTTC T  CGGGAACCTGGGCCTTTGTCAT                   CC  T
                                                                                                                                                                       TTCCAGACTTACCAGCTTTGG GCATGTCGTGAGGGCGGCAG GAGACGCGGAAG A  GCCCTTGGACCCGGAAACGGTA                   GG  A
GGCCGGGGGACAAGCGTAAACCGGTGTGCTCCCTTCTGGAGGCCAAGTGCGGTATAGCTCGGTTGCAGCGGATCCAGGCTTGGACGACCGGCAGTCATTTCATATAACACATCGACGCCGCCACCTACAACTGCGCGTGCCAGCTATAGTATAGGCACGGCTTCGAC                     -                    A            T AA                      GT-----------------  AA

-------------------------------------------------------------------------------------------------------------------------------------------------------------------------------------------------------------------AGACGCGGAAGTAAAGCCCTT----------------------------------------      0.486      0.038      0.038
----------------------------------------------------------------------------------------------------------------------------------------------------------------------------------------------------------GCGGCAGAGAGACGCGGAAGT-------------------------------------------------      0.340      0.038      0.038
----------------------------------------------------------------------------------------------------------------------------------------------------------------------------------CCAGCTTTGG-GCATGTCGTGA------------------------------------------------------------------------      0.097      0.076      0.076
---------------------------------------------------------------------------------------------------------------------------------------------------------------------------------ACCAGCTTTGG-GCATGTCGT--------------------------------------------------------------------------      0.049      0.038      0.038
--------------------------------------------------------------------------------------------------------------------------------------------------------------------------------TACCAGCTTTGG-GCATGTCGT--------------------------------------------------------------------------      0.146      0.189      0.075
---------------------------------------------------------------------------------------------------------------------------------------------------------------------------------ACCAGCTTTGG-GCATGTCG---------------------------------------------------------------------------      0.049      0.000      0.000
---------------------------------------------------------------------------------------------------------------------------------------------------------------------------AGACTTACCAGCTTTGG-GCAT-------------------------------------------------------------------------------      0.631      2.804      1.694 miR_t89*
-------------------------------------------------------------------------------------------------------------------------------------------------------------------CGACTTCCAGACTTACCAGCT----------------------------------------------------------------------------------------      0.389      0.796      0.339
----GGGGGACAAGCGTAAACCGGT-------------------------------------------------------------------------------------------------------------------------------------------------------------------------------------------------------------------------------------------------------      0.049      0.000      0.000

miRNA: miR_t60,miR_t113 chromosome_13 Start: 2001058 End: 2001210 Condition: TAP
-------TAAGTCAGCAAGCAGTCAGG---------------------------------------------------      0.049      0.265      0.377
---------AGTCAGCAAGCAGTCAGGTAG------------------------------------------------      0.049      0.341      0.452
---------AGTCAGCAAGCAGTCAGGTAGA-----------------------------------------------      0.194      2.993      0.753
-----------TCAGCAAGCAGTCAGGTAGAAGCG-------------------------------------------      0.049      0.114      0.114
-----------TCAGCAAGCAGTCAGGTAGAAG---------------------------------------------      1.991      3.902      2.974
-----------TCAGCAAGCAGTCAGGTAGA-----------------------------------------------     16.610     50.692     76.958
-----------TCAGCAAGCAGTCAGGTAG------------------------------------------------      0.146      0.303      0.038
-----------TCAGCAAGCAGTCAGGTAGAA----------------------------------------------      3.885      5.153      6.099
-------------AGCAAGCAGTCAGGTAGAAGCG-------------------------------------------      0.049      0.341      0.341
-------------AGCAAGCAGTCAGGTAGAA----------------------------------------------      0.049      0.000      0.000
--------------GCAAGCAGTCAGGTAGAAGCG-------------------------------------------      0.243      1.894      0.979
---------------CAAGCAGTCAGGTAGAAGCG-------------------------------------------      0.194      1.326      1.468
----------------AAGCAGTCAGGTAGAAGCGC------------------------------------------      0.049      0.038      0.339
----------------AAGCAGTCAGGTAGAAGCG-------------------------------------------      0.049      0.189      0.188
---------------------------TAGAAGCGCCGAGTAGGAG--------------------------------      0.049      0.000      0.000
---------------------------TAGAAGCGCCGAGTAGGAGTGAG----------------------------      0.049      0.000      0.000
---------------------------TAGAAGCGCCGAGTAGGAGTGA-----------------------------      0.049      0.000      0.000
-----------------------------------CCGAGTAGGAGTGAGAGTCAG----------------------     15.736      3.220      5.346
---------------------------------------GTAGGAGTGAGAGTCAG----------------------      0.049      0.000      0.000
----------------------------------------TAGGAGTGAGAGTCAG----------------------      0.729      0.000      0.000
----------------------------------------TAGGAGTGAGAGTCAGTGGGG-----------------      5.634      3.978      2.937
----------------------------------------TAGGAGTGAGAGTCAGTGGG------------------      0.340      1.440      2.673
----------------------------------------TAGGAGTGAGAGTCAGTGG-------------------      3.740      8.790     15.625
----------------------------------------TAGGAGTGAGAGTCAGT---------------------      0.146      0.758      0.941
----------------------------------------TAGGAGTGAGAGTCAGTGGGGT----------------      0.097      0.000      0.000
----------------------------------------TAGGAGTGAGAGTCAGTGGGGTAT--------------      0.049      0.000      0.000
----------------------------------------TAGGAGTGAGAGTCAGTG--------------------      0.146      0.076      0.113
-----------------------------------------AGGAGTGAGAGTCAGTGGGG-----------------      0.194      0.038      0.038
----------------------------------------------TGAGAGTCAGTGGGGTATG-------------      0.097      0.189      1.355
--------------------------------------------------------TGGGGTATGTAAGCGTCTTG--      0.291      0.038      0.075
--------------------------------------------------------TGGGGTATGTAAGCGTCTTGC-     13.211     46.373     54.707
--------------------------------------------------------TGGGGTATGTAAGCGTCT----      0.146      0.114      0.038
---------------------------------------------------------GGGGTATGTAAGCGTCTTGC-      0.146      0.265      0.753

---CGG T                    A                           T              T  TGC
      C AAGTCAGCAAGCAGTCAGGT GAAGCGCCGAGTAGGAGTGAGAGTCAG GGGGTATGTAAGCG CT   C
      G TTCAGTCGTTCGTCAGTCCA CTTCGCGGCTCATCCTCACTCTCAGTC CCCCATATATTCGC GA   /
ATTGTA C                    C                           T              C  TTT

-------------------------------------------------------CTCCCCATATATTCGCCGATG--      0.049      0.000      0.000
------------------------------------------------------TCTCCCCATATATTCGCCGAT---      0.097      0.075      0.075
------------------------------------------CCTCACTCTCAGTCTCCCCAT---------------      0.049      0.000      0.000
----------------------------------CGGCTCATCCTCACTCTCAGTCT---------------------      0.243      0.303      0.753
-----------------------------------GGCTCATCCTCACTCTCAGTCT---------------------      0.826      0.909      0.527
------------------------------------GCTCATCCTCACTCTCAGTCT---------------------      0.583      0.076      0.301
----------------------------------CGGCTCATCCTCACTCTCAGTC----------------------      2.623      4.887      5.535
--------------------------------CGCGGCTCATCCTCACTCTCAGTC----------------------      0.194      0.000      0.000
--------------------------------CGCGGCTCATCCTCACTCTCAGT-----------------------     24.430     13.222      1.920
-----------------------------------GGCTCATCCTCACTCTCAGT-----------------------      8.548      4.357      3.878
---------------------------------GCGGCTCATCCTCACTCTCAGT-----------------------     13.356      4.887      7.154
----------------------------------CGGCTCATCCTCACTCTCAGT-----------------------   1806.833   2599.440   3533.718 miR_t60
-------------------------------TCGCGGCTCATCCTCACTCTCAGT-----------------------      0.340      0.000      0.000
------------------------------------GCTCATCCTCACTCTCAGT-----------------------      0.097      0.076      0.113
---------------------------------GCGGCTCATCCTCACTCTCAG------------------------      0.777      0.227      0.301
--------------------------------CGCGGCTCATCCTCACTCTCAG------------------------      1.311      0.303      0.301
----------------------------------CGGCTCATCCTCACTCTCAG------------------------     27.781     31.673     72.252
----------------------------------CGGCTCATCCTCACTCTCA-------------------------      5.440      0.076      0.452
--------------------------------CGCGGCTCATCCTCACTCTCA-------------------------      0.194      0.113      0.113
---------------------------------GCGGCTCATCCTCACTCTCA-------------------------      0.049      0.114      0.038
---------------------------------GCGGCTCATCCTCACTCT---------------------------      0.194      0.000      0.000
------------------------------TTCGCGGCTCATCCTCACTCT---------------------------      0.049      0.000      0.000
--------------------------------CGCGGCTCATCCTCACTCT---------------------------      1.214      0.000      0.000
-------------------------------TCGCGGCTCATCCTCACT-----------------------------      0.049      0.000      0.000
----------------------------CCTTCGCGGCTCATCCTCACT-----------------------------      0.049      0.000      0.000
--------------------TCAGTCCACCTTCGCGGCTCAT------------------------------------      0.049      0.000      0.000
---------------------CAGTCCACCTTCGCGGCTCAT------------------------------------      0.437      0.189      0.189
----------------------AGTCCACCTTCGCGGCTCAT------------------------------------      0.243      0.038      0.038
------------------CGTCAGTCCACCTTCGCGGCT---------------------------------------      5.003      3.448      3.727
------------------CGTCAGTCCACCTTCGCGGC----------------------------------------      0.049      0.076      0.076
-----------AGTCGTTCGTCAGTCCACCTTC---------------------------------------------      0.389      0.038      0.038
------------GTCGTTCGTCAGTCCACCTTC---------------------------------------------      2.914      4.357      2.447
--------------CGTTCGTCAGTCCACCTT----------------------------------------------      0.923      3.296      2.523
----------CAGTCGTTCGTCAGTCCACCTT----------------------------------------------      0.340      0.038      0.038
-------------TCGTTCGTCAGTCCACCTT----------------------------------------------      0.340      1.440      0.452
------------GTCGTTCGTCAGTCCACCTT----------------------------------------------     30.355     10.911      6.739
---------------GTTCGTCAGTCCACCTT----------------------------------------------      0.049      0.000      0.000
-----------AGTCGTTCGTCAGTCCACCTT----------------------------------------------    601.274    109.491     38.893 miR_t113
---------TCAGTCGTTCGTCAGTCCACCTT----------------------------------------------      0.049      0.076      0.076
-----------AGTCGTTCGTCAGTCCACCT-----------------------------------------------     10.636      2.046      0.753
----------CAGTCGTTCGTCAGTCCACCT-----------------------------------------------      0.389      0.152      0.038
--------------CGTTCGTCAGTCCACCT-----------------------------------------------      0.049      0.455      0.188
------------GTCGTTCGTCAGTCCACCT-----------------------------------------------      2.088      0.871      0.904
---------TCAGTCGTTCGTCAGTCCACCT-----------------------------------------------      0.097      0.189      0.189
-----------AGTCGTTCGTCAGTCCACC------------------------------------------------      0.097      0.000      0.000
-------CTTCAGTCGTTCGTCAGTCCAC-------------------------------------------------      0.874      2.993      0.941
----------CAGTCGTTCGTCAGTCCAC-------------------------------------------------      0.049      0.000      0.000
-----------AGTCGTTCGTCAGTCCAC-------------------------------------------------      1.020      0.038      0.038
------------GTCGTTCGTCAGTCCAC-------------------------------------------------      0.049      0.038      0.038
-----------AGTCGTTCGTCAGTCC---------------------------------------------------      0.049      0.000      0.000
-----AGCTTCAGTCGTTCGTCAGT-----------------------------------------------------      0.291      0.341      0.341
----TAGCTTCAGTCGTTCGTCAGT-----------------------------------------------------      0.049      0.227      0.227

miRNA: miR_t114 chromosome_1 Start: 5106342 End: 5106482 Condition: TAP
------------GAAAAGTTGCACAGCGC-------------------------------------------      0.049      0.000      0.000
------------GAAAAGTTGCACAGCGCC------------------------------------------      0.146      0.000      0.000
-----------CGAAAAGTTGCACAGCGCC------------------------------------------      0.097      0.151      0.151
-----------CGAAAAGTTGCACAGCGCCC-----------------------------------------      0.049      0.075      0.075
----------TCGAAAAGTTGCACAGCGC-------------------------------------------      0.243      0.189      0.038
----------TCGAAAAGTTGCACAGCGCCC-----------------------------------------      5.003      1.326      0.602
----------TCGAAAAGTTGCACAGCGCC------------------------------------------      6.362      1.364      0.678
---------TTCGAAAAGTTGCACAGCGCCC-----------------------------------------      0.583      0.227      0.038
---------TTCGAAAAGTTGCACAGCGC-------------------------------------------      6.508      5.948      5.083
---------TTCGAAAAGTTGCACAGCGCC------------------------------------------     30.647     12.578      6.890 miR_t114
---------TTCGAAAAGTTGCACAGCG--------------------------------------------      0.534      1.364      1.054
--------ATTCGAAAAGTTGCACAG----------------------------------------------      0.049      0.000      0.000
--------ATTCGAAAAGTTGCACAGCGC-------------------------------------------      0.049      0.341      0.188
```

```
--------ATTCGAAAAGTTGCACAGCG--------------------------------------------      0.097      0.303      0.151
--------ATTCGAAAAGTTGCACAGCGCC------------------------------------------      0.049      0.000      0.000
-------CATTCGAAAAGTTGCACAGCG--------------------------------------------      0.049      0.000      0.000

---A              T                                       G         ACA
    TTCCATTCGAAAAG TGCACAGCGCCCCCTGCCCCCCTCCCCTTGTCCCTGTTG CCCCTGGCC   G
    AAGGTAAGCTTTTC ACGTGTCGCGGGGGACGGGGGGAGGGGAACAGGGACAAC GGGGACCGG   /
CCCA              C                                       G         GGG

-----AGGTAAGCTTTTCCACGTGT-----------------------------------------------      0.486      0.075      0.075
------GGTAAGCTTTTCCACGTGT-----------------------------------------------      0.971      0.189      0.189
----AAGGTAAGCTTTTCCACGTGT-----------------------------------------------      0.049      0.000      0.000
------GGTAAGCTTTTCCACGTGTC----------------------------------------------      0.049      0.038      0.038
-------GTAAGCTTTTCCACGTGTCGC--------------------------------------------      0.097      0.038      0.038 miR_t114*
---------AAGCTTTTCCACGTGTCGC--------------------------------------------      0.049      0.000      0.000

miRNA: miR_t120 chromosome_16 Start: 2231046 End: 2231431 Condition: TAP
-----------------------------------GGTTCCGGCAGGCGGCGGAC----------------------------      0.049      0.114      0.075
----------------------------------TGGTTCCGGCAGGCGGCGGAC----------------------------     22.876      2.614      4.895
----------------------------------TGGTTCCGGCAGGCGGCGGA-----------------------------      0.826      0.152      0.188
----------------------------------TGGTTCCGGCAGGCGGCGGACG---------------------------      5.051      1.099      0.489
----------------------------GGGAGCTGGTTCCGGCAGGCG----------------------------------      0.049      0.000      0.000
---------------------------TGGGAGCTGGTTCCGGCAGG------------------------------------      1.020      0.796      0.979
---------------------------TGGGAGCTGGTTCCGGCAGGCG----------------------------------      0.680      2.538      1.054
---------------------------TGGGAGCTGGTTCCGGCAGGCGG---------------------------------      0.097      0.493      0.493
---------------------------TGGGAGCTGGTTCCGGCAGGC-----------------------------------      3.060     19.852     23.720
------------------------CGCTGGGAGCTGGTTCCGGC---------------------------------------      0.049      0.038      0.038
------------------------CGCTGGGAGCTGGTTCCGG----------------------------------------      0.097      0.000      0.000
----------------------TCCGCTGGGAGCTGGTTCCGG----------------------------------------     20.496     15.458      2.334
----------------------TCCGCTGGGAGCTGGTTCCG-----------------------------------------      0.097      0.038      0.038
----------------------TCCGCTGGGAGCTGGTTCCGGC---------------------------------------      0.486     18.261      2.824
--------------------TGTCCGCTGGGAGCTGGTTC-------------------------------------------      0.049      0.000      0.000
--------------------TGTCCGCTGGGAGCTGGTTCCG-----------------------------------------      0.097      0.000      0.000
------------------GATGTCCGCTGGGAGCTGG----------------------------------------------      0.486      0.152      0.151
-----------------TGATGTCCGCTGGGAGC-------------------------------------------------      0.049      0.038      0.038
-----------------TGATGTCCGCTGGGAGCTGG----------------------------------------------      6.945      4.395      1.431
-----------------TGATGTCCGCTGGGAGCTGGT---------------------------------------------      0.971      0.303      0.038
----------------TTGATGTCCGCTGGGAGCTG-----------------------------------------------      0.340      0.493      0.226
----------------TTGATGTCCGCTGGGA---------------------------------------------------      0.097      0.000      0.000
----------------TTGATGTCCGCTGGGAGCTGGTT--------------------------------------------      0.049      0.076      0.076
----------------TTGATGTCCGCTGGGAGCTGGT---------------------------------------------      1.506      1.023      0.414
----------------TTGATGTCCGCTGGGAGCTGG----------------------------------------------    347.700    162.152     82.644 miR_t120
----------------TTGATGTCCGCTGGGAGC-------------------------------------------------      0.486      2.387      1.167
----------------TTGATGTCCGCTGGGAGCT------------------------------------------------      0.049      0.000      0.000
----------------TTGATGTCCGCTGGGAGCTGGTTCCG-----------------------------------------      0.049      0.000      0.000
---------------CTTGATGTCCGCTGGGAGCTGG----------------------------------------------      0.243      0.076      0.076
-------------GCCTTGATGTCCGCTGGGAGC-------------------------------------------------      0.049      0.000      0.000

CACCA    GC                           TC           G   G  --  CCAT      A  C    C
     CGCC  GCGCCTTGATGTCCGCTGGGAGCTGGT  CGGCAGGCGGC GAC GT  GC     GGACGT GG TGTG C
     GTGG  TGCGGAACTACAGGCGACCCTCGACCA  GCCGTCCGCCG CTG CG  CG     CCTGCA CC GCAC G
-----    --                           GA           G   G  AA  CAT-      -  T    G

------------------TGCGGAACTACAGGCGACCCT--------------------------------------------      0.729      2.538      0.904
-------------------GCGGAACTACAGGCGACCCT--------------------------------------------      0.146      1.137      0.527
-------------------------------GCGACCCTCGACCAGAGCCGT-------------------------------      0.049      0.000      0.000
---------------------------------------CGACCAGAGCCGTCCGCCGGCT----------------------      0.146      0.038      0.038

miRNAs identified in HS+N Only :

miRNA: miR_t1 chromosome_9 Start: 5020265 End: 5020545 Condition: HS+N
----TCAACGCTGCCGCGGAGGCGC----------------------------------------------------------------------------------------------------------------------------      0.049      0.038      0.038
-------------------------TCGGCGTGGACGGGGACGAGC-------------------------------------------------------------------------------------------------------      4.905      0.417      1.770
--------------------------CGGCGTGGACGGGGACGAGC-------------------------------------------------------------------------------------------------------      0.000      0.038      0.038
---------------------------GGCGTGGACGGGGACGAGC-------------------------------------------------------------------------------------------------------      0.049      0.038      0.038
-------------------------------TGGACGGGGACGAGCTCGGCGAC-----------------------------------------------------------------------------------------------      0.097      0.114      0.038
-------------------------------TGGACGGGGACGAGCTCGGCG-------------------------------------------------------------------------------------------------     21.710      3.220      2.108
-------------------------------TGGACGGGGACGAGCTCGGC--------------------------------------------------------------------------------------------------      2.331      0.417      0.226
-------------------------------TGGACGGGGACGAGCTCGG---------------------------------------------------------------------------------------------------      1.214      0.114      0.151
-------------------------------TGGACGGGGACGAGCTCGGCGA------------------------------------------------------------------------------------------------      0.777      0.038      0.038
--------------------------------GGACGGGGACGAGCTCGGC--------------------------------------------------------------------------------------------------      0.049      0.113      0.113
----------------------------------------------TCGGCGACCTTCTCTTTACGC----------------------------------------------------------------------------------    113.698      4.925      5.874
----------------------------------------------TCGGCGACCTTCTCTTTACG-----------------------------------------------------------------------------------      0.389      0.152      0.339
-------------------------------------------------------TTCTCTTTACGCTGTCGAAAC-------------------------------------------------------------------------     20.010      0.341      0.226
--------------------------------------------------------TCTCTTTACGCTGTCGAAACTAT----------------------------------------------------------------------      0.534      0.151      0.151
--------------------------------------------------------TCTCTTTACGCTGTCGAAACTA-----------------------------------------------------------------------      0.389      0.038      0.151
------------------------------------------------------------TTTACGCTGTCGAAACTATC---------------------------------------------------------------------      0.340      0.076      0.038
------------------------------------------------------------TTTACGCTGTCGAAACTATCG--------------------------------------------------------------------      2.137      0.341      0.264
-------------------------------------------------------------TTACGCTGTCGAAACTATCGG-------------------------------------------------------------------      7.042      0.152      0.075
--------------------------------------------------------------TACGCTGTCGAAACTATCGGA------------------------------------------------------------------     30.307      0.493      0.941
--------------------------------------------------------------TACGCTGTCGAAACTATCGG-------------------------------------------------------------------      7.042      0.227      0.452
------------------------------------------------------------------CTGTCGAAACTATCGGAGGAGC-------------------------------------------------------------      0.097      0.227      0.264
------------------------------------------------------------------CTGTCGAAACTATCGGAGGAG--------------------------------------------------------------      0.000      0.075      0.075
-------------------------------------------------------------------TGTCGAAACTATCGGAGGAG--------------------------------------------------------------      3.691      0.606      0.640
-------------------------------------------------------------------TGTCGAAACTATCGGAGGAGC-------------------------------------------------------------     93.299      9.888     32.982
--------------------------------------------------------------------GTCGAAACTATCGGAGGAGC-------------------------------------------------------------      0.534      0.038      0.151
---------------------------------------------------------------------TCGAAACTATCGGAGGAGCAGC----------------------------------------------------------     27.198      1.932      1.092
---------------------------------------------------------------------TCGAAACTATCGGAGGAGC-------------------------------------------------------------      4.468      0.189      0.226
---------------------------------------------------------------------TCGAAACTATCGGAGGAGCA------------------------------------------------------------     56.970      0.076      0.602
---------------------------------------------------------------------TCGAAACTATCGGAGGAGCAG-----------------------------------------------------------     18.553      1.099      2.749
----------------------------------------------------------------------CGAAACTATCGGAGGAGCAG-----------------------------------------------------------      0.243      0.152      0.038
-----------------------------------------------------------------------GAAACTATCGGAGGAGC-------------------------------------------------------------      0.097      0.038      0.038
------------------------------------------------------------------------AAACTATCGGAGGAGCAG-----------------------------------------------------------      0.000      0.075      0.075
----------------------------------------------------------------------------TATCGGAGGAGCAGCTCAAAT----------------------------------------------------      5.100      0.379      1.205
----------------------------------------------------------------------------TATCGGAGGAGCAGCTCAAA-----------------------------------------------------      1.214      0.189      0.038
-----------------------------------------------------------------------------ATCGGAGGAGCAGCTCAAA-----------------------------------------------------      0.049      0.075      0.075
------------------------------------------------------------------------------TCGGAGGAGCAGCTCAAATTC--------------------------------------------------      0.729      0.301      0.301
-------------------------------------------------------------------------------------------TCAAATTCTTTCCAATAGCCGC------------------------------------      1.263      0.188      0.188
----------------------------------------------------------------------------------------------AATTCTTTCCAATAGCCGCTG----------------------------------      0.097      0.038      0.038
----------------------------------------------------------------------------------------------AATTCTTTCCAATAGCCGCTGG---------------------------------      0.097      0.076      0.264
------------------------------------------------------------------------------------------------TTCTTTCCAATAGCCGCTGGC--------------------------------      0.486      0.038      0.038
------------------------------------------------------------------------------------------------TTCTTTCCAATAGCCGCTGG---------------------------------      5.682      0.076      0.188
----------------------------------------------------------------------------------------------------------TAGCCGCTGGCAATAAGGCAA----------------------      0.729      0.075      0.075
----------------------------------------------------------------------------------------------------------TAGCCGCTGGCAATAAGGCA-----------------------      0.097      0.038      0.038
--------------------------------------------------------------------------------------------------------------------CAATAAGGCAATCGTTGCGTT------------      0.146      0.113      0.113
-----------------------------------------------------------------------------------------------------------------------TAAGGCAATCGTTGCGTTTG----------      0.000      0.038      0.038

GCTTTCAACGCTGCCG                                           C            A            A       A          A    C                           T G  CC  TC
                CGGAGGCGCTCGGCGTGGACGGGGACGAGCTCGGCGACCTTCT TTTACGCTGTCG AACTATCGGAGG GCAGCTC AATTCTTTCC ATAG CGCTGGCAATAAGGCAATCGTTGCGTT G GT  TC  C
                GCCTCCGCGAGCCGCACCTGCCCCTGCTCGAGCCGCTGGAAGA AAATGCGACAGC TTGATAGCCTCC CGTCGAG TTAAGAAAGG TATC GCGACTGTTATTCCGTTAGCAACGCAA C CG  AG  /
----------------                                           A            C            C       C          C    C                           - G  TC  AA

-----------------------------------------------------------------------------------------------------AGGCTATCCGCGACTGTTATT---------------------------      6.460      0.189      0.678
------------------------------------------------------------------------------------------------------GGCTATCCGCGACTGTTATT---------------------------      1.117      0.076      0.226
----------------------------------------------------------------------------------------------------AAGGCTATCCGCGACTGTTAT----------------------------      0.389      0.113      0.113
------------------------------------------------------------------------------------------------------GGCTATCCGCGACTGTTAT----------------------------      0.291      0.188      0.188
-----------------------------------------------------------------------------------------------------AGGCTATCCGCGACTGTTAT----------------------------      0.874      0.188      0.188
--------------------------------------------------------------------------------------------------GAAAGGCTATCCGCGACTGTT------------------------------     14.959      2.046      5.761
-------------------------------------------------------------------------------------------------AGAAAGGCTATCCGCGACTGTT------------------------------      0.097      0.038      0.113
--------------------------------------------------------------------------------------------------GAAAGGCTATCCGCGACT---------------------------------      0.000      0.038      0.038
-----------------------------------------------------------------------------------------------TAAGAAAGGCTATCCGCGACT---------------------------------      1.748      0.114      0.075
--------------------------------------------------------------------------------------CGTCGAGCTTAAGAAAGGCTAT-----------------------------------------     21.661      1.819      1.130
--------------------------------------------------------------------------------------CGTCGAGCTTAAGAAAGGCTA------------------------------------------      0.389      0.114      0.038
-----------------------------------------------------------------------------------CCCCGTCGAGCTTAAGAAAGGCT-------------------------------------------      0.194      0.114      0.075
--------------------------------------------------------------------------------------CGTCGAGCTTAAGAAAGGCT-------------------------------------------      1.894      1.023      0.791
-----------------------------------------------------------------------------------CCCCGTCGAGCTTAAGAAAGGC--------------------------------------------     23.070      2.917      9.563
------------------------------------------------------------------------------------CCCGTCGAGCTTAAGAAAGGC--------------------------------------------      5.780      0.341      2.523
----------------------------------------------------------------------------------TCCCCGTCGAGCTTAAGAAAGGC--------------------------------------------      0.583      0.076      0.339
-------------------------------------------------------------------------------------CCGTCGAGCTTAAGAAAGGC--------------------------------------------      0.340      0.301      0.301
-----------------------------------------------------------------------------------CCCCGTCGAGCTTAAGAAAGG---------------------------------------------      0.486      0.151      0.151
-----------------------------------------------------------------------------------CCCCGTCGAGCTTAAGAAAG----------------------------------------------      0.243      0.152      0.113
----------------------------------------------------------------------------------TCCCCGTCGAGCTTAAGAAAG----------------------------------------------      4.711      1.743     11.596
----------------------------------------------------------------------------------TCCCCGTCGAGCTTAAGAAA-----------------------------------------------      0.000      0.113      0.113
---------------------------------------------------------------------------------CTCCCCGTCGAGCTTAAGAAA-----------------------------------------------      0.631      0.038      0.339
-------------------------------------------------------------------------------GCCTCCCCGTCGAGCTTAAGA-------------------------------------------------      0.000      0.113      0.113
------------------------------------------------------------------GACAGCCTTGATAGCCTCCCCGT------------------------------------------------------------      0.049      0.038      0.038
--------------------------------------------------------------------CAGCCTTGATAGCCTCCCCGT------------------------------------------------------------      0.049      0.038      0.038
--------------------------------------------------------------ATGCGACAGCCTTGATAGCCT------------------------------------------------------------------      4.711      0.303      0.038
---------------------------------------------------------------TGCGACAGCCTTGATAGCCT------------------------------------------------------------------      0.437      0.038      0.038
----------------------------------------------------------------GCGACAGCCTTGATAGCCT------------------------------------------------------------------      0.000      0.038      0.038
---------------------------------------------------------GAAAAATGCGACAGCCTTGAT-----------------------------------------------------------------------      1.068      0.113      0.113
------------------------------------------------------GAAGAAAAATGCGACAGCCT---------------------------------------------------------------------------      0.583      0.075      0.075
-----------------------------------------------------GGAAGAAAAATGCGACAGCCT---------------------------------------------------------------------------     15.299      0.114      0.791
---------------------------------------------GAGCCGCTGGAAGAAAAATGC-----------------------------------------------------------------------------------      1.457      0.301      0.301
---------------------------------------------GAGCCGCTGGAAGAAAAAT-------------------------------------------------------------------------------------      2.526      0.189      0.565
-----------------------------------------------GCCGCTGGAAGAAAAAT-------------------------------------------------------------------------------------      0.146      0.038      0.038
--------------------------------------------CGAGCCGCTGGAAGAAAAAT-------------------------------------------------------------------------------------    125.160      5.834     36.145
-----------------------------------------GCTCGAGCCGCTGGAAGAAAAAT-------------------------------------------------------------------------------------      0.000      0.301      0.301
-----------------------------------------GCTCGAGCCGCTGGAAGAAAAA--------------------------------------------------------------------------------------      0.777      0.227      2.937
------------------------------------------CTCGAGCCGCTGGAAGAAAA---------------------------------------------------------------------------------------      0.000      0.038      0.038
-----------------------------------------GCTCGAGCCGCTGGAAGAAAA---------------------------------------------------------------------------------------     39.049     30.498    191.906 miR_t1
-----------------------------------------GCTCGAGCCGCTGGAAGAAA----------------------------------------------------------------------------------------      1.360      1.553      7.530
----------------------------------------TGCTCGAGCCGCTGGAAGAAA----------------------------------------------------------------------------------------      0.826      0.226      0.226
---------------------------------------CTGCTCGAGCCGCTGGAAGAA-----------------------------------------------------------------------------------------      2.088      0.417      1.205
--------------------------------------CCTGCTCGAGCCGCTGGAAGAA-----------------------------------------------------------------------------------------      5.197      0.947      2.259
-----------------------------------------GCTCGAGCCGCTGGAAGAA-----------------------------------------------------------------------------------------      0.049      0.076      0.038
--------------------------------------CCTGCTCGAGCCGCTGGAAGA------------------------------------------------------------------------------------------      0.049      0.189      0.038
-----------------------------------------GCTCGAGCCGCTGGAAG-------------------------------------------------------------------------------------------      0.000      0.151      0.151
--------------------------------CCTGCCCCTGCTCGAGCCGCT------------------------------------------------------------------------------------------------      3.011      0.075      0.075
---------------------------------CTGCCCCTGCTCGAGCCGCT------------------------------------------------------------------------------------------------      0.534      0.113      0.113
-----------------------CGAGCCGCACCTGCCCCTGCT---------------------------------------------------------------------------------------------------------      8.985      1.629      3.991
------------------------GAGCCGCACCTGCCCCTGCT---------------------------------------------------------------------------------------------------------      1.457      0.114      0.151
-----------------------CGAGCCGCACCTGCCCCTGC----------------------------------------------------------------------------------------------------------      0.194      0.038      0.151
-----------------------CGAGCCGCACCTGCCCCTG-----------------------------------------------------------------------------------------------------------      0.000      0.151      0.151
---------------------CGCGAGCCGCACCTGCCCCT------------------------------------------------------------------------------------------------------------      0.194      0.038      0.113
--------------------CCGCGAGCCGCACCTGCCCCT------------------------------------------------------------------------------------------------------------      4.565      0.189      0.264
```

```
--------------------CGCGAGCCGCACCTGCCCC-------------------------------------------------------------------------------------------------------------     0.000     0.038     0.038

miRNA: miR_t16 chromosome_8 Start: 347662 End: 348000 Condition: HS+N
-------------------------------------------------------------------------------------------------------------------------------------------------------------------------------------------ACGACGAGGACTTCGAGAC-     0.000     0.038     2.297
-------------------------------------------------------------------------------------------------------------------------------------------------------------------------------------------ACGACGAGGACTTCGAG---     0.000     0.114     0.038
-------------------------------------------------------------------------------------------------------------------------------------------------------------------------------------------ACGACGAGGACTTCGAGA--     0.000     0.188     0.188
------------------------------------------------------------------------------------------------------------------------------------------------------------------------------------------TACGACGAGGACTTCGAGA--     2.186     2.463     9.450
------------------------------------------------------------------------------------------------------------------------------------------------------------------------------------------TACGACGAGGACTTCGAG---     1.020     0.720     2.673
------------------------------------------------------------------------------------------------------------------------------------------------------------------------------------------TACGACGAGGACTTCGA----     1.263     1.932     7.869
------------------------------------------------------------------------------------------------------------------------------------------------------------------------------------------TACGACGAGGACTTCGAGAC-     0.729     3.826    22.779
------------------------------------------------------------------------------------------------------------------------------------------------------------------------------------------TACGACGAGGACTTCGAGACC     2.137    17.541   293.752 miR_t16
-----------------------------------------------------------------------------------------------------------------------------------------------------------------------------------------TTACGACGAGGACTTCGA----     0.146     0.075     0.075
-----------------------------------------------------------------------------------------------------------------------------------------------------------------------------------------TTACGACGAGGACTTCGAGAC-     0.000     0.076     0.188
-----------------------------------------------------------------------------------------------------------------------------------------------------------------------------------------TTACGACGAGGACTTCGAGA--     0.097     0.075     0.075
------------------------------------------------------------------------------------------------------------------------------------------------------------------------------------TGATTACGACGAGGACTTCG-----     0.000     0.038     0.038
-----------------------------------------------------------------------------------------------------------------------------------------------------------------------------------TTGATTACGACGAGGACTTC------     0.000     0.038     0.038
-----------------------------------------------------------------------------------------------------------------------------------------------------------------------------------TTGATTACGACGAGGACTTCG-----     0.097     0.265     0.791
--------------------------------------------------------------------------------------------------------------------------------------------------------------------------------TTCTTGATTACGACGAGGACT--------     0.097     0.075     0.075
----------------------------------------------------------------------------------------------------------------------------------------------------------------------AGGACGAAGAGCTTCTTGAT---------------------     0.000     0.038     0.038
----------------------------------------------------------------------------------------------------------------------------------------------------------------------AGGACGAAGAGCTTCTTGATTA-------------------     0.000     0.264     0.264
----------------------------------------------------------------------------------------------------------------------------------------------------------------------AGGACGAAGAGCTTCTTGATT--------------------     0.194     0.796     0.452
----------------------------------------------------------------------------------------------------------------------------------------------------------------------AGGACGAAGAGCTTCTTGA----------------------     0.000     0.114     0.113
---------------------------------------------------------------------------------------------------------------------------------------------------------------------GAGGACGAAGAGCTTCTTGAT---------------------     0.049     0.379     0.188
---------------------------------------------------------------------------------------------------------------------------------------------------------------------GAGGACGAAGAGCTTCTTGA----------------------     0.000     0.075     0.075
-------------------------------------------------------------------------------------------------------------------------------------------------------TGCGCAGA-GTACCGAGGACG-----------------------------------     0.000     0.038     0.038
----------------------------------------------------------------------------------------------------------------------------------------------------TGCTGCGCAGA-GTACCGAGG--------------------------------------     0.000     0.075     0.075
--------------------------------------------------------------------------------------------------------------------------------------------TTGCCCTCTGCTGCGCAG-------------------------------------------------     0.000     0.038     0.038
---------------------------------------------------------------------------------------------------------------------------------------AGCACTTGCCCTCTGCTGCGC---------------------------------------------------     0.000     0.075     0.075
--------------------------------------------------------------------------------------------------------------------------------------TAGCACTTGCCCTCTGCTGCG----------------------------------------------------     0.000     0.151     0.151
--------------------------------------------------------------------------------------------------------------------------------------TAGCACTTGCCCTCTGCTGC-----------------------------------------------------     0.000     0.075     0.075
--------------------------------------------------------------------------------------------------------------------------------------TAGCACTTGCCCTCTGCTGCGC---------------------------------------------------     0.000     0.075     0.075
-------------------------------------------------------------------------------------------------------------------------------------TTAGCACTTGCCCTCTGCTG------------------------------------------------------     0.000     0.075     0.075
-------------------------------------------------------------------------------------------------------------------------------------TTAGCACTTGCCCTCTGCTGCGC---------------------------------------------------     0.000     0.038     0.038
-------------------------------------------------------------------------------------------------------------------------------------TTAGCACTTGCCCTCTGCTGCG----------------------------------------------------     0.000     0.113     0.113
-------------------------------------------------------------------------------------------------------------------------------------TTAGCACTTGCCCTCTGCTGC-----------------------------------------------------     0.049     0.339     0.339
----------------------------------------------------------------------------------------------------------------------------------TCCTTAGCACTTGCCCTCTGCT-------------------------------------------------------     0.000     0.038     0.113
----------------------------------------------------------------------------------------------------------------------------------TCCTTAGCACTTGCCCTCTGC--------------------------------------------------------     0.097     0.189     1.054
---------------------------------------------------------------------------------------------------------------------------------TTCCTTAGCACTTGCCCTCTGC--------------------------------------------------------     0.097     0.076     0.226
---------------------------------------------------------------------------------------------------------------------------------TTCCTTAGCACTTGCCCTCTG---------------------------------------------------------     0.000     0.038     0.038
---------------------------------------------------------------------------------------------------------------------------------TTCCTTAGCACTTGCCCTCT----------------------------------------------------------     0.049     0.301     0.301
------------------------------------------------------------------------------------------TGCTGATCCGCTACCGCTG--------------------------------------------------------------------------------------------------     0.000     0.038     0.038
---------------------------------------------------------------TCGCAAATTCTGCAATTCTCC---------------------------------------------------------------------------------------------------------------------------     0.000     0.038     0.038
-------------------------------------------TGACGCTTCCGCTTCTGG--------------------------------------------------------------------------------------------------------------------------------------------------     0.000     0.038     0.038
-----------------------------------------TCTGACGCTTCCGCTTC-----------------------------------------------------------------------------------------------------------------------------------------------------     0.000     0.038     0.038
---------------------------------------TCTCTGACGCTTCCGCTTCT----------------------------------------------------------------------------------------------------------------------------------------------------     0.049     0.640     0.640
---------------------------------------TCTCTGACGCTTCCGCTTCTGG--------------------------------------------------------------------------------------------------------------------------------------------------     0.097     0.113     0.113
---------------------------------------TCTCTGACGCTTCCGCTTC-----------------------------------------------------------------------------------------------------------------------------------------------------     0.049     0.038     0.038
-----------------------------------TGGGTCTCTGACGCTTCCGCT-------------------------------------------------------------------------------------------------------------------------------------------------------     0.097     0.038     0.678
-----------------------------------TGGGTCTCTGACGCTTCCGC--------------------------------------------------------------------------------------------------------------------------------------------------------     0.146     0.152     0.301
---------------------------------CGTGGGTCTCTGACGCTTC-----------------------------------------------------------------------------------------------------------------------------------------------------------     0.000     0.075     0.075
--------------------------------TCGTGGGTCTCTGACGCTTCCG---------------------------------------------------------------------------------------------------------------------------------------------------------     0.389     0.227     0.489
--------------------------------TCGTGGGTCTCTGACGCTTCC----------------------------------------------------------------------------------------------------------------------------------------------------------     0.049     0.152     0.565
--------------------------------TCGTGGGTCTCTGACGCTTC-----------------------------------------------------------------------------------------------------------------------------------------------------------     0.000     0.152     0.038
------------------TCTTGGGGGCCCGCTCGTGGG----------------------------------------------------------------------------------------------------------------------------------------------------------------------------     0.000     0.113     0.113
-----------------TTCTTGGGGGCCCGCTCGTGG-----------------------------------------------------------------------------------------------------------------------------------------------------------------------------     0.049     0.114     0.151
-----------------TTCTTGGGGGCCCGCTCGTGGG----------------------------------------------------------------------------------------------------------------------------------------------------------------------------     0.000     0.038     0.038
------TCATCGCTTCTTTCTTGGG------------------------------------------------------------------------------------------------------------------------------------------------------------------------------------------     0.000     0.075     0.075
------TCATCGCTTCTTTCTTGGGGG----------------------------------------------------------------------------------------------------------------------------------------------------------------------------------------     0.146     0.113     0.113
----TATCATCGCTTCTTTCTTGGG------------------------------------------------------------------------------------------------------------------------------------------------------------------------------------------     0.000     0.188     0.188

CTGGTATCATCGCTTCTTTCT            G                                A        A          GGGTGCTGATCCGCTACCGCTGCGCGTTCTTTGGACCTCCTGGTTCCTTAGCACT   CTCT   T     A-                                           AC
                     TGGGGGCCCGCTC TGGGTCTCTGACGCTTCCGCTTCTGGAATCGC AATTCTGC ATTCTCCCTC                                                      TGCC    GC GCGCAG  GTACCGAGGACGAAGAGCTTCTTGATTACGACGAGGACTTCGAG  C
                     ACCCCCGGGCGAG ACCCAGAGACTGCGAAGGCGAAGACCTTAGCG TTAAGACG TAAGAGGGAG                                                      ACGG    CG CGCGTT  CATGGCTCCTGCTTCTCGAGGAGCTAATGCTGCTCCTGAAGCTC  C
---------------------            A                                C        C          A-----------------------------------------------------   ATAC   -     GC                                           TT

----------------------------GGCGAGAACCCAGAGACT-----------------------------------------------------------------------------------------------------------------------------------------------------------------     0.000     0.038     0.038
-------------------------CCGGGCGAGAACCCAGAGACT-----------------------------------------------------------------------------------------------------------------------------------------------------------------     0.000     0.114     0.038
--------------------------CGGGCGAGAACCCAGAGACT-----------------------------------------------------------------------------------------------------------------------------------------------------------------     0.000     0.226     0.226
---------------------------------------AGAGACTGCGAAGGCGAAGA----------------------------------------------------------------------------------------------------------------------------------------------------     0.000     0.038     0.038
-----------------------------------------AGACTGCGAAGGCGAAGACCT-------------------------------------------------------------------------------------------------------------------------------------------------     0.000     0.038     0.038
------------------------------------------GACTGCGAAGGCGAAGACCTT------------------------------------------------------------------------------------------------------------------------------------------------     0.049     0.226     0.226
------------------------------------------------GAAGGCGAAGACCTTAGCGCT------------------------------------------------------------------------------------------------------------------------------------------     0.486     0.303     1.393
---------------------------------------------------GGCGAAGACCTTAGCGCTT-----------------------------------------------------------------------------------------------------------------------------------------     0.000     0.113     0.113
---------------------------------------------------------------------------GCTAAGAGGGAGA-----------------------------------------------------ACGGA-------------------------------------------------------------     0.000     0.038     0.038
-------------------------------------------------------------------------ACGCTAAGAGGGAGA-----------------------------------------------------ACGGA-------------------------------------------------------------     0.000     0.226     0.226
------------------------------------------------------------------------GACGCTAAGAGGGAGA-----------------------------------------------------ACGGA-------------------------------------------------------------     0.000     0.151     0.151
--------------------------------------------------------------------------CGCTAAGAGGGAGA-----------------------------------------------------ACGGAT------------------------------------------------------------     0.000     0.152     0.753
---------------------------------------------------------------------------GCTAAGAGGGAGA-----------------------------------------------------ACGGAT------------------------------------------------------------     0.437     0.720     3.426
------------------------------------------------------------------------GACGCTAAGAGGGAGA-----------------------------------------------------ACGGAT------------------------------------------------------------     0.049     0.265     0.602
-----------------------------------------------------------------------------TAAGAGGGAGA-----------------------------------------------------ACGGAT------------------------------------------------------------     0.000     0.038     0.038
-------------------------------------------------------------------------ACGCTAAGAGGGAGA-----------------------------------------------------ACGGAT------------------------------------------------------------     0.971     1.894    10.429
---------------------------------------------------------------------------GCTAAGAGGGAGA-----------------------------------------------------ACGGATA-----------------------------------------------------------     0.000     0.114     0.226
---------------------------------------------------------------------------GCTAAGAGGGAGA-----------------------------------------------------ACGGATAC----------------------------------------------------------     0.000     0.414     0.414
-----------------------------------------------------------------------------------------------------------------------------------------------------------GTTGCCATGGCTCCTGCTTCT-------------------------------     0.000     0.113     0.113
------------------------------------------------------------------------------------------------------------------------------------------------------------------TGGCTCCTGCTTCTCGAGGA-------------------------     0.000     0.038     0.038
-------------------------------------------------------------------------------------------------------------------------------------------------------------------GGCTCCTGCTTCTCGAGGA-------------------------     0.000     0.038     0.038
-------------------------------------------------------------------------------------------------------------------------------------------------------------------GGCTCCTGCTTCTCGAGGAG------------------------     0.000     0.038     0.038
-------------------------------------------------------------------------------------------------------------------------------------------------------------------GGCTCCTGCTTCTCGAGGAGCT----------------------     0.437     0.038     0.377
----------------------------------------------------------------------------------------------------------------------------------------------------------------------TCCTGCTTCTCGAGGAGCT----------------------     0.000     0.075     0.075
------------------------------------------------------------------------------------------------------------------------------------------------------------------------CTGCTTCTCGAGGAGCT----------------------     0.000     0.038     0.414
---------------------------------------------------------------------------------------------------------------------------------------------------------------------CTCCTGCTTCTCGAGGAGCT----------------------     0.000     0.038     0.038
-----------------------------------------------------------------------------------------------------------------------------------------------------------------------CCTGCTTCTCGAGGAGCT----------------------     0.000     0.113     0.113
--------------------------------------------------------------------------------------------------------------------------------------------------------------------GCTCCTGCTTCTCGAGGAGCT----------------------     0.340     0.114     0.640
--------------------------------------------------------------------------------------------------------------------------------------------------------------------------GCTTCTCGAGGAGCTAAT-------------------     0.000     0.038     0.038
-----------------------------------------------------------------------------------------------------------------------------------------------------------------------CCTGCTTCTCGAGGAGCTAAT-------------------     0.146     1.280     1.280
---------------------------------------------------------------------------------------------------------------------------------------------------------------------------CTTCTCGAGGAGCTAAT-------------------     0.000     0.113     0.113
--------------------------------------------------------------------------------------------------------------------------------------------------------------------------GCTTCTCGAGGAGCTAATGCT----------------     0.049     1.061     3.238
--------------------------------------------------------------------------------------------------------------------------------------------------------------------------------------GCTAATGCTGCTCCTGAAGCT----     0.000     0.038     0.038

miRNA: miR_t17 chromosome_12 Start: 2415460 End: 2415778 Condition: HS+N
----TTTGTGCTGGCGCTCCCCTGG----------------------------------------------------------------------------------------------------------------------------------------------     0.437     0.038     0.038
--------TGCTGGCGCTCCCCTGGACGGT-----------------------------------------------------------------------------------------------------------------------------------------     0.049     0.076     0.113
-----------TGGCGCTCCCCTGGACGGTGG---------------------------------------------------------------------------------------------------------------------------------------     1.166     0.909     0.828
-----------TGGCGCTCCCCTGGACGGTG----------------------------------------------------------------------------------------------------------------------------------------     0.146     0.227     0.226
-----------TGGCGCTCCCCTGGACGGTGGG--------------------------------------------------------------------------------------------------------------------------------------    30.209     1.515     2.259
------------GGCGCTCCCCTGGACGGTGGG--------------------------------------------------------------------------------------------------------------------------------------     0.583     0.113     0.113
------------GGCGCTCCCCTGGACGGTGG---------------------------------------------------------------------------------------------------------------------------------------     0.000     0.038     0.113
------------GGCGCTCCCCTGGACGGTG----------------------------------------------------------------------------------------------------------------------------------------     0.000     0.075     0.075
-----------------TCCCCTGGACGGTGGGACAGC---------------------------------------------------------------------------------------------------------------------------------     1.408     1.629     0.979
----------------------TGGACGGTGGGACAGCTGGG-----------------------------------------------------------------------------------------------------------------------------     0.049     0.265     0.226
----------------------TGGACGGTGGGACAGCTGGGG----------------------------------------------------------------------------------------------------------------------------     7.674     2.349     7.154
----------------------TGGACGGTGGGACAGCTGG------------------------------------------------------------------------------------------------------------------------------     0.049     0.076     0.038
----------------------TGGACGGTGGGACAGCTGGGGT---------------------------------------------------------------------------------------------------------------------------     1.166     0.265     0.113
----------------------TGGACGGTGGGACAGCTGGGGTT--------------------------------------------------------------------------------------------------------------------------     0.291     1.061     0.038
-----------------------------TGGGACAGCTGGGGTTTGCG----------------------------------------------------------------------------------------------------------------------     0.243     0.038     0.151
-----------------------------TGGGACAGCTGGGGTTTGCGT---------------------------------------------------------------------------------------------------------------------     0.049     0.114     0.339
----------------------------------CAGCTGGGGTTTGCGTGGTGG----------------------------------------------------------------------------------------------------------------     0.291     0.038     0.075
--------------------------------------TGGGGTTTGCGTGGTGGCTG-------------------------------------------------------------------------------------------------------------     0.049     0.189     0.113
--------------------------------------TGGGGTTTGCGTGGTGGCTGCG-----------------------------------------------------------------------------------------------------------     7.819     1.061     8.471
--------------------------------------TGGGGTTTGCGTGGTGGCTGCGA----------------------------------------------------------------------------------------------------------     0.000     0.038     0.038
--------------------------------------TGGGGTTTGCGTGGTGGCTGC------------------------------------------------------------------------------------------------------------     0.389     0.452     0.452
--------------------------------------TGGGGTTTGCGTGGTGG----------------------------------------------------------------------------------------------------------------     0.000     0.038     0.038
---------------------------------------GGGGTTTGCGTGGTGGCTGCG-----------------------------------------------------------------------------------------------------------     0.049     0.075     0.075
-------------------------------------------TTTGCGTGGTGGCTGCGAAC--------------------------------------------------------------------------------------------------------     0.000     0.038     0.038
-------------------------------------------------TGGTGGCTGCGAACACGGGGC-------------------------------------------------------------------------------------------------     1.457     3.334     2.861
-------------------------------------------------TGGTGGCTGCGAACACGGG---------------------------------------------------------------------------------------------------     0.000     0.151     0.151
-------------------------------------------------TGGTGGCTGCGAACACGGGGCCC-----------------------------------------------------------------------------------------------     0.000     0.038     0.038
-------------------------------------------------TGGTGGCTGCGAACACGGGG--------------------------------------------------------------------------------------------------     2.088     4.395     3.916
--------------------------------------------------GGTGGCTGCGAACACGGGG--------------------------------------------------------------------------------------------------     0.000     0.076     0.113
----------------------------------------------------TGGCTGCGAACACGGGGCCCCAC--------------------------------------------------------------------------------------------     0.000     0.075     0.075
----------------------------------------------------TGGCTGCGAACACGGGG----------------------------------------------------------------------------------------------------     0.000     0.038     0.038
----------------------------------------------------TGGCTGCGAACACGGGGCCCCA---------------------------------------------------------------------------------------------     0.000     0.038     0.075
--------------------------------------------------------TGCGAACACGGGGCCCCACATT-----------------------------------------------------------------------------------------     0.097     0.114     0.038
----------------------------------------------------------------------------TTTACGACTGCGTCATGCC------------------------------------------------------------------------     0.291     0.189     0.414
----------------------------------------------------------------------------TTTACGACTGCGTCATGCCA-----------------------------------------------------------------------     1.408     1.402     0.565
----------------------------------------------------------------------------TTTACGACTGCGTCATGC-------------------------------------------------------------------------     0.874     1.212     1.544
----------------------------------------------------------------------------TTTACGACTGCGTCATGCCAAT---------------------------------------------------------------------     0.971     0.303     0.038
----------------------------------------------------------------------------TTTACGACTGCGTCATGCCAA----------------------------------------------------------------------     1.748     1.061     0.527
----------------------------------------------------------------------------TTTACGACTGCGTCATG--------------------------------------------------------------------------     0.049     0.227     0.038
-----------------------------------------------------------------------------TTACGACTGCGTCATGCCAAT---------------------------------------------------------------------     0.194     0.189     0.113
------------------------------------------------------------------------------TACGACTGCGTCATGCCAATA--------------------------------------------------------------------     0.097     0.076     0.188
------------------------------------------------------------------------------------TGCGTCATGCCAATACGTCGG--------------------------------------------------------------     0.437     0.038     0.038
----------------------------------------------------------------------------------------TCATGCCAATACGTCGGCGC-----------------------------------------------------------     0.000     0.152     0.075
-------------------------------------------------------------------------------------------TGCCAATACGTCGGCGCCAGC-------------------------------------------------------     0.194     0.833     0.753
-----------------------------------------------------------------------------------------------ATACGTCGGCGCCAGCTTCA----------------------------------------------------     0.000     0.038     0.038
------------------------------------------------------------------------------------------------TACGTCGGCGCCAGCTTCACC--------------------------------------------------     9.179     2.387    39.948 miR_t17
------------------------------------------------------------------------------------------------TACGTCGGCGCCAGCTTCAC---------------------------------------------------     0.000     0.038     0.151
-------------------------------------------------------------------------------------------------ACGTCGGCGCCAGCTTCACC--------------------------------------------------     0.000     0.075     0.075
----------------------------------------------------------------------------------------------------TCGGCGCCAGCTTCACCAGTG----------------------------------------------     0.243     0.038     0.038

CGGCTTTGTGCT                     A       G     C       C             C    C  TTA  A             A                    C                G         ---     T   CTTT--   C
            GGCGCTCCCCTGGACGGTGGG CAGCTGG GTTTG GTGGTGG TGCGAACACGGGG CCCA AT   CG CTGCGTCATGCCA TACGTCGGCGCCAGCTTCAC AGTGTGGCCGCCATGG TCTCCGCCGC  GGCGT CCA      GCA T
            CCGCGAGGGGACCTGTCACCC GTCGACT CAAAC CACCACC ATGCTTGTGCCCC GGGT TA   GC GACGCAGTACGGT ATGCAGCCGCGGTCGAAGTG TCACACCGGCGGTACC AGAGGCGGTG  CCGTA GGT      CGT C
-----------A                     C       G     A       T             C    C  TAC  C             C                    A                -         CGA     -   CCTTGT   C

--------------------------------------------------------------------------------------------------------------CGAAGTGATCACACCGGCGGT------------------------------------     0.146     0.341     0.527
-------------------------------------------------------------------------------------------------------GCCGCGGTCGAAGTGATCACA-------------------------------------------     0.000     0.113     0.113
----------------------------------------------------------------------------------------------------GCAGCCGCGGTCGAAGTGAT-----------------------------------------------     0.000     0.038     0.038
---------------------------------------------------------------------------------------------------TGCAGCCGCGGTCGAAGTGAT-----------------------------------------------     0.000     0.075     0.075
-----------------------------------------------------------------------------------------------CATGCAGCCGCGGTCGAAGT----------------------------------------------------     0.340     0.227     0.339
----------------------------------------------------------------------------------------------TCATGCAGCCGCGGTCGAAGT----------------------------------------------------     6.897     2.463    21.913 miR_t17*
----------------------------------------------------------------------------------------------TCATGCAGCCGCGGTCGAAG-----------------------------------------------------     0.097     0.075     0.075
--------------------------------------------------------------------------------GCCGACGCAGTACGGTCATGC------------------------------------------------------------------     0.049     0.226     0.226
--------------------------------------------------------------------------------GCCGACGCAGTACGGTCAT--------------------------------------------------------------------     0.000     0.038     0.038
----------------------------------------------------------------------GGGTCTATACGCCGACGCAGT----------------------------------------------------------------------------     0.000     0.038     0.038
------------------------------------------------------CTATGCTTGTGCCCCGGGT----------------------------------------------------------------------------------------------     0.000     0.038     0.038
```

```
-----------------------------------------------------CCTATGCTTGTGCCCCCGGGT----------------------------------------------------------------------------------------------      0.874      0.114      0.753
-----------------------------------------------------CCTATGCTTGTGCCCCCGGG-----------------------------------------------------------------------------------------------      0.049      0.038      0.038
------------------------------------CGACTGCAAACACACCACCTAT--------------------------------------------------------------------------------------------------------------      6.945      2.387     10.015
-------------------------------------GACTGCAAACACACCACCTAT--------------------------------------------------------------------------------------------------------------      0.194      0.076      0.527
-----------------------------------TCGACTGCAAACACACCACCTAT--------------------------------------------------------------------------------------------------------------      0.000      0.038      0.038
------------------------------------CGACTGCAAACACACCACCTA---------------------------------------------------------------------------------------------------------------      0.146      0.038      0.452
----------------------------------GTCGACTGCAAACACACCACCT----------------------------------------------------------------------------------------------------------------      0.194      0.038      0.038
--------------------------GTCACCCCGTCGACTGCAAAC-------------------------------------------------------------------------------------------------------------------------      0.000      0.227      0.715
--------------------GGACCTGTCACCCCGTCGACT-------------------------------------------------------------------------------------------------------------------------------      0.049      0.038      0.075
---------------CGAGGGGACCTGTCACCCCGT------------------------------------------------------------------------------------------------------------------------------------      0.243      0.113      0.113

miRNA: miR_t19 chromosome_9 Start: 394647 End: 394808 Condition: HS+N
---------------------------------AGATATGTAGGGCGTATG---------------------------------------      0.000      0.075      0.075
--------------------------------AAGATATGTAGGGCGTATGC--------------------------------------      0.000      0.114      0.038
-------------------------------GAAGATATGTAGGGCGTATGC--------------------------------------      0.000      0.568      0.301
------------------------------AGAAGATATGTAGGGCGTATGC--------------------------------------      0.000      0.038      0.038
------------------------------AGAAGATATGTAGGGCGTATG---------------------------------------      0.000      0.265      0.188
------------------------------AGAAGATATGTAGGGCGTA-----------------------------------------      0.000      0.075      0.075
-----------------------------TAGAAGATATGTAGGGCGTAT----------------------------------------      0.049      0.189      0.640
-----------------------------TAGAAGATATGTAGGGCGTA-----------------------------------------      3.740     20.004     23.795 miR_t19
-----------------------------TAGAAGATATGTAGGGCGTATGC--------------------------------------      0.000      0.114      0.113
-----------------------------TAGAAGATATGTAGGGCGTATG---------------------------------------      0.389      3.751      2.184
-------------------------TGAGTAGAAGATATGTAGGG---------------------------------------------      0.000      0.151      0.151
------------------------ATGAGTAGAAGATATGTAGGG---------------------------------------------      0.097      0.265      0.226
--------------------TCCAATGAGTAGAAGATATG--------------------------------------------------      0.194      0.379      0.151
-----------------TAGTCCAATGAGTAGAAGATA----------------------------------------------------      0.049      0.530      0.640

-------------           A                                           CC----     CTG--   AG
             ACCCTAGTCCA TGAGTAGAAGATATGTAGGGCGTATGCCGCGTAATGCTTTCTAA       TCAG     TGG  T
             TGGGATCAGGT ACTCATCTTCTATACATCCCGCGTACGGCGCATTACGAAGGATT       AGTC     GCC  G
ACGTCCTCTCAGG           C                                           CCCCAT     ATAAA   GG

-------CTCAGGTGGGATCAGGTCACT--------------------------------------------------------------      2.914      1.288      0.828
----CCTCTCAGGTGGGATCAGGTCACT--------------------------------------------------------------      0.000      0.075      0.075
---------------GGATCAGGTCACTCATCTTCT------------------------------------------------------      0.291      0.038      0.113
----------------GATCAGGTCACTCATCTTCTAT----------------------------------------------------      0.049      0.075      0.075
----------------------GTCACTCATCTTCTATACAT------------------------------------------------      0.000      0.038      0.038
---------------------GGTCACTCATCTTCTATACAT------------------------------------------------      1.457      0.871      0.678
----------------------------CATCTTCTATACATCCCGCGT-----------------------------------------      0.437      0.151      0.151

miRNA: miR_t27 chromosome_7 Start: 1992640 End: 1993085 Condition: HS+N
----TAGTATTGAACCTGGACGTCA---------------------------------------------------------------------------------------------------------------------------------------------------------------------------------------      324.727    697.104    443.790 miR_t27
----TAGTATTGAACCTGGACGTCAA--------------------------------------------------------------------------------------------------------------------------------------------------------------------------------------      0.097      0.303      0.113
----TAGTATTGAACCTGGACGTCAAT-------------------------------------------------------------------------------------------------------------------------------------------------------------------------------------      0.243      1.174      0.301
----TAGTATTGAACCTGGACGT-----------------------------------------------------------------------------------------------------------------------------------------------------------------------------------------      0.049      0.038      0.038
----TAGTATTGAACCTGGACGTC----------------------------------------------------------------------------------------------------------------------------------------------------------------------------------------      14.716     69.710     27.824
-----AGTATTGAACCTGGACGTC----------------------------------------------------------------------------------------------------------------------------------------------------------------------------------------      0.049      0.265      0.151
-----AGTATTGAACCTGGACGTCA---------------------------------------------------------------------------------------------------------------------------------------------------------------------------------------      1.603      0.720      1.468
------GTATTGAACCTGGACGTC----------------------------------------------------------------------------------------------------------------------------------------------------------------------------------------      0.000      0.038      0.038
------GTATTGAACCTGGACGTCA---------------------------------------------------------------------------------------------------------------------------------------------------------------------------------------      0.000      0.265      0.075
-------TATTGAACCTGGACGTCAAT-------------------------------------------------------------------------------------------------------------------------------------------------------------------------------------      0.243      0.076      0.075
-------TATTGAACCTGGACGTCA---------------------------------------------------------------------------------------------------------------------------------------------------------------------------------------      0.097      0.455      0.113
-------------------------------------------------------TCACAGTGCATGTAGACCGTC------------------------------------------------------------------------------------------------------------------------------------      0.291      0.341      0.565
-------------------------------------------------------TCACAGTGCATGTAGACCGTCA-----------------------------------------------------------------------------------------------------------------------------------      0.000      0.075      0.075
--------------------------------------------------------CACAGTGCATGTAGACCGTC------------------------------------------------------------------------------------------------------------------------------------      0.437      0.076      0.188
---------------------------------------------------------ACAGTGCATGTAGACCGTC------------------------------------------------------------------------------------------------------------------------------------      0.097      0.227      0.038
-------------------------------------------------------------TGCATGTAGACCGTCACACA-------------------------------------------------------------------------------------------------------------------------------      0.243      0.303      0.113
-------------------------------------------------------------TGCATGTAGACCGTCACACAGC-----------------------------------------------------------------------------------------------------------------------------      0.583      2.425      0.377
-------------------------------------------------------------TGCATGTAGACCGTCACACAG------------------------------------------------------------------------------------------------------------------------------      0.049      0.075      0.075
-------------------------------------------------------------TGCATGTAGACCGTCACAC--------------------------------------------------------------------------------------------------------------------------------      0.000      0.038      0.038
------------------------------------------------------------------TGTAGACCGTCACACAGCTTT-------------------------------------------------------------------------------------------------------------------------      0.000      0.075      0.075
------------------------------------------------------------------TGTAGACCGTCACACAGC----------------------------------------------------------------------------------------------------------------------------      0.000      0.075      0.075
---------------------------------------------------------------------GACCGTCACACAGCTTTTTG-------------------------------------------------------------------------------------------------------------------------      0.049      0.038      0.038
-----------------------------------------------------------------------------------TTTTTGCATGATGGCCAGTCC--------------------------------------------------------------------------------------------------------      0.243      0.189      0.678
------------------------------------------------------------------------------------TTTTGCATGATGGCCAGTCC--------------------------------------------------------------------------------------------------------      0.000      0.341      0.075
-------------------------------------------------------------------------------------------TGATGGCCAGTCCGATGGG--------------------------------------------------------------------------------------------------      0.291      0.265      0.113
-------------------------------------------------------------------------------------------TGATGGCCAGTCCGATGGGTC------------------------------------------------------------------------------------------------      0.000      0.113      0.113
--------------------------------------------------------------------------------------------------------------TCAATAGTGGGCTGCCGTGAG-----------------------------------------------------------------------------------------------      0.146      0.038      0.038
---------------------------------------------------------------------------------------------------------------------------------------------------------------------------TCAGTCTCCGACCATGCCTCT----------------------------------      0.389      0.947      0.866
------------------------------------------------------------------------------------------------------------------------------------------------------------------------------TCTCCGACCATGCCTCTTGTC-------------------------------      1.311      0.455      0.489

TGCGTA                      G                A         T                                                             T                       CA                                                                    CTGG-    C  GG
      GTATTGAACCTGGACGTCAATT AAGGCAGGCGCGGTGC TGCCAGATG CACAGTGCATGTAGACCGTCACACAGCTTTTTGCATGATGGCCAGTCCGATGGGTCAATAG GGGCTGCCGTGAGTTGGGTTAAC  CAATCTATTGACCACATCACCAACAGTTTCAGTCTCCGACCATGCCTCTTGTCACGTATGCCAAGCAG     TGTC TT  T
      CATAACTTGGACCTGCAGTTAA TTCCGTCCGCGCCACG ACGGTCTAC GTGTCACGTACATCTGGCAGTGTGTCGAAAAACGTACTACCGGTCAGGCTACCCAGTTATC CCCGACGGCACTCAACCCAATTG  GTTAGATAACTGGTGTAGTGGTTGTCAGAGTCAGAGGCTGGTACGGAGAACAGTGCATACGGTTCGTC     GCAG AG  T
------                      G                C         T                                                             C                       AC                                                                    TCGGT    A  TT

------------------------------------------------------------------------------------------------------------------------------------------------------------------------------------CTGGTACGGAGAACAGTGCAT-------------------------      2.186      3.561      1.995
----------------------------------------------------------------------------------------------------------------------------------------------------------------------------------GGCTGGTACGGAGAACAGTGCAT-------------------------      0.146      0.303      0.038
--------------------------------------------------------------------------------------------------------------------------------------------------------------------------------------GGTACGGAGAACAGTGCAT-------------------------      0.000      1.212      0.904
-----------------------------------------------------------------------------------------------------------------------------------------------------------------------------------GCTGGTACGGAGAACAGTGCAT-------------------------      0.340      1.061      0.038
-------------------------------------------------------------------------------------------------------------------------------------------------------------------------------------TGGTACGGAGAACAGTGCAT-------------------------      0.194      0.303      0.151
----------------------------------------------------------------------------------------------------------------------------------------------------------------------------------GGCTGGTACGGAGAACAGTGC---------------------------      0.243      0.114      0.264
---------------------------------------------------------------------------------------------------------------------------------------------------------------------------------AGGCTGGTACGGAGAACAGT-----------------------------      2.526      2.235      2.108
-------------------------------------------------------------------------------------------------------------------------------------------------------------------------------AGAGGCTGGTACGGAGAACAGT-----------------------------      7.625      4.698      3.878
------------------------------------------------------------------------------------------------------------------------------------------------------------------------------------CTGGTACGGAGAACAGT-----------------------------      0.049      0.791      0.791
----------------------------------------------------------------------------------------------------------------------------------------------------------------------------------------GAGGCTGGTACGGAGAACAGT-----------------------------      25.935     17.049     40.663
-----------------------------------------------------------------------------------------------------------------------------------------------------------------------------------GCTGGTACGGAGAACAGT-----------------------------      0.049      0.114      0.301
----------------------------------------------------------------------------------------------------------------------------------------------------------------------------------GGCTGGTACGGAGAACAGT-----------------------------      4.614      5.531      4.706
----------------------------------------------------------------------------------------------------------------------------------------------------------------------------------------GAGGCTGGTACGGAGAACAG------------------------------      0.146      0.076      0.678
----------------------------------------------------------------------------------------------------------------------------------------------------------------------------------GGCTGGTACGGAGAACAG------------------------------      0.049      0.038      0.038
---------------------------------------------------------------------------------------------------------------------------------------------------------------------------------AGGCTGGTACGGAGAACAG------------------------------      0.049      0.151      0.151
--------------------------------------------------------------------------------------------------------------------------------------------------------------------------------GAGGCTGGTACGGAGAACA-------------------------------      0.291      0.075      0.075
----------------------------------------------------------------------------------------------------------------------------------------------------------------------------GTCAGAGGCTGGTACGGAGAA---------------------------------      0.000      0.341      0.264
----------------------------------------------------------------------------------------------------------------------------------------------------------------------------GTCAGAGGCTGGTACGGAGA----------------------------------      0.000      1.743      1.092
---------------------------------------------------------------------------------------------------------------------------------------------------------------------------AGTCAGAGGCTGGTACGGAGA----------------------------------      0.874      5.834      5.497
--------------------------------------------------------------------------------------------------------------------------------------------------------------------------GAGTCAGAGGCTGGTACGGAGA----------------------------------      0.000      0.188      0.188
------------------------------------------------------------------------------------------------------------------------------------------------------------------GGTTGTCAGAGTCAGAGGCT--------------------------------------------      0.340      0.075      0.075
-----------------------------------------------------------------------------------------------------------------------------------------------------------------TGGTTGTCAGAGTCAGAGGCT--------------------------------------------      1.166      0.076      0.226
----------------------------------------------------------------------------------------------------------------------------------------------------ATAACTGGTGTAGTGGTTGT----------------------------------------------------------      0.291      0.152      0.038
--------------------------------------------------------------------------------------------------------------------------------------------------AGATAACTGGTGTAGTGGTT------------------------------------------------------------      0.291      0.076      0.075
-------------------------------------------------------------------------------------------------------------------------------------------------TAGATAACTGGTGTAGTGGT-------------------------------------------------------------      0.097      0.076      0.038
------------------------------------------------------------------------------------------------------------------------------------------------TTAGATAACTGGTGTAGTGGT-------------------------------------------------------------      7.042      2.122      0.791
------------------------------------------------------------------------------------------------------------------------------------------------TTAGATAACTGGTGTAGTGG--------------------------------------------------------------      0.000      0.038      0.038
------------------------------------------------------------------------------------------------------------------------------------------------TTAGATAACTGGTGTAGT----------------------------------------------------------------      0.000      0.075      0.075
-----------------------------------------------------------------------------------------------------------------------CCGACGGCACTCAACCCAATT--------------------------------------------------------------------------------------      0.340      0.379      0.339
------------------------------------------------------------------------------------------------------------------------CGACGGCACTCAACCCAATT--------------------------------------------------------------------------------------      0.000      0.038      0.188
-----------------------------------------------------------------------------------------------------------------------CCGACGGCACTCAACCCAAT---------------------------------------------------------------------------------------      0.097      0.152      0.038
----------------------------------------------------------------------------------------------------------------------CCCGACGGCACTCAACCCAAT---------------------------------------------------------------------------------------      0.000      0.151      0.151
-------------------------------------------------------------------------------------------------------------CAGTTATCCCCCGACGGCACT------------------------------------------------------------------------------------------------      0.000      0.038      0.038
-------------------------------------------------------------------------------------------------GGTCAGGCTACCCAGTTAT--------------------------------------------------------------------------------------------------------------      0.340      0.682      0.527
----------------------------------------------------------------------------------------------ACCGGTCAGGCTACCCAGTTAT--------------------------------------------------------------------------------------------------------------      4.954      1.137      0.038
------------------------------------------------------------------------------------------------CGGTCAGGCTACCCAGTTAT--------------------------------------------------------------------------------------------------------------      6.022     18.185     11.333
-----------------------------------------------------------------------------------------------CCGGTCAGGCTACCCAGTTAT--------------------------------------------------------------------------------------------------------------      44.294     16.026      7.154
-----------------------------------------------------------------------------------------------CCGGTCAGGCTACCCAGTTA---------------------------------------------------------------------------------------------------------------      0.583      0.114      0.075
------------------------------------------------------------------------------------------------CGGTCAGGCTACCCAGTT----------------------------------------------------------------------------------------------------------------      0.146      0.568      0.113
-----------------------------------------------------------------------------------------------CCGGTCAGGCTACCCAGTT----------------------------------------------------------------------------------------------------------------      0.923      0.833      0.226
------------------------------------------------------------------------------------------------CGGTCAGGCTACCCAGT-----------------------------------------------------------------------------------------------------------------      0.000      0.076      0.038
-------------------------------------------------------------------------------------------ACTACCGGTCAGGCTACCCAGT-----------------------------------------------------------------------------------------------------------------      0.777      0.038      0.113
--------------------------------------------------------------------------------------------CTACCGGTCAGGCTACCCAGT-----------------------------------------------------------------------------------------------------------------      0.874      0.909      0.038
----------------------------------------------------------------------------------------ACGTACTACCGGTCAGGCTAC---------------------------------------------------------------------------------------------------------------------      0.389      1.667      1.167
---------------------------------------------------------------------------------------AACGTACTACCGGTCAGGCT-----------------------------------------------------------------------------------------------------------------------      0.340      0.493      0.452
--------------------------------------------------------------------------------------AAACGTACTACCGGTCAGGCT-----------------------------------------------------------------------------------------------------------------------      2.331      1.894      1.280
-------------------------------------------------------------------------------------AAAACGTACTACCGGTCAGGCT-----------------------------------------------------------------------------------------------------------------------      0.194      0.303      0.565
----------------------------------------------------------------------------------------ACGTACTACCGGTCAGGCT-----------------------------------------------------------------------------------------------------------------------      1.943      0.758      0.753
--------------------------------------------------------------------------------------AAACGTACTACCGGTCAGGC------------------------------------------------------------------------------------------------------------------------      0.049      0.113      0.113
-----------------------------------------------------------------------TGGCAGTGTGTCGAAAAACGTA-------------------------------------------------------------------------------------------------------------------------------------      0.000      0.076      0.038
------------------------------------------------------------------------GGCAGTGTGTCGAAAAACGTA-------------------------------------------------------------------------------------------------------------------------------------      0.049      0.909      1.092
----------------------------------------------------------------------CTGGCAGTGTGTCGAAAAACGT--------------------------------------------------------------------------------------------------------------------------------------      0.486      1.212      0.640
------------------------------------------------------------------------GGCAGTGTGTCGAAAAACGT--------------------------------------------------------------------------------------------------------------------------------------      3.448      7.274      6.702
--------------------------------------------------------------------------CAGTGTGTCGAAAAACGT--------------------------------------------------------------------------------------------------------------------------------------      0.000      0.038      0.038
-----------------------------------------------------------------------TGGCAGTGTGTCGAAAAACGT--------------------------------------------------------------------------------------------------------------------------------------      3.885      5.493      8.660
------------------------------------------------------------------------GGCAGTGTGTCGAAAAACG---------------------------------------------------------------------------------------------------------------------------------------      0.000      0.075      0.075
-----------------------------------------------------------------------TGGCAGTGTGTCGAAAAACG---------------------------------------------------------------------------------------------------------------------------------------      0.000      0.114      0.151
----------------------------------------------------------GTCACGTACATCTGGCAGTGT---------------------------------------------------------------------------------------------------------------------------------------------------      0.000      0.038      0.038
--------------------------------------------------------GTGTCACGTACATCTGGCAGT-----------------------------------------------------------------------------------------------------------------------------------------------------      0.291      0.076      0.452
----------ACTTGGACCTGCAGTTAA------------------------------------------------------------------------------------------------------------------------------------------------------------------------------------------------------      0.000      0.151      0.151

miRNA: miR_t38,miR_t116 chromosome_15 Start: 109972 End: 110410 Condition: HS+N
---------------------------TCAACAAGGTGCTCACGAACC--------------------------------------------------------------------------------------------------------------------------------------------------------------------------------------      0.000      0.075      0.075 miR_t116*
---------------------------------------TCACGAACCAGGGCAAAATGC--------------------------------------------------------------------------------------------------------------------------------------------------------------------------      247.455     35.386     91.868 miR_t38
---------------------------------------TCACGAACCAGGGCAAAATG---------------------------------------------------------------------------------------------------------------------------------------------------------------------------      6.557      1.781      4.857
----------------------------------------CACGAACCAGGGCAAAATGC--------------------------------------------------------------------------------------------------------------------------------------------------------------------------      0.243      0.303      2.861
-----------------------------------------ACGAACCAGGGCAAAATGC--------------------------------------------------------------------------------------------------------------------------------------------------------------------------      0.486      0.114      0.489
--------------------------------------------AACCAGGGCAAAATGCAGAAG---------------------------------------------------------------------------------------------------------------------------------------------------------------------      0.049      0.414      0.414
------------------------------------------------------------------------------------------TCTCGCAGCTGAAGCAGGACA-----------------------------------------------------------------------------------------------------------------------      5.100      2.425      1.242
------------------------------------------------------------------------------------------TCTCGCAGCTGAAGCAG---------------------------------------------------------------------------------------------------------------------------      0.049      0.038      0.038
------------------------------------------------------------------------------------------TCTCGCAGCTGAAGCAGG--------------------------------------------------------------------------------------------------------------------------      0.097      0.076      0.113
------------------------------------------------------------------------------------------TCTCGCAGCTGAAGCAGGA-------------------------------------------------------------------------------------------------------------------------      0.291      0.038      0.038
------------------------------------------------------------------------------------------TCTCGCAGCTGAAGCAGGACAC----------------------------------------------------------------------------------------------------------------------      0.000      0.114      0.113
------------------------------------------------------------------------------------------TCTCGCAGCTGAAGCAGGAC------------------------------------------------------------------------------------------------------------------------      2.720      2.046      1.581
--------------------------------------------------------------------------------------------TCGCAGCTGAAGCAGGACA-----------------------------------------------------------------------------------------------------------------------      0.049      0.075      0.075
--------------------------------------------------------------------------------------------------------------------------------TTGGTCGCTGTCAACCTGACG---------------------------------------------------------------------------------      16.902     12.502      6.250
--------------------------------------------------------------------------------------------------------------------------------TTGGTCGCTGTCAACCTGACGC--------------------------------------------------------------------------------      1.166      1.061      0.640
---------------------------------------------------------------------------------------------------------------------------------TGGTCGCTGTCAACCTGACG---------------------------------------------------------------------------------      15.202     12.086      7.417
---------------------------------------------------------------------------------------------------------------------------------TGGTCGCTGTCAACCTGACGC--------------------------------------------------------------------------------      14.668      6.327      2.861
---------------------------------------------------------------------------------------------------------------------------------------------------------TGGCGTCACTGGAGCTGGGAG--------------------------------------------------------      3.594      0.114      0.113
------------------------------------------------------------------------------------------------------------------------------------------------------------------TGGAGCTGGGAGCGCTGTAC------------------------------------------------      0.194      0.038      0.038
------------------------------------------------------------------------------------------------------------------------------------------------------------------TGGAGCTGGGAGCGCTGTACT-----------------------------------------------      5.828      1.743      0.828
------------------------------------------------------------------------------------------------------------------------------------------------------------------------TGGGAGCGCTGTACTTTGTGT-----------------------------------------      0.243      0.152      0.113
-----------------------------------------------------------------------------------------------------------------------------------------------------------------------------------------------------TTCAAGGTCTCGGGCGGCGG-------------      0.000      0.038      0.038

-----------------------                                                              TA                         T     C                                              A        C          G       C    TC       C               GC   GA
                       AAGCTCAACAAGGTGCTCACGAACCAGGGCAAAATGCAGAAGGACATCGGCGATATGCAGAA  ATATCTCGCAGCTGAAGCAGGACAC GCAGT AACAAGGCGTTGGTCGCTGTCAACCTGACGCTGGTGGCGTCACTGG GCTGGGAG GCTGTACTTT TGTACAC AACT  AAGGTCT GGGCGGCGGCTGAAG  TGG  T
                       TTCGAGTTGTTCCACGAGTGCTTGGTCCCGTTTTACGTCTTCCTGTAGCCGCTGTACGTCTT  TGTAGAGTGTCGACTTCGTCCTGTG CGTCA TTGTTCCGCAACCAGCGACAGTTGGACTGCGGCTACCGCAGTGACC CGACCCTC CGACATGAAG ACATGTG TTGA  TTCCAGA CCCGCCGTCGACTTC  GTC  /
TGCCGGCCAGTCAGGACCTCGCG                                                              CC                         C     C                                              A        A          G       A    GA       A               AT   GG
```

```
-----------------------------------------------------------------------------------------------------------------------------------------------------------------CGACCCTCACGACATGAA-------------------------------------------      0.000      0.076      0.075
------------------------------------------------------------------------------------------------------------------------------------------------------------TGACCACGACCCTCACGACAT---------------------------------------------      1.457      0.189      0.339
-------------------------------------------------------------------------------------------------------------------------------------------------------------GACCACGACCCTCACGACAT---------------------------------------------      0.194      0.076      0.113
----------------------------------------------------------------------------------------------------CGTCCTGTGCCGTCACTTGTT-----------------------------------------------------------------------------------------------------      2.477      0.265      0.527
---------------------------------------------------------------------------------------------------TCGTCCTGTGCCGTCACTTGT------------------------------------------------------------------------------------------------------      0.291      0.075      0.075
----------------------------------------------------------------------------------------------CGACTTCGTCCTGTGCCGT-------------------------------------------------------------------------------------------------------------      0.000      0.075      0.075
---------------------------------------------------------------------------------------------GTCGACTTCGTCCTGTGCCGT------------------------------------------------------------------------------------------------------------      0.437      0.076      0.038
----------------------------------------------------------------------------------CCTGTAGAGTGTCGACTTCGT-----------------------------------------------------------------------------------------------------------------------      0.340      0.038      0.075
--------------------------------------------------------------------------------CTTCCTGTAGAGTGTCGACTT-------------------------------------------------------------------------------------------------------------------------      0.000      0.076      0.038
--------------------------------------------------------------------------GTACGTCTTCCTGTAGAGTGT-------------------------------------------------------------------------------------------------------------------------------      1.846      0.871      0.489
---------------------------------------------GGTCCCGTTTTACGTCTTCCT----------------------------------------------------------------------------------------------------------------------------------------------------------      3.011      0.189      0.226
-----------------------------------------GCTTGGTCCCGTTTTACGTCT--------------------------------------------------------------------------------------------------------------------------------------------------------------      0.583      0.038      0.038
------------------------------------CGAGTGCTTGGTCCCGTTTTA-------------------------------------------------------------------------------------------------------------------------------------------------------------------      0.049      0.075      0.075 miR_t38*
-------------------------------------GAGTGCTTGGTCCCGTTTT--------------------------------------------------------------------------------------------------------------------------------------------------------------------      0.097      0.038      0.038
-----------------------------------ACGAGTGCTTGGTCCCGTTTT--------------------------------------------------------------------------------------------------------------------------------------------------------------------      1.748      0.114      0.188
------------------------------------CGAGTGCTTGGTCCCGTTTT--------------------------------------------------------------------------------------------------------------------------------------------------------------------      4.808      1.440      1.242
------------------------------------CGAGTGCTTGGTCCCGTTT---------------------------------------------------------------------------------------------------------------------------------------------------------------------      0.049      0.038      0.038
---------------------------------CCACGAGTGCTTGGTCCCGTT----------------------------------------------------------------------------------------------------------------------------------------------------------------------      0.243      0.265      0.151
------------------------CGAGTTGTTCCACGAGTGCTT-------------------------------------------------------------------------------------------------------------------------------------------------------------------------------      25.401      48.040      34.074 miR_t116
------------------------CGAGTTGTTCCACGAGTGCT--------------------------------------------------------------------------------------------------------------------------------------------------------------------------------      1.506      0.417      0.339
----GGCCAGTCAGGACCTCGCGT------------------------------------------------------------------------------------------------------------------------------------------------------------------------------------------------------      0.000      0.038      0.038

miRNA: miR_t122,miR_t70 chromosome_9 Start: 6518812 End: 6519092 Condition: HS+N
----------------------------------------------------------GGACCCGGCGCAGCGGCG--------------------------------------------------------------------      0.000      0.038      0.038
---------------------------------------------------------CGGACCCGGCGCAGCGG----------------------------------------------------------------------      0.000      0.075      0.075
----------------------------------------------------GGACGCGGACCCGGCGC---------------------------------------------------------------------------      0.000      0.188      0.188
----------------------------------------------------GGACGCGGACCCGGCGCAGC------------------------------------------------------------------------      0.583      0.606      0.828
----------------------------------------------------GGACGCGGACCCGGCGCAGCG-----------------------------------------------------------------------      0.146      0.114      0.075
----------------------------------------------------GGACGCGGACCCGGCGCAG-------------------------------------------------------------------------      1.700      0.985      1.393
---------------------------------------------------TGGACGCGGACCCGGCGCA--------------------------------------------------------------------------      0.437      0.227      0.301
---------------------------------------------------TGGACGCGGACCCGGCGCAGC------------------------------------------------------------------------      64.547      80.394      65.588
---------------------------------------------------TGGACGCGGACCCGGCGCAGCG-----------------------------------------------------------------------      13.356      20.080      6.627
---------------------------------------------------TGGACGCGGACCCGGCGCAGCGGCG--------------------------------------------------------------------      0.146      0.227      0.075
---------------------------------------------------TGGACGCGGACCCGGCGC---------------------------------------------------------------------------      0.437      1.856      1.355
---------------------------------------------------TGGACGCGGACCCGGCG----------------------------------------------------------------------------      0.000      0.227      0.226
---------------------------------------------------TGGACGCGGACCCGGCGCAGCGGC---------------------------------------------------------------------      0.049      3.182      0.038
---------------------------------------------------TGGACGCGGACCCGGCGCAGCGG----------------------------------------------------------------------      7.528      45.766      7.794
---------------------------------------------------TGGACGCGGACCCGGCGCAG-------------------------------------------------------------------------      124.383      68.422      72.779
--------------------------------------------------TTGGACGCGGACCCGGCGCA--------------------------------------------------------------------------      26.081      13.828      16.190
--------------------------------------------------TTGGACGCGGACCCGGC-----------------------------------------------------------------------------      2.477      10.191      16.303
--------------------------------------------------TTGGACGCGGACCCGGCGCAG-------------------------------------------------------------------------      1293.516      871.077      922.972 miR_t122
--------------------------------------------------TTGGACGCGGACCCGGCGCAGCGGC---------------------------------------------------------------------      0.049      0.303      0.113
--------------------------------------------------TTGGACGCGGACCCGGCG----------------------------------------------------------------------------      1.166      4.357      4.819
--------------------------------------------------TTGGACGCGGACCCGGCGCAGC------------------------------------------------------------------------      12.968      19.095      15.211
--------------------------------------------------TTGGACGCGGACCCGGCGCAGCG-----------------------------------------------------------------------      0.826      3.334      1.468
--------------------------------------------------TTGGACGCGGACCCGGCGCAGCGGCG--------------------------------------------------------------------      0.000      0.114      0.151
--------------------------------------------------TTGGACGCGGACCCGGCGC---------------------------------------------------------------------------      9.034      51.601      50.753
--------------------------------------------------TTGGACGCGGACCCGGCGCAGCGG----------------------------------------------------------------------      0.389      3.069      0.452
--------------------------------------------------TTGGACGCGGACCCGGCGCAGCGGCGG-------------------------------------------------------------------      0.000      0.038      0.113
-----------------------------------------------AAGTTGGACGCGGACCCGGCGCAG-------------------------------------------------------------------------      0.000      0.076      0.113
-----------------------------------------------AAGTTGGACGCGGACCCGGCG----------------------------------------------------------------------------      0.097      0.038      0.038
-----------------------------------------------AAGTTGGACGCGGACCCGGC-----------------------------------------------------------------------------      0.000      0.076      0.038
------------------------------AGGCACCGCAACGCACCAAG------------------------------------------------------------------------------------------------      0.194      0.114      0.301
-----------------------------TAGGCACCGCAACGCACCAAGT---------------------------------------------------------------------------------------------      1.846      0.341      0.113
-----------------------------TAGGCACCGCAACGCACCAAG----------------------------------------------------------------------------------------------      13.696      9.358      9.827
-------------------TAGGCCACGCTAGGCACCGCA--------------------------------------------------------------------------------------------------------      0.194      0.076      0.113
-------------------TAGGCCACGCTAGGCACCGC---------------------------------------------------------------------------------------------------------      0.194      0.758      1.581
--------TCCAAGCGATGTAGGCCACGC-------------------------------------------------------------------------------------------------------------------      0.049      0.303      0.188
-------TTCCAAGCGATGTAGGCCACG--------------------------------------------------------------------------------------------------------------------      0.097      0.303      0.301
-------TTCCAAGCGATGTAGGCCACGCT------------------------------------------------------------------------------------------------------------------      0.000      0.076      0.038
-------TTCCAAGCGATGTAGGCCACGC-------------------------------------------------------------------------------------------------------------------      0.389      7.160      4.029

--    -    A                                                           C  C                       A         G G    CCT      C  C-      A  T   T
  CAGG TTCC AGCGATGTAGGCCACGCTAGGCACCGCAACGCACCAAGTTGGACGCGGACCCGGCGCAG GG GGGAGCAGCTGCGCACAGCCCTT GAGCCTGTG G CTGA   GACGCT TT  GAAGAC TC TCC A
  GTCC AGGG TCGCTACATCCGGTGCGATCCGTGGCGTTGCGTGGTTCAACCTGCGCCTGGGCCGCGTC TC TCCTCGTCGACGCGTGTCGGGAA CTCGGACAT C GACT   TTGTGG AG  CTTTTG AG AGG /
TG    C    C                                                           T  C                       A         - G    T--      C  TA      G  C   -

-----CCAGGGCTCGCTACATCCGGT----------------------------------------------------------------------------------------------------------------------      0.923      0.909      0.038
----CCCAGGGCTCGCTACATCCGGT----------------------------------------------------------------------------------------------------------------------      1.214      0.265      0.038
------CAGGGCTCGCTACATCCGGT----------------------------------------------------------------------------------------------------------------------      0.097      0.038      0.038
---------------------CCGGTGCGATCCGTGGCGTT-------------------------------------------------------------------------------------------------------      0.049      0.075      0.075
----------------------CGGTGCGATCCGTGGCGTT-------------------------------------------------------------------------------------------------------      0.049      0.152      0.113
---------------------------CGATCCGTGGCGTTGCGT---------------------------------------------------------------------------------------------------      0.049      0.038      0.038
---------------------------CGATCCGTGGCGTTGCGTGGT------------------------------------------------------------------------------------------------      1.214      0.114      0.264
----------------------------GATCCGTGGCGTTGCGTGGT------------------------------------------------------------------------------------------------      0.680      0.076      0.038
-------------------------------CCGTGGCGTTGCGTGGTT-----------------------------------------------------------------------------------------------      0.000      0.038      0.038
-------------------------------------------------------------GGGCCGCGTCTTCCTCCTCGT--------------------------------------------------------------      2.623      0.682      1.130
--------------------------------------------------------------GGCCGCGTCTTCCTCCTCGT--------------------------------------------------------------      0.097      0.075      0.075
---------------------------------------------------------------------TCTTCCTCCTCGTCGACG---------------------------------------------------------      0.000      0.076      0.038
---------------------------------------------------------------------TCTTCCTCCTCGTCGACGC--------------------------------------------------------      0.000      0.038      0.038
---------------------------------------------------------------------TCTTCCTCCTCGTCGACGCG-------------------------------------------------------      0.583      0.530      0.791
--------------------------------------------------------------------GTCTTCCTCCTCGTCGACGCGT------------------------------------------------------      2.234      3.334      3.313
----------------------------------------------------------------------CTTCCTCCTCGTCGACGCGT------------------------------------------------------      6.557      1.553      4.104
---------------------------------------------------------------------TCTTCCTCCTCGTCGACGCGT------------------------------------------------------      96.554      69.066      106.853 miR_t70
-----------------------------------------------------------------------TTCCTCCTCGTCGACGCGT------------------------------------------------------      0.097      0.076      0.075
----------------------------------------------------------------------CTTCCTCCTCGTCGACGCGTGT----------------------------------------------------      0.534      0.227      0.301
---------------------------------------------------------------------TCTTCCTCCTCGTCGACGCGTGT----------------------------------------------------      0.146      0.379      0.640
-------------------------------------------------------------------------------CGTCGACGCGTGTCGGGAAAC--------------------------------------------      0.291      0.265      0.226
----------------------------------------------------------------------------------CGACGCGTGTCGGGAAACT-------------------------------------------      0.049      1.137      0.602
-------------------------------------------------------------------------------CGTCGACGCGTGTCGGGAAACT-------------------------------------------      0.194      0.493      0.113
-----------------------------------------------------------------------------------------TGTCGGGAAACTCGGACAT-CGG--------------------------------      0.000      0.114      0.038
------------------------------------------------------------------------------------------GTCGGGAAACTCGGACAT-CGGA-------------------------------      0.097      0.114      0.226
-----------------------------------------------------------------------------------------TGTCGGGAAACTCGGACAT-CGGA-------------------------------      0.000      0.303      0.301

miRNA: miR_t75 chromosome_7 Start: 5910827 End: 5911129 Condition: HS+N
------------------------------------------------------------------------------------------------------------------------------------------------------------------------------------TGCGCCGCTAGCCGGGACCAG-----------------------------------      0.049      0.038      0.038
-------------------------------------------------------------------------------------------------------------------TCCCGGCTAGCGGCGCACTAC------------------------------------------------------------------------------------------------------      0.243      0.114      0.188
----------------------------------------------------------------------------------------------------------------TGGTCCCGGCTAGCGGCGC-----------------------------------------------------------------------------------------------------------      0.000      0.038      0.038
----------------------------------------------------------------------------------------------------------------TGGTCCCGGCTAGCGGCGCAC---------------------------------------------------------------------------------------------------------      0.000      0.038      0.038
----------------------------------------------------------------------------------------------------------------TGGTCCCGGCTAGCGGCGCACT--------------------------------------------------------------------------------------------------------      0.486      0.189      0.188
------------------------------------------------TCCAAGTCCAGCCCGCAGCAG-----------------------------------------------------------------------------------------------------------------------------------------------------------------------      0.097      0.038      0.038
----------------------------------------------TGTCCAAGTCCAGCCCGCAGC-------------------------------------------------------------------------------------------------------------------------------------------------------------------------      0.097      0.038      0.188
------------------------------------TGGCCGCTCTGTCCAAGTCC------------------------------------------------------------------------------------------------------------------------------------------------------------------------------------      0.049      0.038      0.038
---------------------------------TGGTGGCCGCTCTGTCCAAG---------------------------------------------------------------------------------------------------------------------------------------------------------------------------------------      1.894      1.591      0.941
---------------------------------TGGTGGCCGCTCTGTCCAAGTC-------------------------------------------------------------------------------------------------------------------------------------------------------------------------------------      0.000      0.075      0.075
--------------------------------TTGGTGGCCGCTCTGTCCAAG---------------------------------------------------------------------------------------------------------------------------------------------------------------------------------------      0.291      0.114      0.113
-------------------------------TTTGGTGGCCGCTCTGTCC------------------------------------------------------------------------------------------------------------------------------------------------------------------------------------------      0.437      0.076      0.339
-----------------------------GATTTGGTGGCCGCTCTGTCC------------------------------------------------------------------------------------------------------------------------------------------------------------------------------------------      0.000      0.038      0.038
---------------------------TGGATTTGGTGGCCGCTCTG---------------------------------------------------------------------------------------------------------------------------------------------------------------------------------------------      0.680      0.379      0.151
---------------------------TGGATTTGGTGGCCGCTCTGTC-------------------------------------------------------------------------------------------------------------------------------------------------------------------------------------------      0.000      0.075      0.075
---------------------------TGGATTTGGTGGCCGCTCTGT--------------------------------------------------------------------------------------------------------------------------------------------------------------------------------------------      0.097      0.076      0.038
-------------------------CGTGGATTTGGTGGCCGCTCT----------------------------------------------------------------------------------------------------------------------------------------------------------------------------------------------      0.000      0.038      0.038
-----------------------TACGTGGATTTGGTGGCC---------------------------------------------------------------------------------------------------------------------------------------------------------------------------------------------------      0.097      0.152      0.151
-----------------------TACGTGGATTTGGTGGCCG--------------------------------------------------------------------------------------------------------------------------------------------------------------------------------------------------      0.097      0.038      0.038
-----------------------TACGTGGATTTGGTGGCCGC-------------------------------------------------------------------------------------------------------------------------------------------------------------------------------------------------      0.049      0.114      0.038
--------------------GGCTACGTGGATTTGGTGGC----------------------------------------------------------------------------------------------------------------------------------------------------------------------------------------------------      0.146      0.568      0.113
-------------------TGGCTACGTGGATTTGGTG------------------------------------------------------------------------------------------------------------------------------------------------------------------------------------------------------      0.000      0.075      0.075
-------------------TGGCTACGTGGATTTGGTGGC----------------------------------------------------------------------------------------------------------------------------------------------------------------------------------------------------      8.694      14.851      6.401
-------------------TGGCTACGTGGATTTGGTGG-----------------------------------------------------------------------------------------------------------------------------------------------------------------------------------------------------      0.000      0.189      0.188

---------------          C                 T      A                 TG   A-   GACACGACCCCCGCGGCGGGTGCAGCCAGCTGCTGGTCCCGGCTAGCGGCGCACTACGGCGCTCATGCCGTTCCCCCGCTGTCCAGGATGAAGCGCCGCAGTGCGCCGCTAGCCGGGACCAGCAGCTGGCTGCACCTGCGTGGGGCCCATGTA  G
               CGGGTGGCTA GTGGATTTGGTGGCCGC CTGTCC AGTCCAGCCCGCAGCAG  GCG  GGC                                                                                                                                                         GC T
               GCCCACCGAT CACCTAAACCACCGGCG GACAGG TCAGGTCGGGCGTCGTC  CGT  CCG                                                                                                                                                         CG T
GGGGGGAGTGTGTGC          A                 C      C                 GT   CA   A------------------------------------------------------------------------------------------------------------------------------------------------------  T

----GGAGTGTGTGCGCCCACCGAT-----------------------------------------------------------------------------------------------------------------------------------------------------------------------------------------------------------------      0.000      0.038      0.038
------------------------------TAAACCACCGGCGCGACAGGCT------------------------------------------------------------------------------------------------------------------------------------------------------------------------------------------      0.000      0.038      0.075
-------------------------------AAACCACCGGCGCGACAGGCT------------------------------------------------------------------------------------------------------------------------------------------------------------------------------------------      0.777      0.379      0.452
--------------------------------------CGGCGCGACAGGCTCAGGT-------------------------------------------------------------------------------------------------------------------------------------------------------------------------------------      0.097      0.152      0.301
------------------------------------ACCGGCGCGACAGGCTCAGGT-------------------------------------------------------------------------------------------------------------------------------------------------------------------------------------      0.000      0.075      0.075
---------------------------------------GGCGCGACAGGCTCAGGT-------------------------------------------------------------------------------------------------------------------------------------------------------------------------------------      0.097      0.038      0.226
-------------------------------------------CGACAGGCTCAGGTCGGGCG-------------------------------------------------------------------------------------------------------------------------------------------------------------------------------      0.000      0.038      0.151
--------------------------------------------GACAGGCTCAGGTCGGGCGT------------------------------------------------------------------------------------------------------------------------------------------------------------------------------      0.097      0.038      0.038
------------------------------------------GCGACAGGCTCAGGTCGGGCGT------------------------------------------------------------------------------------------------------------------------------------------------------------------------------      0.000      0.075      0.075
-------------------------------------------CGACAGGCTCAGGTCGGGCGT------------------------------------------------------------------------------------------------------------------------------------------------------------------------------      5.682      19.663      27.673 miR_t75
-------------------------------------------CGACAGGCTCAGGTCGGGCGTC-----------------------------------------------------------------------------------------------------------------------------------------------------------------------------      0.000      0.038      0.038

miRNA: miR_t83 chromosome_6 Start: 8612418 End: 8612840 Condition: HS+N
----TCAGAACTTTGGTTTGCGTCC---------------------------------------------------------------------------------------------------------------------------------------------------------------------------------------------------      0.680      0.530      1.995
-----CAGAACTTTGGTTTGCGTCC---------------------------------------------------------------------------------------------------------------------------------------------------------------------------------------------------      0.000      0.075      0.075
------AGAACTTTGGTTTGCGTCCTG-------------------------------------------------------------------------------------------------------------------------------------------------------------------------------------------------      0.000      0.075      0.075
------------TTGGTTTGCGTCCTGCATG---------------------------------------------------------------------------------------------------------------------------------------------------------------------------------------------      0.000      0.038      0.038
------------TTGGTTTGCGTCCTGCATGAGC------------------------------------------------------------------------------------------------------------------------------------------------------------------------------------------      4.323      1.515      0.715
------------TTGGTTTGCGTCCTGCATGAG-------------------------------------------------------------------------------------------------------------------------------------------------------------------------------------------      0.631      0.303      0.414
-------------TGGTTTGCGTCCTGCATGAGC------------------------------------------------------------------------------------------------------------------------------------------------------------------------------------------      0.826      0.796      0.715
-------------TGGTTTGCGTCCTGCATGAGCC-----------------------------------------------------------------------------------------------------------------------------------------------------------------------------------------      0.000      0.038      0.038
----------------TTTGCGTCCTGCATGAGCCGC---------------------------------------------------------------------------------------------------------------------------------------------------------------------------------------      1.117      0.341      0.264
----------------------TCCTGCATGAGCCGCTTTCG----------------------------------------------------------------------------------------------------------------------------------------------------------------------------------      0.097      0.038      0.038
----------------------TCCTGCATGAGCCGCTTTCGC---------------------------------------------------------------------------------------------------------------------------------------------------------------------------------      0.049      0.114      0.226
-------------------------TGCATGAGCCGCTTTCGCGCT------------------------------------------------------------------------------------------------------------------------------------------------------------------------------      0.146      0.152      0.038
-----------------------------------------------------TCTCCGCTACGGTCTCCTGG---------------------------------------------------------------------------------------------------------------------------------------------------------      4.080      1.932      3.050
-----------------------------------------------------TCTCCGCTACGGTCTCCTGGC--------------------------------------------------------------------------------------------------------------------------------------------------------      6.945      2.122      3.727
------------------------------------------------------CTCCGCTACGGTCTCCTGGC--------------------------------------------------------------------------------------------------------------------------------------------------------      0.000      0.038      0.038
-------------------------------------------------------TCCGCTACGGTCTCCTGGC--------------------------------------------------------------------------------------------------------------------------------------------------------      0.049      0.038      0.038
------------------------------------------------------------TACGGTCTCCTGGCTTTCCAC-------------------------------------------------------------------------------------------------------------------------------------------------      0.194      0.075      0.075
------------------------------------------------------------TACGGTCTCCTGGCTTTCCACG------------------------------------------------------------------------------------------------------------------------------------------------      1.797      0.417      1.017
-------------------------------------------------------------------TCCTGGCTTTCCACGAAGCGC------------------------------------------------------------------------------------------------------------------------------------------      2.428      0.188      0.188
---------------------------------------------------------------------CTGGCTTTCCACGAAGCGCTTGG--------------------------------------------------------------------------------------------------------------------------------------      0.000      0.038      0.038
----------------------------------------------------------------------TGGCTTTCCACGAAGCGCTTGG--------------------------------------------------------------------------------------------------------------------------------------      1.506      0.530      0.264
----------------------------------------------------------------------------TCCACGAAGCGCTTGGTGACC---------------------------------------------------------------------------------------------------------------------------------      0.000      0.113      0.113
---------------------------------------------------------------------------------------CTTGGTGACCTCTCGCTTCGGG---------------------------------------------------------------------------------------------------------------------      0.049      0.075      0.075
----------------------------------------------------------------------------------------TTGGTGACCTCTCGCTTCGGGG--------------------------------------------------------------------------------------------------------------------      3.691      0.038      0.226
----------------------------------------------------------------------------------------TTGGTGACCTCTCGCTTCGGG---------------------------------------------------------------------------------------------------------------------      52.308      8.297      21.122
-----------------------------------------------------------------------------------------TGGTGACCTCTCGCTTCGGG---------------------------------------------------------------------------------------------------------------------      2.477      1.137      1.318
-----------------------------------------------------------------------------------------TGGTGACCTCTCGCTTCGGGGC-------------------------------------------------------------------------------------------------------------------      5.780      3.675      1.318
-----------------------------------------------------------------------------------------TGGTGACCTCTCGCTTCGGGG--------------------------------------------------------------------------------------------------------------------      30.258      2.311      2.899
```

```
-------------------------------------------------------------------------------------------------TCTCGCTTCGGGGCAACCTTG--------------------------------------------------------------------------------------------------------------      0.291      0.038      0.075
-------------------------------------------------------------------------------------------------TCTCGCTTCGGGGCAACCTTGC-------------------------------------------------------------------------------------------------------------      0.049      0.152      0.151
---------------------------------------------------------------------------------------------------TCGCTTCGGGGCAACCTTGC-------------------------------------------------------------------------------------------------------------      0.146      0.114      0.113
--------------------------------------------------------------------------------------------------------TTCGGGGCAACCTTGCGGCAGG------------------------------------------------------------------------------------------------------      1.360      0.644      1.355
--------------------------------------------------------------------------------------------------------TTCGGGGCAACCTTGCGGCAG-------------------------------------------------------------------------------------------------------      0.826      0.644      1.920
---------------------------------------------------------------------------------------------------------TCGGGGCAACCTTGCGGCAG-------------------------------------------------------------------------------------------------------      0.097      0.038      0.038
---------------------------------------------------------------------------------------------------------TCGGGGCAACCTTGCGGCAGG------------------------------------------------------------------------------------------------------      0.874      0.152      0.678
-------------------------------------------------------------------------------------------------------------------TTGCGGCAGGGCACCACGCTGG-------------------------------------------------------------------------------------------      0.729      0.076      0.075
--------------------------------------------------------------------------------------------------------------------TGCGGCAGGGCACCACGCTGG-------------------------------------------------------------------------------------------      0.194      0.076      0.075

AGCTTC                                                                                                                                                  -----        ACTTATT    --     ACT  A-  A  A--        GAA AA  GTAAG T      TTC
      AGAACTTTGGTTTGCGTCCTGCATGAGCCGCTTTCGCGCTGCCGCCGTCTCCGCTACGGTCTCCTGGCTTTCCACGAAGCGCTTGGTGACCTCTCGCTTCGGGGCAACCTTGCGGCAGGGCACCACGCTGGTGGCGGGAGC      TCAGTGAA       AGTG  GACGC   GC  AG GA   CCTGTTTG   C  CT     G CCCTGC   T
      TCTTGAAACCAAACGCAGGACGTACTCGGCGAAAGCGCGACGGCGGCAGAGGCGATGCCAGAGGACCGAAAGGTGCTTCGCGAACCACTGGAGAGCGAAGCCCCGTTGGAACGCCGTCCCGTGGTGCGACCACCGCCCTCG      AGTCACTT       TCAC  CTGCG   CG  TC CT   GGACAAAC   G  GA     C GGGGCG   T
------                                                                                                                                                  CGCCG        -------    TT     ---  CC  C  GAC        --- GA  AGAAA -      TTT

-----------------------------------------------------------------------------------------------------CGAAGCCCCGTTGGAACGCCGT---------------------------------------------------------------------------------------------------------      0.000      0.075      0.075
-------------------------------------------------------------------------------------GCGAACCACTGGAGAGCGAAG--------------------------------------------------------------------------------------------------------------------------      0.000      0.038      0.038
-------------------------------------------------------------------------GAAAGGTGCTTCGCGAACCACT-------------------------------------------------------------------------------------------------------------------------------------      0.049      0.038      0.038
-----------------------------------------------------------------------------GGTGCTTCGCGAACCACT-------------------------------------------------------------------------------------------------------------------------------------      0.000      0.038      0.038
--------------------------------------------------------------------------AAAGGTGCTTCGCGAACCACT-------------------------------------------------------------------------------------------------------------------------------------      0.000      0.151      0.151
--------------------------------------------------------------GCCAGAGGACCGAAAGGTGCTT------------------------------------------------------------------------------------------------------------------------------------------------      0.097      0.113      0.113
----------------------------------------------------------------CAGAGGACCGAAAGGTGCTT------------------------------------------------------------------------------------------------------------------------------------------------      0.097      0.189      0.038
---------------------------------------------------------------CCAGAGGACCGAAAGGTGCTT------------------------------------------------------------------------------------------------------------------------------------------------      0.049      0.493      0.339
------------------------------------------------------------GATGCCAGAGGACCGAAAGGTGCT------------------------------------------------------------------------------------------------------------------------------------------------      0.000      0.038      0.038
--------------------------------------------------------------GCCAGAGGACCGAAAGGTGCT-------------------------------------------------------------------------------------------------------------------------------------------------      5.051      1.819      1.242
---------------------------------------------------------------CCAGAGGACCGAAAGGTGCT-------------------------------------------------------------------------------------------------------------------------------------------------      0.000      0.113      0.113
-------------------------------------------------------------TGCCAGAGGACCGAAAGGTGCT-------------------------------------------------------------------------------------------------------------------------------------------------      0.923      0.644      0.828
--------------------------------------------------------------GCCAGAGGACCGAAAGGT----------------------------------------------------------------------------------------------------------------------------------------------------      0.000      0.151      0.151
---------------------------------------------------------------CCAGAGGACCGAAAGGT----------------------------------------------------------------------------------------------------------------------------------------------------      0.000      0.114      0.264
------------------------------------------------------------ATGCCAGAGGACCGAAAGGT----------------------------------------------------------------------------------------------------------------------------------------------------      0.486      0.114      0.113
----------------------------------------------------------CGATGCCAGAGGACCGAAAGGT----------------------------------------------------------------------------------------------------------------------------------------------------      0.389      0.303      0.301
-------------------------------------------------------------TGCCAGAGGACCGAAAGGT----------------------------------------------------------------------------------------------------------------------------------------------------      0.097      0.076      0.264
-----------------------------------------------------------GATGCCAGAGGACCGAAAGGT----------------------------------------------------------------------------------------------------------------------------------------------------      9.908      10.040     24.473 miR_t83
-----------------------------------------------------------GATGCCAGAGGACCGAAAGG-----------------------------------------------------------------------------------------------------------------------------------------------------      0.097      0.076      0.527
--------------------------------------------------GGCAGAGGCGATGCCAGAGGA-------------------------------------------------------------------------------------------------------------------------------------------------------------      0.000      0.152      0.264
-----------------------------------------CGCGACGGCGGCAGAGGCGAT----------------------------------------------------------------------------------------------------------------------------------------------------------------------      0.000      0.038      0.038
-----------AAACCAAACGCAGGACGTACT----------------------------------------------------------------------------------------------------------------------------------------------------------------------------------------------------      0.049      0.151      0.151
----------GAAACCAAACGCAGGACGTACT----------------------------------------------------------------------------------------------------------------------------------------------------------------------------------------------------      0.777      0.227      0.339
```

miRNA: miR_t86 chromosome_11 Start: 2856066 End: 2856438 Condition: HS+N

```
-----------------------------------------TCCATCCATCGTGCGCGCCCG-----------      0.049      0.038      0.038
---------------------------------------TCTCCATCCATCGTGCGCGCCCG-----------      0.000      0.038      0.038
-----------------------------------ACAGTCTCCATCCATCGTG-------------------      0.000      0.038      0.038
----------------------------------TACAGTCTCCATCCATCGTGC------------------      0.389      0.038      0.038
----------------------------------TACAGTCTCCATCCATCGTG-------------------      0.000      0.188      0.188
------------------TACTGCTGGCCTCTCGTACAG----------------------------------      2.963      0.152      0.151
--------------TACTTACTGCTGGCCTCTCG---------------------------------------      1.360      0.493      0.414
--------------TACTTACTGCTGGCCTCTCGT--------------------------------------      0.097      0.038      0.113
-----------TGTTACTTACTGCTGGCCTCT-----------------------------------------      0.194      0.038      0.038
---------TCTGTTACTTACTGCTGGC---------------------------------------------      1.117      0.038      0.038
---------TCTGTTACTTACTGCTGGCCTCT-----------------------------------------      0.097      0.113      0.113
---------TCTGTTACTTACTGCTGGCCTC------------------------------------------      0.291      0.038      0.151
-----TGCCTCTGTTACTTACTGCTG-----------------------------------------------      1.797      0.227      1.205

-                                  A                      CC   G G TT  T
 GCCCTGCCTCTGTTACTTACTGCTGGCCTCTCGT CAGTCTCCATCCATCGTGCGCG  CGT T T  AG T
 CGGGACGGAGACAATGAATGACGACCGGAGAGCA GTCAGAGGTAGGTAGCACGCGT  GCA A G  TC A
G                                  A                      A-   G G T-  A

--GGGACGGAGACAATGAATGAC--------------------------------------------------      0.000      0.075      0.075
----------------GAATGACGACCGGAGAGCAAGT-----------------------------------      4.760      0.189      0.414
-------------------------CCGGAGAGCAAGTCAGAGG-----------------------------      0.000      0.113      0.113
--------------------------CGGAGAGCAAGTCAGAGG-----------------------------      0.049      0.188      0.188
-------------------------CCGGAGAGCAAGTCAGAGGT----------------------------      0.874      0.227      0.941
------------------------ACCGGAGAGCAAGTCAGAGGT----------------------------      1.700      0.227      1.318
--------------------------CGGAGAGCAAGTCAGAGGT----------------------------      14.813     8.486      26.243 miR_t86
```

miRNA: miR_t102 chromosome_9 Start: 6560167 End: 6560545 Condition: HS+N

```
--------------------------------------------------------------TATTGACTGTACCTACCATCA-----------------------------------------------------------------------------------------------------------------------------------------      0.729      0.152      0.075
----------------------------------------------------------------TTGACTGTACCTACCATCATG---------------------------------------------------------------------------------------------------------------------------------------      0.146      0.038      0.038
-----------------------------------------------------------------TGACTGTACCTACCATCATGG--------------------------------------------------------------------------------------------------------------------------------------      0.000      0.189      0.038
-------------------------------------------------------------------------------------------TTTGAAAGTCGAAGGCCCGAC------------------------------------------------------------------------------------------------------------      1.651      0.417      0.640
--------------------------------------------------------------------------------------------TTGAAAGTCGAAGGCCCGACAG----------------------------------------------------------------------------------------------------------      0.097      0.038      0.038
--------------------------------------------------------------------------------------------TTGAAAGTCGAAGGCCCGACAGC---------------------------------------------------------------------------------------------------------      0.049      0.152      0.151
---------------------------------------------------------------------------------------------TGAAAGTCGAAGGCCCGACAGC---------------------------------------------------------------------------------------------------------      0.146      0.227      0.038
---------------------------------------------------------------------------------------------TGAAAGTCGAAGGCCCGACAG----------------------------------------------------------------------------------------------------------      0.243      0.113      0.113
----------------------------------------------------------------------------------------------------------------------------------TCGCCGCGGGCCTTCAAGC-----------------------------------------------------------------------      0.000      0.113      0.113
----------------------------------------------------------------------------------------------------------------------------------TCGCCGCGGGCCTTCAAGCTC---------------------------------------------------------------------      0.146      0.341      0.151
-------------------------------------------------------------------------------------------------------------------------------------------------------------------------------C-ACTGGCCTGCATCTCCTCTG-----------------------      0.000      0.075      0.075
---------------------------------------------------------------------------------------------------------------------------------------------------------------------------------ACTGGCCTGCATCTCCTCTGC----------------------      0.049      0.075      0.075
-----------------------------------------------------------------------------------------------------------------------------------------------------------------------------------TGGCCTGCATCTCCTCTGC----------------------      0.243      0.227      0.264
-----------------------------------------------------------------------------------------------------------------------------------------------------------------------------------TGGCCTGCATCTCCTCTGCCTG-------------------      0.097      0.038      0.038
-----------------------------------------------------------------------------------------------------------------------------------------------------------------------------------TGGCCTGCATCTCCTCTGCC---------------------      2.283      0.644      0.188
------------------------------------------------------------------------------------------------------------------------------------------------------------------------------------GGCCTGCATCTCCTCTGC----------------------      0.000      0.038      0.038

----------------------------------------------------------NNN                                                                                                                   -                                  TC-   C
                                                             GTATTGACTGTACCTACCATCATGGTAAACTTTGAAAGTCGAAGGCCCGACAGCAGCGGGTTGAGCTCCTCGCCGCGGGCCTTCAAGCTCCGCGCAATGTCCTCTGGCGTCGCCC ACTGGCCTGCATCTCCTCTGCCTGTGCAAGAAAC   TGC T
                                                             CATAACTGACATGGATGGTAGTACCATTTGAAACTTTCAGCTTCCGGGCTGTCGTCGCCCAACTCGAGGAGCGGCGCCCGGAAGTTCGAGGCGCGTTACAGGAGACCGCAGCGGG TGACCGGACGTAGGAGGACGGACACGTTCTTTG   GCG T
ATGGTGGTGTCGGGTAGGGCGGGGGTCGCGGGTCGGCTGATGCGGGGGTGGTTGGATCTAG                                                                                                                   G                                  CCA   T

-----------------------------------------------------------------------------------------------------------------------------------------CCCGGAAGTTCGAGGCGCGTT--------------------------------------------------------------      1.020      0.530      0.527
------------------------------------------------------------------------------------------------------------------------------------------CCGGAAGTTCGAGGCGCGTT--------------------------------------------------------------      0.049      0.682      0.715
-----------------------------------------------------------------------------------------------------------------------------CGAGGAGCGGCGCCCGGAAGT--------------------------------------------------------------------------      0.340      0.114      0.188
----------------------------------------------------------------------------------------------------------------TCGTCGCCCAACTCGAGGAGC---------------------------------------------------------------------------------------      0.000      0.113      0.113
------------------------------------------------------------------------------------------GAAACTTTCAGCTTCCGGGCT-------------------------------------------------------------------------------------------------------------      0.146      0.379      0.113
-----------------------------------------------------------AGCATAACTGACATGGATGGT--------------------------------------------------------------------------------------------------------------------------------------------      0.000      0.038      0.075
-----------------------------------------------------GGATCTAGCATAACTGACAT---------------------------------------------------------------------------------------------------------------------------------------------------      2.040      0.341      0.113
-------------------------------------------------GGTTGGATCTAGCATAACTGA------------------------------------------------------------------------------------------------------------------------------------------------------      0.146      0.947      0.565
----------------------------------------------GGTGGTTGGATCTAGCATAACT--------------------------------------------------------------------------------------------------------------------------------------------------------      11.996     11.593     1.920
-----------------------------------------------GTGGTTGGATCTAGCATAACT--------------------------------------------------------------------------------------------------------------------------------------------------------      5.245      1.364      0.678
------------------------------------------------TGGTTGGATCTAGCATAACT--------------------------------------------------------------------------------------------------------------------------------------------------------      0.631      0.114      0.151
-------------------------------------------------GGTTGGATCTAGCATAACT--------------------------------------------------------------------------------------------------------------------------------------------------------      0.291      0.606      0.188
----------------------------------------------GGTGGTTGGATCTAGCATAAC---------------------------------------------------------------------------------------------------------------------------------------------------------      0.146      0.606      0.075
-----------------------------------------------GTGGTTGGATCTAGCATAAC---------------------------------------------------------------------------------------------------------------------------------------------------------      0.049      0.113      0.113
---------------------------------------------GGGTGGTTGGATCTAGCATAA----------------------------------------------------------------------------------------------------------------------------------------------------------      0.146      4.167      0.753
----------------------------------------------GGTGGTTGGATCTAGCATAA----------------------------------------------------------------------------------------------------------------------------------------------------------      0.049      0.871      0.264
--------------------------------------------GGGGTGGTTGGATCTAGCATAA----------------------------------------------------------------------------------------------------------------------------------------------------------      0.000      0.493      0.113
--------------------------------------------GGGGTGGTTGGATCTAGCATA-----------------------------------------------------------------------------------------------------------------------------------------------------------      0.000      0.455      0.151
-------------------------------------------GGGGGTGGTTGGATCTAGCATA-----------------------------------------------------------------------------------------------------------------------------------------------------------      0.000      0.076      0.151
-----------------------------------------GCGGGGGTGGTTGGATCTAGCAT------------------------------------------------------------------------------------------------------------------------------------------------------------      0.874      0.265      0.075
-------------------------------------------GGGGGTGGTTGGATCTAGCAT------------------------------------------------------------------------------------------------------------------------------------------------------------      2.380      2.122      1.017
---------------------------------------------GGGTGGTTGGATCTAGCAT------------------------------------------------------------------------------------------------------------------------------------------------------------      0.534      0.909      0.377
--------------------------------------------GGGGTGGTTGGATCTAGCAT------------------------------------------------------------------------------------------------------------------------------------------------------------      1.068      1.819      0.452
-----------------------------------------GCGGGGGTGGTTGGATCTAGC--------------------------------------------------------------------------------------------------------------------------------------------------------------      0.000      0.113      0.113
--------------------------------------GATGCGGGGGTGGTTGGATCT-----------------------------------------------------------------------------------------------------------------------------------------------------------------      0.631      0.038      0.075
--------------------------------------GATGCGGGGGTGGTTGGAT-------------------------------------------------------------------------------------------------------------------------------------------------------------------      0.146      0.341      0.075
------------------------------------CTGATGCGGGGGTGGTTGGAT-------------------------------------------------------------------------------------------------------------------------------------------------------------------      0.049      0.038      0.038
-------------------------------------TGATGCGGGGGTGGTTGGAT-------------------------------------------------------------------------------------------------------------------------------------------------------------------      0.049      0.038      0.264
-------------------------------------TGATGCGGGGGTGGTTGGA--------------------------------------------------------------------------------------------------------------------------------------------------------------------      0.000      0.188      0.188
--------------------------------------GATGCGGGGGTGGTTGGA--------------------------------------------------------------------------------------------------------------------------------------------------------------------      0.000      0.038      0.151
---------------------------------CGGCTGATGCGGGGGTGGTT-----------------------------------------------------------------------------------------------------------------------------------------------------------------------      1.700      1.137      2.523
--------------------------------TCGGCTGATGCGGGGGTGGTT-----------------------------------------------------------------------------------------------------------------------------------------------------------------------      6.168      2.879      3.125
----------------------------------GGCTGATGCGGGGGTGGTT-----------------------------------------------------------------------------------------------------------------------------------------------------------------------      0.340      0.152      0.038
-------------------------------GTCGGCTGATGCGGGGGTGGT------------------------------------------------------------------------------------------------------------------------------------------------------------------------      1.020      0.151      0.151
---------------------------------CGGCTGATGCGGGGGTGGT------------------------------------------------------------------------------------------------------------------------------------------------------------------------      0.000      0.038      0.038
---------------------------------CGGCTGATGCGGGGGTGG-------------------------------------------------------------------------------------------------------------------------------------------------------------------------      0.000      0.038      0.038
-----------------------------GGGTCGGCTGATGCGGGGGT---------------------------------------------------------------------------------------------------------------------------------------------------------------------------      2.040      2.046      5.836
------------------------------GGTCGGCTGATGCGGGGGT---------------------------------------------------------------------------------------------------------------------------------------------------------------------------      0.146      0.341      0.301
---------------------------GCGGGTCGGCTGATGCGGGGGT---------------------------------------------------------------------------------------------------------------------------------------------------------------------------      0.340      0.152      0.113
--------------------------CGCGGGTCGGCTGATGCGGGGGT---------------------------------------------------------------------------------------------------------------------------------------------------------------------------      0.146      0.038      0.038
-------------------------------GTCGGCTGATGCGGGGGT---------------------------------------------------------------------------------------------------------------------------------------------------------------------------      0.000      0.038      0.038
----------------------------CGGGTCGGCTGATGCGGGGGT---------------------------------------------------------------------------------------------------------------------------------------------------------------------------      65.956     26.179     78.916 miR_t102
----------------------------CGGGTCGGCTGATGCGGGGG----------------------------------------------------------------------------------------------------------------------------------------------------------------------------      0.146      0.227      0.489
------------------------------GGTCGGCTGATGCGGGGG----------------------------------------------------------------------------------------------------------------------------------------------------------------------------      0.000      0.075      0.075
--------------------GGGGGTCGCGGGTCGGCTGAT-----------------------------------------------------------------------------------------------------------------------------------------------------------------------------------      1.166      1.250      0.489
---------------------GGGGTCGCGGGTCGGCTGAT-----------------------------------------------------------------------------------------------------------------------------------------------------------------------------------      0.000      0.114      0.113
-----------------GGCGGGGGTCGCGGGTCGGCT--------------------------------------------------------------------------------------------------------------------------------------------------------------------------------------      0.389      0.189      0.527
------------GGTAGGGCGGGGGTCGCGGGT-------------------------------------------------------------------------------------------------------------------------------------------------------------------------------------------      0.874      1.061      1.393
-------------GTAGGGCGGGGGTCGCGGGT-------------------------------------------------------------------------------------------------------------------------------------------------------------------------------------------      0.000      0.038      0.038
------------GGTAGGGCGGGGGTCGCGGG--------------------------------------------------------------------------------------------------------------------------------------------------------------------------------------------      0.000      0.038      0.038
-------TGTCGGGTAGGGCGGGGGT--------------------------------------------------------------------------------------------------------------------------------------------------------------------------------------------------      0.194      0.152      0.188
-----GGTGTCGGGTAGGGCGGGGGT--------------------------------------------------------------------------------------------------------------------------------------------------------------------------------------------------      17.485     5.001      8.095
----TGGTGTCGGGTAGGGCGGGGGT--------------------------------------------------------------------------------------------------------------------------------------------------------------------------------------------------      0.097      0.038      0.038
-----GGTGTCGGGTAGGGCGGGGG---------------------------------------------------------------------------------------------------------------------------------------------------------------------------------------------------      0.194      0.151      0.151
```

miRNA: miR_t107 scaffold_21 Start: 47196 End: 47405 Condition: HS+N

```
----TACTGAAATCGCCGACGTC--------------------------------------------------------------------------------------------------------------------------------------------      0.000      0.038      0.038
-------TGAAATCGCCGACGTCAAGG--------------------------------------------------------------------------------------------------------------------------------------------      0.486      0.985      1.130
--------GAAATCGCCGACGTCAAGG--------------------------------------------------------------------------------------------------------------------------------------------      0.000      0.038      0.038
------------TCGCCGACGTCAAGGAGGAGG--------------------------------------------------------------------------------------------------------------------------------------      0.437      0.076      0.188
---------------------------AGGAGGAGCACGCCGCTGCTG-----------------------------------------------------------------------------------------------------------------------      0.000      0.038      0.038
---------------------------AGGAGGAGCACGCCGCTGCTGG----------------------------------------------------------------------------------------------------------------------      0.000      0.038      0.038
-----------------------------------------------------------------------------------------------------------------------TGATCGGTTGGTAGTATGGA----------------------------      0.000      0.075      0.075
-------------------------------------------------------------------------------------------------------------------------------------TATGGATGCAGCTGGGGTTG--------------      0.049      0.038      0.038

GTGATACTGAAATCGCCGACGTCAAGGAGGAGGAGCACGCCGCTGCTGGGCCCTCCACCTCGTCCGTCGTGCTCAGCTGCCGTTGCTACCACTGCGGCCAGCCCGTCA   -    TC  A         AGTATGGAT    C      TGAC    C-   TC
                                                                                                             GCG TCGC  TG TCGGTTGGT         GCAG TGGGGT    CCTC  GCC  T
                                                                                                             CGC GGCG  AC AGCCGGCCG         TGTC ACCTCA    GGAG  CGG  /
----------------------------------------------------------------------------------------------------------TAT   T    TC  -         ---------    -      ----    TT   GG

-----------------------------------------------------------------------------------------------------------------GGCGTCAC-AGCCGGCCG---------TGTC-----------------------      5.585      0.227      5.309
-------------------------------------------------------------------------------------------------------------------CGTCAC-AGCCGGCCG---------TGT------------------------      0.000      0.076      0.377
------------------------------------------------------------------------------------------------------------------GCGTCAC-AGCCGGCCG---------TGT------------------------      0.000      0.038      0.565
---------------------------------------------------------------------------------------------------------------CTGGCGTCAC-AGCCGGCCG---------TGT------------------------      0.049      0.038      0.038
-----------------------------------------------------------------------------------------------------------------GGCGTCAC-AGCCGGCCG---------TGT------------------------      375.821    46.221     688.633 miR_t107
----------------------------------------------------------------------------------------------------------------TGGCGTCAC-AGCCGGCCG---------TGT------------------------      21.224     1.212      20.106
```

```
------------------------------------------------------------------------------------------------------------------------------TGGCGTCAC-AGCCGGCCG---------TG---------------------------------      0.000      0.038      0.038
-------------------------------------------------------------------------------------------------------------------------------GGCGTCAC-AGCCGGCCG---------TG---------------------------------     16.465      0.758     11.069
------------------------------------------------------------------------------------------------------------------------------TGGCGTCAC-AGCCGGCCG---------T----------------------------------      0.097      0.038      0.038
-------------------------------------------------------------------------------------------------------------------------------GGCGTCAC-AGCCGGCCG---------T----------------------------------      0.729      0.265      1.205
----------------------------------------------------------------------------------------------------------------------------GCTGGCGTCAC-AGCCGGCCG---------T----------------------------------      0.097      0.038      0.038
-----------------------------------------------------------------------------------------------------------------------------CTGGCGTCAC-AGCCGGCCG---------T----------------------------------      0.049      0.114      0.113
--------------------------------------------------------------------------------------------------------------------------------GCGTCAC-AGCCGGCCG---------T----------------------------------      0.049      0.075      0.075

miRNA: miR_t38,miR_t116 chromosome_15 Start: 109972 End: 110410 Condition: HS+N
-------------------------------TCAACAAGGTGCTCACGAACC-------------------------------------------------------------------------------------------------------------------------------------------------------------------      0.000      0.075      0.075 miR_t116*
--------------------------------------------TCACGAACCAGGGCAAAATGC--------------------------------------------------------------------------------------------------------------------------------------------------------    247.455     35.386     91.868 miR_t38
--------------------------------------------TCACGAACCAGGGCAAAATG---------------------------------------------------------------------------------------------------------------------------------------------------------      6.557      1.781      4.857
---------------------------------------------CACGAACCAGGGCAAAATGC--------------------------------------------------------------------------------------------------------------------------------------------------------      0.243      0.303      2.861
----------------------------------------------ACGAACCAGGGCAAAATGC--------------------------------------------------------------------------------------------------------------------------------------------------------      0.486      0.114      0.489
--------------------------------------------------AACCAGGGCAAAATGCAGAAG--------------------------------------------------------------------------------------------------------------------------------------------------      0.049      0.414      0.414
-------------------------------------------------------------------------------------------------------TCTCGCAGCTGAAGCAGGACA-------------------------------------------------------------------------------------------------------      5.100      2.425      1.242
-------------------------------------------------------------------------------------------------------TCTCGCAGCTGAAGCAG-----------------------------------------------------------------------------------------------------------      0.049      0.038      0.038
-------------------------------------------------------------------------------------------------------TCTCGCAGCTGAAGCAGG----------------------------------------------------------------------------------------------------------      0.097      0.076      0.113
-------------------------------------------------------------------------------------------------------TCTCGCAGCTGAAGCAGGA---------------------------------------------------------------------------------------------------------      0.291      0.038      0.038
-------------------------------------------------------------------------------------------------------TCTCGCAGCTGAAGCAGGACAC------------------------------------------------------------------------------------------------------      0.000      0.114      0.113
-------------------------------------------------------------------------------------------------------TCTCGCAGCTGAAGCAGGAC--------------------------------------------------------------------------------------------------------      2.720      2.046      1.581
---------------------------------------------------------------------------------------------------------TCGCAGCTGAAGCAGGACA-------------------------------------------------------------------------------------------------------      0.049      0.075      0.075
--------------------------------------------------------------------------------------------------------------------------------------------------TTGGTCGCTGTCAACCTGACG------------------------------------------------------------------------------------------------     16.902     12.502      6.250
--------------------------------------------------------------------------------------------------------------------------------------------------TTGGTCGCTGTCAACCTGACGC-----------------------------------------------------------------------------------------------      1.166      1.061      0.640
---------------------------------------------------------------------------------------------------------------------------------------------------TGGTCGCTGTCAACCTGACG------------------------------------------------------------------------------------------------     15.202     12.086      7.417
---------------------------------------------------------------------------------------------------------------------------------------------------TGGTCGCTGTCAACCTGACGC-----------------------------------------------------------------------------------------------     14.668      6.327      2.861
------------------------------------------------------------------------------------------------------------------------------------------------------------------------------TGGCGTCACTGGAGCTGGGAG--------------------------------------------------------------------      3.594      0.114      0.113
-----------------------------------------------------------------------------------------------------------------------------------------------------------------------------------------TGGAGCTGGGAGCGCTGTAC----------------------------------------------------------      0.194      0.038      0.038
-----------------------------------------------------------------------------------------------------------------------------------------------------------------------------------------TGGAGCTGGGAGCGCTGTACT---------------------------------------------------------      5.828      1.743      0.828
-----------------------------------------------------------------------------------------------------------------------------------------------------------------------------------------------TGGGAGCGCTGTACTTTGTGT---------------------------------------------------      0.243      0.152      0.113
---------------------------------------------------------------------------------------------------------------------------------------------------------------------------------------------------------------------------------TTCAAGGTCTCGGGCGGCGG------------------      0.000      0.038      0.038

--------------------------                                                                       TA                              T      C                                                    A         C            G        C     TC         C                 GC   GA
                          AAGCTCAACAAGGTGCTCACGAACCAGGGCAAAATGCAGAAGGACATCGGCGATATGCAGAA  ATATCTCGCAGCTGAAGCAGGACAC GCAGT AACAAGGCGTTGGTCGCTGTCAACCTGACGCTGGTGGCGTCACTGG GCTGGGAG GCTGTACTTT TGTACAC AACT  AAGGTCT GGGCGGCGGCTGAAG  TGG  T
                          TTCGAGTTGTTCCACGAGTGCTTGGTCCCGTTTTACGTCTTCCTGTAGCCGCTGTACGTCTT  TGTAGAGTGTCGACTTCGTCCTGTG CGTCA TTGTTCCGCAACCAGCGACAGTTGGACTGCGGCTACCGCAGTGACC CGACCCTC CGACATGAAG ACATGTG TTGA  TTCCAGA CCCGCCGTCGACTTC  GTC  /
TGCCGGCCAGTCAGGACCTCGCG                                                                          CC                              C      C                                                    A         A            G        A     GA         A                 AT   GG

--------------------------------------------------------------------------------------------------------------------------------------------------------------------------------------------CGACCCTCACGACATGAA---------------------------------------------------------      0.000      0.076      0.075
------------------------------------------------------------------------------------------------------------------------------------------------------------------------------------TGACCACGACCCTCACGACAT------------------------------------------------------------      1.457      0.189      0.339
-------------------------------------------------------------------------------------------------------------------------------------------------------------------------------------GACCACGACCCTCACGACAT------------------------------------------------------------      0.194      0.076      0.113
---------------------------------------------------------------------------------------------------------------------CGTCCTGTGCCGTCACTTGTT-----------------------------------------------------------------------------------------------------------------------------      2.477      0.265      0.527
--------------------------------------------------------------------------------------------------------------------TCGTCCTGTGCCGTCACTTGT------------------------------------------------------------------------------------------------------------------------------      0.291      0.075      0.075
---------------------------------------------------------------------------------------------------------------CGACTTCGTCCTGTGCCGT-------------------------------------------------------------------------------------------------------------------------------------      0.000      0.075      0.075
------------------------------------------------------------------------------------------------------------GTCGACTTCGTCCTGTGCCGT--------------------------------------------------------------------------------------------------------------------------------------      0.437      0.076      0.038
-------------------------------------------------------------------------------------------------CCTGTAGAGTGTCGACTTCGT-------------------------------------------------------------------------------------------------------------------------------------------------      0.340      0.038      0.075
----------------------------------------------------------------------------------------------CTTCCTGTAGAGTGTCGACTT----------------------------------------------------------------------------------------------------------------------------------------------------      0.000      0.076      0.038
---------------------------------------------------------------------------------------GTACGTCTTCCTGTAGAGTGT-----------------------------------------------------------------------------------------------------------------------------------------------------------      1.846      0.871      0.489
----------------------------------------------------GGTCCCGTTTTACGTCTTCCT----------------------------------------------------------------------------------------------------------------------------------------------------------------------------------------------      3.011      0.189      0.226
------------------------------------------------GCTTGGTCCCGTTTTACGTCT--------------------------------------------------------------------------------------------------------------------------------------------------------------------------------------------------      0.583      0.038      0.038
------------------------------------------CGAGTGCTTGGTCCCGTTTTA--------------------------------------------------------------------------------------------------------------------------------------------------------------------------------------------------------      0.049      0.075      0.075 miR_t38*
-------------------------------------------GAGTGCTTGGTCCCGTTTT---------------------------------------------------------------------------------------------------------------------------------------------------------------------------------------------------------      0.097      0.038      0.038
-----------------------------------------ACGAGTGCTTGGTCCCGTTTT---------------------------------------------------------------------------------------------------------------------------------------------------------------------------------------------------------      1.748      0.114      0.188
------------------------------------------CGAGTGCTTGGTCCCGTTTT---------------------------------------------------------------------------------------------------------------------------------------------------------------------------------------------------------      4.808      1.440      1.242
------------------------------------------CGAGTGCTTGGTCCCGTTT----------------------------------------------------------------------------------------------------------------------------------------------------------------------------------------------------------      0.049      0.038      0.038
---------------------------------------CCACGAGTGCTTGGTCCCGTT-----------------------------------------------------------------------------------------------------------------------------------------------------------------------------------------------------------      0.243      0.265      0.151
-----------------------------CGAGTTGTTCCACGAGTGCTT---------------------------------------------------------------------------------------------------------------------------------------------------------------------------------------------------------------------     25.401     48.040     34.074 miR_t116
-----------------------------CGAGTTGTTCCACGAGTGCT----------------------------------------------------------------------------------------------------------------------------------------------------------------------------------------------------------------------      1.506      0.417      0.339
----GGCCAGTCAGGACCTCGCGT---------------------------------------------------------------------------------------------------------------------------------------------------------------------------------------------------------------------------------------------      0.000      0.038      0.038

miRNA: miR_t118 scaffold_21 Start: 47228 End: 47408 Condition: HS+N
----TCAAGCCCAGGAGGCGGAGGGTC-------------------------------------------------------------------------------------------------------------------------------      0.097      0.301      0.301
----TCAAGCCCAGGAGGCGGAGGGT--------------------------------------------------------------------------------------------------------------------------------      0.000      0.151      0.151
----TCAAGCCCAGGAGGCGGAGGG---------------------------------------------------------------------------------------------------------------------------------      0.000      0.075      0.075
----TCAAGCCCAGGAGGCGGAGGGTCA------------------------------------------------------------------------------------------------------------------------------      0.049      0.113      0.113

GTCCTCAAGCCCAGGAGGCGGAGGGTCAACCCCAGCTGCATCCATACTACCAACCGATCAGAGCGACGCTGACGGGCTGGCCGCAGTGGTAGCAACGGCA     ----  A     GA     T     -  CCA  A
                                                                                                              GCTGA    GC CGACG  CGAGG GGAGG GC    GC G
                                                                                                              TGACT    CG GCTGC  GTTCC CCTCC CG    CG G
-----------------------------------------------------------------------------------------------------TGCCACTA     TTAG  -     A-     T     T  TG-  G

---------------------------------------------------------------------------------------------------------------GACTTTAGCG-GCTGCA-G------------------------      0.000      0.113      0.113
-------------------------------------------------------------------------------------------------------------ATGACTTTAGCG-GCTGCA-G------------------------      1.700      0.417      2.108
------------------------------------------------------------------------------------------------------------TATGACTTTAGCG-GCTGCA-G------------------------      0.194      0.264      0.264
--------------------------------------------------------------------------------------------------------------TGACTTTAGCG-GCTGCA-G------------------------      0.389      0.038      0.941
-----------------------------------------------------------------------------------------------------------CTATGACTTTAGCG-GCTGCA-GT-----------------------     14.862      5.531     11.220
-------------------------------------------------------------------------------------------------------------ATGACTTTAGCG-GCTGCA-GT-----------------------      5.537      0.909     13.140
----------------------------------------------------------------------------------------------------------------ACTTTAGCG-GCTGCA-GT-----------------------      0.000      0.113      0.113
---------------------------------------------------------------------------------------------------------------GACTTTAGCG-GCTGCA-GT-----------------------      0.194      0.568      1.130
------------------------------------------------------------------------------------------------------------TATGACTTTAGCG-GCTGCA-GT-----------------------     26.518     10.457    100.114
----------------------------------------------------------------------------------------------------------ACTATGACTTTAGCG-GCTGCA-GT-----------------------      0.243      0.076      0.602
--------------------------------------------------------------------------------------------------------------TGACTTTAGCG-GCTGCA-GT-----------------------      1.311      0.758      5.535
-----------------------------------------------------------------------------------------------------------CTATGACTTTAGCG-GCTGCA-GTT----------------------      6.654     14.321      0.113
--------------------------------------------------------------------------------------------------------------TGACTTTAGCG-GCTGCA-GTT----------------------     97.816     55.806    390.175 miR_t118
------------------------------------------------------------------------------------------------------------TATGACTTTAGCG-GCTGCA-GTT----------------------     28.121     12.919     39.082
---------------------------------------------------------------------------------------------------------------GACTTTAGCG-GCTGCA-GTT----------------------      4.517      7.085     55.761
----------------------------------------------------------------------------------------------------------------ACTTTAGCG-GCTGCA-GTT----------------------      0.291      0.379      3.803
-----------------------------------------------------------------------------------------------------------------CTTTAGCG-GCTGCA-GTT----------------------      0.146      0.114      0.828
-------------------------------------------------------------------------------------------------------------ATGACTTTAGCG-GCTGCA-GTT----------------------    262.511     54.973    371.651
---------------------------------------------------------------------------------------------------------------GACTTTAGCG-GCTGCA-GTTC---------------------      0.049      0.152      0.791
----------------------------------------------------------------------------------------------------------------ACTTTAGCG-GCTGCA-GTTC---------------------      0.097      0.188      0.188
-----------------------------------------------------------------------------------------------------------------CTTTAGCG-GCTGCA-GTTC---------------------      0.000      0.075      0.075
-------------------------------------------------------------------------------------------------------------ATGACTTTAGCG-GCTGCA-GTTC---------------------      0.146      0.076      0.226
------------------------------------------------------------------------------------------------------------TATGACTTTAGCG-GCTGCA-GTTC---------------------      0.000      0.038      0.038
--------------------------------------------------------------------------------------------------------------TGACTTTAGCG-GCTGCA-GTTC---------------------      0.049      0.640      0.640
--------------------------------------------------------------------------------------------------------------TGACTTTAGCG-GCTGCA-GTTCCT-------------------      0.194      0.227      0.264
-----------------------------------------------------------------------------------------------------------------CTTTAGCG-GCTGCA-GTTCCT-------------------      0.389      0.303      3.577
---------------------------------------------------------------------------------------------------------------GACTTTAGCG-GCTGCA-GTTCCT-------------------      4.323      2.993     20.520
------------------------------------------------------------------------------------------------------------------TTTAGCG-GCTGCA-GTTCCT-------------------      0.000      0.038      0.038
----------------------------------------------------------------------------------------------------------------ACTTTAGCG-GCTGCA-GTTCCT-------------------      4.711      2.235     19.240
--------------------------------------------------------------------------------------------------------------------TAGCG-GCTGCA-GTTCCT-------------------      0.000      0.038      0.038
-------------------------------------------------------------------------------------------------------------------TTAGCG-GCTGCA-GTTCCT-------------------      0.000      0.038      0.038
--------------------------------------------------------------------------------------------------------------------TAGCG-GCTGCA-GTTCCTCCT----------------      0.000      0.038      0.038
---------------------------------------------------------------------------------------------------------------------AGCG-GCTGCA-GTTCCTCCT----------------      0.049      0.038      0.038
-------------------------------------------------------------------------------------------------------------------TTAGCG-GCTGCA-GTTCCTCCT----------------      0.146      0.076      1.355
----------------------------------------------------------------------------------------------------------------------GCG-GCTGCA-GTTCCTCCTC---------------      0.000      0.038      0.038
----------------------------------------------------------------------------------------------------------------------GCG-GCTGCA-GTTCCTCCTCCT-------------      0.389      0.341      1.167
-----------------------------------------------------------------------------------------------------------------------CG-GCTGCA-GTTCCTCCTCCT-------------      0.000      0.076      1.770
---------------------------------------------------------------------------------------------------------------------AGCG-GCTGCA-GTTCCTCCTCCT-------------      0.340      0.264      0.264
------------------------------------------------------------------------------------------------------------------------G-GCTGCA-GTTCCTCCTCCT-------------      0.049      0.565      0.565
```

miRNAs identified in HS-N Only :

```
miRNA: miR_t11 chromosome_6 Start: 6854037 End: 6854272 Condition: HS-N
------------------------------------------------------------------------------------------TACGCAACTGCGAAGCTCTTC------------------------------------      0.777      0.076      0.301
------------------------------------------------------------------------------------TCCTGTACGCAACTGCGAAGC------------------------------------------      1.457      0.833      0.188
---------------------------------------------------------------------------------TGCTCCTGTACGCAACTGC-----------------------------------------------      0.000      0.038      0.038
---------------------------------------------------------------------------------TGCTCCTGTACGCAACTGCGA---------------------------------------------      0.097      0.114      0.114
---------------------------------------------------------------------------TCATCATGCTCCTGTACGCAAC--------------------------------------------------      0.243      0.152      0.113
---------------------------------------------------------------------------TCATCATGCTCCTGTACGC-----------------------------------------------------      0.000      0.076      0.113
-----------------------------------------------------------------------TCCTCATCATGCTCCTGTACGC------------------------------------------------------      0.097      0.076      0.113
-----------------------------------------------------------------------TCCTCATCATGCTCCTGTACG-------------------------------------------------------      0.826      0.417      0.377
-------------------------------------------------------------TGGTCGGCATCCTCATCATGC-----------------------------------------------------------------      3.545      0.379      0.452
--------------------------------------------------GGGCTGCTGGTGGTCGGCAT-----------------------------------------------------------------------------      0.340      0.417      0.828
--------------------------------------------------GGGCTGCTGGTGGTCGGC-------------------------------------------------------------------------------      0.049      0.227      0.113
--------------------------------------------------GGGCTGCTGGTGGTCGGCA------------------------------------------------------------------------------      0.631      0.076      0.226
-------------------------------------------------TGGGCTGCTGGTGGTCGGC-------------------------------------------------------------------------------      6.314     14.662     15.663
-------------------------------------------------TGGGCTGCTGGTGGTCGGCATC----------------------------------------------------------------------------      0.680      1.819      0.866
-------------------------------------------------TGGGCTGCTGGTGGTCGG--------------------------------------------------------------------------------      0.340      0.303      0.151
-------------------------------------------------TGGGCTGCTGGTGGTCGGCA------------------------------------------------------------------------------     79.943     20.648     26.017
-------------------------------------------------TGGGCTGCTGGTGGTCGGCAT-----------------------------------------------------------------------------     39.729     36.787     85.882
------------------------------------------------TTGGGCTGCTGGTGGTCGGCATC----------------------------------------------------------------------------      0.000      0.038      0.038
------------------------------------------------TTGGGCTGCTGGTGGTCGGCAT-----------------------------------------------------------------------------      2.477      3.637      0.828
------------------------------------------------TTGGGCTGCTGGTGGTCGGC-------------------------------------------------------------------------------     73.095    127.411    168.149
------------------------------------------------TTGGGCTGCTGGTGGTCGGCA------------------------------------------------------------------------------    122.683     21.785     20.934
------------------------------------------TCGGCGTTGGGCTGCTGGTG-------------------------------------------------------------------------------------      0.389      0.076      0.188
------------------------------------------TCGGCGTTGGGCTGCTGGTGG------------------------------------------------------------------------------------     31.132      3.410      7.944
------------------------------------------TCGGCGTTGGGCTGCTGGTGGT-----------------------------------------------------------------------------------      0.146      0.189      0.189
------------------------------------------TCGGCGTTGGGCTGCTGG---------------------------------------------------------------------------------------      0.000      0.076      0.076
--------------------------------------TGGTCGGCGTTGGGCTGCTGG----------------------------------------------------------------------------------------      2.526      0.038      0.264
-----------------------------------TGTTGGTCGGCGTTGGGCTG--------------------------------------------------------------------------------------------      0.146      0.038      0.038
-----------------------------------TGTTGGTCGGCGTTGGG-----------------------------------------------------------------------------------------------      0.000      0.038      0.038
-----------------------------------TGTTGGTCGGCGTTGGGCTGC-------------------------------------------------------------------------------------------      0.194      0.341      0.264
----------------------------------TTGTTGGTCGGCGTTGG------------------------------------------------------------------------------------------------      0.000      0.114      0.113
----------------------------------TTGTTGGTCGGCGTTGGGCTG--------------------------------------------------------------------------------------------     10.296      1.137      2.033
----------------------------------TTGTTGGTCGGCGTTGGG-----------------------------------------------------------------------------------------------      0.000      0.076      0.038
----------------------------------TTGTTGGTCGGCGTTGGGC----------------------------------------------------------------------------------------------      0.000      0.189      0.075
----------------------------------TTGTTGGTCGGCGTTGGGCT---------------------------------------------------------------------------------------------      0.146      0.076      0.076
----------------------------------TTGTTGGTCGGCGTTGGGCTGC-------------------------------------------------------------------------------------------      1.020      0.833      0.866
--------------------------------GGTTGTTGGTCGGCGTTG-------------------------------------------------------------------------------------------------      0.000      0.038      0.113
--------------------------------GGTTGTTGGTCGGCGTTGG------------------------------------------------------------------------------------------------      1.020      0.303      0.226
--------------------------------GGTTGTTGGTCGGCGTTGGG-----------------------------------------------------------------------------------------------      0.243      0.038      0.113
-------------------------------TGGTTGTTGGTCGGCGTTG-------------------------------------------------------------------------------------------------      3.108      3.978      4.330
-------------------------------TGGTTGTTGGTCGGCGT---------------------------------------------------------------------------------------------------      0.097      0.076      0.264
-------------------------------TGGTTGTTGGTCGGCGTTGGGC----------------------------------------------------------------------------------------------      1.603      5.380      2.899
-------------------------------TGGTTGTTGGTCGGCGTTGGG-----------------------------------------------------------------------------------------------     25.838      7.501     11.295
-------------------------------TGGTTGTTGGTCGGCGTTGG------------------------------------------------------------------------------------------------     87.908     21.936     35.655
-------------------------------TGGTTGTTGGTCGGCGTT--------------------------------------------------------------------------------------------------      0.000      0.076      0.226
----------------------------TCTGGTTGTTGGTCGGCGTT---------------------------------------------------------------------------------------------------      0.631      0.076      0.038
----------------------------TCTGGTTGTTGGTCGGCGTTG--------------------------------------------------------------------------------------------------      4.857      0.833      1.958
----------------------------TCTGGTTGTTGGTCGGCGTTGG-------------------------------------------------------------------------------------------------      0.583      0.227      0.188
----------------------------TCTGGTTGTTGGTCGGCGT----------------------------------------------------------------------------------------------------      2.040      0.985      1.054
---------------------------ATCTGGTTGTTGGTCGGCGT----------------------------------------------------------------------------------------------------      0.000      0.379      0.113
---------------------------ATCTGGTTGTTGGTCGGCGTTG--------------------------------------------------------------------------------------------------      0.000      0.038      0.226
---------------------------ATCTGGTTGTTGGTCGGCGTT---------------------------------------------------------------------------------------------------      0.000      0.152      0.113
--------------------------GATCTGGTTGTTGGTCGGC------------------------------------------------------------------------------------------------------      0.000      0.038      0.038
-------------------------TGATCTGGTTGTTGGTCGGC------------------------------------------------------------------------------------------------------     15.882     14.965      8.321
```

```
---------------------TGATCTGGTTGTTGGTCGGCGTT-----------------------------------------------------------------------------     0.194     0.114     0.114
---------------------TGATCTGGTTGTTGGTCGGCGT------------------------------------------------------------------------------     9.519     6.479     3.539
---------------------TGATCTGGTTGTTGGTCGGCG-------------------------------------------------------------------------------    15.445     4.433     3.765
------------------TCCTGATCTGGTTGTTGGTC-----------------------------------------------------------------------------------     0.243     0.076     0.076
------------------TCCTGATCTGGTTGTTGGTCGGC--------------------------------------------------------------------------------     0.000     0.152     0.152
---------------ACGTCCTGATCTGGTTGTTGG-------------------------------------------------------------------------------------     0.049     0.189     0.075

-------------TTC              T                                        T                           ATTAAACTGAACCAGAAGGAT   C   T
              GACGTCCTGATCTGGT GTTGGTCGGCGTTGGGCTGCTGGTGGTCGGCATCCTCATCA GCTCCTGTACGCAACTGCGAAGCTCTTCC                    GCG ACC G
              CTGCAGGACTGGACCA CAACCAGCCGCAACCCGACGACCACCAGCCGTAGGAGTAGT CGAGGACATGCGTTGACGCTTCGAGAAGG                    CGC TGG C
CGACGTCCCGGTGACG              C                                        C                           AG-------------------   -   C

----GTCCCGGTGACGCTGCAGGACT-----------------------------------------------------------------------------------------------     0.000     0.038     0.038
------CCCGGTGACGCTGCAGGACT-----------------------------------------------------------------------------------------------    26.178     3.258     3.614
------------GACGCTGCAGGACTGGACCAC----------------------------------------------------------------------------------------     0.000     0.076     0.076
--------------GCTGCAGGACTGGACCACCAA--------------------------------------------------------------------------------------     1.457     2.084     3.878
------------------------------------------CCCGACGACCACCAGCCGT------------------------------------------------------------     0.194     0.038     0.038
----------------------------------------------CGACCACCAGCCGTAGGAGT-------------------------------------------------------     0.000     0.152     0.152
--------------------------------------------------------CGTAGGAGTAGTCCGAGGAC---------------------------------------------     0.437     0.493     0.188
--------------------------------------------------------CGTAGGAGTAGTCCGAGGACA--------------------------------------------     1.068     1.781     1.130
---------------------------------------------------------GTAGGAGTAGTCCGAGGACA--------------------------------------------     0.486     0.227     0.339
---------------------------------------------------------GTAGGAGTAGTCCGAGGACAT-------------------------------------------    33.221    58.269    32.003
----------------------------------------------------------TAGGAGTAGTCCGAGGACAT-------------------------------------------     0.097     0.152     0.152
--------------------------------------------------------CGTAGGAGTAGTCCGAGGACAT-------------------------------------------   135.797   355.826   125.340 miR_t11
-----------------------------------------------------------AGGAGTAGTCCGAGGACAT-------------------------------------------     0.486     0.076     0.602
-------------------------------------------------------CCGTAGGAGTAGTCCGAGGACAT-------------------------------------------     0.243     0.189     0.189
-----------------------------------------------------------AGGAGTAGTCCGAGGACATGC-----------------------------------------     0.000     0.038     0.038
------------------------------------------------------------GAGTAGTCCGAGGACATGCG-----------------------------------------     0.194     0.227     0.227
--------------------------------------------------------------GTAGTCCGAGGACATGCGT----------------------------------------     0.097     0.114     0.114
----------------------------------------------------------------AGTCCGAGGACATGCGT----------------------------------------     0.000     0.076     0.075
-----------------------------------------------------------GGAGTAGTCCGAGGACATGCGT----------------------------------------     0.389     3.940     0.075
------------------------------------------------------------GAGTAGTCCGAGGACATGCGT----------------------------------------    67.801     6.479     6.965
-------------------------------------------------------------AGTAGTCCGAGGACATGCGT----------------------------------------     5.100     2.500     2.786
---------------------------------------------------------TAGGAGTAGTCCGAGGACATGCGT----------------------------------------     0.000     0.341     0.341
-------------------------------------------------------------AGTAGTCCGAGGACATGCGTT---------------------------------------     1.894     0.909     0.904
--------------------------------------------------------------GTAGTCCGAGGACATGCGTT---------------------------------------     4.371     3.713     3.803
---------------------------------------------------------------TAGTCCGAGGACATGCGTT---------------------------------------     0.000     0.038     0.038
-----------------------------------------------------------GGAGTAGTCCGAGGACATGCGTT---------------------------------------     0.000     0.114     0.114
--------------------------------------------------------------------CGAGGACATGCGTTGACGCTT--------------------------------     0.631     0.417     0.489

miRNA: miR_t88,miR_t25,miR_t53 chromosome_2 Start: 9173273 End: 9173464 Condition: HS-N
----------------------------------------------------GAGGGCGCAAGCACGCAACG------------------------------------     0.000     0.038     0.038
---------------------------------------------------GGAGGGCGCAAGCACGCAAC-------------------------------------     0.291     0.038     0.339
---------------------------------------------------GGAGGGCGCAAGCACGCAACG------------------------------------     0.291     0.038     0.452
--------------------------------------------------TGGAGGGCGCAAGCACGCAAC-------------------------------------    29.772     3.372    13.856
--------------------------------------------------TGGAGGGCGCAAGCACGCAACG------------------------------------    47.305    12.957    23.871 miR_t88
-------------------------------------------------CTGGAGGGCGCAAGCACGCAAC-------------------------------------     0.000     0.038     0.075
------------------------------------------TAGACGGCTGGAGGGCGCAAG---------------------------------------------     0.243     0.303     0.565
------------------------------------------TAGACGGCTGGAGGGCGCAAGC--------------------------------------------     0.000     0.038     0.075

--------------------------------------- A                                               C           CCT  TC
                                      G GCTAGACGGCTGGAGGGCGCAAGCACGCAACGAGGGGATGACGAGCACGATG GGGCCTGATACAG   CG  C
                                      C CGATCTGCCGACCTCCCGCGTTCGTGCGTTGCTCCCCTACTGCTCGTGCTAC CCCGGACTATGTC   GC  /
ACAGCGGTCGGTGTAGCGGTCGCCGTCCGAGTGTCGAGGACTG -                                               A           C--  CC

----CGGTCGGTGTAGCGGTCGCCGT----------------------------------------------------------------------------------     0.000     0.038     0.038
------------------------CGAGTGTCGAGGACTGC-CG----------------------------------------------------------------     0.049     0.038     0.075
------------------------CGAGTGTCGAGGACTGC-CGA---------------------------------------------------------------     0.000     0.379     0.038
-----------------------CCGAGTGTCGAGGACTGC-CGA---------------------------------------------------------------     0.049     0.303     0.113
-------------------------GAGTGTCGAGGACTGC-CGAT--------------------------------------------------------------     2.380     1.819     0.527
-----------------------CCGAGTGTCGAGGACTGC-CGAT--------------------------------------------------------------    10.782    30.347     1.130
--------------------CGTCCGAGTGTCGAGGACTGC-CGAT--------------------------------------------------------------     0.097     0.038     0.038
---------------------GTCCGAGTGTCGAGGACTGC-CGAT--------------------------------------------------------------     0.049     0.265     0.038
----------------------TCCGAGTGTCGAGGACTGC-CGAT--------------------------------------------------------------     0.486     0.796     0.796
------------------------CGAGTGTCGAGGACTGC-CGAT--------------------------------------------------------------    14.619    73.310    15.437 miR_t25
------------------------CGAGTGTCGAGGACTGC-CGATC-------------------------------------------------------------     0.000     0.038     0.038
---------------------------GTGTCGAGGACTGC-CGATCT------------------------------------------------------------     0.049     0.038     0.038
--------------------CGTCCGAGTGTCGAGGACTGC-CGATCT------------------------------------------------------------     0.000     0.038     0.038
------------------------CGAGTGTCGAGGACTGC-CGATCT------------------------------------------------------------     0.146     2.993     2.993
-----------------------CCGAGTGTCGAGGACTGC-CGATCT------------------------------------------------------------     0.000     0.227     0.227
-------------------------GAGTGTCGAGGACTGC-CGATCT------------------------------------------------------------     0.146     0.076     0.076
---------------------------------------------TGCCGACCTCCCGCGTTCGT-------------------------------------------     0.049     0.038     0.038
--------------------------------------------CTGCCGACCTCCCGCGTTCGT-------------------------------------------     0.971     0.152     0.678
------------------------------------------------CGACCTCCCGCGTTCGTGCGT---------------------------------------     2.817     0.871     1.355
------------------------------------------------CGACCTCCCGCGTTCGTGCGTT--------------------------------------    19.670    15.117    10.354 miR_t88*
--------------------------------------------------ACCTCCCGCGTTCGTGCGTT--------------------------------------     0.583     0.038     0.038
---------------------------------------------------CCTCCCGCGTTCGTGCGTT--------------------------------------     0.194     0.152     0.075
-------------------------------------------------GACCTCCCGCGTTCGTGCGTT--------------------------------------     0.389     0.152     0.113
--------------------------------------------------------GCGTTCGTGCGTTGCTCCCCT-------------------------------     0.243     0.076     0.151
---------------------------------------------------------CGTTCGTGCGTTGCTCCCCT-------------------------------     0.146     0.265     0.264
-------------------------------------------------------------CGTGCGTTGCTCCCCTACT----------------------------     0.049     0.038     0.075
-----------------------------------------------------------------GTTGCTCCCCTACTGCTCG------------------------     0.146     0.114     0.113
---------------------------------------------------------------GCGTTGCTCCCCTACTGCTCG------------------------     0.146     0.227     0.075
----------------------------------------------------------------CGTTGCTCCCCTACTGCTCG------------------------     0.146     0.038     0.113
--------------------------------------------------------------------GCTCCCCTACTGCTCGT-----------------------     0.000     0.076     0.038
----------------------------------------------------------------CGTTGCTCCCCTACTGCTCGT-----------------------     4.128     1.212     1.619
-----------------------------------------------------------------GTTGCTCCCCTACTGCTCGT-----------------------     2.186     0.909     1.280
---------------------------------------------------------------GCGTTGCTCCCCTACTGCTCGT-----------------------     5.585     4.774     1.770
--------------------------------------------------------------------GCTCCCCTACTGCTCGTG----------------------     0.971     0.114     0.414
--------------------------------------------------------------------GCTCCCCTACTGCTCGTGC---------------------     2.768     0.682     1.431
-----------------------------------------------------------------GTTGCTCCCCTACTGCTCGTGCT--------------------     0.000     0.038     0.038
--------------------------------------------------------------------GCTCCCCTACTGCTCGTGCT--------------------   413.461   144.725   116.303 miR_t53
-------------------------------------------------------------------TGCTCCCCTACTGCTCGTGCT--------------------    13.551     1.326     1.167
---------------------------------------------------------------------CTCCCCTACTGCTCGTGCT--------------------     0.049     0.189     0.189

miRNA: miR_t31 chromosome_16 Start: 858784 End: 859179 Condition: HS-N
-------------------------------------------------------------------------------------------------------------------------------------------------TACTACACGCTCACTGCCAGC------------------------------     0.583     0.152     0.151
--------------------------------------------------------------------------------------------------------------------------------------------TGCACTACTACACGCTCACT------------------------------------     0.097     0.114     0.114
--------------------------------------------------------------------------------------------------------------------------------------------TGCACTACTACACGCTCACTG-----------------------------------     0.097     0.076     0.038
--------------------------------------------------------------------------------------------------------------------------------------------TGCACTACTACACGCTCACTGC----------------------------------     0.243     0.076     0.075
-------------------------------------------------------------------------------------------------------------------------------------------TTGCACTACTACACGCTCACT------------------------------------     0.437     0.076     0.301
----------------------------------------------------------------------------------------------------------------------------------------TACTTGCACTACTACACGCTC---------------------------------------     0.631     0.114     0.114
----------------------------------------------------------------------------------------------------------------------------------------TACTTGCACTACTACACGCT----------------------------------------     0.583     0.076     0.113
--------------------------------------------------------------------------------------------------------------------------------------TATACTTGCACTACTACACG------------------------------------------     0.340     0.114     0.114
--------------------------------------------------------------------------------------------------------------------------------------TATACTTGCACTACTACACGC-----------------------------------------    34.483    10.229     7.003
--------------------------------------------------------------------------------------------------------------------------------TGACTGTATACTTGCACTAC------------------------------------------------     0.194     0.152     0.038
--------------------------------------------------------------------------------------------------------------------------------TGACTGTATACTTGCACTACT-----------------------------------------------     0.680     0.189     0.038
-------------------------------------------------------------------------------------------------------------------------TGCCCGCTGACTGTATACT--------------------------------------------------------     0.049     0.189     0.151
-------------------------------------------------------------------------------------------------------------------------TGCCCGCTGACTGTATACTTGC-----------------------------------------------------     0.583     0.682     0.226
-------------------------------------------------------------------------------------------------------------------------TGCCCGCTGACTGTATACTTG------------------------------------------------------     1.846     2.046     1.920
-------------------------------------------------------------------------------------------------------------TGCCCGGCCACGGCTGCCCGC------------------------------------------------------------------     0.291     0.038     0.188
-----------------------------------------------------------------------------------------------------TCAAGCGGCTGCCCGGCCAC---------------------------------------------------------------------------     0.049     0.038     0.038
-----------------------------------------------------------------------------------------------------TCAAGCGGCTGCCCGGCCACG--------------------------------------------------------------------------     0.194     0.227     0.151
-------------------------------------------------------------------------------------------------CGCGTCAAGCGGCTGCCCGGC------------------------------------------------------------------------------     0.000     0.076     0.076
---------------------------------------------------------------TCGCGGCCGGCGCCAAGGCAGC---------------------------------------------------------------------------------------------------------------     0.097     0.076     0.076
-----------------------------------------------------------CGCACTCGCGGCCGGCGCCA---------------------------------------------------------------------------------------------------------------------     0.000     0.038     0.038
----------------------------------------------------------TCGCACTCGCGGCCGGCGC-----------------------------------------------------------------------------------------------------------------------     0.097     0.038     0.075
----------------------------------------------------------TCGCACTCGCGGCCGGCGCCA---------------------------------------------------------------------------------------------------------------------     0.777     0.076     0.076
----------------------------------------------------------TCGCACTCGCGGCCGGCGCC----------------------------------------------------------------------------------------------------------------------     0.146     0.038     0.038
--------------------------------------------------ACGGCCGCTCGCACTCGCGGC-----------------------------------------------------------------------------------------------------------------------------     0.000     0.038     0.038
------------------------------------------------AGGACGGCCGCTCGCACTCG--------------------------------------------------------------------------------------------------------------------------------     0.000     0.189     0.188
------------------------------------------------AGGACGGCCGCTCGCACTCGC-------------------------------------------------------------------------------------------------------------------------------     0.000     0.076     0.414
----------------------------------------------AAAGGACGGCCGCTCGCACTCG--------------------------------------------------------------------------------------------------------------------------------     0.049     0.265     0.489
---------------------------------------------AAAAGGACGGCCGCTCGCA------------------------------------------------------------------------------------------------------------------------------------     0.097     0.038     0.075
---------------------------------------------AAAAGGACGGCCGCTCGC-------------------------------------------------------------------------------------------------------------------------------------     0.049     0.038     0.038
---------------------------------------------AAAAGGACGGCCGCTCGCACT----------------------------------------------------------------------------------------------------------------------------------     0.049     0.152     0.678
--------------------------------------------CAAAAGGACGGCCGCTCGC-------------------------------------------------------------------------------------------------------------------------------------     0.000     0.038     0.038
--------------------------------------------CAAAAGGACGGCCGCTCGCAC-----------------------------------------------------------------------------------------------------------------------------------     0.097     0.152     0.226
-------------------------------------------TCAAAAGGACGGCCGCTCGC-------------------------------------------------------------------------------------------------------------------------------------     3.983     5.153     9.977
-------------------------------------------TCAAAAGGACGGCCGCTCGCAC-----------------------------------------------------------------------------------------------------------------------------------     1.603     0.833     0.753
-------------------------------------------TCAAAAGGACGGCCGCTCGCACT----------------------------------------------------------------------------------------------------------------------------------     0.680     0.038     0.038
-------------------------------------------TCAAAAGGACGGCCGCTCG--------------------------------------------------------------------------------------------------------------------------------------     0.146     0.152     0.226
-------------------------------------------TCAAAAGGACGGCCGCTC---------------------------------------------------------------------------------------------------------------------------------------     0.000     0.076     0.151
-------------------------------------------TCAAAAGGACGGCCGCTCGCA------------------------------------------------------------------------------------------------------------------------------------     9.616    10.381    16.905
-------------------TGGACGTGCGTGCCCACATT-------------------------------------------------------------------------------------------------------------------------------------------------------------     0.000     0.038     0.038
----------------TGCTGGACGTGCGTGCCCACA---------------------------------------------------------------------------------------------------------------------------------------------------------------     1.943     0.038     0.075
-------------TGGTGCTGGACGTGCGTGC--------------------------------------------------------------------------------------------------------------------------------------------------------------------     0.874     2.387     2.485
-------------TGGTGCTGGACGTGCGTGCC-------------------------------------------------------------------------------------------------------------------------------------------------------------------     0.389     0.379     0.640
-------------TGGTGCTGGACGTGCGTG---------------------------------------------------------------------------------------------------------------------------------------------------------------------     1.068     0.871     1.355
-------------TGGTGCTGGACGTGCGTGCCC------------------------------------------------------------------------------------------------------------------------------------------------------------------     4.274     1.629     1.958
--------GCCGCTGGTGCTGGACGTGC------------------------------------------------------------------------------------------------------------------------------------------------------------------------     0.000     0.076     0.076
-------TGCCGCTGGTGCTGGACGTGC------------------------------------------------------------------------------------------------------------------------------------------------------------------------     0.680     1.743     1.920

----                                    C                                                                                                                              AACGCGAGCGGCCAGTCTTCTACTCAC   TCC
    GCGCTGCCGCTGGTGCTGGACGTGCGTGCCCACATTATCA GTCAAAAGGACGGCCGCTCGCACTCGCGGCCGGCGCCAAGGCAGCTGCCGGCGTATCACCGCGTCAAGCGGCTGCCCGGCCACGGCTGCCCGCTGACTGTATACTTGCACTACTACACGCTCACTGCCAGC                           AGC   A
    CGCGACGGCGACCACGACCTGCACGCACGGGTGTAATAGT CAGTTTTCCTGCCGGCGAGCGTGAGCGCCGGCCGCGGTTCCGTCGACGGCCGCATAGTGGCGCAGTTCGCCGACGGGCCGGTGCCGACGGGCGACTGACATATGAACGTGATGATGTGCGAGTGACGGTCG                           TCG   /
GCGT                                    A                                                                                                                              CCACACT--------------------   CCC

----CGCGACGGCGACCACGACCT----------------------------------------------------------------------------------------------------------------------------------------------------------------------------     1.166     0.076     0.226
----------------------GCACGCACGGGTGTAATAG-----------------------------------------------------------------------------------------------------------------------------------------------------------     0.486     0.152     0.301
-------------------------CGCACGGGTGTAATAGT----------------------------------------------------------------------------------------------------------------------------------------------------------     0.000     0.076     0.301
-----------------------CACGCACGGGTGTAATAGT----------------------------------------------------------------------------------------------------------------------------------------------------------     0.049     0.114     0.114
----------------------GCACGCACGGGTGTAATAGT----------------------------------------------------------------------------------------------------------------------------------------------------------    51.045    10.987    14.533
--------------------CTGCACGCACGGGTGTAATAGT----------------------------------------------------------------------------------------------------------------------------------------------------------     0.729     0.114     0.226
-------------------------------------------AGTTTTCCTGCCGGCGAGCG-------------------------------------------------------------------------------------------------------------------------------------     0.049     0.076     0.076
------------------------------------------CAGTTTTCCTGCCGGCGAGCGT------------------------------------------------------------------------------------------------------------------------------------     0.194     0.114     0.038
-------------------------------------------AGTTTTCCTGCCGGCGAGCGT------------------------------------------------------------------------------------------------------------------------------------    10.345     8.714    15.136
--------------------------------------------GTTTTCCTGCCGGCGAGCGT------------------------------------------------------------------------------------------------------------------------------------     0.729     0.189     0.113
----------------------------------------------------------AGCGTGAGCGCCGGCCGCGGT---------------------------------------------------------------------------------------------------------------------     0.146     0.038     0.038
-----------------------------------------------------------GCGTGAGCGCCGGCCGCGGTT--------------------------------------------------------------------------------------------------------------------     0.340     0.568     1.581
------------------------------------------------------------CGTGAGCGCCGGCCGCGGTT--------------------------------------------------------------------------------------------------------------------     0.000     0.076     0.075
---------------------------------------------------------------GAGCGCCGGCCGCGGTTCCGT----------------------------------------------------------------------------------------------------------------     0.680     0.114     0.038
----------------------------------------------------------------------------GTTCCGTCGACGGCCGCAT-----------------------------------------------------------------------------------------------------     0.049     0.038     0.038
---------------------------------------------------------------------------GGTTCCGTCGACGGCCGCAT-----------------------------------------------------------------------------------------------------     0.486     0.227     0.151
--------------------------------------------------------------------------CGGTTCCGTCGACGGCCGCAT-----------------------------------------------------------------------------------------------------     0.583     0.530     0.301
----------------------------------------------------------------------------GTTCCGTCGACGGCCGCATAGT--------------------------------------------------------------------------------------------------     1.311     0.455     0.414
```

```
-----------------------------------------------------------------------------------TCCGTCGACGGCCGCATAGT-----------------------------------------------------------------------------------------------------------      0.680      0.076      0.075
--------------------------------------------------------------------------------GGTTCCGTCGACGGCCGCATAGT-----------------------------------------------------------------------------------------------------------      0.097      0.076      0.075
------------------------------------------------------------------------------------CCGTCGACGGCCGCATAGT-----------------------------------------------------------------------------------------------------------      0.631      0.114      0.114
--------------------------------------------------------------------------------------GTCGACGGCCGCATAGTGGC--------------------------------------------------------------------------------------------------------      0.049      0.871      0.339
-------------------------------------------------------------------------------------------CGGCCGCATAGTGGCGCAGT---------------------------------------------------------------------------------------------------      0.146      0.114      0.038
-----------------------------------------------------------------------------------------GACGGCCGCATAGTGGCGCAGTT--------------------------------------------------------------------------------------------------      0.000      0.038      0.038
-------------------------------------------------------------------------------------------CGGCCGCATAGTGGCGCAGTT--------------------------------------------------------------------------------------------------      1.166      5.153      2.447
----------------------------------------------------------------------------------------------------------GCAGTTCGCCGACGGGCCGGT-----------------------------------------------------------------------------------      1.846      0.644      0.414
-----------------------------------------------------------------------------------------------------------CAGTTCGCCGACGGGCCGGT-----------------------------------------------------------------------------------      0.194      0.076      0.076
---------------------------------------------------------------------------------------------------------CGCAGTTCGCCGACGGGCCGGT-----------------------------------------------------------------------------------      0.097      0.152      0.113
-----------------------------------------------------------------------------------------------------------------------GGGCCGGTGCCGACGGGCGAC----------------------------------------------------------------------      0.000      0.038      0.038
----------------------------------------------------------------------------------------------------------------------CGGGCCGGTGCCGACGGGCGAC----------------------------------------------------------------------      0.000      0.038      0.038
-----------------------------------------------------------------------------------------------------------------------GGGCCGGTGCCGACGGGCGACT---------------------------------------------------------------------      0.534      0.796      1.092
------------------------------------------------------------------------------------------------------------------------GGCCGGTGCCGACGGGCGACT---------------------------------------------------------------------      3.254      2.387      4.480
----------------------------------------------------------------------------------------------------------------------CGGGCCGGTGCCGACGGGCGACT---------------------------------------------------------------------      0.049      0.038      0.489
-------------------------------------------------------------------------------------------------------------------------GCCGGTGCCGACGGGCGACT---------------------------------------------------------------------      0.194      0.303      0.264
----------------------------------------------------------------------------------------------------------------------------GGTGCCGACGGGCGACTGAC------------------------------------------------------------------      0.000      0.038      0.038
-----------------------------------------------------------------------------------------------------------------------------GTGCCGACGGGCGACTGACAT----------------------------------------------------------------      0.146      0.227      0.264
-------------------------------------------------------------------------------------------------------------------------------GCCGACGGGCGACTGACAT----------------------------------------------------------------      0.000      0.189      0.339
----------------------------------------------------------------------------------------------------------------------------GGTGCCGACGGGCGACTGACAT----------------------------------------------------------------      0.194      0.189      0.189
-------------------------------------------------------------------------------------------------------------------------------GCCGACGGGCGACTGACATA---------------------------------------------------------------      0.631      0.076      0.678
-------------------------------------------------------------------------------------------------------------------------------GCCGACGGGCGACTGACATAT--------------------------------------------------------------      85.091     46.448     55.610 miR_t31
-----------------------------------------------------------------------------------------------------------------------------GTGCCGACGGGCGACTGACATAT--------------------------------------------------------------      0.000      0.038      0.075
--------------------------------------------------------------------------------------------------------------------------------CCGACGGGCGACTGACATAT--------------------------------------------------------------      0.243      0.303      0.301
---------------------------------------------------------------------------------------------------------------------------------CGACGGGCGACTGACATAT--------------------------------------------------------------      0.097      0.265      0.414
----------------------------------------------------------------------------------------------------------------------------------GACGGGCGACTGACATAT--------------------------------------------------------------      0.340      1.932      1.506
----------------------------------------------------------------------------------------------------------------------------------GACGGGCGACTGACATATGA------------------------------------------------------------      0.000      0.493      1.092
----------------------------------------------------------------------------------------------------------------------------------GACGGGCGACTGACATATGAA-----------------------------------------------------------      0.146      2.046      4.405
--------------------------------------------------------------------------------------------------------------------------------------GGCGACTGACATATGAACGT--------------------------------------------------------      0.243      0.189      0.038
------------------------------------------------------------------------------------------------------------------------------------CGGGCGACTGACATATGAACGT--------------------------------------------------------      0.097      0.076      0.076
-------------------------------------------------------------------------------------------------------------------------------------GGGCGACTGACATATGAACGT--------------------------------------------------------      0.340      1.061      1.657
----------------------------------------------------------------------------------------------------------------------------------------CGACTGACATATGAACGTGAT-----------------------------------------------------      0.826      1.440      3.953
--------------------------------------------------------------------------------------------------------------------------------------------TGACATATGAACGTGATGAT--------------------------------------------------      0.826      0.076      0.113
-------------------------------------------------------------------------------------------------------------------------------------------CTGACATATGAACGTGATGAT--------------------------------------------------      0.923      0.758      0.791
-----------------------------------------------------------------------------------------------------------------------------------------GACTGACATATGAACGTGATGAT--------------------------------------------------      0.049      0.189      0.189
---------------------------------------------------------------------------------------------------------------------------------------------GACATATGAACGTGATGAT--------------------------------------------------      0.097      0.227      0.226
----------------------------------------------------------------------------------------------------------------------------------------------------TGAACGTGATGATGTGCGAGT-----------------------------------------      0.486      0.038      0.075
-----------------------------------------------------------------------------------------------------------------------------------------------------GAACGTGATGATGTGCGAGT-----------------------------------------      1.748      0.758      0.377
--------------------------------------------------------------------------------------------------------------------------------------------------------CGTGATGATGTGCGAGTGAC--------------------------------------      0.000      0.038      0.038
-------------------------------------------------------------------------------------------------------------------------------------------------------ACGTGATGATGTGCGAGTGAC--------------------------------------      0.000      0.038      0.075
--------------------------------------------------------------------------------------------------------------------------------------------------------CGTGATGATGTGCGAGTGACG-------------------------------------      0.000      0.076      0.076
-----------------------------------------------------------------------------------------------------------------------------------------------------------GATGATGTGCGAGTGACGGT-----------------------------------      5.197      2.917      3.840
----------------------------------------------------------------------------------------------------------------------------------------------------------TGATGATGTGCGAGTGACGGT-----------------------------------      0.583      0.530      0.715

miRNA: miR_t34,miR_t32 chromosome_7 Start: 2591450 End: 2591850 Condition: HS-N
-------------------------------------------------------------------------------------------------------------------------------------------TGCATGGCTCGCTGTCAGAGGC-------------------------------------------------------      0.243      1.061      0.038
-------------------------------------------------------------------------------------------------------------------------------------------TGCATGGCTCGCTGTCAGAGGCTTCCAGC------------------------------------------------      0.049      1.061      0.602
-------------------------------------------------------------------------------------------------------------------------------------------TGCATGGCTCGCTGTCAGAGG--------------------------------------------------------      0.000      0.227      0.227
-------------------------------------------------------------------------------------------------------------------TGGGCTTTCAGGCTTTCAGCTTCCT----------------------------------------------------------------------------      0.194      0.076      0.076
-------------------------------------------------------------------------------------------------------------------TGGGCTTTCAGGCTTTCAGCTTC------------------------------------------------------------------------------      0.000      0.038      0.038
-------------------------------------------------------------------------------------------------------------------TGGGCTTTCAGGCTTTCAGC---------------------------------------------------------------------------------      0.000      0.038      0.038
-------------------------------------------------------------------------------------------------------------------TGGGCTTTCAGGCTTTCAGCTTCC-----------------------------------------------------------------------------      0.243      1.061      0.113
-------------------------------------------------------------------------------------------------------------TGCAGGTGGGCTTTCAGGCT---------------------------------------------------------------------------------------      0.000      0.038      0.038
-------------------------------------------------------------------------------------------------------------TGCAGGTGGGCTTTCAGGCTTTC------------------------------------------------------------------------------------      0.097      0.833      0.489
---------------------------------------------------------------------------------TGCAAACAGGGTCGGCCCCAGT-----------------------------------------------------------------------------------------------------------------      0.000      0.038      0.038
-------------------------------------------------------------------------------TTTGCAAACAGGGTCGGCCCC--------------------------------------------------------------------------------------------------------------------      0.243      0.114      0.264
------------------------------------------------------------------------GGCGAGCTTTGCAAACAGG-----------------------------------------------------------------------------------------------------------------------------      0.000      0.038      0.038
-----------------------------------------------------------------------CGGCGAGCTTTGCAAACAGGG----------------------------------------------------------------------------------------------------------------------------      0.340      0.265      0.264
-----------------------------------------------------------------------CGGCGAGCTTTGCAAACAGG-----------------------------------------------------------------------------------------------------------------------------      0.000      0.076      0.076
----------------------------------------------------------------------TCGGCGAGCTTTGCAAA---------------------------------------------------------------------------------------------------------------------------------      0.000      0.038      0.038
----------------------------------------------------------------------TCGGCGAGCTTTGCAAACAGG-----------------------------------------------------------------------------------------------------------------------------      10.685     8.827      8.886
----------------------------------------------------------------------TCGGCGAGCTTTGCAAACAG------------------------------------------------------------------------------------------------------------------------------      1.068      4.054      7.229
----------------------------------------------------------------------TCGGCGAGCTTTGCAAACAGGG----------------------------------------------------------------------------------------------------------------------------      0.340      0.682      0.188
---------------------------------------------------------------------CTCGGCGAGCTTTGCAAA---------------------------------------------------------------------------------------------------------------------------------      0.000      0.038      0.038
--------------------------------------------------------------------TCTCGGCGAGCTTTGCAAA---------------------------------------------------------------------------------------------------------------------------------      0.000      0.227      0.038
--------------------------------------------------------------------TCTCGGCGAGCTTTGCAAAC--------------------------------------------------------------------------------------------------------------------------------      0.534      1.023      0.904
--------------------------------------------------------------------TCTCGGCGAGCTTTGCAAACA-------------------------------------------------------------------------------------------------------------------------------      2.234      0.796      1.242
--------------------------------------------------------------------TCTCGGCGAGCTTTGCAAACAG------------------------------------------------------------------------------------------------------------------------------      1.603      3.902      3.577
--------------------------------------------------------------------TCTCGGCGAGCTTTGCA-----------------------------------------------------------------------------------------------------------------------------------      0.000      0.076      0.076
--------------------------------------------------------------------TCTCGGCGAGCTTTGCAAACAGG-----------------------------------------------------------------------------------------------------------------------------      1.943      2.008      0.075
----------------------------------------------------------------TCCCTCTCGGCGAGCTTTGC------------------------------------------------------------------------------------------------------------------------------------      0.000      0.114      0.114
----------------------------------------------------------------TCCCTCTCGGCGAGCTTTGCAA----------------------------------------------------------------------------------------------------------------------------------      0.049      0.038      0.038
--------------------------------------------------------------TGTCCCTCTCGGCGAGCTTTGC------------------------------------------------------------------------------------------------------------------------------------      0.049      0.114      0.114
----------------------------------------------------------TCAATGTCCCTCTCGGCGAGC-----------------------------------------------------------------------------------------------------------------------------------------      0.874      1.553      1.619
----------------------------------------------------AGCGCATCAATGTCCCTCTCG-----------------------------------------------------------------------------------------------------------------------------------------------      0.049      0.076      0.076
---------------------------------------------------CAGCGCATCAATGTCCCTCTC------------------------------------------------------------------------------------------------------------------------------------------------      0.049      0.038      0.038
-------------------------------------TACCCCGCTTGGTGCAGCGC---------------------------------------------------------------------------------------------------------------------------------------------------------------      0.000      0.114      0.038
----------------------------------TCATACCCCGCTTGGTGCAG------------------------------------------------------------------------------------------------------------------------------------------------------------------      0.583      0.152      0.264
----------------------------------TCATACCCCGCTTGGTGCAGC-----------------------------------------------------------------------------------------------------------------------------------------------------------------      0.194      0.644      0.452
----------------------------TCCCCGTCATACCCCGCTTGGT----------------------------------------------------------------------------------------------------------------------------------------------------------------------      0.000      0.076      0.076
----------------------------TCCCCGTCATACCCCGCTTGG-----------------------------------------------------------------------------------------------------------------------------------------------------------------------      3.303      0.455      0.301
----TCCCTGGACGCGAAGCGGAGC-----------------------------------------------------------------------------------------------------------------------------------------------------------------------------------------------      0.000      0.076      0.076

GCTTTCCCTGGACGCGA         T                              A             C               C               CG         G        CAG                             AGAGGCTTCCA       C      A     TGA       AC        GA  T--  AT
                 AGCGGAGCG ATCCCCGTCATACCCCGCTTGGTGCAGCGC TCAATGTCCCTCT GGCGAGCTTTGCAAA AGGGTCGGCCCCAGT  CCGCTGCAG TGGGCTTT   GCTTTCAGCTTCCTGCATGGCTCGCTGTC           GCGCCTT CTTGCC CCCGA   TACGCG  TCGGATT  GT   CG  T
                 TCGCCTCGT TAGGGGCAGTATGGGGCGAACCACGTCGCG AGTTATAGGGAGA CCGCTCGAAGCGTTT TCTCAGCCGGGGTCA  GGCGACGTC ACCCGAAG   CGAAGGTCGAAGGACGTGCCGAGCGGCAG           CGCGGAA GAACGG GGGCT   ATGCGT  AGCCTAA  CA   GC  /
---------------G          C                              C             A               A                AA        G         CA-                             GCT--------       C      C     ---       CA        G-  TAT  GG

--------------------CCTCGTCTAGGGGCAGTAT-----------------------------------------------------------------------------------------------------------------------------------------------------------------------------      0.049      0.114      0.075
--------------------------------GCAGTATGGGGCGAACCACGT-------------------------------------------------------------------------------------------------------------------------------------------------------------------      4.905      6.479      2.523
---------------------------------------GGGGCGAACCACGTCGCGCAGT-----------------------------------------------------------------------------------------------------------------------------------------------------------      0.049      0.076      0.038
-----------------------------------------GGCGAACCACGTCGCGCAGT-----------------------------------------------------------------------------------------------------------------------------------------------------------      0.000      0.038      0.038
---------------------------------------GGGGCGAACCACGTCGCGCAGTT----------------------------------------------------------------------------------------------------------------------------------------------------------      0.000      0.189      0.189
--------------------------------------TGGGGCGAACCACGTCGCGCAGTT----------------------------------------------------------------------------------------------------------------------------------------------------------      0.000      0.114      0.114
------------------------------------------GCGAACCACGTCGCGCAGTT----------------------------------------------------------------------------------------------------------------------------------------------------------      0.049      0.341      0.341
-----------------------------------------GGCGAACCACGTCGCGCAGTT----------------------------------------------------------------------------------------------------------------------------------------------------------      0.437      1.212      0.339
-------------------------------------------CGAACCACGTCGCGCAGTT----------------------------------------------------------------------------------------------------------------------------------------------------------      0.000      0.417      0.377
-------------------------------------------CGAACCACGTCGCGCAGTTA---------------------------------------------------------------------------------------------------------------------------------------------------------      0.000      0.114      0.151
--------------------------------------------GAACCACGTCGCGCAGTTAT--------------------------------------------------------------------------------------------------------------------------------------------------------      0.049      0.114      0.114
-------------------------------------------CGAACCACGTCGCGCAGTTAT--------------------------------------------------------------------------------------------------------------------------------------------------------      3.885      32.696     23.042 miR_t34
------------------------------------------GCGAACCACGTCGCGCAGTTAT--------------------------------------------------------------------------------------------------------------------------------------------------------      0.000      0.038      0.038
-----------------------------------------------CCACGTCGCGCAGTTATAGGG----------------------------------------------------------------------------------------------------------------------------------------------------      0.000      0.038      0.075
-----------------------------------------------CCACGTCGCGCAGTTATAGGGA---------------------------------------------------------------------------------------------------------------------------------------------------      0.000      0.038      0.038
--------------------------------------------------CGTCGCGCAGTTATAGGGAGA-------------------------------------------------------------------------------------------------------------------------------------------------      0.000      0.038      0.038
---------------------------------------------------GTCGCGCAGTTATAGGGAGAA------------------------------------------------------------------------------------------------------------------------------------------------      0.243      0.152      0.113
--------------------------------------------------CGTCGCGCAGTTATAGGGAGAA------------------------------------------------------------------------------------------------------------------------------------------------      0.291      0.796      0.414
--------------------------------------------------------GCAGTTATAGGGAGAACCGCT-------------------------------------------------------------------------------------------------------------------------------------------      0.194      0.076      0.076
-------------------------------------------------------CGCAGTTATAGGGAGAACCGCT-------------------------------------------------------------------------------------------------------------------------------------------      0.000      0.038      0.038
--------------------------------------------------------------ATAGGGAGAACCGCTCGAAGCGT-----------------------------------------------------------------------------------------------------------------------------------      0.000      0.189      0.189
----------------------------------------------------------------AGGGAGAACCGCTCGAAGCGT-----------------------------------------------------------------------------------------------------------------------------------      0.097      0.379      0.113
-----------------------------------------------------------------GGGAGAACCGCTCGAAGCGTT----------------------------------------------------------------------------------------------------------------------------------      0.049      0.114      0.038
------------------------------------------------------------------GGAGAACCGCTCGAAGCGTTT---------------------------------------------------------------------------------------------------------------------------------      0.340      0.189      0.038
------------------------------------------------------------------------------GAAGCGTTTATCTCAGCCGGGG--------------------------------------------------------------------------------------------------------------------      0.000      0.038      0.038
-----------------------------------------------------------------------------CGAAGCGTTTATCTCAGCCGGGG--------------------------------------------------------------------------------------------------------------------      0.000      0.038      0.038
-----------------------------------------------------------------------------CGAAGCGTTTATCTCAGCCGGGGT-------------------------------------------------------------------------------------------------------------------      0.777      0.833      0.038
----------------------------------------------------------------------------------CGTTTATCTCAGCCGGGGT-------------------------------------------------------------------------------------------------------------------      0.049      0.038      0.038
--------------------------------------------------------------------------------AGCGTTTATCTCAGCCGGGGT-------------------------------------------------------------------------------------------------------------------      0.631      0.038      0.075
---------------------------------------------------------------------------------GCGTTTATCTCAGCCGGGGT-------------------------------------------------------------------------------------------------------------------      6.945      1.288      1.167
------------------------------------------------------------------------------GAAGCGTTTATCTCAGCCGGGGT-------------------------------------------------------------------------------------------------------------------      2.526      1.137      0.979
--------------------------------------------------------------------------------------------AGCCGGGGTCAAAGGCGA----------------------------------------------------------------------------------------------------------      0.000      0.038      0.038
----------------------------------------------------------------------------------------TCTCAGCCGGGGTCAAAGGCGA----------------------------------------------------------------------------------------------------------      0.000      0.038      0.038
----------------------------------------------------------------------------------------TCTCAGCCGGGGTCAAAGGCGACG--------------------------------------------------------------------------------------------------------      0.000      0.038      0.038
---------------------------------------------------------------------------------------------GCCGGGGTCAAAGGCGACGT-------------------------------------------------------------------------------------------------------      0.291      1.326      0.904
-------------------------------------------------------------------------------------------CAGCCGGGGTCAAAGGCGACGT-------------------------------------------------------------------------------------------------------      0.243      0.985      2.598
--------------------------------------------------------------------------------------------AGCCGGGGTCAAAGGCGACGT-------------------------------------------------------------------------------------------------------      0.729      1.819      1.883
----------------------------------------------------------------------------------------TCTCAGCCGGGGTCAAAGGCGACGT-------------------------------------------------------------------------------------------------------      0.000      0.038      0.377
--------------------------------------------------------------------------------------------------------------CGTCGACCCGAAGCA-CGAAGGT-----------------------------------------------------------------------------------      0.049      0.076      0.038
-----------------------------------------------------------------------------------------------------------------------GAAGCA-CGAAGGTCGAAGGAC---------------------------------------------------------------------------      0.000      0.076      0.076
-----------------------------------------------------------------------------------------------------------------------GAAGCA-CGAAGGTCGAAGGACGT-------------------------------------------------------------------------      0.000      0.038      0.038
-------------------------------------------------------------------------------------------------------------------------AGCA-CGAAGGTCGAAGGACGT-------------------------------------------------------------------------      0.777      1.364      0.715
--------------------------------------------------------------------------------------------------------------------------GCA-CGAAGGTCGAAGGACGT-------------------------------------------------------------------------      0.194      0.076      0.151
-----------------------------------------------------------------------------------------------------------------------------------------GGACGTGCCGAGCGGCAGGC-----------------------------------------------------------      0.000      0.038      0.038
------------------------------------------------------------------------------------------------------------------------------------------GACGTGCCGAGCGGCAGGCT----------------------------------------------------------      0.243      0.682      0.038
-----------------------------------------------------------------------------------------------------------------------------------------GGACGTGCCGAGCGGCAGGCT----------------------------------------------------------      0.777      7.994      7.380
--------------------------------------------------------------------------------------------------------------------------------------------CGTGCCGAGCGGCAGGCT----------------------------------------------------------      0.097      0.606      0.151
-------------------------------------------------------------------------------------------------------------------------------------CGAAGGACGTGCCGAGCGGCAGGCT----------------------------------------------------------      0.000      0.076      0.076
----------------------------------------------------------------------------------------------------------------------------------------AGGACGTGCCGAGCGGCAGGCT----------------------------------------------------------      0.000      0.114      0.114
-------------------------------------------------------------------------------------------------------------------------------------------------CGAGCGGCAGGCT--------CGCGGAAC------------------------------------------      0.000      0.038      0.038
-------------------------------------------------------------------------------------------------------------------------------------------------------------T--------CGCGGAACGAACGGCGGGCT------------------------------      0.243      0.038      0.113
----------------------------------------------------------------------------------------------------------------------------------------------------------------------CGCGGAACGAACGGCGGGCT------------------------------      0.146      0.189      0.339
----------------------------------------------------------------------------------------------------------------------------------------------------------------------CGCGGAACGAACGGCGGGCT---A--------------------------      0.049      0.265      0.414
--------------------------------------------------------------------------------------------------------------------------------------------------------------------------GAACGAACGGCGGGCT---AT-------------------------      0.000      0.568      0.339
-----------------------------------------------------------------------------------------------------------------------------------------------------------------------GCGGAACGAACGGCGGGCT---AT-------------------------      9.762      19.436     39.684
-------------------------------------------------------------------------------------------------------------------------------------------------------------------------GGAACGAACGGCGGGCT---AT-------------------------      0.097      1.288      1.845
------------------------------------------------------------------------------------------------------------------------------------------------------------CT--------CGCGGAACGAACGGCGGGCT---AT-------------------------      0.000      0.038      0.038
------------------------------------------------------------------------------------------------------------------------------------------------------------------------CGGAACGAACGGCGGGCT---AT-------------------------      0.097      2.955      4.518
----------------------------------------------------------------------------------------------------------------------------------------------------------------------CGCGGAACGAACGGCGGGCT---AT-------------------------      9.374      94.564     53.276 miR_t32
-------------------------------------------------------------------------------------------------------------------------------------------------------------T--------CGCGGAACGAACGGCGGGCT---AT-------------------------      1.117      7.388      2.033

miRNA: miR_t35 chromosome_8 Start: 347684 End: 348102 Condition: HS-N
--------------------------------------------------CGATTACGACGAGGACTTCG-----      0.000      0.076      0.076
-----------------------------------------------------TTACGACGAGGACTTCGAGAC-      0.000      0.076      0.188
------------------------------------------------------TACGACGAGGACTTCGAG---      1.020      0.720      2.673
------------------------------------------------------TACGACGAGGACTTCGAGAC-      0.729      3.826      22.779
------------------------------------------------------TACGACGAGGACTTCGAGA--      2.186      2.463      9.450
------------------------------------------------------TACGACGAGGACTTCGA----      1.263      1.932      7.869
-------------------------------------------------------ACGACGAGGACTTCGAG---      0.000      0.114      0.038
-------------------------------------------------------ACGACGAGGACTTCGAGAC-      0.000      0.038      2.297

--------------ATG  -     AC                    C  C                     AC
                 GC GCGCA  GGTACCGAGGACGAAGAGCT CT GATTACGACGAGGACTTCGAG  G
                 CG CGCGT  TCATGGCTCCTGCTTCTCGA GA CTAATGCTGCTCCTGAAGCTC  /
GAATCGTGAACGGGAGA  A     C-                    A  A                     TT

--------------------------------------------CGAAGAACTAATGCTGCTCCT----------      0.146      0.227      0.226
-----------------------------------CCTGCTTCTCGAAGAACTAAT-------------------      1.166      0.720      1.355
```

```
-----------------------------------CCTGCTTCTCGAAGAACTAA--------------------      0.097      0.076      0.076
-------------------------------GGCTCCTGCTTCTCGAAGAACTA---------------------      0.000      0.038      0.038
-----------------------------------CCTGCTTCTCGAAGAACT----------------------      0.049      0.038      0.038
-------------------------------GGCTCCTGCTTCTCGAAGAACT----------------------    442.602     91.836    143.713 miR_t35
--------------------------------GCTCCTGCTTCTCGAAGAACT----------------------    304.960     68.725    103.050
------------------------------TGGCTCCTGCTTCTCGAAGAACT----------------------      3.254      0.606      0.678
---------------------------------CTCCTGCTTCTCGAAGAACT----------------------      0.097      0.038      0.075
--------------------------------GCTCCTGCTTCTCGAAGAAC-----------------------      2.186      0.493      1.017
-------------------------------GGCTCCTGCTTCTCGAAGAAC-----------------------      2.283      0.379      0.678
-------------------------------GGCTCCTGCTTCTCGAAGAA------------------------     11.608      0.644      1.092
--------------------------------GCTCCTGCTTCTCGAAGAA------------------------      3.448      0.720      1.205
----------------------CGTC-TCATGGCTCCTGCTTCT-------------------------------      0.000      0.038      0.038
----------CGGGAGACGACGCGTC-TCAT--------------------------------------------      0.000      0.076      0.076
------TGAACGGGAGACGACGCGTC-T-----------------------------------------------     37.883      0.796     23.682
-----GTGAACGGGAGACGACGCGTC-T-----------------------------------------------      3.837      0.341      1.017
-------GAACGGGAGACGACGCGTC-T-----------------------------------------------    335.169      6.516     93.073
---TCGTGAACGGGAGACGACGCGTC-T-----------------------------------------------      0.097      0.038      0.075
----CGTGAACGGGAGACGACGCGTC-T-----------------------------------------------      4.711      0.644      0.941
--------AACGGGAGACGACGCGTC-T-----------------------------------------------      1.263      0.076      0.301
----CGTGAACGGGAGACGACGCGT--------------------------------------------------      0.631      0.455      3.389
```

```
miRNA: miR_t52,miR_t79 chromosome_2 Start: 3047193 End: 3047455 Condition: HS-N
--------------TGA---TGGATGAGCAGGAGGTC---------------------------------------------------------------------------------------------------------      0.000      0.038      0.113
--------------TGA---TGGATGAGCAGGAGGTCC--------------------------------------------------------------------------------------------------------      0.049      0.189      0.189
--------------TGA---TGGATGAGCAGGAGG-----------------------------------------------------------------------------------------------------------      0.000      0.038      0.038
----------------A---TGGATGAGCAGGAGGTCCAGC-----------------------------------------------------------------------------------------------------      0.000      0.038      0.188
----------------A---TGGATGAGCAGGAGGTCCAG------------------------------------------------------------------------------------------------------      0.049      0.152      0.152
--------------------TGGATGAGCAGGAGGTC---------------------------------------------------------------------------------------------------------      0.000      0.038      0.226
--------------------TGGATGAGCAGGAGGTCCAGC-----------------------------------------------------------------------------------------------------      7.868      1.326      2.033
--------------------TGGATGAGCAGGAGGTCCAG------------------------------------------------------------------------------------------------------      2.623      0.076      0.076
----------------------GATGAGCAGGAGGTCCAGCAG---------------------------------------------------------------------------------------------------      0.049      0.417      1.619
------------------------TGAGCAGGAGGTCCAGCAGC--------------------------------------------------------------------------------------------------      0.000      0.038      0.188
-----------------------------------TCCAGCAGCACGCCGCAGCAG--------------------------------------------------------------------------------------      2.428      1.781      0.565
-----------------------------------TCCAGCAGCACGCCGCAGCAGG-------------------------------------------------------------------------------------      1.554      0.644      0.188 miR_t79*
--------------------------------------AGCAGCACGCCGCAGCAGGCA-----------------------------------------------------------------------------------      0.000      0.038      0.038
------------------------------------------------------AGGCAGCTGTGGGCCTACTGG-------------------------------------------------------------------      0.000      0.114      0.114
--------------------------------------------------------GCAGCTGTGGGCCTACTGGAG-----------------------------------------------------------------      0.146      0.530      0.530
---------------------------------------------------------CAGCTGTGGGCCTACTGGAGGCG--------------------------------------------------------------      0.194      2.008      0.377
---------------------------------------------------------CAGCTGTGGGCCTACTGGAG-----------------------------------------------------------------     38.806     31.635     15.964
---------------------------------------------------------CAGCTGTGGGCCTACTGGAGGC---------------------------------------------------------------      0.243      4.243      1.017
---------------------------------------------------------CAGCTGTGGGCCTACTGGAGG----------------------------------------------------------------      9.616      9.320      3.539
---------------------------------------------------------CAGCTGTGGGCCTACTGGAGGCGC-------------------------------------------------------------      0.000      0.114      0.114
----------------------------------------------------------AGCTGTGGGCCTACTGGAGGCG--------------------------------------------------------------      0.000      0.114      0.114
----------------------------------------------------------AGCTGTGGGCCTACTGGAGGC---------------------------------------------------------------      0.000      0.493      0.602
----------------------------------------------------------AGCTGTGGGCCTACTGGAG-----------------------------------------------------------------      0.097      0.038      0.113
-------------------------------------------------------------TGTGGGCCTACTGGAGGCGCC------------------------------------------------------------      3.497      0.379      0.226
-------------------------------------------------------------TGTGGGCCTACTGGAGGCG--------------------------------------------------------------      3.351      1.288      0.791
-------------------------------------------------------------TGTGGGCCTACTGGAGG----------------------------------------------------------------      1.651      0.189      0.339
-------------------------------------------------------------TGTGGGCCTACTGGAGGCGC-------------------------------------------------------------      3.448      2.463      1.958
-------------------------------------------------------------TGTGGGCCTACTGGAGGC---------------------------------------------------------------      0.340      0.493      0.866
---------------------------------------------------------------TGGGCCTACTGGAGGCG--------------------------------------------------------------      0.146      0.076      0.038
---------------------------------------------------------------TGGGCCTACTGGAGGCGC-------------------------------------------------------------      0.389      0.038      0.075
---------------------------------------------------------------------TACTGGAGGCGCCGCAGCGC-----------------------------------------------------      0.049      0.189      0.113
---------------------------------------------------------------------TACTGGAGGCGCCGCAGCGCA----------------------------------------------------      0.000      0.152      0.075
-----------------------------------------------------------------------------------------------ATCGATTGCCAGCGCCACAGC--------------------------      0.000      0.114      0.114
------------------------------------------------------------------------------------------------TCGATTGCCAGCGCCACAGC--------------------------      1.166      2.879      3.125
------------------------------------------------------------------------------------------------TCGATTGCCAGCGCCACAGCC-------------------------      0.097      0.038      0.075
------------------------------------------------------------------------------------------------TCGATTGCCAGCGCCAC-----------------------------      0.000      0.038      0.038

----------  C    ---    T                                                                                  C          A-  ACGCCGCCGAAC  A   A
          GC GTGA   TGGA GAGCAGGAGGTCCAGCAGCACGCCGCAGCAGGCAGCTGTGGGCCTACTGGAGGCGCCGCAGCGCAACAACATCGATTGCCAG GCCACAGCCA  GC            GC AGG A
          CG CGCT   ACCT CTCGTCCTCCAGGTCGTCGTGCGGCGTCGTCCGTCGACACCCGGATGACCTCCGCGGCGTCGCGTTGTTGTGGCTAACGGTT CGGTGTCGGT  CG            CG TCC C
CCGCCGCCGC  C    GCC    C                                                                                  T          CA  CTCA--------  C   C

--------------------------------------------------------------------------------------------------CTAACGGTTTCGGTGTCGGT------------------------      0.194      0.909      1.393
-----------------------------------------------------------------------------------------------TGGCTAACGGTTTCGGTGTCGGT------------------------      0.000      0.038      0.038
------------------------------------------------------------------------------------------------GGCTAACGGTTTCGGTGTCGGT------------------------      0.486      0.985      0.301
-------------------------------------------------------------------------------------------------GCTAACGGTTTCGGTGTCGGT------------------------      3.448      2.917      3.502
-----------------------------------------------------------------------------------------------TGGCTAACGGTTTCGGTGT----------------------------      0.000      0.189      0.189
----------------------------------------------------------------------------------------------GTGGCTAACGGTTTCGGTGT----------------------------      0.486      0.152      0.075
---------------------------------------------------------------------------------------------TGTGGCTAACGGTTTCGGTGT----------------------------      0.486      0.076      0.038
------------------------------------------------------------------------------------------TGTTGTGGCTAACGGTTTCGGT------------------------------      0.631      0.568      0.866
-----------------------------------------------------------------------------------------TTGTTGTGGCTAACGGTTTCGGT------------------------------      0.097      0.152      0.152
-------------------------------------------------------------------------------------------GTTGTGGCTAACGGTTTCGGT------------------------------     34.338      6.554      9.827
------------------------------------------------------------------------------------------TGTTGTGGCTAACGGTTTCGG-------------------------------      0.097      0.038      0.038
-------------------------------------------------------------------------------------------GTTGTGGCTAACGGTTTCGG-------------------------------      1.166      0.189      0.527
----------------------------------------------------------------------------------------GTTGTTGTGGCTAACGGTTT----------------------------------      0.049      0.038      0.038
---------------------------------------------------------------------------------------CGTTGTTGTGGCTAACGGTTT----------------------------------      0.049      0.227      0.075
-------------------------------------------------------------------------------------CGCGTTGTTGTGGCTAACGGT------------------------------------      0.243      0.303      0.151
----------------------------------------------------------------------------------CGTCGCGTTGTTGTGGCTAAC---------------------------------------      0.049      0.038      0.038
-------------------------------------------------------------------------------CGGCGTCGCGTTGTTGTGGCTAA----------------------------------------      0.000      0.152      0.152
------------------------------------------------------------------------------GCGGCGTCGCGTTGTTGTGGCT------------------------------------------      2.963      1.250      0.075
----------------------------------------------------------------------------CCGCGGCGTCGCGTTGTTGTGGCT------------------------------------------      0.000      0.076      0.076
---------------------------------------------------------------------------TCCGCGGCGTCGCGTTGTTGTGGCT------------------------------------------      0.000      0.038      0.038
--------------------------------------------------------------------------------GGCGTCGCGTTGTTGTGGCT------------------------------------------      8.597      1.061      0.715
-------------------------------------------------------------------------------CGGCGTCGCGTTGTTGTGGCT------------------------------------------    114.378    472.288    363.105 miR_t52
-------------------------------------------------------------------------------CGGCGTCGCGTTGTTGTGGC-------------------------------------------      0.826      0.530      0.301
-------------------------------------------------------------------------------CGGCGTCGCGTTGTTGTGG--------------------------------------------      0.146      0.530      0.339
---------------------------------------------------------------------------TCCGCGGCGTCGCGTTGTTGT----------------------------------------------      0.631      0.189      0.113
------------------------------------------------------------------------------GCGGCGTCGCGTTGTTGT----------------------------------------------      0.049      0.265      0.038
-----------------------------------------------------------------------------CGCGGCGTCGCGTTGTTGT----------------------------------------------      4.857      2.084      1.506
-------------------------------------------------------------------------------CGGCGTCGCGTTGTTGT----------------------------------------------      0.097      0.303      0.264
----------------------------------------------------------------------------CCGCGGCGTCGCGTTGTTGT----------------------------------------------      2.137      1.440      0.527
-----------------------------------------------------------------------GACCTCCGCGGCGTCGCGTT---------------------------------------------------      0.000      0.038      0.038
----------------------------------------------------------------------TGACCTCCGCGGCGTCGCGTT---------------------------------------------------      0.097      0.189      0.151
---------------------------------------------------------GTCGACACCCGGATGACCTCC----------------------------------------------------------------      0.000      0.038      0.038
--------------------------------------------------------CGTCGACACCCGGATGACCT------------------------------------------------------------------      0.097      0.038      0.226
-------------------------------------------------------CCGTCGACACCCGGATGACCT------------------------------------------------------------------      1.117      0.833      0.339
--------------------------------------TCGTCGTGCGGCGTCGTCCGT-----------------------------------------------------------------------------------      0.049      0.038      0.038
------------------------------------GGTCGTCGTGCGGCGTCGT---------------------------------------------------------------------------------------      3.060      2.917      1.167
----------------------------------CAGGTCGTCGTGCGGCGTCGT---------------------------------------------------------------------------------------      5.780      3.296      0.866
--------------------------------TCCAGGTCGTCGTGCGGCGTCGT---------------------------------------------------------------------------------------      2.866      3.523      0.301
-----------------------------------AGGTCGTCGTGCGGCGTCGT---------------------------------------------------------------------------------------      2.866      0.909      0.941
---------------------------------CCAGGTCGTCGTGCGGCGTCGT---------------------------------------------------------------------------------------    340.366    104.831      4.179 miR_t79
----------------------------------CAGGTCGTCGTGCGGCGTCG----------------------------------------------------------------------------------------      0.000      0.038      0.038
---------------------------------CCAGGTCGTCGTGCGGCGTCG----------------------------------------------------------------------------------------      2.234      0.076      0.076
---------------------------------CCAGGTCGTCGTGCGGCGTC-----------------------------------------------------------------------------------------      0.000      0.076      0.076
--------------------------------TCCAGGTCGTCGTGCGGCGT------------------------------------------------------------------------------------------      0.146      0.038      0.226
---------------------------------CCAGGTCGTCGTGCGGCGT------------------------------------------------------------------------------------------      0.340      0.189      0.188
------------------------------CCTCCAGGTCGTCGTGCGGCGT------------------------------------------------------------------------------------------      0.146      0.871      1.393
-----------------------------TCCTCCAGGTCGTCGTGCGGCGT------------------------------------------------------------------------------------------      0.000      0.076      0.076
-------------------------------CTCCAGGTCGTCGTGCGGCGT------------------------------------------------------------------------------------------      0.000      0.265      0.075
------------------------------CCTCCAGGTCGTCGTGCGGC--------------------------------------------------------------------------------------------      0.000      0.038      0.038
------------------------------CCTCCAGGTCGTCGTGC-----------------------------------------------------------------------------------------------      0.000      0.076      0.151
---------------------------CGTCCTCCAGGTCGTCGTGC-----------------------------------------------------------------------------------------------      0.000      0.152      0.152
------------------------CCTCGTCCTCCAGGTCGTCGT-------------------------------------------------------------------------------------------------      1.117      0.227      0.227
---------------------------CGTCCTCCAGGTCGTCGT-------------------------------------------------------------------------------------------------      0.049      0.038      0.113
----------------------CTCCTCGTCCTCCAGGTCGT----------------------------------------------------------------------------------------------------      0.194      0.189      0.189
---------------------CCTCCTCGTCCTCCAGGTCGT----------------------------------------------------------------------------------------------------      2.234      3.410      1.506
------------------CCACCTCCTCGTCCTCCAGGT-------------------------------------------------------------------------------------------------------      0.971      0.227      0.301
-------------CGCTGCCACCTCCTCGTCCT-------------------------------------------------------------------------------------------------------------      1.263      0.265      0.339
-------CGCCGCCGCTGCCACCTCCTCGT----------------------------------------------------------------------------------------------------------------      0.000      0.038      0.038
------CCGCCGCCGCTGCCACCTCCT-------------------------------------------------------------------------------------------------------------------      0.097      0.038      0.075
-----GCCGCCGCCGCTGCCACCT----------------------------------------------------------------------------------------------------------------------      0.000      0.038      0.038
----CGCCGCCGCCGCTGCCACCT----------------------------------------------------------------------------------------------------------------------      0.000      0.038      0.038
```

```
miRNA: miR_t54,miR_t15 chromosome_5 Start: 935243 End: 935506 Condition: HS-N
------AGACGTCTGCACCGCACATG------------------------------------------------------------------------------------------------------------      0.049      0.189      0.075
-------------TGCACCGCACATGTCTCAGG-----------------------------------------------------------------------------------------------------      2.186      0.303      0.452
------------------------TGTCTCAGGAAGAGATCGTC-CGTAG------------------------------------------------------------------------------------      0.000      0.038      0.038
------------------------TGTCTCAGGAAGAGATCGTC------------------------------------------------------------------------------------------      0.631      0.076      0.113
--------------------------TCTCAGGAAGAGATCGTC-CG---------------------------------------------------------------------------------------      0.146      0.303      0.151
--------------------------TCTCAGGAAGAGATCGTC-CGTAGTA----------------------------------------------------------------------------------      0.000      0.227      0.227
--------------------------TCTCAGGAAGAGATCGTC-CGTAGT-----------------------------------------------------------------------------------      2.380      3.789      0.527
--------------------------TCTCAGGAAGAGATCGTC-CGTA-------------------------------------------------------------------------------------      0.437      1.099      1.092
--------------------------TCTCAGGAAGAGATCGTC-CGTAG------------------------------------------------------------------------------------     21.321     30.385     23.720 miR_t54
--------------------------TCTCAGGAAGAGATCGTC-CGTAGTAGC--------------------------------------------------------------------------------      0.000      0.455      0.455
--------------------------TCTCAGGAAGAGATCGTC-C----------------------------------------------------------------------------------------      0.243      0.720      0.264
--------------------------TCTCAGGAAGAGATCGTC-CGT--------------------------------------------------------------------------------------      2.477      1.781      1.506
--------------------------TCTCAGGAAGAGATCGTC------------------------------------------------------------------------------------------      0.000      0.682      0.038
---------------------------CTCAGGAAGAGATCGTC-CGTAG------------------------------------------------------------------------------------      0.049      0.152      0.151
----------------------------TCAGGAAGAGATCGTC-CGTAG------------------------------------------------------------------------------------      0.486      0.682      0.791
----------------------------TCAGGAAGAGATCGTC-CGT--------------------------------------------------------------------------------------      0.000      0.076      0.076
---------------------------------------TCGTC-CGTAGTAGCAAGCTGC-------------------------------------------------------------------------      0.000      0.114      0.038
--------------------------------------------------TAGCAAGCTGCTGGAGGGTAT---------------------------------------------------------------      0.000      0.038      0.113
--------------------------------------------------TAGCAAGCTGCTGGAGGGTA----------------------------------------------------------------      0.097      0.076      0.113
-----------------------------------------------------CAAGCTGCTGGAGGGTATCT-------------------------------------------------------------      0.000      0.038      0.038
------------------------------------------------------------------------------------TCGATTGGCAAGACGTCCACT-----------------------------      0.097      0.038      0.038
----------------------------------------------------------------------------------------TTGGCAAGACGTCCACTCCGG-------------------------      0.194      0.076      0.075
----------------------------------------------------------------------------------------TTGGCAAGACGTCCACTCCGGG------------------------      1.408      0.871      1.092
-----------------------------------------------------------------------------------------TGGCAAGACGTCCACTCCGGG------------------------      0.631      0.265      0.753
--------------------------------------------------------------------------------------------CAAGACGTCCACTCCGGGTAC---------------------      0.243      0.038      0.264
---------------------------------------------------------------------------------------------AAGACGTCCACTCCGGGTACG--------------------      0.097      0.038      0.151
---------------------------------------------------------------------------------------------------TCCACTCCGGGTACGCGCCGC--------------      0.194      0.038      0.151
--------------------------------------------------------------------------------------------------------------TACGCGCCGCTCATGCGAT-----      0.000      0.076      0.076

--TA  A         A                 A  G      - GTAG            GG                   C   A         C    A  C                         CG
    CG GACGTCTGC CCGCACATGTCTCAGGA GA ATCGTC C    TAGCAAGCTGCT  AGGGTATCTCGGTCAGCAT TCG TTGGCAAGA GTCC CT CGGGTACGCGCCGCTCATGCGATGA  C
    GC CTGCAGACG GGCGTGTACAGAGTCCT CT TAGCGG G    ATCGTTCGATGA  TCCCATAGAGCCAGTCGTA AGC AACCGTTCT CAGG GA GTCCATGTGCGGCGAGTGCGCTACT  /
TTAC  C         C                 C  G      T ATA-            AG                   A   A         A    C  A                         TT

-------------------------------------------------------------------------------------------------------GAAGTCCATGTGCGGCGAGT-----------      0.777      0.076      0.113
------------------------------------------------------------------------------------------------------CGAAGTCCATGTGCGGCGAGT-----------      0.194      0.227      0.151
----------------------------------------------------------------------------------------------------GGCGAAGTCCATGTGCGGCGA-------------      0.000      0.038      0.075
------------------------------------------------------------------------------------------CCGTTCTACAGGCGAAGTCCAT----------------------     20.399      7.577      1.920
---------------------------------------------------------------------------------------------TTCTACAGGCGAAGTCCAT----------------------      0.049      0.076      0.226
```

```
----------------------------------------------------------------------------------------------TCTACAGGCGAAGTCCAT----------------------      0.000      0.038      0.038
-------------------------------------------------------------------------------------------CGTTCTACAGGCGAAGTCCAT----------------------    225.308    278.652    484.491 miR_t15
-----------------------------------------------------------------------------------------ACCGTTCTACAGGCGAAGTCCAT----------------------      0.097      0.114      0.114
--------------------------------------------------------------------------------------------GTTCTACAGGCGAAGTCCAT----------------------      6.411      2.576      3.125
------------------------------------------------------------------------------------------CCGTTCTACAGGCGAAGTCCA-----------------------      0.097      0.076      0.075
--------------------------------------------------------------------------------------------GTTCTACAGGCGAAGTCCA-----------------------      0.000      0.152      0.038
-------------------------------------------------------------------------------------------CGTTCTACAGGCGAAGTCCA-----------------------      1.797      2.046      6.062
-------------------------------------------------------------------------------------------CGTTCTACAGGCGAAGTCC------------------------      0.437      0.152      0.602
------------------------------------------------------------------------------------------CCGTTCTACAGGCGAAGTCC------------------------      0.000      0.076      0.076
---------------------------------------------------------------------------------------AAACCGTTCTACAGGCGAAGT--------------------------      0.000      0.076      0.076
-------------------------------------------------------------------------------------GCAAACCGTTCTACAGGCGAAGT--------------------------      0.049      0.114      0.114
-------------------------------------------------------------------------GCCAGTCGTAAAGCAAACCGT----------------------------------------      2.526      0.265      0.339
-----------------------------------------------------------------------GAGCCAGTCGTAAAGCAAA--------------------------------------------      0.000      0.038      0.038
----------------------------------------------------------------TCCCATAGAGCCAGTCGTAAA-------------------------------------------------      0.000      0.076      0.226
--------------------------------------------------------------AGTCCCATAGAGCCAGTCGTAA--------------------------------------------------      0.971      1.743      3.502
-------------------------------------------------------------AAGTCCCATAGAGCCAGTCGT----------------------------------------------------      0.680      0.379      0.379
------------------------------------------------------------GAAGTCCCATAGAGCCAGTCGT----------------------------------------------------      0.777      2.008      2.008
--------------------------------------------------------------AGTCCCATAGAGCCAGTCGT----------------------------------------------------      1.554      0.796      0.602
----------------------------------------------------------ATGAAGTCCCATAGAGCCAGT-------------------------------------------------------      0.777      0.644      1.092
--------------------------------------------------------CGATGAAGTCCCATAGAGCCAGT-------------------------------------------------------      0.049      0.152      0.152
-------------------------------------------------------TCGATGAAGTCCCATAGAGC-----------------------------------------------------------      0.000      0.076      0.076
----------------------------------------------------CGTTCGATGAAGTCCCAT----------------------------------------------------------------      0.000      0.076      0.076
---------------------------------------------------TCGTTCGATGAAGTCCCAT----------------------------------------------------------------      0.049      0.038      0.038
-------------------------CAGAGTCCTCCTGTAGCGGTGAT--------------------------------------------------------------------------------------      0.049      0.227      0.227
------------------------ACAGAGTCCTCCTGTAGCGGTGATT--------------------------------------------------------------------------------------      0.000      0.303      0.075 miR_t54*
--------------------------AGAGTCCTCCTGTAGCGGTGAT--------------------------------------------------------------------------------------      0.340      0.038      0.038
-----------------GGCGTGTACAGAGTCCTCCTGT-----------------------------------------------------------------------------------------------      3.691      0.530      0.301
--------------CGCGGCGTGTACAGAGTCCTCCT-------------------------------------------------------------------------------------------------      0.291      0.114      0.114
--------------CGCGGCGTGTACAGAGTCCTC---------------------------------------------------------------------------------------------------      0.291      0.114      0.038
--------------CGCGGCGTGTACAGAGTCCT----------------------------------------------------------------------------------------------------     15.590      6.516     10.806
-------------ACGCGGCGTGTACAGAGTCCT----------------------------------------------------------------------------------------------------      4.614      0.644      1.318
-----------AGACGCGGCGTGTACAGAGT-------------------------------------------------------------------------------------------------------      1.554      0.379      1.092
---------GCAGACGCGGCGTGTACAGAGT-------------------------------------------------------------------------------------------------------     21.370     14.889     16.152
---------GCAGACGCGGCGTGTACAGAG--------------------------------------------------------------------------------------------------------      0.049      0.038      0.113
----GCCCTGCAGACGCGGCGTGT--------------------------------------------------------------------------------------------------------------      8.985      0.114      0.151
```

```
miRNA: miR_t64 chromosome_16 Start: 7434874 End: 7435152 Condition: HS-N
---------TAGACAGTCACAGCGTGCAG-----------------------------------------------------------------------------------------------------------------      0.000      0.038      0.038
---------TAGACAGTCACAGCGTGCAGG----------------------------------------------------------------------------------------------------------------      8.742      2.160      4.179 miR_t64*
----------AGACAGTCACAGCGTGCAGG----------------------------------------------------------------------------------------------------------------      0.049      0.114      0.151
-----------GACAGTCACAGCGTGCAGG----------------------------------------------------------------------------------------------------------------      0.000      0.114      0.114
-------------CAGTCACAGCGTGCAGG----------------------------------------------------------------------------------------------------------------      0.000      0.038      0.038
-----------------CACAGCGTGCAGGTGCCACC---------------------------------------------------------------------------------------------------------      0.000      0.038      0.038
------------------------TGCAGGTGCCACCGCGGTTTTG------------------------------------------------------------------------------------------------      0.000      0.114      0.038
------------------------------TGCCACCGCGGTTTTGAAGCT-------------------------------------------------------------------------------------------      0.437      0.189      0.038
------------------------------TGCCACCGCGGTTTTGAAGC--------------------------------------------------------------------------------------------      1.117     16.405     21.009
------------------------------TGCCACCGCGGTTTTGAAGCTC------------------------------------------------------------------------------------------      0.534      0.303      0.226
-------------------------------GCCACCGCGGTTTTGAAGC--------------------------------------------------------------------------------------------      0.000      0.038      0.565
--------------------------------------CGGTTTTGAAGCTCACGC--------------------------------------------------------------------------------------      0.000      0.038      0.038
-----------------------------------------TTTTGAAGCTCACGCAATGGAGG------------------------------------------------------------------------------      0.049      0.038      0.038
------------------------------------------TTTGAAGCTCACGCAATGGAGG------------------------------------------------------------------------------      1.506      0.341      0.341
-------------------------------------------TTGAAGCTCACGCAATGGAGG------------------------------------------------------------------------------      1.311      0.189      0.301
--------------------------------------------TGAAGCTCACGCAATGGAGGG-----------------------------------------------------------------------------      0.486      0.076      0.076
--------------------------------------------TGAAGCTCACGCAATGGAGG------------------------------------------------------------------------------      2.768      0.114      0.038
---------------------------------------------GAAGCTCACGCAATGGAGG------------------------------------------------------------------------------      0.000      0.038      0.038
----------------------------------------------------------------------------------------------------GCGAGGACGCAGGAGGCGCCG---------------------      0.146      0.038      0.264
-----------------------------------------------------------------------------------------------------CGAGGACGCAGGAGGCGCCG---------------------      0.000      0.152      0.113

-----                                                                      C                                                              TTC
     CCGCTAGACAGTCACAGCGTGCAGGTGCCACCGCGGTTTTGAAGCTCACGCAATGGAGGGCAGTAAAGGA GCCAAGGCGCCGGGAGCGGCCGCGGCGAGGACGCAGGAGGCGCCGGGCGGGCTCAGGTAGGG   C
     GGCGATCTGTCAGTGTCGCACGTCCACGGTGGCGCCAAAACTTCGAGTGCGTTACCTCCCGTCGTTTCCT CGGTTCCGCGGCCCTCGTCGGCGCCGCTCCTGCGTCCTCCGCGGCCCGCCCGAGTCCATTCC   /
GTGAC                                                                      C                                                              TTT

---------------------------------------------------------------------------------------------------------------CCTCCGCGGCCCGCCCGAGT-----------      1.846      0.076      0.038
---------------------------------------------------------------------------------------------------------------CCTCCGCGGCCCGCCCGAG------------      0.000      0.038      0.038
----------------------------------------------------------------------------------------------CGGCGCCGCTCCTGCGTCCT----------------------------      0.097      0.038      0.038
------------------------------------------------------------------------------------------TCGTCGGCGCCGCTCCTGCGT-------------------------------      3.934      0.909      1.242
-------------------------------------------------------------------------------------------CGTCGGCGCCGCTCCTGCGT-------------------------------     29.918     12.048     21.649
-------------------------------------------------------------------------------------------CGTCGGCGCCGCTCCTGCG--------------------------------      0.291      0.152      0.414
-------------------------------------------------------------------------------------GGCCCTCGTCGGCGCCGCTCCT-----------------------------------      0.729      0.114      0.114
--------------------------------------------------------------------------------------GCCCTCGTCGGCGCCGCTCCT-----------------------------------      1.408      0.152      0.565
---------------------------------------------------------------------------------------CCCTCGTCGGCGCCGCTCCT-----------------------------------      0.000      0.038      0.151
----------------------------------------------------------------------------------------CCTCGTCGGCGCCGCTCCT-----------------------------------      0.049      0.038      0.038
------------------------------------------------------------------------CCTCCGGTTCCGCGGCCCTCGT------------------------------------------------      0.049      0.076      0.075
---------------------------------------------------------------------TTTCCTCCGGTTCCGCGGCCCT---------------------------------------------------      0.194      0.038      0.038
-----------------------------------------------------------CCTCCCGTCGTTTCCTCCGGT--------------------------------------------------------------      0.243      0.038      0.075
------------------------------------------------------CGTTACCTCCCGTCGTTTCCT-------------------------------------------------------------------      1.894      0.530      0.640
-------------------------------------------------------GTTACCTCCCGTCGTTTCCT-------------------------------------------------------------------      0.291      0.114      0.038
--------------------------------------------------AGTGCGTTACCTCCCGTCGTTT----------------------------------------------------------------------      0.097      0.038      0.038
---------------------------------------------------GTGCGTTACCTCCCGTCGTTT----------------------------------------------------------------------      0.583      0.038      0.113
------------------------------------------------CGAGTGCGTTACCTCCCGTCGT------------------------------------------------------------------------      4.177      0.379      0.188
--------------------------------------------------AGTGCGTTACCTCCCGTCGT------------------------------------------------------------------------      0.486      0.189      0.189
-------------------------------------------------GAGTGCGTTACCTCCCGTCGT------------------------------------------------------------------------      0.486      0.076      0.076
--------------------------------------GCCAAAACTTCGAGTGCGTT------------------------------------------------------------------------------------      0.000      0.038      0.038
-------------------------------------CGCCAAAACTTCGAGTGCGTT------------------------------------------------------------------------------------      0.486      0.455      0.151
-------------------------------CGGTGGCGCCAAAACTTCGAGT-----------------------------------------------------------------------------------------      1.603      0.455      0.640
----------------------------------TGGCGCCAAAACTTCGAGT-----------------------------------------------------------------------------------------      1.166      0.114      0.075
-----------------------------------GGCGCCAAAACTTCGAGT-----------------------------------------------------------------------------------------      1.846      0.076      0.151
---------------------------------GTGGCGCCAAAACTTCGAGT-----------------------------------------------------------------------------------------     15.979      0.379      0.640
--------------------------------GGTGGCGCCAAAACTTCGAGT-----------------------------------------------------------------------------------------     57.165      4.963      9.714
--------------------------------GGTGGCGCCAAAACTTCGAG------------------------------------------------------------------------------------------      0.340      0.038      0.075
--------------------------GTCCACGGTGGCGCCAAAACTT----------------------------------------------------------------------------------------------      0.097      0.114      0.038
-----------------------------CACGGTGGCGCCAAAACTT----------------------------------------------------------------------------------------------      0.049      0.038      0.038
----------------------------CCACGGTGGCGCCAAAACTT----------------------------------------------------------------------------------------------      2.574      0.682      0.941
-------------------------CGTCCACGGTGGCGCCAAAACT-----------------------------------------------------------------------------------------------      0.097      0.038      0.038
-------------GTCAGTGTCGCACGTCCACGGT-----------------------------------------------------------------------------------------------------------      2.040      0.871      0.979
--------------TCAGTGTCGCACGTCCACGGT-----------------------------------------------------------------------------------------------------------      2.088      0.530      0.339
-----------------GTGTCGCACGTCCACGGT-----------------------------------------------------------------------------------------------------------      0.000      0.038      0.038
----------------AGTGTCGCACGTCCACGGT-----------------------------------------------------------------------------------------------------------      0.194      0.038      0.075
---------------CAGTGTCGCACGTCCACGGT-----------------------------------------------------------------------------------------------------------      2.671      0.114      0.678
-----------CTGTCAGTGTCGCACGTCCAC--------------------------------------------------------------------------------------------------------------      0.049      0.076      0.076
-------CGATCTGTCAGTGTCGCACGT------------------------------------------------------------------------------------------------------------------    103.450    221.255     96.876 miR_t64
----------TCTGTCAGTGTCGCACGT------------------------------------------------------------------------------------------------------------------      0.049      0.038      0.038
--------GATCTGTCAGTGTCGCACGT------------------------------------------------------------------------------------------------------------------      2.283      0.568      0.414
-------CGATCTGTCAGTGTCGCACG-------------------------------------------------------------------------------------------------------------------      0.194      0.303      0.151
-------CGATCTGTCAGTGTCGCA---------------------------------------------------------------------------------------------------------------------      0.000      0.076      0.076
----CGGCGATCTGTCAGTGT-------------------------------------------------------------------------------------------------------------------------      0.000      0.152      0.152
```

```
miRNA: miR_t73 chromosome_5 Start: 852576 End: 852782 Condition: HS-N
----TGCGATATGGCACCCCCGGTG-------------------------------------------------------------------------------------      0.000      0.038      0.113
----TGCGATATGGCACCCCCGGTGC------------------------------------------------------------------------------------      0.097      0.076      0.075
-----------------------TGCACCAGACCTTTTACGATC------------------------------------------------------------------      1.457      0.265      0.565
-----------------------TGCACCAGACCTTTTACGATCC-----------------------------------------------------------------      0.777      0.455      0.038
-----------------------TGCACCAGACCTTTTACGA--------------------------------------------------------------------      0.000      0.076      0.076
-----------------------------------TTTACGATCCGCACGCCCTCTC-----------------------------------------------------      0.049      0.038      0.226
-----------------------------------TTTACGATCCGCACGCCCTCT------------------------------------------------------      0.097      0.038      0.038
------------------------------------TTACGATCCGCACGCCCTCTC-----------------------------------------------------      0.534      0.833      1.318
------------------------------------TTACGATCCGCACGCCCTCTCAG---------------------------------------------------      0.000      0.114      0.038
-------------------------------------TACGATCCGCACGCCCTCTCAG---------------------------------------------------      0.000      0.114      0.114
------------------------------------------------------------------TCGCAAAGACCTGATGGGCTG-----------------------      8.257      0.606      0.301
------------------------------------------------------------------TCGCAAAGACCTGATGGGCTGC----------------------      1.651      0.303      0.602

ACCCTGCGATAT               C   A         A                           C   G     A     T C          G  CCCA   TA
            GGCACCCCCGGTGCA CAG CCTTTTACG TCCGCACGCCCTCTCAGGCCCCCATCG AAA ACCTG TGGGC G GTCAGGCGTA GC    GCG  A
            CCGTGGGGGCCACGT GTC GGAAAGTGT AGGCGTGCGGGAGGGTCCGGGGGTAGC TTT TGGAC GCCCG C CGGTCCGCGT CG    CGC  /
------------               C   G         G                           T   G     C     T T          A  C---   CC

-------------------------------------------------------GGTCCGGGGGTAGCTTTTGT-----------------------------------      2.283      0.038      0.038
---------------------------------------------------GGAGGGTCCGGGGGTAGCTTTT-------------------------------------      2.137      0.265      0.265
---------------------------------------------------GGAGGGTCCGGGGGTAGCTTT--------------------------------------      3.545      0.720      0.151
--------------------------------------------------GGGAGGGTCCGGGGGTAGCTT---------------------------------------      0.049      0.076      0.075
---------------------------------------------------GGAGGGTCCGGGGGTAGCTT---------------------------------------      0.680      0.493      0.678
--------------------------------------------------GGGAGGGTCCGGGGGTAGCT----------------------------------------      0.049      0.038      0.038
-----------------------------------------------------AGGGTCCGGGGGTAGCT----------------------------------------      0.000      0.038      0.038
------------------------------------------------GCGGGAGGGTCCGGGGGTAGCT----------------------------------------      0.631      0.076      0.076
-------------------------------------------------CGGGAGGGTCCGGGGGTAGCT----------------------------------------      0.000      0.038      0.075
---------------------------------------------CGTGCGGGAGGGTCCGGGGGT--------------------------------------------     45.266      5.190     36.258
--------------------------------------------GCGTGCGGGAGGGTCCGGGGGT--------------------------------------------      0.194      0.189      0.565
---------------------------------------------CGTGCGGGAGGGTCCGGGGG---------------------------------------------      0.389      0.038      0.489
-------------------------------------------GGCGTGCGGGAGGGTCCGGGG----------------------------------------------      0.049      0.265      0.188
-------------------------------------GTGTGAGGCGTGCGGGAGGGT----------------------------------------------------      0.631      0.189      0.301
------------------------------------AGTGTGAGGCGTGCGGGAGGGT----------------------------------------------------      0.146      0.076      0.075
---------------------------------GAAAGTGTGAGGCGTGCGGGAG-------------------------------------------------------      0.340      0.530      0.113
---------------------------------GAAAGTGTGAGGCGTGCGGGA--------------------------------------------------------      0.000      0.076      0.038
------------------------------CGGGAAAGTGTGAGGCGT--------------------------------------------------------------      0.194      0.341      0.640
-----------------------------TCGGGAAAGTGTGAGGCGT--------------------------------------------------------------      0.146      0.152      0.075
----------------------------GTCGGGAAAGTGTGAGGCGT--------------------------------------------------------------      3.011      0.606      1.280
---------------------------CGTCGGGAAAGTGTGAGGCGT--------------------------------------------------------------     56.048     89.676     50.226 miR_t73
--------------------------TCGTCGGGAAAGTGTGAGGCGT--------------------------------------------------------------      0.146      0.076      0.076
---------------------------CGTCGGGAAAGTGTGAGGCG---------------------------------------------------------------      0.243      0.265      0.188
----------------------CACGTCGTCGGGAAAGTGT---------------------------------------------------------------------      0.000      0.076      0.076
---------------------CCACGTCGTCGGGAAAGTGT---------------------------------------------------------------------      0.000      0.152      0.301
-----------------GGGGCCACGTCGTCGGGAAAGT-----------------------------------------------------------------------      0.049      0.114      0.264
----------------GGGGGCCACGTCGTCGGGAAAGT-----------------------------------------------------------------------      0.631      0.038      0.038
---------------------CCACGTCGTCGGGAAAGT-----------------------------------------------------------------------      0.194      0.189      0.188
------------------GGGCCACGTCGTCGGGAAAGT-----------------------------------------------------------------------     41.671      9.434     11.559
----------------------CACGTCGTCGGGAAAGT-----------------------------------------------------------------------      0.000      0.038      0.038
-------------------GGCCACGTCGTCGGGAAAGT-----------------------------------------------------------------------      0.486      0.265      0.151
```

```
miRNA: miR_t79,miR_t43 chromosome_2 Start: 3047224 End: 3047445 Condition: HS-N
----------------------------------------------TACTGGAGGCGCCGCAGCGC-------------------------------------------------      0.049      0.189      0.113
----------------------------------------------TACTGGAGGCGCCGCAGCGCA------------------------------------------------      0.000      0.152      0.075
----------------------------------------TGGGCCTACTGGAGGCG----------------------------------------------------------      0.146      0.076      0.038
----------------------------------------TGGGCCTACTGGAGGCGC---------------------------------------------------------      0.389      0.038      0.075
--------------------------------------TGTGGGCCTACTGGAGGC-----------------------------------------------------------      0.340      0.493      0.866
--------------------------------------TGTGGGCCTACTGGAGGCG----------------------------------------------------------      3.351      1.288      0.791
```

Sequence				
TGTGGGCCTACTGGAGGCGC	3.448	2.463	1.958	
TGTGGGCCTACTGGAGG	1.651	0.189	0.339	
TGTGGGCCTACTGGAGGCGCC	3.497	0.379	0.226	
AGCTGTGGGCCTACTGGAGGC	0.000	0.493	0.602	
AGCTGTGGGCCTACTGGAG	0.097	0.038	0.113	
AGCTGTGGGCCTACTGGAGGCG	0.000	0.114	0.114	
CAGCTGTGGGCCTACTGGAGGCGC	0.000	0.114	0.114	
CAGCTGTGGGCCTACTGGAGG	9.616	9.320	3.539	
CAGCTGTGGGCCTACTGGAGGC	0.243	4.243	1.017	
CAGCTGTGGGCCTACTGGAGGCG	0.194	2.008	0.377	
CAGCTGTGGGCCTACTGGAG	38.806	31.635	15.964	
GCAGCTGTGGGCCTACTGGAG	0.146	0.530	0.530	
AGGCAGCTGTGGGCCTACTGG	0.000	0.114	0.114	
AGCAGCACGCCGCAGCAGGCA	0.000	0.038	0.038	
TCCAGCAGCACGCCGCAGCAG	2.428	1.781	0.565	

```
--------                                                                   C         AA          - TG  A-  G    G  TT
        GAGGTCCAGCAGCACGCCGCAGCAGGCAGCTGTGGGCCTACTGGAGGCGCCGCAGCGCAACAACA CGATTGCCA  GCCACAGCCA G  CG  GT CGAG GC  C
        CTCCAGGTCGTCGTGCGGCGTCGTCCGTCGACACCCGGATGACCTCCGCGGCGTCGCGTTGTTGT GCTAACGGT  CGGTGTCGGT C  GC  CG GCTT CG  T
TACTCGTC                                                                   A         CG          T GT  GG  -    G  TT
```

Sequence				
CGTCCTCCAGGTCGTCGT	0.049	0.038	0.113	
CCTCCAGGTCGTCGTGC	0.000	0.076	0.151	
CGTCCTCCAGGTCGTCGTGC	0.000	0.152	0.152	
CCTCCAGGTCGTCGTGCGGC	0.000	0.038	0.038	
CCAGGTCGTCGTGCGGCGT	0.340	0.189	0.188	
CCTCCAGGTCGTCGTGCGGCGT	0.146	0.871	1.393	
TCCAGGTCGTCGTGCGGCGT	0.146	0.038	0.226	
TCCTCCAGGTCGTCGTGCGGCGT	0.000	0.076	0.076	
CTCCAGGTCGTCGTGCGGCGT	0.000	0.265	0.075	
CCAGGTCGTCGTGCGGCGTC	0.000	0.076	0.076	
CCAGGTCGTCGTGCGGCGTCG	2.234	0.076	0.076	
CAGGTCGTCGTGCGGCGTCG	0.000	0.038	0.038	
AGGTCGTCGTGCGGCGTCGT	2.866	0.909	0.941	
TCCAGGTCGTCGTGCGGCGTCGT	2.866	3.523	0.301	
CCAGGTCGTCGTGCGGCGTCGT	340.366	104.831	4.179	miR_t79
CAGGTCGTCGTGCGGCGTCGT	5.780	3.296	0.866	
GGTCGTCGTGCGGCGTCGT	3.060	2.917	1.167	
TCGTCGTGCGGCGTCGTCCGT	0.049	0.038	0.038	
CCGTCGACACCCGGATGACCT	1.117	0.833	0.339	
CGTCGACACCCGGATGACCT	0.097	0.038	0.226	
GTCGACACCCGGATGACCTCC	0.000	0.038	0.038	
TGACCTCCGCGGCGTCGCGTT	0.097	0.189	0.151	
GACCTCCGCGGCGTCGCGTT	0.000	0.038	0.038	
CGGCGTCGCGTTGTTGT	0.097	0.303	0.264	
TCCGCGGCGTCGCGTTGTTGT	0.631	0.189	0.113	
GCGGCGTCGCGTTGTTGT	0.049	0.265	0.038	
CGCGGCGTCGCGTTGTTGT	4.857	2.084	1.506	
CCGCGGCGTCGCGTTGTTGT	2.137	1.440	0.527	
CGGCGTCGCGTTGTTGTA	0.000	0.038	0.038	
CCGCGGCGTCGCGTTGTTGTA	0.000	0.076	0.076	
GGCGTCGCGTTGTTGTAG	0.437	0.038	0.038	
GCGGCGTCGCGTTGTTGTAG	0.000	0.152	0.152	
CGCGGCGTCGCGTTGTTGTAG	0.000	0.038	0.038	
CGGCGTCGCGTTGTTGTAG	1.748	4.546	2.297	
GCGGCGTCGCGTTGTTGTAGC	0.097	0.152	0.152	
GGCGTCGCGTTGTTGTAGC	0.534	0.076	0.076	
CGGCGTCGCGTTGTTGTAGC	2.137	1.023	0.414	
CGGCGTCGCGTTGTTGTAGCT	429.100	1520.293	964.388	miR_t43
GCGGCGTCGCGTTGTTGTAGCT	4.517	2.879	0.414	
GGCGTCGCGTTGTTGTAGCT	237.110	59.481	32.229	
GCGTCGCGTTGTTGTAGCT	0.486	1.629	0.452	
CGTCGCGTTGTTGTAGCT	0.000	0.720	0.038	
GGCGTCGCGTTGTTGTAGCTA	0.437	1.061	1.205	
GCGTCGCGTTGTTGTAGCTA	0.000	0.038	0.038	
GGCGTCGCGTTGTTGTAGCTAA	0.389	0.985	0.527	
CGGCGTCGCGTTGTTGTAGCTAA	0.000	0.076	0.076	
GCGTCGCGTTGTTGTAGCTAA	0.583	5.153	2.184	
CGTCGCGTTGTTGTAGCTAA	0.097	0.455	0.226	
CGTCGCGTTGTTGTAGCTAAC	0.243	0.985	1.167	

miRNA: miR_t81 chromosome_10 Start: 5650253 End: 5650622 Condition: HS-N

Sequence				
TGGGCTTCAAACCAGCAGGTC	0.389	0.189	0.188	
TGGGCTTCAAACCAGCAG	0.000	0.038	0.113	
TGGGCTTCAAACCAGCAGG	0.729	0.114	0.113	
TGGCAGCAGCGCAGTGGGCTTC	0.146	0.114	0.038	
TTGGATGGCAGCAGCGCAGTGG	0.146	0.265	0.489	
TTTGGATGGCAGCAGCGCAG	1.943	0.114	0.339	
TCCGTTTGGATGGCAGCAGCGC	0.049	0.152	0.113	
TCCGTTTGGATGGCAGCAG	8.839	5.456	14.157	
TCCGTTTGGATGGCAGCAGC	0.097	0.455	0.489	
TCCGTTTGGATGGCAGCAGCG	0.486	1.061	2.184	
ATCCGTTTGGATGGCAGCAG	0.000	0.114	0.377	
GATCCGTTTGGATGGCAGCAG	0.000	0.076	0.113	
GGATCCGTTTGGATGGCAGC	0.000	0.152	0.151	
TGGATCCGTTTGGATGGCAGC	1.068	1.781	1.280	
TTGGATCCGTTTGGATGGCAG	0.097	0.152	0.152	
TTGGATCCGTTTGGATGGCAGC	0.777	1.364	0.301	
GCTTGGATCCGTTTGGATGG	0.194	0.568	0.414	
TGCTTGGATCCGTTTGGATGG	11.705	57.625	40.738	miR_t81
TGCTTGGATCCGTTTGGATGGC	1.748	3.296	0.188	
TGCTTGGATCCGTTTGGATG	0.437	0.152	0.038	
TCGTGCTTGGATCCGTTTGG	0.000	0.265	0.075	
TCGTGCTTGGATCCGTTTGGA	0.437	0.720	0.678	
TATCGTGCTTGGATCCGTTTG	0.097	0.114	0.114	
TCTCAGGCTATCGTGCTTGGA	0.049	0.038	0.151	
TCAGGTCCGGCCTGGAGCAGC	0.049	0.265	0.264	
TCTCAGGTCCGGCCTGGAGC	0.146	0.076	0.038	
TTCTCAGGTCCGGCCTGGAGC	0.340	0.152	0.075	
TGTTCTCAGGTCCGGCCTGGA	0.340	0.493	0.565	
TGTTCTCAGGTCCGGCCTGG	0.583	0.038	0.038	
TCGCGTCCCTGTTCTCAGG	0.000	0.152	0.152	
TTCCGCGCCAGCTGCTCCAGG	0.194	0.076	0.113	
TTCCGCGCCAGCTGCTCCAG	0.000	0.038	0.038	
TGAAAGTTCCGCGCCAGCTGC	1.603	0.038	0.113	

```
------------------          TTC             C              C                                                                                                                  ----       -----    A     ---    CCTC
                  AACATGAAAG   CGCGCCAGCTGCT CAGGAGCTCGCGTCC TGTTCTCAGGTCCGGCCTGGAGCAGCAATGTCTCAGGCTATCGTGCTTGGATCCGTTTGGATGGCAGCAGCGCAGTGGGCTTCAAACCAGCAGGTCCGGAT    TCAGGAG     GCCA GCCGG   AGAC    G
                  TTGTACTTTC   GCGCGGTCGACGA GTCCTCGAGCGCAGG ACAAGAGTCCAGGCCGGACCTCGTCGTTACAGAGTCCGATAGCACGAACCTAGGCAAACCTACCGTCGTCGCGTCACCCGAAGTTTGGTCGTCCAGGTCTA    AGTCCTC     CGGT CGGCC   TCTG    /
GGTTGTTTCGCTTCGTTCC         CAA             C              C                                                                                                                  AGCA       GGAAG    -     CCG    AAAA
```

Sequence			
GTTTCGCTTCGTTCCTTGTACTT	0.000	0.114	0.114
CCTTGTACTTTCCAAGCGCGGT	0.243	0.038	0.038
TCCAAGCGCGGTCGACGACGT	0.049	0.076	0.151
GACGACGTCCTCGAGCGCAGGC	0.049	0.152	0.226
CGACGTCCTCGAGCGCAGGC	0.000	0.038	0.113
CGTCCTCGAGCGCAGGCACAAGAGT	0.000	0.038	0.038
CCTCGAGCGCAGGCACAAGAGT	0.777	0.530	1.468
CTCGAGCGCAGGCACAAGAGT	0.923	0.076	0.640
CGAGCGCAGGCACAAGAGT	0.194	0.341	0.941
GGCACAAGAGTCCAGGCCGGAC	0.000	0.076	0.076
GGCACAAGAGTCCAGGCCGGACCT	0.000	0.038	0.038
CCAGGCCGGACCTCGTCGTT	0.000	0.114	0.038
GATAGCACGAACCTAGGCAAA	0.000	0.265	0.265
GCACGAACCTAGGCAAACCT	0.000	0.038	0.038
CGCGTCACCCGAAGTTTGGT	0.194	0.076	0.076

miRNA: miR_t47,miR_t84 chromosome_10 Start: 5682678 End: 5683022 Condition: HS-N

Sequence				
TTTGCGTCCTCACCCCCCTGGC	0.000	0.076	0.076	
TTTGCGTCCTCACCCCCCTG	0.000	0.038	0.038	
TTTGCGTCCTCACCCCCCTGG	0.000	0.038	0.075	
TGTTTGCGTCCTCACCCCCCT	0.000	0.265	0.075	
GGAAGGGAGAACGGTGGCC	1.603	1.629	3.916	
GGAAGGGAGAACGGTGGC	0.146	0.114	0.264	
CGGAAGGGAGAACGGTGGCC	5.100	3.107	8.170	
CGGAAGGGAGAACGGTGGCCA	0.340	0.227	0.264	
CGGAAGGGAGAACGGTGGC	0.097	0.189	0.828	
TCGGAAGGGAGAACGGTGGC	72.172	239.289	306.214	
TCGGAAGGGAGAACGGTGGCCA	26.858	6.895	18.411	
TCGGAAGGGAGAACGGTGGCCAGT	0.049	0.038	0.038	
TCGGAAGGGAGAACGGTGGCC	1040.525	1723.628	3273.287	miR_t47
TCGGAAGGGAGAACGGTGGCCAG	0.097	0.076	0.113	
TTCGGAAGGGAGAACGGTGGCC	2.137	2.500	2.033	
TTCGGAAGGGAGAACGGTGGCCAG	0.049	0.038	0.038	
TTCGGAAGGGAGAACGGTGGCCA	0.049	0.038	0.075	
TTCGGAAGGGAGAACGGTGGC	46.577	133.170	185.506	
TTTCGGAAGGGAGAACGGTGGC	43.517	138.019	46.198	
TTTCGGAAGGGAGAACGGTGGCCAG	0.000	0.038	0.038	
TTTCGGAAGGGAGAACGGTGGCC	0.243	0.114	0.075	
TTTTCGGAAGGGAGAACGGTGGCCAG	0.000	0.038	0.038	
TTTTCGGAAGGGAGAACGGTGGC	0.243	0.909	0.264	
CAGCGAACAACTTTGAGC	0.000	0.114	0.114	
CAGCGAACAACTTTGAGCAAACATAGGGC	0.000	0.076	0.076	
GACAGCGAACAACTTTGAGC	0.049	0.038	0.038	
GGACAGCGAACAACTTTGAG	1.068	0.455	0.455	
GGACAGCGAACAACTTTG	0.437	0.985	0.151	
GGACAGCGAACAACTTTGAGC	1.797	3.372	0.301	
GGACAGCGAACAACTTTGA	0.097	0.189	0.189	
TGGACAGCGAACAACTTTGAGCAA	0.049	0.076	0.076	
TGGACAGCGAACAACTTTGA	70.230	35.158	11.408	
TGGACAGCGAACAACTTT	0.194	0.341	0.113	
TGGACAGCGAACAACTTTGAGCA	0.291	0.152	0.152	
TGGACAGCGAACAACTTTG	67.461	65.884	28.313	
TGGACAGCGAACAACTTTGAG	201.364	55.238	14.721	
TGGACAGCGAACAACTTTGAGCAAA	0.049	0.114	0.114	

```
----------------------------------------------------------------------------------------TGGACAGCGAACAACTTTGAGC------------------------------------------------------------------------------   428.857   1387.237   45.859 miR_t84
---------------------------------------------------------------------------------------ATGGACAGCGAACAACTTTG---------------------------------------------------------------------------------   0.097   0.114   0.114
--------------------------------------------------------------------------------------GATGGACAGCGAACAACTTTG---------------------------------------------------------------------------------   0.000   0.076   0.076
-------------------------------------------------------------------------------------CGATGGACAGCGAACAACT------------------------------------------------------------------------------------   0.000   0.038   0.038
-----------------------------------------------------------------------------------CCCGATGGACAGCGAACAAC-------------------------------------------------------------------------------------   0.000   0.076   0.075
-----------------------------------------------------------------------------------CCCGATGGACAGCGAACAACT------------------------------------------------------------------------------------   0.097   0.227   0.038
----------TGGGGGCAA-AAAACAACTTG-------------------------------------------------------------------------------------------------------------------------------------------------------------   1.263   0.076   0.151
------GGGATGGGGGCAA-AAAACAAC----------------------------------------------------------------------------------------------------------------------------------------------------------------   0.049   0.227   0.227
-----CGGGATGGGGGCAA-AAAACAAC----------------------------------------------------------------------------------------------------------------------------------------------------------------   0.000   0.076   0.076
----TCGGGATGGGGGCAA-AAAAC-------------------------------------------------------------------------------------------------------------------------------------------------------------------   0.000   0.038   0.414
----TCGGGATGGGGGCAA-AAAACA------------------------------------------------------------------------------------------------------------------------------------------------------------------   0.243   0.038   0.904
----TCGGGATGGGGGCAA-AAAACAA-----------------------------------------------------------------------------------------------------------------------------------------------------------------   0.097   0.076   0.076
----TCGGGATGGGGGCAA-AAAACAAC----------------------------------------------------------------------------------------------------------------------------------------------------------------   0.049   0.038   0.038

GGGG             AA-               T                                                                                                              C   TCCACGCTGTTTGCGTCCTCACCCCCCT  CTTCC   AG
    TCGGGATGGGGGC   AAAACAACTTGTTAA CAACTCATCAAGTTGTTATTGCGTGCGCACCCAATCCCCCCGACACCCCCGATGGACAGCGAACAACTTTGAGCAAACATAGGGCTTTTCGGAAGGGAGAACGGTGG CAG                            GG     CGC  G
    AGCCCTACCCCCG   TTTTGTTGAACAATT GTTGAGTAGTTCAACAATAACGCACGCGTGGGTTAGGGGGGCTGTGGGGGCTACCTGTCGCTTGTTGAAACTCGTTTGTATCCCGAAAAGCCTTCCCTCTTGCCACC GTT                            CC     GCG  /
----             CTC               T                                                                                                              A   TG--------------------------  ACTTC   CC

--------CTACCCCCGCTCTTTTGTT-----------------------------------------------------------------------------------------------------------------------------------------------------------------   0.000   0.038   0.038
---------------------------GAACAATTTGTTGAGTAGT----------------------------------------------------------------------------------------------------------------------------------------------   0.049   0.038   0.038
--------------------------TGAACAATTTGTTGAGTAGT----------------------------------------------------------------------------------------------------------------------------------------------   0.243   0.038   0.038
------------------------------CAATTTGTTGAGTAGTT---------------------------------------------------------------------------------------------------------------------------------------------   0.000   0.038   0.038
--------------------------TGAACAATTTGTTGAGTAGTT---------------------------------------------------------------------------------------------------------------------------------------------   0.680   0.076   0.076
---------------------------GAACAATTTGTTGAGTAGTT---------------------------------------------------------------------------------------------------------------------------------------------   31.424   3.789   1.995
---------------------------GAACAATTTGTTGAGTAGTTC--------------------------------------------------------------------------------------------------------------------------------------------   0.097   0.152   0.152
-----------------------------------------------------------------------------------GGGCTACCTGTCGCTTGTTGA------------------------------------------------------------------------------------   0.486   0.871   0.489
-----------------------------------------------------------------------------------GGGCTACCTGTCGCTTGTTGAA-----------------------------------------------------------------------------------   0.049   0.265   0.075
---------------------------------------------------------------------------------GGGGGCTACCTGTCGCTTGTTGAA-----------------------------------------------------------------------------------   0.000   0.038   0.038
------------------------------------------------------------------------------------GGCTACCTGTCGCTTGTTGAA-----------------------------------------------------------------------------------   3.643   2.425   2.861
-------------------------------------------------------------------------------------GCTACCTGTCGCTTGTTGAA-----------------------------------------------------------------------------------   0.826   2.841   4.782
-------------------------------------------------------------------------------------GCTACCTGTCGCTTGTTGAAA----------------------------------------------------------------------------------   0.097   0.189   1.017
------------------------------------------------------------------------------------------CTGTCGCTTGTTGAAACT--------------------------------------------------------------------------------   0.000   0.076   0.076
----------------------------------------------------------------------------------------ACCTGTCGCTTGTTGAAACT--------------------------------------------------------------------------------   7.285   1.402   1.167
--------------------------------------------------------------------------------------CTACCTGTCGCTTGTTGAAACT--------------------------------------------------------------------------------   0.826   0.341   0.791 miR_t84*
-----------------------------------------------------------------------------------------CCTGTCGCTTGTTGAAACT--------------------------------------------------------------------------------   0.097   0.114   0.114
-------------------------------------------------------------------------------------GCTACCTGTCGCTTGTTGAAACT--------------------------------------------------------------------------------   0.243   0.606   0.715
---------------------------------------------------------------------------------------TACCTGTCGCTTGTTGAAACT--------------------------------------------------------------------------------   0.389   0.038   0.075
-------------------------------------------------------------------------------------------TGTCGCTTGTTGAAACTCGT-----------------------------------------------------------------------------   0.194   0.076   0.076
------------------------------------------------------------------------------------------CTGTCGCTTGTTGAAACTCGT-----------------------------------------------------------------------------   2.477   0.265   0.075
----------------------------------------------------------------------------------------ACCTGTCGCTTGTTGAAACTCGT-----------------------------------------------------------------------------   0.049   0.114   0.038
-----------------------------------------------------------------------------------------CCTGTCGCTTGTTGAAACTCGT-----------------------------------------------------------------------------   1.603   0.455   0.414
------------------------------------------------------------------------------------------CTGTCGCTTGTTGAAACTCGTT----------------------------------------------------------------------------   0.826   0.417   0.339
-------------------------------------------------------------------------------------------TGTCGCTTGTTGAAACTCGTT----------------------------------------------------------------------------   3.400   0.417   0.301
--------------------------------------------------------------------------------------------GTCGCTTGTTGAAACTCGTT----------------------------------------------------------------------------   0.874   0.114   0.114
------------------------------------------------------------------------------------------CTGTCGCTTGTTGAAACTCGTTT---------------------------------------------------------------------------   0.049   0.076   0.076
-------------------------------------------------------------------------------------------TGTCGCTTGTTGAAACTCGTTT---------------------------------------------------------------------------   0.874   0.038   0.038
--------------------------------------------------------------------------------------------GTCGCTTGTTGAAACTCGTTT---------------------------------------------------------------------------   21.467   2.122   1.544
```

miRNA: miR_t85,miR_t119 chromosome_8 Start: 1981843 End: 1982190 Condition: HS-N

```
-------------------------------------------------------------------------------------------------------------------------------------------------------------------------------------------------TGTGGGCTGATTTGCCAGC--------------------   0.729   0.152   0.075
----------------------------------------------------------------------------------------------------------------------------------------------------------------------------------------------GGGTGTGGGCTGATTTGCCAG---------------------   0.000   0.038   0.038
---------------------------------------------------------------------------------------------------------------------------------------------------------------------------------------------TGGGTGTGGGCTGATTTGC------------------------   0.049   0.038   0.038
--------------------------------------------------------------------------------------------------------------------------------------------------------------------------------------------GTGGGTGTGGGCTGATTTGC------------------------   0.097   0.038   0.038
-------------------------------------------------------------------------------------------------------------------------------------------------------------------------------------------TGTGGGTGTGGGCTGATTT--------------------------   0.000   0.076   0.076
-------------------------------------------------------------------------------------------------------------------------------------------------------------------------------------------TGTGGGTGTGGGCTGATTTGC------------------------   9.908   7.350   4.330
------------------------------------------------------------------------------------------------------------------------------------------------------------------------------------------TTGTGGGTGTGGGCTGATTTGCCAGC--------------------   0.000   0.038   0.038
------------------------------------------------------------------------------------------------------------------------------------------------------------------------------------------TTGTGGGTGTGGGCTGATTTGCC-----------------------   0.146   0.114   0.114
------------------------------------------------------------------------------------------------------------------------------------------------------------------------------------------TTGTGGGTGTGGGCTGATTTGC------------------------   11.171   5.153   1.167
----------------------------------------------------------------------------------------------------------------------------------------------------------------------------TGGGTGTGACAACATTGTGGG---------------------------------------   0.291   0.189   0.189
----------------------------------------------------------------------------------------------------------------------------------------------------------------------------TGGGTGTGACAACATTGTGG----------------------------------------   0.000   0.038   0.038
--------------------------------------------------------------------------------------------------------------------------------------------TGAAGCTGGAGCTGCGCCTATA----------------------------------------------------------------------   0.000   0.038   0.075
---------------------------------------------------------------------------------------------------------------------------------GGACGAGCTCCTGAAGCTGG-----------------------------------------------------------------------------------   0.340   0.530   0.489
--------------------------------------------------------------------------------------------------------------------------------TGGACGAGCTCCTGAAGCTGG-----------------------------------------------------------------------------------   32.395   60.807   37.237 miR_t85
--------------------------------------------------------------------------------------------------------------------------------TGGACGAGCTCCTGAAGCTGGAG---------------------------------------------------------------------------------   0.000   0.114   0.114
--------------------------------------------------------------------------------------------------------------------------------TGGACGAGCTCCTGAAGCTGGAGC--------------------------------------------------------------------------------   0.000   0.114   0.114
--------------------------------------------------------------------------------------------------------------------------------TGGACGAGCTCCTGAAGCTGGA----------------------------------------------------------------------------------   3.254   2.197   0.791
----------------------------------------------------------------------------------------------------------------------------TGCGTGGACGAGCTCCTGA-----------------------------------------------------------------------------------------   0.631   0.947   0.301
----------------------------------------------------------------------------------------------------------------------------TGCGTGGACGAGCTCCTGAAGC--------------------------------------------------------------------------------------   1.166   1.137   1.137
----------------------------------------------------------------------------------------------------------------------------TGCGTGGACGAGCTCCTGAA----------------------------------------------------------------------------------------   1.846   1.364   0.678
----------------------------------------------------------------------------------------------------------------------------TGCGTGGACGAGCTCCTGAAG---------------------------------------------------------------------------------------   15.396   10.267   4.029
--------------------------------------------------------------------------------------------------------------------------ACTGCGTGGACGAGCTCCTGA-----------------------------------------------------------------------------------------   0.243   0.417   0.188
-------------------------------------------------------------------------------------------------------------------------GACTGCGTGGACGAGCTCCTG------------------------------------------------------------------------------------------   0.340   0.493   0.113
-----------------------------------------------------------------------------------------------------------------------CGGACTGCGTGGACGAGCTCC--------------------------------------------------------------------------------------------   0.049   0.114   0.114
------------------------------------------------------------------------------------------------------------TGCCAAGGCAGCGGACTGCG--------------------------------------------------------------------------------------------------------   0.146   0.189   0.188
------------------------------------------------------------------------------------------------------------TGCCAAGGCAGCGGACTGCGTGGACGAGC-----------------------------------------------------------------------------------------------   0.000   0.038   0.038
----------------------------------------------------------------------------------------------------TTCCCCACTGCCAAGGCAGC----------------------------------------------------------------------------------------------------------------   0.194   0.076   0.076
--------------------------------------------------------------------------------------------------TTTTCCCCACTGCCAAGGCAGC----------------------------------------------------------------------------------------------------------------   0.000   0.038   0.038
--------------------------------------------------------------------------------GGACAGACGCGCTAACCATTT-----------------------------------------------------------------------------------------------------------------------------------   0.049   0.114   0.114
-------------------------------------------------------------------------------TGGACAGACGCGCTAACCA--------------------------------------------------------------------------------------------------------------------------------------   0.000   0.038   0.038
-------------------------------------------------------------------------------TGGACAGACGCGCTAACCATTTT----------------------------------------------------------------------------------------------------------------------------------   1.554   0.644   0.075
-------------------------------------------------------------------------------TGGACAGACGCGCTAACCATT------------------------------------------------------------------------------------------------------------------------------------   5.780   1.250   2.334
-------------------------------------------------------------------------------TGGACAGACGCGCTAACCAT-------------------------------------------------------------------------------------------------------------------------------------   0.097   0.038   0.075
-------------------------------------------------------------------------------TGGACAGACGCGCTAACCATTT-----------------------------------------------------------------------------------------------------------------------------------   33.949   18.034   20.783
--------------------------------------------------------------------------TCAAGTGGACAGACGCGCTAA-----------------------------------------------------------------------------------------------------------------------------------------   0.000   0.152   0.038
--------------------------------------------------------------------------TCAAGTGGACAGACGCGCTAACC---------------------------------------------------------------------------------------------------------------------------------------   0.097   0.114   0.114
-------------------------------------------------------------TCGCTCAAGGTAATCAAGTGGACA---------------------------------------------------------------------------------------------------------------------------------------------------   0.000   0.076   0.038
-------------------------------------------------------------TCGCTCAAGGTAATCAAGTGG------------------------------------------------------------------------------------------------------------------------------------------------------   0.923   0.379   0.075
-------------------------------------------------------------TCGCTCAAGGTAATCAAGTGGA-----------------------------------------------------------------------------------------------------------------------------------------------------   0.340   0.114   0.038
--------------------------------TATGGGTCAGCATCCTGCTGA-----------------------------------------------------------------------------------------------------------------------------------------------------------------------------------   0.097   0.038   0.038
-----------------TGATCCGGCTGGCAGTATG----------------------------------------------------------------------------------------------------------------------------------------------------------------------------------------------------   0.049   0.038   0.038
-----------------TGATCCGGCTGGCAGTATGGG--------------------------------------------------------------------------------------------------------------------------------------------------------------------------------------------------   6.460   4.433   7.116
-----------------TGATCCGGCTGGCAGTATGGGT-------------------------------------------------------------------------------------------------------------------------------------------------------------------------------------------------   1.263   0.038   0.038
-----------------TGATCCGGCTGGCAGTATGG---------------------------------------------------------------------------------------------------------------------------------------------------------------------------------------------------   0.000   0.076   0.339
----------------TTGATCCGGCTGGCAGTAT-----------------------------------------------------------------------------------------------------------------------------------------------------------------------------------------------------   0.000   0.038   0.038
----------------TTGATCCGGCTGGCAGTATG----------------------------------------------------------------------------------------------------------------------------------------------------------------------------------------------------   0.340   1.137   0.565
----------------TTGATCCGGCTGGCAGTATGGGT-------------------------------------------------------------------------------------------------------------------------------------------------------------------------------------------------   1.554   0.379   0.379
----------------TTGATCCGGCTGGCAGTATGGG--------------------------------------------------------------------------------------------------------------------------------------------------------------------------------------------------   50.462   46.297   68.600 miR_t119
----------------TTGATCCGGCTGGCAGTATGG---------------------------------------------------------------------------------------------------------------------------------------------------------------------------------------------------   2.671   2.311   8.246
---------------CTTGATCCGGCTGGCAGTATGGG--------------------------------------------------------------------------------------------------------------------------------------------------------------------------------------------------   0.000   0.038   0.188
--------------CCTTGATCCGGCTGGCAG--------------------------------------------------------------------------------------------------------------------------------------------------------------------------------------------------------   0.000   0.038   0.038
-------------ACCTTGATCCGGCTGGCAGTATG----------------------------------------------------------------------------------------------------------------------------------------------------------------------------------------------------   0.000   0.038   0.038
-------------ACCTTGATCCGGCTGGCAG--------------------------------------------------------------------------------------------------------------------------------------------------------------------------------------------------------   0.097   0.114   0.226
-----------GCACCTTGATCCGGCTGGCAG--------------------------------------------------------------------------------------------------------------------------------------------------------------------------------------------------------   0.097   0.038   0.038
----------AGCACCTTGATCCGGCTGGCAG--------------------------------------------------------------------------------------------------------------------------------------------------------------------------------------------------------   0.097   0.341   0.341
--------TGAGCACCTTGATCCGGCTGG-----------------------------------------------------------------------------------------------------------------------------------------------------------------------------------------------------------   1.117   0.227   0.113
--------TGAGCACCTTGATCCGGCTGGCAG--------------------------------------------------------------------------------------------------------------------------------------------------------------------------------------------------------   0.000   0.152   0.152
--------TGAGCACCTTGATCCGGCTGGC----------------------------------------------------------------------------------------------------------------------------------------------------------------------------------------------------------   3.303   1.023   1.242
-------GTGAGCACCTTGATCCGGC--------------------------------------------------------------------------------------------------------------------------------------------------------------------------------------------------------------   0.000   0.076   0.076
------TGTGAGCACCTTGATCCGGC--------------------------------------------------------------------------------------------------------------------------------------------------------------------------------------------------------------   0.049   0.038   0.038
-----TTGTGAGCACCTTGATCCGGC--------------------------------------------------------------------------------------------------------------------------------------------------------------------------------------------------------------   1.894   5.797   3.614
-----TTGTGAGCACCTTGATCCGGCT-------------------------------------------------------------------------------------------------------------------------------------------------------------------------------------------------------------   0.437   0.076   0.076
----TTTGTGAGCACCTTGATCCGGC--------------------------------------------------------------------------------------------------------------------------------------------------------------------------------------------------------------   0.729   1.174   1.883
----TTTGTGAGCACCTTGATCCGG---------------------------------------------------------------------------------------------------------------------------------------------------------------------------------------------------------------   0.826   0.833   0.904

GCAGTTTGTGAGCACCTTGATCCGGCTGGCAGTATGGGTCAGCATCCTGCTGATGTTTATCTCGCTCAAGGTAATCAAGTGGACAGACGCGCTAACCATTTTCCCCACTGCCAAGGCA           G  C                A        TATA  ACA      G                            AT                   GCTT     ACT
                                                                                                                      GCGGACTGCGT GA GAGCTCCTGAAGCTGG GCTGCGCC    CA   GTGAGT GGTGTGACAACATTGTGGGTGTGGGCTG  TTGCCAGCAAGTCCTTGT    GCCCC   A
                                                                                                                      CGCCTGACGTA CT CTCGAGGACTTCGACC CGACGTGG    GT   CGCTCA CCGTACTGTTGTAACACCCACACCCGAC  AACGGTCGTTCAGGAACA    CGGGG   /
----------------------------------------------------------------------------------------------------------------------           G  A                C        TAAC  CCC      G                            CT                   ----     AAA

--------------------------------------------------------------------------------------------------------------------------TGACGTAGCTACTCGAGGACT-----------------------------------------------------------------------------------------   0.777   0.189   0.264 miR_t119*
---------------------------------------------------------------------------------------------------------------------------GACGTAGCTACTCGAGGACT-----------------------------------------------------------------------------------------   0.389   0.227   0.227
---------------------------------------------------------------------------------------------------------------------------GACGTAGCTACTCGAGGACTT----------------------------------------------------------------------------------------   4.468   2.538   1.393
--------------------------------------------------------------------------------------------------------------------------TGACGTAGCTACTCGAGGACTT----------------------------------------------------------------------------------------   0.049   0.152   0.152
------------------------------------------------------------------------------------------------------------------------------------------GGACTTCGACCCCGACGTGGT-------------------------------------------------------------------------   0.097   0.076   0.076
--------------------------------------------------------------------------------------------------------------------------------------------------------------------CCCCGCTCAGCCGTACTGTT------------------------------------------------   0.000   0.076   0.076
-------------------------------------------------------------------------------------------------------------------------------------------------------------------TCCCCGCTCAGCCGTACTGTT------------------------------------------------   0.049   0.038   0.151
-------------------------------------------------------------------------------------------------------------------------------------------------------------------------------------------ACCCACACCCGACCTAACGG-----------------------   0.000   0.076   0.076
-------------------------------------------------------------------------------------------------------------------------------------------------------------------------------------------ACCCACACCCGACCTAACGGT----------------------   1.068   0.265   0.265
```

miRNA: miR_t95 chromosome_16 Start: 5542812 End: 5543065 Condition: HS-N

```
-----------------------------------------------------TCGGGTCCTCGCAGCCAGGCC------------------------------------------------------------------------------------------------------   0.000   0.076   0.075
---------------------------------------------------TGTCGGGTCCTCGCAGCCAGG--------------------------------------------------------------------------------------------------------   0.049   0.114   0.301 miR_t95*
------------------------------------------------TGCTGTCGGGTCCTCGCAGC------------------------------------------------------------------------------------------------------------   0.049   0.038   0.038
------------------------------------------------TGCTGTCGGGTCCTCGC---------------------------------------------------------------------------------------------------------------   0.000   0.038   0.038
--------------------------------------------TGCGTGCTGTCGGGTCCTCG----------------------------------------------------------------------------------------------------------------   0.486   0.530   0.565
--------------------------------------------TGCGTGCTGTCGGGTCCTCGC---------------------------------------------------------------------------------------------------------------   0.583   0.114   0.038
-------------------------------------------TTGCGTGCTGTCGGGTCCTCG----------------------------------------------------------------------------------------------------------------   0.146   0.644   0.301
-------------------------------------------TTGCGTGCTGTCGGGTCCTC-----------------------------------------------------------------------------------------------------------------   0.000   0.076   0.076
-----------------------------------------CATTGCGTGCTGTCGGGTCCTCG----------------------------------------------------------------------------------------------------------------   0.000   0.076   0.076
---------------TACCGCTGCGCTCCCGCCCA---------------------------------------------------------------------------------------------------------------------------------------------   0.000   0.114   0.114
----TTTGCCGAAGCTACCGCTGC--------------------------------------------------------------------------------------------------------------------------------------------------------   0.000   0.038   0.038

ATGGTTTGCCGAA                             A                               TA-       AGCGCCGGCCATGGTGCGCACCTCCTGTTCTGCCTCGGCCGAGGACGCCTCCCTGCCCGCGCGCCTTGGCCTGCACACCAGCGTC      C
             GCTACCGCTGCGCTCCCGCCCAGACGCGC TTGCGTGCTGTCGGGTCCTCGCAGCCAGGCC   GCGCACC                                                                                           TTTGG A
             CGATGGCGACGCGAGGGCGGGTCTGCGCG GACGCACGACAGCCCAGGAGCGTCGGTCCGG   CGTGTGG                                                                                           AGACC G
-------------                             G                               TAC       -------------------------------------------------------------------------------------      G

-----------------GGCGACGCGAGGGCGGGTCT-------------------------------------------------------------------------------------------------------------------------------------------   0.243   0.076   0.038
------------------------CGAGGGCGGGTCTGCGCGGGAC----------------------------------------------------------------------------------------------------------------------------------   0.000   0.038   0.038
----------------------------------------------GCACGACAGCCCAGGAGCGT--------------------------------------------------------------------------------------------------------------   0.097   0.114   0.038
---------------------------------------------CGCACGACAGCCCAGGAGCGT--------------------------------------------------------------------------------------------------------------   11.753   7.388   12.425
-------------------------------------------------CGACAGCCCAGGAGCGT--------------------------------------------------------------------------------------------------------------   0.000   0.076   0.076
-------------------------------------------------CGACAGCCCAGGAGCGTCGG-----------------------------------------------------------------------------------------------------------   0.049   0.871   0.188
---------------------------------------------------ACAGCCCAGGAGCGTCGGT----------------------------------------------------------------------------------------------------------   0.000   0.038   0.038
-------------------------------------------------CGACAGCCCAGGAGCGTCGGT----------------------------------------------------------------------------------------------------------   1.943   35.461   11.559 miR_t95
-------------------------------------------------CGACAGCCCAGGAGCGTCGGTC---------------------------------------------------------------------------------------------------------   0.000   0.038   0.038
---------------------------------------------------ACAGCCCAGGAGCGTCGGTCCGGT-----------------------------------------------------------------------------------------------------   0.000   0.038   0.038
-----------------------------------------------------AGCCCAGGAGCGTCGGTCCGGT-----------------------------------------------------------------------------------------------------   0.000   1.515   0.791
----------------------------------------------------CAGCCCAGGAGCGTCGGTCCGGT-----------------------------------------------------------------------------------------------------   0.000   0.114   0.113
-------------------------------------------------------CCCAGGAGCGTCGGTCCGGT-----------------------------------------------------------------------------------------------------   0.049   0.265   0.075
--------------------------------------------------------CCAGGAGCGTCGGTCCGGT-----------------------------------------------------------------------------------------------------   0.000   0.114   0.114
------------------------------------------------------GCCCAGGAGCGTCGGTCCGGT-----------------------------------------------------------------------------------------------------   0.243   2.538   1.130
```

miRNA: miR_t101 chromosome_13 Start: 2301411 End: 2301724 Condition: HS-N

```
-----------------------------------------------------------------------------------------------------------TGTGACGTGGTTCTTTCATGTG-------------------------------------   0.049   0.114   0.114
```

```
-----------------------------------------------------------------------------------------------------------TGTGACGTGGTTCTTTCATG---------------------------------------      5.342      2.576      1.092
--------------------------------------------------------------------------------------------------------TTGTGTGACGTGGTTCTTT-------------------------------------------      0.389      0.114      0.301
--------------------------------------------------------------------------------------------------------TTGTGTGACGTGGTTCTTTC------------------------------------------      0.437      0.341      0.452
--------------------------------------------------------------------------------------------------------TTGTGTGACGTGGTTCTTTCA-----------------------------------------      2.088      0.644      0.339
-------------------------------------------------------------------------------------------------------TTTGTGTGACGTGGTTCTTT-------------------------------------------     16.270      2.879      4.292
-------------------------------------------------------------------------------------------------------TTTGTGTGACGTGGTTC----------------------------------------------      0.097      0.114      0.114
-------------------------------------------------------------------------------------------------------TTTGTGTGACGTGGTTCTTTCA-----------------------------------------      0.680      0.038      0.151
-------------------------------------------------------------------------------------------------------TTTGTGTGACGTGGTTCTTTC------------------------------------------      0.583      0.227      0.301
-------------------------------------------------------------------------------------------------------TTTGTGTGACGTGGTTCTT--------------------------------------------      1.554      0.189      0.602
-------------------------------------------------------------------------------------------------------TTTGTGTGACGTGGTTCT---------------------------------------------      0.243      0.152      0.075
------------------------------------------------------------------------------------------------------GTTTGTGTGACGTGGTTCTT--------------------------------------------      0.971      0.038      0.226
-----------------------------------------------------------------------------------------------------TGTTTGTGTGACGTGGT------------------------------------------------      0.631      0.796      1.054
-----------------------------------------------------------------------------------------------------TGTTTGTGTGACGTGGTT-----------------------------------------------      0.486      0.417      0.301
-----------------------------------------------------------------------------------------------------TGTTTGTGTGACGTGGTTCTT--------------------------------------------    102.042     16.670     24.737
-----------------------------------------------------------------------------------------------------TGTTTGTGTGACGTGGTTCTTT-------------------------------------------     15.250      4.092      7.455
-----------------------------------------------------------------------------------------------------TGTTTGTGTGACGTGGTTC----------------------------------------------      1.894      0.720      0.602
-----------------------------------------------------------------------------------------------------TGTTTGTGTGACGTGGTTCT---------------------------------------------      7.334      0.947      1.431
----------------------------------------------------------------------------------------------------CTGTTTGTGTGACGTGGTTCT---------------------------------------------      0.146      0.341      0.301
----------------------------------------------------------------------------------------------------CTGTTTGTGTGACGTGGTTCTT--------------------------------------------      0.000      0.038      0.038
--------------------------------------------------------------------------------------------------AGCTGTTTGTGTGACGTGGT------------------------------------------------      0.097      0.152      0.152
-------------------------------------------------------------------------------------------------AAGCTGTTTGTGTGACGTGG-------------------------------------------------      0.291      0.189      0.189
------------------------------------------------------------------------------------------------CAAGCTGTTTGTGTGACGTGG-------------------------------------------------      0.971      1.023      0.113
-----------------------------------------------------------------------------------------------TCAAGCTGTTTGTGTGACGTG--------------------------------------------------      1.020      0.114      0.114
-----------------------------------------------------------------------------------------------TCAAGCTGTTTGTGTGACGTGG-------------------------------------------------      5.391      1.174      0.452
-----------------------------------------------------------------------------------------------TCAAGCTGTTTGTGTGACG----------------------------------------------------      0.146      0.076      0.076
---------------------------------------------------------------------------------------------TGTCAAGCTGTTTGTGTGACGTGG-------------------------------------------------      0.049      0.152      0.152
-----------------------------------------------------------------------------TTTGAAGCTGAGCCGCTGTC---------------------------------------------------------------------      0.000      0.038      0.038
-------------------------------------------------------------------------TGGCTTTGAAGCTGAGCCGC-------------------------------------------------------------------------      0.340      0.038      0.038
--------------------------------------------------------------------TGGTGTGGCTTTGAAGCTGAGC----------------------------------------------------------------------------      0.000      0.038      0.038
-----------------------------TCATTTGTGCGTACTGAAT----------------------------------------------------------------------------------------------------------------------      1.894      0.644      0.866
-----------------------------TCATTTGTGCGTACTGAATT---------------------------------------------------------------------------------------------------------------------      1.360      0.606      0.188
---------------------------TATCATTTGTGCGTACT--------------------------------------------------------------------------------------------------------------------------      0.000      0.114      0.114
---------------------------TATCATTTGTGCGTACTGAA-----------------------------------------------------------------------------------------------------------------------     10.199      3.902      3.727
---------------------------TATCATTTGTGCGTACTGAATTAT-------------------------------------------------------------------------------------------------------------------      0.000      0.076      0.076
---------------------------TATCATTTGTGCGTACTGA------------------------------------------------------------------------------------------------------------------------      1.214      1.515      1.017
---------------------------TATCATTTGTGCGTACTG-------------------------------------------------------------------------------------------------------------------------      0.097      0.227      0.151
---------------------------TATCATTTGTGCGTACTGAATT---------------------------------------------------------------------------------------------------------------------      0.243      0.076      0.076
---------------------------TATCATTTGTGCGTACTGAAT----------------------------------------------------------------------------------------------------------------------     43.080      6.933      4.593
--------------------------TTATCATTTGTGCGTACTGAA-----------------------------------------------------------------------------------------------------------------------      0.971      0.227      0.715
----------------------TCAATTATCATTTGTGCGTAC---------------------------------------------------------------------------------------------------------------------------      0.194      0.152      0.075
----------------GGGGCATCAATTATCATTTG----------------------------------------------------------------------------------------------------------------------------------      1.360      0.644      2.560
---------------TGGGGCATCAATTATCATT------------------------------------------------------------------------------------------------------------------------------------      0.291      0.076      0.264
---------------TGGGGCATCAATTATCATTT-----------------------------------------------------------------------------------------------------------------------------------      0.437      0.152      0.113
---------------TGGGGCATCAATTATCATTTGT---------------------------------------------------------------------------------------------------------------------------------      0.243      0.076      0.151
---------------TGGGGCATCAATTATCATTTG----------------------------------------------------------------------------------------------------------------------------------     65.033     53.647    116.153 miR_t101
-------------AGTGGGGCATCAATTATCATT------------------------------------------------------------------------------------------------------------------------------------      0.000      0.038      0.038
-----------CGAGTGGGGCATCAATTATCA--------------------------------------------------------------------------------------------------------------------------------------      0.000      0.076      0.076

-------                                            GTACGTACTGAGTTA               A                                                 C  A G--                       TTT
       CCGGCGAGTGGGGCATCAATTATCATTTGTGCGTACTGAATTAT               TGTGGTGTGGCTTTG AGCTGAGCCGCTGTCAAGCTGTTTGTGTGACGTGGTTCTTTCATGTGTA CA G   GCGTGATGGCTCGCAGTCTTTTG   A
       GGCCGCTCACCCCGTAGTTAATAGTAAACACGCATGACTTAGTA               ACATCACACCGAAAC TCGACTCGGCGACAGTTCGACAAACACACTGCACCAAGAAAGTACACAT GT C   TGCACTACCGAGCGTCAGAAAAT   /
CTCACGT                                            ATTACA---------               C                                                 A  A ACA                       GGG

----CGTGGCCGCTCACCCCG-------------------------------------------------------------------------------------------------------------------------------------------------      0.049      0.796      1.770
----CGTGGCCGCTCACCCCGT------------------------------------------------------------------------------------------------------------------------------------------------      0.583      4.887      7.568
----CGTGGCCGCTCACCCCGTAG----------------------------------------------------------------------------------------------------------------------------------------------      1.263      0.530      2.560
----CGTGGCCGCTCACCCCGTAGT---------------------------------------------------------------------------------------------------------------------------------------------     55.271     40.008     54.631
-----GTGGCCGCTCACCCCGTAGT---------------------------------------------------------------------------------------------------------------------------------------------      0.437      0.038      0.377
-------GGCCGCTCACCCCGTAGTT--------------------------------------------------------------------------------------------------------------------------------------------      2.963      0.417      0.602
-----GTGGCCGCTCACCCCGTAGTT--------------------------------------------------------------------------------------------------------------------------------------------     10.345      1.970      2.221
------TGGCCGCTCACCCCGTAGTT--------------------------------------------------------------------------------------------------------------------------------------------      1.311      0.493      0.941
----CGTGGCCGCTCACCCCGTAGTT--------------------------------------------------------------------------------------------------------------------------------------------      6.945     12.578     14.609
-------GGCCGCTCACCCCGTAGTTA-------------------------------------------------------------------------------------------------------------------------------------------      1.311      0.189      1.017
------TGGCCGCTCACCCCGTAGTTA-------------------------------------------------------------------------------------------------------------------------------------------      0.389      0.227      0.339
-------GGCCGCTCACCCCGTAGTTAA------------------------------------------------------------------------------------------------------------------------------------------      1.360      0.038      0.527
----CGTGGCCGCTCACCCCGTAGTTAAT-----------------------------------------------------------------------------------------------------------------------------------------      0.000      0.076      0.076
---------CCGCTCACCCCGTAGTTAAT-----------------------------------------------------------------------------------------------------------------------------------------      2.234      0.947      1.017
----------CGCTCACCCCGTAGTTAAT-----------------------------------------------------------------------------------------------------------------------------------------      0.097      0.152      0.565
------TGGCCGCTCACCCCGTAGTTAAT-----------------------------------------------------------------------------------------------------------------------------------------      0.340      0.152      0.489
--------GCCGCTCACCCCGTAGTTAAT-----------------------------------------------------------------------------------------------------------------------------------------      9.325      4.471      8.886
-------GGCCGCTCACCCCGTAGTTAAT-----------------------------------------------------------------------------------------------------------------------------------------     50.171     13.866     23.795
-----------GCTCACCCCGTAGTTAATAG---------------------------------------------------------------------------------------------------------------------------------------      0.049      0.076      0.075
------------CTCACCCCGTAGTTAATAGT--------------------------------------------------------------------------------------------------------------------------------------      0.049      0.114      0.038
-----------GCTCACCCCGTAGTTAATAGT--------------------------------------------------------------------------------------------------------------------------------------     17.339      3.751      7.003
----------CGCTCACCCCGTAGTTAATAGT--------------------------------------------------------------------------------------------------------------------------------------      1.020      0.076      0.264
-------------TCACCCCGTAGTTAATAGT--------------------------------------------------------------------------------------------------------------------------------------      0.534      0.114      0.226
-------------------------TAATAGTAAACACGCATGACT------------------------------------------------------------------------------------------------------------------------      0.486      0.076      0.076
--------------------------AATAGTAAACACGCATGACT------------------------------------------------------------------------------------------------------------------------      0.194      0.038      0.038
----------------------------TAGTAAACACGCATGACT------------------------------------------------------------------------------------------------------------------------      0.000      0.076      0.076
---------------------------ATAGTAAACACGCATGACTT-----------------------------------------------------------------------------------------------------------------------      0.146      0.076      0.076
--------------------------------------------------------------------------------CCTCGACTCGGCGACAGTT-------------------------------------------------------------------      0.000      0.038      0.038
-------------------------------------------------------------------------------------------CGACAGTTCGACAAACACA--------------------------------------------------------      0.097      0.379      0.113
-------------------------------------------------------------------------------------------CGACAGTTCGACAAACACACT------------------------------------------------------      6.508      3.486      4.255
--------------------------------------------------------------------------------------------------TCGACAAACACACTGCACCAA-----------------------------------------------      0.389      0.493      0.038
----------------------------------------------------------------------------------------------------GACAAACACACTGCACCAAGA---------------------------------------------      0.146      0.038      0.301
---------------------------------------------------------------------------------------------------CGACAAACACACTGCACCAAGA---------------------------------------------      0.291      0.114      0.114
---------------------------------------------------------------------------------------------------------ACACACTGCACCAAGAAAGT-----------------------------------------      0.874      0.909      0.866
--------------------------------------------------------------------------------------------------------------CTGCACCAAGAAAGTACACAT-----------------------------------      0.097      0.114      0.075
```

miRNA: miR_t103 chromosome_4 Start: 259096 End: 259431 Condition: HS-N

```
-----------------------------------------------------------------------------------------------------------------------------------------------------------------------------------------AACACAGAGCATCACCACGG--------------------------      0.000      0.076      0.075
-------------------------------------------------------------------------------------------------------------------------------------------------------------------------TGGTCGGCGGCAGCCCAACACAG---------------------------------------      0.097      0.038      0.038
----------------------------------------------------------------------------------------------------------------------------------------------------------------------TCATGGTCGGCGGCAGCCC----------------------------------------------      0.097      0.038      0.113
----------------------------------------------------------------------------------------------------------------------------------------------------------------------TCATGGTCGGCGGCAGCCCAA--------------------------------------------      0.874      0.644      0.941
----------------------------------------------------------------------------------------------------------------------------------------------------------------------TCATGGTCGGCGGCAGCC-----------------------------------------------      0.000      0.038      0.038
---------------------------------------------------------------------------------------------------------------------------------------------------------------------TTCATGGTCGGCGGCAGC------------------------------------------------      0.097      0.341      0.038
-----------------------------------------------------------------------------------------------------------------------------------------------------------------GGGCTTCATGGTCGGCGGC---------------------------------------------------      0.437      0.530      3.878
-----------------------------------------------------------------------------------------------------------------------------------------------------------------GGGCTTCATGGTCGGCGGCAG-------------------------------------------------      0.000      0.038      0.038
-----------------------------------------------------------------------------------------------------------------------------------------------------------------GGGCTTCATGGTCGGCGGCA--------------------------------------------------      0.194      0.227      1.242
----------------------------------------------------------------------------------------------------------------------------------------------------------------TGGGCTTCATGGTCGGCGG----------------------------------------------------      0.340      0.038      0.113
----------------------------------------------------------------------------------------------------------------------------------------------------------------TGGGCTTCATGGTCGGCGGCA--------------------------------------------------     31.958     25.876     50.716
----------------------------------------------------------------------------------------------------------------------------------------------------------------TGGGCTTCATGGTCGGCGGCAG-------------------------------------------------      0.971      0.455      0.489
----------------------------------------------------------------------------------------------------------------------------------------------------------------TGGGCTTCATGGTCGGCGGCAGC------------------------------------------------      0.097      0.076      0.264
----------------------------------------------------------------------------------------------------------------------------------------------------------------TGGGCTTCATGGTCGGCGGC---------------------------------------------------     35.212     52.662    218.751
---------------------------------------------------------------------------------------------------------------------------------------------------------------CTGGGCTTCATGGTCGGCGGC---------------------------------------------------      0.291      0.265      1.092
--------------------------------------------------------------------------------------------------------------------------------------------------------------TCTGGGCTTCATGGTCGGCGG----------------------------------------------------     51.677      3.220      3.690
--------------------------------------------------------------------------------------------------------------------------------------------------------------TCTGGGCTTCATGGTCGGCGGCA--------------------------------------------------      1.311      0.189      0.377
--------------------------------------------------------------------------------------------------------------------------------------------------------------TCTGGGCTTCATGGTCGGCGGC---------------------------------------------------    290.001     66.755    172.554
--------------------------------------------------------------------------------------------------------------------------------------------------------------TCTGGGCTTCATGGTCGGC------------------------------------------------------      1.603      1.288      0.979
--------------------------------------------------------------------------------------------------------------------------------------------------------------TCTGGGCTTCATGGTCGGCG-----------------------------------------------------      1.360      0.076      0.076
----------------------------------------------------------------------------------------------------------------------------------------------------------CGGGTCTGGGCTTCATGGTCG--------------------------------------------------------      0.097      0.038      0.038
--------------------------------------------------------------------------------------------------------------------------------------------------------GGCGGGTCTGGGCTTCATGG-----------------------------------------------------------      0.243      0.038      0.640
-------------------------------------------------------------------------------------------------------------------------------------------------------TGGCGGGTCTGGGCTTCATG------------------------------------------------------------      0.243      0.341      0.301
-------------------------------------------------------------------------------------------------------------------------------------------------------TGGCGGGTCTGGGCTTCATGG-----------------------------------------------------------     26.518     17.844     46.762
------------------------------------------------------------------------------------------------------------------------------------------AGGCTTGCGATTGTGGCGGG-------------------------------------------------------------------------      0.194      0.265      0.414
-----------------------------------------------------------------------------------------------------------------------------------------CAGGCTTGCGATTGTGGCGGG-------------------------------------------------------------------------      0.243      0.152      0.226
----------------------------------------------------------------------------------------------------------------------------------------TCAGGCTTGCGATTGTGGCG---------------------------------------------------------------------------      0.583      0.152      0.075
----------------------------------------------------------------------------------------------------------------------------------------TCAGGCTTGCGATTGTGG-----------------------------------------------------------------------------      0.097      0.114      0.114
----------------------------------------------------------------------------------------------------------------------------------------TCAGGCTTGCGATTGTGGCGG--------------------------------------------------------------------------      6.460      4.812      1.995
----------------------------------------------------------------------------------------------------------------------------------------TCAGGCTTGCGATTGTGGCGGG-------------------------------------------------------------------------     38.612     22.012     16.981
--------------------------------------------------------------------------------------------------------------------------------------TGTCAGGCTTGCGATTGTGGCG---------------------------------------------------------------------------      0.340      0.303      0.303
--------------------------------------------------------------------------------------------------------------------------------------TGTCAGGCTTGCGATTGTGGCGGG-------------------------------------------------------------------------      0.000      0.038      0.038
--------------------------------------------------------------------------------------------------------------------------------------TGTCAGGCTTGCGATTGTG------------------------------------------------------------------------------      0.146      0.114      0.075
--------------------------------------------------------------------------------------------------------------------------------------TGTCAGGCTTGCGATTGTGGC----------------------------------------------------------------------------      1.408      5.759      9.902
--------------------------------------------------------------------------------------------------------------------------------------TGTCAGGCTTGCGATTGTGG-----------------------------------------------------------------------------     10.734      7.047      6.438
-----------------------------------------------------------------------------------------------------------------------------------TACGTGTCAGGCTTGCGATTG-------------------------------------------------------------------------------      1.700      2.690      2.711
----------------------------------------------------------------------------------------------------------------------------------ATACGTGTCAGGCTTGCGATTG-------------------------------------------------------------------------------      0.049      0.303      0.303
------------------------------------------------------------------------------------------------------------------------------AGGATACGTGTCAGGCTTGCG------------------------------------------------------------------------------------      0.000      0.189      0.113
----------------------------------------------------------------------------------------------------------------------------CCAGGATACGTGTCAGGC-----------------------------------------------------------------------------------------      0.049      0.076      0.076
--------------------------------------------------------------------------------------------------------------------------TCCCAGGATACGTGTCAGGC-----------------------------------------------------------------------------------------      0.000      0.076      0.076
--------------------------------------------------------------------------------------------------------------------------TCCCAGGATACGTGTCAGGCTT---------------------------------------------------------------------------------------      0.097      0.076      0.076
--------------------------------------------------------------------------------------------------------------------------TCCCAGGATACGTGTCAGGCT----------------------------------------------------------------------------------------      0.826      0.530      0.377
-------------------------------------------------------------------------------------------------------------------------TTCCCAGGATACGTGTCAGGCT----------------------------------------------------------------------------------------      0.291      0.076      0.076
-------------------------------------------------------------------------------------------------------------------------TTCCCAGGATACGTGTCAGGC-----------------------------------------------------------------------------------------      2.671      3.940      2.334
-------------------------------------------------------------------------------------------------------------------------TTCCCAGGATACGTGTCAGG------------------------------------------------------------------------------------------      0.583      0.493      0.226
------------------------------------------------------------------------------------------------------------------------TTTCCCAGGATACGTGTCAGGC-----------------------------------------------------------------------------------------      0.146      0.152      0.152
------------------------------------------------------------------------------------------------------------------------TTTCCCAGGATACGTGTCAGG------------------------------------------------------------------------------------------     24.381      5.493      9.187
-----------------------------------------------------------------------------------------------------------------------CTTTCCCAGGATACGTGTCAG-------------------------------------------------------------------------------------------      0.000      0.038      0.038
-----------------------------------------------------------------------------------TCAGGACTCCAGCCCAGCGAC-------------------------------------------------------------------------------------------------------------------------------     10.588      3.296      2.485
-----------------------------------------------------------------------------------TCAGGACTCCAGCCCAGC----------------------------------------------------------------------------------------------------------------------------------      0.000      0.038      0.038
-----------------------------------------------------------------------------------TCAGGACTCCAGCCCAGCGACA------------------------------------------------------------------------------------------------------------------------------      0.146      0.076      0.038
-----------------------------------------------------------------------------------TCAGGACTCCAGCCCAGCGA--------------------------------------------------------------------------------------------------------------------------------      0.389      0.038      0.038
------------------------------------------------------------TGAACCTCCTCAAAACCCACA------------------------------------------------------------------------------------------------------------------------------------------------------      0.631      0.038      0.188
-------------------------------------------------TGCAAACGGACTGAACCTCC------------------------------------------------------------------------------------------------------------------------------------------------------------------      0.486      0.189      0.075
---------------------------------------------AAACTGCAAACGGACTGAAC----------------------------------------------------------------------------------------------------------------------------------------------------------------------      0.000      0.038      0.075
-------------------------------------------ACAAACTGCAAACGGACTGAAC----------------------------------------------------------------------------------------------------------------------------------------------------------------------      0.000      0.076      0.076
-----------------------------------------CAACAAACTGCAAACGGACTG-------------------------------------------------------------------------------------------------------------------------------------------------------------------------      0.097      0.114      0.114
---------------------------------------TACAACAAACTGCAAACGGAC---------------------------------------------------------------------------------------------------------------------------------------------------------------------------      0.243      0.303      0.113
--------------------------------------ATACAACAAACTGCAAACGGA----------------------------------------------------------------------------------------------------------------------------------------------------------------------------      0.000      0.076      0.076
--------------------------------TCGACAATACAACAAACTGC-----------------------------------------------------------------------------------------------------------------------------------------------------------------------------------      2.137      0.606      0.075
--------------------------------TCGACAATACAACAAACTG------------------------------------------------------------------------------------------------------------------------------------------------------------------------------------      0.000      0.038      0.038
-----------------------------TGGTCGACAATACAACAAAC--------------------------------------------------------------------------------------------------------------------------------------------------------------------------------------      0.097      0.189      0.038
-----------------------------TGGTCGACAATACAACAAACTGC-----------------------------------------------------------------------------------------------------------------------------------------------------------------------------------      0.049      0.076      0.075
-----------------------------TGGTCGACAATACAACAAACTG------------------------------------------------------------------------------------------------------------------------------------------------------------------------------------      0.534      0.493      0.226
-------------TCAATCAGGCAGCGGGTGGT--------------------------------------------------------------------------------------------------------------------------------------------------------------------------------------------------------      0.000      0.114      0.301
-------------TCAATCAGGCAGCGGGTGGTC-------------------------------------------------------------------------------------------------------------------------------------------------------------------------------------------------------      0.049      0.379      0.489
-------------TCAATCAGGCAGCGGGTGGTCG------------------------------------------------------------------------------------------------------------------------------------------------------------------------------------------------------      0.534      0.379      0.226
-------------TCAATCAGGCAGCGGGTGG---------------------------------------------------------------------------------------------------------------------------------------------------------------------------------------------------------      0.097      0.227      0.715
----------ACGTCAATCAGGCAGCGGG------------------------------------------------------------------------------------------------------------------------------------------------------------------------------------------------------------      0.000      0.038      0.038
---------TACGTCAATCAGGCAGCGGG------------------------------------------------------------------------------------------------------------------------------------------------------------------------------------------------------------      0.146      0.076      0.264
----TCAACTACGTCAATCAGGCAGC---------------------------------------------------------------------------------------------------------------------------------------------------------------------------------------------------------------      0.194      0.227      0.227

CGCGTCAACTACGTCAATCAGGCAGCGGGTGGTCGACAATACAACAAACTGCAAACGGACTGAACCTCCTCAAAACCCACACCTCAGGACTCCAGCCCAGCGACAATCAGCGACATGCCCTTTCCCA                                                             A                            C  ----    CT
                                                                                                                               GGATACGTGTCAGGCTTGCGATTGTGGCGGGTCTGGGCTTCATGGTCGGCGGCAGCCCAAC CAGAGCATCACCACGGTGATGGGCCTCG AC    CGGC  T
                                                                                                                               CCTATGCACAGTCCGAACGCTAACACCGCCCAGACCCGAAGTACCAGCCGCCGTCGGGTTG GTCTCGTAGTGGTGCCGCTACCCGGAGC TG    GTCG  A
---------------------------------------------------------------------------------------------------------------------------NNNN                                                             A                            A  CACA    AA
```

```
----------CCTATGCACAGTCCGAACGCT----------     0.146   0.152   0.452
----------CCCGAAGTACCAGCCGCCGT----------     0.097   0.076   0.076
----------CCGCCGTCGGGTTGAGT----------     0.000   0.038   0.038
----------CCAGCCGCCGTCGGGTTGAGT----------     0.777   0.455   0.941
----------CAGCCGCCGTCGGGTTGAGT----------     0.000   0.038   0.038
----------GCCGCCGTCGGGTTGAGTCT----------     0.631   0.076   0.151
----------CCGCCGTCGGGTTGAGTCT----------     0.049   0.038   0.038
----------GCCGCCGTCGGGTTGAGTCTC----------     0.000   0.038   0.113
----------GCCGCCGTCGGGTTGAGTCTCGT----------     0.049   0.076   0.076
----------CGCCGTCGGGTTGAGTCTCGT----------     1.068   0.114   0.151
----------CCGCCGTCGGGTTGAGTCTCGT----------     0.340   1.326   1.883
----------CCGTCGGGTTGAGTCTCGTAGT----------     0.146   0.114   0.114
----------CGGGTTGAGTCTCGTAGT----------     0.000   0.038   0.038
----------CGTCGGGTTGAGTCTCGTAGT----------     3.108   0.568   0.715
----------GGGTTGAGTCTCGTAGTGG----------     0.146   0.038   0.113
----------CGGGTTGAGTCTCGTAGTGG----------     0.729   1.326   4.066
----------CGGGTTGAGTCTCGTAGTGGT----------     36.378   169.540   192.057 miR_t103
----------GGGTTGAGTCTCGTAGTGGT----------     26.810   28.263   20.181
----------GGTTGAGTCTCGTAGTGGT----------     0.680   1.743   1.054
----------TCGGGTTGAGTCTCGTAGTGGT----------     0.243   0.038   0.038
----------TGAGTCTCGTAGTGGTGCCGCT----------     0.146   0.038   0.038
----------AGTCTCGTAGTGGTGCCGCT----------     0.049   0.038   0.038

miRNA: miR_t106 chromosome_16 Start: 4056061 End: 4056397 Condition: HS-N
----------------TGATGCGAGTAACCTGAAGC----------     10.199   32.317   32.719
-----------------GATGCGAGTAACCTGAAGC----------     0.194   0.076   0.866
----------------------CGAGTAACCTGAAGCTGCGGA----------     0.049   0.114   0.038
------------------------AGTAACCTGAAGCTGCGGATG----------     0.729   0.227   0.151
--------------------------TAACCTGAAGCTGCGGATGA----------     13.016   3.940   3.238
--------------------------TAACCTGAAGCTGCGGATGATG----------     10.879   22.504   19.503
--------------------------TAACCTGAAGCTGCGGATG----------     1.311   0.833   0.753
--------------------------TAACCTGAAGCTGCGGATGATGA----------     0.097   0.114   0.114
--------------------------TAACCTGAAGCTGCGGATGAT----------     0.243   0.076   0.226
---------------------------AACCTGAAGCTGCGGATGA----------     0.097   0.038   0.038
---------------------------AACCTGAAGCTGCGGATGATG----------     0.389   0.379   0.828
-----------------------------CCTGAAGCTGCGGATGATGAC----------     0.049   0.114   0.038
-------------------------------TGAAGCTGCGGATGATGACG----------     1.748   0.379   0.791
-------------------------------------TGCGGATGATGACGACGAA----------     0.049   0.076   0.076
-------------------------------------TGCGGATGATGACGACGAAG----------     1.506   0.341   0.452
-------------------------------------TGCGGATGATGACGACGAAGC----------     2.040   3.940   4.631
-------------------------------------------TGATGACGACGAAGCTGC---ACG----------     1.651   0.265   0.715
----------------------------------------------TGACGACGAAGCTGC---ACGC----------     0.049   0.038   0.075
----------------------------------------------TGACGACGAAGCTGC---ACGCCGC----------     0.000   0.038   0.038
----------ATGCAATGGGGGCACCGGCGG----------     0.000   0.038   0.038
----------TGCAATGGGGGCACCGGCGGC----------     1.117   0.303   0.264
----------TGCAATGGGGGCACCGGCGG----------     4.031   0.303   0.264
----------TGGGGGCACCGGCGGCGGCC----------     0.923   0.038   0.038
----------TGGGGGCACCGGCGGCGGC----------     0.000   0.076   0.038
----------CATGCACAAGGATCTCCGAACACC----------     0.000   0.303   0.303
----------CATGCACAAGGATCTCCGAAC----------     0.000   0.152   0.152
----------TGCACAAGGATCTCCGAACACC----------     12.045   6.138   3.238
----------TGCACAAGGATCTCCGAAC----------     0.194   0.114   0.075
----------TGCACAAGGATCTCCGAACAC----------     0.729   0.076   0.076

-----------               C                               ---              --------                                          - G     A                     A                     TAA
           CCAATGATGCGAGTAA CTGAAGCTGCGGATGATGACGACGAAGCTGC   ACGCCGCTCCAGCA       GTCCCGCCATGCAATGGGGGCACCGGCGGCGGCCGGGAGCC C CCGCC CCCATGCACAAGGATCTCCG ACACCTTGCTATCGGTGAGCC   G
           GGTTACTACGCTCATT GACTTCGACGCCTACTACTGCTGCTTCGACG   TGCGGCGAGGTCGT       CAGGGCGGTACGTTACCCCCGTGGCCGCCGCCGGCCCTCGG G GGCGG GGGTACGTGTTCCTAGAGGT TGTGGAACGATAGCCACTCGG   /
TCGTGGTCTAAC               A                               ACG              GCGTACGT                                          T -     C                     C                     TTT

----------CCTAGAGGTCTGTGGAACGAT----------     0.000   0.038   0.038
----------GGGTACGTGTTCCTAGAGGTCTGT----------     0.049   0.114   0.114
----------GTACGTGTTCCTAGAGGTCTGT----------     70.035   34.097   23.005 miR_t106
----------ACGTGTTCCTAGAGGTCTGT----------     26.081   3.713   2.523
----------GGTACGTGTTCCTAGAGGTCTGT----------     0.291   0.720   0.301
----------TACGTGTTCCTAGAGGTCTGT----------     10.539   2.766   2.636
----------CGTGTTCCTAGAGGTCTGT----------     2.866   8.297   4.857
----------TGTTCCTAGAGGTCTGT----------     0.000   0.038   0.038
----------GTACGTGTTCCTAGAGGTCTG----------     1.506   0.530   0.828
----------ACGTGTTCCTAGAGGTCTG----------     0.486   0.038   0.113
----------TACGTGTTCCTAGAGGTCTG----------     0.486   0.038   0.038
----------CGTGTTCCTAGAGGTCTG----------     0.049   0.076   0.339
----------GGTACGTGTTCCTAGAGGTCT----------     23.167   0.568   1.619
----------GGGTACGTGTTCCTAGAGGTCT----------     159.984   5.797   6.965
----------GGGTACGTGTTCCTAGAGGTC----------     0.680   1.402   1.393
----------CGGGTACGTGTTCCTAGAGGTC----------     0.000   0.189   0.189
----------GGGTACGTGTTCCTAGAGGT----------     0.146   0.076   0.151
----------GGCGGCGGGTACGTGTTCCT----------     0.000   0.114   0.114
----------CCTCGGTG-GGCGGCGGGTACGT----------     0.000   0.076   0.076
----------GCGGCGAGGTCGTGCGTACGTCAGGGCGGT----------     0.097   0.114   0.114
----------CGGCGAGGTCGTGCGTACGTCAGGGCGGT----------     0.000   0.076   0.640
----------ACTACTGCTGCTTCGACGACGT----------     6.751   0.114   0.038
----------ACTACTGCTGCTTCGACGACG----------     0.000   0.152   0.152
----------CGACGCCTACTACTGCTGCTT----------     1.651   0.417   0.339
----------CGACGCCTACTACTGCTGCT----------     0.971   0.303   0.113
----------AGACTTCGACGCCTACTACT----------     0.194   0.076   0.038
----------CATTAGACTTCGACGCCTACT----------     0.389   0.038   0.038
----------GCTCATTAGACTTCGACGCCT----------     1.846   0.114   0.113
------------GGTTACTACGCTCATTAGACTT----------     1.651   0.152   0.602
------------GGTTACTACGCTCATTAGACT----------     3.691   1.819   1.506
-----------CGGTTACTACGCTCATTAGACT----------     0.049   0.265   0.265
-----GTCTAACGGTTACTACGCT----------     0.000   0.038   0.038
----GGTCTAACGGTTACTACGCT----------     7.188   1.478   1.280

miRNA: miR_t119 chromosome_8 Start: 1981962 End: 1982308 Condition: HS-N
------TTTGTGAGC------ACCTTGATCCGG--------------------     0.826   0.833   0.904
------TTTGTGAGC------ACCTTGATCCGGC-------------------     0.729   1.174   1.883
-------TTGTGAGC------ACCTTGATCCGGC-------------------     1.894   5.797   3.614
-------TTGTGAGC------ACCTTGATCCGGCT------------------     0.437   0.076   0.076
--------TGTGAGC------ACCTTGATCCGGC-------------------     0.049   0.038   0.038
---------GTGAGC------ACCTTGATCCGGC-------------------     0.000   0.076   0.076
----------TGAGC------ACCTTGATCCGGCTGGC---------------     3.303   1.023   1.242
----------TGAGC------ACCTTGATCCGGCTGGCAG-------------     0.000   0.152   0.152
----------TGAGC------ACCTTGATCCGGCTGG----------------     1.117   0.227   0.113
------------AGC------ACCTTGATCCGGCTGGCAG-------------     0.097   0.341   0.341
-------------GC------ACCTTGATCCGGCTGGCAG-------------     0.097   0.038   0.038
---------------------ACCTTGATCCGGCTGGCAG-------------     0.097   0.114   0.226
---------------------ACCTTGATCCGGCTGGCAGTATG---------     0.000   0.038   0.038
----------------------CCTTGATCCGGCTGGCAG-------------     0.000   0.038   0.038
-----------------------CTTGATCCGGCTGGCAGTATGGG-------     0.000   0.038   0.188
------------------------TTGATCCGGCTGGCAGTATGG--------     2.671   2.311   8.246
------------------------TTGATCCGGCTGGCAGTATG---------     0.340   1.137   0.565
------------------------TTGATCCGGCTGGCAGTATGGGT------     1.554   0.379   0.379
------------------------TTGATCCGGCTGGCAGTATGGG-------     50.462   46.297   68.600 miR_t119
------------------------TTGATCCGGCTGGCAGTAT----------     0.000   0.038   0.038
-------------------------TGATCCGGCTGGCAGTATG---------     0.049   0.038   0.038
-------------------------TGATCCGGCTGGCAGTATGGGT------     1.263   0.038   0.038
-------------------------TGATCCGGCTGGCAGTATGG--------     0.000   0.076   0.339
-------------------------TGATCCGGCTGGCAGTATGGG-------     6.460   4.433   7.116

-  A-      GA  ------       TCCG  T  CA   T G      AT
 GC  GTTTGT  GC      ACCTTGA    GC GG  GTA G GTCAGC  C
 CG  CAGACA  TG      TGGAACT    CG TC  TAT T TAGTCG  /
T  CG      GG  AACTAA       ----  C  --   T G      TT

--------ACAGGTGAACTAATGGAACT----CGCT-----------------     0.000   0.076   0.038
-----------GGTGAACTAATGGAACT----CGCT-----------------     0.923   0.379   0.075
----------AGGTGAACTAATGGAACT----CGCT-----------------     0.340   0.114   0.038

miRNA: miR_t121 chromosome_7 Start: 77616 End: 78054 Condition: HS-N
----------TCAACATCAACACGGTGCTGA----------     0.000   0.038   0.038
----------TCTGC------TCGTGGTGGTGATCG----------     0.000   0.038   0.038
----------TGC------TCGTGGTGGTGATCGTGC----------     0.049   0.038   0.113
----------TCGTGGTGGTGATCGTGCTCT----------     0.097   0.076   0.075
----------TGGTGGTGATCGTGCTCTTG----------     0.049   0.341   0.341
----------TGGTGATCGTGCTCTTGTTG----------     0.291   0.227   0.151
----------TGCTCTTGTTGTGGATTGCCG----------     0.000   0.038   0.038
----------TTGTGGATTGCCGCATGGAAGG----------     0.000   0.038   0.113
----------TGTGGATTGCCGCATGGAAGG----------     0.000   0.038   0.075
----------TCAAGTCGGCACTCCAGAC----------     0.000   0.114   0.114
----------TCAAGTCGGCACTCCAGACAG----------     0.146   0.076   0.075
----------AAGTCGGCACTCCAGACAGTCT----------     0.000   0.038   0.075
----------TCGGCACTCCAGACAGTCTTC----------     10.831   1.970   8.622
----------TCGGCACTCCAGACAGTCT----------     0.049   0.076   0.113
----------TCGGCACTCCAGACAGT----------     0.000   0.303   0.452

----------------------------------------------CA                            A       -------                                    G   G AA        AGTGATCA                             TGGGTCGCGGAATAATAACCTAACAGCCAACTACCTGTTGAAGGAGTGCGGGTAGGGGGCGCGGCCGCGGGGTAAGTGTCAGTGGCAGCATAGAGG       CAAGTT A     GA
                                                TGTCAACATCAACACGGTGCTGATTCT CTCTGC       TCGTGGTGGTGATCGTGCTCTTGTTGTGGATTGCC CAT G  GGTGTTCA        AGTCGGCACTCCAGACAGTCTTCAGGTGC                                                                                                    GCAGTTC      G TGGCT  A
                                                ACAGTTGTAGTTGTGCCACGACTAAGA GAGACG       AGCACCACCACTAGCACGAGAGCAACACCTAACGG GTA C  CCACAAGT        TCAGCCGTGAGGTCTGTCAGAAGTCCACG                                                                                                    CGTCGAG      C ACTGG  T
GTCTCGGACAGGTTGGAGCTGGTCTTTCACAGGTCGCACGTCAGGGC                            C       GACCACG                                    A   G CG        --------                             CGTA----------------------------------------------------------------------------------------------       TAAGAT C     TT

----------CCGTGAGGTCTGTCAGAAGT----------     0.049   0.530   0.301
----------GCCGTGAGGTCTGTCAGAAGT----------     0.000   0.114   0.264
----------CGTGAGGTCTGTCAGAAGT----------     0.049   0.076   0.113
----------CAGCCGTGAGGTCTGTCAGAA----------     0.000   0.152   0.152
----------TCAGCCGTGAGGTCTGTCAGA----------     1.408   0.189   0.075
----------CAGGGCACAGTTGTAGTTGT----------     0.049   0.189   0.038
----------CGTCAGGGCACAGTTGTAGTTGT----------     0.000   0.114   0.075
----------CGTCAGGGCACAGTTGTAGTT----------     0.437   31.294   35.128 miR_t121
----------ACGTCAGGGCACAGTTGTAGT----------     0.000   0.038   0.038
----------CGTCAGGGCACAGTTGTAGT----------     0.000   0.720   0.414
----------CGTCAGGGCACAGTTGTAG----------     0.000   0.076   0.038
----------GTCGCACGTCAGGGCACAGTTGT----------     0.000   0.076   0.076
----------TCGCACGTCAGGGCACAGTTGT----------     0.000   0.038   0.038
----------GGTCGCACGTCAGGGCACAGTT----------     0.049   0.114   0.114
----CGGACAGGTTGGAGCTGGTCTT----------     0.000   0.038   0.038
```

```
miRNA: miR_t126 chromosome_5 Start: 1790619 End: 1790866 Condition: HS-N
------------------------------------------------------------------------------------------------------TATACAAGCGAAGTC-GAGACTT-----      0.000      0.152      0.152
------------------------------------------------------------------------------------------------------TATACAAGCGAAGTC-GAGACTTTGGT-      0.000      0.038      0.038
-----------------------------------------------------------------------------------------------AAGGATGTATACAAGCGAAG---------------      0.000      0.076      0.076
-------------------------------------------------------------------------------------------TTCCAAGGATGTATACAAGCGA-----------------      0.874      0.530      0.530
-------------------------------------------------------------------------------------------TTCCAAGGATGTATACAAGC-------------------      0.049      0.152      0.152
------------------------------------------------------------------------------------------TTTCCAAGGATGTATACAAGC-------------------      0.874      4.433      2.749
----------------------------------------------------------------------------------------TGTTTCCAAGGATGTATA------------------------      0.000      0.038      0.038
----------------------------------------------------------------------------------------TGTTTCCAAGGATGTATAC-----------------------      0.000      0.076      0.076
----------------------------------------------------------------------------------TGGCCGTGTTTCCAAGGATGTATA------------------------      0.097      0.114      0.038
----------------------------------------------------------------------------------TGGCCGTGTTTCCAAGGATGTA--------------------------      1.457      0.871      0.414
----------------------------------------------------------------------------------TGGCCGTGTTTCCAAGGATGTAT-------------------------      0.049      0.417      0.075
----------------------------------------------------------------------------------TGGCCGTGTTTCCAAGGATG----------------------------      3.351      1.856      1.318
-----------------------------------------------------------------GCACACTTGCGAGTCCGTGG---------------------------------------------      0.000      0.038      0.038
----------------------------------------------------------------TGCACACTTGCGAGTCCGTGG---------------------------------------------      5.051      1.061      0.715
---------------------------------------------------------------TTGCACACTTGCGAGTCCGTGGC--------------------------------------------      0.000      0.076      0.076
---------------------------------------------------------------TTGCACACTTGCGAGTCCGTGG---------------------------------------------      5.634      0.455      0.188
---------------------------------------------------------------TTGCACACTTGCGAGTCCGTG----------------------------------------------      0.389      0.152      0.075
--------------------------------------------------------TGGGGGCTTGCACACTTGCGA-----------------------------------------------------      0.097      0.038      0.075
-------------------------------------------------------TTGGGGGCTTGCACACTTGC-------------------------------------------------------      0.097      0.038      0.038
-------------------------------------------------------TTGGGGGCTTGCACACTTGCG------------------------------------------------------      5.974      0.189      0.715
------------------------------------------------------TTTGGGGGCTTGCACACTTGCG------------------------------------------------------      0.340      0.038      0.038
------------------------------------------------------TTTGGGGGCTTGCACACTTGC-------------------------------------------------------     10.491      4.092      6.777
-----------------------------------------------------TTTTGGGGGCTTGCACACTTGC-------------------------------------------------------      9.908     10.987     11.747
-----------------------------------------------------TTTTGGGGGCTTGCACACTTGCG------------------------------------------------------      0.097      0.189      0.189
-----------------------------------------------------TTTTGGGGGCTTGCACACTTG--------------------------------------------------------      0.194      0.114      0.301
----------------------------------ACGAATTCCCTCGCCACGTT----------------------------------------------------------------------------      0.000      0.114      0.114
---------------------------ACAGCCAACGAATTCCCTC------------------------------------------------------------------------------------      0.000      0.038      0.038
---------------------CAAGGGACAGCCAACGAATTC----------------------------------------------------------------------------------------      0.146      0.152      0.377
-------------------TGCAAGGGACAGCCAACGAAT------------------------------------------------------------------------------------------      0.486      0.227      0.151
-------------------TGCAAGGGACAGCCAACGA--------------------------------------------------------------------------------------------      0.000      0.114      0.038
-------------------TGCAAGGGACAGCCAACGAA-------------------------------------------------------------------------------------------      0.389      0.227      0.188
-----------------CATGCAAGGGACAGCCAACG---------------------------------------------------------------------------------------------      0.000      0.038      0.038
----------------TCATGCAAGGGACAGCCAACG---------------------------------------------------------------------------------------------      0.049      0.379      0.301
----------------TCATGCAAGGGACAGCCAACGA--------------------------------------------------------------------------------------------      0.243      0.227      0.527
----------------TCATGCAAGGGACAGCCAAC----------------------------------------------------------------------------------------------      0.049      0.038      0.038
----------TGTCGCTCATGCAAGGGACAGC--------------------------------------------------------------------------------------------------      0.049      0.038      0.038
--------TGTGTCGCTCATGCAAGGGACA----------------------------------------------------------------------------------------------------      0.000      0.076      0.076
------TCTGTGTCGCTCATGCAAGGGA------------------------------------------------------------------------------------------------------      0.000      0.038      0.038
----TATCTGTGTCGCTCATGCAAG---------------------------------------------------------------------------------------------------------     11.025      5.342      1.130

ACAGTATCT                           C         C  C                                                        C         C-   ACTT  GT
         GTGTCGCTCATGCAAGGGACAGCCAA GAATTCCCT GC ACGTTTTGGGGGCTTGCACACTTGCGAGTCCGTGGCCGTGTTTCCAAGGATGTATA AAGCGAAGT   GAG    TG  A
         CACAGCGAGTACGTTCCCTGTCGGTT CTTAAGGGA CG TGCAAAGCCCCCGAACGTGTGGACGCTCAGGCACCGGCACAGAGGTTCTTACATAT TTCGCTTCA   CTC    AC  A
--------A                           T         C  C                                                        T         CC   AT--  AA

-------------GCGAGTACGTTCCCTGTCGG-------------------------------------------------------------------------------------------------      0.146      0.038      0.075
--------------CGAGTACGTTCCCTGTCGG-------------------------------------------------------------------------------------------------      0.000      0.152      0.152
--------------CGAGTACGTTCCCTGTCGGT------------------------------------------------------------------------------------------------      1.700      5.418      3.125
------------AGCGAGTACGTTCCCTGTCGGT------------------------------------------------------------------------------------------------      0.000      0.038      0.038
-------------GCGAGTACGTTCCCTGTCGGT------------------------------------------------------------------------------------------------      5.974      2.425      1.619
-------------GCGAGTACGTTCCCTGTCGGTT-----------------------------------------------------------------------------------------------      0.000      0.076      0.076
--------------CGAGTACGTTCCCTGTCGGTT-----------------------------------------------------------------------------------------------      5.051     19.398      5.685
--------------CGAGTACGTTCCCTGTCGGTTT----------------------------------------------------------------------------------------------     38.466    125.630      7.530 miR_t126
---------------GAGTACGTTCCCTGTCGGTTT----------------------------------------------------------------------------------------------      0.583      0.985      0.678
--------------CGAGTACGTTCCCTGTCGGTTTC---------------------------------------------------------------------------------------------      0.049      0.038      0.038
--------------CGAGTACGTTCCCTGTCGGTTTCT--------------------------------------------------------------------------------------------      0.000      0.303      0.303
-----------------GTACGTTCCCTGTCGGTTTCT--------------------------------------------------------------------------------------------      7.625      1.212      3.163
-------------------ACGTTCCCTGTCGGTTTCTT-------------------------------------------------------------------------------------------      2.817      0.227      0.038
--------------------CGTTCCCTGTCGGTTTCTT-------------------------------------------------------------------------------------------      0.049      0.303      0.303
--------------CGAGTACGTTCCCTGTCGGTTTCTT-------------------------------------------------------------------------------------------      0.000      0.038      0.038
------------------TACGTTCCCTGTCGGTTTCTT-------------------------------------------------------------------------------------------      0.486      0.265      0.038
-----------------GTACGTTCCCTGTCGGTTTCTT-------------------------------------------------------------------------------------------      2.720      0.152      0.151
-------------------ACGTTCCCTGTCGGTTTCTTA------------------------------------------------------------------------------------------      0.000      0.076      0.076
--------------------CGTTCCCTGTCGGTTTCTTAA-----------------------------------------------------------------------------------------      2.477      1.402      0.264
---------------------GTTCCCTGTCGGTTTCTTAA-----------------------------------------------------------------------------------------      0.486      0.455      0.455
---------------------GTTCCCTGTCGGTTTCTTAAG----------------------------------------------------------------------------------------      1.360      0.341      0.113
----------------------------------TTCTTAAGGGACCGCTGCAAA---------------------------------------------------------------------------      0.000      0.152      0.152
------------------------------------CTTAAGGGACCGCTGCAAAGC-------------------------------------------------------------------------      0.194      0.303      0.075
--------------------------------------------ACCGCTGCAAAGCCCCCGAACGT---------------------------------------------------------------      0.000      0.038      0.038
------------------------------------------------------------CGAACGTGTGGACGCTCAGGC-------------------------------------------------      0.097      0.114      0.114
-------------------------------------------------------------GAACGTGTGGACGCTCAGGCA------------------------------------------------      1.894      1.250      1.431
------------------------------------------------------------CGAACGTGTGGACGCTCAGGCA------------------------------------------------      0.243      0.871      0.038
----------------------------------------------------------------------GACGCTCAGGCACCGGCACAGA--------------------------------------      0.049      0.568      0.678
---------------------------------------------------------------------------TCAGGCACCGGCACAGAGG------------------------------------      0.000      0.038      0.038
---------------------------------------------------------------------------TCAGGCACCGGCACAGAGGT-----------------------------------      0.097      0.076      0.113
-------------------------------------------------------------------------GCTCAGGCACCGGCACAGAGGT-----------------------------------      0.000      0.038      0.038
----------------------------------------------------------------------------CAGGCACCGGCACAGAGGTT----------------------------------      0.146      0.038      0.038
---------------------------------------------------------------------------TCAGGCACCGGCACAGAGGTT----------------------------------      2.137      1.061      1.061
------------------------------------------------------------------------------GGCACCGGCACAGAGGTT----------------------------------      0.194      0.038      0.038
--------------------------------------------------------------------------CTCAGGCACCGGCACAGAGGTT----------------------------------      0.000      0.038      0.038
-------------------------------------------------------------------------GCTCAGGCACCGGCACAGAGGTT----------------------------------      0.000      0.076      0.076
------------------------------------------------------------------------------GGCACCGGCACAGAGGTTCTT-------------------------------      2.768      1.856      0.640
-------------------------------------------------------------------------------GCACCGGCACAGAGGTTCTT-------------------------------      0.291      0.303      0.113
------------------------------------------------------------------------------------GGCACAGAGGTTCTTAC-----------------------------      0.000      0.038      0.038
----------------------------------------------------------------------------------CCGGCACAGAGGTTCTTAC-----------------------------      0.097      0.114      0.151
----------------------------------------------------------------------------------CCGGCACAGAGGTTCTTACA----------------------------      1.797      2.311      2.485
---------------------------------------------------------------------------------ACCGGCACAGAGGTTCTTACA----------------------------      0.000      0.038      0.038
-------------------------------------------------------------------------------GCACCGGCACAGAGGTTCTTACA----------------------------      0.000      0.152      0.188
------------------------------------------------------------------------------------GGCACAGAGGTTCTTACAT---------------------------      0.097      0.265      0.038
---------------------------------------------------------------------------------ACCGGCACAGAGGTTCTTACAT---------------------------      0.389      0.114      0.114
-------------------------------------------------------------------------------GCACCGGCACAGAGGTTCTTACAT---------------------------      0.097      1.515      0.489
-----------------------------------------------------------------------------------CGGCACAGAGGTTCTTACAT---------------------------      0.486      1.743      1.920
--------------------------------------------------------------------------------CACCGGCACAGAGGTTCTTACAT---------------------------      0.000      0.227      0.038
----------------------------------------------------------------------------------CCGGCACAGAGGTTCTTACAT---------------------------     42.934     81.910     55.836
-----------------------------------------------------------------------------------CGGCACAGAGGTTCTTACATA--------------------------      0.000      0.114      0.113
------------------------------------------------------------------------------------GGCACAGAGGTTCTTACATA--------------------------      0.680      0.152      0.602
------------------------------------------------------------------------------------GGCACAGAGGTTCTTACATAT-------------------------     47.888     28.566     57.267
-------------------------------------------------------------------------------------GCACAGAGGTTCTTACATAT-------------------------      0.389      0.455      0.339
-----------------------------------------------------------------------------------CGGCACAGAGGTTCTTACATAT-------------------------      1.117      7.577      6.438
----------------------------------------------------------------------------------CCGGCACAGAGGTTCTTACATAT-------------------------      0.146      2.766      0.038

miRNAs identified in TAP and HS+N :

miRNA: miR_t51,miR_t12 chromosome_5 Start: 935250 End: 935514 Condition: TAP
----------------------------------------------------------------------------------------------------------TCCGCTTCAGGTACACGCCGC--------------      0.049      0.000      0.000
---------------------------------------------------------------------------------------------------CAAGATGTCCGCTTCAGG------------------------      0.049      0.493      1.242
---------------------------------------------------------------------------------------------------CAAGATGTCCGCTTCAGGTAC---------------------      0.680      4.584      4.029
------------------------------------------------------------------------------------------------TGGCAAGATGTCCGCTTCAGGT-----------------------      0.049      0.000      0.000
------------------------------------------------------------------------------------------------TGGCAAGATGTCCGCTTCAGG------------------------      0.243      1.099      0.979
-----------------------------------------------------------------------------------------------TTGGCAAGATGTCCGCTTCAG-------------------------      0.340      0.076      0.075
-----------------------------------------------------------------------------------------------TTGGCAAGATGTCCGCTTCAGGT-----------------------      0.049      0.000      0.000
-----------------------------------------------------------------------------------------------TTGGCAAGATGTCCGCTTCAGG------------------------      7.625     18.829     29.744
----------------------------------------------------------------------------------------------TTTGGCAAGATGTCCGCTTCAGG------------------------      1.943      1.781      2.485
----------------------------------------------------------------------------------------------TTTGGCAAGATGTCCGCTTCAG-------------------------      0.194      0.075      0.075
-------------------------------------------------------------------------------------AGCATTTCGTTTGG------------------------------------------      0.146      0.000      0.000
------------------------------------------------------------------------------------CAGCATTTCGTTTGG------------------------------------------      0.146      0.000      0.000
------------------------------------------------------------------------------------CAGCATTTCGTTTGGCAAG--------------------------------------      0.049      0.000      0.000
-----------------------------------------------------------------------------------TCAGCATTTCGTTTGGCAAGA-------------------------------------      0.097      0.000      0.000
-----------------------------------------------------------------------------------TCAGCATTTCGTTTGG------------------------------------------      0.097      0.000      0.000
-----------------------------------------------------------------------------------TCAGCATTTCGTTTGGCAAG--------------------------------------      0.874      0.379      0.264
----------------------------------------------------------------------------------GTCAGCATTTCGTTTGG------------------------------------------      0.146      0.038      0.038
---------------------------------------------------------------------------------GGTCAGCATTTCGTTTGG------------------------------------------      6.411      1.250      1.205
---------------------------------------------------------------------------------GGTCAGCATTTCGTTTG-------------------------------------------      0.097      0.038      0.075
---------------------------------------------------------------------------------GGTCAGCATTTCGTTTGGC-----------------------------------------      0.146      0.000      0.000
--------------------------------------------------------------------------------CGGTCAGCATTTCGTTTGG------------------------------------------     18.650      3.107      3.125
--------------------------------------------------------------------------------CGGTCAGCATTTCGTTTGGC-----------------------------------------      0.437      0.265      0.264
-------------------------------------------------------------------------------TCGGTCAGCATTTCGTTTGGCA----------------------------------------      0.146      0.000      0.000
-------------------------------------------------------------------------------TCGGTCAGCATTTCGTTTGG------------------------------------------   4444.765    622.165    427.149 miR_t51
-------------------------------------------------------------------------------TCGGTCAGCATTTCGTTTG-------------------------------------------     47.548      5.304      7.041
-------------------------------------------------------------------------------TCGGTCAGCATTTCGTTT--------------------------------------------      0.194      0.075      0.075
-------------------------------------------------------------------------------TCGGTCAGCATTTCGTTTGGC-----------------------------------------    231.719     21.936     24.435
------------------------------------------------------------------------------CTCGGTCAGCATTTCGTTTG-------------------------------------------      0.194      0.038      0.038
------------------------------------------------------------------------------CTCGGTCAGCATTTCGTTTGG------------------------------------------      3.060      0.871      0.565
-----------------------------------------------------------------------------TCTCGGTCAGCATTTCGTTTG-------------------------------------------    106.704      3.220      4.631
-----------------------------------------------------------------------------TCTCGGTCAGCATTTCGTTTGGC-----------------------------------------      0.389      0.455      0.113
-----------------------------------------------------------------------------TCTCGGTCAGCATTTCGTT---------------------------------------------      0.243      0.000      0.000
-----------------------------------------------------------------------------TCTCGGTCAGCATTTCGT----------------------------------------------      0.097      0.000      0.000
-----------------------------------------------------------------------------TCTCGGTCAGCATTTCGTTTGG------------------------------------------    344.445     55.048     30.309
-----------------------------------------------------------------------------TCTCGGTCAGCATTTCGTTT--------------------------------------------      0.049      0.038      0.038
---------------------------------------------------------------------------TATCTCGGTCAGCATTTCG-----------------------------------------------      2.477      0.000      0.000
---------------------------------------------------------------------------TATCTCGGTCAGCATTTCGTTTG-------------------------------------------      0.389      0.000      0.000
---------------------------------------------------------------------------TATCTCGGTCAGCATTTCGT----------------------------------------------     11.025      0.000      0.000
---------------------------------------------------------------------------TATCTCGGTCAGCATTTCGTTT--------------------------------------------      0.243      0.000      0.000
---------------------------------------------------------------------------TATCTCGGTCAGCATTTCGTTTGG------------------------------------------      0.291      0.000      0.000
---------------------------------------------------------------------------TATCTCGGTCAGCATTTCGTT---------------------------------------------     14.813      0.000      0.000
----------------------------------------------------------AGCAAGCTACTTCAGGGTATC--------------------------------------------------------------      0.049      0.000      0.000
---------------------------------------------------------TAGCAAGCTACTTCAGGGTAT---------------------------------------------------------------      4.031      1.137      1.280
---------------------------------------------------------TAGCAAGCTACTTCAGGGTA----------------------------------------------------------------      0.097      0.000      0.000
---------------------------------------------------------TAGCAAGCTACTTCAGGGTATC--------------------------------------------------------------      0.340      0.038      0.075
--------------------------------------------------------TTAGCAAGCTACTTCAGG-------------------------------------------------------------------      0.146      0.038      0.075
--------------------------------------------------------TTAGCAAGCTACTTCAGGGTATC--------------------------------------------------------------      1.408      1.667      0.264
--------------------------------------------------------TTAGCAAGCTACTTCAGGGT-----------------------------------------------------------------      0.146      0.227      0.038
--------------------------------------------------------TTAGCAAGCTACTTCAGGGTAT---------------------------------------------------------------     16.853     10.722      3.351
--------------------------------------------------------TTAGCAAGCTACTTCAGGGTA----------------------------------------------------------------     10.782     11.783      3.878
------------------------------------------------------TATTAGCAAGCTACTTCAGGGTAT---------------------------------------------------------------      0.146      0.379      0.379
----------------------------------------------TCG-CCACTATTAGCAAGCTACTTC----------------------------------------------------------------------      0.049      0.000      0.000
---------------------------------------GAGGACATCG-CCACT--------------------------------------------------------------------------------------      0.049      0.000      0.000
-------------------------------------AGGAGGACATCG-CCACT--------------------------------------------------------------------------------------      0.049      0.000      0.000
-------------------------------------AGGAGGACATCG-CCACTAT------------------------------------------------------------------------------------      0.049      0.000      0.000
-----------------------------------TCAGGAGGACATCG-CCACTATT-----------------------------------------------------------------------------------      0.049      0.076      0.038
-----------------------------------TCAGGAGGACATCG-CCACTA-------------------------------------------------------------------------------------      0.049      1.061      0.489
-----------------------------------TCAGGAGGACATCG-CCACT--------------------------------------------------------------------------------------      0.243      3.523      1.167
-----------------------------------TCAGGAGGACATCG-CCACTAT------------------------------------------------------------------------------------      0.146      0.076      0.038
----------------------------------CTCAGGAGGACATCG-CCAC---------------------------------------------------------------------------------------      0.097      0.076      0.075
```

```
---------------------------------CTCAGGAGGACATCG-CCACTA-------------------------------------------------------------------------------------   0.291   0.379   0.377
---------------------------------CTCAGGAGGACATCG-CCACTAT------------------------------------------------------------------------------------   0.097   0.189   0.113
---------------------------------CTCAGGAGGACATCG-CCACT--------------------------------------------------------------------------------------   0.971   0.493   0.602
--------------------------------TCTCAGGAGGACATCG-CCACT--------------------------------------------------------------------------------------   90.822   232.848   174.700
--------------------------------TCTCAGGAGGACATCG-CCACTAT------------------------------------------------------------------------------------   14.668   16.973   11.295
--------------------------------TCTCAGGAGGACATCG-CCACTATTAG---------------------------------------------------------------------------------   0.194   0.152   0.152
--------------------------------TCTCAGGAGGACATCG-CCACTA-------------------------------------------------------------------------------------   42.837   105.740   94.466
--------------------------------TCTCAGGAGGACATCG-CC-----------------------------------------------------------------------------------------   0.097   0.493   0.602
--------------------------------TCTCAGGAGGACATCG-CCA----------------------------------------------------------------------------------------   0.194   0.038   0.038
--------------------------------TCTCAGGAGGACATCG-CCACTATT-----------------------------------------------------------------------------------   1.457   2.160   0.339
--------------------------------TCTCAGGAGGACATCG-CCAC---------------------------------------------------------------------------------------   6.022   12.616   11.747
--------------------------------TCTCAGGAGGACATCG-C------------------------------------------------------------------------------------------   0.049   0.152   0.152
------------------------------TGTCTCAGGAGGACATCG-CCAC---------------------------------------------------------------------------------------   0.049   0.000   0.000
------------------------------TGTCTCAGGAGGACATCG-CC-----------------------------------------------------------------------------------------   0.049   0.000   0.000
---------------------CGCCGCACATGTCTCAGGAGG--------------------------------------------------------------------------------------------------   0.049   0.000   0.000
-------------------TGCGCCGCACATGTCTCAGG-----------------------------------------------------------------------------------------------------   0.389   0.341   0.264
-----------------TCTGCGCCGCACATGTCTCA-------------------------------------------------------------------------------------------------------   0.146   0.000   0.000
-----------------TCTGCGCCGCACATGTCTCAGG-----------------------------------------------------------------------------------------------------   0.097   0.000   0.000
-----------------TCTGCGCCGCACATGTCTCAG------------------------------------------------------------------------------------------------------   0.146   0.076   0.075
-----------------TCTGCGCCGCACATGTCTC--------------------------------------------------------------------------------------------------------   0.097   0.000   0.000

-------------                              C    -  AC  T         A  TC                       T                   A     A         C       AG
             GACGTCTGCGCCGCACATGTCTCAGGAGGA ATCG CC  TA TAGCAAGCT CT  AGGGTATCTCGGTCAGCATTTCG TTGGCAAGATGTCCGCTTC GGTAC CGCCGCTCA GCGATGA  C
             CTGCAGACGTGGCGTGTACAGAGTCCTTCT TAGC GG  AT ATCGTTCGA GA  TCCCATAGAGCCAGTCGTAGAGC AACCGTTCTGCAGGTGAGG CCATG GCGGCGAGT CGCTACT  /
GCCCGGTTATGCT                              C    A  C-  C         C  CC                       T                   C     C         A       GG

-------TATGCTCTGCAGACGTGGCGT----------------------------------------------------------------------------------------------------------------   0.680   0.038   0.038
------TTATGCTCTGCAGACGTGGCGT----------------------------------------------------------------------------------------------------------------   1.408   0.114   0.114
---------TGCTCTGCAGACGTGGCGT----------------------------------------------------------------------------------------------------------------   0.291   0.341   0.075
-----GTTATGCTCTGCAGACGTGGCGT----------------------------------------------------------------------------------------------------------------   0.049   0.000   0.000
--------ATGCTCTGCAGACGTGGCGT----------------------------------------------------------------------------------------------------------------   1.943   0.227   0.264
--------ATGCTCTGCAGACGTGGCGTG---------------------------------------------------------------------------------------------------------------   0.097   0.038   0.038
----------GCTCTGCAGACGTGGCGTG---------------------------------------------------------------------------------------------------------------   0.049   0.000   0.000
------TTATGCTCTGCAGACGTGGCGTGT--------------------------------------------------------------------------------------------------------------   1.603   0.227   0.227
--------ATGCTCTGCAGACGTGGCGTGT--------------------------------------------------------------------------------------------------------------   51.142   6.819   9.036
-----------CTCTGCAGACGTGGCGTGT--------------------------------------------------------------------------------------------------------------   0.146   0.076   0.038
-------TATGCTCTGCAGACGTGGCGTGT--------------------------------------------------------------------------------------------------------------   0.583   0.038   0.038
----------GCTCTGCAGACGTGGCGTGT--------------------------------------------------------------------------------------------------------------   1.263   0.758   0.414
--------------TGCAGACGTGGCGTGT--------------------------------------------------------------------------------------------------------------   0.049   0.000   0.000
-----GTTATGCTCTGCAGACGTGGCGTGT--------------------------------------------------------------------------------------------------------------   0.049   0.000   0.000
---------TGCTCTGCAGACGTGGCGTGT--------------------------------------------------------------------------------------------------------------   3.837   2.273   1.318
----GGTTATGCTCTGCAGACGTGGCGTGT--------------------------------------------------------------------------------------------------------------   0.049   0.000   0.000
------------TCTGCAGACGTGGCGTGT--------------------------------------------------------------------------------------------------------------   0.049   0.000   0.000
-----------------AGACGTGGCGTGTACAGAGT-------------------------------------------------------------------------------------------------------   1.020   0.758   0.904
---------------GCAGACGTGGCGTGTACAGAGT-------------------------------------------------------------------------------------------------------   0.826   1.856   0.640
--------------------CGTGGCGTGTACAGAGTCCT----------------------------------------------------------------------------------------------------   0.049   0.000   0.000
----------------------TGGCGTGTACAGAGTCCTTCT-------------------------------------------------------------------------------------------------   2.671   0.758   1.054
--------------------CGTGGCGTGTACAGAGTCCTTCT-------------------------------------------------------------------------------------------------   0.971   0.341   0.038
---------------------GTGGCGTGTACAGAGTCCTTCT-------------------------------------------------------------------------------------------------   0.826   1.099   0.791
-----------------------GGCGTGTACAGAGTCCTTCTCT-----------------------------------------------------------------------------------------------   0.389   0.303   0.339
----------------------------GTACAGAGTCCTTCTCTAGCAGGC----------------------------------------------------------------------------------------   0.146   0.000   0.000
---------------------------------GAGTCCTTCTCTAGCAGGC----------------------------------------------------------------------------------------   0.049   0.038   0.038
------------------------------ACAGAGTCCTTCTCTAGCAGGC----------------------------------------------------------------------------------------   0.971   0.871   0.871
-------------------------------CAGAGTCCTTCTCTAGCAGGC-A--------------------------------------------------------------------------------------   0.194   1.440   0.038
---------------------------------GAGTCCTTCTCTAGCAGGC-A--------------------------------------------------------------------------------------   0.291   1.174   2.523
------------------------------ACAGAGTCCTTCTCTAGCAGGC-A--------------------------------------------------------------------------------------   0.049   1.402   1.402
-----------------------------------GTCCTTCTCTAGCAGGC-AT-------------------------------------------------------------------------------------   0.049   0.076   0.038
--------------------------------------CTTCTCTAGCAGGC-AT-------------------------------------------------------------------------------------   0.049   0.000   0.000
---------------------------------GAGTCCTTCTCTAGCAGGC-AT-------------------------------------------------------------------------------------   29.675   238.000   185.807
-------------------------------------CCTTCTCTAGCAGGC-AT-------------------------------------------------------------------------------------   0.146   0.000   0.000
------------------------------------TCCTTCTCTAGCAGGC-AT-------------------------------------------------------------------------------------   0.049   0.000   0.000
----------------------------------AGTCCTTCTCTAGCAGGC-AT-------------------------------------------------------------------------------------   1.068   0.341   0.113
------------------------------ACAGAGTCCTTCTCTAGCAGGC-AT-------------------------------------------------------------------------------------   5.585   192.196   1.694
--------------------------------AGAGTCCTTCTCTAGCAGGC-AT-------------------------------------------------------------------------------------   7.140   21.595   0.226
-------------------------------CAGAGTCCTTCTCTAGCAGGC-AT-------------------------------------------------------------------------------------   6.945   188.067   1.845
-----------------------------TACAGAGTCCTTCTCTAGCAGGC-AT-------------------------------------------------------------------------------------   0.146   1.288   0.038
-------------------------------------CCTTCTCTAGCAGGC-ATCATCGT-------------------------------------------------------------------------------   0.243   2.728   0.377
---------------------------------------TTCTCTAGCAGGC-ATCATCGT-------------------------------------------------------------------------------   0.049   0.682   0.038
-----------------------------------------CTCTAGCAGGC-ATCATCGTT------------------------------------------------------------------------------   0.097   0.114   0.038
----------------------------------------TCTCTAGCAGGC-ATCATCGTT------------------------------------------------------------------------------   1.068   1.553   1.017
-------------------------------------CCTTCTCTAGCAGGC-ATCATCGTT------------------------------------------------------------------------------   0.049   0.114   0.114
-------------------------------------------------------CATCGTTCGACGACCTC--------------------------------------------------------------------   0.049   0.000   0.000
-------------------------------------------------------CATCGTTCGACGACCTCCCA-----------------------------------------------------------------   0.097   0.075   0.075
------------------------------------------------------TCATCGTTCGACGACCTCCCA-----------------------------------------------------------------   0.049   0.152   0.038
------------------------------------------------------TCATCGTTCGACGACCTCCCAT----------------------------------------------------------------   0.340   0.152   0.188
-------------------------------------------------------CATCGTTCGACGACCTCCCAT----------------------------------------------------------------   30.695   9.585   7.493
---------------------------------------------------C-ATCATCGTTCGACGACCTCCCAT----------------------------------------------------------------   0.049   0.000   0.000
---------------------------------------------------------TCGTTCGACGACCTCCCAT----------------------------------------------------------------   0.049   0.000   0.000
-------------------------------------------------GGC-ATCATCGTTCGACGACCTCCCAT----------------------------------------------------------------   0.049   0.000   0.000
-----------------------------------------------------ATCATCGTTCGACGACCTCCCAT----------------------------------------------------------------   0.049   0.000   0.000
-----------------------------------------------------------------CGACCTCCCATAGAGCCAGT-------------------------------------------------------   0.049   0.038   0.038
--------------------------------------------------------------CGACGACCTCCCATAGAGCCAGT-------------------------------------------------------   0.097   0.038   0.038
---------------------------------------------------------------GACGACCTCCCATAGAGCCAGT-------------------------------------------------------   0.049   0.000   0.000
----------------------------------------------------------------ACGACCTCCCATAGAGCCAGT-------------------------------------------------------   0.146   0.000   0.000
-------------------------------------------------------------------ACCTCCCATAGAGCCAGTCGT----------------------------------------------------   0.146   0.265   0.113
------------------------------------------------------------------GACCTCCCATAGAGCCAGTCGT----------------------------------------------------   0.049   0.038   0.038
----------------------------------------------------------------------TCCCATAGAGCCAGTCGTAGA-------------------------------------------------   0.389   0.417   0.339
-------------------------------------------------------------------------CATAGAGCCAGTCGTAGAGCT----------------------------------------------   0.146   0.303   0.565
-----------------------------------------------------------------------------GAGCCAGTCGTAGAGCTAA--------------------------------------------   0.049   0.000   0.000
----------------------------------------------------------------------------AGAGCCAGTCGTAGAGCTAA--------------------------------------------   0.146   0.493   0.377 miR_t51*
---------------------------------------------------------------------------TAGAGCCAGTCGTAGAGCTAA--------------------------------------------   0.194   0.417   0.151
------------------------------------------------------------------------------AGCCAGTCGTAGAGCTAACCG-----------------------------------------   0.097   0.113   0.113
-------------------------------------------------------------------------------GCCAGTCGTAGAGCTAACCG-----------------------------------------   0.097   0.303   0.339
------------------------------------------------------------------------------AGCCAGTCGTAGAGCTAACCGT----------------------------------------   0.437   0.189   0.188
-------------------------------------------------------------------------------GCCAGTCGTAGAGCTAACCGT----------------------------------------   4.857   7.805   9.977
----------------------------------------------------------------------------------AGTCGTAGAGCTAACCGTTCT-------------------------------------   0.194   0.076   0.076
----------------------------------------------------------------------------------------AGAGCTAACCGTTCTGCAGGT-------------------------------   0.049   0.000   0.000
---------------------------------------------------------------------------------------------TAACCGTTCTGCAGGTGAGGC--------------------------   0.049   0.000   0.000
--------------------------------------------------------------------------------------------CTAACCGTTCTGCAGGTGAGGC--------------------------   0.097   0.038   0.038
-------------------------------------------------------------------------------------------GCTAACCGTTCTGCAGGTGAGGC--------------------------   0.097   0.000   0.000
-------------------------------------------------------------------------------------------------CGTTCTGCAGGTGAGGCC-------------------------   0.049   0.000   0.000
------------------------------------------------------------------------------------------------CCGTTCTGCAGGTGAGGCC-------------------------   0.049   0.000   0.000
-------------------------------------------------------------------------------------------------CGTTCTGCAGGTGAGGCCC------------------------   1.214   0.568   1.958
------------------------------------------------------------------------------------------------CCGTTCTGCAGGTGAGGCCC------------------------   0.923   0.189   0.301
-------------------------------------------------------------------------------------------------CGTTCTGCAGGTGAGGCCCA-----------------------   5.828   5.910   26.694
------------------------------------------------------------------------------------------------CCGTTCTGCAGGTGAGGCCCA-----------------------   5.440   25.687   25.979
--------------------------------------------------------------------------------------------------GTTCTGCAGGTGAGGCCCAT----------------------   3.788   6.857   5.723
----------------------------------------------------------------------------------------------------TCTGCAGGTGAGGCCCAT----------------------   0.049   0.038   0.038
-----------------------------------------------------------------------------------------------ACCGTTCTGCAGGTGAGGCCCAT----------------------   2.137   9.434   0.188
-------------------------------------------------------------------------------------------------------GCAGGTGAGGCCCAT----------------------   0.049   0.000   0.000
------------------------------------------------------------------------------------------------CCGTTCTGCAGGTGAGGCCCAT----------------------   608.802   813.717   501.810
-------------------------------------------------------------------------------------------------CGTTCTGCAGGTGAGGCCCAT----------------------   752.467   1662.972   2815.566 miR_t12
-----------------------------------------------------------------------------------------------------CTGCAGGTGAGGCCCAT----------------------   0.049   0.038   0.075
---------------------------------------------------------------------------------------------------TTCTGCAGGTGAGGCCCAT----------------------   0.194   0.114   0.301
```

```
miRNA: miR_t66,miR_t65 chromosome_11 Start: 1442246 End: 1442394 Condition: TAP
----------------CTGTTTACCAACGTGCAGGGGG--------------------------------------------   0.049   0.000   0.000
----------------CTGTTTACCAACGTGCAGGGG---------------------------------------------   0.097   0.000   0.000
-----------------TGTTTACCAACGTGCAGGGGG--------------------------------------------   5.003   0.455   1.167
-----------------TGTTTACCAACGTGCAGGG----------------------------------------------   0.049   0.038   0.038
-----------------TGTTTACCAACGTGCAGGGG---------------------------------------------   2.428   0.379   0.377
-------------------TTTACCAACGTGCAGGGGG--------------------------------------------   0.097   0.038   0.038
-------------------TTTACCAACGTGCAGGGGGACT-----------------------------------------   0.194   1.364   0.377
-------------------TTTACCAACGTGCAGG-----------------------------------------------   0.049   0.000   0.000
-------------------TTTACCAACGTGCAGGGGGAC------------------------------------------   1.214   0.720   1.280
-------------------TTTACCAACGTGCAGGGGGA-------------------------------------------   0.534   0.114   0.188
--------------------TTACCAACGTGCAGGGGGACTTAG--------------------------------------   0.049   0.000   0.000
--------------------TTACCAACGTGCAGGGGGA-------------------------------------------   0.097   0.000   0.000
--------------------TTACCAACGTGCAGGGGGACT-----------------------------------------   0.291   0.227   0.226
---------------------TACCAACGTGCAGGGGGACT-----------------------------------------   0.146   0.038   0.151
-----------------------CCAACGTGCAGGGGGACTTAG--------------------------------------   0.049   0.227   0.227
-------------------------AACGTGCAGGGGGACTTAGTGG-----------------------------------   0.291   3.978   3.978
-----------------------------TGCAGGGGGACTTAGTGGAG---------------------------------   78.826   57.511   81.627
-----------------------------TGCAGGGGGACTTAGTGGAGATCC-----------------------------   0.049   0.000   0.000
-----------------------------TGCAGGGGGACTTAGTGGAGA--------------------------------   227.833   102.974   106.589 miR_t66
-----------------------------TGCAGGGGGACTTAGTGG-----------------------------------   0.874   4.471   1.920
-----------------------------TGCAGGGGGACTTAG--------------------------------------   0.049   0.000   0.000
-----------------------------TGCAGGGGGACTTAGTGGA----------------------------------   8.014   16.897   8.660
-----------------------------TGCAGGGGGACTTAGTGGAGAT-------------------------------   1.020   0.114   0.678
-----------------------------TGCAGGGGGACTTAGTGGAGATC------------------------------   0.097   0.038   0.038
------------------------------GCAGGGGGACTTAGTGGAG---------------------------------   0.583   0.114   0.565
------------------------------GCAGGGGGACTTAGTGGAGAT-------------------------------   0.049   0.000   0.000
------------------------------GCAGGGGGACTTAGTGGAGA--------------------------------   1.408   0.417   1.318
-------------------------------CAGGGGGACTTAGTGGAGATC------------------------------   0.826   2.046   4.706
-------------------------------CAGGGGGACTTAGTGGAGA--------------------------------   0.049   0.114   0.114
--------------------------------AGGGGGACTTAGTGGAGA--------------------------------   0.049   0.000   0.000
--------------------------------AGGGGGACTTAGTGGAG---------------------------------   0.049   0.000   0.000
----------------------------------GGGGACTTAGTGGAGA--------------------------------   0.097   0.000   0.000
-----------------------------------------TAGTGGAGATCCTCCTGTCCGG-------------------   0.097   0.455   0.339
-----------------------------------------TAGTGGAGATCCTCCTGTCCG--------------------   0.923   0.985   0.188
-----------------------------------------TAGTGGAGATCCTCCTGTCCGGC------------------   0.049   0.038   0.226
--------------------------------------------TGGAGATCCTCCTGTCCGGC------------------   2.234   22.239   10.994
--------------------------------------------TGGAGATCCTCCTGTCCGGCT---ACCTGG--------   0.049   0.076   0.076
--------------------------------------------TGGAGATCCTCCTGTCCGGCT-----------------   1.408   1.212   0.753
--------------------------------------------TGGAGATCCTCCTGTCCGG-------------------   0.049   0.114   0.038
--------------------------------------------------TCCTCCTGTCCGGCT---ACCTGG--------   0.049   0.000   0.000
--------------------------------------------------------TGTCCGGCT---ACCTGGAGCGCAAG   0.000   0.038   0.038

------------TTA                                                  ---    GGA  G AA
               GCTGTTTACCAACGTGCAGGGGGACTTAGTGGAGATCCTCCTGTCCGGCT   ACCT   GC C  C
               TGACAAATGGTTGCACGTCCCCCTGAATCACCTCTAGGAGGACAGGCCGA   TGGA   CG G  /
CGAACGCGAGGTCCC                                                  TTT    GAG  G TT

------------------------------CGTCCCCCTGAATCACCTCT--------------------------------   0.534   0.038   0.226
-----------------------------ACGTCCCCCTGAATCACCTCT--------------------------------   5.585   0.909   1.393
----------------------------CACGTCCCCCTGAATCACCT----------------------------------   0.049   0.038   0.038
```

```
---------------------------GCACGTCCCCCTGAATCACCT----------------------------------      2.768     1.819     2.334 miR_t66*
--------------------------TGCACGTCCCCCTGAATCACCT----------------------------------      0.049     0.000     0.000
-------------------------------GTCCCCCTGAATCACCT----------------------------------      0.049     0.000     0.000
---------------------------GCACGTCCCCCTGAATCAC------------------------------------      0.049     0.038     0.075
----------------------TGGTTGCACGTCCCCCTGAAT---------------------------------------      0.389     0.000     0.000
-----------------------GGTTGCACGTCCCCCTGAAT---------------------------------------      0.777     0.606     0.188
----------------GACAAATGGTTGCACGTCCCCCT-------------------------------------------      0.291     1.023     1.023
---------------TGACAAATGGTTGCACGTCCCCCT-------------------------------------------      0.049     0.152     0.152
------------CCCTGACAAATGGTTGCACGTC------------------------------------------------      0.389     0.227     0.226
---------------TGACAAATGGTTGCACGT-------------------------------------------------      0.243     0.265     0.265
-------------CCTGACAAATGGTTGCACGT-------------------------------------------------      7.334    40.803    20.331
-----------TCCCTGACAAATGGTTGCACGT-------------------------------------------------      0.971     1.515     0.941
----------------GACAAATGGTTGCACGT-------------------------------------------------      0.049     0.000     0.000
--------------CTGACAAATGGTTGCACGT-------------------------------------------------      0.437     0.152     0.339
------CGAGGTCCCTGACAAATGGTTGCACGT-------------------------------------------------      0.049     0.000     0.000
----------GTCCCTGACAAATGGTTGCACGT-------------------------------------------------      2.720     5.910     1.242
------------CCCTGACAAATGGTTGCACGT-------------------------------------------------    993.948   593.713   670.674 miR_t65
-------GAGGTCCCTGACAAATGGTTGCACGT-------------------------------------------------      0.146     0.265     0.265
-----------------ACAAATGGTTGCACGT-------------------------------------------------      0.097     0.000     0.000
---------GGTCCCTGACAAATGGTTGCACGT-------------------------------------------------      2.526     5.001     0.452
----------GTCCCTGACAAATGGTTGCACG--------------------------------------------------      0.049     0.000     0.000
------------CCCTGACAAATGGTTGCACG--------------------------------------------------      3.303     0.833     4.518
------------CCCTGACAAATGGTTGCAC---------------------------------------------------      0.049     0.000     0.000
------------CCCTGACAAATGGTTGCA----------------------------------------------------      0.194     0.076     0.301
-------GAGGTCCCTGACAAATGGTTGC-----------------------------------------------------      0.389     0.189     0.189
----CGCGAGGTCCCTGACAAATGGTT-------------------------------------------------------      0.049     0.227     0.075
------CGAGGTCCCTGACAAATGGTT-------------------------------------------------------      4.225     7.767     1.920
-------GAGGTCCCTGACAAATGGTT-------------------------------------------------------      1.554     1.099     0.941
-----GCGAGGTCCCTGACAAATGGTT-------------------------------------------------------      0.437     0.417     0.038
------CGAGGTCCCTGACAAATGGT--------------------------------------------------------      0.049     0.189     0.038
-----GCGAGGTCCCTGACAAAT-----------------------------------------------------------      0.049     0.000     0.000

miRNA: miR_t88,miR_t53 chromosome_2 Start: 9173274 End: 9173422 Condition: TAP
----TAGACGGCTGGAGGGCGCAAG--------------------------------------------------      0.243     0.303     0.565
------------TGGAGGGCGCAAGCACGCA--------------------------------------------      0.049     0.188     0.188
------------TGGAGGGCGCAAGCACGCAACG-----------------------------------------     47.305    12.957    23.871 miR_t88
------------TGGAGGGCGCAAGCACGCAACGA----------------------------------------      0.049     0.000     0.000
------------TGGAGGGCGCAAGCACGCAAC------------------------------------------     29.772     3.372    13.856
------------TGGAGGGCGCAAGCACGCAA-------------------------------------------      0.097     0.038     0.038
-------------GGAGGGCGCAAGCACGCAAC------------------------------------------      0.291     0.038     0.339
-------------GGAGGGCGCAAGCACGCAACG-----------------------------------------      0.291     0.038     0.452

C                                                                   G G- G
 GGCTAGACGGCTGGAGGGCGCAAGCACGCAACGAGGGGATGACGAGCACGATGTGGGCCTGATACAG C  G C
 TCGATCTGCCGACCTCCCGCGTTCGTGCGTTGCTCCCCTACTGCTCGTGCTACGCCCGGACTATGTC G  C A
-                                                                   G AG A

--------------------------------GCTCCCCTACTGCTCGTGCT-----------------------    413.461   144.725   116.303 miR_t53
-------------------------------TGCTCCCCTACTGCTCGTGCT-----------------------     13.551     1.326     1.167
--------------------------------GCTCCCCTACTGCTCGTGC------------------------      2.768     0.682     1.431
--------------------------------GCTCCCCTACTGCTCGTG-------------------------      0.971     0.114     0.414
-------------------------------TGCTCCCCTACTGCTCGTG-------------------------      0.049     0.000     0.000
------------------------------TTGCTCCCCTACTGCTCGT--------------------------      0.049     0.000     0.000
----------------------------CGTTGCTCCCCTACTGCTCGT--------------------------      4.128     1.212     1.619
-----------------------------GTTGCTCCCCTACTGCTCGT--------------------------      2.186     0.909     1.280
---------------------------GCGTTGCTCCCCTACTGCTCGT--------------------------      5.585     4.774     1.770
---------------------------GCGTTGCTCCCCTACTGCTCG---------------------------      0.146     0.227     0.075
-----------------------------GTTGCTCCCCTACTGCTCG---------------------------      0.146     0.114     0.113
----------------------------CGTTGCTCCCCTACTGCTCG---------------------------      0.146     0.038     0.113
------------------------CGTGCGTTGCTCCCCTACTGCT-----------------------------      0.049     0.000     0.000
-------------------------GTGCGTTGCTCCCCTACTGCT-----------------------------      0.291     0.075     0.075
-----------------------TCGTGCGTTGCTCCCCTACTGCT-----------------------------      0.049     0.000     0.000
------------------------CGTGCGTTGCTCCCCTACT--------------------------------      0.049     0.038     0.075
---------------------GTTCGTGCGTTGCTCCCCTACT--------------------------------      0.097     0.000     0.000
-------------------GCGTTCGTGCGTTGCTCCCCT-----------------------------------      0.243     0.076     0.151
--------------------CGTTCGTGCGTTGCTCCCCT-----------------------------------      0.146     0.265     0.264
------------ACCTCCCGCGTTCGTGCGTT-------------------------------------------      0.583     0.038     0.038
----------CGACCTCCCGCGTTCGTGCGTT-------------------------------------------     19.670    15.117    10.354 miR_t88*
-----------GACCTCCCGCGTTCGTGCGTT-------------------------------------------      0.389     0.152     0.113
-------------CCTCCCGCGTTCGTGCGTT-------------------------------------------      0.194     0.152     0.075
----------CGACCTCCCGCGTTCGTGCGT--------------------------------------------      2.817     0.871     1.355
-----TCTGCCGACCTCCCGCGTTCGT------------------------------------------------      0.097     0.038     0.038
------CTGCCGACCTCCCGCGTTCGT------------------------------------------------      0.971     0.152     0.678
-------TGCCGACCTCCCGCGTTCGT------------------------------------------------      0.049     0.038     0.038

miRNA: miR_t115 chromosome_12 Start: 2444595 End: 2444930 Condition: TAP
----AAGCTCTTCGAGGTCGGCTGG-----------------------------------------------------------------------------------------------------------------------------------------------------      0.097     2.614     2.410
--------TCTTCGAGGTCGGCTGGAA---------------------------------------------------------------------------------------------------------------------------------------------------      0.049     0.188     0.188
--------TCTTCGAGGTCGGCTGGAAG--------------------------------------------------------------------------------------------------------------------------------------------------      0.097     1.629     0.602
--------TCTTCGAGGTCGGCTGGAAGG-------------------------------------------------------------------------------------------------------------------------------------------------      7.140   162.683    74.963
---------CTTCGAGGTCGGCTGGAAGGTC-----------------------------------------------------------------------------------------------------------------------------------------------      0.049     0.152     0.151
----------TTCGAGGTCGGCTGGAAGGTC-----------------------------------------------------------------------------------------------------------------------------------------------     89.705   352.682   815.667 miR_t115
----------TTCGAGGTCGGCTGGAAGGT------------------------------------------------------------------------------------------------------------------------------------------------      0.049     0.379     0.377
----------TTCGAGGTCGGCTGGAAGG-------------------------------------------------------------------------------------------------------------------------------------------------      0.097     1.099     0.791
-----------TCGAGGTCGGCTGGAAGGTC-----------------------------------------------------------------------------------------------------------------------------------------------      5.440    53.003    58.208
-----------TCGAGGTCGGCTGGAAGGTCG----------------------------------------------------------------------------------------------------------------------------------------------      0.097     1.440     0.226
-----------TCGAGGTCGGCTGGAAGG-------------------------------------------------------------------------------------------------------------------------------------------------      0.049     0.189     0.075
-------------GAGGTCGGCTGGAAGGTC-----------------------------------------------------------------------------------------------------------------------------------------------      0.049     0.076     0.301
-----------------TCGGCTGGAAGGTCGGCGGTG----------------------------------------------------------------------------------------------------------------------------------------      3.254     0.682     0.791
-----------------TCGGCTGGAAGGTCGGCGGT-----------------------------------------------------------------------------------------------------------------------------------------      0.146     0.188     0.188
------------------CGGCTGGAAGGTCGGCGGTG----------------------------------------------------------------------------------------------------------------------------------------      0.049     0.000     0.000
----------------------TGGAAGGTCGGCGGTGCCCTG-----------------------------------------------------------------------------------------------------------------------------------      3.740     2.349     2.071
----------------------TGGAAGGTCGGCGGTGCCCTGG----------------------------------------------------------------------------------------------------------------------------------      7.819     5.304     5.761
----------------------TGGAAGGTCGGCGGTGCCCT------------------------------------------------------------------------------------------------------------------------------------      0.049     0.000     0.000
----------------------TGGAAGGTCGGCGGTGCCC-------------------------------------------------------------------------------------------------------------------------------------      0.049     0.152     0.188
-----------------------------TCGGCGGTGCCCTGGTCTTTG----------------------------------------------------------------------------------------------------------------------------      0.146     0.189     0.866
------------------------------------TGCCCTGGTCTTTGTGAGC-----------------------------------------------------------------------------------------------------------------------      0.049     0.000     0.000
-----------------------------------------TGGTCTTTGTGAGCCAGGGGT----------------------------------------------------------------------------------------------------------------      0.437     0.076     0.075
--------------------------------------------------TGAGCCAGGGGTTCACCGTGC-------------------------------------------------------------------------------------------------------      3.448     0.076     0.151
--------------------------------------------------TGAGCCAGGGGTTCACCGTG--------------------------------------------------------------------------------------------------------      2.526     0.606     0.377
--------------------------------------------------------------TCACCGTGCTTCAGCTTGTGA-------------------------------------------------------------------------------------------      0.049     0.000     0.000
--------------------------------------------------------------------TGCTTCAGCTTGTGATATTTCGG-----------------------------------------------------------------------------------      0.049     0.758     0.758
----------------------------------------------------------------------CTTCAGCTTGTGATATTTCGG-----------------------------------------------------------------------------------      0.049     0.076     0.188
-----------------------------------------------------------------------TTCAGCTTGTGATATTTCGG-----------------------------------------------------------------------------------      6.557     9.396     3.351
-----------------------------------------------------------------------TTCAGCTTGTGATATTTCGGAT---------------------------------------------------------------------------------      0.049     0.682     0.038
-----------------------------------------------------------------------TTCAGCTTGTGATATTTCGGATG--------------------------------------------------------------------------------      0.049     0.038     0.038
-----------------------------------------------------------------------TTCAGCTTGTGATATTTCGGA----------------------------------------------------------------------------------      5.974    13.753     3.614
------------------------------------------------------------------------TCAGCTTGTGATATTTCGGATG--------------------------------------------------------------------------------      0.049     0.189     0.189
------------------------------------------------------------------------TCAGCTTGTGATATTTCGGAT---------------------------------------------------------------------------------      0.583     0.227     0.339
------------------------------------------------------------------------TCAGCTTGTGATATTTCGGA----------------------------------------------------------------------------------      4.420     3.751     1.318
------------------------------------------------------------------------TCAGCTTGTGATATTTCGG-----------------------------------------------------------------------------------      1.651     4.205     0.941
-------------------------------------------------------------------------CAGCTTGTGATATTTCGGATG--------------------------------------------------------------------------------      0.194     0.000     0.000
-------------------------------------------------------------------------CAGCTTGTGATATTTCGGA----------------------------------------------------------------------------------      0.146     0.114     0.114
--------------------------------------------------------------------------AGCTTGTGATATTTCGGATG--------------------------------------------------------------------------------      0.097     0.038     0.038
-----------------------------------------------------------------------------TTGTGATATTTCGGATGAACA----------------------------------------------------------------------------      0.971     0.644     0.151
-----------------------------------------------------------------------------TTGTGATATTTCGGATGAAC-----------------------------------------------------------------------------      0.291     0.758     0.414
------------------------------------------------------------------------------TGTGATATTTCGGATGAACAT---------------------------------------------------------------------------      0.097     0.038     0.038
------------------------------------------------------------------------------TGTGATATTTCGGATGAACA----------------------------------------------------------------------------      0.146     0.000     0.000
--------------------------------------------------------------------------------TGATATTTCGGATGAACATAA-------------------------------------------------------------------------      0.486     0.188     0.188
--------------------------------------------------------------------------------TGATATTTCGGATGAACAT---------------------------------------------------------------------------      0.049     0.000     0.000
-----------------------------------------------------------------------------------TATTTCGGATGAACATAAAGCA---------------------------------------------------------------------      0.049     0.000     0.000
-----------------------------------------------------------------------------------TATTTCGGATGAACATAAAGC----------------------------------------------------------------------     21.273    31.786    21.160
------------------------------------------------------------------------------------ATTTCGGATGAACATAAAGC----------------------------------------------------------------------      0.243     0.265     0.113
-------------------------------------------------------------------------------------TTTCGGATGAACATAAAGCAAG-------------------------------------------------------------------      4.663     2.728     0.452
-------------------------------------------------------------------------------------TTTCGGATGAACATAAAGCA---------------------------------------------------------------------      0.243     0.152     0.188
-------------------------------------------------------------------------------------TTTCGGATGAACATAAAGCAAGA------------------------------------------------------------------      0.194     0.038     0.151
--------------------------------------------------------------------------------------TTCGGATGAACATAAAGCAAG-------------------------------------------------------------------      0.243     0.833     0.075
---------------------------------------------------------------------------------------TCGGATGAACATAAAGCAAG-------------------------------------------------------------------      3.108    11.328     6.099
---------------------------------------------------------------------------------------TCGGATGAACATAAAGCAAGACTT---------------------------------------------------------------      0.097     1.174     0.075
---------------------------------------------------------------------------------------TCGGATGAACATAAAGCAAGAC-----------------------------------------------------------------      0.146     4.887     1.242
---------------------------------------------------------------------------------------TCGGATGAACATAAAGCAAGA------------------------------------------------------------------      0.243     0.303     0.113
---------------------------------------------------------------------------------------TCGGATGAACATAAAGCAAGACT----------------------------------------------------------------      0.680    15.003     0.678
-------------------------------------------------------------------------------------------ATGAACATAAAGCAAGACTTC--------------------------------------------------------------      0.340     3.031     0.301
--------------------------------------------------------------------------------------------TGAACATAAAGCAAGACTTCA-------------------------------------------------------------      8.354     0.341     0.452
--------------------------------------------------------------------------------------------TGAACATAAAGCAAGACTTCAA------------------------------------------------------------      0.049     0.000     0.000
--------------------------------------------------------------------------------------------TGAACATAAAGCAAGACTTCAAC-----------------------------------------------------------      0.291     0.038     0.075
--------------------------------------------------------------------------------------------TGAACATAAAGCAAGACTTC--------------------------------------------------------------     42.789    10.532     4.593

GC                                                                                                                              GCGTGAGCGCAACA-----   --     TG  ---   -    T
  CGAAGCTCTTCGAGGTCGGCTGGAAGGTCGGCGGTGCCCTGGTCTTTGTGAGCCAGGGGTTCACCGTGCTTCAGCTTGTGATATTTCGGATGAACATAAAGCAAGACTTCAACAGCCTCGCGGCCA                   GCC  GTCTG  CT   GAT CACA A
  GCTTCGAGAAGCTCCAGCCGACCTTCCAGCCGCCACGGGACCAGAAACACTCGGTCCCCAAGTGGCACGAAGTCGAACACTATAAAGCCTACTTGTATTTCGTTCTGAAGTTGTCGGAGCGCCGGT                   CGG  TAGAC  GG   CTA GTGT T
--                                                                                                                              CAAGGTTTGTACAAGGTAA   AT     GT  AGT   T    T

----------------------------------------------------------------------------------------GCCTACTTGTATTTCGTTCT------------------------------------------------------------------      0.097     0.038     0.038
--------------------------------------------------------------------------------------AAGCCTACTTGTATTTCGTTCT------------------------------------------------------------------      0.097     0.000     0.000
---------------------------------------------------------------------------CGAACACTATAAAGCCTACTT------------------------------------------------------------------------------      0.097     0.000     0.000
----------------------------------------------------------------------------GAACACTATAAAGCCTACTT------------------------------------------------------------------------------      0.097     0.000     0.000
-----------------------------------------------------------------------AAGTCGAACACTATAAAGCCT----------------------------------------------------------------------------------      4.371     0.000     0.000
-------------------------------------------------------------------------GTCGAACACTATAAAGCCT----------------------------------------------------------------------------------      0.049     0.000     0.000
------------------------------------------------------------------------AGTCGAACACTATAAAGCCT----------------------------------------------------------------------------------      4.857     0.379     0.339
----------------------------------------------------------------------GAAGTCGAACACTATAAAGCCT----------------------------------------------------------------------------------      0.389     0.265     0.265
----------------------------------------------------------------------GAAGTCGAACACTATAAAGC------------------------------------------------------------------------------------      0.049     0.000     0.000
------------------------------------------------------------------------AGTCGAACACTATAAAGC------------------------------------------------------------------------------------      0.049     0.000     0.000
-----------------------------------------------------------------------AAGTCGAACACTATAAAGC------------------------------------------------------------------------------------      0.049     0.000     0.000
-------------------------------------------------------GTCCCCAAGTGGCACGAAGT---------------------------------------------------------------------------------------------------      0.146     0.075     0.075
-----------------------------------------------------CGGTCCCCAAGTGGCACGAAGT---------------------------------------------------------------------------------------------------      0.049     0.189     0.189
------------------------------------------------------GGTCCCCAAGTGGCACGAAGT---------------------------------------------------------------------------------------------------      0.389     0.038     0.113
--------------------------------------------AGAAACACTCGGTCCCCAAGT-------------------------------------------------------------------------------------------------------------      0.097     0.000     0.000
-------------------------------------CGGGACCAGAAACACTCGGT---------------------------------------------------------------------------------------------------------------------      0.097     0.038     0.038
--------------------------------CGCCACGGGACCAGAAACACT-------------------------------------------------------------------------------------------------------------------------      0.049     0.152     0.188
------------------------------GCCGCCACGGGACCAGAAAC----------------------------------------------------------------------------------------------------------------------------      0.049     0.076     0.076
------CGAGAAGCTCCAGCCGACCTT---------------------------------------------------------------------------------------------------------------------------------------------------      1.117    26.558    22.590
```

miRNAs identified in HS+N and HS-N :

miRNA: miR_t6 chromosome_17 Start: 5228339 End: 5228527 Condition: HS+N

```
----TAAGGAGAACATCAAGGAGC---------------------------------------------------------------------------     0.146     0.226     0.226
-----AAGGAGAACATCAAGGAGC---------------------------------------------------------------------------     0.000     0.038     0.038
-----AAGGAGAACATCAAGGAGCTG-------------------------------------------------------------------------     0.631     0.076     0.527
-----AAGGAGAACATCAAGGAGCTGC------------------------------------------------------------------------     0.097     1.137     1.318
------AGGAGAACATCAAGGAGCTGC------------------------------------------------------------------------     0.000     0.075     0.075
--------GAGAACATCAAGGAGCTGCG-----------------------------------------------------------------------     0.049     0.075     0.075
--------GAGAACATCAAGGAGCTGCGG----------------------------------------------------------------------     39.340    49.934    78.088 miR_t6
--------GAGAACATCAAGGAGCTGCGGC---------------------------------------------------------------------     1.457     1.970     1.468
---------AGAACATCAAGGAGCTGCGG----------------------------------------------------------------------     0.874     2.804     1.431
---------AGAACATCAAGGAGCTGCGGC---------------------------------------------------------------------     0.194     0.871     1.017
----------GAACATCAAGGAGCTGCGG----------------------------------------------------------------------     0.049     0.038     0.038
------------------------TGCGGCTTTACCTTTTCTTCCTGT---------------------------------------------------     7.334     3.599     1.581
------------------------TGCGGCTTTACCTTTTCTTCCTG----------------------------------------------------     1.166     0.265     0.038
------------------------------TTTACCTTTTCTTCCTGTTGGC-----------------------------------------------     0.874     3.220     0.791
------------------------------TTTACCTTTTCTTCCTGTTGGCG----------------------------------------------     5.440     1.099     0.226
------------------------------TTTACCTTTTCTTCCTGTTGG------------------------------------------------     0.340     0.075     0.075
-------------------------------TTACCTTTTCTTCCTGTTGGC-----------------------------------------------     0.291     0.076     0.188
-------------------------------TTACCTTTTCTTCCTGTTGGCG----------------------------------------------     0.049     0.038     0.075
-------------------------------TTACCTTTTCTTCCTGTTGGCGG---------------------------------------------     1.020     0.455     0.075
---------------------------------------------TGTTGGCGGGGATGCTCTTG----------------------------------     0.049     0.038     0.038
---------------------------------------------TGTTGGCGGGGATGCTCTTGA---------------------------------     1.797     0.076     0.188
---------------------------------------------------------TGCTCTTGAGCGTCACTCCC----------------------     0.049     0.113     0.113
---------------------------------------------------------TGCTCTTGAGCGTCACTCCCG---------------------     0.389     0.417     0.226

CAA  A     A                          TTCT                                          T- T    --  TG
   CT AGGAG ACATCAAGGAGCTGCGGCTTTACCTT    TCCTGTTGGCGGGGATGCTCTTGAGCGTCACTCCCGAGAGCA  C CGGG  AG  C
   GA TCCTC TGTAGTTCCTCGACGCTGAAATGGAA    AGGACAACCGCCCCTACGAGGATTCGCAGTGAGGGCTCTCGT  G GTCC  TC  /
---  A     C                          C---                                          TC C    GT  GG

---------------------------------------------------GCCCCTACGAGGATTCGCAGT---------------------------     4.517     1.137     2.146
----------------------------------------------------CCCCTACGAGGATTCGCAGT---------------------------     0.194     0.075     0.075
-----------------------------GAAATGGAAC---AGGACAACCGC----------------------------------------------     0.291     0.038     0.075
-------------GTAGTTCCTCGACGCTGAAAT-----------------------------------------------------------------     4.080     1.402     0.565
---------TCCTGTAGTTCCTCGACGCT----------------------------------------------------------------------     0.097     0.038     0.038
-------CCTCCTGTAGTTCCTCGACGCT----------------------------------------------------------------------     0.340     0.114     0.151
--------CTCCTGTAGTTCCTCGACGCT----------------------------------------------------------------------     0.049     0.038     0.038
```

miRNA: miR_t14 chromosome_4 Start: 457760 End: 457979 Condition: HS+N

```
---------------------------------------------------------------------------------------------TGGTGGAGGAGGAGGACGGG-     0.000     0.038     0.038
---------------------------------------------------------------------------------------------TGGTGGAGGAGGAGGACGG--     0.000     0.265     0.075
---------------------------------------------------------------------------------------------TGGTGGAGGAGGAGGAC----     0.000     0.038     0.038
----------------------------------------------------------------------------------------GCTGCTGGTGGAGGAGGAGG------     0.000     0.379     0.188
---------------------------------------------------------------------------------------TGCTGCTGGTGGAGGAGG---------     0.000     0.038     0.038
---------------------------------------------------------------------------------------TGCTGCTGGTGGAGGAGGAGG------     2.331     42.811    11.408
---------------------------------------------------------------------------------------TGCTGCTGGTGGAGGAGGAG-------     0.049     0.493     0.188
----------------------------------------------------------------------------CAGCGCGGGGCTGCTGCTGG------------------     0.000     0.038     0.038
---------------------------------------------------------------------CAGCAGGCAGCGCGGGGCTG-------------------------     0.000     0.113     0.113
---------------------------------------------------------------------CAGCAGGCAGCGCGGGGCT--------------------------     0.000     0.038     0.038
---------------------------------------------------------------------CAGCAGGCAGCGCGGGGCTGC------------------------     0.291     8.790     5.121
---------------------------------------------------------------------CAGCAGGCAGCGCGGGGCTGCT-----------------------     0.000     0.303     0.377
--------------------------------------------------------------CGGCAGGCAGCAGGCAGCGCGG------------------------------     0.000     0.265     0.075
--------------------------------------------------------------CGGCAGGCAGCAGGCAGCGCG-------------------------------     0.194     2.652     1.355
------------------------------------------------------------TGCGGCAGGCAGCAGGCAGCGCG-------------------------------     0.777     1.705     0.113
------------------------------------------------------------TGCGGCAGGCAGCAGGCAGCGC--------------------------------     0.000     0.038     0.038
------------------------------------------------------------TGCGGCAGGCAGCAGGCAGCG---------------------------------     0.000     0.038     0.038
------------------------------------------------------TAGTGTTGCGGCAGGCAGCAGG--------------------------------------     0.389     3.448     0.565
------------------------------------------------------TAGTGTTGCGGCAGGCAGCAG---------------------------------------     0.194     0.417     0.452
------------------------------------------------------TAGTGTTGCGGCAGGCAGCAGGC-------------------------------------     0.000     0.455     0.038
------------------------------------------------------TAGTGTTGCGGCAGGCAG------------------------------------------     0.000     0.038     0.038
---------------------------------------------------GGGTAGTGTTGCGGCAGGC--------------------------------------------     0.000     0.038     0.038
---------------------------------------------------GGGTAGTGTTGCGGCAGGCAGC-----------------------------------------     0.437     8.297     0.527
---------------------------------------------------GGGTAGTGTTGCGGCAGGCAG------------------------------------------     0.437     6.706     0.264
---------------------------------------------------GGGTAGTGTTGCGGCAGGCA-------------------------------------------     0.000     0.152     0.339
--------------------------------------------------TGGGTAGTGTTGCGGCAG----------------------------------------------     0.000     0.152     0.113
--------------------------------------------------TGGGTAGTGTTGCGGCAGG---------------------------------------------     0.097     0.076     0.075
--------------------------------------------------TGGGTAGTGTTGCGGCA-----------------------------------------------     0.000     0.114     0.151
--------------------------------------------------TGGGTAGTGTTGCGGCAGGCA-------------------------------------------     1.166     13.525    11.596
--------------------------------------------------TGGGTAGTGTTGCGGCAGGCAG------------------------------------------     32.249    539.725   5.083
--------------------------------------------------TGGGTAGTGTTGCGGCAGGC--------------------------------------------     0.000     0.379     0.414
--------------------------------------------------TGGGTAGTGTTGCGGCAGGCAGC-----------------------------------------     18.699    475.129   7.530
------------------------------------------------GGTGGGTAGTGTTGCGGCAGG---------------------------------------------     0.000     0.038     0.038
-----------------------------------------------AGGTGGGTAGTGTTGCGGCAG----------------------------------------------     0.097     0.265     0.527
-----------------------------------------------AGGTGGGTAGTGTTGCGGCAGG---------------------------------------------     0.097     0.417     0.527
-------------------------------------------CAGGAGGTGGGTAGTGTTGCGG-------------------------------------------------     0.000     0.152     0.038
-------------------------------------------CAGGAGGTGGGTAGTGTTGCGGCAGGCAG------------------------------------------     0.000     0.644     0.038
-------------------------------------------CAGGAGGTGGGTAGTGT------------------------------------------------------     0.000     0.076     0.151
-----------------------------------------CGCAGGAGGTGGGTAGTGTTG----------------------------------------------------     1.263     5.039     1.130
-----------------------------------------CGCAGGAGGTGGGTAGTGTT-----------------------------------------------------     0.583     5.115     1.355
-----------------------------------------CGCAGGAGGTGGGTAGT--------------------------------------------------------     0.000     0.038     0.038
----------------------------------------ACGCAGGAGGTGGGTAGTGTT-----------------------------------------------------     0.000     0.151     0.151
---------------------------------ACCGGGCACGCAGGAGGTG--------------------------------------------------------------     0.340     0.301     0.301
---------------------------------ACCGGGCACGCAGGAGGTGGG------------------------------------------------------------     0.000     0.075     0.075
--------------------------------CACCGGGCACGCAGGAGGTGG-------------------------------------------------------------     0.000     0.417     0.226
--------------------------------CACCGGGCACGCAGGAGGTGGG------------------------------------------------------------     0.049     0.189     0.075
--------------------------------CACCGGGCACGCAGGAGGTG--------------------------------------------------------------     0.000     0.114     0.038
--------------------------------CACCGGGCACGCAGGAGG----------------------------------------------------------------     0.000     0.265     0.075
-------------------------------GCACCGGGCACGCAGGAGG----------------------------------------------------------------     0.097     0.038     0.226
------------------------------GGCACCGGGCACGCAGGAGGT---------------------------------------------------------------     0.000     0.189     0.038
------------------------------GGCACCGGGCACGCAGGAGG----------------------------------------------------------------     0.583     5.531     5.196
------------------------------GGCACCGGGCACGCAGGAG-----------------------------------------------------------------     0.000     0.189     0.264
-----------------------------TGGCACCGGGCACGCAGGA------------------------------------------------------------------     0.194     1.781     0.715
-----------------------------TGGCACCGGGCACGCAGGAGGT---------------------------------------------------------------     10.005    18.072    6.024
-----------------------------TGGCACCGGGCACGCAGGAGG----------------------------------------------------------------     146.239   573.861   426.207
-----------------------------TGGCACCGGGCACGCAGG-------------------------------------------------------------------     0.194     1.743     0.264
-----------------------------TGGCACCGGGCACGCAG--------------------------------------------------------------------     0.097     0.379     0.113
-----------------------------TGGCACCGGGCACGCAGGAG-----------------------------------------------------------------     5.440     35.196    18.411
----------------------------TTGGCACCGGGCACGCAGGAGG----------------------------------------------------------------     321.958   3361.064  849.402
----------------------------TTGGCACCGGGCACGCAG--------------------------------------------------------------------     0.437     1.478     1.092
----------------------------TTGGCACCGGGCACGCA---------------------------------------------------------------------     0.049     0.455     0.188
----------------------------TTGGCACCGGGCACGCAGG-------------------------------------------------------------------     0.534     2.879     1.694
----------------------------TTGGCACCGGGCACGCAGGA------------------------------------------------------------------     0.486     2.160     1.581
----------------------------TTGGCACCGGGCACGCAGGAGGT---------------------------------------------------------------     30.161    163.554   8.095
----------------------------TTGGCACCGGGCACGCAGGAG-----------------------------------------------------------------     7.479     108.582   46.273
---------------------------CTTGGCACCGGGCACGCAGGAGG----------------------------------------------------------------     0.097     2.500     0.489
---------------------------CTTGGCACCGGGCACGCAGGAGGT---------------------------------------------------------------     0.000     0.227     0.113
-----TGGACTGCTGGAGTGCAGC------------------------------------------------------------------------------------------     0.000     0.038     0.038
----TTGGACTGCTGGAGTGCAGCAGG---------------------------------------------------------------------------------------     1.506     44.781    0.301
----TTGGACTGCTGGAGTGCAGCAGGC--------------------------------------------------------------------------------------     3.254     298.959   1.506
----TTGGACTGCTGGAGTGCAGC------------------------------------------------------------------------------------------     0.291     14.207    0.828
----TTGGACTGCTGGAGTGCAGCA-----------------------------------------------------------------------------------------     0.243     4.546     0.301
----TTGGACTGCTGGAGTGC---------------------------------------------------------------------------------------------     0.000     2.538     0.075
----TTGGACTGCTGGAGTGCAGCAG----------------------------------------------------------------------------------------     8.111     38.985    2.071
----TTGGACTGCTGGAGTGCAG-------------------------------------------------------------------------------------------     0.049     0.189     0.188
----TTGGACTGCTGGAGTGCA--------------------------------------------------------------------------------------------     0.389     4.130     0.264

GG          CT  A  GCA          C        C     A            T C  C   C                   C     G   A            G
  CGTTGGACTG  GG GT   GCAGGCTTGG ACCGGGCA GCAGG GGTGGGTAGTGT G GG AGG AGCAGGCAGCGCGGGGCTG TGCTG TGG GGAGGAGGACGG C
  GCAACCTGAC  CC CA   TGTCCGAACC TGGCCCGT CGTCC CCACCCATCACA C CC TCC TCGTCCGTCGCGCCCCGAC ACGAC ACC CCTTCTCCTGTC C
--          --  -  ---          A        A     C            C A  -   A                   C     A   A            C

------CCTGAC--CC-CA---TGTCCGAACCAT--------------------------------------------------------------------------------     0.000     0.038     0.038
------------------A---TGTCCGAACCATGGCCCGT-------------------------------------------------------------------------     0.000     0.189     0.038
-----------------------GTCCGAACCATGGCCCGTACG----------------------------------------------------------------------     0.000     0.038     0.038
-----------------------GTCCGAACCATGGCCCGTACGT---------------------------------------------------------------------     0.049     0.379     0.414
------------------------TCCGAACCATGGCCCGTACGT---------------------------------------------------------------------     0.729     1.629     0.377
--------------------------CGAACCATGGCCCGTACGT---------------------------------------------------------------------     0.000     0.682     0.301
-------------------------CCGAACCATGGCCCGTACGT---------------------------------------------------------------------     0.243     2.160     0.377
----------------------TGTCCGAACCATGGCCCGTACGT---------------------------------------------------------------------     0.000     0.075     0.075
--------------------------CGAACCATGGCCCGTACGTCCC------------------------------------------------------------------     0.000     0.341     0.038
----------------------------------GGCCCGTACGTCCCCCACCCAT----------------------------------------------------------     0.000     0.038     0.038
--------------------------------------CGTACGTCCCCCACCCAT----------------------------------------------------------     0.000     0.038     0.038
---------------------------------------GTACGTCCCCCACCCATCAC-------------------------------------------------------     0.049     0.303     0.264
--------------------------------------CGTACGTCCCCCACCCATCAC-------------------------------------------------------     0.049     0.530     0.038
---------------------------------------GTACGTCCCCCACCCATCACAC-----------------------------------------------------     0.000     0.227     0.188
---------------------------------------------CCCCCACCCATCACACCAC--------------------------------------------------     0.000     0.075     0.075
---------------------------------------------CCCCCACCCATCACACCACC-T-----------------------------------------------     1.651     2.993     1.242
------------------------------------------------CCACCCATCACACCACC-TC----------------------------------------------     0.291     0.075     0.075
-----------------------------------------------CCCACCCATCACACCACC-TCC---------------------------------------------     0.000     0.038     0.038
-------------------------------------------------CACCCATCACACCACC-TCC---------------------------------------------     0.049     0.152     0.038
------------------------------------------------CCACCCATCACACCACC-TCC---------------------------------------------     0.437     0.796     0.678
-----------------------------------------------CCCACCCATCACACCACC-TCCA--------------------------------------------     0.097     0.189     0.151
-------------------------------------------------CACCCATCACACCACC-TCCA--------------------------------------------     0.000     0.114     0.113
---------------------------------------------------CCCATCACACCACC-TCCA--------------------------------------------     0.000     0.038     0.075
------------------------------------------------CCACCCATCACACCACC-TCCA--------------------------------------------     2.186     4.887     7.417
--------------------------------------------------ACCCATCACACCACC-TCCA--------------------------------------------     0.000     0.075     0.075
---------------------------------------------CCCCCACCCATCACACCACC-TCCAT-------------------------------------------     0.000     0.076     0.038
----------------------------------------------CCCCACCCATCACACCACC-TCCAT-------------------------------------------     0.000     0.114     0.038
----------------------------------------------------CCATCACACCACC-TCCAT-------------------------------------------     0.243     0.038     0.188
--------------------------------------------------ACCCATCACACCACC-TCCAT-------------------------------------------     1.846     7.994     2.636
-----------------------------------------------CCCACCCATCACACCACC-TCCAT-------------------------------------------     7.771     20.458    16.077
---------------------------------------------------CCCATCACACCACC-TCCAT-------------------------------------------     0.437     3.410     0.452
------------------------------------------------CCACCCATCACACCACC-TCCAT-------------------------------------------     319.627   814.702   445.296 miR_t14
-------------------------------------------------CACCCATCACACCACC-TCCAT-------------------------------------------     14.910    23.376    12.086
----------------------------------------------------CCATCACACCACC-TCCATC------------------------------------------     0.000     0.038     0.038
------------------------------------------------CCACCCATCACACCACC-TCCATC------------------------------------------     0.243     0.114     0.075
-----------------------------------------------------CATCACACCACC-TCCATCGT----------------------------------------     4.857     4.812     1.506
----------------------------------------------------CCATCACACCACC-TCCATCGT----------------------------------------     4.323     5.493     2.636
-----------------------------------------------CCCACCCATCACACCACC-TCCATCGT----------------------------------------     0.000     0.038     0.038
-------------------------------------------------------TCACACCACC-TCCATCGT----------------------------------------     0.000     0.113     0.113
----------------------------------------------------------CACCACC-TCCATCGTCCGT------------------------------------     0.000     0.038     0.038
--------------------------------------------------------CACACCACC-TCCATCGTCCGT------------------------------------     0.194     0.038     0.038
-------------------------------------------------------TCACACCACC-TCCATCGTCCGT------------------------------------     0.194     0.455     0.151
---------------------------------------------------------------------------------GCCCCGACCACGACAACCAC-------------     0.000     0.075     0.075
-------------------------------------------------------------------------------------CGACCACGACAACCACCTTCT--------     1.214     1.023     0.828
```

miRNA: miR_t21,miR_t80 chromosome_2 Start: 9129502 End: 9129598 Condition: HS+N

```
,Äî,Äî,ÄîCGGCTAGAGGGCGCAAGCACGC---------------------      0.049      2.008      0.904
-------CGGCTAGAGGGCGCAAGCACG----------------------      0.146      0.568      0.452 miR_t80*
--------GGCTAGAGGGCGCAAGCACGC---------------------      0.000      0.038      0.038
----------CTAGAGGGCGCAAGCACGC---------------------      0.000      0.038      0.038
----------CTAGAGGGCGCAAGCACGCA--------------------      0.097      0.038      0.113
-----------TAGAGGGCGCAAGCACGCAAGG-----------------      0.583      0.341      0.452
-----------TAGAGGGCGCAAGCACG----------------------      0.000      0.114      0.075
-----------TAGAGGGCGCAAGCACGCAAG------------------     31.424     17.693     25.527
-----------TAGAGGGCGCAAGCACGC---------------------      6.897     32.620     30.384
-----------TAGAGGGCGCAAGCACGCAA-------------------      1.700      2.841      3.502
-----------TAGAGGGCGCAAGCACGCA--------------------     38.757     60.163    109.602 miR_t21
------------AGAGGGCGCAAGCACGCAA-------------------      0.000      0.076      0.113
------------AGAGGGCGCAAGCACGCA--------------------      0.291      0.152      1.355
------------AGAGGGCGCAAGCACGC---------------------      0.000      0.530      0.414
------------AGAGGGCGCAAGCACGCAAG------------------      0.437      0.114      0.264
-------------GAGGGCGCAAGCACGCAAG------------------      0.049      0.076      0.038
-------------GAGGGCGCAAGCACGCA--------------------      0.049      0.038      0.038

---T                                           AT
    AGACGGCTAGAGGGCGCAAGCACGCAAGGAGGGGATGACGAGC  G
    TCTGCCGATCTCCCGCGTTCGTGCGTTCCTCCCCTACTGCTCG  /
TGGC                                           TT

--------------------------GCGTTCCTCCCCTACTGC------      0.000      0.075      0.075
-------------------CGTTCGTGCGTTCCTCCCCT-----------      0.194      0.151      0.151
---------CGATCTCCCGCGTTCGTGCGTT-------------------      0.583      0.493      0.151
----------GATCTCCCGCGTTCGTGCGTT-------------------      0.146      0.076      0.038
---------CGATCTCCCGCGTTCGTGCGT--------------------      2.186      0.985      1.205
----------GATCTCCCGCGTTCGTGCGT--------------------      0.000      0.038      0.038
-------GCCGATCTCCCGCGTTCGTGC----------------------      4.420      1.212      0.301
---------CGATCTCCCGCGTTCGTGC----------------------      0.000      0.227      0.075 miR_t21*
-------GCCGATCTCCCGCGTTCGTG-----------------------      0.631      0.038      0.038
------TGCCGATCTCCCGCGTTCGT------------------------      2.234      0.227      0.339
-----CTGCCGATCTCCCGCGTTCGT------------------------    434.928    229.211    264.610 miR_t80
--------CCGATCTCCCGCGTTCGT------------------------      0.194      0.189      0.038
-------GCCGATCTCCCGCGTTCGT------------------------     11.414      2.955      1.958
----TCTGCCGATCTCCCGCGTTCGT------------------------      1.068      0.189      0.188
---------CGATCTCCCGCGTTCGT------------------------      0.291      0.075      0.075
-----CTGCCGATCTCCCGCGTTCG-------------------------      6.071      3.031      4.593
-----CTGCCGATCTCCCGCGTT---------------------------      0.000      0.075      0.075

miRNA: miR_t22 chromosome_13 Start: 2000553 End: 2000712 Condition: HS-N
------------------------AATAACGGTACGATGCTATGA-----------------------------------------------      0.000      0.076      0.038
-------------------------ATAACGGTACGATGCTATGAA----------------------------------------------      0.097      0.303      0.303
--------------------------TAACGGTACGATGCTATGAAA---------------------------------------------      2.283      0.606      1.544
--------------------------TAACGGTACGATGCTATGAAAA--------------------------------------------      0.146      0.455      0.455
--------------------------TAACGGTACGATGCTATGAA----------------------------------------------      0.097      0.152      0.602
---------------------------AACGGTACGATGCTATGAAA---------------------------------------------      0.000      0.038      0.038
--------------------------------TACGATGCTATGAAAAGGAAC---------------------------------------      1.748      0.833      0.038
-------------------------------------TGCTATGAAAAGGAACGGGGC----------------------------------      0.000      0.038      0.264
-------------------------------------TGCTATGAAAAGGAACGGG------------------------------------      0.000      0.076      0.076
-------------------------------------TGCTATGAAAAGGAACGGGGCG---------------------------------      0.000      0.038      0.038
----------------------------------------TATGAAAAGGAACGGGGCGTG-------------------------------     13.016      0.682      2.071
----------------------------------------TATGAAAAGGAACGGGGCG---------------------------------      0.000      0.038      0.038
------------------------------------------TGAAAAGGAACGGGGCGTGTGG----------------------------      1.554      0.227      0.226
--------------------------------------------AAAAGGAACGGGGCGTG-------------------------------      0.000      0.038      0.038
--------------------------------------------AAAAGGAACGGGGCGTGTGG----------------------------      0.243      0.682      1.205
---------------------------------------------AAAGGAACGGGGCGTGTGGTG--------------------------      0.097      0.114      0.151
----------------------------------------------AAGGAACGGGGCGTGTGGTGG-------------------------      0.000      0.038      0.113
-----------------------------------------------------------------TGGGCCTGGAGGGGG-CCAG-CGGATG      0.389      0.152      0.038

--------------------       A                          G                         -   G-    T
                    GAACAAT ACGGTACGATGCTATGAAAAGGAACG GGCGTGTGGTGGGCCTGGAGGGGG CCA  CGGA G
                    CTTGTTA TGCCATGCTACGATACTTTTCCTTGC CCGCATACCACCCGGACCTCCCCC GGT  GTTT /
GAACAGGCGTTGAGTTCTCA       A                          G                         C   AA    -

------------------------------CCATGCTACGATACTTTTCCTT----------------------------------------      0.000      0.038      0.038
--------------------------AATGCCATGCTACGATACTTT---------------------------------------------      0.486      0.038      0.188
-------------------------TAATGCCATGCTACGATACTTT---------------------------------------------      0.291      0.076      0.038
-----------------------GTTAATGCCATGCTACGATACT-----------------------------------------------      0.194      0.076      0.076
----------------------TGTTAATGCCATGCTACGATACT-----------------------------------------------      0.049      0.038      0.038
------------------------TTAATGCCATGCTACGATACT-----------------------------------------------      0.291      0.038      0.301
-------------------------TAATGCCATGCTACGATACT-----------------------------------------------      0.631      0.114      0.114
-----------------------GTTAATGCCATGCTACGATAC------------------------------------------------      0.049      0.076      0.076
----------------------TGTTAATGCCATGCTACGATAC------------------------------------------------      0.000      0.038      0.038
------------------------TTAATGCCATGCTACGAT--------------------------------------------------      0.049      0.038      0.038
----------------------TGTTAATGCCATGCTACGAT--------------------------------------------------     21.807    211.670    125.415 miR_t22
---------------------TTGTTAATGCCATGCTACGAT--------------------------------------------------      9.956      5.721      2.184
---------------------TTGTTAATGCCATGCTACGA---------------------------------------------------      0.097      0.076      0.076
----------------------TGTTAATGCCATGCTACGA---------------------------------------------------      0.291      1.705      1.167
----------------------TGTTAATGCCATGCTAC-----------------------------------------------------      0.000      0.038      0.038
----AGGCGTTGAGTTCTCACTTGTT------------------------------------------------------------------      0.097      0.038      0.188

miRNA: miR_t23 chromosome_13 Start: 2000575 End: 2000715 Condition: HS+N
-----------------------------AAGGAACGCGGCGTATGGTGG------------------------      0.000      0.038      0.038
---------------------------AAAAGGAACGCGGCGTATGG---------------------------      0.049      0.076      0.151
-------------------------TGAAAAGGAACGCGGCGTATG----------------------------      1.360      1.591      2.108
-------------------------TGAAAAGGAACGCGGCGTATGG---------------------------      0.389      0.265      0.151
-----------------------TATGAAAAGGAACGCGGCGTA------------------------------      0.000      0.038      0.038
---------------TACGATGCTATGAAAAGGAAC--------------------------------------      1.748      0.833      0.038
-----------ACGGTACGATGCTATGAAA--------------------------------------------      0.000      0.038      0.038
---------TTACGGTACGATGCTATGAAAA-------------------------------------------      0.000      0.038      0.038

-----     T                          C     A                  G   TTCAAA
     ACAAT ACGGTACGATGCTATGAAAAGGAACG GGCGT TGGTGGGCCTGGAGGGGG CCA      C
     TGTTA TGCCATGCTACGATACTTTTCCTTGC CCGCA ACCACCCGGACCTCCCCC GGT      C
ACGCT     T                          C     C                  -   CCCCCC

-----TGTTATTGCCATGCTACGA--------------------------------------------------      0.049      0.493      0.377
----TTGTTATTGCCATGCTACGAT-------------------------------------------------      0.729      1.023      0.188
-----TGTTATTGCCATGCTACGAT-------------------------------------------------      4.711     48.115     27.372 miR_t23
-----TGTTATTGCCATGCTACGATA------------------------------------------------      0.000      0.075      0.075

miRNA: miR_t24 chromosome_11 Start: 1130253 End: 1130529 Condition: HS-N
----TGCCGTAGCAGCATTCTTGGAT--------------------------------------------------------------------------------------------------------------------------------------------------------------------------------------------------------      0.000      0.038      0.038
---------TAGCAGCATTCTTGGATTCAG----------------------------------------------------------------------------------------------------------------------------------------------------------------------------------------------------      0.291      0.114      0.075
-----------------TTCTTGGATTCAGTCAACGGTAGGGCC----------------------------------------------------------------------------------------------------------------------------------------------------------------------------------      0.000      0.038      0.038
----------------------------------------------------------------------------------------------------------------------------------------------------------------------------------TGCGCCCCCGTCTACATGGC----------------------------      0.000      0.038      0.038

CTGTTGCCGTAGCAGCATTCTTGGATTCAGTCAACGGTAGGGCCCGGAGGAGGACTGATTCCAAGAATGCTGCTACGGGAGGACCCGAAGGAGGCAGGGGATGCGGGGACGGATGACAGGAATGCATGTCGTGTGCACCACCTTGTAACCACAGACAACACAACAGGATGAGT                            T T  AGA    -         TA
                                                                                                                                                                             GAGTATGCGCCCCCGTCTACATGGCTAT G CC   TTGC TGCCGCGGC  C
                                                                                                                                                                             CTCATACGCGGGGGCAGATGTACCGATA C GG   AATG GTGGCGCCG  /
-----------------------------------------------------------------------------------------------------------------------------------------------------------------------------                            T C  AG-    C         TT

---------------------------------------------------------------------------------------------------------------------------------------------------------------------------------CGGGGGCAGATGTACCGATAT------------------------      0.000      0.189      0.565
--------------------------------------------------------------------------------------------------------------------------------------------------------------------------------GCGGGGGCAGATGTACCGATAT------------------------      0.000      0.076      0.076
-------------------------------------------------------------------------------------------------------------------------------------------------------------------------------CGCGGGGGCAGATGTACCGAT--------------------------     34.872     28.642     64.232 miR_t24
-----------------------------------------------------------------------------------------------------------------------------------------------------------------------------TACGCGGGGGCAGATGTACCGAT--------------------------      0.097      0.114      1.130
------------------------------------------------------------------------------------------------------------------------------------------------------------------------------ACGCGGGGGCAGATGTACCGAT--------------------------      3.448      1.137      2.146
----------------------------------------------------------------------------------------------------------------------------------------------------------------------------------GGGGGCAGATGTACCGAT--------------------------      0.000      0.038      0.038
-------------------------------------------------------------------------------------------------------------------------------------------------------------------------------CGCGGGGGCAGATGTACCGA---------------------------      0.146      0.114      0.602

miRNA: miR_t28 chromosome_2 Start: 9207620 End: 9207907 Condition: HS-N
----------------------------------------------------------------------------------------------------------------------TATGCTCTGACTACC-CGAC-----------------      0.000      0.152      0.113
----------------------------------------------------------------------------------------------------------------------TATGCTCTGACTACC-CGACAAGCAAGC---------      0.000      0.341      0.341
----------------------------------------------------------------------------------------------------------------------TATGCTCTGACTACC-CGACAAGC-------------      0.000      0.189      0.189
--------------------------------------------------------------------------------------------------------------------GCTATGCTCTGACTACC-CGACAAGC-------------      0.000      0.076      0.076
-------------------------------------------------------------------------------------------------------------------TGCTATGCTCTGACTACC-CGACAAG--------------      0.000      0.871      0.871
-------------------------------------------------------------------------------------------------------------------TGCTATGCTCTGACTACC-CGA------------------      0.049      0.038      0.038
-------------------------------------------------------------------------------------------------------------------TGCTATGCTCTGACTACC-CGAC-----------------      0.049      1.932      0.075
-------------------------------------------------------------------------------------------------------------------TGCTATGCTCTGACTACC-CGACAAGCAAGC---------      0.000      1.553      0.301
-------------------------------------------------------------------------------------------------------------------TGCTATGCTCTGACTACC-CGACA----------------      0.000      0.152      0.152
-------------------------------------------------------------------------------------------------------------------TGCTATGCTCTGACTACC-CGACAAGCAAG----------      0.000      0.038      0.038
-------------------------------------------------------------------------------------------------------------------TGCTATGCTCTGACTACC----------------------      0.000      0.265      0.151
-------------------------------------------------------------------------------------------------------------------TGCTATGCTCTGACTACC-CGACAAGC-------------      0.000      3.940      3.940
-------------------------------------------------------------------------------------------------------------------TGCTATGCTCTGACTACC-CGACAA---------------      0.000      0.038      0.038
-----------------------------------------------------------------------------------------------------------------GGTGCTATGCTCTGACTACC----------------------      0.000      0.114      0.114
----------------------------------------------------------------------------------------------------------------TGGTGCTATGCTCTGACTACC----------------------     12.725     27.960      3.765
----------------------------------------------------------------------------------------------------------------TGGTGCTATGCTCTGACTAC-----------------------      0.000      0.189      0.189
-------------------------------------------------------------------------------------------------------------TGCTGGTGCTATGCTCTGACT-------------------------      0.146      0.417      0.075
------------------------------------------------------------------------------------------------------------TTGCTGGTGCTATGCTCTGACT-------------------------      0.000      0.189      0.189
------------------------------------------------------------------------------------------------------------TTGCTGGTGCTATGCTCTGAC--------------------------      0.000      0.152      0.152
---------------------------------------------------------------------------------------------------TCGGCCCAGTTGCTGGTGC-------------------------------------      0.000      0.114      0.114
----------------------------------------------------------------------------------------------G-ACCTCGGCCCAGTTGCTGG----------------------------------------      0.097      0.265      0.452
-------------------------------------------------------------------------------ACACACTCAGTCCGGGG-ACC-------------------------------------------------------      0.000      0.303      0.303
------------------------------------------------------------------------------CACACACTCAGTCCGGGG-ACC-------------------------------------------------------      0.049      0.265      0.265
-----------------------------------------------------------------------------TCACACACTCAGTCCGGGG-AC--------------------------------------------------------      0.000      0.568      0.568
-----------------------------------------------------------------------------TCACACACTCAGTCCGGGG-ACC-------------------------------------------------------      1.797     34.476      0.753
-----------------------------------------------------------------------------TCACACACTCAGTCCGGGG-ACCTCGGC--------------------------------------------------      0.000      0.038      0.038
-----------------------------------------------------------------------------TCACACACTCAGTCCGG-------------------------------------------------------------      0.000      0.530      0.530
-----------------------------------------------------------------------------TCACACACTCAGTCCGGG------------------------------------------------------------      0.000      0.038      0.038
-----------------------------------------------------------------------------TCACACACTCAGTCCGGGG-ACCT------------------------------------------------------      0.000      0.076      0.076
--------------------------------------------------------------------------TGGTCACACACTCAGTCCGGG------------------------------------------------------------      0.049      0.038      0.038
--------------------------------------------------------------------------TGGTCACACACTCAGTCCGGGG-ACC-------------------------------------------------------      0.000      0.189      0.189
--------------------------------------------------------------------------TGGTCACACACTCAGTCCGG-------------------------------------------------------------      1.991      5.493      0.828
--------------------------------------------------------------------------TGGTCACACACTCAGTCC---------------------------------------------------------------      0.049      0.038      0.075
--------------------------------------------------------------------------TGGTCACACACTCAGTCCG--------------------------------------------------------------      0.000      0.114      0.114
-------------------------------------------------------------------------GTGGTCACACACTCAGTCCG--------------------------------------------------------------      0.000      0.038      0.038
------------------------------------------------------------------------TGTGGTCACACACTCAGTCCG--------------------------------------------------------------      0.000      0.038      0.038
------------------------------------------------------------------------TGTGGTCACACACTCAGTCCGG-------------------------------------------------------------      0.243      0.530      0.113
-----------------------------------------------------------------------TTGTGGTCACACACTCAGTCCG--------------------------------------------------------------      1.603     13.828      3.125
-----------------------------------------------------------------------TTGTGGTCACACACTCAGTCC---------------------------------------------------------------      0.097      0.606      0.038
-----------------------------------------------------------------------TTGTGGTCACACACTCAGTCCGG-------------------------------------------------------------      5.440     57.322     16.077
----------------------------------------------------------------TTCGAGGTTGTGGTCACACACT---------------------------------------------------------------------      0.000      0.758      0.758
----------------------------------------------------------------TTCGAGGTTGTGGTCACACAC----------------------------------------------------------------------      0.000      0.379      0.113
----------------------------------------------------------GGCACCTTCGAGGTTGTGG------------------------------------------------------------------------------      0.000      0.038      0.038
```

```
--------------------------------------------------------TGGCACCTTCGAGGTTGTGGT------------------------------------------------------------------------------      0.000      0.341      0.113
--------------------------------------------------------TGGCACCTTCGAGGTTGTGG-------------------------------------------------------------------------------      0.389      5.418      0.377
------------------------------------------------------TATGGCACCTTCGAGGTTGTG--------------------------------------------------------------------------------      0.000      0.038      0.038
---------------------------------------------------GGATATGGCACCTTCGAGG-------------------------------------------------------------------------------------      0.049      0.152      0.113
--------------------------------------------------TGGATATGGCACCTTCGAGG-------------------------------------------------------------------------------------      0.389      6.365      1.694
--------------------------------------------------TGGATATGGCACCTTCGA---------------------------------------------------------------------------------------      0.000      0.038      0.075
--------------------------------------------------TGGATATGGCACCTTCGAGGT------------------------------------------------------------------------------------      0.097      0.152      0.152
----------------------------------------------TCCCTGGATATGGCACCTTCGAG--------------------------------------------------------------------------------------      0.000      0.038      0.038
----------------------------------------------TCCCTGGATATGGCACCTTCG----------------------------------------------------------------------------------------      0.291      0.985      0.339
----------------------------------------------TCCCTGGATATGGCACCTTCGA---------------------------------------------------------------------------------------      1.166      2.576      0.264
----------------------------------------TGGAGGTCCCTGGATATGGCA----------------------------------------------------------------------------------------------      0.097      0.682      0.075
----------------------------------------TGGAGGTCCCTGGATATGGCAC---------------------------------------------------------------------------------------------      0.000      0.189      0.113
----------------------------------------TGGAGGTCCCTGGATATGGC-----------------------------------------------------------------------------------------------      0.243      4.319      1.657
----------------------------------TCGACCTGGAGGTCCCTGGATA---------------------------------------------------------------------------------------------------      0.000      0.265      0.265
-----------------------------TGTGATCGACCTGGAGGTCC----------------------------------------------------------------------------------------------------------      0.437      2.766      0.678
-----------------------------TGTGATCGACCTGGAGGTCCC---------------------------------------------------------------------------------------------------------      0.049      1.137      0.226
-----------------------------TGTGATCGACCTGGAGGTCCCTG-------------------------------------------------------------------------------------------------------      0.000      0.076      0.076
-----------------------------TGTGATCGACCTGGAGGTC-----------------------------------------------------------------------------------------------------------      0.194      0.265      0.265
-----------------------------TGTGATCGACCTGGAGG-------------------------------------------------------------------------------------------------------------      0.049      1.250      0.113
----------------------------CTGTGATCGACCTGGAGGTCC----------------------------------------------------------------------------------------------------------      0.000      0.189      0.189
----------------------------CTGTGATCGACCTGGAGG-------------------------------------------------------------------------------------------------------------      0.000      0.076      0.076
------------------------CGGGCTGTGATCGACCTGGAGG-------------------------------------------------------------------------------------------------------------      0.194      1.212      0.038
------------------------CGGGCTGTGATCGACCTGGAG--------------------------------------------------------------------------------------------------------------      0.000      0.038      0.038
--------------TGGGGCCGGGCGGGCTGTGA-------------------------------------------------------------------------------------------------------------------------      0.097      0.076      0.075
--------------TGGGGCCGGGCGGGCTGTGAT------------------------------------------------------------------------------------------------------------------------      0.146      0.152      0.264
--------------TGGGGCCGGGCGGGCTGTGATC-----------------------------------------------------------------------------------------------------------------------      0.000      0.682      0.682
----TGTGCCGGGCTGGGGCCGGGC----------------------------------------------------------------------------------------------------------------------------------      0.486      6.819      2.033

ACGCTGTGCCGGGCTGGG                                                              A        C    G-                       A  C          -  ACA      CT     CC
                  GCCGGGCGGGCTGTGATCGACCTGGAGGTCCCTGGATATGGCACCTTCGAGGTTGTGGTCAC CACTCAGT CGGG  ACCTCGGCCCAGTTGCTGGTGCT TG TCTGACTACC CG   AGCAAG  TGGGG  C
                  CGGCCCGCCCGACACTAGCTGGACCTCCAGGGACCTATACCGTGGAAGCTCCAACACCAGTG GTGAGTCA GCCC  TGGAGCCGGGTCAGCGATCACGA AC AGACTGATGG GT   TCGTTC  ACCCC  /
------------------                                                              C        C   AA                        G  A          C  CC-      C-     CC

----------------------CCGCCCGACACTAGCTGGACCT---------------------------------------------------------------------------------------------------------------      0.146      0.758      0.151
---------------------------CGACACTAGCTGGACCT---------------------------------------------------------------------------------------------------------------      0.000      0.076      0.076
-----------------------CGCCCGACACTAGCTGGACCT---------------------------------------------------------------------------------------------------------------      1.263      2.500      0.489
------------------------GCCCGACACTAGCTGGACCT---------------------------------------------------------------------------------------------------------------      0.097      1.402      0.075
--------------------------CCGACACTAGCTGGACCTCCA------------------------------------------------------------------------------------------------------------      0.000      0.038      0.038
--------------------------------CTAGCTGGACCTCCAGGGACCT-----------------------------------------------------------------------------------------------------      0.000      0.114      0.114
--------------------------------------GGACCTCCAGGGACCTATACCGT----------------------------------------------------------------------------------------------      0.000      0.038      0.038
-----------------------------------------CCTCCAGGGACCTATACCGT----------------------------------------------------------------------------------------------      0.000      0.189      0.075
---------------------------------------GACCTCCAGGGACCTATACCGT----------------------------------------------------------------------------------------------      0.000      0.038      0.038
------------------------------------------------GGACCTATACCGTGGAAGCT---------------------------------------------------------------------------------------      0.000      0.189      0.189
-----------------------------------------------GGGACCTATACCGTGGAAGCT---------------------------------------------------------------------------------------     15.493      5.872      1.355
-----------------------------------------------------------GTGGAAGCTCCAACACCAGTGC--------------------------------------------------------------------------      0.000      0.114      0.114
--------------------------------------------------------------GAAGCTCCAACACCAGTGCGT------------------------------------------------------------------------      0.097      0.227      0.227
-------------------------------------------------------------GGAAGCTCCAACACCAGTGCGT------------------------------------------------------------------------      0.389      2.766      0.452
------------------------------------------------------------------CTCCAACACCAGTGCGTGAGT--------------------------------------------------------------------      0.194      0.606      0.226
-----------------------------------------------------------------GCTCCAACACCAGTGCGTGAGT--------------------------------------------------------------------      0.000      0.227      0.075
----------------------------------------------------------------AGCTCCAACACCAGTGCGTGAGT--------------------------------------------------------------------      0.000      0.038      0.038
-------------------------------------------------------------------TCCAACACCAGTGCGTGAGT--------------------------------------------------------------------      0.049      0.038      0.038
--------------------------------------------------------------------CCAACACCAGTGCGTGAGT--------------------------------------------------------------------      0.049      2.008      0.866
--------------------------------------------------------------------CCAACACCAGTGCGTGAGTCA------------------------------------------------------------------      0.000      0.038      0.038
--------------------------------------------------------------------CCAACACCAGTGCGTGAGTCAC-----------------------------------------------------------------      0.000      1.591      0.188
--------------------------------------------------------------------CCAACACCAGTGCGTGAGTCACG----------------------------------------------------------------      0.000      0.114      0.114
-----------------------------------------------------------------------------------GAGTCACGCCCAATGGAGCCGGGT------------------------------------------------      0.000      0.038      0.038
---------------------------------------------------------------------------------------CACGCCCAATGGAGCCGGGT------------------------------------------------      0.000      0.189      0.189
--------------------------------------------------------------------------------------TCACGCCCAATGGAGCCGGGT------------------------------------------------      0.000      0.303      0.075
-------------------------------------------------------------------------------------GTCACGCCCAATGGAGCCGGGT------------------------------------------------      0.049      0.114      0.113
------------------------------------------------------------------------------------AGTCACGCCCAATGGAGCCGGGT------------------------------------------------      0.000      0.076      0.075
--------------------------------------------------------------------------------------------CCAATGGAGCCGGGTCAGCGA------------------------------------------      0.000      0.038      0.151
---------------------------------------------------------------------------------------------CAATGGAGCCGGGTCAGCGA------------------------------------------      0.000      0.038      0.038
-------------------------------------------------------------------------------------------CCCAATGGAGCCGGGTCAGCGA------------------------------------------      0.000      0.038      0.038
-----------------------------------------------------------------------------------------CGCCCAATGGAGCCGGGTCAGCGAT-----------------------------------------      0.000      0.114      0.114
--------------------------------------------------------------------------------------------CCAATGGAGCCGGGTCAGCGAT-----------------------------------------      0.000      0.038      0.038
---------------------------------------------------------------------------------------------CAATGGAGCCGGGTCAGCGATCA---------------------------------------      0.000      0.227      0.227
---------------------------------------------------------------------------------------------------AGCCGGGTCAGCGATCA---------------------------------------      0.000      0.189      0.189
-----------------------------------------------------------------------------------------------ATGGAGCCGGGTCAGCGATCA---------------------------------------      0.000      0.076      0.076
--------------------------------------------------------------------------------------TCACGCCCAATGGAGCCGGGTCAGCGATCA---------------------------------------      0.000      0.758      0.188
---------------------------------------------------------------------------------------CACGCCCAATGGAGCCGGGTCAGCGATCA---------------------------------------      0.000      0.227      0.227
----------------------------------------------------------------------------------------ACGCCCAATGGAGCCGGGTCAGCGATCA---------------------------------------      0.000      0.114      0.114
----------------------------------------------------------------------------------------------AATGGAGCCGGGTCAGCGATCA---------------------------------------      0.000      0.076      0.076
-----------------------------------------------------------------------------------------------------------CAGCGATCACGAGACAAGA-----------------------------      0.049      0.265      0.113
-------------------------------------------------------------------------------------------------------------GCGATCACGAGACAAGA-----------------------------      0.000      0.076      0.076
-----------------------------------------------------------------------------------------------------------CAGCGATCACGAGACAAGAC----------------------------      0.000      0.076      0.076
-----------------------------------------------------------------------------------------------------------CAGCGATCACGAGACAAGACT---------------------------     12.773     25.687      7.041
------------------------------------------------------------------------------------------------------------AGCGATCACGAGACAAGACT---------------------------      0.000      0.114      0.114
----------------------------------------------------------------------------------------------------------TCAGCGATCACGAGACAAGACT---------------------------      1.554      0.227      0.264
-------------------------------------------------------------------------------------------------------------GCGATCACGAGACAAGACT---------------------------      0.097      0.455      0.455
-------------------------------------------------------------------------------------------------------------GCGATCACGAGACAAGACTG--------------------------      0.000      0.189      0.189
--------------------------------------------------------------------------------------------------------------CGATCACGAGACAAGACTG--------------------------      0.000      0.189      0.264
--------------------------------------------------------------------------------------------------------------CGATCACGAGACAAGACTGA-------------------------      0.097      2.917      2.297
-------------------------------------------------------------------------------------------------------------GCGATCACGAGACAAGACTGA-------------------------      0.049      0.568      0.568
-----------------------------------------------------------------------------------------------------------------TCACGAGACAAGACTGAT------------------------      0.000      1.174      0.264
------------------------------------------------------------------------------------------------------------AGCGATCACGAGACAAGACTGAT------------------------      0.000      0.341      0.341
-----------------------------------------------------------------------------------------------------------CAGCGATCACGAGACAAGACTGAT------------------------      0.000      0.758      0.038
-------------------------------------------------------------------------------------------------------------GCGATCACGAGACAAGACTGAT------------------------      8.791    114.227      3.012
---------------------------------------------------------------------------------------------------------------GATCACGAGACAAGACTGAT------------------------      0.049      0.114      0.226
------------------------------------------------------------------------------------------------------------------CACGAGACAAGACTGAT------------------------      0.000      0.227      0.038
--------------------------------------------------------------------------------------------------------------CGATCACGAGACAAGACTGAT------------------------      9.714    439.554    136.484 miR_t28
-----------------------------------------------------------------------------------------------------------------TCACGAGACAAGACTGATGGC---------------------      0.000      0.076      0.076
-----------------------------------------------------------------------------------------------------------------TCACGAGACAAGACTGATGGCGT-------------------      0.000      0.076      0.038
```

```
miRNA: miR_t30 chromosome_4 Start: 457804 End: 457980 Condition: HS+N
----TGGACTGGGGTACAGGCTTGGT---------------------------------------------------------------------------------      0.000      0.038      0.038
---------------------TTGGTACCGGGCATGCA---------------------------------------------------------------------      0.000      0.038      0.038
---------------------TTGGTACCGGGCATGCAGGGGG----------------------------------------------------------------      0.243      0.152      0.113
----------------------TGGTACCGGGCATGCAGGGG-----------------------------------------------------------------      0.000      0.038      0.038
----------------------TGGTACCGGGCATGCAGGGGGT---------------------------------------------------------------      0.486      0.493      0.226
----------------------TGGTACCGGGCATGCAGGGGG----------------------------------------------------------------      1.020      2.008      2.372
-----------------------GGTACCGGGCATGCAGGGGG----------------------------------------------------------------      0.000      0.038      0.038
-------------------------TACCGGGCATGCAGGGGGTG--------------------------------------------------------------      0.000      0.038      0.038
-------------------------TACCGGGCATGCAGGGGGTGG-------------------------------------------------------------      0.049      0.038      0.038
-------------------------------------------TGGGTAGTGTGGTGG-AGGTAGC-----------------------------------------     14.959     11.934      7.304
-------------------------------------------TGGGTAGTGTGGTGG-AGGTA-------------------------------------------      0.437      0.152      0.715
-------------------------------------------TGGGTAGTGTGGTGG-AGGT--------------------------------------------      0.000      0.113      0.113
-------------------------------------------TGGGTAGTGTGGTGG-AGGTAG------------------------------------------    110.541     11.404     24.661
-------------------------------------------TGGGTAGTGTGGTGG-AGG---------------------------------------------      0.049      0.038      0.038
--------------------------------------------GGGTAGTGTGGTGG-AGGTAGC-----------------------------------------      0.291      0.076      0.151
--------------------------------------------GGGTAGTGTGGTGG-AGGTAG------------------------------------------      3.254      0.227      0.791
-----------------------------------------------TAGTGTGGTGG-AGGTAGC-----------------------------------------      0.583      1.553      6.551
-----------------------------------------------TAGTGTGGTGG-AGGTAGCAGG--------------------------------------    560.477    116.575    417.886 miR_t30
-----------------------------------------------TAGTGTGGTGG-AGGTAG------------------------------------------      0.291      0.152      0.339
-----------------------------------------------TAGTGTGGTGG-AGGTAGCAG---------------------------------------     10.879      8.221     60.279
-----------------------------------------------TAGTGTGGTGG-AGGTAGCA----------------------------------------      1.068      1.326      7.944
-----------------------------------------------TAGTGTGGTGG-AGGTAGCAGGCAGC-----------------------------------      0.000      0.075      0.075
-----------------------------------------------TAGTGTGGTGG-AGGTAGCAGGC--------------------------------------      4.323      8.562     13.856
------------------------------------------------AGTGTGGTGG-AGGTAGCAG----------------------------------------      0.194      0.527      0.527
------------------------------------------------AGTGTGGTGG-AGGTAGCAGGC--------------------------------------      0.097      0.151      0.151
------------------------------------------------AGTGTGGTGG-AGGTAGCA-----------------------------------------      0.000      0.038      0.038
------------------------------------------------AGTGTGGTGG-AGGTAGCAGG---------------------------------------      4.371      0.303      2.598
------------------------------------------------AGTGTGGTGG-AGGTAGC------------------------------------------      0.000      0.038      0.038
-------------------------------------------------GTGTGGTGG-AGGTAGCAG----------------------------------------      0.000      0.038      0.038
-------------------------------------------------GTGTGGTGG-AGGTAGCAGG---------------------------------------      0.631      0.151      0.151
--------------------------------------------------TGTGGTGG-AGGTAGCAGG---------------------------------------      0.194      0.113      0.113
--------------------------------------------------TGTGGTGG-AGGTAGCAGGCAG------------------------------------      0.923      1.553      6.137
--------------------------------------------------TGTGGTGG-AGGTAGCAGGCA-------------------------------------      1.554      0.265      0.678
--------------------------------------------------TGTGGTGG-AGGTAGCAGGCAGC-----------------------------------      0.534      0.455      1.883
--------------------------------------------------TGTGGTGG-AGGTAGCAGGC--------------------------------------      0.000      0.151      0.151
--------------------------------------------------TGTGGTGG-AGGTAGCAG----------------------------------------      0.000      0.075      0.075
----------------------------------------------------TGGTGG-AGGTAGCAGGCAG------------------------------------      0.049      0.038      0.075
----------------------------------------------------TGGTGG-AGGTAGCAGGCAGC-----------------------------------      0.971      0.455      4.142
----------------------------------------------------TGGTGG-AGGTAGCAGGCAGCG----------------------------------      0.000      0.038      0.113
-----------------------------------------------------GGTGG-AGGTAGCAGGCAGC-----------------------------------      0.000      0.038      0.038
-------------------------------------------------------TGG-AGGTAGCAGGCAGCGCGG-------------------------------      0.000      0.038      0.038
-------------------------------------------------------TGG-AGGTAGCAGGCAGCGCGGG------------------------------      0.000      0.188      0.188

GCGTTGGACTGGGGTACAGGCTTGGTACCGGGCATGC                G    -                       G     T   T   A      A G
                                     AGGGGGTGGGTAGTGT GTGG AGGTAGCAGGCAGCGCGGGGCTG TGCTG TGG GGA GAGGAC G C
                                     TCCTCCACCCATCACA CGCC TCCGTCGTCCGTCGCGCCCCGAC ACGAC ACC CCT CTCCTG C C
-------------------------------------                A    G                       G     C   T   C      C C

----------------------------------------------CATCACAACGCCGTCCGTCGT----------------------------------------      0.000      0.038      0.038
-----------------------------------------CCACCCATCACAACGCCGTCCGT-------------------------------------------      0.000      0.151      0.151
```

```
miRNA: miR_t34,miR_t32 chromosome_7 Start: 2591474 End: 2591850 Condition: HS+N
---------------------------------------------------------------------------------------------------------------------------TGCATGGCTCGCTGTCAGAGGC-------------------------------------------------------      0.243      1.061      0.038
---------------------------------------------------------------------------------------------------------------------------TGCATGGCTCGCTGTCAGAGGCTTCCAGC------------------------------------------------      0.049      1.061      0.602
---------------------------------------------------------------------------------------------------TGGGCTTTCAGGCTTTCAGC---------------------------------------------------------------------------------      0.000      0.038      0.038
---------------------------------------------------------------------------------------------------TGGGCTTTCAGGCTTTCAGCTTCC-----------------------------------------------------------------------------      0.243      1.061      0.113
----------------------------------------------------------------------------------------------GCAGGTGGGCTTTCAGGCTTTC------------------------------------------------------------------------------------      0.000      0.038      0.038
---------------------------------------------------------------------------------------------TGCAGGTGGGCTTTCAGGCTTTC------------------------------------------------------------------------------------      0.097      0.833      0.489
-------------------------------------------------------------------------------GCCCCAGTCGCCGCTGCAG------------------------------------------------------------------------------------------------------      0.097      0.151      0.151
----------------------------------------------------------------TTGCAAACAGGGTCGGCCCCAG------------------------------------------------------------------------------------------------------------------      0.000      0.038      0.038
---------------------------------------------------------------TTTGCAAACAGGGTCGGCCCC--------------------------------------------------------------------------------------------------------------------      0.243      0.114      0.264
-------------------------------------------------------CGGCGAGCTTTGCAAACAGGG----------------------------------------------------------------------------------------------------------------------------      0.340      0.265      0.264
------------------------------------------------------TCGGCGAGCTTTGCAAACAGG-----------------------------------------------------------------------------------------------------------------------------     10.685      8.827      8.886
------------------------------------------------------TCGGCGAGCTTTGCAAACAG------------------------------------------------------------------------------------------------------------------------------      1.068      4.054      7.229
------------------------------------------------------TCGGCGAGCTTTGCAAACAGGG----------------------------------------------------------------------------------------------------------------------------      0.340      0.682      0.188
-----------------------------------------------------CTCGGCGAGCTTTGCAAACAG------------------------------------------------------------------------------------------------------------------------------      0.000      0.038      0.038
----------------------------------------------------TCTCGGCGAGCTTTGCAAA---------------------------------------------------------------------------------------------------------------------------------      0.000      0.227      0.038
----------------------------------------------------TCTCGGCGAGCTTTGCAAAC--------------------------------------------------------------------------------------------------------------------------------      0.534      1.023      0.904
----------------------------------------------------TCTCGGCGAGCTTTGCAAACA-------------------------------------------------------------------------------------------------------------------------------      2.234      0.796      1.242
----------------------------------------------------TCTCGGCGAGCTTTGCAAACAG------------------------------------------------------------------------------------------------------------------------------      1.603      3.902      3.577
----------------------------------------------------TCTCGGCGAGCTTTGCAAACAGG-----------------------------------------------------------------------------------------------------------------------------      1.943      2.008      0.075
---------------------------------------------------CTCTCGGCGAGCTTTGCAAAC--------------------------------------------------------------------------------------------------------------------------------      0.000      0.038      0.038
```

```
------------------------------------------TCAATGTCCCTCTCGGCGAGC------------------------------------------------------------------------------------------------------------------------------------------      0.874      1.553      1.619
--------------------------------------CGCATCAATGTCCCTCTCGGC----------------------------------------------------------------------------------------------------------------------------------------------      0.000      0.226      0.226
---------------------TACCCCGCTTGGTGCAGCGC----------------------------------------------------------------------------------------------------------------------------------------------------------------      0.000      0.114      0.038
--------------------ATACCCCGCTTGGTGCAGC------------------------------------------------------------------------------------------------------------------------------------------------------------------      0.000      0.038      0.038
--------------------ATACCCCGCTTGGTGCAGCGC----------------------------------------------------------------------------------------------------------------------------------------------------------------      0.000      0.075      0.075
------------------TCATACCCCGCTTGGTGCAG-------------------------------------------------------------------------------------------------------------------------------------------------------------------      0.583      0.152      0.264
------------------TCATACCCCGCTTGGTGCAGC------------------------------------------------------------------------------------------------------------------------------------------------------------------      0.194      0.644      0.452
------------TCCCCGTCATACCCCGCTTGG------------------------------------------------------------------------------------------------------------------------------------------------------------------------      3.303      0.455      0.301

------C  --                              A             C               C               CG         G        CAG                             AGAGGCTTCCA       C      A     TGA      AC       GA  T--  AT
       GT  ATCCCCGTCATACCCCGCTTGGTGCAGCGC TCAATGTCCCTCT GGCGAGCTTTGCAAA AGGGTCGGCCCCAGT  CCGCTGCAG TGGGCTTT   GCTTTCAGCTTCCTGCATGGCTCGCTGTC           GCGCCTT CTTGCC CCCGA   TACGCG  TCGGATT  GT   CG  T
       CG  TAGGGGCAGTATGGGGCGAACCACGTCGCG AGTTATAGGGAGA CCGCTCGAAGCGTTT TCTCAGCCGGGGTCA  GGCGACGTC ACCCGAAG   CGAAGGTCGAAGGACGTGCCGAGCGGCAG           CGCGGAA GAACGG GGGCT   ATGCGT  AGCCTAA  CA   GC  /
GTCGCCT  TC                              C             A               A               AA         G        CA-                             GCT--------       C      C     ---      CA       G-  TAT  GG

----CCTCGTCTAGGGGCAGTAT----------------------------------------------------------------------------------------------------------------------------------------------------------------------------------      0.049      0.114      0.075
----------------GCAGTATGGGGCGAACCACGT--------------------------------------------------------------------------------------------------------------------------------------------------------------------      4.905      6.479      2.523
-----------------------GGGGCGAACCACGTCGCGCAGT------------------------------------------------------------------------------------------------------------------------------------------------------------      0.049      0.076      0.038
-------------------------GGCGAACCACGTCGCGCAGTT-----------------------------------------------------------------------------------------------------------------------------------------------------------      0.437      1.212      0.339
---------------------------CGAACCACGTCGCGCAGTT-----------------------------------------------------------------------------------------------------------------------------------------------------------      0.000      0.417      0.377
---------------------------CGAACCACGTCGCGCAGTTA----------------------------------------------------------------------------------------------------------------------------------------------------------      0.000      0.114      0.151
-------------------------GGCGAACCACGTCGCGCAGTTAT---------------------------------------------------------------------------------------------------------------------------------------------------------      0.000      0.038      0.038
---------------------------CGAACCACGTCGCGCAGTTAT---------------------------------------------------------------------------------------------------------------------------------------------------------      3.885     32.696     23.042 miR_t34
-------------------------------CCACGTCGCGCAGTTATAGGG-----------------------------------------------------------------------------------------------------------------------------------------------------      0.000      0.038      0.075
-------------------------------CCACGTCGCGCAGTTATAGGGAG---------------------------------------------------------------------------------------------------------------------------------------------------      0.000      0.075      0.075
-----------------------------------GTCGCGCAGTTATAGGGAGAA-------------------------------------------------------------------------------------------------------------------------------------------------      0.243      0.152      0.113
---------------------------------------TCGCGCAGTTATAGGGAGAA----------------------------------------------------------------------------------------------------------------------------------------------      0.000      0.038      0.038
----------------------------------CGTCGCGCAGTTATAGGGAGAA-------------------------------------------------------------------------------------------------------------------------------------------------      0.291      0.796      0.414
------------------------------------------------AGGGAGAACCGCTCGAAGCGT------------------------------------------------------------------------------------------------------------------------------------      0.097      0.379      0.113
-------------------------------------------------GGGAGAACCGCTCGAAGCGTT-----------------------------------------------------------------------------------------------------------------------------------      0.049      0.114      0.038
--------------------------------------------------GGAGAACCGCTCGAAGCGTTT----------------------------------------------------------------------------------------------------------------------------------      0.340      0.189      0.038
-----------------------------------------------------------------GCGTTTATCTCAGCCGGGG---------------------------------------------------------------------------------------------------------------------      0.097      0.151      0.151
-------------------------------------------------------------CGAAGCGTTTATCTCAGCCGGGGT--------------------------------------------------------------------------------------------------------------------      0.777      0.833      0.038
------------------------------------------------------------------CGTTTATCTCAGCCGGGGT--------------------------------------------------------------------------------------------------------------------      0.049      0.038      0.038
-------------------------------------------------------------------GTTTATCTCAGCCGGGGT--------------------------------------------------------------------------------------------------------------------      0.000      0.075      0.075
----------------------------------------------------------------AGCGTTTATCTCAGCCGGGGT--------------------------------------------------------------------------------------------------------------------      0.631      0.038      0.075
-----------------------------------------------------------------GCGTTTATCTCAGCCGGGGT--------------------------------------------------------------------------------------------------------------------      6.945      1.288      1.167
--------------------------------------------------------------GAAGCGTTTATCTCAGCCGGGGT--------------------------------------------------------------------------------------------------------------------      2.526      1.137      0.979
-----------------------------------------------------------------------------GCCGGGGTCAAAGGCGACG-----------------------------------------------------------------------------------------------------      0.000      0.038      0.038
----------------------------------------------------------------------------AGCCGGGGTCAAAGGCGACG-----------------------------------------------------------------------------------------------------      0.000      0.075      0.075
-----------------------------------------------------------------------------GCCGGGGTCAAAGGCGACGT----------------------------------------------------------------------------------------------------      0.291      1.326      0.904
---------------------------------------------------------------------------CAGCCGGGGTCAAAGGCGACGT----------------------------------------------------------------------------------------------------      0.243      0.985      2.598
-------------------------------------------------------------------------CTCAGCCGGGGTCAAAGGCGACGT----------------------------------------------------------------------------------------------------      0.000      0.038      0.038
----------------------------------------------------------------------------AGCCGGGGTCAAAGGCGACGT----------------------------------------------------------------------------------------------------      0.729      1.819      1.883
------------------------------------------------------------------------TCTCAGCCGGGGTCAAAGGCGACGT----------------------------------------------------------------------------------------------------      0.000      0.038      0.377
--------------------------------------------------------------------------TCAGCCGGGGTCAAAGGCGACGT----------------------------------------------------------------------------------------------------      0.000      0.075      0.075
----------------------------------------------------------------------------------------------CGTCGACCCGAAGCA-CGAAGGT------------------------------------------------------------------------------------      0.049      0.076      0.038
-----------------------------------------------------------------------------------------------GTCGACCCGAAGCA-CGAAGGT------------------------------------------------------------------------------------      0.049      0.038      0.038
---------------------------------------------------------------------------------------------------------AGCA-CGAAGGTCGAAGGACGT--------------------------------------------------------------------------      0.777      1.364      0.715
----------------------------------------------------------------------------------------------------------GCA-CGAAGGTCGAAGGACGT--------------------------------------------------------------------------      0.194      0.076      0.151
----------------------------------------------------------------------------------------------------------------------------CGTGCCGAGCGGCAGGC------------------------------------------------------------      0.000      0.038      0.038
--------------------------------------------------------------------------------------------------------------------------GACGTGCCGAGCGGCAGGCT-----------------------------------------------------------      0.243      0.682      0.038
-------------------------------------------------------------------------------------------------------------------------GGACGTGCCGAGCGGCAGGCT-----------------------------------------------------------      0.777      7.994      7.380
---------------------------------------------------------------------------------------------------------------------------ACGTGCCGAGCGGCAGGCT-----------------------------------------------------------      0.000      0.264      0.264
----------------------------------------------------------------------------------------------------------------------------CGTGCCGAGCGGCAGGCT-----------------------------------------------------------      0.097      0.606      0.151
------------------------------------------------------------------------------------------------------------------------------------------------------CGCGGAACGAACGGCGGG---------------------------------      0.000      0.038      0.038
---------------------------------------------------------------------------------------------------------------------------------------------T--------CGCGGAACGAACGGCGGGCT-------------------------------      0.243      0.038      0.113
------------------------------------------------------------------------------------------------------------------------------------------------------CGCGGAACGAACGGCGGGCT-------------------------------      0.146      0.189      0.339
------------------------------------------------------------------------------------------------------------------------------------------------------CGCGGAACGAACGGCGGGCT---A---------------------------      0.049      0.265      0.414
-------------------------------------------------------------------------------------------------------------------------------------------------------GCGGAACGAACGGCGGGCT---A---------------------------      0.000      0.188      0.188
----------------------------------------------------------------------------------------------------------------------------------------------------------GAACGAACGGCGGGCT---AT--------------------------      0.000      0.568      0.339
-------------------------------------------------------------------------------------------------------------------------------------------------------GCGGAACGAACGGCGGGCT---AT--------------------------      9.762     19.436     39.684
---------------------------------------------------------------------------------------------------------------------------------------------------------GGAACGAACGGCGGGCT---AT--------------------------      0.097      1.288      1.845
--------------------------------------------------------------------------------------------------------------------------------------------------------CGGAACGAACGGCGGGCT---AT--------------------------      0.097      2.955      4.518
------------------------------------------------------------------------------------------------------------------------------------------------------CGCGGAACGAACGGCGGGCT---AT--------------------------      9.374     94.564     53.276 miR_t32
---------------------------------------------------------------------------------------------------------------------------------------------T--------CGCGGAACGAACGGCGGGCT---AT--------------------------      1.117      7.388      2.033

miRNA: miR_t33,miR_t96 chromosome_17 Start: 1972737 End: 1972997 Condition: HS+N
--------------------------------------------------------------------------------------------------------------CTCCCCCGCAG-CAAGCAC-----      0.000      0.038      0.038
-------------------------------------------------------------------------------------------------------TCCGCGACTCCCCCGCAG-CAAG--------      0.000      0.075      0.075
-------------------------------------------------------------------------------------------------------TCCGCGACTCCCCCGCAG-CAAGCAC-----      0.097      0.871      0.339
-------------------------------------------------------------------------------------------------------TCCGCGACTCCCCCGCAG-CAAGC-------      0.146      0.189      0.489
-------------------------------------------------------------------------------------------------------TCCGCGACTCCCCCGCAG-CAAGCA------      0.097      0.038      0.038
-------------------------------------------------------------------------------TGCCCGGGCCCGCAACGCGC-----------------------------------      0.049      0.189      0.075
----------------------------------------------------------TGACTGACA------GCAGGGTGCCC--------------------------------------------------      0.097      0.341      0.188
----------------------------------------------------------TGACTGACA------GCAGGGTGCCCG-------------------------------------------------      0.243      0.152      0.075
---------------------------------------------------------ATGACTGACA------GCAGGGTGC----------------------------------------------------      0.000      0.226      0.226
---------------------------------------------------------ATGACTGACA------GCAGGGTGCC---------------------------------------------------      0.243      1.478      0.791
--------------------------------------------------------TATGACTGACA------GCAGGGTGCC---------------------------------------------------     31.764    316.652    121.047 miR_t33
--------------------------------------------------------TATGACTGACA------GCAGGGTGC----------------------------------------------------      0.631     31.521     14.721
--------------------------------------------------------TATGACTGACA------GCAGGGT------------------------------------------------------      0.000      0.379      0.264
--------------------------------------------------------TATGACTGACA------GCAGGGTGCCC--------------------------------------------------      0.194      1.894      0.301
--------------------------------------------------------TATGACTGACA------GCAGGGTG-----------------------------------------------------      0.000      0.985      0.715
-------------------------------------------------------CTATGACTGACA------GCAGGGTGCC---------------------------------------------------      0.000      0.227      0.188
-----------------------------TCACCAGCCACGCAGAAGCA-------------------------------------------------------------------------------------      0.049      0.038      0.075
-----------------------------TCACCAGCCACGCAGAAGCAT------------------------------------------------------------------------------------      0.631      0.038      0.075
----------------------------TTCACCAGCCACGCAGAAGCA-------------------------------------------------------------------------------------      0.874      0.265      0.452
----------------------------TTCACCAGCCACGCAGAAG---------------------------------------------------------------------------------------      0.049      0.038      0.038
----------------------------TTCACCAGCCACGCAGAAGC--------------------------------------------------------------------------------------      0.194      0.530      0.489
-------------------------ACAAATTCACCAGCCACGCAG----------------------------------------------------------------------------------------      0.097      0.189      0.188
------------------------TACAAATTCACCAGCCAC--------------------------------------------------------------------------------------------      0.000      0.038      0.038
------------------------TACAAATTCACCAGCCACGC------------------------------------------------------------------------------------------      0.631      0.227      0.678
------------TCCGCTCTACTACAAATTCACC----------------------------------------------------------------------------------------------------      0.049      0.038      0.188
-------TTGTCTCCGCTCTACTACAAA----------------------------------------------------------------------------------------------------------      0.049      0.038      0.038

---CTTTT         T                                                 ------                       C                      C  - AA       G
        TGTCTCCGC CTACTACAAATTCACCAGCCACGCAGAAGCATGCCTGCTATGACTGACA      GCAGGGTGCCCGGGCCCGCAACG GCAGCGCTCCGCGACTCCCCCG AG C  GCACCAC C
        ACAGAGGCG GATGATGTTTAAGTGGTCGGTGCGTCTTCGTACGGACGATACTGACTGT      CGTTCCACGGGCTTGGGCGTTGC CGTCGCGAGGCGCTGAGGGGGC TC G  CGTGGTG C
CCGGTAAT         T                                                 GACTGT                       C                      A  T GC       C

----TAATACAGAGGCGTGATGAT--------------------------------------------------------------------------------------------------------------      0.000      0.114      0.038
----TAATACAGAGGCGTGATGATGT------------------------------------------------------------------------------------------------------------      0.000      0.075      0.075
-----AATACAGAGGCGTGATGATGT------------------------------------------------------------------------------------------------------------      0.146      0.076      0.151
--------ACAGAGGCGTGATGATGTT-----------------------------------------------------------------------------------------------------------      0.000      0.038      0.038
--------ACAGAGGCGTGATGATGTTT----------------------------------------------------------------------------------------------------------      0.389      1.137      1.845
-------TACAGAGGCGTGATGATGTTT----------------------------------------------------------------------------------------------------------      0.826      0.455      1.355
------------AGGCGTGATGATGTTTAAG-------------------------------------------------------------------------------------------------------      0.049      0.038      0.038
-----------GAGGCGTGATGATGTTTAAG-------------------------------------------------------------------------------------------------------      0.291      0.341      0.715
--------------GCGTGATGATGTTTAAGT------------------------------------------------------------------------------------------------------      0.049      0.189      0.151
------------AGGCGTGATGATGTTTAAGT------------------------------------------------------------------------------------------------------      4.468      3.523      5.083
-------------GGCGTGATGATGTTTAAGT------------------------------------------------------------------------------------------------------      9.665     11.139      6.815
-----------GAGGCGTGATGATGTTTAAGT------------------------------------------------------------------------------------------------------     43.274     18.072     22.327
---------------CGTGATGATGTTTAAGT------------------------------------------------------------------------------------------------------      0.000      0.303      0.339
----------AGAGGCGTGATGATGTTTAAGT------------------------------------------------------------------------------------------------------     16.659      6.668      4.104
-------------GGCGTGATGATGTTTAAGTGG----------------------------------------------------------------------------------------------------      0.000      0.038      0.188
---------------CGTGATGATGTTTAAGTGGT---------------------------------------------------------------------------------------------------      7.188      6.554      2.937
--------------GCGTGATGATGTTTAAGTGGT---------------------------------------------------------------------------------------------------      6.265      3.789      0.979
-------------GGCGTGATGATGTTTAAGTGGT---------------------------------------------------------------------------------------------------      6.071      7.160      2.899
------------------GATGATGTTTAAGTGGTC--------------------------------------------------------------------------------------------------      0.000      0.152      0.151
------------------GATGATGTTTAAGTGGTCGG------------------------------------------------------------------------------------------------      4.080      2.690      1.092
--------------------TGATGTTTAAGTGGTCGG------------------------------------------------------------------------------------------------      0.097      0.227      0.075
-----------------TGATGATGTTTAAGTGGTCGG------------------------------------------------------------------------------------------------      0.000      0.227      0.075
-------------------ATGATGTTTAAGTGGTCGGT-----------------------------------------------------------------------------------------------      2.428      4.471      2.259
--------------------TGATGTTTAAGTGGTCGGT-----------------------------------------------------------------------------------------------      2.768     16.064      5.685
---------------------GATGTTTAAGTGGTCGGT-----------------------------------------------------------------------------------------------      0.437      3.145      0.866
-----------------TGATGATGTTTAAGTGGTCGGT-----------------------------------------------------------------------------------------------     12.191     16.329      2.560
------------------GATGATGTTTAAGTGGTCGGT-----------------------------------------------------------------------------------------------    351.391    389.393    162.764 miR_t96
------------------GATGATGTTTAAGTGGTCGGTG----------------------------------------------------------------------------------------------      0.000      0.038      0.075
----------------------ATGTTTAAGTGGTCGGTGCGT-------------------------------------------------------------------------------------------      0.971      0.227      0.791
---------------------GATGTTTAAGTGGTCGGTGCGT-------------------------------------------------------------------------------------------      4.371      3.296      2.673
------------------------GTTTAAGTGGTCGGTGCGT-------------------------------------------------------------------------------------------      0.049      0.076      0.188
-----------------------TGTTTAAGTGGTCGGTGCGT-------------------------------------------------------------------------------------------      3.788      3.410      4.480
------------------------GTTTAAGTGGTCGGTGCGTCT-----------------------------------------------------------------------------------------      0.534      0.076      0.527
-------------------------TTTAAGTGGTCGGTGCGTCTT----------------------------------------------------------------------------------------      0.000      0.076      0.151
------------------------GTTTAAGTGGTCGGTGCGTCTT----------------------------------------------------------------------------------------      0.146      0.114      0.151
--------------------------TTAAGTGGTCGGTGCGTCTT----------------------------------------------------------------------------------------      0.000      0.227      0.075
------------------------------GTGGTCGGTGCGTCTTCGT-------------------------------------------------------------------------------------      0.049      0.038      0.038
----------------------------AAGTGGTCGGTGCGTCTTCGT-------------------------------------------------------------------------------------      0.243      0.114      0.151
-----------------------------AGTGGTCGGTGCGTCTTCGT-------------------------------------------------------------------------------------      0.243      0.189      0.075
----------------------------------------CGTCTTCGTACGGACGATACT-------------------------------------------------------------------------      0.000      0.189      0.075
---------------------------------------------TCGTACGGACGATACTGACT---------------------------------------------------------------------      0.000      0.038      0.038
-------------------------------------------------------------------------CGTTCCACGGGCTTGGGCGT-----------------------------------------      0.000      0.075      0.075
-------------------------------------------------------------------------CGTTCCACGGGCTTGGGCGTT----------------------------------------      0.971      0.833      1.205
------------------------------------------------------------------------------------------CGTTGCCCGTCGCGAGGCGCT------------------------      0.194      0.114      0.113
----------------------------------------------------------------------------------------------------CGCGAGGCGCTGAGGGGGCAT-------------      0.583      0.379      1.393
------------------------------------------------------------------------------------------------------CGAGGCGCTGAGGGGGCATCT-----------      0.389      0.114      0.264
------------------------------------------------------------------------------------------------------------GCTGAGGGGGCATCTGGCCGT------      6.557      1.174      2.334
------------------------------------------------------------------------------------------------------CGAGGCGCTGAGGGGGCATCTGGCCGT------      1.020      5.607      0.151
--------------------------------------------------------------------------------------------------------AGGCGCTGAGGGGGCATCTGGCCGT------      0.243      0.076      0.188
-------------------------------------------------------------------------------------------------------GAGGCGCTGAGGGGGCATCTGGCCGT------      0.097      0.038      0.038
-------------------------------------------------------------------------------------------------------------CTGAGGGGGCATCTGGCCGT------      0.049      0.076      0.075
-----------------------------------------------------------------------------------------------------------CGCTGAGGGGGCATCTGGCCGT------      0.291      1.478      1.318
--------------------------------------------------------------------------------------------------------------TGAGGGGGCATCTGGCCGTGGT---      0.097      0.152      0.113
---------------------------------------------------------------------------------------------------------------GAGGGGGCATCTGGCCGTGGT---      0.097      0.265      0.188

miRNA: miR_t37,miR_t39 chromosome_10 Start: 6282475 End: 6282576 Condition: HS+N
-------CGGTCATCAATTGCTCTTTCTG-----------------------      0.291      0.189      0.038
----------TCATCAATTGCTCTTTCTG-----------------------      0.000      0.075      0.075
----------TCATCAATTGCTCTTTCTGATG--------------------      0.826      0.113      0.113
-------------TCAATTGCTCTTTCTGATGA-------------------     15.785      3.675      1.054
-------------TCAATTGCTCTTTCTGAT---------------------      0.146      0.417      0.038
-------------TCAATTGCTCTTTCTGATGAG------------------      1.408      0.076      0.075
-------------TCAATTGCTCTTTCTGATG--------------------    153.718    272.136     90.324 miR_t37
-------------TCAATTGCTCTTTCTGA----------------------      0.146      0.189      0.075
--------------CAATTGCTCTTTCTGATG--------------------      3.497      2.766      1.205
---------------AATTGCTCTTTCTGATG--------------------      1.166      0.909      0.565
------------------------TTCTGATGAGCGATGCCGTGG-------      0.000      0.227      0.038
```

```
--G   -                 T                       CCG
   CTC CGGTCATCAATTGCTCT TCTGATGAGCGATGCCGTGGCCT   C
   GAG GCCAGTAGTTAACGAGA AGACTACTCGCTACGGCACCGGA   C
GCG   T                 T                       CCC

-------------AGTTAACGAGATAGACTACT-------------------      0.146      0.152      0.038
-----------GTAGTTAACGAGATAGACT----------------------     14.570    100.663     57.756 miR_t39
--------CCAGTAGTTAACGAGATAGACT----------------------      0.826      3.031      0.301
-------------AGTTAACGAGATAGACT----------------------      0.000      0.265      0.075
----AGTGCCAGTAGTTAACGAGATAGACT----------------------      0.000      0.038      0.038
----------AGTAGTTAACGAGATAGACT----------------------      4.128      2.993      2.824
---------CAGTAGTTAACGAGATAGACT----------------------     10.394      2.955      2.033
-----------GTAGTTAACGAGATAGAC-----------------------      0.049      0.493      0.339
---------CAGTAGTTAACGAGATAGA------------------------      0.049      0.114      0.151
-----------GTAGTTAACGAGATAGA------------------------      0.097      7.805      2.749
----------AGTAGTTAACGAGATAGA------------------------      0.000      0.189      0.113
-----GTGCCAGTAGTTAACGAGATAG-------------------------      0.243      0.076      0.075
-----GTGCCAGTAGTTAACGAGAT---------------------------      0.340      0.038      0.038
----AGTGCCAGTAGTTAACGAGAT---------------------------      0.049      0.114      0.075

miRNA: miR_t79,miR_t43 chromosome_2 Start: 3047224 End: 3047445 Condition: HS+N
----------------------------------------------TACTGGAGGCGCCGCAGCGC--------------------------------------------------      0.049      0.189      0.113
----------------------------------------------TACTGGAGGCGCCGCAGCGCA-------------------------------------------------      0.000      0.152      0.075
----------------------------------------TGGGCCTACTGGAGGCG-----------------------------------------------------------      0.146      0.076      0.038
----------------------------------------TGGGCCTACTGGAGGCGC----------------------------------------------------------      0.389      0.038      0.075
----------------------------------------TGGGCCTACTGGAGGCGCCGC-------------------------------------------------------      2.914      0.075      0.075
----------------------------------------TGGGCCTACTGGAGGCGCCG--------------------------------------------------------      0.097      0.113      0.113
--------------------------------------TGTGGGCCTACTGGAGGC------------------------------------------------------------      0.340      0.493      0.866
--------------------------------------TGTGGGCCTACTGGAGGCG-----------------------------------------------------------      3.351      1.288      0.791
--------------------------------------TGTGGGCCTACTGGAGGCGC----------------------------------------------------------      3.448      2.463      1.958
--------------------------------------TGTGGGCCTACTGGAGG-------------------------------------------------------------      1.651      0.189      0.339
--------------------------------------TGTGGGCCTACTGGAGGCGCC---------------------------------------------------------      3.497      0.379      0.226
-----------------------------------AGCTGTGGGCCTACTGGAGGC------------------------------------------------------------      0.000      0.493      0.602
-----------------------------------AGCTGTGGGCCTACTGGAG--------------------------------------------------------------      0.097      0.038      0.113
-----------------------------------AGCTGTGGGCCTACTGGAGG-------------------------------------------------------------      0.097      0.038      0.038
----------------------------------CAGCTGTGGGCCTACTGGAGG-------------------------------------------------------------      9.616      9.320      3.539
----------------------------------CAGCTGTGGGCCTACTGGAGGC------------------------------------------------------------      0.243      4.243      1.017
----------------------------------CAGCTGTGGGCCTACTGGAGGCG-----------------------------------------------------------      0.194      2.008      0.377
----------------------------------CAGCTGTGGGCCTACTGGAG--------------------------------------------------------------     38.806     31.635     15.964
------------
------------TCCAGCAGCACGCCGCAGCAG-----------------------------------------------------------------------------------      2.428      1.781      0.565

--------                                                                  C        AA           - TG  A-  G    G  TT
        GAGGTCCAGCAGCACGCCGCAGCAGGCAGCTGTGGGCCTACTGGAGGCGCCGCAGCGCAACAACA CGATTGCCA  GCCACAGCCA G  CG  GT CGAG GC  C
        CTCCAGGTCGTCGTGCGGCGTCGTCCGTCGACACCCGGATGACCTCCGCGGCGTCGCGTTGTTGT GCTAACGGT  CGGTGTCGGT C  GC  CG GCTT CG  T
TACTCGTC                                                                  A        CG           T GT  GG  -    G  TT

----CGTCCTCCAGGTCGTCG-----------------------------------------------------------------------------------------------      0.000      0.038      0.038
----CGTCCTCCAGGTCGTCGT----------------------------------------------------------------------------------------------      0.049      0.038      0.113
-------CCTCCAGGTCGTCGTGC--------------------------------------------------------------------------------------------      0.000      0.076      0.151
----------CCAGGTCGTCGTGCGGCGT---------------------------------------------------------------------------------------      0.340      0.189      0.188
-------CCTCCAGGTCGTCGTGCGGCGT---------------------------------------------------------------------------------------      0.146      0.871      1.393
---------TCCAGGTCGTCGTGCGGCGT---------------------------------------------------------------------------------------      0.146      0.038      0.226
--------CTCCAGGTCGTCGTGCGGCGT---------------------------------------------------------------------------------------      0.000      0.265      0.075
------------AGGTCGTCGTGCGGCGTCG-------------------------------------------------------------------------------------      0.097      0.038      0.038
-----------CAGGTCGTCGTGCGGCGTCG-------------------------------------------------------------------------------------      0.000      0.038      0.038
------------AGGTCGTCGTGCGGCGTCGT------------------------------------------------------------------------------------      2.866      0.909      0.941
---------TCCAGGTCGTCGTGCGGCGTCGT------------------------------------------------------------------------------------      2.866      3.523      0.301
----------CCAGGTCGTCGTGCGGCGTCGT------------------------------------------------------------------------------------    340.366    104.831      4.179 miR_t79
-----------CAGGTCGTCGTGCGGCGTCGT------------------------------------------------------------------------------------      5.780      3.296      0.866
-------------GGTCGTCGTGCGGCGTCGT------------------------------------------------------------------------------------      3.060      2.917      1.167
--------------------------------CCGTCGACACCCGGATGACCT---------------------------------------------------------------      1.117      0.833      0.339
---------------------------------CGTCGACACCCGGATGACCT---------------------------------------------------------------      0.097      0.038      0.226
-----------------------------------------CCCGGATGACCTCCGCGGCGT------------------------------------------------------      0.049      0.113      0.113
-----------------------------------------------TGACCTCCGCGGCGTCGCGTT------------------------------------------------      0.097      0.189      0.151
-----------------------------------------------------CCGCGGCGTCGCGTTGTT---------------------------------------------      0.000      0.075      0.075
--------------------------------------------------------CGGCGTCGCGTTGTTGT-------------------------------------------      0.097      0.303      0.264
----------------------------------------------------TCCGCGGCGTCGCGTTGTTGT-------------------------------------------      0.631      0.189      0.113
-------------------------------------------------------GCGGCGTCGCGTTGTTGT-------------------------------------------      0.049      0.265      0.038
------------------------------------------------------CGCGGCGTCGCGTTGTTGT-------------------------------------------      4.857      2.084      1.506
-----------------------------------------------------CCGCGGCGTCGCGTTGTTGT-------------------------------------------      2.137      1.440      0.527
--------------------------------------------------------CGGCGTCGCGTTGTTGTAG-----------------------------------------      1.748      4.546      2.297
--------------------------------------------------------CGGCGTCGCGTTGTTGTAGC----------------------------------------      2.137      1.023      0.414
--------------------------------------------------------CGGCGTCGCGTTGTTGTAGCT---------------------------------------    429.100   1520.293    964.388 miR_t43
-------------------------------------------------------GCGGCGTCGCGTTGTTGTAGCT---------------------------------------      4.517      2.879      0.414
---------------------------------------------------------GGCGTCGCGTTGTTGTAGCT---------------------------------------    237.110     59.481     32.229
----------------------------------------------------------GCGTCGCGTTGTTGTAGCT---------------------------------------      0.486      1.629      0.452
-----------------------------------------------------------CGTCGCGTTGTTGTAGCT---------------------------------------      0.000      0.720      0.038
---------------------------------------------------------GGCGTCGCGTTGTTGTAGCTA--------------------------------------      0.437      1.061      1.205
--------------------------------------------------------CGGCGTCGCGTTGTTGTAGCTA--------------------------------------      0.000      0.075      0.075
---------------------------------------------------------GGCGTCGCGTTGTTGTAGCTAA-------------------------------------      0.389      0.985      0.527
----------------------------------------------------------GCGTCGCGTTGTTGTAGCTAA-------------------------------------      0.583      5.153      2.184
-----------------------------------------------------------CGTCGCGTTGTTGTAGCTAA-------------------------------------      0.097      0.455      0.226
-----------------------------------------------------------CGTCGCGTTGTTGTAGCTAAC------------------------------------      0.243      0.985      1.167

miRNA: miR_t45 chromosome_12 Start: 9543557 End: 9543686 Condition: HS+N
--------------------------------TGATACTGCCCCTGTTCAC----GGCTCCT-------      0.097      0.227      0.075
--------------------------------TGATACTGCCCCTGTTCAC----GG------------      0.097      0.076      0.264
--------------------------------TGATACTGCCCCTGTTCAC----GGC-----------      0.049      0.265      1.807
----------------------TACCGTGACGTGATACTGCCCC-------------------------      1.311      4.016      2.447
----------------------TACCGTGACGTGATACTGCCC--------------------------      0.000      0.076      0.151
----------------------TACCGTGACGTGATACTGC----------------------------      0.000      0.076      0.038
----------------------TACCGTGACGTGATACTGCC---------------------------      0.049      0.076      0.038
----------------------TACCGTGACGTGATACTGCCCCT------------------------      0.049      0.038      0.301
--------------------TATACCGTGACGTGATACTGCC---------------------------      0.437      0.265      0.414
--------------------TATACCGTGACGTGATACTGC----------------------------      6.120      3.599      5.158
--------------AGAGCCTATACCGTGACGTGA----------------------------------      0.000      0.264      0.264
------------CAAGAGCCTATACCGTGACG-------------------------------------      0.097      0.152      0.151
----TTCGGGCCCAAGAGCCTA-----------------------------------------------      0.000      0.075      0.075

TGCG        C        A              A     C        ----      T    TC
    TTCGGGCC AAGAGCCT TACCGTGACGTGAT CTGCC CTGTTCAC    GGCTCC CTGT  A
    AAGTCCGG TTCTCGGA ATGGCGCTGCACTA GACGG GACAAGTG    TCGAGG GGCG  /
----        A        C              C     T        GTGG      T    TT

---------CGGATTCTCGGACATGG-------------------------------------------      0.000      0.038      0.038
---------CGGATTCTCGGACATGGC------------------------------------------      0.000      0.038      0.075
---------CGGATTCTCGGACATGGCG-----------------------------------------      1.166      3.182      3.238
----------GGATTCTCGGACATGGCG-----------------------------------------      0.000      0.076      0.414
----------GGATTCTCGGACATGGCGC----------------------------------------      0.097      0.227      0.753
---------CGGATTCTCGGACATGGCGC----------------------------------------      2.283      5.304      5.610
-----------GATTCTCGGACATGGCGCT---------------------------------------      0.874      1.553      1.581
---------CGGATTCTCGGACATGGCGCT---------------------------------------    665.724   2571.518   1544.361 miR_t45
--------CCGGATTCTCGGACATGGCGCT---------------------------------------      0.583      2.614      0.038
----------GGATTCTCGGACATGGCGCT---------------------------------------     20.301     93.882    137.313
-------------TTCTCGGACATGGCGCT---------------------------------------      0.000      0.076      0.038
----------GGATTCTCGGACATGGCGCTGC-------------------------------------      0.000      0.075      0.075
-----------GATTCTCGGACATGGCGCTGC-------------------------------------      0.000      0.303      0.678
--------------TCTCGGACATGGCGCTGCACT----------------------------------      0.097      0.076      0.075
-----------------CGGACATGGCGCTGCACTAC--------------------------------      0.000      0.038      0.038
-------------------GACATGGCGCTGCACTACGAC-----------------------------      0.097      0.038      0.113
----------------------ATGGCGCTGCACTACGACGGT--------------------------      0.000      0.038      0.038
------------------------GGCGCTGCACTACGACGGT--------------------------      0.000      0.038      0.113

miRNA: miR_t47 chromosome_12 Start: 3262556 End: 3262941 Condition: HS+N
-------------------------------------------------------------------------------------------------------------------------------------------------------------------GGAAGGGAGAACGGTGGC---------------------------------      0.146      0.114      0.264
-------------------------------------------------------------------------------------------------------------------------------------------------------------------GGAAGGGAGAACGGTGGCC--------------------------------      1.603      1.629      3.916
-------------------------------------------------------------------------------------------------------------------------------------------------------------------GGAAGGGAGAACGGTGGCCA-------------------------------      0.000      0.075      0.075
------------------------------------------------------------------------------------------------------------------------------------------------------------------CGGAAGGGAGAACGGTGGCCA-------------------------------      0.340      0.227      0.264
------------------------------------------------------------------------------------------------------------------------------------------------------------------CGGAAGGGAGAACGGTGGC---------------------------------      0.097      0.189      0.828
------------------------------------------------------------------------------------------------------------------------------------------------------------------CGGAAGGGAGAACGGTGGCC--------------------------------      5.100      3.107      8.170
-----------------------------------------------------------------------------------------------------------------------------------------------------------------TCGGAAGGGAGAACGGTGGCCAG------------------------------      0.097      0.076      0.113
-----------------------------------------------------------------------------------------------------------------------------------------------------------------TCGGAAGGGAGAACGGTGGCC--------------------------------   1040.525   1723.628   3273.287 miR_t47
-----------------------------------------------------------------------------------------------------------------------------------------------------------------TCGGAAGGGAGAACGGTGGC---------------------------------     72.172    239.289    306.214
-----------------------------------------------------------------------------------------------------------------------------------------------------------------TCGGAAGGGAGAACGGTGGCCA-------------------------------     26.858      6.895     18.411
----------------------------------------------------------------------------------------------------------------------------------------------------------------TTCGGAAGGGAGAACGGTGGCCA-------------------------------      0.049      0.038      0.075
----------------------------------------------------------------------------------------------------------------------------------------------------------------TTCGGAAGGGAGAACGGTGGCC--------------------------------      2.137      2.500      2.033
----------------------------------------------------------------------------------------------------------------------------------------------------------------TTCGGAAGGGAGAACGGTGGC---------------------------------     46.577    133.170    185.506
---------------------------------------------------------------------------------------------------------------------------------------------------------------TTTCGGAAGGGAGAACGGTGGC---------------------------------     43.517    138.019     46.198
---------------------------------------------------------------------------------------------------------------------------------------------------------------TTTCGGAAGGGAGAACGGTGGCC--------------------------------      0.243      0.114      0.075
--------------------------------------------------------------------------------------------------------------------------------------------------------------TTTTCGGAAGGGAGAACGGTGGC---------------------------------      0.243      0.909      0.264
---------------------------------------------------------------------------------------------------------------------------------TAGCGAACAACTTTGAGCAAACA--------------------------------------------------------------      0.049      0.151      0.151
-----------------------------------------------------------------------------------------------------------------------------TGGATAGCGAACAACTTTG----------------------------------------------------------------------      0.340      0.152      0.038
-----------------------------------------------------------------------------------------------------------------------------TGGATAGCGAACAACTTTGAG--------------------------------------------------------------------      4.274      0.455      0.264
-----------------------------------------------------------------------------------------------------------------------------TGGATAGCGAACAACTTTGAGC-------------------------------------------------------------------     12.336     25.952      1.807
-----------------------------------------------------------------------------------------------------------------------------TGGATAGCGAACAACTTTGA---------------------------------------------------------------------      0.291      0.038      0.113
--------------------------------------------------------------------------------------------------------------------------CGATGGATAGCGAACAACTTT-----------------------------------------------------------------------      0.000      0.188      0.188
------------------------------------------------TGGGGGCGAAAAACAACTTGT-------------------------------------------------------------------------------------------------------------------------------------------------      0.389      0.301      0.301
------------------------------------------TCGGGATGGGGGCGAAAAACA-------------------------------------------------------------------------------------------------------------------------------------------------------      0.000      0.038      0.038

--------------------------------------                                                                                                                                              C   TCC   C  TT-     C  -   CCCCC
                                      GGGGTCGGGATGGGGGCGAAAAACAACTTGTTAATCAACTCATCAAGTTGTTATTGCGTGCGCACCCAATCCCCCCGACACCCCCGATGGATAGCGAACAACTTTGAGCAAACATAGGGCTTTTCGGAAGGGAGAACGGTGG CAG   ACG TG   TGCGT CT CGC     T
                                      CCCCAGCCCTACCCCCGCTTTTTGTTGAACAATTAGTTGAGTAGTTCAACAATAACGCACGCGTGGGTTAGGGGGGCTGTGGGGGCTACCTATCGCTTGTTGAAACTCGTTTGTATCCCGAAAAGCCTTCCCTCTTGCCACC GTT   TGC AC   GCGCG GA GCG     C
GGTCCAATACCGACAAGACTCTTGTTTGAAGGCTAGCG                                                                                                                                              A   ---   C  TTC     C  C   CCCCC

----CAATACCGACAAGACTCTT-----------------------------------------------------------------------------------------------------------------------------------------------------------------------------------------------      0.000      0.038      0.038
-------------------------------------------GCCCTACCCCCGCTTTTTGTT------------------------------------------------------------------------------------------------------------------------------------------------------      0.000      0.151      0.151
-----------------------------------------------------------TTGTTGAACAATTAGTTGAG---------------------------------------------------------------------------------------------------------------------------------------      0.000      0.038      0.038
----------------------------------------------------------------------------------------------------------------GGCTGTGGGGGCTACCTAT-----------------------------------------------------------------------------------      0.777      0.417      0.715
--------------------------------------------------------------------------------------------------------------GGGGCTGTGGGGGCTACCTAT-----------------------------------------------------------------------------------      0.874      0.227      0.301
---------------------------------------------------------------------------------------------------------------GGGCTGTGGGGGCTACCTAT-----------------------------------------------------------------------------------      9.956      0.758      2.899
-----------------------------------------------------------------------------------------------------------------GCTGTGGGGGCTACCTATCGCT-------------------------------------------------------------------------------      3.157      0.417      0.377
-----------------------------------------------------------------------------------------------------------------GCTGTGGGGGCTACCTATCGCTT------------------------------------------------------------------------------      0.486      0.114      0.038
--------------------------------------------------------------------------------------------------------------------GTGGGGGCTACCTATCGCTT------------------------------------------------------------------------------      2.331      0.493      0.527
----------------------------------------------------------------------------------------------------------------------GGGGGCTACCTATCGCTT------------------------------------------------------------------------------      0.194      0.152      0.226
```

Read				
GGGGGCTACCTATCGCTTG	0.243	0.038	0.038	
TGGGGGCTACCTATCGCTTGT	0.000	0.151	0.151	
GGGGCTACCTATCGCTTGT	0.000	0.038	0.075	
GGGGGCTACCTATCGCTTGT	2.380	0.985	1.995	
GTGGGGGCTACCTATCGCTTGT	0.583	0.075	0.075	
GGGGGCTACCTATCGCTTGTT	37.106	22.883	28.125	
GGCTACCTATCGCTTGTT	0.729	0.227	0.226	
GGGGCTACCTATCGCTTGTT	1.408	0.455	0.678	
GGGCTACCTATCGCTTGTT	1.020	0.417	0.941	
GGGCTACCTATCGCTTGTTGA	0.000	0.151	0.151	

miRNA: miR_t84,miR_t48 chromosome_10 Start: 5682640 End: 5683026 Condition: HS+N

Read				
TCGGGATGGGGGCGAGAAAACA	0.000	0.038	0.075	
TCGGGATGGGGGCGAGAAAAC	0.000	0.076	0.715	
CGGGATGGGGGCGAGAAAAC	0.000	0.038	0.038	
ATGGGGGCGAGAAAACAACTTG	0.000	0.038	0.188	
TGGGGGCGAGAAAACAACTTGT	0.486	0.113	0.113	
TGGGGGCGAGAAAACAACTTG	7.140	1.970	7.003	
TGGGGGCGAGAAAACAACTT	0.049	0.076	0.075	
TGGGGGCGAGAAAACAACT	0.097	0.189	0.038	
GGGGGCGAGAAAACAACTTG	0.049	0.489	0.489	
CCCGATGGACAGCGAACAACT	0.097	0.227	0.038	
CCCGATGGACAGCGAACAAC	0.000	0.076	0.075	
CCGATGGACAGCGAACAAC	0.000	0.075	0.075	
CCGATGGACAGCGAACAACT	0.000	0.038	0.038	
CGATGGACAGCGAACAACTTT	0.097	0.377	0.377	
CGATGGACAGCGAACAACT	0.000	0.038	0.038	
GATGGACAGCGAACAACT	0.000	0.038	0.038	
ATGGACAGCGAACAACTTTGA	0.000	0.188	0.188	
TGGACAGCGAACAACTTTG	67.461	65.884	28.313	
TGGACAGCGAACAACTTT	0.194	0.341	0.113	
TGGACAGCGAACAACTTTGAGC	428.857	1387.237	45.859	miR_t84
TGGACAGCGAACAACTTTGA	70.230	35.158	11.408	
TGGACAGCGAACAACTTTGAG	201.364	55.238	14.721	
GGACAGCGAACAACTTTG	0.437	0.985	0.151	
GGACAGCGAACAACTTTGAGC	1.797	3.372	0.301	
TTTCGGAAGGGAGAACGGTGGTCAA	0.049	0.076	0.452	
TTTCGGAAGGGAGAACGGTGGTC	0.097	0.038	0.038	
TTTCGGAAGGGAGAACGGTGGT	6.217	8.524	3.502	
TTCGGAAGGGAGAACGGTGGTCAA	0.000	0.076	0.075	
TTCGGAAGGGAGAACGGTGGTCA	0.049	0.114	0.038	
TTCGGAAGGGAGAACGGTGGTC	1.263	2.576	2.108	
TTCGGAAGGGAGAACGGTGGT	13.745	14.776	20.746	
TCGGAAGGGAGAACGGTGGTCA	15.785	8.638	20.821	
TCGGAAGGGAGAACGGTGGT	53.862	29.854	52.448	
TCGGAAGGGAGAACGGTGGTC	987.925	1841.719	3408.529	miR_t48
CGGAAGGGAGAACGGTGGTC	4.954	3.334	11.107	
CGGAAGGGAGAACGGTGGTCA	0.146	0.114	0.038	
CGGAAGGGAGAACGGTGGT	0.146	0.114	0.414	
GGAAGGGAGAACGGTGGTC	1.554	1.326	3.765	
GGAAGGGAGAACGGTGGT	0.146	0.113	0.113	

```
--------------------------------------                G                 A                                                                                                                    A---   G  AA-   ----   G  G -  G
                                  GGGGTCGGGATGGGGGC AGAAAACAACTTGTTAA CAACTCATCAAGTTGTTATTGCGTGCGCACCCAATCCCCCCGACACCCCCGATGGACAGCGAACAACTTTGAGCAAACATAGGGCTTTTCGGAAGGGAGAACGGTGGTCA    ACG TG   GCG    CGC CT C GG A
                                  CCCCAGCCCTACCCCCG TTTTTTGTTGAACAATT GTTGAGTAGTTCAACAATAACGCACGCGTGGGTTAGGGGGGCTGTGGGGGCTACCTGTCGCTTGTTGAAACTCGTTTGTATCCCGAAAAGCCTTCCCTCTTGCCACCGGT    TGC AC   CGC    GTG GG G CC G
GGTCCAATACCGACAAGACTCTTGTTTGAAGGCTAGCG                -                 A                                                                                                                    CAGG   G  AAA   AGGA   G  G A  G
```

Read				
CTCTTGCCACCGGTCAGGT	0.000	0.038	0.038	
AGCCTTCCCTCTTGCCACCGGT	0.000	0.113	0.113	
GCCTTCCCTCTTGCCACCGGT	0.437	0.114	0.264	
GTCGCTTGTTGAAACTCGTTTGT	0.243	0.038	0.038	
GTCGCTTGTTGAAACTCGTTT	21.467	2.122	1.544	
CTGTCGCTTGTTGAAACTCGTT	0.826	0.417	0.339	
TGTCGCTTGTTGAAACTCGTT	3.400	0.417	0.301	
CCTGTCGCTTGTTGAAACTCGTT	0.097	0.038	0.038	
ACCTGTCGCTTGTTGAAACTCGT	0.049	0.114	0.038	
CTGTCGCTTGTTGAAACTCGT	2.477	0.265	0.075	
CCTGTCGCTTGTTGAAACTCGT	1.603	0.455	0.414	
GCTACCTGTCGCTTGTTGAAACT	0.243	0.606	0.715	

TACCTGTCGCTTGTTGAAACT	0.389	0.038	0.075	
ACCTGTCGCTTGTTGAAACT	7.285	1.402	1.167	
GGCTACCTGTCGCTTGTTGAAACT	0.000	0.075	0.075	
GCTACCTGTCGCTTGTTGAAA	0.097	0.189	1.017	
CTACCTGTCGCTTGTTGAA	0.000	0.075	0.075	
GGCTACCTGTCGCTTGTTGAA	3.643	2.425	2.861	
GCTACCTGTCGCTTGTTGAA	0.826	2.841	4.782	
GGGCTACCTGTCGCTTGTTGAA	0.049	0.265	0.075	
GGCTACCTGTCGCTTGTTGA	0.097	0.038	0.038	
GGGCTACCTGTCGCTTGTTGA	0.486	0.871	0.489	
TTGTTGAACAATTAGTTGAG	0.000	0.038	0.038	
ACCCCCG-TTTTTTGTTGAACAAT	0.000	0.038	0.038	
CCCTACCCCCG-TTTTTTGTT	0.000	0.227	0.151	
GCCCTACCCCCG-TTTTTTGTT	0.437	0.152	0.452	
GCCCTACCCCCG-TTTTTTGT	0.291	0.339	0.339	
AGCCCTACCCCCG-TTTTTTGT	0.000	0.151	0.151	
CCAGCCCTACCCCCG-TTTTTT	1.408	0.493	1.694	
CAGCCCTACCCCCG-TTTTTT	0.049	0.038	0.075	
GCCCCAGCCCTACCCCCG-TTTTT	0.000	0.075	0.075	
CCCCAGCCCTACCCCCG-TTTTT	0.000	0.038	0.038	
CCCAGCCCTACCCCCG-TTTTT	0.097	0.152	0.188	
CCAGCCCTACCCCCG-TTTTT	0.534	0.227	0.452	
GCCCCAGCCCTACCCCCG-TTTT	0.097	0.113	0.113	
CCCCAGCCCTACCCCCG-TTT	0.000	0.038	0.038	
GCCCCAGCCCTACCCCCG-TTT	0.291	0.152	0.452	
GCGCCCCAGCCCTACCCCCG-TT	0.000	0.075	0.075	
CGCCCCAGCCCTACCCCCG-TT	0.146	0.038	0.075	
GCCCCAGCCCTACCCCCG-TT	0.000	0.038	0.038	
GCCCCAGCCCTACCCCCG-T	0.000	0.038	0.038	
GCGCCCCAGCCCTACCCCCG-T	0.000	0.075	0.075	
CAATACCGACAAGACTCTT	0.000	0.038	0.038	

miRNA: miR_t112,miR_t56,miR_t4 chromosome_5 Start: 3227662 End: 3227766 Condition: HS+N

Read				
TCACGCCTCTCGCCCAGAAGG	0.049	0.417	0.264	
TTCACGCCTCTCGCCCAGAAG	0.000	0.038	0.038	
CACACTTTTCACGCCTCTC	1.797	0.303	0.715	
CACACTTTTCACGCCTCT	2.040	0.264	0.264	
TCCACACTTTTCACGCCTCT	7.334	0.909	1.242	
TCCACACTTTTCACGCCTCTC	235.361	22.429	35.881	
TCCACACTTTTCACGCCTC	0.194	0.038	0.038	
TCCACACTTTTCACGCCTCTCG	8.597	1.326	3.087	
TCCACACTTTTCACGCC	0.049	0.038	0.038	
TTCCACACTTTTCACGCCTC	8.888	1.174	1.017	
TTCCACACTTTTCACGCCT	0.291	0.038	0.075	
TTCCACACTTTTCACGCCTCTC	45.800	18.526	22.929	
TTCCACACTTTTCACGCC	0.340	0.076	0.038	
TTCCACACTTTTCACGC	0.049	0.227	0.527	
TTCCACACTTTTCACGCCTCT	522.545	118.129	106.627	miR_t112
TTCCACACTTTTCACGCCTCTCG	40.360	25.005	38.178	
ATTCCACACTTTTCACGCCTC	0.049	0.075	0.075	

```
---------                                            AGT
         GCCCATTCCACACTTTTCACGCCTCTCGCCCAGAAGGCAGCAGC   C
         CGGGTAAGGTGTGAAAAGTGCGGAGAGCGGGTCTTCCGTCGTCG   /
TCGCCACGG                                            CCC
```

Read				
ACGGCGGGTAAGGTGTGAAA	0.000	0.151	0.151	
CACGGCGGGTAAGGTGTGAAA	0.000	0.038	0.226	
ACGGCGGGTAAGGTGTGAAAA	0.000	0.189	0.301	
CACGGCGGGTAAGGTGTGAAAA	0.000	0.076	0.038	
CGGCGGGTAAGGTGTGAAAA	0.000	0.152	0.489	
CGGCGGGTAAGGTGTGAAAAG	0.097	0.189	0.414	
GGCGGGTAAGGTGTGAAAAG	0.486	0.493	1.280	
ACGGCGGGTAAGGTGTGAAAAGT	0.146	0.303	0.715	
GGCGGGTAAGGTGTGAAAAGT	35.115	88.653	150.904	miR_t56
GCGGGTAAGGTGTGAAAAGT	0.243	0.038	0.151	
GGTAAGGTGTGAAAAGT	0.340	0.606	0.527	
CGGCGGGTAAGGTGTGAAAAGT	2.186	9.585	9.601	
GGGTAAGGTGTGAAAAGT	0.243	0.152	0.339	
CGGGTAAGGTGTGAAAAGT	0.146	0.530	1.920	
GTAAGGTGTGAAAAGTGCGG	0.000	0.113	0.113	
GGTAAGGTGTGAAAAGTGCGG	0.243	0.303	0.414	
GTAAGGTGTGAAAAGTGCGGA	1.117	1.440	2.184	miR_t112*
GGTAAGGTGTGAAAAGTGCGGA	0.291	0.568	0.339	
TAAGGTGTGAAAAGTGCGGAG	2.477	0.682	1.130	
GTAAGGTGTGAAAAGTGCGGAG	0.049	0.113	0.113	
GGTGTGAAAAGTGCGGAG	0.000	0.075	0.075	
AGGTGTGAAAAGTGCGGAG	0.000	0.113	0.113	
AAGGTGTGAAAAGTGCGGAG	0.000	0.076	0.038	
GGTGTGAAAAGTGCGGAGA	0.146	0.152	0.151	
TGTGAAAAGTGCGGAGA	0.000	0.038	0.038	
AAGGTGTGAAAAGTGCGGAGA	2.040	1.061	2.071	miR_t4
GAAAAGTGCGGAGAGCGGGT	0.291	0.568	1.130	
TGAAAAGTGCGGAGAGCGGGT	0.097	0.189	0.640	
AAAGTGCGGAGAGCGGGTCT	0.000	0.113	0.113	
GAAAAGTGCGGAGAGCGGGTCT	0.194	0.076	0.038	
AAAAGTGCGGAGAGCGGGTCT	0.097	0.038	0.038	
TGAAAAGTGCGGAGAGCGGGTCTT	0.000	0.151	0.151	
AAAGTGCGGAGAGCGGGTCTT	0.194	0.568	1.167	
AAAAGTGCGGAGAGCGGGTCTT	0.049	0.985	0.339	
AGTGCGGAGAGCGGGTCTT	0.000	0.151	0.151	
GAAAAGTGCGGAGAGCGGGTCTT	0.000	0.758	0.113	
AAGTGCGGAGAGCGGGTCTT	0.000	0.038	0.075	
GCGGAGAGCGGGTCTTCCGT	0.000	0.113	0.113	

miRNA: miR_t63 chromosome_7 Start: 3871147 End: 3871578 Condition: HS+N

```
--------------------------------------------------------------------------------------------------TGACGCCGCACCTCAAGTCCTG----------------------------------------------------------------------------------------------------      0.000      0.038      0.038
-----------------------------------------------------------------------------------TGCCCGTCAGGAAGGTGACGC--------------------------------------------------------------------------------------------------------------------      0.146      0.833      1.167
-----------------------------------------------------------------------------------TGCCCGTCAGGAAGGTGACG---------------------------------------------------------------------------------------------------------------------      0.534      0.152      0.075
-----------------------------------------------------------------AAGGGCTGCGAGCAGGAG-----------------------------------------------------------------------------------------------------------------------------------------      0.000      0.151      0.151
-----------------------------------------------------------------AAGGGCTGCGAGCAGGAGCTG--------------------------------------------------------------------------------------------------------------------------------------      0.000      0.114      0.188
---------------------------------------------------------------GCAAGGGCTGCGAGCAGGAG-----------------------------------------------------------------------------------------------------------------------------------------      0.243      0.303      1.958
---------------------------------------------------------------GCAAGGGCTGCGAGCAGGAGC----------------------------------------------------------------------------------------------------------------------------------------      0.146      0.114      0.226
--------------------------------------------------------------TGCAAGGGCTGCGAGCAGGAG-----------------------------------------------------------------------------------------------------------------------------------------      34.289     71.908     261.937 miR_t63
--------------------------------------------------------------TGCAAGGGCTGCGAGCAGG-------------------------------------------------------------------------------------------------------------------------------------------      0.000      0.076      0.038
--------------------------------------------------------------TGCAAGGGCTGCGAGCAGGAGC----------------------------------------------------------------------------------------------------------------------------------------      22.924     54.442     46.612
-------------------------------------------------------------CTGCAAGGGCTGCGAGCAGGAG-----------------------------------------------------------------------------------------------------------------------------------------      0.049      0.076      0.113
--------------------------TGACGGGCGGCCACACCTGGG-----------------------------------------------------------------------------------------------------------------------------------------------------------------------------      0.049      0.075      0.075
----------------------TCAATGACGGGCGGCCACACC---------------------------------------------------------------------------------------------------------------------------------------------------------------------------------      0.437      0.152      0.489
------------------CGGATCAATGACGGGCGGCCA-------------------------------------------------------------------------------------------------------------------------------------------------------------------------------------      0.000      0.038      0.038
-------------TGAAGCGGATCAATGACGGGC------------------------------------------------------------------------------------------------------------------------------------------------------------------------------------------      0.049      0.038      0.038

---------                                               C                                             C                      C    G                                                                      TTCCCCAGGGAGA   C  T
         AGTATGAAGCGGATCAATGACGGGCGGCCACACCTGGGGAGTTCACC TGACCTGCAAGGGCTGCGAGCAGGAGCTGCCCGTCAGGAAGGTGA GCCGCACCTCAAGTCCTGGGACA TGGT CCAACACCTTCAAGAAGACCGTGCTCTTCCGTTTCAAGTGCAACAAGTGCACAAAGTTCGCCACGTACTCC             AGG GC G
         TCATACTTCGCCTAGTTACTGCCCGCCGGTGTGGACCCCCTCAAGTGG ACTGGACGTTCCCGACGCTCGTCCTCGACGGGCAGTCCTTCCACT CGGCGTGGAGTTCAGGACCCTGT ACCA GGTTGTGGAAGTTCTTCTGGCACGAGAAGGCAAAGTTCACGTTGTTCACGTGTTTCAAGCGGTGCATGAGG             TCC CG A
TGATGTCCG                                               A                                             A                      T    A                                                                      TCG----------   A  A

----GTCCGTCATACTTCGCCTAG----------------------------------------------------------------------------------------------------------------------------------------------------------------------------------------------------      0.243      0.038      0.038
------CCGTCATACTTCGCCTAGTT--------------------------------------------------------------------------------------------------------------------------------------------------------------------------------------------------      0.049      0.038      0.038
------------------GCCTAGTTACTGCCCGCCGGT-------------------------------------------------------------------------------------------------------------------------------------------------------------------------------------      0.583      0.113      0.113
---------------------------------------------------------------------------------CGACGGGCAGTCCTTCCACT-----------------------------------------------------------------------------------------------------------------------      1.166      1.326      0.715
-------------------------------------------------------------------------------------------------------GGCGTGGAGTTCAGGACC---------------------------------------------------------------------------------------------------      0.000      0.038      0.038
------------------------------------------------------------------------------------------------------CGGCGTGGAGTTCAGGACCCT-------------------------------------------------------------------------------------------------      4.760      15.420     18.524
--------------------------------------------------------------------------------------------------------GCGTGGAGTTCAGGACCCT-------------------------------------------------------------------------------------------------      0.000      0.038      0.038
-----------------------------------------------------------------------------------------------------ACGGCGTGGAGTTCAGGACCCT-------------------------------------------------------------------------------------------------      0.146      0.075      0.075
-------------------------------------------------------------------------------------------------------GGCGTGGAGTTCAGGACCCT-------------------------------------------------------------------------------------------------      0.340      0.227      0.038
----------------------------------------------------------------------------------------------------TACGGCGTGGAGTTCAGGACCCT-------------------------------------------------------------------------------------------------      0.049      0.038      0.075
--------------------------------------------------------------------------------------------------------GCGTGGAGTTCAGGACCCTGT-----------------------------------------------------------------------------------------------      0.340      0.038      0.038
------------------------------------------------------------------------------------------------------CGGCGTGGAGTTCAGGACCCTGT-----------------------------------------------------------------------------------------------      0.000      0.076      0.038
--------------------------------------------------------------------------------------------------------------------------------------------------CTGGCACGAGAAGGCAAAGT------------------------------------------------------      0.097      0.038      0.038
-------------------------------------------------------------------------------------------------------------------------------------------------TCTGGCACGAGAAGGCAAAGT------------------------------------------------------      0.631      0.265      0.565
--------------------------------------------------------------------------------------------------------------------------------------------------CTGGCACGAGAAGGCAAAGTT-----------------------------------------------------      0.000      0.038      0.038
--------------------------------------------------------------------------------------------------------------------------------------------------------CGAGAAGGCAAAGTTCACGTT-----------------------------------------------      0.340      1.402      0.452
------------------------------------------------------------------------------------------------------------------------------------------------------------------------CGTTGTTCACGTGTTTCAAGC-------------------------------      0.000      0.038      0.038
---------------------------------------------------------------------------------------------------------------------------------------------------------------------------------CGTGTTTCAAGCGGTGCAT------------------------      0.340      0.606      0.301

miRNA: miR_t68 chromosome_2 Start: 5502478 End: 5502846 Condition: HS+N
--------------TCCGGGAGAATGACATGACTGTG---------------------------------------------------------------------------------------------------------------------------------------------------------      0.000      0.075      0.075
----------------CGGGAGAATGACATGACTGTG---------------------------------------------------------------------------------------------------------------------------------------------------------      0.049      0.114      0.414
-------------------GAGAATGACATGACTGTGCCG------------------------------------------------------------------------------------------------------------------------------------------------------      0.923      0.076      0.188
------------------------TGACATGACTGTGCCGGAGACA------------------------------------------------------------------------------------------------------------------------------------------------      0.291      0.644      0.151
-----------------------------TGACTGTGCCGGAGACAAAC---------------------------------------------------------------------------------------------------------------------------------------------      0.049      0.152      0.038
-----------------------------TGACTGTGCCGGAGACAAACA--------------------------------------------------------------------------------------------------------------------------------------------      0.923      0.833      0.828
---------------------------------------------------------TCCAGGTCGCCGGGGAATGGC----------------------------------------------------------------------------------------------------------------      2.186      2.425      2.598
-----------------------------------------------------------CAGGTCGCCGGGGAATGGC----------------------------------------------------------------------------------------------------------------      0.000      0.038      0.038
------------------------------------------------------------------------------------------------------TCCTCCAGGGTCAGCTCTGTG-------------------------------------------------------------------      0.583      0.038      0.038
----------------------------------------------------------------------------------------------------------------TCAGCTCTGTGCCAAGCTGC----------------------------------------------------------      0.000      0.075      0.075
---------------------------------------------------------------------------------------------------------------------------------TGCTGCACGCCTGCCACCCC-----------------------------------------      0.049      0.038      0.038
---------------------------------------------------------------------------------------------------------------------------------TGCTGCACGCCTGCCACCCCA----------------------------------------      0.000      0.038      0.038
---------------------------------------------------------------------------------------------------------------------------------TGCTGCACGCCTGCCACCCCCAT--------------------------------------      0.000      0.075      0.075
------------------------------------------------------------------------------------------------------------------------------------TGCACGCCTGCCACCCCCATG-------------------------------------      0.049      0.114      0.226
--------------------------------------------------------------------------------------------------------------------------------------------------------------------TGGACCAGGGATCCGAGCCAC-----      0.000      0.113      0.113
--------------------------------------------------------------------------------------------------------------------------------------------------------------------TGGACCAGGGATCCGAGCCACG----      0.243      0.152      0.640
--------------------------------------------------------------------------------------------------------------------------------------------------------------------TGGACCAGGGATCCGAGCCACGC---      0.583      0.417      0.640
--------------------------------------------------------------------------------------------------------------------------------------------------------------------TGGACCAGGGATCCGAGC--------      0.000      0.038      0.038

---------           A                  G                                      C                          T                                        C       G                  GA   G   CAC  TG
          CACGTCCGGG GAATGACATGACTGTGCC GAGACAAACACCACCAGCTCCAGGTCGCCGGGGAATGGC CGAGGTCCAGCTTCTTGCGCAGCTCC CCAGGGTCAGCTCTGTGCCAAGCTGCTGCACGCCTGCCAC CCCATGG GCCCAGCCCCTGGACCAGG  TCC AGC   GC  C
          GTGCAGGTCC CTTACTGTACTGACACGG CTCTGTTTGTGGTGGTCGAGGTCCAGCGGCCCCTTACCG GCTCCAGGTCGAAGAACGCGTCGAGG GGTCCCAGTCGAGACACGGTTCGACGACGTGCGGACGGTG GGGTACC CGGGTCGGGGACCTGGTCC  AGG TCG   CG  /
GCAGTGTCCG          A                  A                                      A                          C                                        C       G                  --   -   T--  TT

-------------------------------------------------------------------------------------------------------------------------------------------GACGGTGCGGGTACCGCGGGT------------------------------      0.000      0.038      0.038
----------------------------------------------------------------------------------------------------------------------------------CGACGTGCGGACGGTGCGGGT---------------------------------------      1.506      1.440      3.652
----------------------------------------------------------------------------------------------------------GGTCCCAGTCGAGACACGGTT---------------------------------------------------------------      2.768      1.781      1.995
---------------------------------------------------------------------------------------------------------CGGTCCCAGTCGAGACACGGTT---------------------------------------------------------------      0.097      0.189      0.264
----------------------------------------------------------------------------------------------------------GGTCCCAGTCGAGACACGGT----------------------------------------------------------------      0.097      0.075      0.075
---------------------------------------------------------------------------------------------------------CGGTCCCAGTCGAGACACGGT----------------------------------------------------------------      0.097      0.265      0.527
----------------------------------------------------------------------------------------------------CGAGGCGGTCCCAGTCGAGAC---------------------------------------------------------------------      0.194      0.265      0.678
-----------------------------------------------------------------------------------------------CGCGTCGAGGCGGTCCCAGT---------------------------------------------------------------------------      0.437      0.038      0.075
-----------------------------------------------------------------------------------------GAAGAACGCGTCGAGGCGGT---------------------------------------------------------------------------------      0.000      0.114      0.113
----------------------------------------------------------------------------------------CGAAGAACGCGTCGAGGCGGT---------------------------------------------------------------------------------      1.360      6.895      11.333
----------------------------------------------------------------------------------------CGAAGAACGCGTCGAGGCGG----------------------------------------------------------------------------------      0.000      0.075      0.075
-------------------------------------------------------------------------------GCTCCAGGTCGAAGAACGCGTC-----------------------------------------------------------------------------------------      0.000      0.038      0.038
-------------------------------------------------------------------------------GCTCCAGGTCGAAGAACGCGT------------------------------------------------------------------------------------------      72.609     40.462     85.618 miR_t68
----------------------------------------------------------------------------CGAGCTCCAGGTCGAAGAACGCGT------------------------------------------------------------------------------------------      0.097      0.113      0.113
------------------------------------------------------------------------------AGCTCCAGGTCGAAGAACGCGT------------------------------------------------------------------------------------------      0.826      0.417      0.264
--------------------------------------------------------------------------------CTCCAGGTCGAAGAACGCGT------------------------------------------------------------------------------------------      0.000      0.152      0.113
-------------------------------------------------------------------------------GCTCCAGGTCGAAGAACGCG-------------------------------------------------------------------------------------------      0.583      0.341      1.205
----------------------------------------------------------------------CCTTACCGAGCTCCAGGTCGAA--------------------------------------------------------------------------------------------------      0.000      0.113      0.113
------------------------------------------------------------------GGCCCCTTACCGAGCTCCAGGT------------------------------------------------------------------------------------------------------      0.097      0.152      0.264
-------------------------------------------------------------------GCCCCTTACCGAGCTCCAGGT------------------------------------------------------------------------------------------------------      0.049      0.227      0.226
-----------------------------------------------------------------CGGCCCCTTACCGAGCTCCAGGT------------------------------------------------------------------------------------------------------      0.000      0.038      0.038
-------------------------------------------CTGTTTGTGGTGGTCGAGGT-------------------------------------------------------------------------------------------------------------------------------      0.000      0.038      0.151
-----------------------------------ACGGACTCTGTTTGTGGTGGT--------------------------------------------------------------------------------------------------------------------------------------      0.680      2.425      2.711
------------------------------------CGGACTCTGTTTGTGGTGGT--------------------------------------------------------------------------------------------------------------------------------------      0.534      0.985      1.242
-----------------------------------ACGGACTCTGTTTGTGGTGG---------------------------------------------------------------------------------------------------------------------------------------      0.000      0.113      0.113
---------------------------GTACTGACACGGACTCTGTTT----------------------------------------------------------------------------------------------------------------------------------------------      0.000      0.038      0.038
--------CGGTGCAGGTCCACTTACTGT-----------------------------------------------------------------------------------------------------------------------------------------------------------------      0.389      0.189      0.226
-----GTCCGGTGCAGGTCCACTTACT-------------------------------------------------------------------------------------------------------------------------------------------------------------------      0.000      0.076      0.075
----TGTCCGGTGCAGGTCCACTTACT-------------------------------------------------------------------------------------------------------------------------------------------------------------------      0.000      0.075      0.075

miRNA: miR_t69 chromosome_4 Start: 3822975 End: 3823349 Condition: HS+N
---------------------------------------------------------------------------------------------------TCGTCAAGGTGGTGAGCACGC--------------------------------------------      0.000      0.038      0.038
--------------------------------------------------------------------------------------------------ATCGTCAAGGTGGTGAGCACGC--------------------------------------------      0.000      0.152      0.152
----------------------------------------------------------------------------------------TACTCCAAACCATCGTCAAGG-------------------------------------------------------      0.389      0.189      0.075
---------------------------------------------------------------------------------TGCGCATACTCCAAACCATCGTCA-----------------------------------------------------------      0.000      0.076      0.076
-----------------------------------------------------------------------------CCGCTGCGCATACTCCAAACC------------------------------------------------------------------      0.097      0.341      0.188
-----------------------------------------------------------------TTTCCAGATGATGCCGCTGC-------------------------------------------------------------------------------      0.000      0.038      0.038
----------------------TGCAGAAGCTGCAGACCCGCG-------------------------------------------------------------------------------------------------------------------------      0.340      0.189      0.075
----TCCGCGCGCTGGGGCAGCTGC-------------------------------------------------------------------------------------------------------------------------------------------      0.000      0.038      0.264

C                                              GGGCACCGTC                                A   C       C             GCAC   A-    -   ACCAGCATCTG      C-----        A
 GCGTCCGCGCGCTGGGGCAGCTGCAGAAGCTGCAGACCCGCGTGGC          GCGGCGGGTTTCCAGATGATGCCGCTGCGCAT CTC AAACCAT GTCAAGGTGGTGA    GCA  GGCG CTC           ATTTGC     GTGCGCGT A
 CGCGGGCGCGCGACCCCGTCGACGTCTTCGACGTCTGGGCGCACCG          CGCCGCCCAAAGGTCTACTACGGCGACGCGTA GAG TTTGGTA CAGTTCCACCACT    CGT  CCGC GAG           TAAACG     CACGCGCG G
T                                              ----------                                C   C       A             AGC-   CG    C   G----------      CCGCAC        G

-----------------------------------------GCACCG----------CGCCGCCCAAAGGTCT-------------------------------------------------------------------------------------------      0.000      0.076      0.076
-------------------------------------------ACCG----------CGCCGCCCAAAGGTCT-------------------------------------------------------------------------------------------      0.000      0.076      0.076
---------------------------------------------CG----------CGCCGCCCAAAGGTCTACT----------------------------------------------------------------------------------------      0.000      0.189      0.113
----------------------------------------------G----------CGCCGCCCAAAGGTCTACT----------------------------------------------------------------------------------------      0.000      0.038      0.038
-------------------------------------------------------------------AGGTCTACTACGGCGACGCG-----------------------------------------------------------------------------      0.000      0.417      0.414
--------------------------------------------------------------------GGTCTACTACGGCGACGCG-----------------------------------------------------------------------------      0.097      0.038      0.038
------------------------------------------------------------------AAGGTCTACTACGGCGACGCGT----------------------------------------------------------------------------      0.146      0.379      0.113
----------------------------------------------------------------------TCTACTACGGCGACGCGT----------------------------------------------------------------------------      0.000      0.796      2.146
-----------------------------------------------------------------------CTACTACGGCGACGCGT----------------------------------------------------------------------------      0.000      0.114      0.264
-----------------------------------------------------------------AAAGGTCTACTACGGCGACGCGT----------------------------------------------------------------------------      0.000      0.038      0.038
--------------------------------------------------------------------GGTCTACTACGGCGACGCGT----------------------------------------------------------------------------      10.879     25.497     21.084
-------------------------------------------------------------------AGGTCTACTACGGCGACGCGT----------------------------------------------------------------------------      7.042      40.652     39.082 miR_t69
---------------------------------------------------------------------GTCTACTACGGCGACGCGT----------------------------------------------------------------------------      0.000      0.076      0.113
--------------------------------------------------------------------GGTCTACTACGGCGACGCGTA---------------------------------------------------------------------------      0.000      0.152      0.151
--------------------------------------------------------------------GGTCTACTACGGCGACGCGTAC--------------------------------------------------------------------------      0.000      0.303      0.075
-------------------------------------------------------------------------ACTACGGCGACGCGTACGAGC----------------------------------------------------------------------      0.000      0.076      0.113
--------------------------------------------------------------------------CTACGGCGACGCGTACGAGCT---------------------------------------------------------------------      0.049      0.076      0.038
-----------------------------------------------------------------------------CGGCGACGCGTACGAGCTT--------------------------------------------------------------------      0.000      0.076      0.188
------------------------------------------------------------------------------GGCGACGCGTACGAGCTT--------------------------------------------------------------------      0.000      0.038      0.038
----------------------------------------------------------------------------ACGGCGACGCGTACGAGCTT--------------------------------------------------------------------      0.194      0.530      0.377
-----------------------------------------------------------------------------CGGCGACGCGTACGAGCTTT-------------------------------------------------------------------      0.000      0.341      0.226
----------------------------------------------------------------------------ACGGCGACGCGTACGAGCTTT-------------------------------------------------------------------      8.742      19.246     24.398
--------------------------------------------------------------------------CTACGGCGACGCGTACGAGCTTT-------------------------------------------------------------------      0.000      0.038      0.038
---------------------------------------------------------------------------TACGGCGACGCGTACGAGCTTT-------------------------------------------------------------------      0.000      0.152      0.151
-------------------------------------------------------------------------------GCGACGCGTACGAGCTTTGGT----------------------------------------------------------------      0.000      0.114      0.301
--------------------------------------------------------------------------------------GTACGAGCTTTGGTAACAGTT---------------------------------------------------------      0.000      0.076      0.076

miRNA: miR_t71 chromosome_17 Start: 2561427 End: 2561795 Condition: HS+N
-------------TCTCCAGCGTCACTGACTCGATG------------------------------------------------------------------------------------------------------------------------------------------------------------      0.049      0.075      0.075
---------------TCCAGCGTCACTGACTCGATG------------------------------------------------------------------------------------------------------------------------------------------------------------      0.389      0.530      0.113
---------------------------------------------------------------------TGCATCTTGTGCACGCGGCTG------------------------------------------------------------------------------------------------------      0.097      0.075      0.075
---------------------------------------------------------------------TGCATCTTGTGCACGCGGCTGC-----------------------------------------------------------------------------------------------------      0.000      0.075      0.075
----------------------------------------------------------------------------TGTGCACGCGGCTGCCCTTGC-----------------------------------------------------------------------------------------------      0.000      0.152      0.226
------------------------------------------------------------------------------TGCACGCGGCTGCCCTTGCGGG--------------------------------------------------------------------------------------------      0.291      0.038      0.038
---------------------------------------------------------------------------------------------------------------TACCAGCACCTCATCCTCGCG------------------------------------------------------------      0.340      0.038      0.452
-------------------------------------------------------------------------------------------------------------------------------TCGCGCAGGTACAGCATGATG--------------------------------------------      0.049      0.075      0.075
----------------------------------------------------------------------------------------------------------------------------------------TACAGCATGATGGCCGCCGCC-----------------------------------      0.583      0.758      6.363
-----------------------------------------------------------------------------------------------------------------------------------------ACAGCATGATGGCCGCCGCC-----------------------------------      0.000      0.113      0.113
------------------------------------------------------------------------------------------------------------------------------------------CAGCATGATGGCCGCCGCC-----------------------------------      0.000      0.038      0.038

---------          G                           C   ----                C             G           G                     G                    G                 C      A   A      ----  TG A    CA
         CACGTCTCCA CGTCACTGACTCGATGACGCGTGCGAG GACT    TGGCGCCGCTCGCCTG ATCTTGTGCACGC GCTGCCCTTGC GGCCGCCGGCACCTACCAGCA CTCATCCTCGCGCAGGTACA CATGATGGCCGCCGCCA CTACGC GCG GCAGC    GGG  C GGTG  C
         GTGCAGAGGT GCGGTGGCTGAGCTGCTGCGCACGCTC CTGA    ACCGCGGCGAGCGGAC TAGAACACGTGCG CGACGGGAACG CCGGCGGCCGTGGATGGTCGT GAGTAGGAGCGCGTCCATGT GTACTACCGGCGGCGGT GATGTG CGC CGTTG    CCC  G CCGC  /
GAGTAGACG          A                           A   ACTA                C             G           A                     A                    A                 C      C   -      AAGA  GT -   CC

---------------------------------------------------------------------------------------------------------------------------GTAGGAGCGCGTCCATGTAGT------------------------------------------------      0.729      0.227      0.452
-----------------------------------------------------------------------------------------------------------------------AGAGTAGGAGCGCGTCCATGT---------------------------------------------------      0.000      0.076      0.113
---------------------------------------------------------------------------------------------------------------------GTAGAGTAGGAGCGCGTCCAT-----------------------------------------------------      0.437      0.227      0.075
----------------------------------------------------------------------------------------------------------------------TAGAGTAGGAGCGCGTCCAT-----------------------------------------------------      0.000      0.075      0.075
------------------------------------------------------------------------------------------------------------------GTCGTAGAGTAGGAGCGCGT---------------------------------------------------------      0.000      0.038      0.038
-----------------------------------------------------------------------------------------------------------------GGTCGTAGAGTAGGAGCGCGT---------------------------------------------------------      49.880     38.151     116.906 miR_t71
----------------------------------------------------------------------------------------------------------------TGGTCGTAGAGTAGGAGCGCGT---------------------------------------------------------      0.049      0.265      0.151
-----------------------------------------------------------------------------------------------------------------GGTCGTAGAGTAGGAGCGCG----------------------------------------------------------      0.291      0.114      0.226
--------------------------------------------------------------------------------------------------------------GGATGGTCGTAGAGTAGGAGC------------------------------------------------------------      0.000      0.075      0.075
----------------------------------------------------------------------------------------------CGACCGGCGGCCGTGGATGGT----------------------------------------------------------------------------      0.000      0.189      0.226
-------------------------------------------------------------------------------------------GAACGACCGGCGGCCGTGGAT-------------------------------------------------------------------------------      0.971      0.455      1.355
```

```
----------------------------------------------------------------------------------------------GGAACGACCGGCGGCCGTGGAT----------------------------------------------------------------------------------      0.146      0.038      0.113
-----------------------------------------------------------------------------------------------GAACGACCGGCGGCCGTGGA-----------------------------------------------------------------------------------      0.000      0.075      0.075
--------------------------------------------------------------------------------------------CGGGAACGACCGGCGGCCGT--------------------------------------------------------------------------------------      0.000      0.038      0.038
--------------------------------------------------------------GGCGAGCGGACCTAGAACACGT------------------------------------------------------------------------------------------------------------------      0.049      0.075      0.075
-----------------------------------------------------------------GAGCGGACCTAGAACACGT------------------------------------------------------------------------------------------------------------------      0.000      0.151      0.151
----------------------------------------------------------------CGAGCGGACCTAGAACACGT------------------------------------------------------------------------------------------------------------------      0.000      0.151      0.151
-------------------------TGGCTGAGCTGCTGCGCACGCT-------------------------------------------------------------------------------------------------------------------------------------------------------      0.243      0.113      0.113
--------------------------GGCTGAGCTGCTGCGCACGCT-------------------------------------------------------------------------------------------------------------------------------------------------------      1.991      0.303      0.301
--------------GAGGTAGCGGTGGCTGAGC---------------------------------------------------------------------------------------------------------------------------------------------------------------------      0.000      0.075      0.075
----------TGCAGAGGTAGCGGTGGCT-------------------------------------------------------------------------------------------------------------------------------------------------------------------------      0.000      0.038      0.038
--------GGTGCAGAGGTAGCGGTGGCT-------------------------------------------------------------------------------------------------------------------------------------------------------------------------      0.291      0.076      0.715
-------CGGTGCAGAGGTAGCGGTGGCT-------------------------------------------------------------------------------------------------------------------------------------------------------------------------      0.000      0.076      0.038
----AGACGGTGCAGAGGTAGCGGT-----------------------------------------------------------------------------------------------------------------------------------------------------------------------------      0.097      0.038      0.038

miRNA: miR_t74 chromosome_14 Start: 3469579 End: 3469793 Condition: HS+N
----TGCACCACCACACACGCCAAC-------------------------------------------------------------------------------------------------------------------------------------------------      0.340      0.152      0.152
------------------------------------------------------TGCCCATGCCCCGCGCCGTACG----------------------------------------------------------------------------------------------      0.049      0.038      0.038
------------------------------------------------------TGCCCATGCCCCGCGCCGTAC-----------------------------------------------------------------------------------------------      0.194      0.076      0.076
-----------------------------------------------------------------------------------------------------------------------------------TATTGCCGGCGGACTGCAGC-------------------      0.049      0.114      0.114

CCACTGCACCACCACACACGCCAACGCAAAAATGCCACCAATGCCGGCGGCTATGCCCATGCCCCGCGCCGTACGCGACCCGTGCTCCTCGGCGGCATCAGTCAGCCCCTTCAAG       G       A TG    CG   TG   CC    CT        TT
                                                                                                                     CCCCCCT CACGCT T  CCGG  GAC  CAG  CCGC  GCACGGTG  C
                                                                                                                     GGGGGGA GTGCGA A  GGTT  TTG  GTC  GGCG  TGTGTCGC  /
--------------------------------------------------------------------------------------------------------------------G       A      C GA    AT   --   C-    TT        GG

-----------------------------------------------------------------------------------------------------------------------------------------GGTTATTTG--GTCC-GGCGTTT----------      0.340      0.152      0.152
----------------------------------------------------------------------------------------------------------------------------------------AGGTTATTTG--GTCC-GGCGTTT----------      0.243      0.038      0.038
---------------------------------------------------------------------------------------------------------------------------------------GAGGTTATTTG--GTCC-GGCGTTT----------      0.000      0.152      0.152
----------------------------------------------------------------------------------------------------------------------------------------AGGTTATTTG--GTCC-GGCGTT-----------      0.049      0.114      0.075
---------------------------------------------------------------------------------------------------------------------------------------GAGGTTATTTG--GTCC-GGCGTT-----------      12.628     3.334      0.715
------------------------------------------------------------------------------------------------------------------------------------ACAGAGGTTATTTG--GTCC-GGCGTT-----------      0.000      0.076      0.076
-------------------------------------------------------------------------------------------------------------------------------------CAGAGGTTATTTG--GTCC-GGCGTT-----------      0.097      0.076      0.076
--------------------------------------------------------------------------------------------------------------------------------------AGAGGTTATTTG--GTCC-GGCGTT-----------      10.005     7.274      0.377
----------------------------------------------------------------------------------------------------------------------------------CGACAGAGGTTATTTG--GTCC-GGCGTT-----------      0.000      0.076      0.076
-----------------------------------------------------------------------------------------------------------------------------------------GGTTATTTG--GTCC-GGCGTT-----------      0.097      0.076      0.076
-------------------------------------------------------------------------------------------------------------------------------------CAGAGGTTATTTG--GTCC-GGCGT------------      2.331      0.606      0.606
-----------------------------------------------------------------------------------------------------------------------------------------GGTTATTTG--GTCC-GGCGT------------      0.097      0.227      0.188
----------------------------------------------------------------------------------------------------------------------------------------AGGTTATTTG--GTCC-GGCGT------------      0.146      0.265      0.075
------------------------------------------------------------------------------------------------------------------------------------ACAGAGGTTATTTG--GTCC-GGCGT------------      0.097      0.379      0.379
---------------------------------------------------------------------------------------------------------------------------------------GAGGTTATTTG--GTCC-GGCGT------------      8.014      3.978      2.372
--------------------------------------------------------------------------------------------------------------------------------------AGAGGTTATTTG--GTCC-GGCGT------------   1625.237    462.172    143.374 miR_t74
--------------------------------------------------------------------------------------------------------------------------------------AGAGGTTATTTG--GTCC-GGCG-------------      17.630     3.145      1.544
--------------------------------------------------------------------------------------------------------------------------------------AGAGGTTATTTG--GTCC-G----------------      0.680      0.114      0.114
---------------------------------------------------------------------------------------------------------------------------GAAGTGCGACAGAGGTTATTT--------------------------      0.194      0.189      0.189
--------------------------------------------------------------------------------------------------------------------------GGAAGTGCGACAGAGGTTATTT--------------------------      0.049      0.038      0.188
--------------------------------------------------------------------------------------------------------------------------GGAAGTGCGACAGAGGTTATT---------------------------      1.117      1.553      3.727
-------------------------------------------------------------------------------------------------------------------------GGGAAGTGCGACAGAGGTTAT----------------------------      0.097      0.265      0.452
--------------------------------------------------------------------------------------------------------------------------GGAAGTGCGACAGAGGTTAT----------------------------      0.243      0.758      0.489

miRNA: miR_t92 chromosome_12 Start: 6166774 End: 6167181 Condition: HS+N
------------------------------------------------------------------------------------------------------------------------------------------------------------------------------CGGCTACGGGAGAATGG-------------------------------------------------------------------------------------------------      0.000      0.038      0.038
----------------------------------------------------------------------------------------------------------------------------------------------------------------------------------TACGGGAGAATGGCAAAGA-------------------------------------------------------------------------------------------      0.000      0.038      0.038
----------------------------------------------------------------------------------------------------------------------------------------------------------------------------------TACGGGAGAATGGCAAAGAG------------------------------------------------------------------------------------------      0.146      0.114      0.075
----------------------------------------------------------------------------------------------------------------------------------------------------------------------------------TACGGGAGAATGGCAAAG--------------------------------------------------------------------------------------------      0.000      0.076      0.113
----------------------------------------------------------------------------------------------------------------------------------------------------------------------------------TACGGGAGAATGGCAAAGAGG-----------------------------------------------------------------------------------------      3.448      3.637      5.911
-----------------------------------------------------------------------------------------------------------------------------------------------------------------------------------ACGGGAGAATGGCAAAGAGG-----------------------------------------------------------------------------------------      0.000      0.075      0.075
-----------------------------------------------------------------------------------------------------------------------------------------------------------------------------------------AATGGCAAAGAGGAAAGC-------------------------------------------------------------------------------------      0.000      0.075      0.075
-------------------------------------------------------------------------------------------------------------------------------------------------------------------------------------------TGGCAAAGAGGAAAGCTGAGC--------------------------------------------------------------------------------     82.469    170.146    153.465 miR_t92
--------------------------------------------------------------------------------------------------------------------------------------------------------------------------------------------GGCAAAGAGGAAAGCTGAGC--------------------------------------------------------------------------------      0.534      1.856      2.221
---------------------------------------------------------------------------------------------------------------------------------------------------------------------------------------------CAAAGAGGAAAGCTGAGCACG------------------------------------------------------------------------------      0.049      0.265      0.151
----------------------------------------------------------------------------------------------------------------------------------------------------------------------------------------------AAAGAGGAAAGCTGAGCACG------------------------------------------------------------------------------      0.146      0.038      0.038
-----------------------------------------------------------------------------------------------------------------------------------------------------------------------------------------------AAGAGGAAAGCTGAGCACGA-----------------------------------------------------------------------------      0.000      0.038      0.113
--------------------------------------------------------------------------------------------------------------------------------------------------------------------------------------------------------------------------TGCACCGGCAGCCAAGGCT---------------------------------------------------      0.583      0.076      0.038
--------------------------------------------------------------------------------------------------------------------------------------------------------------------------------------------------------------------------TGCACCGGCAGCCAAGGCTC--------------------------------------------------      1.846      0.114      0.038
--------------------------------------------------------------------------------------------------------------------------------------------------------------------------------------------------------------------------TGCACCGGCAGCCAAGGCTCG-------------------------------------------------      3.885      0.606      0.527
--------------------------------------------------------------------------------------------------------------------------------------------------------------------------------------------------------------------------------------AAGGCTCGTCGCCCGAATGGC-------------------------------------      0.000      0.076      0.339

------------------------------------------------------------------------------------------------------------------------------------------------------------------------   C                            T                                         G            G   A                 AC    AA
                                                                                                                                                                         AG CCGGCTACGGGAGAATGGCAAAGAGGAAAGC GAGCACGATGCCGAGGGTGCACCGGCAGCCAAGGCTCGTCGCCCC AATGGCTACTGGC TCC TTCAACACCTGTAAGCCTT  AGTT  A
                                                                                                                                                                         TC GGCCGATGCCCTCTTACCGTTTCTCCTTTCG CTCGTGCTACGGCTCCCACGTGGCCGTCGGTTCCGAGCAGCGGG TTACCGATGACCG AGG AAGTTGTGGACATTTGGAA  TCGG  /
CGCTGCTCGGAACCGACGGCCACGTGGGAGCCGTAGCAGGAGTCGAAAGGCGAAACCGTAAGAGGCTGTCCACTCGAAGCAGCGGACCGGCGCTTCTGGCACGGACGACCGGGACCAAAGCAACGATCCGCATAAGTACCGTACGTGTCCACGAGC   C                            C                                         G            A   C                 CC    AA

---------------------------------------------------------------------------------------------------------------------------------------------------------------------------------------------------------------------------------------------------------------GAAGGCAAGTTGTGGACATTT------------      1.700      0.493      0.828
--------------------------------------------------------------------------------------------------------------------------------------------------------------------------------------------------------------------------------------------------------------CGAAGGCAAGTTGTGGACATTT------------      0.000      0.038      0.038
-------------------------------------------------------------------------------------------------------------------------------------------------------------------------------------------------------------------------------------------------------------CCGAAGGCAAGTTGTGGACAT--------------      2.720      1.061      1.280
--------------------------------------------------------------------------------------------------------------------------------------------------------------------------------------------------------------------------------------------------------------CGAAGGCAAGTTGTGGACAT--------------      1.068      0.796      1.581
-------------------------------------------------------------------------------------------------------------------------------------------------------------------------------------------------------------------------------------------------------------CCGAAGGCAAGTTGTGGACA---------------      0.000      0.038      0.113
-------------------------------------------------------------------------------------------------------------------------------------------------------------------------------------------------------------------------------------------------------CCGATGACCGAAGGCAAGTTGT-------------------      1.797      0.493      0.226
--------------------------------------------------------------------------------------------------------------------------------------------------------------------------------------------------------------------------------------------------------CGATGACCGAAGGCAAGTTGT-------------------      4.080      2.576      4.518
---------------------------------------------------------------------------------------------------------------------------------------------------------------------------------------------------------------------------------------------------------GATGACCGAAGGCAAGTTGT-------------------      4.128      1.137      0.753
--------------------------------------------------------------------------------------------------------------------------------------------------------------------------------------------------------------------------------------------------------CGATGACCGAAGGCAAGTTG--------------------      0.146      0.075      0.075
--------------------------------------------------------------------------------------------------------------------------------------------------------------------------------------------------------------------------------------------------------CGATGACCGAAGGCAAGTT---------------------      0.000      0.075      0.075
------------------------------------------------------------------------------------------------------------------------------------------------------------------------------------------------------------------------------------------------------ACCGATGACCGAAGGCAAGTT---------------------     13.502      2.804      1.732
-------------------------------------------------------------------------------------------------------------------------------------------------------------------------------------------------------------------------------------------------------CCGATGACCGAAGGCAAGTT---------------------      1.311      2.197      1.130
-----------------------------------------------------------------------------------------------------------------------------------------------------------------------------------------------------------------------------------------------------TACCGATGACCGAAGGCAAGTT---------------------     11.074      1.856      1.506
----------------------------------------------------------------------------------------------------------------------------------------------------------------------------------------------------------------------------------------------------TTACCGATGACCGAAGGCAAGT----------------------      9.277      0.303      0.151
-----------------------------------------------------------------------------------------------------------------------------------------------------------------------------------------------------------------------------------------------------TACCGATGACCGAAGGCAAGT----------------------   1049.752     12.389     11.973
-------------------------------------------------------------------------------------------------------------------------------------------------------------------------------------------------------------------------------------------------------CCGATGACCGAAGGCAAGT----------------------      4.808      0.303      0.226
---------------------------------------------------------------------------------------------------------------------------------------------------------------------------------------------------------------------------------------------------------GATGACCGAAGGCAAGT----------------------      0.097      0.114      0.038
-----------------------------------------------------------------------------------------------------------------------------------------------------------------------------------------------------------------------------------------------------TACCGATGACCGAAGGCAA------------------------      0.000      0.038      0.038
-------------------------------------------------------------------------------------------------------------------------------------------------------------------------------------------------------------------------------------------------GGGTTACCGATGACCGAAGGC--------------------------      0.049      0.226      0.226
-----------------------------------------------------------------------------------------------------------------------------------------------------------------------------------------------------------------------------------CGGTTCCGAGCAGCGGGGTT-----------------------------------------      0.000      0.114      0.038
------------------------------------------------------------------------------------------------------------------------------------------------------------------------------------------------------------------------------------------------CGTCGGTTCCGAGCAGCGGGGT------------------------------------------     13.356      5.266      5.685
-----------------------------------------------------------------------------------------------------------------------------------------------------------------------------------------------------------------------------------------------CCGTCGGTTCCGAGCAGCGGGGT------------------------------------------      0.389      0.075      0.075
-------------------------------------------------------------------------------------------------------------------------------------------------------------------------------------------------------------------------------------------------GTCGGTTCCGAGCAGCGGGGT------------------------------------------      1.603      0.152      0.188
-----------------------------------------------------------------------------------------------------------------------------------------------------------------------------------------------------------------------------------CGGTTCCGAGCAGCGGGGT------------------------------------------      0.000      0.038      0.038
------------------------------------------------------------------------------------------------------------------------------------------------------------------------------------------------------------------------------------------------CGTCGGTTCCGAGCAGCGGGG-------------------------------------------      0.777      0.114      0.339
------------------------------------------------------------------------------------------------------------------------------------------------------------------------------------------------------------------------------------------------CGTCGGTTCCGAGCAGCGGG--------------------------------------------      2.234      1.440      1.544
-----------------------------------------------------------------------------------------------------------------------------------------------------------------------------------------------------------------------------------------------CCGTCGGTTCCGAGCAGCGGG--------------------------------------------      0.194      0.114      0.113
-------------------------------------------------------------------------------------------------------------------------------------------------------------------------------------------------------------------------------------------------GTCGGTTCCGAGCAGCGGG--------------------------------------------      0.000      0.038      0.038
-----------------------------------------------------------------------------------------------------------------------------------------------------------------------------------------------------------TCGTGCTACGGCTCCCACGT-----------------------------------------------------------------      0.389      0.038      0.038
------------------------------------------------------------------------------------------------------------------------------------------------------------------------------------------------------------CGTGCTACGGCTCCCACGT-----------------------------------------------------------------      3.400      2.084      1.468
-------------------------------------------------------------------------------------------------------------------------------------------------------------------------------------------------------------GTGCTACGGCTCCCACGT-----------------------------------------------------------------      0.000      0.227      0.113
------------------------------------------------------------------------------------------------------------------------------------------------------------------------------------------------------------CGTGCTACGGCTCCCACG------------------------------------------------------------------      0.000      0.075      0.075
-----------------------------------------------------------------------------------------------------------------------------------------------------------------------------------------------TTCTCCTTTCGCCTCGTGCT-----------------------------------------------------------------------------      0.049      0.113      0.113
---------------------------------------------------------------------------------------------------------------------------------------------------------------------------------------------CGTTTCTCCTTTCGCCTCGT-------------------------------------------------------------------------------      1.603      0.152      0.151
--------------------------------------------------------------------------------------------------------------------------------------------------------------------------------------------CCGTTTCTCCTTTCGCCTCGT-------------------------------------------------------------------------------      5.828      0.796      0.301
-----------------------------------------------------------------------------------------------------------------------------------------------------------------------------------------TTACCGTTTCTCCTTTCGCCT----------------------------------------------------------------------------------      1.214      0.076      0.038 miR_t92*
------------------------------------------------------------------------------------------------------------------------------------------------------------------------------------CCCTCTTACCGTTTCTCCTTT---------------------------------------------------------------------------------------      0.194      0.038      0.038
-----------------------------------------------------------------------------------------------------------------------------------------------------------------------------------GCCCTCTTACCGTTTCTCCTTT---------------------------------------------------------------------------------------      0.097      0.038      0.075
-----------------------------------------------------------------------------------------------------------------------------------------------------------------------------------GCCCTCTTACCGTTTCTCCTT----------------------------------------------------------------------------------------      0.389      0.227      0.113
----------------------------------------------------------------------------------------------------------------------------------------------------------------------------------TGCCCTCTTACCGTTTCTCCT---------------------------------------------------------------------------------------------      0.583      0.038      0.038
-----------------------------------------------------------------------------------------------------------------------------------------------------------------------------------GCCCTCTTACCGTTTCTCCT---------------------------------------------------------------------------------------------      1.360      0.114      0.339
---------------------------------------------------------------------------------------------------------------------------------------------------------------------------------ATGCCCTCTTACCGTTTCTCCT---------------------------------------------------------------------------------------------      0.000      0.075      0.075
-------------------------------------------------------------------------------------------------------------------------------------------------------------------------------CGATGCCCTCTTACCGTTTCT------------------------------------------------------------------------------------------------      0.971      0.303      0.264
-----------------------------------------------------------------------------------------------------------------------------------------------------------------------------GCCGATGCCCTCTTACCGTTT--------------------------------------------------------------------------------------------------      0.049      0.264      0.264
----------------------------------------------------------------------------------------------------------------------------------------------------------------------------GGCCGATGCCCTCTTACCGTTT--------------------------------------------------------------------------------------------------      0.049      0.075      0.075
----------------------------------------------------------------------------------------------------------------------------------------------------------------------------GGCCGATGCCCTCTTACCGTT---------------------------------------------------------------------------------------------------      0.486      0.227      0.113
----------------------------------------------------------------------------------------------------------------------------------------------------------------------GAGCTCCGGCCGATGCCCTCT---------------------------------------------------------------------------------------------------------      1.166      0.038      0.038
---------------------------------------CGTGGGAGCCGTAGCAGGAGT------------------------------------------------------------------------------------------------------------------------------------------------------------------------------------------------------------------------------------      0.049      0.038      0.188
-----CTCGGAACCGACGGCCACGT--------------------------------------------------------------------------------------------------------------------------------------------------------------------------------------------------------------------------------------------------------------------------      1.846      0.114      0.038
------TCGGAACCGACGGCCACGT--------------------------------------------------------------------------------------------------------------------------------------------------------------------------------------------------------------------------------------------------------------------------      0.583      0.076      0.038
----GCTCGGAACCGACGGCCACGT--------------------------------------------------------------------------------------------------------------------------------------------------------------------------------------------------------------------------------------------------------------------------      3.885      0.606      0.527

miRNA: miR_t93 chromosome_12 Start: 4826917 End: 4827161 Condition: HS+N
---------------------------------------------------------TCCTCCAGCCATGCCAGCAG----------------------------------------------------      0.097      0.038      0.038
-------------------------------------------------------GCTCCTCCAGCCATGCCAGC------------------------------------------------------      0.049      0.075      0.075
-------------------------------------------------------TGCTCCTCCAGCCATGCCAGC-----------------------------------------------------      2.623      1.440      1.205 miR_t93*
-------------------------------------------------------TGCTCCTCCAGCCATGCCAG------------------------------------------------------      0.000      0.038      0.038
--------------------------------------------TGCCCTGACCCTGCTCCTCC-----------------------------------------------------------------      0.049      0.038      0.038
-------------------------------------------TTGCCCTGACCCTGCTCCTCC-----------------------------------------------------------------      0.291      0.303      0.301
------------------------------------TCAAGCGCTTGCCCTGACCC-------------------------------------------------------------------------      0.049      0.075      0.075
-----------------------------------TTCAAGCGCTTGCCCTGACCC-------------------------------------------------------------------------      0.534      0.303      0.489
----------------TACTGGACTGCTTTGGC------------------------------------------------------------------------------------------------      0.097      0.796      0.264
----------------TACTGGACTGCTTTGGCC-----------------------------------------------------------------------------------------------      0.146      0.417      0.038
--------------AGTACTGGACTGCTTTGGCC-----------------------------------------------------------------------------------------------      0.000      0.038      0.038
----------TGCAAGTACTGGACTGCTTTGGC------------------------------------------------------------------------------------------------      0.729      9.661      0.151
----------TGCAAGTACTGGACTGCTTTGG-------------------------------------------------------------------------------------------------      0.534      1.288      0.151
-------CGCCTGCAAGTACTGGACTGC-----------------------------------------------------------------------------------------------------      0.000      0.075      0.075
----TAGCGCCTGCAAGTACTGGAC--------------------------------------------------------------------------------------------------------      0.000      0.038      0.038

AGTCTAGCGCCT      A                                                                      GCCGCAGGTGGCAGCGCGGGCAGCGGCAG       CC  AG
           GCAAGT CTGGACTGCTTTGGCCCAGTTCAAGCGCTTGCCCTGACCCTGCTCCTCCAGCCATGCCAGCAGTGCAGACTTGGTCT                             GGCCAAGG  GC  G
           CGTTCA GACTTGACGAAACCGGGTCAAGTTCGCGAACGGGACTGGGACGAGGAGGTCGGTACGGTCGTCACGTCTGAACCAGA                             CTGGTTTC  CG  A
-----------      C                                                                       AGACA-------------------------       CA  AA

---------------CACGACTTGACGAAACCGGG----------------------------------------------------------------------------------------------      0.000      0.075      0.075
---------------CACGACTTGACGAAACCGGGT---------------------------------------------------------------------------------------------      4.323      0.189      0.414
----------------------GACGAAACCGGGTCAAGTT----------------------------------------------------------------------------------------      0.049      0.038      0.038
------------------------------GGGTCAAGTTCGCGAACGGGA------------------------------------------------------------------------------      0.000      0.265      0.301
---------------------------------TCAAGTTCGCGAACGGGACT----------------------------------------------------------------------------      0.097      0.341      0.188
--------------------------------GTCAAGTTCGCGAACGGGACT----------------------------------------------------------------------------      1.894      0.417      0.075
----------------------------------CAAGTTCGCGAACGGGACT----------------------------------------------------------------------------      0.049      0.379      0.075
-------------------------------GGTCAAGTTCGCGAACGGGACT----------------------------------------------------------------------------      0.680      0.833      0.377
---------------------------------------------CGGGACTGGGACGAGGAGGT----------------------------------------------------------------      0.097      0.151      0.151
--------------------------------------------ACGGGACTGGGACGAGGAGGT----------------------------------------------------------------      0.389      0.076      0.188
--------------------------------------------------CTGGGACGAGGAGGTCGG-------------------------------------------------------------      0.000      0.038      0.038
-------------------------------------------------ACTGGGACGAGGAGGTCGGT------------------------------------------------------------      0.049      0.076      0.113
----------------------------------------------------GGGACGAGGAGGTCGGT------------------------------------------------------------      0.000      0.038      0.038
--------------------------------------------------CTGGGACGAGGAGGTCGGT------------------------------------------------------------      0.049      0.038      0.075
-----------------------------------------------GGACTGGGACGAGGAGGTCGGT------------------------------------------------------------      0.534      0.303      0.602
-----------------------------------------------------GGACGAGGAGGTCGGTA-----------------------------------------------------------      0.000      0.038      0.038
-----------------------------------------------------GGACGAGGAGGTCGGTACGG--------------------------------------------------------      0.389      0.833      3.502
----------------------------------------------------GGGACGAGGAGGTCGGTACGG--------------------------------------------------------      0.049      0.076      0.301
-----------------------------------------------------GGACGAGGAGGTCGGTACGGT-------------------------------------------------------     63.916    131.162    282.231 miR_t93
---------------------------------------------------TGGGACGAGGAGGTCGGTACGGT-------------------------------------------------------      0.146      0.076      0.075
```

```
-----------------------------------------------------------ACGAGGAGGTCGGTACGGT--------------------------------------------------------------      0.000      0.113      0.113
----------------------------------------------------------GACGAGGAGGTCGGTACGGT--------------------------------------------------------------      0.340      0.227      0.678
--------------------------------------------------------GGGACGAGGAGGTCGGTACGGT--------------------------------------------------------------      3.545     14.700     20.896
------------------------------------------------------------CGAGGAGGTCGGTACGGT--------------------------------------------------------------      0.049      0.303      0.075
---------------------------------------------------------GGACGAGGAGGTCGGTACGGTC-------------------------------------------------------------      0.000      0.076      0.038
------------------------------------------------------------CGAGGAGGTCGGTACGGTCG------------------------------------------------------------      0.000      0.038      0.038
------------------------------------------------------------CGAGGAGGTCGGTACGGTCGT-----------------------------------------------------------      1.506      0.871      1.845
---------------------------------------------------------------------CGGTACGGTCGTCACGTCT----------------------------------------------------      0.194      0.076      0.113

miRNA: miR_t33,miR_t96 chromosome_17 Start: 1972737 End: 1972997 Condition: HS+N
---------------------------------------------------------------------------------------------------------------CTCCCCCGCAG-CAAGCAC------      0.000      0.038      0.038
--------------------------------------------------------------------------------------------------------TCCGCGACTCCCCCGCAG-CAAG--------      0.000      0.075      0.075
--------------------------------------------------------------------------------------------------------TCCGCGACTCCCCCGCAG-CAAGCAC-----      0.097      0.871      0.339
--------------------------------------------------------------------------------------------------------TCCGCGACTCCCCCGCAG-CAAGC-------      0.146      0.189      0.489
--------------------------------------------------------------------------------------------------------TCCGCGACTCCCCCGCAG-CAAGCA------      0.097      0.038      0.038
-------------------------------------------------------------------------------TGCCCGGGCCCGCAACGCGC------------------------------------      0.049      0.189      0.075
----------------------------------------------------------TGACTGACA------GCAGGGTGCCC---------------------------------------------------      0.097      0.341      0.188
----------------------------------------------------------TGACTGACA------GCAGGGTGCCCG--------------------------------------------------      0.243      0.152      0.075
---------------------------------------------------------ATGACTGACA------GCAGGGTGC-----------------------------------------------------      0.000      0.226      0.226
---------------------------------------------------------ATGACTGACA------GCAGGGTGCC----------------------------------------------------      0.243      1.478      0.791
--------------------------------------------------------TATGACTGACA------GCAGGGTGCC----------------------------------------------------     31.764    316.652    121.047 miR_t33
--------------------------------------------------------TATGACTGACA------GCAGGGTGC-----------------------------------------------------      0.631     31.521     14.721
--------------------------------------------------------TATGACTGACA------GCAGGGT-------------------------------------------------------      0.000      0.379      0.264
--------------------------------------------------------TATGACTGACA------GCAGGGTGCCC---------------------------------------------------      0.194      1.894      0.301
--------------------------------------------------------TATGACTGACA------GCAGGGTG------------------------------------------------------      0.000      0.985      0.715
-------------------------------------------------------CTATGACTGACA------GCAGGGTGCC----------------------------------------------------      0.000      0.227      0.188
-----------------------------TCACCAGCCACGCAGAAGCA--------------------------------------------------------------------------------------      0.049      0.038      0.075
-----------------------------TCACCAGCCACGCAGAAGCAT-------------------------------------------------------------------------------------      0.631      0.038      0.075
----------------------------TTCACCAGCCACGCAGAAGCA--------------------------------------------------------------------------------------      0.874      0.265      0.452
----------------------------TTCACCAGCCACGCAGAAG----------------------------------------------------------------------------------------      0.049      0.038      0.038
----------------------------TTCACCAGCCACGCAGAAGC---------------------------------------------------------------------------------------      0.194      0.530      0.489
-----------------------ACAAATTCACCAGCCACGCAG-------------------------------------------------------------------------------------------      0.097      0.189      0.188
----------------------TACAAATTCACCAGCCAC-----------------------------------------------------------------------------------------------      0.000      0.038      0.038
----------------------TACAAATTCACCAGCCACGC---------------------------------------------------------------------------------------------      0.631      0.227      0.678
------------TCCGCTCTACTACAAATTCACC-----------------------------------------------------------------------------------------------------      0.049      0.038      0.188
-------TTGTCTCCGCTCTACTACAAA-----------------------------------------------------------------------------------------------------------      0.049      0.038      0.038

---CTTTT         T                                                 ------                  C                      C  - AA       G
        TGTCTCCGC CTACTACAAATTCACCAGCCACGCAGAAGCATGCCTGCTATGACTGACA      GCAGGGTGCCCGGGCCCGCAACG GCAGCGCTCCGCGACTCCCCCG AG C  GCACCAC C
        ACAGAGGCG GATGATGTTTAAGTGGTCGGTGCGTCTTCGTACGGACGATACTGACTGT      CGTTCCACGGGCTTGGGCGTTGC CGTCGCGAGGCGCTGAGGGGGC TC G  CGTGGTG C
CCGGTAAT         T                                                 GACTGT                  C                      A  T GC       C

----TAATACAGAGGCGTGATGAT----------------------------------------------------------------------------------------------------------------      0.000      0.114      0.038
----TAATACAGAGGCGTGATGATGT--------------------------------------------------------------------------------------------------------------      0.000      0.075      0.075
-----AATACAGAGGCGTGATGATGT--------------------------------------------------------------------------------------------------------------      0.146      0.076      0.151
--------ACAGAGGCGTGATGATGTT-------------------------------------------------------------------------------------------------------------      0.000      0.038      0.038
--------ACAGAGGCGTGATGATGTTT------------------------------------------------------------------------------------------------------------      0.389      1.137      1.845
-------TACAGAGGCGTGATGATGTTT------------------------------------------------------------------------------------------------------------      0.826      0.455      1.355
------------AGGCGTGATGATGTTTAAG---------------------------------------------------------------------------------------------------------      0.049      0.038      0.038
-----------GAGGCGTGATGATGTTTAAG---------------------------------------------------------------------------------------------------------      0.291      0.341      0.715
--------------GCGTGATGATGTTTAAGT--------------------------------------------------------------------------------------------------------      0.049      0.189      0.151
------------AGGCGTGATGATGTTTAAGT--------------------------------------------------------------------------------------------------------      4.468      3.523      5.083
-------------GGCGTGATGATGTTTAAGT--------------------------------------------------------------------------------------------------------      9.665     11.139      6.815
-----------GAGGCGTGATGATGTTTAAGT--------------------------------------------------------------------------------------------------------     43.274     18.072     22.327
---------------CGTGATGATGTTTAAGT--------------------------------------------------------------------------------------------------------      0.000      0.303      0.339
----------AGAGGCGTGATGATGTTTAAGT--------------------------------------------------------------------------------------------------------     16.659      6.668      4.104
-------------GGCGTGATGATGTTTAAGTGG------------------------------------------------------------------------------------------------------      0.000      0.038      0.188
---------------CGTGATGATGTTTAAGTGGT-----------------------------------------------------------------------------------------------------      7.188      6.554      2.937
--------------GCGTGATGATGTTTAAGTGGT-----------------------------------------------------------------------------------------------------      6.265      3.789      0.979
-------------GGCGTGATGATGTTTAAGTGGT-----------------------------------------------------------------------------------------------------      6.071      7.160      2.899
------------------GATGATGTTTAAGTGGTC----------------------------------------------------------------------------------------------------      0.000      0.152      0.151
------------------GATGATGTTTAAGTGGTCGG--------------------------------------------------------------------------------------------------      4.080      2.690      1.092
--------------------TGATGTTTAAGTGGTCGG--------------------------------------------------------------------------------------------------      0.097      0.227      0.075
-----------------TGATGATGTTTAAGTGGTCGG--------------------------------------------------------------------------------------------------      0.000      0.227      0.075
-------------------ATGATGTTTAAGTGGTCGGT-------------------------------------------------------------------------------------------------      2.428      4.471      2.259
--------------------TGATGTTTAAGTGGTCGGT-------------------------------------------------------------------------------------------------      2.768     16.064      5.685
---------------------GATGTTTAAGTGGTCGGT-------------------------------------------------------------------------------------------------      0.437      3.145      0.866
-----------------TGATGATGTTTAAGTGGTCGGT-------------------------------------------------------------------------------------------------     12.191     16.329      2.560
------------------GATGATGTTTAAGTGGTCGGT-------------------------------------------------------------------------------------------------    351.391    389.393    162.764 miR_t96
------------------GATGATGTTTAAGTGGTCGGTG------------------------------------------------------------------------------------------------      0.000      0.038      0.075
----------------------ATGTTTAAGTGGTCGGTGCGT---------------------------------------------------------------------------------------------      0.971      0.227      0.791
---------------------GATGTTTAAGTGGTCGGTGCGT---------------------------------------------------------------------------------------------      4.371      3.296      2.673
------------------------GTTTAAGTGGTCGGTGCGT---------------------------------------------------------------------------------------------      0.049      0.076      0.188
-----------------------TGTTTAAGTGGTCGGTGCGT---------------------------------------------------------------------------------------------      3.788      3.410      4.480
------------------------GTTTAAGTGGTCGGTGCGTCT-------------------------------------------------------------------------------------------      0.534      0.076      0.527
-------------------------TTTAAGTGGTCGGTGCGTCTT------------------------------------------------------------------------------------------      0.000      0.076      0.151
------------------------GTTTAAGTGGTCGGTGCGTCTT------------------------------------------------------------------------------------------      0.146      0.114      0.151
--------------------------TTAAGTGGTCGGTGCGTCTT------------------------------------------------------------------------------------------      0.000      0.227      0.075
------------------------------GTGGTCGGTGCGTCTTCGT---------------------------------------------------------------------------------------      0.049      0.038      0.038
----------------------------AAGTGGTCGGTGCGTCTTCGT---------------------------------------------------------------------------------------      0.243      0.114      0.151
-----------------------------AGTGGTCGGTGCGTCTTCGT---------------------------------------------------------------------------------------      0.243      0.189      0.075
----------------------------------------CGTCTTCGTACGGACGATACT---------------------------------------------------------------------------      0.000      0.189      0.075
---------------------------------------------TCGTACGGACGATACTGACT-----------------------------------------------------------------------      0.000      0.038      0.038
-------------------------------------------------------------------------CGTTCCACGGGCTTGGGCGT------------------------------------------      0.000      0.075      0.075
-------------------------------------------------------------------------CGTTCCACGGGCTTGGGCGTT-----------------------------------------      0.971      0.833      1.205
------------------------------------------------------------------------------------------CGTTGCCCGTCGCGAGGCGCT------------------------      0.194      0.114      0.113
----------------------------------------------------------------------------------------------------CGCGAGGCGCTGAGGGGGCAT--------------      0.583      0.379      1.393
------------------------------------------------------------------------------------------------------CGAGGCGCTGAGGGGGCATCT------------      0.389      0.114      0.264
------------------------------------------------------------------------------------------------------------GCTGAGGGGGCATCTGGCCGT------      6.557      1.174      2.334
------------------------------------------------------------------------------------------------------CGAGGCGCTGAGGGGGCATCTGGCCGT------      1.020      5.607      0.151
--------------------------------------------------------------------------------------------------------AGGCGCTGAGGGGGCATCTGGCCGT------      0.243      0.076      0.188
-------------------------------------------------------------------------------------------------------GAGGCGCTGAGGGGGCATCTGGCCGT------      0.097      0.038      0.038
-------------------------------------------------------------------------------------------------------------CTGAGGGGGCATCTGGCCGT------      0.049      0.076      0.075
-----------------------------------------------------------------------------------------------------------CGCTGAGGGGGCATCTGGCCGT------      0.291      1.478      1.318
--------------------------------------------------------------------------------------------------------------TGAGGGGGCATCTGGCCGTGGT---      0.097      0.152      0.113
---------------------------------------------------------------------------------------------------------------GAGGGGGCATCTGGCCGTGGT---      0.097      0.265      0.188

miRNA: miR_t98,miR_t20 chromosome_6 Start: 6776059 End: 6776276 Condition: HS+N
---------------------------------------------------------------------------------------------TCGAGCTCTACCCGAACGTCG------------      1.360      0.038      0.075
------------------------------------------------------------------------------------------CCTTCGAGCTCTACCCG-------------------      0.000      0.114      0.038
---------------------------------------------------------------------------------------AGACCTTCGAGCTCTACCCG-------------------      0.340      0.644      0.753
--------------------------------------------------------------------------------------AAGACCTTCGAGCTCTACCC--------------------      0.000      2.576      0.979
--------------------------------------------------------------------------------------AAGACCTTCGAGCTCTACCCG-------------------      9.665     51.070     37.726
--------------------------------------------------------------------------------------AAGACCTTCGAGCTCTACCCGA------------------      0.049      0.113      0.113
-------------------------------------------------------------------------------------CAAGACCTTCGAGCTCTACC---------------------      0.146      0.341      0.151
------------------------------------------------------------------------------------CCAAGACCTTCGAGCTCTACC---------------------      3.594      7.198      4.066
------------------------------------------------------------------------------------CCAAGACCTTCGAGCTC-------------------------      0.000      0.038      0.038
------------------------------------------------------------------------------------CCAAGACCTTCGAGCTCTA-----------------------      0.194      0.114      0.151
------------------------------------------------------------------------------------CCAAGACCTTCGAGCTCTAC----------------------      1.846      0.568      1.393
-----------------------------------------------------------------------------------CCCAAGACCTTCGAGCTCTACC---------------------      1.020      6.895      3.426
-----------------------------------------------------------------------------------CCCAAGACCTTCGAGCTCTAC----------------------      0.049      0.114      0.075
----------------------------------------------------------------------------------TCCCAAGACCTTCGAGCTCTACCC--------------------      1.214      5.759      0.151
----------------------------------------------------------------------------------TCCCAAGACCTTCGAGCTCTA-----------------------      1.166      0.909      1.694
----------------------------------------------------------------------------------TCCCAAGACCTTCGAGCTCT------------------------      0.291      0.682      0.339
----------------------------------------------------------------------------------TCCCAAGACCTTCGAGCTCTAC----------------------     24.381     49.631     62.124
----------------------------------------------------------------------------------TCCCAAGACCTTCGAGCTCTACC---------------------     25.595     61.603     63.517
---------------------------------------------------------------------------------TTCCCAAGACCTTCGAGCTCTACC---------------------    232.496    322.562    225.190
---------------------------------------------------------------------------------TTCCCAAGACCTTCGAGCTCTACCCG-------------------      0.146      0.682      0.301
---------------------------------------------------------------------------------TTCCCAAGACCTTCGAGCTCT------------------------     12.919     18.185     13.291
---------------------------------------------------------------------------------TTCCCAAGACCTTCGAGCTC-------------------------      1.020      0.114      0.301
---------------------------------------------------------------------------------TTCCCAAGACCTTCGAGCTCTAC----------------------    276.256    199.584    226.620
---------------------------------------------------------------------------------TTCCCAAGACCTTCGAGCTCTACCC--------------------      3.303      4.698      0.527
---------------------------------------------------------------------------------TTCCCAAGACCTTCGAGCTCTA-----------------------     28.704     42.281     62.877
--------------------------------------------------------------------------------CTTCCCAAGACCTTCGAGCTCTAC----------------------      0.146      0.151      0.151
--------------------------------------------------------------------------------CTTCCCAAGACCTTCGAGCTCTA-----------------------      0.000      0.264      0.264
--------------------------------------------------------------------------------CTTCCCAAGACCTTCGAGCTCTACC---------------------      0.049      0.379      0.151
------------------------------------------------------------------------------TCCTTCCCAAGACCTTCGAGC---------------------------      0.097      0.076      0.151
---------------------------------------------------------------------TGACCGTCCTCCTTCCCAAGA------------------------------------      1.603      0.341      0.828
---------------------------------------------------------------------TGACCGTCCTCCTTCCCAAG-------------------------------------      6.654      2.387      4.029
---------------------------------------------------------------------TGACCGTCCTCCTTCCC----------------------------------------      0.049      0.076      0.075
-----------------------------------------------------------------GGCGTGACCGTCCTCCTTCCC----------------------------------------      0.049      0.038      0.038
---------------------------------------------------------------TGGGCGTGACCGTCCTCCTTC------------------------------------------      8.548      2.311      2.861
---------------------------------------------------------------TGGGCGTGACCGTCCTCCTTCC-----------------------------------------      0.389      0.038      0.188
---------------------------------------------------------------TGGGCGTGACCGTCCTCCTTCCC----------------------------------------      0.049      0.038      0.038
--------------------------------------------------------------GTGGGCGTGACCGTCCTCC---------------------------------------------      0.049      0.075      0.075
-------------------------------------------------------------TGTGGGCGTGACCGTCCTCCT--------------------------------------------      0.000      0.076      0.113
-----------------------------------------------------------GTTGTGGGCGTGACCGTCC------------------------------------------------      0.000      0.038      0.038
----------------------------------------------------------TGTTGTGGGCGTGACCGTCC------------------------------------------------      0.146      0.076      0.264
----------------------------------------------------------TGTTGTGGGCGTGACCGTC-------------------------------------------------      0.194      0.227      0.565
----------------------------------------------GACCGACCCCGGTGTTGTGG------------------------------------------------------------      0.291      0.038      0.038
---------------------------------------------TGACCGACCCCGGTGTTG---------------------------------------------------------------      0.049      0.114      0.113
---------------------------------------------TGACCGACCCCGGTGTTGTG-------------------------------------------------------------      1.214      0.303      0.828
---------------------------------------------TGACCGACCCCGGTGTTGTGG------------------------------------------------------------     27.781      3.182      2.297
---------------------------------------------TGACCGACCCCGGTGTTGTGGG-----------------------------------------------------------      0.097      0.038      0.038
--------------------------------------------ATGACCGACCCCGGTGTTGTG-------------------------------------------------------------      0.194      0.227      0.489
------------------------------------------ACATGACCGACCCCGGTGTTG---------------------------------------------------------------      0.194      0.379      0.715
---------------------------------------TGAACATGACCGACCCCGGTG------------------------------------------------------------------      0.194      0.075      0.075
--------------------------------------TTGAACATGACCGACCCCGGTG------------------------------------------------------------------      0.049      0.264      0.264

----------------------------------CGCCTTGA                                               AC                                  C
                                          ACATGACCGACCCCGGTGTTGTGGGCGTGACCGTCCTCCTTCCCAAG  CTTCGAGCTCTACCCGAACGTCGAGTTCAAGTA G
                                          TGTACTGGCTGGGGCCACAACACCCGCACTGGCAGGAGGAAGGGTTC  GAAGCTCGAGATGGGCTTGCAGCTCAAGTTCAT /
TTGCGGCCCGAGAGGTGTCGTTTATGCCCAGGTTATACCTCT                                               AA                                  -

----GGCCCGAGAGGTGTCGTT--------------------------------------------------------------------------------------------------------      0.049      0.038      0.038
------------------------------------------TGTACTGGCTGGGGCCACAAC---------------------------------------------------------------      0.000      0.038      0.038
--------------------------------------------------------------CACCCGCACTGGCAGGAGGA---------------------------------------------      0.000      0.075      0.075
---------------------------------------------------------------ACCCGCACTGGCAGGAGGAA--------------------------------------------      0.000      0.038      0.038
----------------------------------------------------------------CCCGCACTGGCAGGAGGAAGG------------------------------------------      0.000      0.303      0.866
-------------------------------------------------------------------GCACTGGCAGGAGGAAGGG----------------------------------------      0.049      0.038      0.038
------------------------------------------------------------------CGCACTGGCAGGAGGAAGGG----------------------------------------      0.874      0.417      2.410
-------------------------------------------------------------------GCACTGGCAGGAGGAAGGGT---------------------------------------      1.894      2.311      3.502
------------------------------------------------------------------CGCACTGGCAGGAGGAAGGGT---------------------------------------     69.064     65.998    118.901 miR_t98
----------------------------------------------------------------------CTGGCAGGAGGAAGGGT---------------------------------------      0.097      0.038      0.038
--------------------------------------------------------------------CACTGGCAGGAGGAAGGGT---------------------------------------      0.000      0.038      0.038
-----------------------------------------------------------------CCGCACTGGCAGGAGGAAGGGT---------------------------------------      0.680      0.114      0.715
```

```
-------------------------------------------------------------------GCACTGGCAGGAGGAAGGGTT--------------------------------------      9.374      7.805     11.295
---------------------------------------------------------------------ACTGGCAGGAGGAAGGGTT--------------------------------------      0.049      0.038      0.038
---------------------------------------------------------------------ACTGGCAGGAGGAAGGGTTCA------------------------------------      0.146      0.038      0.038
----------------------------------------------------------------------CTGGCAGGAGGAAGGGTTCAA-----------------------------------      0.000      0.076      0.038
---------------------------------------------------------------------ACTGGCAGGAGGAAGGGTTCAA-----------------------------------      0.777      0.417      0.527
------------------------------------------------------------------------GGCAGGAGGAAGGGTTCAAGA---------------------------------      0.000      0.038      0.038
-------------------------------------------------------------------------GCAGGAGGAAGGGTTCAAGAA--------------------------------      0.146      0.339      0.339
----------------------------------------------------------------------------GGAGGAAGGGTTCAAGAAG-------------------------------      0.631      2.235      1.054
----------------------------------------------------------------------------GGAGGAAGGGTTCAAGAAGC------------------------------      0.729      1.667      1.017
--------------------------------------------------------------------------------GAAGGGTTCAAGAAGCT-----------------------------      0.243      0.076      0.113
---------------------------------------------------------------------------AGGAGGAAGGGTTCAAGAAGCT-----------------------------      0.534      1.099      0.038
----------------------------------------------------------------------------GGAGGAAGGGTTCAAGAAGCT-----------------------------    114.038    333.473    250.227
-----------------------------------------------------------------------------GAGGAAGGGTTCAAGAAGCT-----------------------------      0.146      0.720      0.565
-------------------------------------------------------------------------------GGAAGGGTTCAAGAAGCT-----------------------------      1.797      0.909      1.318
------------------------------------------------------------------------------AGGAAGGGTTCAAGAAGCT-----------------------------      0.000      0.226      0.226
----------------------------------------------------------------------------GGAGGAAGGGTTCAAGAAGCTC----------------------------      0.000      0.076      0.151
--------------------------------------------------------------------------------GAAGGGTTCAAGAAGCTCGA--------------------------      0.437      0.227      0.264
-------------------------------------------------------------------------------GGAAGGGTTCAAGAAGCTCGA--------------------------      3.545      1.932      0.038
-------------------------------------------------------------------------------GGAAGGGTTCAAGAAGCTCGAG-------------------------     15.493      8.562      0.565
---------------------------------------------------------------------------------AAGGGTTCAAGAAGCTCGAG-------------------------      0.146      0.075      0.075
----------------------------------------------------------------------------------AGGGTTCAAGAAGCTCGAG-------------------------     20.301      3.751      1.506
------------------------------------------------------------------------------------GGTTCAAGAAGCTCGAG-------------------------      0.049      0.038      0.264
--------------------------------------------------------------------------------GAAGGGTTCAAGAAGCTCGAG-------------------------      4.177      4.433      0.188
-----------------------------------------------------------------------------------GGGTTCAAGAAGCTCGAG-------------------------      0.729      0.606      2.485
--------------------------------------------------------------------------------GAAGGGTTCAAGAAGCTCGAGA------------------------     37.349     70.771     10.053
-------------------------------------------------------------------------------GGAAGGGTTCAAGAAGCTCGAGA------------------------    239.538    267.930      7.417
----------------------------------------------------------------------------------AGGGTTCAAGAAGCTCGAGA------------------------    169.114     51.487     18.976
------------------------------------------------------------------------------------GGTTCAAGAAGCTCGAGA------------------------      0.534      2.614      5.233
-----------------------------------------------------------------------------------GGGTTCAAGAAGCTCGAGA------------------------      6.848     17.162     30.422
-----------------------------------------------------------------------------------GGGTTCAAGAAGCTCGAGAT-----------------------    753.827   2008.796   2089.432
-------------------------------------------------------------------------------GGAAGGGTTCAAGAAGCTCGAGAT-----------------------  12983.972  15291.886    302.863 miR_t20
------------------------------------------------------------------------------AGGAAGGGTTCAAGAAGCTCGAGAT-----------------------     39.195    223.604      1.318
--------------------------------------------------------------------------------------TTCAAGAAGCTCGAGAT-----------------------      1.117      0.720      1.845
-------------------------------------------------------------------------GCAGGAGGAAGGGTTCAAGAAGCTCGAGAT-----------------------      0.000      0.796      0.489
--------------------------------------------------------------------------CAGGAGGAAGGGTTCAAGAAGCTCGAGAT-----------------------      0.000      0.379      0.188
----------------------------------------------------------------------------GGAGGAAGGGTTCAAGAAGCTCGAGAT-----------------------      0.097      2.804      0.075
-----------------------------------------------------------------------------GAGGAAGGGTTCAAGAAGCTCGAGAT-----------------------      0.631     12.540      0.941
-------------------------------------------------------------------------------------GTTCAAGAAGCTCGAGAT-----------------------      1.360      0.796      1.355
---------------------------------------------------------------------------------AAGGGTTCAAGAAGCTCGAGAT-----------------------     53.182     48.305      2.485
--------------------------------------------------------------------------------GAAGGGTTCAAGAAGCTCGAGAT-----------------------   5946.153  11810.306    112.350
----------------------------------------------------------------------------------AGGGTTCAAGAAGCTCGAGAT-----------------------  17626.944   7832.456   1552.757
------------------------------------------------------------------------------------GGTTCAAGAAGCTCGAGAT-----------------------     76.009    328.775    385.883
---------------------------------------------------------------------------AGGAGGAAGGGTTCAAGAAGCTCGAGAT-----------------------      0.000      0.189      0.188
-----------------------------------------------------------------------------------GGGTTCAAGAAGCTCGAGATG----------------------      0.000      0.113      0.113
------------------------------------------------------------------------------------GGTTCAAGAAGCTCGAGATGG---------------------      0.486      0.265      0.151
--------------------------------------------------------------------------------------TTCAAGAAGCTCGAGATGGG--------------------      0.000      0.038      0.038
---------------------------------------------------------------------------------------TCAAGAAGCTCGAGATGGGCT------------------      0.049      0.379      0.264
---------------------------------------------------------------------------------------TCAAGAAGCTCGAGATGGGCTT-----------------      0.389      0.796      0.226
----------------------------------------------------------------------------------------CAAGAAGCTCGAGATGGGCTT-----------------      0.389      0.682      0.264
----------------------------------------------------------------------------------------------GCTCGAGATGGGCTTGCAGCT-----------      0.243      0.038      0.151
-------------------------------------------------------------------------------------------------CGAGATGGGCTTGCAGCTCAA--------      0.000      0.038      0.038
----------------------------------------------------------------------------------------------------------CTTGCAGCTCAAGTTCAT--      0.000      0.038      0.038

miRNA: miR_t100 chromosome_6 Start: 7676978 End: 7677176 Condition: HS+N
-------------------------------------------------------------------------------GGGCTGTAATGACGGGACGC-----------------------      0.000      0.075      0.075
-------------------------------------------------------------------------------GGGCTGTAATGACGGGACG------------------------      4.274      6.933     11.709
-------------------------------------------------------------------------------GGGCTGTAATGACGGGAC-------------------------      0.534      0.455      0.828
------------------------------------------------------------------------------TGGGCTGTAATGACGGG---------------------------      0.000      0.341      0.264
------------------------------------------------------------------------------TGGGCTGTAATGACGGGACGCG----------------------      0.777      2.766      3.539
------------------------------------------------------------------------------TGGGCTGTAATGACGGGA--------------------------      0.000      0.227      0.188
------------------------------------------------------------------------------TGGGCTGTAATGACGGGACGC-----------------------      3.740     13.980     10.392
------------------------------------------------------------------------------TGGGCTGTAATGACGGGAC-------------------------     76.592    170.298    175.942
------------------------------------------------------------------------------TGGGCTGTAATGACGGGACG------------------------    816.771   2062.746   1865.975 miR_t100
-----------------------------------------------------------------------------GTGGGCTGTAATGACGGGACG------------------------      1.457      9.472      4.443
-----------------------------------------------------------------------------GTGGGCTGTAATGACGGGACGC-----------------------      0.000      0.113      0.113
-------------------------------------------------------------------------AAGGGTGGGCTGTAATGACGG----------------------------      0.000      0.152      0.113
------------------------------------------------------------------------AAAGGGTGGGCTGTAATG--------------------------------      0.000      0.038      0.038
-----------------------------------------------------------------------TAAAGGGTGGGCTGTAATGAC------------------------------      5.585      2.387      1.205
-----------------------------------------------------------------------TAAAGGGTGGGCTGTAAT---------------------------------      0.049      0.076      0.075
-----------------------------------------------------------------------TAAAGGGTGGGCTGTAATGA-------------------------------      0.389      0.227      0.188
-----------------------------------------------------------------------TAAAGGGTGGGCTGTAATG--------------------------------      2.137      1.023      0.979
----------------------------------------------------------------------ATAAAGGGTGGGCTGTAATG--------------------------------      0.097      0.038      0.113
-------------------------------------------------------------------GGGATAAAGGGTGGGCTGTAA----------------------------------      0.000      0.075      0.075
----------------------------------------------------------------TGCGGGATAAAGGGTGGGCTG-------------------------------------      0.243      0.038      0.075
--------------------------------------------------------------TATGCGGGATAAAGGGTGG-----------------------------------------      0.049      0.076      0.038
--------------------------------------------------------------TATGCGGGATAAAGGGTGGGC---------------------------------------      0.000      0.038      0.301
----------------------------------------------------------TCGGTATGCGGGATAAAGG---------------------------------------------      0.049      0.075      0.075
----------------------------------------------------------TCGGTATGCGGGATAAAGGG--------------------------------------------      2.380      5.759      3.953
---------------------------------------------------------ATCGGTATGCGGGATAAAGGG--------------------------------------------      0.000      0.189      0.113
-----------------------------------------------TGGGCACGGGATCGGTATGCG------------------------------------------------------      0.243      0.189      0.188
---------------------------------------------TTTGGGCACGGGATCGGTATGCG------------------------------------------------------      0.000      0.038      0.038

-----------------------------------------GGGGTT                                                              ----       GC
                                               TGGGCACGGGATCGGTATGCGGGATAAAGGGTGGGCTGTAATGACGGGACGCGCCGCTGGT    GGCGCCA  G
                                               ACCCGTGCCCTAGCCATACGCCCTATTTCCCACCCGACATTACTGCCCTGCGCGGCGACCG    CCGCGGT  /
GGCTCCTATTTCCCACCCGACATTACTGCCCTGCGCGGCGACCGTCG                                                             TCGA       GG

----CCTATTTCCCACCCGACATT--------------------------------------------------------------------------------------------------      0.243      0.265      0.264
-----------CCCACCCGACATTACTGCCCT------------------------------------------------------------------------------------------      1.846      0.606      0.339
----------TCCCACCCGACATTACTGCCCT------------------------------------------------------------------------------------------      0.000      0.038      0.038
------------------------------------------CGTCGACCCGTGCCCTAGCCAT----------------------------------------------------------      0.000      0.076      0.038
--------------------------------------------------CGTGCCCTAGCCATACGCCCT---------------------------------------------------      0.243      0.151      0.151
-----------------------------------------------------GCCCTAGCCATACGCCCTATT------------------------------------------------      0.534      0.341      0.151
----------------------------------------------------TGCCCTAGCCATACGCCCTATTT-----------------------------------------------      0.146      0.038      0.301
-----------------------------------------------------GCCCTAGCCATACGCCCTATTT-----------------------------------------------      6.508      2.197      2.071
------------------------------------------------------CCCTAGCCATACGCCCTATTT-----------------------------------------------      0.097      0.152      0.301
-----------------------------------------------------------------CGCCCTATTTCCCACCCGACAT-----------------------------------      0.194      0.265      0.075
------------------------------------------------------------------GCCCTATTTCCCACCCGACAT-----------------------------------      2.720      4.774      2.447
----------------------------------------------------------------ACGCCCTATTTCCCACCCGACAT-----------------------------------      0.049      0.152      0.075
--------------------------------------------------------------------CCTATTTCCCACCCGACATT----------------------------------      0.243      0.265      0.264
------------------------------------------------------------------GCCCTATTTCCCACCCGACATT----------------------------------      3.594      5.418      2.786
-------------------------------------------------------------------CCCTATTTCCCACCCGACATT----------------------------------      2.137      1.478      0.602
---------------------------------------------------------------------------CCCACCCGACATTACTGCCCT--------------------------      1.846      0.606      0.339
--------------------------------------------------------------------------TCCCACCCGACATTACTGCCCT--------------------------      0.000      0.038      0.038

miRNA: miR_t105 chromosome_7 Start: 632165 End: 632262 Condition: HS+N
-----------GTCAATGACAGTCCTGCAG----------------------      0.243      0.189      0.489
----------TGTCAATGACAGTCCTGCAGT---------------------      0.146      0.038      0.151
----------TGTCAATGACAGTCCTGCAG----------------------     33.221     25.384     42.470 miR_t105
---------GTGTCAATGACAGTCCTGCAG----------------------      0.826      0.152      0.377

-----         A                    -  AG-       TGA
     GTCAGTGTC ATGACAGTCCTGCAGTACAC AC   GCAGGCG   C
     TAGTCACAG TACTGTCAGGACGTCATGTG TG   CGTCCGC   C
AACAG         C                    C  CGA       CCC

----GTAGTCACAGCTACTGTCAGGA--------------------------      0.049      0.985      0.151
--------TCACAGCTACTGTCAGGACGT-----------------------      0.340      0.151      0.151
------------AGCTACTGTCAGGACGTCAT--------------------      0.097      0.038      0.038
-----------CAGCTACTGTCAGGACGTCAT--------------------      0.146      0.227      0.075

miRNA: miR_t110 chromosome_9 Start: 1866669 End: 1867112 Condition: HS+N
-------------------------------------------------------------------------------------------------------------------------------------------------------------------------TGAAGCCCAAGCCCAGAACTG-------------------------------------------------------------------------------      6.751      0.114      0.226
-------------------------------------------------------------------------------------------------------------------------------------------------------------------------TGAAGCCCAAGCCCAGAACTGT------------------------------------------------------------------------------      0.680      0.226      0.226
----------------------------------------------------------------------------------------------------------------------------------------------------------TGCGTCAACGCTGCGTGAAGC----------------------------------------------------------------------------------------------      0.049      0.038      0.038
----------------------------------------------------------------------------------------------------------------------------------------TGGGAATGCACGCCGCTATGC----------------------------------------------------------------------------------------------------------------      0.049      0.075      0.075
-------------------------------------------------------------------------------------------------------------------------AGAACGCAGCAAAGGTGGG---------------------------------------------------------------------------------------------------------------------------------      0.000      0.076      0.113
-------------------------------------------------------------------------------------------------------------------------AGAACGCAGCAAAGGTGG----------------------------------------------------------------------------------------------------------------------------------      0.146      0.188      0.188
------------------------------------------------------------------------------------------------------------------------CAGAACGCAGCAAAGGTGGG---------------------------------------------------------------------------------------------------------------------------------      0.000      0.114      0.075
------------------------------------------------------------------------------------------------------------------------CAGAACGCAGCAAAGGTGGGA--------------------------------------------------------------------------------------------------------------------------------      0.049      0.113      0.113
------------------------------------------------------------------------------------------------------------------------CAGAACGCAGCAAAGGTGG----------------------------------------------------------------------------------------------------------------------------------      0.000      0.038      0.038
-----------------------------------------------------------------------------------------------------------------------TCAGAACGCAGCAAAGGTGG----------------------------------------------------------------------------------------------------------------------------------      0.923      0.758      1.355
-----------------------------------------------------------------------------------------------------------------------TCAGAACGCAGCAAAGGTG-----------------------------------------------------------------------------------------------------------------------------------      0.777      0.114      0.075
-----------------------------------------------------------------------------------------------------------------------TCAGAACGCAGCAAAGGTGGG---------------------------------------------------------------------------------------------------------------------------------      9.859      8.221      6.702
----------------------------------------------------------------------------------------------------------------------TTCAGAACGCAGCAAAGGTGGG---------------------------------------------------------------------------------------------------------------------------------      1.408      2.046      0.264
----------------------------------------------------------------------------------------------------------------------TTCAGAACGCAGCAAAGGT------------------------------------------------------------------------------------------------------------------------------------      0.389      0.227      0.075
----------------------------------------------------------------------------------------------------------------------TTCAGAACGCAGCAAAGG-------------------------------------------------------------------------------------------------------------------------------------      0.000      0.038      0.038
----------------------------------------------------------------------------------------------------------------------TTCAGAACGCAGCAAAGGTG-----------------------------------------------------------------------------------------------------------------------------------      9.228      2.046      2.824
----------------------------------------------------------------------------------------------------------------------TTCAGAACGCAGCAAAGGTGG----------------------------------------------------------------------------------------------------------------------------------     20.253     27.733     27.560 miR_t110
--------------------------------------------------------------------------TTCCCTTGCGACTGCCTCAGC------------------------------------------------------------------------------------------------------------------------------------------------------------------------------      1.846      0.379      0.489
-------------------------------------------TGACATAGCAAAGAGAAAGC------------------------------------------------------------------------------------------------------------------------------------------------------------------------------------------------------------------      0.243      0.075      0.075
-------------------------------------------TGACATAGCAAAGAGAAAGCA-----------------------------------------------------------------------------------------------------------------------------------------------------------------------------------------------------------------      1.457      0.113      0.113
------------------------------------------TTGACATAGCAAAGAGAAAGC------------------------------------------------------------------------------------------------------------------------------------------------------------------------------------------------------------------      0.049      0.038      0.075

--------------------------------------                                                          G                                                                                                   TCGACAATGACGGTCAGAATAAGTCCGACGGTGCCCACGTCGAGCTGAGTTTTCG     -     TCT  CG
                                      CCACTTGACATAGCAAAGAGAAAGCAATAGCATGGCTTCCCTTGCGACTGCCTCAGCT CAACCCGCAGCCGCGGCTGCGTTCAGAACGCAGCAAAGGTGGGAATGCACGCCGCTATGCGTCAACGCTGCGTGAAGCCCAAGCCCAGAACTGTTCCGT                                                       GCGGG GGGA   GG  A
                                      GGTGAACTGTATCGTTTCTCTTTCGTTATCGTACCGAAGGGAACGCTGACGGAGTCGA GTTGGGCGTCGGCGCCGACGCAAGTCTTGCGTCGTTTCCACCCTTACGTGCGGCGATACGCAGTTGCGACGCACTTCGGGTTCGGGTCTTGACAAGGCA                                                       CGCCC CCCT   CC  /
ATTCCGACGTATGCAAGTCCGGGGTGTCGGGTGGGCAG                                                          A                                                                                                   CG-----------------------------------------------------     G     T--  AA

----CGACGTATGCAAGTCCGGGGT--------------------------------------------------------------------------------------------------------------------------------------------------------------------------------------------------------------------------------------------------------      0.000      0.076      0.075
-----------------------------GGTGGGCAGGGTGAACTGTAT-------------------------------------------------------------------------------------------------------------------------------------------------------------------------------------------------------------------------------------------      0.194      0.076      0.075
--------------------------------GGGCAGGGTGAACTGTATCGT------------------------------------------------------------------------------------------------------------------------------------------------------------------------------------------------------------------------------------------      0.146      0.152      0.151
--------------------------------GGGCAGGGTGAACTGTATCGTT-----------------------------------------------------------------------------------------------------------------------------------------------------------------------------------------------------------------------------------------      0.097      0.076      0.075
---------------------------------GGCAGGGTGAACTGTATCGTT-----------------------------------------------------------------------------------------------------------------------------------------------------------------------------------------------------------------------------------------      0.097      0.075      0.075
-------------------------------------------------------------------CGTACCGAAGGGAACGCTGAC---------------------------------------------------------------------------------------------------------------------------------------------------------------------------------------------------      0.000      0.076      0.188
------------------------------------------------------------------------CGAAGGGAACGCTGACGGAGT----------------------------------------------------------------------------------------------------------------------------------------------------------------------------------------------      0.680      0.303      2.071
-----------------------------------------------------------------------------------CTGACGGAGTCGAAGTTGGGC---------------------------------------------------------------------------------------------------------------------------------------------------------------------------------------      0.000      0.113      0.113
-------------------------------------------------------------------------------------GACGGAGTCGAAGTTGGGCGT-------------------------------------------------------------------------------------------------------------------------------------------------------------------------------------      0.194      0.076      0.301
---------------------------------------------------------------------------------------CGGAGTCGAAGTTGGGCGT-------------------------------------------------------------------------------------------------------------------------------------------------------------------------------------      0.000      0.038      0.038
------------------------------------------------------------------------------------TGACGGAGTCGAAGTTGGGCGT-------------------------------------------------------------------------------------------------------------------------------------------------------------------------------------      0.097      0.075      0.075
----------------------------------------------------------------------------------------------------------CGGCGCCGACGCAAGTCT-------------------------------------------------------------------------------------------------------------------------------------------------------------      0.000      0.038      0.038
--------------------------------------------------------------------------------------------------------GTCGGCGCCGACGCAAGTCTT------------------------------------------------------------------------------------------------------------------------------------------------------------      4.371      0.227      0.753
---------------------------------------------------------------------------------------------------------TCGGCGCCGACGCAAGTCTT------------------------------------------------------------------------------------------------------------------------------------------------------------      0.437      0.606      0.339
----------------------------------------------------------------------------------------------------------CGGCGCCGACGCAAGTCTT------------------------------------------------------------------------------------------------------------------------------------------------------------      0.680      0.417      0.301
----------------------------------------------------------------------------------------------------------CGGCGCCGACGCAAGTCTTGC----------------------------------------------------------------------------------------------------------------------------------------------------------      0.000      0.038      0.038
------------------------------------------------------------------------------------------------------------GCGCCGACGCAAGTCTTGCGT--------------------------------------------------------------------------------------------------------------------------------------------------------      0.049      0.038      0.075
```

```
-----------------------------------------------------------------------------------------------------------------------------GCGTCGTTTCCACCCTTACGT--------------------------------------------------------------------------------------------------------------------------      0.729      0.075      0.075
----------------------------------------------------------------------------------------------------------------------------------------------ACGTGCGGCGATACGCAGT-----------------------------------------------------------------------------------------------------------      0.000      0.038      0.038
-------------------------------------------------------------------------------------------------------------------------------------------------GCGGCGATACGCAGTTGCGAC------------------------------------------------------------------------------------------------------      0.000      0.075      0.075
-------------------------------------------------------------------------------------------------------------------------------------------------------GATACGCAGTTGCGACGCACT------------------------------------------------------------------------------------------------      0.000      0.038      0.038
-----------------------------------------------------------------------------------------------------------------------------------------------------------GCAGTTGCGACGCACTTCGGG--------------------------------------------------------------------------------------------      0.000      0.038      0.038
------------------------------------------------------------------------------------------------------------------------------------------------------------CAGTTGCGACGCACTTCGGG--------------------------------------------------------------------------------------------      0.000      0.038      0.038
------------------------------------------------------------------------------------------------------------------------------------------------------------CAGTTGCGACGCACTTCGGGT-------------------------------------------------------------------------------------------      1.311      0.188      0.188
-----------------------------------------------------------------------------------------------------------------------------------------------------------GCAGTTGCGACGCACTTCGGGT-------------------------------------------------------------------------------------------      2.234      1.099      1.468
-------------------------------------------------------------------------------------------------------------------------------------------------------------AGTTGCGACGCACTTCGGGT-------------------------------------------------------------------------------------------      0.923      0.076      0.753
-------------------------------------------------------------------------------------------------------------------------------------------------------------AGTTGCGACGCACTTCGGGTT------------------------------------------------------------------------------------------      1.311      0.076      0.075
------------------------------------------------------------------------------------------------------------------------------------------------------------------CGACGCACTTCGGGTTCGGG--------------------------------------------------------------------------------------      0.194      0.038      0.038
-------------------------------------------------------------------------------------------------------------------------------------------------------------------GACGCACTTCGGGTTCGGGT-------------------------------------------------------------------------------------      0.146      0.038      0.188
------------------------------------------------------------------------------------------------------------------------------------------------------------------CGACGCACTTCGGGTTCGGGT-------------------------------------------------------------------------------------     11.462      4.774      5.309
-----------------------------------------------------------------------------------------------------------------------------------------------------------------GCGACGCACTTCGGGTTCGGGT-------------------------------------------------------------------------------------      0.777      0.152      0.226
---------------------------------------------------------------------------------------------------------------------------------------------------------------------CGCACTTCGGGTTCGGGTCTT----------------------------------------------------------------------------------      2.477      0.076      0.151

miRNA: miR_t112,miR_t56,miR_t4 chromosome_5 Start: 3227662 End: 3227766 Condition: HS+N
-------------------------TCACGCCTCTCGCCCAGAAGG-----------      0.049      0.417      0.264
------------------------TTCACGCCTCTCGCCCAGAAG------------      0.000      0.038      0.038
-----------------CACACTTTTCACGCCTCTC---------------------      1.797      0.303      0.715
-----------------CACACTTTTCACGCCTCT----------------------      2.040      0.264      0.264
---------------TCCACACTTTTCACGCCTCT----------------------      7.334      0.909      1.242
---------------TCCACACTTTTCACGCCTCTC---------------------    235.361     22.429     35.881
---------------TCCACACTTTTCACGCCTC-----------------------      0.194      0.038      0.038
---------------TCCACACTTTTCACGCCTCTCG--------------------      8.597      1.326      3.087
---------------TCCACACTTTTCACGCC-------------------------      0.049      0.038      0.038
--------------TTCCACACTTTTCACGCCTC-----------------------      8.888      1.174      1.017
--------------TTCCACACTTTTCACGCCT------------------------      0.291      0.038      0.075
--------------TTCCACACTTTTCACGCCTCTC---------------------     45.800     18.526     22.929
--------------TTCCACACTTTTCACGCC-------------------------      0.340      0.076      0.038
--------------TTCCACACTTTTCACGC--------------------------      0.049      0.227      0.527
--------------TTCCACACTTTTCACGCCTCT----------------------    522.545    118.129    106.627 miR_t112
--------------TTCCACACTTTTCACGCCTCTCG--------------------     40.360     25.005     38.178
-------------ATTCCACACTTTTCACGCCTC-----------------------      0.049      0.075      0.075

---------                                            AGT
         GCCCATTCCACACTTTTCACGCCTCTCGCCCAGAAGGCAGCAGC   C
         CGGGTAAGGTGTGAAAAGTGCGGAGAGCGGGTCTTCCGTCGTCG   /
TCGCCACGG                                            CCC

-----ACGGCGGGTAAGGTGTGAAA--------------------------------      0.000      0.151      0.151
----CACGGCGGGTAAGGTGTGAAA--------------------------------      0.000      0.038      0.226
-----ACGGCGGGTAAGGTGTGAAAA-------------------------------      0.000      0.189      0.301
----CACGGCGGGTAAGGTGTGAAAA-------------------------------      0.000      0.076      0.038
------CGGCGGGTAAGGTGTGAAAA-------------------------------      0.000      0.152      0.489
------CGGCGGGTAAGGTGTGAAAAG------------------------------      0.097      0.189      0.414
-------GGCGGGTAAGGTGTGAAAAG------------------------------      0.486      0.493      1.280
-----ACGGCGGGTAAGGTGTGAAAAGT-----------------------------      0.146      0.303      0.715
-------GGCGGGTAAGGTGTGAAAAGT-----------------------------     35.115     88.653    150.904 miR_t56
--------GCGGGTAAGGTGTGAAAAGT-----------------------------      0.243      0.038      0.151
-----------GGTAAGGTGTGAAAAGT-----------------------------      0.340      0.606      0.527
------CGGCGGGTAAGGTGTGAAAAGT-----------------------------      2.186      9.585      9.601
----------GGGTAAGGTGTGAAAAGT-----------------------------      0.243      0.152      0.339
---------CGGGTAAGGTGTGAAAAGT-----------------------------      0.146      0.530      1.920
------------GTAAGGTGTGAAAAGTGCGG-------------------------      0.000      0.113      0.113
-----------GGTAAGGTGTGAAAAGTGCGG-------------------------      0.243      0.303      0.414
------------GTAAGGTGTGAAAAGTGCGGA------------------------      1.117      1.440      2.184 miR_t112*
-----------GGTAAGGTGTGAAAAGTGCGGA------------------------      0.291      0.568      0.339
-------------TAAGGTGTGAAAAGTGCGGAG-----------------------      2.477      0.682      1.130
------------GTAAGGTGTGAAAAGTGCGGAG-----------------------      0.049      0.113      0.113
----------------GGTGTGAAAAGTGCGGAG-----------------------      0.000      0.075      0.075
---------------AGGTGTGAAAAGTGCGGAG-----------------------      0.000      0.113      0.113
--------------AAGGTGTGAAAAGTGCGGAG-----------------------      0.000      0.076      0.038
----------------GGTGTGAAAAGTGCGGAGA----------------------      0.146      0.152      0.151
------------------TGTGAAAAGTGCGGAGA----------------------      0.000      0.038      0.038
--------------AAGGTGTGAAAAGTGCGGAGA----------------------      2.040      1.061      2.071 miR_t4
---------------------GAAAAGTGCGGAGAGCGGGT----------------      0.291      0.568      1.130
--------------------TGAAAAGTGCGGAGAGCGGGT----------------      0.097      0.189      0.640
-----------------------AAAGTGCGGAGAGCGGGTCT--------------      0.000      0.113      0.113
---------------------GAAAAGTGCGGAGAGCGGGTCT--------------      0.194      0.076      0.038
----------------------AAAAGTGCGGAGAGCGGGTCT--------------      0.097      0.038      0.038
--------------------TGAAAAGTGCGGAGAGCGGGTCTT-------------      0.000      0.151      0.151
-----------------------AAAGTGCGGAGAGCGGGTCTT-------------      0.194      0.568      1.167
----------------------AAAAGTGCGGAGAGCGGGTCTT-------------      0.049      0.985      0.339
-------------------------AGTGCGGAGAGCGGGTCTT-------------      0.000      0.151      0.151
---------------------GAAAAGTGCGGAGAGCGGGTCTT-------------      0.000      0.758      0.113
------------------------AAGTGCGGAGAGCGGGTCTT-------------      0.000      0.038      0.075
----------------------------GCGGAGAGCGGGTCTTCCGT---------      0.000      0.113      0.113

miRNA: miR_t117 chromosome_5 Start: 1813891 End: 1814320 Condition: HS+N
---------------------------------------------------------------------------------------------------------------------------------------------------------------------------------------------------------------------------------------------------------------------------AACTTGCTCTGCGCCCACGGC----------------------------------      0.000      0.076      0.188
----------------------------------------------------------------------------------------------------------------------------------------------------------------------------------------------------------------------------------------------------------------------TGAGGAACTTGCTCTGCGCCC---------------------------------------      0.631      0.114      0.038
-------------------------------------------------------------------------------------------------------------------------------------------------------------------------------------------------------------------------------------------------------------TCTGCAGAATGAGGAACTTGC------------------------------------------------      0.049      0.038      0.038
------------------------------------------------------------------------------------------------------------------------------------------------------------------------------------------------------------------------------------------------------------TTCTGCAGAATGAGGAACTTGC------------------------------------------------      0.000      0.341      0.753
--------------------------------------------------------------------------------------------------------------------------------------------------------------------------------------------------------------------------------------------TGACCCCAGCGGCTGGTTCTG-----------------------------------------------------------------      0.000      0.075      0.075
---------------------------------------------------------------------------------------------------------------------------------------------------------------------------------------------------------------------------------------TGGCTTGACCCCAGCGGCTGG----------------------------------------------------------------------     14.813      0.985      0.979
---------------------------------------------------------------------------------------------------------------------------------------------------------------------------------------------------------------------------------------TGGCTTGACCCCAGCGGCTGGT---------------------------------------------------------------------      1.117      0.152      0.075
------------------------------------------------------------------------------------------------------------------------------------------------------------------------------------------------------------------------------------GAACAACTCTCCCACGCTGT------------------------------------------------------------------------------------------      0.000      0.038      0.075
------------------------------------------------------------------------------------------------------------------------------------------------------------------------------------------------------------------------------------GAACAACTCTCCCACGCTG-------------------------------------------------------------------------------------------      0.097      1.393      1.393
-----------------------------------------------------------------------------------------------------------------------------------------------------------------------------------------------------------------------------------TGAACAACTCTCCCACGCTG-------------------------------------------------------------------------------------------      2.088      0.796      8.848
-----------------------------------------------------------------------------------------------------------------------------------------------------------------------------------------------------------------------------------TGAACAACTCTCCCACGCT--------------------------------------------------------------------------------------------      0.049      0.038      0.038
-----------------------------------------------------------------------------------------------------------------------------------------------------------------------------------------------------------------------------------TGAACAACTCTCCCACGCTGT------------------------------------------------------------------------------------------     28.849      1.705      3.238
-----------------------------------------------------------------------------------------------------------------------------------------------------------------------------------------------------------------------------------TGAACAACTCTCCCACGCTGTG-----------------------------------------------------------------------------------------      0.000      0.038      0.038
----------------------------------------------------------------------------------------------------------------------------------------------------------------------------------------------------------------------------------TTGAACAACTCTCCCACGCT--------------------------------------------------------------------------------------------      0.049      0.265      0.339
----------------------------------------------------------------------------------------------------------------------------------------------------------------------------------------------------------------------------------TTGAACAACTCTCCCACGCTG-------------------------------------------------------------------------------------------     44.246     49.744    362.088 miR_t117
----------------------------------------------------------------------------------------------------------------------------------------------------------------------------------------------------------------------------------TTGAACAACTCTCCCACGCTGT------------------------------------------------------------------------------------------      0.729      0.303      0.527
----------------------------------------------------------------------------------------------------------------------------------------------------------------------------------------------------------------------------------TTGAACAACTCTCCCACGC---------------------------------------------------------------------------------------------      0.000      0.493      0.151

------------------------------------------------------------------------------------------------------------------------------------------------------------------------------------------------------------------------------                                                                               G      GTTAGT    G   A-   A-     CGG
                                                                                                                                                                                                                              GCGGTTGAACAACTCTCCCACGCTGTGGCTTGACCCCAGCGGCTGGTTCTGCAGAATGAGGAACTTGCTCTGCGCCCAC GCTGCA      TGCG GCT  CAC  GGTTG   C
                                                                                                                                                                                                                              CGCCAACTTGTTGAGAGGGTGCGACACCGAACTGGGGTCGCCGACCAAGACGTTTTACTCCTTGAACGAGACGCGGGTG CGACGT      ACGT CGG  GTG  CCGAC   /
GCCACGGCAGATTCGTCGCAGGCTTCTTCACGCACCGCACACCTTCACGCACCGCACAGGAAAGGTGTGCGGTGTGTGAAGAACCCTGCGACGAATCTGCCGTGGCTCATCCGCGGCGACCGGTGGTACAGCAGGAACAACAGGACGTGCCTCCGGCGGTCGAGGTACGGCGGGACAACGCCGGTGGTGAACAGACGCGGGCAGCA                                                                               A      ------    G   CG   GA     AAA

----CGGCAGATTCGTCGCAGGCT----------------------------------------------------------------------------------------------------------------------------------------------------------------------------------------------------------------------------------------------------------------------------------------------------------      0.000      0.038      0.038
-----GGCAGATTCGTCGCAGGCTTCT-------------------------------------------------------------------------------------------------------------------------------------------------------------------------------------------------------------------------------------------------------------------------------------------------------      0.000      0.076      0.075
---------------------------------------------------------------GGTGTGCGGTGTGTGAAGAAC----------------------------------------------------------------------------------------------------------------------------------------------------------------------------------------------------------------------------------------------      0.000      0.038      0.038
-------------------------------------------------------------------------------------------------------------------------------------------------------------------------------------------------------------ACGCCAACTTGTTGAGAGGGT------------------------------------------------------------------------------------------------      0.049      0.530      0.113
-------------------------------------------------------------------------------------------------------------------------------------------------------------------------------------------------------------------------------GGTGCGACACCGAACTGGGGT------------------------------------------------------------------------------      0.631      0.341      0.151
----------------------------------------------------------------------------------------------------------------------------------------------------------------------------------------------------------------------------------------------------CGCCGACCAAGACGTTTTACT---------------------------------------------------------      0.049      0.075      0.075
-----------------------------------------------------------------------------------------------------------------------------------------------------------------------------------------------------------------------------------------------------GCCGACCAAGACGTTTTACT---------------------------------------------------------      0.000      0.113      0.113

miRNA: miR_t123 chromosome_10 Start: 4598528 End: 4598811 Condition: HS+N
-----------------------------------------------------------------------------------------------------------------------------------------------------GGCGGGCTGAAGACATAGA-------      0.923      0.871      2.861
----------------------------------------------------------------------------------------------------------------------------------------------------TGGCGGGCTGAAGACATAGA-------      5.828      5.304     14.119
----------------------------------------------------------------------------------------------------------------------------------------------------TGGCGGGCTGAAGACATAGAA------      0.049      0.152      0.038
----------------------------------------------------------------------------------------------------------------------------------------------------TGGCGGGCTGAAGACATAG--------      0.389      0.947      1.544
----------------------------------------------------------------------------------------------------------------------------------------------------TGGCGGGCTGAAGACAT----------      0.000      0.038      0.038
---------------------------------------------------------------------------------------------------------------------------------------------------TTGGCGGGCTGAAGACATAGAA------      3.351      5.342      0.828
---------------------------------------------------------------------------------------------------------------------------------------------------TTGGCGGGCTGAAGACAT----------      0.049      0.265      0.188
---------------------------------------------------------------------------------------------------------------------------------------------------TTGGCGGGCTGAAGACATA---------      0.049      0.303      0.226
---------------------------------------------------------------------------------------------------------------------------------------------------TTGGCGGGCTGAAGACATAGA-------    583.012   1908.360   2827.577 miR_t123
---------------------------------------------------------------------------------------------------------------------------------------------------TTGGCGGGCTGAAGACATAG--------     22.196     42.546     70.143
---------------------------------------------------------------------------------------------------------------------------------------------------TTGGCGGGCTGAAGACA-----------      0.000      0.038      0.151
--------------------------------------------------------------------------------------------------------------------------------------------------CTTGGCGGGCTGAAGACA-----------      0.000      0.417      0.753
--------------------------------------------------------------------------------------------------------------------------------------------------CTTGGCGGGCTGAAGACATAGAA------      0.000      0.114      0.038
--------------------------------------------------------------------------------------------------------------------------------------------------CTTGGCGGGCTGAAGACATAG--------    177.517   1853.387   2094.289
--------------------------------------------------------------------------------------------------------------------------------------------------CTTGGCGGGCTGAAGACATAGA-------      0.583      9.812      7.116
--------------------------------------------------------------------------------------------------------------------------------------------------CTTGGCGGGCTGAAGACAT----------      0.049      0.038      0.038
--------------------------------------------------------------------------------------------------------------------------------------------------CTTGGCGGGCTGAAGACATA---------      0.146      0.530      0.640
-------------------------------------------------------------------------------------------------------------------------------------------------GCTTGGCGGGCTGAAGACAT----------      0.000      0.038      0.075
------------------------------------------------------------------------------------------------------------------------------------------------AGCTTGGCGGGCTGAAGACAT----------      0.049      1.440      2.749
-----------------------------------------------------------------------------------------------------------------------------------------------AAGCTTGGCGGGCTGAAGACAT----------      0.146      0.682      0.188
-----------------------------------------------------------------------------------------------------------------------------------------------AAGCTTGGCGGGCTGAAGACA-----------      0.000      0.493      0.866
---------------------------------------------------------------------------------------------------------------------------GGAGCGCGATGATGGATGAGA-------------------------------      0.000      0.075      0.075
--------------------------------------------------------------------------------------------------------------------------GGGAGCGCGATGATGGATGAG--------------------------------      0.097      0.152      0.226
-------------------------------------------------------------------------------------------------------------------------TGGGAGCGCGATGATGGATGAGAAGC----------------------------      0.000      0.075      0.075
-------------------------------------------------------------------------------------------------------------------------TGGGAGCGCGATGATGGATGA---------------------------------      0.097      0.341      0.301
-------------------------------------------------------------------------------------------------------------------------TGGGAGCGCGATGATGGATGAG--------------------------------      2.283      7.274      4.593
-------------------------------------------------------------------------------------------------------------------------TGGGAGCGCGATGATGGATGAGAA------------------------------      0.097      0.189      0.038
-------------------------------------------------------------------------------------------------------------------------TGGGAGCGCGATGATGGATG----------------------------------      0.097      0.227      0.264
-------------------------------------------------------------------------------------------------------------------------TGGGAGCGCGATGATGGATGAGA-------------------------------      2.720     11.025      1.619
-------------------------------------------------------------------------------------------------------------------------TGGGAGCGCGATGATGGATGAGAAG-----------------------------      0.243      1.174      0.038
-----------------------------------------------------------------------------------------------------TGGCGGGCTGAAGACATAC-------------------------------------------------------      0.000      0.114      0.188
-----------------------------------------------------------------------------------------------------TGGCGGGCTGAAGACATACCTG----------------------------------------------------      0.000      0.038      0.038
-----------------------------------------------------------------------------------------------------TGGCGGGCTGAAGACAT---------------------------------------------------------      0.000      0.038      0.038
-----------------------------------------------------------------------------------------------------TGGCGGGCTGAAGACATACCTGG---------------------------------------------------      0.000      0.227      0.226
-----------------------------------------------------------------------------------------------------TGGCGGGCTGAAGACATACCT-----------------------------------------------------      0.000      0.075      0.075
-----------------------------------------------------------------------------------------------------TGGCGGGCTGAAGACATACC------------------------------------------------------      0.000      0.038      0.038
----------------------------------------------------------------------------------------------------TTGGCGGGCTGAAGACATACC------------------------------------------------------      0.243      0.644      0.452
----------------------------------------------------------------------------------------------------TTGGCGGGCTGAAGACATA--------------------------------------------------------      0.049      0.303      0.226
----------------------------------------------------------------------------------------------------TTGGCGGGCTGAAGACATAC-------------------------------------------------------      0.097      1.478      1.883
----------------------------------------------------------------------------------------------------TTGGCGGGCTGAAGACATACCT-----------------------------------------------------      0.146      0.227      0.038
----------------------------------------------------------------------------------------------------TTGGCGGGCTGAAGACA----------------------------------------------------------      0.000      0.038      0.151
----------------------------------------------------------------------------------------------------TTGGCGGGCTGAAGACAT---------------------------------------------------------      0.049      0.265      0.188
---------------------------------------------------------------------------------------------------CTTGGCGGGCTGAAGACATACC------------------------------------------------------      0.049      0.644      0.038
---------------------------------------------------------------------------------------------------CTTGGCGGGCTGAAGACAT---------------------------------------------------------      0.049      0.038      0.038
---------------------------------------------------------------------------------------------------CTTGGCGGGCTGAAGACATA--------------------------------------------------------      0.146      0.530      0.640
---------------------------------------------------------------------------------------------------CTTGGCGGGCTGAAGACA----------------------------------------------------------      0.000      0.417      0.753
---------------------------------------------------------------------------------------------------CTTGGCGGGCTGAAGACATAC-------------------------------------------------------      0.194      1.894      2.372
--------------------------------------------------------------------------------------------------GCTTGGCGGGCTGAAGACAT---------------------------------------------------------      0.000      0.038      0.075
-------------------------------------------------------------------------------------------------AGCTTGGCGGGCTGAAGACAT---------------------------------------------------------      0.049      1.440      2.749
------------------------------------------------------------------------------------------------AAGCTTGGCGGGCTGAAGACAT---------------------------------------------------------      0.146      0.682      0.188
------------------------------------------------------------------------------------------------AAGCTTGGCGGGCTGAAGACA----------------------------------------------------------      0.000      0.493      0.866
----------------------------------------------------------------------------GGAGCGCGATGATGGATGAGA------------------------------------------------------------------------------      0.000      0.075      0.075
-------------------------------GGAGCGCGATGATGGATGAGA-------------------------------------------------------------------------------------------------------------------------------      0.000      0.075      0.075
------------------------------GGGAGCGCGATGATGGATGAG--------------------------------------------------------------------------------------------------------------------------------      0.097      0.152      0.226
```

```
-----------------------------TGGGAGCGCGATGATGGATGAG----------------------------------------------------------------------------------------------------------------------------     2.283     7.274     4.593
-----------------------------TGGGAGCGCGATGATGGATGAGAAG-------------------------------------------------------------------------------------------------------------------------     0.243     1.174     0.038
-----------------------------TGGGAGCGCGATGATGGATGA-----------------------------------------------------------------------------------------------------------------------------     0.097     0.341     0.301
-----------------------------TGGGAGCGCGATGATGGATGAGA---------------------------------------------------------------------------------------------------------------------------     2.720    11.025     1.619
-----------------------------TGGGAGCGCGATGATGGATGAGAA--------------------------------------------------------------------------------------------------------------------------     0.097     0.189     0.038
-----------------------------TGGGAGCGCGATGATGGATGAGAAGC------------------------------------------------------------------------------------------------------------------------     0.000     0.075     0.075
-----------------------------TGGGAGCGCGATGATGGATG------------------------------------------------------------------------------------------------------------------------------     0.097     0.227     0.264
---------TGGCGGGCTGAAGACATACCTG------------------------------------------------------------------------------------------------------------------------------------------------     0.000     0.038     0.038
---------TGGCGGGCTGAAGACATACCT-------------------------------------------------------------------------------------------------------------------------------------------------     0.000     0.075     0.075
---------TGGCGGGCTGAAGACAT-----------------------------------------------------------------------------------------------------------------------------------------------------     0.000     0.038     0.038
---------TGGCGGGCTGAAGACATAC---------------------------------------------------------------------------------------------------------------------------------------------------     0.000     0.114     0.188
---------TGGCGGGCTGAAGACATACCTGG-----------------------------------------------------------------------------------------------------------------------------------------------     0.000     0.227     0.226
---------TGGCGGGCTGAAGACATACC--------------------------------------------------------------------------------------------------------------------------------------------------     0.000     0.038     0.038
--------TTGGCGGGCTGAAGACA------------------------------------------------------------------------------------------------------------------------------------------------------     0.000     0.038     0.151
--------TTGGCGGGCTGAAGACATA----------------------------------------------------------------------------------------------------------------------------------------------------     0.049     0.303     0.226
--------TTGGCGGGCTGAAGACATACCT-------------------------------------------------------------------------------------------------------------------------------------------------     0.146     0.227     0.038
--------TTGGCGGGCTGAAGACATACC--------------------------------------------------------------------------------------------------------------------------------------------------     0.243     0.644     0.452
--------TTGGCGGGCTGAAGACATAC---------------------------------------------------------------------------------------------------------------------------------------------------     0.097     1.478     1.883
--------TTGGCGGGCTGAAGACAT-----------------------------------------------------------------------------------------------------------------------------------------------------     0.049     0.265     0.188
-------CTTGGCGGGCTGAAGACAT-----------------------------------------------------------------------------------------------------------------------------------------------------     0.049     0.038     0.038
-------CTTGGCGGGCTGAAGACATA----------------------------------------------------------------------------------------------------------------------------------------------------     0.146     0.530     0.640
-------CTTGGCGGGCTGAAGACA------------------------------------------------------------------------------------------------------------------------------------------------------     0.000     0.417     0.753
-------CTTGGCGGGCTGAAGACATACC--------------------------------------------------------------------------------------------------------------------------------------------------     0.049     0.644     0.038
-------CTTGGCGGGCTGAAGACATAC---------------------------------------------------------------------------------------------------------------------------------------------------     0.194     1.894     2.372
------GCTTGGCGGGCTGAAGACAT-----------------------------------------------------------------------------------------------------------------------------------------------------     0.000     0.038     0.075
-----AGCTTGGCGGGCTGAAGACAT-----------------------------------------------------------------------------------------------------------------------------------------------------     0.049     1.440     2.749
----AAGCTTGGCGGGCTGAAGACAT-----------------------------------------------------------------------------------------------------------------------------------------------------     0.146     0.682     0.188
----AAGCTTGGCGGGCTGAAGACA------------------------------------------------------------------------------------------------------------------------------------------------------     0.000     0.493     0.866

TGAGAAGCTTGGCGGGCTGAAGACATACCTGGGAGCGCGATGATGG  G     C                     G                      C                  ACCTGGGAGCGCGATGATGGATG                       T GA    TG
                                              AT AGAAG TTGGCGGGCTGAAGACCGAGA GAGCGCGATGATGGATGAGAAG TTGGCGGGCTGAAGACAT                       AGAAGCTTGGCGGGCTGAAGACA A  AGCT  C
                                              TG TCTTC AACCGCCCGACTTCTGGCTCT CTCGCGCTACTACCTACTCTTC AACCGCCCGACTTCTGTA                       TCTTCGAACCGCCCGACTTCTGT T  TCGG  /
-------------------------------------------AGG  G     T                     A                      T                  -----------------------                       C AG    GG

-----------------------------------------------GGTCTTCTAACCGCCCGACTT-----------------------------------------------------------------------------------------------------------     0.000     0.038     0.038
--------------------------------------------------CTTCTAACCGCCCGACTTCT---------------------------------------------------------------------------------------------------------    11.656     1.174     1.995
-------------------------------------------------TCTTCTAACCGCCCGACTTCT---------------------------------------------------------------------------------------------------------     6.945     0.796     1.242
----------------------------------------------------TCTAACCGCCCGACTTCTG--------------------------------------------------------------------------------------------------------     0.729     0.038     0.075
----------------------------------------------------TCTAACCGCCCGACTTCTGG-------------------------------------------------------------------------------------------------------     0.000     0.075     0.075
----------------------------------------------------TCTAACCGCCCGACTTCTGGCT-----------------------------------------------------------------------------------------------------     0.049     0.038     0.188
----------------------------------------------------TCTAACCGCCCGACTTCTGGCTCT---------------------------------------------------------------------------------------------------     0.194     0.076     0.151
-------------------------------------------------------------------------TCTACTCGCGCTACTACCTA----------------------------------------------------------------------------------     0.000     0.075     0.075
--------------------------------------------------------------------------CTACTCGCGCTACTACCTACT--------------------------------------------------------------------------------     0.486     1.099     0.226
----------------------------------------------------------------------------ACTCGCGCTACTACCTACT--------------------------------------------------------------------------------     0.097     0.038     0.038
---------------------------------------------------------------------------TACTCGCGCTACTACCTACT--------------------------------------------------------------------------------     0.000     0.114     0.075
-------------------------------------------------------------------------TCTACTCGCGCTACTACCTACT--------------------------------------------------------------------------------     0.000     0.076     0.075
----------------------------------------------------------------------------ACTCGCGCTACTACCTACTC-------------------------------------------------------------------------------     0.243     0.038     0.151
--------------------------------------------------------------------------CTACTCGCGCTACTACCTACTC-------------------------------------------------------------------------------     0.000     0.038     0.038
---------------------------------------------------------------------------TACTCGCGCTACTACCTACTCT------------------------------------------------------------------------------     1.166     0.985     0.602
-------------------------------------------------------------------------TCTACTCGCGCTACTACCTACTCT------------------------------------------------------------------------------     0.971     0.833     0.602
----------------------------------------------------------------------------ACTCGCGCTACTACCTACTCT------------------------------------------------------------------------------    35.358    15.079    12.500
--------------------------------------------------------------------------CTACTCGCGCTACTACCTACTCT------------------------------------------------------------------------------     0.874     2.084     1.732
----------------------------------------------------------------------------ACTCGCGCTACTACCTACTCTT-----------------------------------------------------------------------------     0.437     0.189     0.452
--------------------------------------------------------------------------CTACTCGCGCTACTACCTACTCTT-----------------------------------------------------------------------------     0.389     0.606     0.339
-------------------------------------------------------------------------TCTACTCGCGCTACTACCTACTCTT-----------------------------------------------------------------------------     0.097     0.038     0.038
---------------------------------------------------------------------------TACTCGCGCTACTACCTACTCTT-----------------------------------------------------------------------------     0.097     0.189     0.151
-------------------------------------------------------------------------------CGCGCTACTACCTACTCTTCT---------------------------------------------------------------------------     2.234     0.227     0.188
--------------------------------------------------------------------------------------------ACTCTTCTAACCGCCCGACT---------------------------------------------------------------     0.486     0.152     0.113
-------------------------------------------------------------------------------------------TACTCTTCTAACCGCCCGACT---------------------------------------------------------------     0.000     0.075     0.075
-----------------------------------------------------------------------------------------------CTTCTAACCGCCCGACTTCT------------------------------------------------------------    11.656     1.174     1.995
----------------------------------------------------------------------------------------------TCTTCTAACCGCCCGACTTCT------------------------------------------------------------     6.945     0.796     1.242
-------------------------------------------------------------------------------------------------TCTAACCGCCCGACTTCTG-----------------------------------------------------------     0.729     0.038     0.075
-------------------------------------------------------------------------------------------------TCTAACCGCCCGACTTCTGT----------------------------------------------------------    96.262     6.479    12.462
------------------------------------------------------------------------------------------------TTCTAACCGCCCGACTTCTGT----------------------------------------------------------     0.874     0.076     0.113
--------------------------------------------------------------------------------------------------CTAACCGCCCGACTTCTGT----------------------------------------------------------     0.486     0.152     0.075
-----------------------------------------------------------------------------------------------CTTCTAACCGCCCGACTTCTGT----------------------------------------------------------     2.380     0.265     0.075
--------------------------------------------------------------------------------------------------CTAACCGCCCGACTTCTGTA---------------------------------------------------------     0.291     0.038     0.038
-------------------------------------------------------------------------------------------------TCTAACCGCCCGACTTCTGTA---------------------------------------------------------     0.194     0.455     0.678
--------------------------------------------------------------------------------------------------CTAACCGCCCGACTTCTGTA-----------------------T---------------------------------    38.952     6.024     7.869
---------------------------------------------------------------------------------------------------TAACCGCCCGACTTCTGTA-----------------------T---------------------------------     0.146     0.038     0.075
-------------------------------------------------------------------------------------------------TCTAACCGCCCGACTTCTGTA-----------------------T---------------------------------    19.767     4.471    10.467
------------------------------------------------------------------------------------------------------------------------------------------------TCGAACCGCCCGACTTCTGT-----------     0.000     0.038     0.038

miRNA: miR_t125 chromosome_12 Start: 600837 End: 601216 Condition: HS+N
---------------------------------------------------------------------------------------TGGCGCACCGGTCCTCGTACC-----------------------------------------------------------------------------------------------------------------------------------------------------     0.000     0.038     0.038

-----------------------------------------------------------------------------------AC                                   CACGAGCCCGCTC     CA               A   C          GATATGGCC  G          C    T           CC-- -      A  TCGCCCCGGATCGTGTTCGGCTGCATT    A
                                                                                     TTTGGCGCACCGGTCCTCGTACCTCGAAATGTCTA             TAGGT  TTCGCCATGCTCGCA CGC CGAAGGCAGT         GG GGCAAGACCG CCTT GGGCAAGGTCG    C CGGCCT GG                           GGAG A
                                                                                     AAACCGCGTGGCCAGGAGCATGGAGCTTTACAGAT             ATCCA  AAGCGGTACGAGCGT GCG GCTTCCGTCA         TC CCGTTCTGGC GGAA CCCGTTCCAGC    G GCTGGA CC                           CTTC G
ATCACGAGGTGGTTCAAACGGTCGTCGCCCAAGTCGACCGAGAGCGTCGACGTACCAGTCGCCAAGATCTATCGAGGAAGAGTTC                                   TG-----------     AG               G   T          ---------  A          A    -           CATT T      -  ---------------------------    G

----CGAGGTGGTTCAAACGGTCGT----------------------------------------------------------------------------------------------------------------------------------------------------------------------------------------------------------------------------------------     0.340     0.758     0.678
------------------------------------------------------------------------------------------CGTGGCCAGGAGCATGGAGC---------------------------------------------------------------------------------------------------------------------------------------------------     0.000     0.038     0.038
------------------------------------------------------------------------------------------CGTGGCCAGGAGCATGGAGCT--------------------------------------------------------------------------------------------------------------------------------------------------     1.020     2.387     5.610
--------------------------------------------------------------------------------------------TGGCCAGGAGCATGGAGCT--------------------------------------------------------------------------------------------------------------------------------------------------     0.000     0.189     0.489
-------------------------------------------------------------------------------------------GTGGCCAGGAGCATGGAGCT--------------------------------------------------------------------------------------------------------------------------------------------------     0.194     0.038     0.414
---------------------------------------------------------------------------------------------GGCCAGGAGCATGGAGCT--------------------------------------------------------------------------------------------------------------------------------------------------     0.000     0.151     0.151
-------------------------------------------------------------------------------------------GTGGCCAGGAGCATGGAGCTT-------------------------------------------------------------------------------------------------------------------------------------------------     1.311     1.705     2.786
--------------------------------------------------------------------------------------------TGGCCAGGAGCATGGAGCTT-------------------------------------------------------------------------------------------------------------------------------------------------     0.194     0.530     0.715
---------------------------------------------------------------------------------------------GGCCAGGAGCATGGAGCTT-------------------------------------------------------------------------------------------------------------------------------------------------     0.000     0.038     0.038
-------------------------------------------------------------------------------------------GTGGCCAGGAGCATGGAGCTTT------------------------------------------------------------------------------------------------------------------------------------------------     2.331     7.312     4.518
------------------------------------------------------------------------------------------CGTGGCCAGGAGCATGGAGCTTT------------------------------------------------------------------------------------------------------------------------------------------------     0.049     0.038     0.038
--------------------------------------------------------------------------------------------TGGCCAGGAGCATGGAGCTTT------------------------------------------------------------------------------------------------------------------------------------------------    19.184    58.648    93.223 miR_t125
---------------------------------------------------------------------------------------------GGCCAGGAGCATGGAGCTTT------------------------------------------------------------------------------------------------------------------------------------------------     0.923     1.364     0.866

miRNA: miR_t127 chromosome_5 Start: 2264251 End: 2264412 Condition: HS+N
-----------------------------------------------------------------------------TGAAACAGGTGGGGCGTAA------------------     0.000     0.075     0.075
-----------------------------------------------------------------------------TGAAACAGGTGGGGCGTAACT----------------     0.097     0.113     0.113
---------------------------------------------------------------------TGGGTGCGTGAAACAGGTG--------------------------     0.000     0.038     0.038
---------------------------------------------------------------------TGGGTGCGTGAAACAGGTGGG------------------------     0.194     0.038     0.151
--------------------------------------------------------------------TTGGGTGCGTGAAACAGGTGG-------------------------     1.846     0.417     0.565
--------------------------------------------------------------------TTGGGTGCGTGAAACAGGTG--------------------------     0.486     0.189     0.715
-------------------------------------------------------------------TTTGGGTGCGTGAAACAGGTGG-------------------------     0.097     0.189     0.113
-------------------------------------------------------------------TTTGGGTGCGTGAAACAGGTG--------------------------    26.664    25.156    34.865 miR_t127
-------------------------------------------------------------------TTTGGGTGCGTGAAACAGGT---------------------------     1.991     1.061     5.045
-------------------------------------------------------------------TTTGGGTGCGTGAAACAGG----------------------------     0.000     0.075     0.075

---------------------------------------------------------------                     G       G  A    C-    - TGTCT
                                                               TGCCTTTGGGTGCGTGAAACA GTGGGGC TA CTTG  TCAG C     T
                                                               ACGGAAACTCACGCACTTTGT CACCCTG GT GAAC  AGTC G     /
CGAACGCAGTGGTAACGAAGTTACATTGAAGACGATTACGATTTGAGAGTAGATTGATTGTCG                     A       G  -    AA    C TTTTT

----CGCAGTGGTAACGAAGTTACAT----------------------------------------------------------------------------------------     0.000     0.075     0.075

miRNAs identified in TAP and HS-N :

miRNA: miR_t90 chromosome_16 Start: 185086 End: 185184 Condition: TAP
------------------------------TAAGCCGGGGTTGGTAG----     14.328     4.167     1.017
------------------------------TAAGCCGGGGTTGGTAGATG-      0.097     0.000     0.000
------------------------------TAAGCCGGGGTTGGTA-----      0.146     0.000     0.000
------------------------------TAAGCCGGGGTTGGTAGA---      0.486     0.076     0.188
------------------------------TAAGCCGGGGTTGGT------      0.049     0.000     0.000
------------------------------TAAGCCGGGGTTGG-------      0.049     0.000     0.000
------------------------TCCACATAAGCCGGGGTTGG-------      1.020     0.114     0.188
------------CAGCAAGCAACATCCACA---------------------      0.049     0.000     0.000
------------CAGCAAGCAACATCCACATA-------------------      0.049     0.000     0.000
----------TCCAGCAAGCAACATCCAC----------------------      0.194     0.000     0.000
----------TCCAGCAAGCAACATCCACA---------------------      5.003     0.985     1.242
----------TCCAGCAAGCAACATCCACATA-------------------      0.777     0.341     0.226
----------TCCAGCAAGCAACATCCACAT--------------------      1.457     0.530     0.377
---------ATCCAGCAAGCAACATCCACA---------------------      0.097     0.000     0.000 miR_t90*
--------TATCCAGCAAGCAACATCCAC----------------------      0.291     0.189     0.377
--------TATCCAGCAAGCAACATCCACA---------------------      4.420     1.478     2.937
--------TATCCAGCAAGCAACATCCACAT--------------------      0.049     0.075     0.075

----                                           ATG
    ACGGTATCCAGCAAGCAACATCCACATAAGCCGGGGTTGGTAG   T
    TGCCATAGGTCGTTCGTTGTAGGTGTATTCGGCCCCAACCATC   G
GATA                                           GGG

--------ATAGGTCGTTCGTTGT---------------------------      0.049     0.000     0.000
-----GCCATAGGTCGTTCGTTGT---------------------------      0.097     0.000     0.000
------CCATAGGTCGTTCGTTGTAGG------------------------     11.705     2.917     1.995
--------ATAGGTCGTTCGTTGTAGG------------------------     30.501     4.357     8.283
-------CATAGGTCGTTCGTTGTAGG------------------------    102.770    58.648   186.560
----------AGGTCGTTCGTTGTAGG------------------------      0.049     0.076     0.038
---------TAGGTCGTTCGTTGTAGG------------------------      0.486     0.568     0.602
-----------GGTCGTTCGTTGTAGG------------------------      0.097     0.000     0.000
---------TAGGTCGTTCGTTGTAGGT-----------------------     64.304    42.660    57.756
--------------CGTTCGTTGTAGGT-----------------------      0.049     0.000     0.000
------------GTCGTTCGTTGTAGGT-----------------------      0.389     0.000     0.000
--------ATAGGTCGTTCGTTGTAGGT-----------------------   4629.810   846.944  1017.513
----TGCCATAGGTCGTTCGTTGTAGGT-----------------------      0.486     0.568     0.188
-------CATAGGTCGTTCGTTGTAGGT-----------------------   8917.991  7059.466 13144.240 miR_t90
------CCATAGGTCGTTCGTTGTAGGT-----------------------   1537.231   334.913   189.685
-----GCCATAGGTCGTTCGTTGTAGGT-----------------------      2.380     1.061     0.527
-----------GGTCGTTCGTTGTAGGT-----------------------      6.362     5.153     4.330
-------------TCGTTCGTTGTAGGT-----------------------      0.049     0.000     0.000
----------AGGTCGTTCGTTGTAGGT-----------------------      5.245     5.001     3.389
--------ATAGGTCGTTCGTTGTAGGTG----------------------      1.748     0.152     0.152
-------CATAGGTCGTTCGTTGTAGGTG----------------------      0.049     0.000     0.000
---------TAGGTCGTTCGTTGTAGGTG----------------------     22.536     1.288     1.581
----------AGGTCGTTCGTTGTAGGTG----------------------      3.594     0.152     0.678
-----GCCATAGGTCGTTCGTTGTAGGTGT---------------------      0.146     0.114     0.114
---------TAGGTCGTTCGTTGTAGGTGT---------------------   3653.784   285.320   373.007
-------CATAGGTCGTTCGTTGTAGGTGT---------------------      1.166     3.599     0.075
```

```
------------GTCGTTCGTTGTAGGTGT---------------------      0.971      0.227      0.527
------CCATAGGTCGTTCGTTGTAGGTGT---------------------      0.486      1.288      1.288
-------------TCGTTCGTTGTAGGTGT---------------------      0.389      0.076      0.113
--------------CGTTCGTTGTAGGTGT---------------------      0.243      0.000      0.000
----------AGGTCGTTCGTTGTAGGTGT---------------------    415.355     51.070     72.478
--------ATAGGTCGTTCGTTGTAGGTGT---------------------    302.385     72.817      4.104
-----------GGTCGTTCGTTGTAGGTGT---------------------     13.890      5.683      7.831
---------TAGGTCGTTCGTTGTAGGTGTAT-------------------      0.097      0.000      0.000
----------AGGTCGTTCGTTGTAGGTGTAT-------------------      0.389      0.113      0.113
-----------GGTCGTTCGTTGTAGGTGTAT-------------------      6.751      1.364      1.092
------------GTCGTTCGTTGTAGGTGTATT------------------      0.049      0.038      0.038

miRNAs identified in All conditions :

miRNA: miR_t76,miR_t5 chromosome_14 Start: 3218700 End: 3218925 Condition: TAP
-----------------------------TGCACCCGG--AGGTGC---CGG-AGCGGTTGG-------------------------------------------------------------------      0.049      0.000      0.000
-----------------------------------------------------------TGGGGTTGCGGGGCCCGCAGG-------------------------------------------------      0.049      0.000      0.000
-----------------------------------------------------------------TGCGGGGCCCGCAGGTAGCTG-------------------------------------------      0.583      0.000      0.000
-----------------------------------------------------------------TGCGGGGCCCGCAGGTAGCTGC------------------------------------------     34.192      1.061      2.146 miR_t76
------------------------------------------------------------------GCGGGGCCCGCAGGTAGCTGC------------------------------------------      0.097      0.000      0.000
--------------------------------------------------------------------------------TAGCTGCCGTTGAGCCCGACGC---------------------------      0.437      0.114      0.114
--------------------------------------------------------------------------------TAGCTGCCGTTGAGCCCGACG----------------------------      0.097      0.038      0.113
------------------------------------------------------------------------------------TGCCGTTGAGCCCGACGCTGC------------------------      0.291      0.000      0.000
---------------------------------------------------------------------------------------CGTTGAGCCCGACGCTGCTGC---------------------      0.049      0.000      0.000
-----------------------------------------------------------------------------------------TTGAGCCCGACGCTGCTGC---------------------      0.291      0.379      0.075
------------------------------------------------------------------------------------------TGAGCCCGACGCTGCTGC---------------------      0.194      0.000      0.000
------------------------------------------------------------------------------------------TGAGCCCGACGCTGCTGCCGC------------------      0.049      0.000      0.000

-------------------------        C    --      ---   -   G                        A     C                    C    C - C         G
                         CAGGTGCA CCGG  AGGTGC   CGG AGC GTTGGGGTTGCGGGGCCCGCAGGT GCTGC GTTGAGCCCGACGCTGCTGC GCCG G C GGTGCTGCT C
                         GTCCACGT GGCC  TCCACG   GCC TCG CGACCCCGACGCCCTGGGCGTCCA CGACG CAGCTCGGGCTGCGACGACG CGGC C G CCACGACGG C
TGGGCAGGGGCAGGGACGTCGGCCA        C    AG      TCA   A   A                        C     C                    T    - A T         C

---------------------------------------------------------------------------------------CCAGCTCGGGCTGCGACGACGT--------------------      0.146      0.076      0.301
----------------------------------------------------------------------------------------CAGCTCGGGCTGCGACGACG---------------------      0.049      0.038      0.038
-------------------------------------------------------------------------------------CGCCAGCTCGGGCTGCGACGAC----------------------      0.631      0.379      0.075
--------------------------------------------------------------------------------------GCCAGCTCGGGCTGCGACGAC----------------------      0.049      0.038      0.113
---------------------------------------------------------------------------------------CCAGCTCGGGCTGCGACGA-----------------------      0.049      0.075      0.075
-------------------------------------------------------------------------------------CGCCAGCTCGGGCTGCGACGA-----------------------     30.355     54.367     39.420 miR_t5
----------------------------------------------------------GACCCCGACGCCCTGGGCGT---------------------------------------------------      0.049      0.000      0.000
-----------------------------ACGTCGGCCAGTCCACGTCAGCCATC--------------------------------------------------------------------------      0.146      0.038      0.038
------------------------------------CCAGTCCACGTCAGCCAT---------------------------------------------------------------------------      0.097      0.000      0.000
------------------------------CGTCGGCCAGTCCACGTCAGCCAT---------------------------------------------------------------------------      0.049      0.114      0.114
-----------------------------ACGTCGGCCAGTCCACGTCAGCC-----------------------------------------------------------------------------      0.049      0.000      0.000
------------------TCGGCCAGTCCACGTCGGCCAGT----------------------------------------------------------------------------------------      0.097      0.000      0.000
----CAGGGGCAGGGACGTCGGCCAGT------------------------------------------------------------------------------------------------------      0.680      0.076      0.076
-----AGGGGCAGGGACGTCGGCCAGT------------------------------------------------------------------------------------------------------      0.049      0.000      0.000

miRNA: miR_t44,miR_t7 chromosome_17 Start: 6144109 End: 6144230 Condition: TAP
----TCGTGTGCCTGCCGTCGTCTCG-------------------------------------------      0.049      0.038      0.038
-------TGTGCCTGCCGTCGTCTCGGT-----------------------------------------      0.291      0.038      0.038
-------TGTGCCTGCCGTCGTCTCGG------------------------------------------      0.680      0.568      0.565
--------------------GTCTCGGTCGCCTTGTCTGTT----------------------------      0.049      0.076      0.076
---------------------TCTCGGTCGCCTTGTCTGTT----------------------------      0.049      0.000      0.000
----------------------CTCGGTCGCCTTGTCTGTTTATG------------------------      0.097      0.000      0.000
-----------------------TCGGTCGCCTTGTCTGTTTATG------------------------     21.419     16.329      8.208 miR_t7*
-----------------------TCGGTCGCCTTGTCTGTTTAT-------------------------      0.049      0.000      0.000
------------------------CGGTCGCCTTGTCTGTTTATGTG----------------------      0.049      0.000      0.000
------------------------CGGTCGCCTTGTCTGTTTATG------------------------     16.756      5.645      4.179
-------------------------GGTCGCCTTGTCTGTTTATG------------------------     11.462      6.441      4.179
--------------------------GTCGCCTTGTCTGTTTATG------------------------      0.049      0.000      0.000
--------------------------GTCGCCTTGTCTGTTTATGTG----------------------      0.049      0.000      0.000
---------------------------TCGCCTTGTCTGTTTATGTGG---------------------    483.205    100.929     35.881 miR_t44
---------------------------TCGCCTTGTCTGTTTATGTGGAG-------------------      0.097      0.000      0.000
---------------------------TCGCCTTGTCTGTTTATG------------------------      0.437      0.076      0.038
---------------------------TCGCCTTGTCTGTTTATGTGGAGC------------------      0.049      0.303      0.303
---------------------------TCGCCTTGTCTGTTTATGTGGA--------------------      7.577      1.515      1.515
---------------------------TCGCCTTGTCTGTTTATGTG----------------------     42.206     35.802     19.654
----------------------------CGCCTTGTCTGTTTATG------------------------      0.049      0.000      0.000
----------------------------CGCCTTGTCTGTTTATGTGGA--------------------      0.291      0.000      0.000
----------------------------CGCCTTGTCTGTTTATGTGG---------------------      7.479      1.061      0.188
----------------------------CGCCTTGTCTGTTTATGTG----------------------      0.680      0.189      0.226
-----------------------------GCCTTGTCTGTTTATG------------------------      0.049      0.000      0.000
-----------------------------GCCTTGTCTGTTTATGTG----------------------      0.243      0.038      0.038
-----------------------------GCCTTGTCTGTTTATGTGG---------------------      1.700      0.114      0.226
-------------------------------CTTGTCTGTTTATGTGGAGC------------------      0.049      0.000      0.000
--------------------------------TTGTCTGTTTATGTGGAGC------------------      0.680      0.379      0.075
--------------------------------TTGTCTGTTTATGTGG---------------------      0.097      0.000      0.000
-----------------------------------TCTGTTTATGTGGAGCAGGGC-------------      0.194      0.000      0.000
---------------------------------------TTTATGTGGAGCAGGGCAGG----------      0.049      0.038      0.075
---------------------------------------------TGGAGCAGGGCAGGCCGCCA----      1.263      0.152      0.113
---------------------------------------------TGGAGCAGGGCAGGCCGC------      2.137      0.076      0.038
---------------------------------------------TGGAGCAGGGCAGGCCGCC-----      7.722      0.568      0.452
---------------------------------------------TGGAGCAGGGCAGGCCGCCAA---      4.274      0.227      0.151
---------------------------------------------TGGAGCAGGGCAGGCCGCCAATG-      1.603      0.076      0.075
---------------------------------------------TGGAGCAGGGCAGGCCGCCAAT--      4.225      0.455      0.151

CAGCTCGTGTGCCTG       C                                        C  TG
               CCGTCGT TCGGTCGCCTTGTCTGTTTATGTGGAGCAGGGCAGGCCGC AA  A
               GGCAGTA AGCCAGCGGAACAGACAAATACACCTCGTCCCGTCCGGCG TT  /
---------------       C                                        -  TT

----------------------------------------------CCTCGTCCCGTCCGGCG-TTT--      0.486      0.076      0.113
---------------------------------------------ACCTCGTCCCGTCCGGCG-TTT--      0.049      0.000      0.000
-------------------------------------------ACACCTCGTCCCGTCCGGCG-T----      0.146      0.075      0.075
----------------------------GCGGAACAGACAAATACACCT--------------------      1.457      0.114      0.151
---------------------------AGCGGAACAGACAAATACACCT--------------------      0.049      0.000      0.000
-------------------------CCAGCGGAACAGACAAATACA-----------------------      0.049      0.076      0.076 miR_t44*
--------------------TACAGCCAGCGGAACAGACAAAT--------------------------      1.457      4.812      3.577
---------------------ACAGCCAGCGGAACAGACAAAT--------------------------    914.976    478.122   1037.995 miR_t7
-------------------GTACAGCCAGCGGAACAGACAAAT--------------------------      0.097      0.000      0.000
------------------------GCCAGCGGAACAGACAAAT--------------------------      0.729      0.947      1.506
-----------------------AGCCAGCGGAACAGACAAAT--------------------------     76.301     51.070     61.860
----------------------CAGCCAGCGGAACAGACAAAT--------------------------     41.526     63.573    117.508
---------------------ACAGCCAGCGGAACAGACAAA---------------------------     13.599      3.410     19.616
--------------------TACAGCCAGCGGAACAGACAAA---------------------------      0.097      0.076      0.038
----------------------CAGCCAGCGGAACAGACAAA---------------------------      0.437      0.493      3.389
-----------------------AGCCAGCGGAACAGACAAA---------------------------      0.826      0.493      1.807
------------------------GCCAGCGGAACAGACAAA---------------------------      0.049      0.075      0.075
---------------------ACAGCCAGCGGAACAGACAA----------------------------      2.720      0.455      3.087
-----------------------AGCCAGCGGAACAGACAA----------------------------      0.291      0.038      0.075
----------------------CAGCCAGCGGAACAGACAA----------------------------      0.097      0.114      0.264
---------------------ACAGCCAGCGGAACAG--------------------------------      0.097      0.000      0.000

miRNA: miR_t9 chromosome_6 Start: 7063790 End: 7063888 Condition: TAP
------AAGACGGCGCACCTTCTTAAC-----------------------      0.049      0.000      0.000 miR_t9*

--                                            CAC
  CGTCAAGACGGCGCACCTTCTTAACGTCCTTCGGTGCTTGCTTG   A
  GCAGTTCTGTCGCGTGGAAGAATTGCGGGAAGCCACGGACGAAC   T
TG                                            TTT

-----GTTCTGTCGCGTGGAAGAATT------------------------      0.243      0.114      0.226
-------TCTGTCGCGTGGAAGAATT------------------------      0.097      0.000      0.000
----AGTTCTGTCGCGTGGAAGAATT------------------------      0.049      0.076      0.076
-----GTTCTGTCGCGTGGAAGAAT-------------------------      2.186      0.909      1.130
----AGTTCTGTCGCGTGGAAGAAT-------------------------     82.614     47.282     89.007 miR_t9
----AGTTCTGTCGCGTGGAAGAA--------------------------      0.680      0.341      0.904
----AGTTCTGTCGCGTGGAAGA---------------------------      0.243      0.038      0.377
-----GTTCTGTCGCGTGG-------------------------------      0.049      0.000      0.000
----AGTTCTGTCGCGTGG-------------------------------      0.146      0.000      0.000

miRNA: miR_t13,miR_t109 chromosome_10 Start: 3399743 End: 3400006 Condition: TAP
-----------------------------------------------------------------------------------------------------------------------------------CGATAAGAAGGAGCCGTAAGG-------------------------------------------      0.534      0.265      0.188 miR_t109*
------------------------------------------------------------------------------------------------------------------------------------GATAAGAAGGAGCCGTAAGGT------------------------------------------      0.049      0.000      0.000
-------------------------------------------------------------------------------------------------------------------------------------ATAAGAAGGAGCCGTAAGGT------------------------------------------      0.389      0.114      0.301
-------------------------------------------------------------------------------------------------------------------------------------ATAAGAAGGAGCCGTAAGGTA-----------------------------------------      0.097      0.038      0.038
--------------------------------------------------------------------------------------------------------------------------------------TAAGAAGGAGCCGTAAGGTAC----------------------------------------      0.534      0.076      0.076
--------------------------------------------------------------------------------------------------------------------------------------TAAGAAGGAGCCGTAAGG-------------------------------------------      3.837     11.934     36.785
--------------------------------------------------------------------------------------------------------------------------------------TAAGAAGGAGCCGTAAGGTACC---------------------------------------      0.049      0.000      0.000
--------------------------------------------------------------------------------------------------------------------------------------TAAGAAGGAGCCGTAAGGT------------------------------------------      9.179      3.448      8.697
--------------------------------------------------------------------------------------------------------------------------------------TAAGAAGGAGCCGTAAG--------------------------------------------      2.137      7.539     17.432
--------------------------------------------------------------------------------------------------------------------------------------TAAGAAGGAGCCGTAAGGTA-----------------------------------------      0.923      0.682      1.355
---------------------------------------------------------------------------------------------------------------------------------------AAGAAGGAGCCGTAAGGT------------------------------------------      0.049      0.075      0.075
---------------------------------------------------------------------------------------------------------------------------------------AAGAAGGAGCCGTAAGGTA-----------------------------------------      0.049      0.000      0.000
------------------------------------------------------------------------------------------------------------------------------------------------CCGTAAGGTACCGGGCGTGGGGA----------------------------      0.049      0.000      0.000
---------------------------------------------------------------------------------------------------------------------------------------------------TAAGGTACCGGGCGTGGGGA----------------------------      0.389      0.227      0.414
---------------------------------------------------------------------------------------------------------------------------------------------------TAAGGTACCGGGCGTGGGGAG---------------------------     15.153      2.425      3.953
---------------------------------------------------------------------------------------------------------------------------------------------------TAAGGTACCGGGCGTGGGG-----------------------------      1.603      0.303      1.581
---------------------------------------------------------------------------------------------------------------------------------------------------TAAGGTACCGGGCGTGGGGAGG--------------------------     87.714     93.617     65.701
---------------------------------------------------------------------------------------------------------------------------------------------------TAAGGTACCGGGCGTGGG------------------------------      0.146      0.265      1.054
---------------------------------------------------------------------------------------------------------------------------------------------------TAAGGTACCGGGCGTGG-------------------------------      0.146      0.076      0.113
---------------------------------------------------------------------------------------------------------------------------------------------------TAAGGTACCGGGCGTGGGGAGGGC------------------------      0.049      0.417      0.301
---------------------------------------------------------------------------------------------------------------------------------------------------TAAGGTACCGGGCGTGGGGAGGG-------------------------      0.097      0.038      0.038
---------------------------------------------------------------------------------------------------------------------------------------------------TAAGGTACCGGGCGTGGGGAGGGCA-----------------------      0.049      0.076      0.527
----------------------------------------------------------------------------------------------------------------------------------------------------AAGGTACCGGGCGTGGGGAG---------------------------      0.049      0.038      0.038
----------------------------------------------------------------------------------------------------------------------------------------------------AAGGTACCGGGCGTGGGGAGG--------------------------      0.534      0.265      0.602
-----------------------------------------------------------------------------------------------------------------------------------------------------AGGTACCGGGCGTGGGGAGGGCAG----------------------      0.049      0.000      0.000
-----------------------------------------------------------------------------------------------------------------------------------------------------AGGTACCGGGCGTGGGGAG---------------------------      0.049      0.000      0.000
-----------------------------------------------------------------------------------------------------------------------------------------------------AGGTACCGGGCGTGGGGAGG--------------------------      0.291      0.151      0.151
-------------------------------------------------------------------------------------------------------------------------------------------------------GTACCGGGCGTGGGGAGGGCAGG---------------------      0.243      0.038      0.038
--------------------------------------------------------------------------------------------------------------------------------------------------------TACCGGGCGTGGGGAGGGCAGG---------------------  43557.005  34052.471  45758.810 miR_t13
--------------------------------------------------------------------------------------------------------------------------------------------------------TACCGGGCGTGGGGA----------------------------     48.665      0.000      0.000
--------------------------------------------------------------------------------------------------------------------------------------------------------TACCGGGCGTGGGGAGGGCAGGGG-------------------      1.020      0.833      0.640
--------------------------------------------------------------------------------------------------------------------------------------------------------TACCGGGCGTGGGGAG---------------------------    107.578      0.000      0.000
--------------------------------------------------------------------------------------------------------------------------------------------------------TACCGGGCGTGGGGAGGGCAGGG--------------------     94.708     69.938     77.975
```

```
--------------------------------------------------------------------------------------------------------------------------------------------------------TACCGGGCGTGGGGAGGGCAGGGGC------------------      0.097      0.038      0.038
--------------------------------------------------------------------------------------------------------------------------------------------------------TACCGGGCGTGGGGAGGGC------------------------     32.492     46.789    134.451
--------------------------------------------------------------------------------------------------------------------------------------------------------TACCGGGCGTGGGGAGG--------------------------     99.516     37.128    104.406
--------------------------------------------------------------------------------------------------------------------------------------------------------TACCGGGCGTGGGGAGGGCA-----------------------     54.736     70.392    273.646
--------------------------------------------------------------------------------------------------------------------------------------------------------TACCGGGCGTGGGGAGGGCAG----------------------   1172.921   1310.215   2422.416
--------------------------------------------------------------------------------------------------------------------------------------------------------TACCGGGCGTGGGGAGGG-------------------------     23.604     34.779    110.806
---------------------------------------------------------------------------------------------------------------------------------------------------------ACCGGGCGTGGGGAGGGCAGGG--------------------      1.166      0.455      0.565
---------------------------------------------------------------------------------------------------------------------------------------------------------ACCGGGCGTGGGGAGGGCAG----------------------     10.102      6.441     28.125
---------------------------------------------------------------------------------------------------------------------------------------------------------ACCGGGCGTGGGGAGGGCA-----------------------      0.437      0.606      4.669
---------------------------------------------------------------------------------------------------------------------------------------------------------ACCGGGCGTGGGGAGGGCAGG---------------------    380.920    158.818    510.206
---------------------------------------------------------------------------------------------------------------------------------------------------------ACCGGGCGTGGGGAGGGC------------------------      0.194      0.265      2.108
----------------------------------------------------------------------------------------------------------------------------------------------------------CCGGGCGTGGGGAGGGCAG----------------------      1.700      0.265      2.071
----------------------------------------------------------------------------------------------------------------------------------------------------------CCGGGCGTGGGGAGGGC------------------------      0.049      0.301      0.301
----------------------------------------------------------------------------------------------------------------------------------------------------------CCGGGCGTGGGGAGGGCA-----------------------      0.049      0.113      0.113
----------------------------------------------------------------------------------------------------------------------------------------------------------CCGGGCGTGGGGAGGGCAGGG--------------------      0.194      0.151      0.151
----------------------------------------------------------------------------------------------------------------------------------------------------------CCGGGCGTGGGGAGGGCAGG---------------------     57.845      8.600     36.672
-----------------------------------------------------------------------------------------------------------------------------------------------------------CGGGCGTGGGGAGGGCAGG---------------------      0.826      0.227      0.038
------------------------------------------------------------------------------------------------------------------------------------------------------------GGGCGTGGGGAGGGCAGG---------------------      0.049      0.152      0.152

-------------------------------------------------------------------------------------------------------------------------------A                                                                GG
                                                                                                                                TGCCGATAAGAAGGAGCCGTAAGGTACCGGGCGTGGGGAGGGCAGGGGCAGGGACGGGGATCAG  G
                                                                                                                                ACGGCTATTCTTCCTCGGCATTCCATGGCCCGCGCCCCTCCCGTCCCCGTCCCTGCCCCTAGTC  G
TCGGCAGTCTACTGTCGTTGATGTATGTGAGGGAACGGCCGGGTACGAAGATCGTCGTATGATACTCGGACGGAGCCGTTGGGGGTTCTGGGAAAGGTGAGAGCCAAGAGGTCTCGGGCCTTTGGGAC                                                                GG

-----------------------------------------------------------------------------------------------------------------------------------------------------------------------CCCGTCCCCGTCCCTGCCCCT-------      0.097      0.114      0.188
----------------------------------------------------------------------------------------------------------------------------------------------------------------------TCCCGTCCCCGTCCCTGCCCCT-------      0.049      0.000      0.000
-----------------------------------------------------------------------------------------------------------------------------------------------------------------GCCCCTCCCGTCCCCGTCCCT-------------      0.291      0.038      0.075
----------------------------------------------------------------------------------------------------------------------------------------------------------GGCCCGCGCCCCTCCCGT-----------------------      0.146      0.075      0.075
----------------------------------------------------------------------------------------------------------------------------------------------------TTCCATGGCCCGCGCCCCTCCCGT-----------------------      0.291      0.000      0.000
--------------------------------------------------------------------------------------------------------------------------------------------------------ATGGCCCGCGCCCCTCCCGT-----------------------      0.437      0.189      0.226
------------------------------------------------------------------------------------------------------------------------------------------------------CCATGGCCCGCGCCCCTCCCGT-----------------------     42.449      3.713      1.318 miR_t13*
-------------------------------------------------------------------------------------------------------------------------------------------------------CATGGCCCGCGCCCCTCCCGT-----------------------     17.679      1.250      1.732
---------------------------------------------------------------------------------------------------------------------------------------------------------TGGCCCGCGCCCCTCCCGT-----------------------      0.097      0.038      0.038
-----------------------------------------------------------------------------------------------------------------------------------------------------TCCATGGCCCGCGCCCCTCCCGT-----------------------      2.331      0.038      0.226
------------------------------------------------------------------------------------------------------------------------------------------------------CCATGGCCCGCGCCCCTCCCG------------------------      0.340      0.076      0.038
-------------------------------------------------------------------------------------------------------------------------------------------------------CATGGCCCGCGCCCCTCCCG------------------------      0.146      0.038      0.038
-----------------------------------------------------------------------------------------------------------------------------------------------------TCCATGGCCCGCGCCCCTCCCG------------------------      0.049      0.000      0.000
------------------------------------------------------------------------------------------------------------------------------------------------------CCATGGCCCGCGCCCCTCC--------------------------      0.146      0.000      0.000
--------------------------------------------------------------------------------------------------------------------------------------------------CATTCCATGGCCCGCGCCCCT----------------------------      0.049      0.000      0.000
-------------------------------------------------------------------------------------------------------------------------------------TATTCTTCCTCGGCATTCCAT-----------------------------------------      3.011      0.417      0.188
------------------------------------------------------------------------------------------------------------------------------------CTATTCTTCCTCGGCATTCCAT-----------------------------------------      0.097      0.000      0.000
--------------------------------------------------------------------------------------------------------------------------------------ATTCTTCCTCGGCATTCCAT-----------------------------------------      4.905      0.417      0.188
-------------------------------------------------------------------------------------------------------------------------------------TATTCTTCCTCGGCATTCCA------------------------------------------      0.097      0.000      0.000
--------------------------------------------------------------------------------------------------------------------------------------ATTCTTCCTCGGCATTCCA------------------------------------------      0.049      0.000      0.000
--------------------------------------------------------------------------------------------------------------------------------------ATTCTTCCTCGGCATTCC-------------------------------------------      0.049      0.000      0.000
-----------------------------------------------------------------------------------------------------------------------------------GCTATTCTTCCTCGGCATTCC-------------------------------------------      0.049      0.000      0.000
---------------------------------------------------------------------------------------------------------------------------------CGGCTATTCTTCCTCGGCATTC--------------------------------------------     19.816     11.366     17.206
-----------------------------------------------------------------------------------------------------------------------------------GCTATTCTTCCTCGGCATTC--------------------------------------------      0.146      0.114      0.452
--------------------------------------------------------------------------------------------------------------------------------ACGGCTATTCTTCCTCGGCATTC--------------------------------------------      0.097      0.000      0.000
----------------------------------------------------------------------------------------------------------------------------------GGCTATTCTTCCTCGGCATTC--------------------------------------------      1.068      1.250      2.560
------------------------------------------------------------------------------------------------------------------------------------CTATTCTTCCTCGGCATT---------------------------------------------      5.391      5.569     11.069
--------------------------------------------------------------------------------------------------------------------------TGGGACACGGCTATTCTTCCTCGGCATT---------------------------------------------      0.049      0.076      0.076
--------------------------------------------------------------------------------------------------------------------------------ACGGCTATTCTTCCTCGGCATT---------------------------------------------    254.691     90.472     67.583
------------------------------------------------------------------------------------------------------------------------------ACACGGCTATTCTTCCTCGGCATT---------------------------------------------      0.097      0.114      0.114
-----------------------------------------------------------------------------------------------------------------------------------GCTATTCTTCCTCGGCATT---------------------------------------------    188.299    170.260    342.698
-------------------------------------------------------------------------------------------------------------------------TTGGGACACGGCTATTCTTCCTCGGCATT---------------------------------------------      0.049      0.000      0.000
---------------------------------------------------------------------------------------------------------------------------------CGGCTATTCTTCCTCGGCATT---------------------------------------------  31466.541  27971.867  39879.597 miR_t109
-------------------------------------------------------------------------------------------------------------------------------CACGGCTATTCTTCCTCGGCATT---------------------------------------------      0.389      0.152      0.075
-----------------------------------------------------------------------------------------------------------------------------GACACGGCTATTCTTCCTCGGCATT---------------------------------------------      0.583      0.303      0.113
-------------------------------------------------------------------------------------------------------------------------------------TATTCTTCCTCGGCATT---------------------------------------------      0.194      0.000      0.000
----------------------------------------------------------------------------------------------------------------------------------GGCTATTCTTCCTCGGCATT---------------------------------------------   1343.493   2005.841   3034.317
--------------------------------------------------------------------------------------------------------------------------------ACGGCTATTCTTCCTCGGCAT----------------------------------------------     14.910      3.145      2.861
-----------------------------------------------------------------------------------------------------------------------------------GCTATTCTTCCTCGGCAT----------------------------------------------      1.797      1.402      4.480
----------------------------------------------------------------------------------------------------------------------------------GGCTATTCTTCCTCGGCAT----------------------------------------------     22.001     34.022     57.417
------------------------------------------------------------------------------------------------------------------------------------CTATTCTTCCTCGGCAT----------------------------------------------      0.049      0.038      0.113
---------------------------------------------------------------------------------------------------------------------------------CGGCTATTCTTCCTCGGCAT----------------------------------------------    633.912    369.200    671.992
----------------------------------------------------------------------------------------------------------------------------------GGCTATTCTTCCTCGGCA-----------------------------------------------     15.590      1.705      5.271
--------------------------------------------------------------------------------------------------------------------------------ACGGCTATTCTTCCTCGGCA-----------------------------------------------      0.534      0.189      0.226
---------------------------------------------------------------------------------------------------------------------------------CGGCTATTCTTCCTCGGCA-----------------------------------------------     72.318     16.784     78.991
-----------------------------------------------------------------------------------------------------------------------------------GCTATTCTTCCTCGGCA-----------------------------------------------      0.437      0.565      0.565
----------------------------------------------------------------------------------------------------------------------------------GGCTATTCTTCCTCGGC------------------------------------------------      0.583      0.682      1.883
--------------------------------------------------------------------------------------------------------------------------------ACGGCTATTCTTCCTCGGC------------------------------------------------      0.049      0.000      0.000
---------------------------------------------------------------------------------------------------------------------------------CGGCTATTCTTCCTCGGC------------------------------------------------      0.243      0.076      0.075
----------------------------------------------------------------------------------------------------------------------------------GGCTATTCTTCCTCGG-------------------------------------------------      1.263      0.000      0.000
---------------------------------------------------------------------------------------------------------------------------------CGGCTATTCTTCCTCGG-------------------------------------------------      0.146      0.076      0.038
--------------------------------------------------------------------------------------------------------------------------------ACGGCTATTCTTCCTCGG-------------------------------------------------      0.049      0.000      0.000
----------------------------------------------------------------------------------------------------------------------------------GGCTATTCTTCCTCG--------------------------------------------------      7.091      0.000      0.000
----------------------------------------------------------------------------------------------------------------------------------GGCTATTCTTCCTC---------------------------------------------------      2.186      0.000      0.000
---------------------------------------------------------------------------------------------------------------------------------CGGCTATTCTTCCTC---------------------------------------------------      0.243      0.000      0.000
-------------------------------------------------------------------------------------------------------------------GGGCCTTTGGGACACGGCT-------------------------------------------------------------      0.049      0.000      0.000
--------------------------------------------------------------------------------------------------------CAAGAGGTCTCGGGCCTTT------------------------------------------------------------------------      0.243      0.000      0.000
----------------------------------------------------------------------------------------------------------AGAGGTCTCGGGCCTTT------------------------------------------------------------------------      0.097      0.000      0.000
----------------------------------------------------------------------------------------------------GAGCCAAGAGGTCTCGGGCCTTT------------------------------------------------------------------------      0.146      0.000      0.000
------------------------------------------------------------------------------------------------------GCCAAGAGGTCTCGGGCCTTT------------------------------------------------------------------------      0.097      0.000      0.000
---------------------------------------------------------------------------------------------------AGAGCCAAGAGGTCTCGGGCCTTT------------------------------------------------------------------------      0.097      0.076      0.076
-------------------------------------------------------------------------------------------------------CCAAGAGGTCTCGGGCCTTT------------------------------------------------------------------------      0.049      0.113      0.113
--------------------------------------------------------------------------------------------------------CAAGAGGTCTCGGGCCTT-------------------------------------------------------------------------      0.097      0.000      0.000
---------------------------------------------------------------------------------------------------AGAGCCAAGAGGTCTCGGGCCTT-------------------------------------------------------------------------      0.097      0.038      0.038
----------------------------------------------------------------------------------------------------GAGCCAAGAGGTCTCGGGCCTT-------------------------------------------------------------------------      0.049      0.000      0.000
--------------------------------------------------------------------GACGGAGCCGTTGGGGGTT------------------------------------------------------------------------------------------------------------      0.049      0.000      0.000
------------------------------------GGCCGGGTACGAAGATCGTCGTAT---------------------------------------------------------------------------------------------------------------------------------------      0.389      0.188      0.188
---------------------------------AACGGCCGGGTACGAAGATCGTCGTAT---------------------------------------------------------------------------------------------------------------------------------------      0.049      0.000      0.000
-----------------------------------CGGCCGGGTACGAAGATCGTCGTAT---------------------------------------------------------------------------------------------------------------------------------------      0.049      0.038      0.038
----CAGTCTACTGTCGTTGAT-----------------------------------------------------------------------------------------------------------------------------------------------------------------------------      0.097      0.000      0.000

miRNA: miR_t54,miR_t50,miR_t15 chromosome_5 Start: 935240 End: 935510 Condition: TAP
-----TACGAGACGTCTGCACCGCAC----------------------------------------------------------------------------------------------------------------      0.049      0.000      0.000
---------AGACGTCTGCACCGCACATG-------------------------------------------------------------------------------------------------------------      0.049      0.189      0.075
--------------TCTGCACCGCACATGTCTCAG-------------------------------------------------------------------------------------------------------      3.011      0.075      0.075
--------------TCTGCACCGCACATGTCTCAGG------------------------------------------------------------------------------------------------------      0.923      0.000      0.000
--------------TCTGCACCGCACATGTCTCA--------------------------------------------------------------------------------------------------------      0.534      0.000      0.000
--------------TCTGCACCGCACATGTCTC---------------------------------------------------------------------------------------------------------      0.049      0.000      0.000
----------------TGCACCGCACATGTCTCAGG------------------------------------------------------------------------------------------------------      2.186      0.303      0.452
----------------TGCACCGCACATGTCTCAG-------------------------------------------------------------------------------------------------------      0.049      0.000      0.000
---------------------------TGTCTCAGGAAGAGATCGTC-------------------------------------------------------------------------------------------      0.631      0.076      0.113
-----------------------------TCTCAGGAAGAGATCGTC-CG----------------------------------------------------------------------------------------      0.146      0.303      0.151
-----------------------------TCTCAGGAAGAGATCGTC-CGTAGT------------------------------------------------------------------------------------      2.380      3.789      0.527
-----------------------------TCTCAGGAAGAGATCGTC-CGTA--------------------------------------------------------------------------------------      0.437      1.099      1.092
-----------------------------TCTCAGGAAGAGATCGTC-CGTAG-------------------------------------------------------------------------------------     21.321     30.385     23.720 miR_t54
-----------------------------TCTCAGGAAGAGATCGTC-C-----------------------------------------------------------------------------------------      0.243      0.720      0.264
-----------------------------TCTCAGGAAGAGATCGTC-CGT---------------------------------------------------------------------------------------      2.477      1.781      1.506
------------------------------CTCAGGAAGAGATCGTC-CGTAG-------------------------------------------------------------------------------------      0.049      0.152      0.151
-------------------------------TCAGGAAGAGATCGTC-CGTAG-------------------------------------------------------------------------------------      0.486      0.682      0.791
-------------------------------TCAGGAAGAGATCGTC-CGTAGT------------------------------------------------------------------------------------      0.146      0.113      0.113
-----------------------------------------------------TAGCAAGCTGCTGGAGGGTA-----------------------------------------------------------------      0.097      0.076      0.113
---------------------------------------------------------AAGCTGCTGGAGGGT------------------------------------------------------------------      0.631      0.000      0.000
----------------------------------------------------------------TGGAGGGTATCTCGGTCAGC------------------------------------------------------      0.097      0.000      0.000
---------------------------------------------------------------------------TCGGTCAGCATCTCGATTG--------------------------------------------      0.097      0.000      0.000
---------------------------------------------------------------------------TCGGTCAGCATCTCGATTGGC------------------------------------------   1009.635      0.000      0.000 miR_t50
---------------------------------------------------------------------------TCGGTCAGCATCTCGATTGGCA-----------------------------------------      0.243      0.000      0.000
---------------------------------------------------------------------------TCGGTCAGCATCTCGATTGG-------------------------------------------      7.868      0.000      0.000
----------------------------------------------------------------------------CGGTCAGCATCTCGATTGGC------------------------------------------      6.071      0.000      0.000
----------------------------------------------------------------------------CGGTCAGCATCTCGATTGG-------------------------------------------      0.097      0.000      0.000
-----------------------------------------------------------------------------GGTCAGCATCTCGATTGG-------------------------------------------      0.049      0.000      0.000
-----------------------------------------------------------------------------GGTCAGCATCTCGATTGGC------------------------------------------      1.846      0.000      0.000
-----------------------------------------------------------------------------GGTCAGCATCTCGATT---------------------------------------------      0.049      0.000      0.000
-------------------------------------------------------------------------------TCAGCATCTCGATTGGCAAG---------------------------------------      9.811      0.000      0.000
-------------------------------------------------------------------------------TCAGCATCTCGATTGGCAAGA--------------------------------------      0.194      0.000      0.000
-------------------------------------------------------------------------------TCAGCATCTCGATTGGCAAGACG------------------------------------      0.097      0.000      0.000
---------------------------------------------------------------------------------------TCGATTGGCAAGACGTCCACT------------------------------      0.097      0.038      0.038
-------------------------------------------------------------------------------------------TTGGCAAGACGTCCACTCCGG--------------------------      0.194      0.076      0.075
-------------------------------------------------------------------------------------------TTGGCAAGACGTCCACTCCGGG-------------------------      1.408      0.871      1.092
--------------------------------------------------------------------------------------------TGGCAAGACGTCCACTCCGG--------------------------      0.049      0.000      0.000
--------------------------------------------------------------------------------------------TGGCAAGACGTCCACTCCGGGT------------------------      0.049      0.000      0.000
--------------------------------------------------------------------------------------------TGGCAAGACGTCCACTCCGGG-------------------------      0.631      0.265      0.753
-----------------------------------------------------------------------------------------------CAAGACGTCCACTCCGGGTAC----------------------      0.243      0.038      0.264
-----------------------------------------------------------------------------------------------CAAGACGTCCACTCCGGGTACG---------------------      0.049      0.000      0.000
------------------------------------------------------------------------------------------------AAGACGTCCACTCCGGGTACG---------------------      0.097      0.038      0.151
------------------------------------------------------------------------------------------------------TCCACTCCGGGTACGCGCCGC---------------      0.194      0.038      0.151
------------------------------------------------------------------------------------------------------TCCACTCCGGGTACGCGCCGCT--------------      0.097      0.000      0.000

-     A  A         A                 A  G      - GTAG            GG                   C   A         C    A  C                           CG
 CCAAT CG GACGTCTGC CCGCACATGTCTCAGGA GA ATCGTC C    TAGCAAGCTGCT  AGGGTATCTCGGTCAGCAT TCG TTGGCAAGA GTCC CT CGGGTACGCGCCGCTCATGCGATGA  C
 GGTTA GC CTGCAGACG GGCGTGTACAGAGTCCT CT TAGCGG G    ATCGTTCGATGA  TCCCATAGAGCCAGTCGTA AGC AACCGTTCT CAGG GA GTCCATGTGCGGCGAGTGCGCTACT  /
T     C  C         C                 C  G      T ATA-            AG                   A   A         A    C  A                           TT

----------------------------------------------------------------------------------------------------------GAAGTCCATGTGCGGCGAGT------------      0.777      0.076      0.113
---------------------------------------------------------------------------------------------------------CGAAGTCCATGTGCGGCGAGT------------      0.194      0.227      0.151
------------------------------------------------------------------------------------------------TTCTACAGGCGAAGTCCAT-----------------------      0.049      0.076      0.226
----------------------------------------------------------------------------------------------CGTTCTACAGGCGAAGTCCAT-----------------------    225.308    278.652    484.491 miR_t15
---------------------------------------------------------------------------------------------CCGTTCTACAGGCGAAGTCCAT-----------------------     20.399      7.577      1.920
--------------------------------------------------------------------------------------------ACCGTTCTACAGGCGAAGTCCAT-----------------------      0.097      0.114      0.114
-----------------------------------------------------------------------------------------------GTTCTACAGGCGAAGTCCAT-----------------------      6.411      2.576      3.125
----------------------------------------------------------------------------------------------CGTTCTACAGGCGAAGTCCA------------------------      1.797      2.046      6.062
---------------------------------------------------------------------------------------------CCGTTCTACAGGCGAAGTCCA------------------------      0.097      0.076      0.075
----------------------------------------------------------------------------------------------CGTTCTACAGGCGAAGTCC-------------------------      0.437      0.152      0.602
-----------------------------------------------------------------------------------------CAAACCGTTCTACAGGCGAAGT---------------------------      0.146      0.000      0.000
----------------------------------------------------------------------------------------GCAAACCGTTCTACAGGCGAAGT---------------------------      0.049      0.114      0.114
--------------------------------------------------------------------------------GTCGTAAAGCAAACCGTTCT--------------------------------------      0.097      0.000      0.000
-------------------------------------------------------------------------------AGTCGTAAAGCAAACCGTTCT--------------------------------------      0.826      0.075      0.075
--------------------------------------------------------------------------GAGCCAGTCGTAAAGCAAACCGT-----------------------------------------      0.049      0.000      0.000
----------------------------------------------------------------------------GCCAGTCGTAAAGCAAACCGT-----------------------------------------      2.526      0.265      0.339
----------------------------------------------------------------------------GCCAGTCGTAAAGCAAACCG------------------------------------------      0.049      0.038      0.038
-------------------------------------------------------------------------AGAGCCAGTCGTAAAGCAAAC--------------------------------------------      0.194      0.000      0.000 miR_t50*
------------------------------------------------------------------------TAGAGCCAGTCGTAAAGCAAA---------------------------------------------      0.049      0.000      0.000
-------------------------------------------------------------------------AGAGCCAGTCGTAAAGCAAA---------------------------------------------      0.340      0.565      0.565
```

```
-----------------------------------------------------------------------ATAGAGCCAGTCGTAAAGCAA---------------------------------------------    0.097    0.000    0.000
--------------------------------------------------------------------CCCATAGAGCCAGTCGTAAAG------------------------------------------------    0.049    0.000    0.000
------------------------------------------------------------------GTCCCATAGAGCCAGTCGTAAA-------------------------------------------------    0.049    0.000    0.000
-----------------------------------------------------------------AGTCCCATAGAGCCAGTCGTAA--------------------------------------------------    0.971    1.743    3.502
---------------------------------------------------------------GAAGTCCCATAGAGCCAGTCGT----------------------------------------------------    0.777    2.008    2.008
-----------------------------------------------------------------AGTCCCATAGAGCCAGTCGT----------------------------------------------------    1.554    0.796    0.602
----------------------------------------------------------------AAGTCCCATAGAGCCAGTCGT----------------------------------------------------    0.680    0.379    0.379
----------------------------------------------------------------AAGTCCCATAGAGCCAGTCG-----------------------------------------------------    0.049    0.000    0.000
-------------------------------------------------------------ATGAAGTCCCATAGAGCCAGT-------------------------------------------------------    0.777    0.644    1.092
-----------------------------------------------------------CGATGAAGTCCCATAGAGCCAGT-------------------------------------------------------    0.049    0.152    0.152
-------------------------------------------------------CGTTCGATGAAGTCCCATAGA-------------------------------------------------------------    0.049    0.000    0.000
---------------------------------------------------A-ATCGTTCGATGAAGTCCCAT----------------------------------------------------------------    1.748    0.113    0.113
--------------------------------------------------TA-ATCGTTCGATGAAGTCCCAT----------------------------------------------------------------    0.049    0.000    0.000
------------------------------------------------------TCGTTCGATGAAGTCCCAT----------------------------------------------------------------    0.049    0.038    0.038
--------------------------TACAGAGTCCTCCTGTAGCGGTGAT--------------------------------------------------------------------------------------    0.049    0.000    0.000
----------------------------CAGAGTCCTCCTGTAGCGGTGAT--------------------------------------------------------------------------------------    0.049    0.227    0.227
-----------------------------AGAGTCCTCCTGTAGCGGTGAT--------------------------------------------------------------------------------------    0.340    0.038    0.038
---------------------------ACAGAGTCCTCCTGTAGCGGT-----------------------------------------------------------------------------------------    0.097    0.000    0.000
--------------------GGCGTGTACAGAGTCCTCCTGT-----------------------------------------------------------------------------------------------    3.691    0.530    0.301
-----------------CGCGGCGTGTACAGAGTCCTCCT-------------------------------------------------------------------------------------------------    0.291    0.114    0.114
--------------------GGCGTGTACAGAGTCCTCCT-------------------------------------------------------------------------------------------------    0.340    0.000    0.000
------------------GCGGCGTGTACAGAGTCCTCCT-------------------------------------------------------------------------------------------------    0.097    0.000    0.000
-------------------CGGCGTGTACAGAGTCCTCCT-------------------------------------------------------------------------------------------------    0.146    0.226    0.226
-------------------CGGCGTGTACAGAGTCCTCC--------------------------------------------------------------------------------------------------    0.049    0.000    0.000
-----------------CGCGGCGTGTACAGAGTCCTC---------------------------------------------------------------------------------------------------    0.291    0.114    0.038
----------------ACGCGGCGTGTACAGAGTCCT----------------------------------------------------------------------------------------------------    4.614    0.644    1.318
---------------GACGCGGCGTGTACAGAGT-------------------------------------------------------------------------------------------------------    0.049    0.038    0.038
------------GCAGACGCGGCGTGTACAGAGT-------------------------------------------------------------------------------------------------------   21.370   14.889   16.152
--------------AGACGCGGCGTGTACAGAGT-------------------------------------------------------------------------------------------------------    1.554    0.379    1.092
----------------ACGCGGCGTGTACAGAGT-------------------------------------------------------------------------------------------------------    0.097    0.000    0.000
------------GCAGACGCGGCGTGTACAGAG--------------------------------------------------------------------------------------------------------    0.049    0.038    0.113
----TACGCCCTGCAGACGCGGCGTGT--------------------------------------------------------------------------------------------------------------    0.049    0.000    0.000
-------GCCCTGCAGACGCGGCGTGT--------------------------------------------------------------------------------------------------------------    8.985    0.114    0.151
--------CCCTGCAGACGCGGCGTGT--------------------------------------------------------------------------------------------------------------    0.389    0.038    0.038

miRNA: miR_t98,miR_t20 chromosome_6 Start: 6776059 End: 6776262 Condition: HS-N
---------------------------------------------------------------------------------------TACCCGAACGTCGAGTTCAAG----      0.291      0.152      0.152
-------------------------------------------------------------------------------TCGAGCTCTACCCGAACGTCG------------      1.360      0.038      0.075
----------------------------------------------------------------------------CCTTCGAGCTCTACCCG-------------------      0.000      0.114      0.038
---------------------------------------------------------------------------ACCTTCGAGCTCTACCCGAACG---------------      0.049      0.076      0.076
-------------------------------------------------------------------------AGACCTTCGAGCTCTACCCG-------------------      0.340      0.644      0.753
------------------------------------------------------------------------AAGACCTTCGAGCTCTACCC--------------------      0.000      2.576      0.979
------------------------------------------------------------------------AAGACCTTCGAGCTCTACCCG-------------------      9.665     51.070     37.726
-----------------------------------------------------------------------CAAGACCTTCGAGCTCTAC----------------------      0.000      0.038      0.038
-----------------------------------------------------------------------CAAGACCTTCGAGCTCTACC---------------------      0.146      0.341      0.151
-----------------------------------------------------------------------CAAGACCTTCGAGCTCTACCCG-------------------      0.097      0.076      0.076
----------------------------------------------------------------------CCAAGACCTTCGAGCTCTACC---------------------      3.594      7.198      4.066
----------------------------------------------------------------------CCAAGACCTTCGAGCTCTACCC--------------------      0.146      0.114      0.114
----------------------------------------------------------------------CCAAGACCTTCGAGCTCTA-----------------------      0.194      0.114      0.151
----------------------------------------------------------------------CCAAGACCTTCGAGCTCTAC----------------------      1.846      0.568      1.393
---------------------------------------------------------------------CCCAAGACCTTCGAGCTCTACC---------------------      1.020      6.895      3.426
---------------------------------------------------------------------CCCAAGACCTTCGAGCTCTACCC--------------------      0.000      0.303      0.303
---------------------------------------------------------------------CCCAAGACCTTCGAGCTCTAC----------------------      0.049      0.114      0.075
--------------------------------------------------------------------TCCCAAGACCTTCGAGCTCTACCC--------------------      1.214      5.759      0.151
--------------------------------------------------------------------TCCCAAGACCTTCGAGCTCTACCCG-------------------      0.097      0.076      0.076
--------------------------------------------------------------------TCCCAAGACCTTCGAGCTCTA-----------------------      1.166      0.909      1.694
--------------------------------------------------------------------TCCCAAGACCTTCGAGCTCT------------------------      0.291      0.682      0.339
--------------------------------------------------------------------TCCCAAGACCTTCGAGCTCTAC----------------------     24.381     49.631     62.124
--------------------------------------------------------------------TCCCAAGACCTTCGAGCTCTACC---------------------     25.595     61.603     63.517
-------------------------------------------------------------------TTCCCAAGACCTTCGAGCTCTACC---------------------    232.496    322.562    225.190
-------------------------------------------------------------------TTCCCAAGACCTTCGAGCTCTACCCG-------------------      0.146      0.682      0.301
-------------------------------------------------------------------TTCCCAAGACCTTCGAGCTCT------------------------     12.919     18.185     13.291
-------------------------------------------------------------------TTCCCAAGACCTTCGAGC---------------------------      0.146      0.152      0.152
-------------------------------------------------------------------TTCCCAAGACCTTCGAGCTC-------------------------      1.020      0.114      0.301
-------------------------------------------------------------------TTCCCAAGACCTTCGAGCTCTAC----------------------    276.256    199.584    226.620
-------------------------------------------------------------------TTCCCAAGACCTTCGAGCTCTACCC--------------------      3.303      4.698      0.527
-------------------------------------------------------------------TTCCCAAGACCTTCGAGCTCTA-----------------------     28.704     42.281     62.877
------------------------------------------------------------------CTTCCCAAGACCTTCGAGCTCTACC---------------------      0.049      0.379      0.151
----------------------------------------------------------------TCCTTCCCAAGACCTTCGAGC---------------------------      0.097      0.076      0.151
--------------------------------------------------------GACCGTCCTCCTTCCCAAGA------------------------------------      0.000      0.076      0.076
-------------------------------------------------------TGACCGTCCTCCTTCCCAAGA------------------------------------      1.603      0.341      0.828
-------------------------------------------------------TGACCGTCCTCCTTCCCAAG-------------------------------------      6.654      2.387      4.029
-------------------------------------------------------TGACCGTCCTCCTTCCC----------------------------------------      0.049      0.076      0.075
---------------------------------------------------GGCGTGACCGTCCTCCTTCCC----------------------------------------      0.049      0.038      0.038
--------------------------------------------------GGGCGTGACCGTCCTCCTTCC-----------------------------------------      0.000      0.038      0.038
-------------------------------------------------TGGGCGTGACCGTCCTCCTTC------------------------------------------      8.548      2.311      2.861
-------------------------------------------------TGGGCGTGACCGTCCTCCTTCC-----------------------------------------      0.389      0.038      0.188
------------------------------------------------GTGGGCGTGACCGTCCTCCT--------------------------------------------      0.000      0.038      0.038
-----------------------------------------------TGTGGGCGTGACCGTCCTCCTTC------------------------------------------      0.000      0.038      0.038
-----------------------------------------------TGTGGGCGTGACCGTCCTCCT--------------------------------------------      0.000      0.076      0.113
----------------------------------------------TTGTGGGCGTGACCGTCCTCC---------------------------------------------      1.068      0.038      0.038
----------------------------------------------TTGTGGGCGTGACCGTC-------------------------------------------------      0.049      0.227      0.227
----------------------------------------------TTGTGGGCGTGACCGTCCTCCT--------------------------------------------      0.000      0.189      0.189
--------------------------------------------TGTTGTGGGCGTGACCGTCC------------------------------------------------      0.146      0.076      0.264
--------------------------------------------TGTTGTGGGCGTGACCGTC-------------------------------------------------      0.194      0.227      0.565
-------------------------------TGACCGACCCCGGTGTTG---------------------------------------------------------------      0.049      0.114      0.113
-------------------------------TGACCGACCCCGGTGTTGTG-------------------------------------------------------------      1.214      0.303      0.828
-------------------------------TGACCGACCCCGGTGTTGTGG------------------------------------------------------------     27.781      3.182      2.297
------------------------------ATGACCGACCCCGGTGTTG---------------------------------------------------------------      0.000      0.076      0.076
------------------------------ATGACCGACCCCGGTGTTGTG-------------------------------------------------------------      0.194      0.227      0.489
----------------------------ACATGACCGACCCCGGTGTTG---------------------------------------------------------------      0.194      0.379      0.715
------------------------TTGAACATGACCGACCCCGGT-------------------------------------------------------------------      0.000      0.038      0.038

--------------------CGCCTT                                                 AC                                 C
                          GAACATGACCGACCCCGGTGTTGTGGGCGTGACCGTCCTCCTTCCCAAG  CTTCGAGCTCTACCCGAACGTCGAGTTCAAGTA G
                          CTTGTACTGGCTGGGGCCACAACACCCGCACTGGCAGGAGGAAGGGTTC  GAAGCTCGAGATGGGCTTGCAGCTCAAGTTCAT /
GTGTCGTTTATGCCCAGGTTATACCT                                                 AA                                 -

----CGTTTATGCCCAGGTTATACCTCT------------------------------------------------------------------------------------      0.000      0.152      0.152
--------------------------------------------------CCCGCACTGGCAGGAGGAAGG-----------------------------------------      0.000      0.303      0.866
--------------------------------------------------CCCGCACTGGCAGGAGGAAGGG----------------------------------------      0.000      0.038      0.038
----------------------------------------------------CGCACTGGCAGGAGGAAGGG----------------------------------------      0.874      0.417      2.410
-----------------------------------------------------GCACTGGCAGGAGGAAGGGT---------------------------------------      1.894      2.311      3.502
----------------------------------------------------CGCACTGGCAGGAGGAAGGGT---------------------------------------     69.064     65.998    118.901 miR_t98
-------------------------------------------------------ACTGGCAGGAGGAAGGGT---------------------------------------      0.000      0.076      0.076
---------------------------------------------------CCGCACTGGCAGGAGGAAGGGT---------------------------------------      0.680      0.114      0.715
-----------------------------------------------------GCACTGGCAGGAGGAAGGGTT--------------------------------------      9.374      7.805     11.295
------------------------------------------------------CACTGGCAGGAGGAAGGGTT--------------------------------------      0.000      0.076      0.076
-------------------------------------------------------ACTGGCAGGAGGAAGGGTTCA------------------------------------      0.146      0.038      0.038
--------------------------------------------------------CTGGCAGGAGGAAGGGTTCAA-----------------------------------      0.000      0.076      0.038
-------------------------------------------------------ACTGGCAGGAGGAAGGGTTCAA-----------------------------------      0.777      0.417      0.527
----------------------------------------------------------GGCAGGAGGAAGGGTTCAAGAA--------------------------------      0.049      0.379      0.379
--------------------------------------------------------------GGAGGAAGGGTTCAAGAAG-------------------------------      0.631      2.235      1.054
--------------------------------------------------------------GGAGGAAGGGTTCAAGAAGC------------------------------      0.729      1.667      1.017
------------------------------------------------------------------GAAGGGTTCAAGAAGCT-----------------------------      0.243      0.076      0.113
-----------------------------------------------------------GCAGGAGGAAGGGTTCAAGAAGCT-----------------------------      0.000      0.493      0.493
-------------------------------------------------------------AGGAGGAAGGGTTCAAGAAGCT-----------------------------      0.534      1.099      0.038
--------------------------------------------------------------GGAGGAAGGGTTCAAGAAGCT-----------------------------    114.038    333.473    250.227
---------------------------------------------------------------GAGGAAGGGTTCAAGAAGCT-----------------------------      0.146      0.720      0.565
-----------------------------------------------------------------GGAAGGGTTCAAGAAGCT-----------------------------      1.797      0.909      1.318
-----------------------------------------------------------------GGAAGGGTTCAAGAAGCTC----------------------------      0.194      0.303      0.303
--------------------------------------------------------------GGAGGAAGGGTTCAAGAAGCTC----------------------------      0.000      0.076      0.151
------------------------------------------------------------------GAAGGGTTCAAGAAGCTC----------------------------      0.000      0.038      0.038
-----------------------------------------------------------------GGAAGGGTTCAAGAAGCTCG---------------------------      0.534      0.038      0.038
------------------------------------------------------------------GAAGGGTTCAAGAAGCTCG---------------------------      0.194      0.038      0.038
------------------------------------------------------------------GAAGGGTTCAAGAAGCTCGA--------------------------      0.437      0.227      0.264
----------------------------------------------------------------AGGAAGGGTTCAAGAAGCTCGA--------------------------      0.049      0.076      0.076
-----------------------------------------------------------------GGAAGGGTTCAAGAAGCTCGA--------------------------      3.545      1.932      0.038
-----------------------------------------------------------------GGAAGGGTTCAAGAAGCTCGAG-------------------------     15.493      8.562      0.565
--------------------------------------------------------------------AGGGTTCAAGAAGCTCGAG-------------------------     20.301      3.751      1.506
----------------------------------------------------------------------GGTTCAAGAAGCTCGAG-------------------------      0.049      0.038      0.264
----------------------------------------------------------------AGGAAGGGTTCAAGAAGCTCGAG-------------------------      0.049      0.076      0.076
------------------------------------------------------------------GAAGGGTTCAAGAAGCTCGAG-------------------------      4.177      4.433      0.188
---------------------------------------------------------------------GGGTTCAAGAAGCTCGAG-------------------------      0.729      0.606      2.485
------------------------------------------------------------------GAAGGGTTCAAGAAGCTCGAGA------------------------     37.349     70.771     10.053
-----------------------------------------------------------------GGAAGGGTTCAAGAAGCTCGAGA------------------------    239.538    267.930      7.417
--------------------------------------------------------------------AGGGTTCAAGAAGCTCGAGA------------------------    169.114     51.487     18.976
-------------------------------------------------------------------AAGGGTTCAAGAAGCTCGAGA------------------------      0.874      0.568      0.568
----------------------------------------------------------------------GGTTCAAGAAGCTCGAGA------------------------      0.534      2.614      5.233
---------------------------------------------------------------------GGGTTCAAGAAGCTCGAGA------------------------      6.848     17.162     30.422
---------------------------------------------------------------GAGGAAGGGTTCAAGAAGCTCGAGA------------------------      0.000      0.152      0.152
----------------------------------------------------------------AGGAAGGGTTCAAGAAGCTCGAGA------------------------      0.437      1.743      1.743
---------------------------------------------------------------------GGGTTCAAGAAGCTCGAGAT-----------------------    753.827   2008.796   2089.432
-----------------------------------------------------------------GGAAGGGTTCAAGAAGCTCGAGAT-----------------------  12983.972  15291.886    302.863 miR_t20
----------------------------------------------------------------AGGAAGGGTTCAAGAAGCTCGAGAT-----------------------     39.195    223.604      1.318
------------------------------------------------------------------------TTCAAGAAGCTCGAGAT-----------------------      1.117      0.720      1.845
-----------------------------------------------------------GCAGGAGGAAGGGTTCAAGAAGCTCGAGAT-----------------------      0.000      0.796      0.489
------------------------------------------------------------CAGGAGGAAGGGTTCAAGAAGCTCGAGAT-----------------------      0.000      0.379      0.188
--------------------------------------------------------------GGAGGAAGGGTTCAAGAAGCTCGAGAT-----------------------      0.097      2.804      0.075
---------------------------------------------------------------GAGGAAGGGTTCAAGAAGCTCGAGAT-----------------------      0.631     12.540      0.941
-----------------------------------------------------------------------GTTCAAGAAGCTCGAGAT-----------------------      1.360      0.796      1.355
-------------------------------------------------------------------AAGGGTTCAAGAAGCTCGAGAT-----------------------     53.182     48.305      2.485
------------------------------------------------------------------GAAGGGTTCAAGAAGCTCGAGAT-----------------------   5946.153  11810.306    112.350
--------------------------------------------------------------------AGGGTTCAAGAAGCTCGAGAT-----------------------  17626.944   7832.456   1552.757
----------------------------------------------------------------------GGTTCAAGAAGCTCGAGAT-----------------------     76.009    328.775    385.883
-------------------------------------------------------------AGGAGGAAGGGTTCAAGAAGCTCGAGAT-----------------------      0.000      0.189      0.188
----------------------------------------------------------------------GGTTCAAGAAGCTCGAGATGG---------------------      0.486      0.265      0.151
--------------------------------------------------------------------------CAAGAAGCTCGAGATGGGCT------------------      0.049      0.038      0.038
-------------------------------------------------------------------------TCAAGAAGCTCGAGATGGGCT------------------      0.049      0.379      0.264
-------------------------------------------------------------------------TCAAGAAGCTCGAGATGGGCTT-----------------      0.389      0.796      0.226
--------------------------------------------------------------------------CAAGAAGCTCGAGATGGGCTT-----------------      0.389      0.682      0.264
--------------------------------------------------------------------------------GCTCGAGATGGGCTTGCAGCT-----------      0.243      0.038      0.151
-----------------------------------------------------------------------------------CGAGATGGGCTTGCAGCTCAA--------      0.000      0.038      0.038
--------------------------------------------------------------------------------------GATGGGCTTGCAGCTCAAGT------      0.000      0.076      0.076

miRNA: miR_t97,miR_t26 chromosome_8 Start: 121853 End: 121958 Condition: TAP
```

```
------------------------TCGGTCCTAGGCGGCGCGTG------------      0.146      0.076      0.038
------------------------TCGGTCCTAGGCGGCGCGTGT-----------      0.049      0.038      0.038
-------------------TTTCGTCGGTCCTAGGCGGC-----------------      0.340      1.894      0.828
-------------------TTTCGTCGGTCCTAGG---------------------      0.049      0.000      0.000
----------------GGCTTTCGTCGGTCCTAGGC--------------------      0.049      0.038      0.038
----------------GGCTTTCGTCGGTCCTAG----------------------      0.049      0.000      0.000
----------------GGCTTTCGTCGGTCCTAGG---------------------      6.168      0.985      0.715
---------------TGGCTTTCGTCGGTCCTAGG---------------------   1283.074    119.947     89.948 miR_t97
---------------TGGCTTTCGTCGGTCCTAG----------------------     10.734      2.955      2.937
---------------TGGCTTTCGTCGGTCCTAGGC--------------------     19.282     15.533      4.405
-------------AGTGGCTTTCGTCGGTCCTAG----------------------      0.146      0.152      0.152
------------TAGTGGCTTTCGTCGGTCCTAGG---------------------      0.729      0.038      0.226
------------TAGTGGCTTTCGTCGGTCCTA-----------------------      0.146      0.076      0.076
------------TAGTGGCTTTCGTCGGT---------------------------      0.049      0.000      0.000
------------TAGTGGCTTTCGTCGGTCCTAG----------------------      0.049      0.038      0.038
-----------TTAGTGGCTTTCGTCGGTCCTAG----------------------      0.291      0.038      0.038
-----------TTAGTGGCTTTCGTCGGT---------------------------      0.049      0.038      0.038
-----------TTAGTGGCTTTCGTCGGTCCTAGG---------------------      0.194      0.000      0.000
-----------TTAGTGGCTTTCGTCGGTCCTA-----------------------      2.234      1.326      0.113
-----------TTAGTGGCTTTCGTCGGTCCT------------------------      0.631      1.212      0.038

-------                                             GAC
       CAGCTTAGTGGCTTTCGTCGGTCCTAGGCGGCGCGTGTGCGCGCG   G
       GTCGAATCACCGAAAGCAGCCAGGATCCGCCGCGCACACGCGCGC   A
AGCACTG                                             AAA

-------------TCACCGAAAGCAGCCAGG-------------------------      0.291      0.038      0.113
------------ATCACCGAAAGCAGCCAGG-------------------------      0.194      0.076      0.076
--------------CACCGAAAGCAGCCAGGA------------------------      0.049      0.000      0.000
-----------AATCACCGAAAGCAGCCAGGA------------------------      0.097      0.076      0.076
-----------------CGAAAGCAGCCAGGA------------------------      0.097      0.000      0.000
-------------TCACCGAAAGCAGCCAGGA------------------------      5.780      1.250      1.017
------------ATCACCGAAAGCAGCCAGGA------------------------      1.554      0.606      0.606
------GGTCGAATCACCGAAAGCAGCCAGGAT-----------------------      0.146      0.417      0.417
------------ATCACCGAAAGCAGCCAGGAT-----------------------     73.484     30.801     12.274
-------------TCACCGAAAGCAGCCAGGAT-----------------------    416.035    198.409    104.594 miR_t26
----------GAATCACCGAAAGCAGCCAGGAT-----------------------      0.389      0.644      0.301
-----------AATCACCGAAAGCAGCCAGGAT-----------------------      3.351      2.652      0.941
--------TCGAATCACCGAAAGCAGCCAGGAT-----------------------      0.097      0.152      0.075
--------------CACCGAAAGCAGCCAGGAT-----------------------      3.011      5.721      2.071
-----------------CGAAAGCAGCCAGGAT-----------------------      1.603      0.000      0.000
-------GTCGAATCACCGAAAGCAGCCAGGAT-----------------------      0.049      0.076      0.076
-----TGGTCGAATCACCGAAAGCAGCCAGGAT-----------------------      0.097      1.326      0.151
----CTGGTCGAATCACCGAAAGCAGCCAGGAT-----------------------      0.049      0.227      0.075
---------CGAATCACCGAAAGCAGCCAGGAT-----------------------      0.194      1.932      0.151
-------------------AAAGCAGCCAGGAT-----------------------      0.340      0.000      0.000
----------------CCGAAAGCAGCCAGGAT-----------------------      2.331      3.486      2.372
---------------ACCGAAAGCAGCCAGGAT-----------------------      0.049      0.379      0.038
------------------GAAAGCAGCCAGGAT-----------------------      0.291      0.000      0.000
-----------AATCACCGAAAGCAGCCAGGATC----------------------      0.049      0.000      0.000
-------------TCACCGAAAGCAGCCAGGATC----------------------      0.194      0.038      0.038

miRNA: miR_t29 chromosome_11 Start: 1448180 End: 1448276 Condition: TAP
-------TTAGTGCCAGCGCGCTCTCGG----------------------      2.574      0.189      0.038
-------TTAGTGCCAGCGCGCTCTCGGC---------------------      0.194      0.758      0.640
--------TAGTGCCAGCGCGCTCTCGGCC--------------------      1.603      1.023      0.264
--------TAGTGCCAGCGCGCTCTCGGC---------------------    145.899    662.135    408.963 miR_t29
--------TAGTGCCAGCGCGCTCTCGG----------------------     32.978      6.933      3.652
---------AGTGCCAGCGCGCTCTCGGC---------------------      5.537     21.633     27.485
---------AGTGCCAGCGCGCTCTCGGCC--------------------      0.049      0.038      0.038
---------AGTGCCAGCGCGCTCTCGG----------------------      0.194      0.000      0.000
----------GTGCCAGCGCGCTCTCGGCC--------------------      0.049      0.038      0.038
----------GTGCCAGCGCGCTCTCGGC---------------------      0.583      0.833      1.393
----------GTGCCAGCGCGCTCTCGG----------------------      0.049      0.000      0.000
-----------TGCCAGCGCGCTCTCGGCC--------------------      1.700      1.137      0.602
-----------TGCCAGCGCGCTCTCGGCCA-------------------      0.291      0.038      0.038
-----------TGCCAGCGCGCTCTCGGCCAAG-----------------      3.060      3.599      3.313
------------GCCAGCGCGCTCTCGGCCAAG-----------------      0.049      0.227      0.038
------------GCCAGCGCGCTCTCGGCC--------------------      0.049      0.038      0.038
---------------------CTCTCGGCCAAGTCTGGCAGA--------      0.194      0.000      0.000
----------------------TCTCGGCCAAGTCTGGCAGAAGC-----      0.097      0.265      0.038
----------------------TCTCGGCCAAGTCTGGCAGAAGCGGA--      0.146      0.455      1.468
----------------------TCTCGGCCAAGTCTGGCAGA--------      0.194      0.038      0.038
----------------------TCTCGGCCAAGTCTGGCAGAAGCGGAG-      0.923      2.463      0.038
----------------------TCTCGGCCAAGTCTGGCAGAAGCGG---      0.097      0.341      0.341
-----------------------CTCGGCCAAGTCTGGCAG---------      0.049      0.000      0.000
------------------------TCGGCCAAGTCTGGCAGAAGCGGAG-      1.603      2.841      0.113
------------------------TCGGCCAAGTCTGGCAGAAG------      2.088      2.311      1.092
------------------------TCGGCCAAGTCTGGCAGAAGCG----      0.534      1.099      0.075
------------------------TCGGCCAAGTCTGGCAGAAGCGGA--      0.389      0.682      2.824
------------------------TCGGCCAAGTCTGGCAGAAGC-----      4.663      5.759      1.581
------------------------TCGGCCAAGTCTGGCAGAAGCGG---      0.243      1.174      0.075

---A                 C                   AA    AG
    TAATTAGTGCCAGCGCG TCTCGGCCAAGTCTGGCAG  GCGG  A
    GTTAATCACGGTCGCGC AGAGCCGGTTCAGACCGTC  CGCC  /
CGGG                 A                   GC    GG

-------------------GCAAGAGCCGGTTCAGACCGT----------      0.243      0.076      0.076
----------------CGCGCAAGAGCCGGTTCAGAC-------------      0.146      3.410      1.355
---------------TCGCGCAAGAGCCGGTTCAGA--------------      0.049      0.076      0.038
----------------CGCGCAAGAGCCGGTTCAGA--------------      0.583     12.995      9.337
------------CGGTCGCGCAAGAGCCGGTT------------------      0.146      0.152      0.038
---------TCACGGTCGCGCAAGAGCCGGT-------------------      2.040      1.970      0.075
-----------ACGGTCGCGCAAGAGCCGGT-------------------      0.097      0.076      0.038
------TAATCACGGTCGCGCAAGAGCCGGT-------------------      0.049      0.000      0.000
----------CACGGTCGCGCAAGAGCCGGT-------------------     13.648      4.471      3.614
------------CGGTCGCGCAAGAGCCGGT-------------------      0.194      0.947      0.489
-------------GGTCGCGCAAGAGCCGGT-------------------      0.146      0.076      0.076
------------CGGTCGCGCAAGAGCCGG--------------------      0.049      0.038      0.038
----------CACGGTCGCGCAAGAGCCGG--------------------      0.194      0.113      0.113
---------TCACGGTCGCGCAAGAGCC----------------------      0.049      0.038      0.038
------TAATCACGGTCGCGCAAGAGC-----------------------      0.437      0.076      0.038 miR_t29*
---------TCACGGTCGCGCAAGAG------------------------      0.049      0.000      0.000
----GTTAATCACGGTCGCGCAAGA-------------------------      0.049      0.113      0.113

miRNA: miR_t37,miR_t39 chromosome_10 Start: 6282475 End: 6282576 Condition: HS+N
-------CGGTCATCAATTGCTCTTTCTG-----------------------      0.291      0.189      0.038
----------TCATCAATTGCTCTTTCTG-----------------------      0.000      0.075      0.075
----------TCATCAATTGCTCTTTCTGATG--------------------      0.826      0.113      0.113
-------------TCAATTGCTCTTTCTGATGA-------------------     15.785      3.675      1.054
-------------TCAATTGCTCTTTCTGAT---------------------      0.146      0.417      0.038
-------------TCAATTGCTCTTTCTGATGAG------------------      1.408      0.076      0.075
-------------TCAATTGCTCTTTCTGATG--------------------    153.718    272.136     90.324 miR_t37
-------------TCAATTGCTCTTTCTGA----------------------      0.146      0.189      0.075
--------------CAATTGCTCTTTCTGATG--------------------      3.497      2.766      1.205
---------------AATTGCTCTTTCTGATG--------------------      1.166      0.909      0.565
------------------------TTCTGATGAGCGATGCCGTGG-------      0.000      0.227      0.038

--G   -                 T                       CCG
   CTC CGGTCATCAATTGCTCT TCTGATGAGCGATGCCGTGGCCT   C
   GAG GCCAGTAGTTAACGAGA AGACTACTCGCTACGGCACCGGA   C
GCG   T                 T                       CCC

-------------AGTTAACGAGATAGACTACT-------------------      0.146      0.152      0.038
-----------GTAGTTAACGAGATAGACT----------------------     14.570    100.663     57.756 miR_t39
--------CCAGTAGTTAACGAGATAGACT----------------------      0.826      3.031      0.301
-------------AGTTAACGAGATAGACT----------------------      0.000      0.265      0.075
----AGTGCCAGTAGTTAACGAGATAGACT----------------------      0.000      0.038      0.038
----------AGTAGTTAACGAGATAGACT----------------------      4.128      2.993      2.824
---------CAGTAGTTAACGAGATAGACT----------------------     10.394      2.955      2.033
-----------GTAGTTAACGAGATAGAC-----------------------      0.049      0.493      0.339
---------CAGTAGTTAACGAGATAGA------------------------      0.049      0.114      0.151
-----------GTAGTTAACGAGATAGA------------------------      0.097      7.805      2.749
----------AGTAGTTAACGAGATAGA------------------------      0.000      0.189      0.113
-----GTGCCAGTAGTTAACGAGATAG-------------------------      0.243      0.076      0.075
-----GTGCCAGTAGTTAACGAGAT---------------------------      0.340      0.038      0.038
----AGTGCCAGTAGTTAACGAGAT---------------------------      0.049      0.114      0.075

miRNA: miR_t41 chromosome_7 Start: 2713436 End: 2713688 Condition: TAP
--------------------------------------------------------------------------------------------GACGCGGTGATGGGCCTGG------------------------      0.486      0.682      0.489
-------------------------------------------------------------------------------------------CGACGCGGTGATGGGCCTGG------------------------      0.437      0.189      0.189
------------------------------------------------------------------------------------------TCGACGCGGTGATGGGCCTGGT-----------------------      2.380      2.349      1.468
------------------------------------------------------------------------------------------TCGACGCGGTGATGGGCCTGG------------------------    131.231    310.325    170.106 miR_t41
-----------------------------------------------------------------------------------------TTCGACGCGGTGATGGGCCTGG------------------------      3.254      3.675      2.786
-------------------------------TGCTGCGAGATGCAGCAGGCCAGG--------------------------------------------------------------------------------      0.097      0.000      0.000
----------------------ACTCCCATGTGCTGCGAGATG--------------------------------------------------------------------------------------------      0.097      0.000      0.000
------------------TGGAACTCCCATGTGCTGCGAG-----------------------------------------------------------------------------------------------      0.049      0.000      0.000
----------------TATGGAACTCCCATGTGCTGC--------------------------------------------------------------------------------------------------      1.360      3.599      1.393
----TCGACGATGCGGTATGGAACT--------------------------------------------------------------------------------------------------------------      0.923      9.623      3.614
----TCGACGATGCGGTATGGAACTC-------------------------------------------------------------------------------------------------------------      1.214     16.632      2.334

CTTGTCGACG       A                    A      GCA             AT--     A           C                                         A--   CGCA
          ATGCGGT TGGAACTCCCATGTGCTGCG GATGCA   GGCCAGGGCCATT    GACAC AGTAACGATAT CATTACTTCGACGCGGTGATGGGCCTGGTCCGTCACAGGAC   GGG    C
          TACGCTA GTCTTGAGGGTACACGACGC CTACGT   CCGGTCCCGGTAA    TTGTG TCGTTGCTGTA GTAATGAAGCTGCGCCATTATCCGGACCAGGCAGTGTCCTG   CCC    /
----------       A                    G      ---             ATTC     A           C                                         GCA   AAAA

--------------CTAAGTCTTGAGGGTACACGA----------------------------------------------------------------------------------------------------      0.097      0.227      0.038
--------------------CTTGAGGGTACACGACGCGCT----------------------------------------------------------------------------------------------      0.971      0.227      0.151
--------------------------------------------------GGTCCCGGTAAATTCTTGTGAT---------------------------------------------------------------      0.049      0.000      0.000
-------------------------------------------------------------------GTGATCGTTGCTGTACGTAA------------------------------------------------      0.049      0.000      0.000
-------------------------------------------------------------------GTGATCGTTGCTGTACGTAAT-----------------------------------------------      1.748      3.296      0.264
----------------------------------------------------------------------------------------GAAGCTGCGCCATTATCCGGA--------------------------      0.049      0.000      0.000 miR_t41*

miRNA: miR_t40,miR_t42 chromosome_14 Start: 1950047 End: 1950359 Condition: TAP
------------------------------------------------------------------------------------------------------------------------------------------------AGGCGCGGAGCTCATCAAGC----------      0.049      0.076      0.075
```

```
------------------------------------------------------------------------------------------------------------------------------------------------TAGGCGCGGAGCTCATCAAGC----------      1.554      7.426      7.530
------------------------------------------------------------------------------------------------------------------------------------------------TAGGCGCGGAGCTCATCAAG-----------      0.146      0.188      0.188
------------------------------------------------------------------------------------------------------------------------------------------------TAGGCGCGGAGCTCATCAAGCG---------      0.049      0.000      0.000
------------------------------------------------------------------------------------------------------------------TGCAGGGGCATCTCGATGTCC----------------------------------------      0.049      0.000      0.000
---------------------------------------------------------------------------------------------------------------TACTGCAGGGGCATCTC-----------------------------------------------      0.146      0.152      0.414
---------------------------------------------------------------------------------------------------------------TACTGCAGGGGCATCT------------------------------------------------      0.049      0.000      0.000
---------------------------------------------------------------------------------------------------TCGCCGCGCAGGTACTGCAGG-------------------------------------------------------      0.874      0.114      0.114
------------------------------------------------------------------------------------------------TTCTCGCCGCGCAGGTACTGC----------------------------------------------------------      0.534      0.227      0.339
---------------------------------------------------------------------------------------------ACGTTCTCGCCGCGCAGG----------------------------------------------------------------      0.049      0.000      0.000
--------------------------------------------------------------------------------------------CACGTTCTCGCCGCGCAGG----------------------------------------------------------------      9.811      1.364      1.920
------------------------------------------------------------------------------------------TCCACGTTCTCGCCGCGCAGG----------------------------------------------------------------    339.443     84.221     61.672 miR_t40
------------------------------------------------------------------------------------------TCCACGTTCTCGCCGCGCAGGT---------------------------------------------------------------      0.729      0.076      0.113
------------------------------------------------------------------------------------------TCCACGTTCTCGCCGCGCAGGTACT------------------------------------------------------------      0.049      0.000      0.000
------------------------------------------------------------------------------------------TCCACGTTCTCGCCGC---------------------------------------------------------------------      0.097      0.000      0.000
------------------------------------------------------------------------------------------TCCACGTTCTCGCCGCG--------------------------------------------------------------------      0.049      0.000      0.000
------------------------------------------------------------------------------------------TCCACGTTCTCGCCGCGCAG-----------------------------------------------------------------      0.971      0.720      0.941
-----------------------------------------------------------------------------------------CTCCACGTTCTCGCCGCGCAGG----------------------------------------------------------------      0.243      0.152      0.152
---------------------------------------------------------------------------------------TTCTCCACGTTCTCGCCGCGC-------------------------------------------------------------------      0.243      0.038      0.038
---------------------------------------------------------------------------------------TTCTCCACGTTCTCGCCGCGCA------------------------------------------------------------------      0.146      0.038      0.038
---------------------------------------------------------------------------TTCTCCGTCACCTTCTCCACG-------------------------------------------------------------------------------      0.340      0.227      0.452
-------------------------------------------------------------------------CATTCTCCGTCACCTTCTCC----------------------------------------------------------------------------------      0.049      0.000      0.000
-------------------------------------------------------------------------CATTCTCCGTCACCTTCT------------------------------------------------------------------------------------      0.049      0.000      0.000
------------------------------------------------------------------------ACATTCTCCGTCACCTTCTCC----------------------------------------------------------------------------------      0.146      0.038      0.226
-----------------------------------------------------------------------TACATTCTCCGTCACCTTCT------------------------------------------------------------------------------------      2.477      1.364      0.904
-----------------------------------------------------------------------TACATTCTCCGTCACCTTC-------------------------------------------------------------------------------------      2.574      5.380      3.238
-----------------------------------------------------------------------TACATTCTCCGTCACCTTCTCC----------------------------------------------------------------------------------      4.808      2.538      3.276
-----------------------------------------------------------------------TACATTCTCCGTCACCTTCTC-----------------------------------------------------------------------------------      0.291      0.189      0.075
-----------------------------------------------------------------------TACATTCTCCGTCACCTTCTCCA---------------------------------------------------------------------------------      0.437      0.114      0.114
-----------------------------------------------------------------------TACATTCTCCGTCACC----------------------------------------------------------------------------------------      0.049      0.000      0.000
----------------------------------------------------------------------TTACATTCTCCGTCACCTTC-------------------------------------------------------------------------------------      0.874      0.265      1.017
----------------------------------------------------------------------TTACATTCTCCGTCACCTTCTCC----------------------------------------------------------------------------------      0.049      0.038      0.038
----------------------------------------------------------------------TTACATTCTCCGTCACC----------------------------------------------------------------------------------------      0.049      0.038      0.038
----------------------------------------------------------------------TTACATTCTCCGTCACCTTCTC-----------------------------------------------------------------------------------      0.146      0.113      0.113
----------------------------------------------------------------------TTACATTCTCCGTCACCTTCT------------------------------------------------------------------------------------      0.146      0.301      0.301
-----------------------------------------------------------------TTACCTTACATTCTCCGTCACCTTCT------------------------------------------------------------------------------------      0.049      0.000      0.000
-----------------------------------------------------------------TTACCTTACATTCTCCGTCACCTTC-------------------------------------------------------------------------------------      0.049      0.152      0.152
----------------------------------------------------------TACCCACTTACCTTACATTCTCC----------------------------------------------------------------------------------------------      0.971      1.174      0.113
----------------------------------------------------------TACCCACTTACCTTACATTCTC-----------------------------------------------------------------------------------------------      0.243      0.113      0.113
------------------------------------CGAAGTTATCAGGAACGGGAAG---------------------------------------------------------------------------------------------------------------------      0.049      0.000      0.000
----TTTTTGTAACGACGGGAAAAG------------------------------------------------------------------------------------------------------------------------------------------------------      0.049      0.038      0.038
----TTTTTGTAACGACGGGAAAAGG-----------------------------------------------------------------------------------------------------------------------------------------------------      0.049      0.000      0.000
----TTTTTGTAACGACGGGAAAA-------------------------------------------------------------------------------------------------------------------------------------------------------      0.049      0.000      0.000

GGGGTTTTTGTAACGACGGGAAAAGGCCCGCCCCTTC      AT                  A  TACC                       A                                                                            C TC
                                     GAAGTT  CAGGAACGGGAAGTACCC CT    TTACATTCTCCGTCACCTTCTCC CGTTCTCGCCGCGCAGGTACTGCAGGGGCATCTCGATGTCCATCGCCTCGTAGGCGCGGAGCTCATCAAGCGCCTG G  T
                                     CTTCAA  GTCCTTGCCCTTCATGGG GG    AATGTAAGAGGCGGTGGAAGAGG GCAAGAGCGGCGCGTCCATGACGTCCCCGTAGAGCTACAGGTAGCGGAGCATCCGCGCCTCGAGTAGTTCGCGGAC C  T
-----------------------------------AT      CG                  C  CGC-                       C                                                                            - TT

---------------------------------------TCAACGGTCCTTGCCCTTCAT-------------------------------------------------------------------------------------------------------------------      0.049      0.000      0.000
------------------------------------------------------------------CGC-AATGTAAGAGGCGGTGGA---------------------------------------------------------------------------------------      0.049      0.682      0.866
----------------------------------------------------------------------AATGTAAGAGGCGGTGGAAGA------------------------------------------------------------------------------------      0.049      1.894      1.995
-------------------------------------------------------------------------------------GGAAGAGGCGCAAGAGCGGCGC--------------------------------------------------------------------      0.049      0.265      0.113
-------------------------------------------------------------------------------------GGAAGAGGCGCAAGAGCGGCGCGT------------------------------------------------------------------      0.097      0.265      0.188
------------------------------------------------------------------------------------TGGAAGAGGCGCAAGAGCGGCGCGT------------------------------------------------------------------      0.097      0.038      0.038
-------------------------------------------------------------------------------------------------AGAGCGGCGCGTCCATGACGT---------------------------------------------------------      0.146      0.076      0.038
--------------------------------------------------------------------------------------------------GAGCGGCGCGTCCATGACGT---------------------------------------------------------      0.049      0.076      0.038
------------------------------------------------------------------------------------------------------GGCGCGTCCATGACGTCCCCGT---------------------------------------------------      0.146      0.000      0.000
-------------------------------------------------------------------------------------------------------GCGCGTCCATGACGTCCCCGT---------------------------------------------------      0.049      0.038      0.113
-------------------------------------------------------------------------------------------------------------CCATGACGTCCCCGTAGA------------------------------------------------      0.049      0.075      0.075
--------------------------------------------------------------------------------------------------------------CATGACGTCCCCGTAGAG-----------------------------------------------      0.049      0.114      0.075
-----------------------------------------------------------------------------------------------------------GTCCATGACGTCCCCGTAGAG-----------------------------------------------      0.097      0.265      0.188
-------------------------------------------------------------------------------------------------------------CCATGACGTCCCCGTAGAG-----------------------------------------------      0.097      1.326      1.468
-------------------------------------------------------------------------------------------------------------CCATGACGTCCCCGTAGAGCT---------------------------------------------     38.175     30.385     33.133 miR_t42
-----------------------------------------------------------------------------------------------------------GTCCATGACGTCCCCGTAGAGCT---------------------------------------------      0.291      0.379      0.226
------------------------------------------------------------------------------------------------------------TCCATGACGTCCCCGTAGAGCT---------------------------------------------      0.729      0.076      0.151
---------------------------------------------------------------------------------------------------------------ATGACGTCCCCGTAGAGCTAC-------------------------------------------      0.049      0.339      0.339
--------------------------------------------------------------------------------------------------------------------GTCCCCGTAGAGCTACAGGT---------------------------------------      0.049      0.038      0.038
-----------------------------------------------------------------------------------------------------------------GACGTCCCCGTAGAGCTACAGGT---------------------------------------      0.049      0.000      0.000
------------------------------------------------------------------------------------------------------------------ACGTCCCCGTAGAGCTACAGGT---------------------------------------      0.049      0.076      0.151
-------------------------------------------------------------------------------------------------------------------CGTCCCCGTAGAGCTACAGGT---------------------------------------     10.296      2.046      2.297
----------------------------------------------------------------------------------------------------------------------------------------------GCATCCGCGCCTCGAGTAGTT------------      0.243      0.000      0.000

miRNA: miR_t44 chromosome_17 Start: 6144113 End: 6144214 Condition: TAP
-----------------------------TGGAGCAGGGCAGGCCGCAA---      0.049      0.075      0.075
-----------------------------TGGAGCAGGGCAGGCCGCAAA--      0.049      0.000      0.000
-----------------------------TGGAGCAGGGCAGGCCGCA----      0.049      0.000      0.000
-----------------------------TGGAGCAGGGCAGGCCGCAAAT-      0.146      0.000      0.000
-----------------------------TGGAGCAGGGCAGGCCGC-----      2.137      0.076      0.038
-----------------------TTTATGTGGAGCAGGGCAGG---------      0.049      0.038      0.075
-------------------TCTGTTTATGTGGAGCAGGGC------------      0.194      0.000      0.000
----------------TTGTCTGTTTATGTGGAGC-----------------      0.680      0.379      0.075
----------------TTGTCTGTTTATGTGG--------------------      0.097      0.000      0.000
---------------CTTGTCTGTTTATGTGGAGC-----------------      0.049      0.000      0.000
-------------GCCTTGTCTGTTTATG-----------------------      0.049      0.000      0.000
-------------GCCTTGTCTGTTTATGTGG--------------------      1.700      0.114      0.226
-------------GCCTTGTCTGTTTATGTG---------------------      0.243      0.038      0.038
------------CGCCTTGTCTGTTTATG-----------------------      0.049      0.000      0.000
------------CGCCTTGTCTGTTTATGTGG--------------------      7.479      1.061      0.188
------------CGCCTTGTCTGTTTATGTG---------------------      0.680      0.189      0.226
------------CGCCTTGTCTGTTTATGTGGA-------------------      0.291      0.000      0.000
-----------TCGCCTTGTCTGTTTATGTGG--------------------    483.205    100.929     35.881 miR_t44
-----------TCGCCTTGTCTGTTTATG-----------------------      0.437      0.076      0.038
-----------TCGCCTTGTCTGTTTATGTGGA-------------------      7.577      1.515      1.515
-----------TCGCCTTGTCTGTTTATGTGGAG------------------      0.097      0.000      0.000
-----------TCGCCTTGTCTGTTTATGTG---------------------     42.206     35.802     19.654
-----------TCGCCTTGTCTGTTTATGTGGAGC-----------------      0.049      0.303      0.303
----------GTCGCCTTGTCTGTTTATGTG---------------------      0.049      0.000      0.000
----------GTCGCCTTGTCTGTTTATG-----------------------      0.049      0.000      0.000
---------GGTCGCCTTGTCTGTTTATG-----------------------     11.462      6.441      4.179
--------CGGTCGCCTTGTCTGTTTATG-----------------------     16.756      5.645      4.179
--------CGGTCGCCTTGTCTGTTTATGTG---------------------      0.049      0.000      0.000
-------TCGGTCGCCTTGTCTGTTTAT------------------------      0.049      0.000      0.000
-------TCGGTCGCCTTGTCTGTTTATG-----------------------     21.419     16.329      8.208

---CA G                                        AAAT
     T TCGGTCGCCTTGTCTGTTTATGTGGAGCAGGGCAGGCCGC    G
     A AGCCAGCGGAACAGACAAATACACCTCGTCCCGTCCGGCG    G
GCAGC G                                        GGGG

-------AGCCAGCGGAACAGACAA---------------------------      0.291      0.038      0.075
-------AGCCAGCGGAACAGACAAA--------------------------      0.826      0.493      1.807
--------GCCAGCGGAACAGACAAA--------------------------      0.049      0.075      0.075
-----AGAGCCAGCGGAACAGACAAA--------------------------      0.194      0.000      0.000
-------AGCCAGCGGAACAGACAAAT-------------------------     76.301     51.070     61.860
--------GCCAGCGGAACAGACAAAT-------------------------      0.729      0.947      1.506
-----AGAGCCAGCGGAACAGACAAAT-------------------------      5.537      0.866      0.866
------GAGCCAGCGGAACAGACAAAT-------------------------      0.097      0.075      0.075
----CAGAGCCAGCGGAACAGACAAAT-------------------------      0.049      0.000      0.000
---------CCAGCGGAACAGACAAATACA----------------------      0.049      0.076      0.076 miR_t44*
-----------AGCGGAACAGACAAATACACCT-------------------      0.049      0.000      0.000
------------GCGGAACAGACAAATACACCT-------------------      1.457      0.114      0.151

miRNA: miR_t46 chromosome_2 Start: 9101099 End: 9101289 Condition: TAP
------------------------------------------------------------------------TCTCAGTACGAGCGACATAGG-----      0.049      0.152      0.152
-----------------------------------------------------TTCCGACGCAGCGGCAGTGTC------------------------      1.117      0.152      0.151
----------------------------------------------------TTTCCGACGCAGCGGCAGTGTC------------------------      1.457      0.947      0.226
----------------------------------------------------TTTCCGACGCAGCGGCAGTG--------------------------      0.049      0.113      0.113
-------------------------------------------------TCCTTTCCGACGCAGCGGCAGT---------------------------      0.097      0.076      0.076
-------------------------------------------------TCCTTTCCGACGCAGCGGCAG----------------------------      6.265      0.947      1.242
------------------------------------------------TTCCTTTCCGACGCAGCGGCAG----------------------------      0.631      0.227      0.113
------------------------------------------------TTCCTTTCCGACGCAGCGGC------------------------------      0.049      0.152      0.152
--------------------------------------------TGGATTCCTTTCCGACGCAGC---------------------------------      0.243      0.455      0.678
-------------------------------------------TTGGATTCCTTTCCGACGC------------------------------------      0.097      0.038      0.113
-------------------------------------AGGCCGTTGGATTCCTTTCCG----------------------------------------      1.263      2.349      4.104
-------------------------------------AGGCCGTTGGATTCCTTTCCGA---------------------------------------      0.049      1.402      2.523
------------------------------------GAGGCCGTTGGATTCCTTTCCG----------------------------------------      0.049      0.075      0.075
----------------------------------TGGAGGCCGTTGGATTCCTTTCC-----------------------------------------      0.049      0.114      0.114
----------------------------------TGGAGGCCGTTGGATTCCTTT-------------------------------------------      0.243      0.303      0.188
--------------------------------CATGGAGGCCGTTGGATTCC----------------------------------------------      0.097      0.227      0.227
-------------------------------TCATGGAGGCCGTTGGATTCCT---------------------------------------------      0.049      0.000      0.000
-------------------------------TCATGGAGGCCGTTGGATTCC----------------------------------------------     15.105     14.548      5.045
----------TGGACGCAAACCACACCAG---------------------------------------------------------------------      0.097      0.000      0.000
----------TGGACGCAAACCACACCAGCG-------------------------------------------------------------------      0.437      0.303      0.602
----------TGGACGCAAACCACACCAGCGTC-----------------------------------------------------------------      0.049      0.000      0.000
----------TGGACGCAAACCACACCAGC--------------------------------------------------------------------      1.360      0.379      0.151

------                                                     C                            TA   C A
      AACATGGACGCAAACCACACCAGCGTCATGGAGGCCGTTGGATTCCTTTCCGA GCAGCGGCAGTGTCTCAGTACGAGCGACA  GGC C T
      TTGTACCTGCGTTTGGTGTGGTCGCAGTACCTCCGGCAACCTAAGGAAAGGCT CGTCGCCGTCACAGAGTCATGCTCGCTGT  CCG G A
CTCGCC                                                     C                            TC   A A

----CCTTGTACCTGCGTTTGGTGT-------------------------------------------------------------------------      0.049      0.000      0.000
-----------CCTGCGTTTGGTGTGGTCGCAGT----------------------------------------------------------------      0.049      0.000      0.000
-------------------------------------TCCGGCAACCTAAGGAAAGG-----------------------------------------      0.049      0.000      0.000
--------------------------------------CCGGCAACCTAAGGAAAGG-----------------------------------------      1.360      0.720      0.791
--------------------------------------CCGGCAACCTAAGGAAAGGC----------------------------------------      5.828      3.258      4.593
-------------------------------------TCCGGCAACCTAAGGAAAGGC----------------------------------------      0.146      0.076      0.113
-----------------------------------CCTCCGGCAACCTAAGGAAAGGCT---------------------------------------      0.049      0.000      0.000
------------------------------------CTCCGGCAACCTAAGGAAAGGCT---------------------------------------      0.146      0.076      0.075
--------------------------------------CCGGCAACCTAAGGAAAGGCT---------------------------------------    718.080    976.211    865.630 miR_t46
----------------------------------------GGCAACCTAAGGAAAGGCT---------------------------------------      0.194      3.864      1.242
-----------------------------------------GCAACCTAAGGAAAGGCT---------------------------------------      0.049      0.076      0.076
---------------------------------------CGGCAACCTAAGGAAAGGCT---------------------------------------      3.545     37.280     25.339
-------------------------------------TCCGGCAACCTAAGGAAAGGCT---------------------------------------     21.856      9.244      5.497
--------------------------------------CCGGCAACCTAAGGAAAGGCTC--------------------------------------      0.146      0.871      0.941
-------------------------------------TCCGGCAACCTAAGGAAAGGCTC--------------------------------------      1.894      0.909      0.602
```

```
---------------------------------------CGGCAACCTAAGGAAAGGCTC--------------------------------------      0.097      0.303      0.527
----------------------------------------GGCAACCTAAGGAAAGGCTCCGT-----------------------------------      0.049      0.000      0.000
-----------------------------------------GCAACCTAAGGAAAGGCTCCGT-----------------------------------      0.097      0.038      0.038
------------------------------------------CAACCTAAGGAAAGGCTCCGT-----------------------------------      0.437      0.076      0.076

miRNA: miR_t49 chromosome_17 Start: 5152778 End: 5152924 Condition: TAP
-------------------------------------------TCTCCGAGCTAGCTTGACGC-----------      0.049      0.000      0.000
-------------------------------------CCCGCTTCTCCGAGCTAGC------------------      0.097      0.000      0.000
------------------------------------ACCCGCTTCTCCGAGCTAG-------------------      0.049      0.000      0.000
-----------------------------------TACCCGCTTCTCCGAGCT---------------------      0.049      0.000      0.000
-----------------------------------TACCCGCTTCTCCGAGCTAGC------------------      2.477      0.189      0.264
-----------------------------------TACCCGCTTCTCCGAGCTAG-------------------     12.385      1.553      1.242
-----------------------------------TACCCGCTTCTCCGAGCTA--------------------      0.146      0.000      0.000
----------------------------------CTACCCGCTTCTCCGAGCTAG-------------------      0.049      0.000      0.000
--------------------------------AGCTACCCGCTTCTCCGAGCT---------------------      0.049      0.038      0.038
--------------------------------AGCTACCCGCTTCTCCGAG-----------------------      0.049      0.000      0.000
--------------------------------AGCTACCCGCTTCTCCGAGC----------------------      0.389      0.075      0.075 miR_t49*
-------------------------------CAGCTACCCGCTTCTCCGAG-----------------------      0.049      0.038      0.038
-------------------------------CAGCTACCCGCTTCTCCGAGC----------------------      0.486      0.038      0.038
------------------------------TCAGCTACCCGCTTCTCCG-------------------------      0.291      0.076      0.076
------------------------------TCAGCTACCCGCTTCTCC--------------------------      0.097      0.075      0.075
------------------------------TCAGCTACCCGCTTCTCCGAGC----------------------     98.593     12.427     16.115
------------------------------TCAGCTACCCGCTTCTCCGAG-----------------------      9.374      1.705      1.355
------------------------------TCAGCTACCCGCTTCTCCGAGCT---------------------      2.137      0.417      0.151
------------------------------TCAGCTACCCGCTTCTCCGA------------------------      0.097      0.000      0.000
-----------------------------CTCAGCTACCCGCTTCTCCGAGCT---------------------      0.049      0.000      0.000
--------------------------TACCTCAGCTACCCGCTTCTCCG-------------------------      0.291      0.114      0.114
------------------------TCTACCTCAGCTACCCGCTTC-----------------------------      0.049      0.226      0.226
---------------------TCCTCTACCTCAGCTACCCGC--------------------------------      0.049      0.000      0.000
-------------------TCTCCTCTACCTCAGC---------------------------------------      0.049      0.000      0.000
-----------------CTTCTCCTCTACCTCAGC---------------------------------------      0.049      0.000      0.000
----------------TCTTCTCCTCTACCTCAGC---------------------------------------      0.097      0.152      0.152
--------------AATCTTCTCCTCTACCTCAG----------------------------------------      0.146      0.113      0.113
-------------TAATCTTCTCCTCTACCTCA-----------------------------------------      0.680      0.038      0.038
-------------TAATCTTCTCCTCTACCTCAGC---------------------------------------      2.671      0.682      0.791
-------------TAATCTTCTCCTCTACCTCAG----------------------------------------      3.497      2.160      1.845
-------------TAATCTTCTCCTCTACCTC------------------------------------------      0.291      0.000      0.000
-------------TAATCTTCTCCTCTACC--------------------------------------------      0.049      0.000      0.000
------------TTAATCTTCTCCTCTACC--------------------------------------------      0.049      0.038      0.038
------------TTAATCTTCTCCTCTACCTC------------------------------------------      2.526      0.758      0.414
------------TTAATCTTCTCCTCTACCTCA-----------------------------------------     17.630      5.228      2.297
------------TTAATCTTCTCCTCTACCTCAGC---------------------------------------      4.905      4.698      5.083
------------TTAATCTTCTCCTCTACCTCAG----------------------------------------      0.194      0.455      0.791
------------TTAATCTTCTCCTCTACCT-------------------------------------------      0.097      0.076      0.076
-----------TTTAATCTTCTCCTCTACCTC------------------------------------------    112.484     19.170     14.270
-----------TTTAATCTTCTCCTCTACC--------------------------------------------      3.934      0.833      1.657
-----------TTTAATCTTCTCCTCTAC---------------------------------------------      0.146      0.075      0.075
-----------TTTAATCTTCTCCTCTACCT-------------------------------------------      6.217      1.591      1.167
-----------TTTAATCTTCTCCTCTACCTCA-----------------------------------------      4.031      0.833      0.527
-----------TTTAATCTTCTCCTCTACCTCAGC---------------------------------------      0.194      0.833      0.377
----------GTTTAATCTTCTCCTCTACCTC------------------------------------------      0.049      0.000      0.000
----------GTTTAATCTTCTCCTCTACC--------------------------------------------      0.146      0.000      0.000
----------GTTTAATCTTCTCCTCTACCT-------------------------------------------      0.097      0.000      0.000
---------TGTTTAATCTTCTCCTCTACC--------------------------------------------     24.090      6.819     10.090
---------TGTTTAATCTTCTCCTC------------------------------------------------      0.049      0.000      0.000
---------TGTTTAATCTTCTCCTCTA----------------------------------------------      0.049      0.000      0.000
---------TGTTTAATCTTCTCCTCTACCTC------------------------------------------      4.711      0.947      0.715
---------TGTTTAATCTTCTCCTCTAC---------------------------------------------      0.049      0.076      0.038
---------TGTTTAATCTTCTCCTCTACCT-------------------------------------------      4.711      1.553      1.280
-------TGTGTTTAATCTTCTCCTCTA----------------------------------------------      0.243      0.076      0.076
-------TGTGTTTAATCTTCTCCTCT-----------------------------------------------      0.097      0.000      0.000
------TTGTGTTTAATCTTCTCCTCT-----------------------------------------------      6.800      0.758      0.301
------TTGTGTTTAATCTTCTCCTC------------------------------------------------      0.291      0.000      0.000
------TTGTGTTTAATCTTCTCCTCTA----------------------------------------------      0.680      0.152      0.151

--                                                                  G  AT
  GACATTGTGTTTAATCTTCTCCTCTACCTCAGCTACCCGCTTCTCCGAGCTAGCTTGACGCTCTCT AG  G
  CTGTAACACAAATTAGAAGAGGAGATGGAGTCGATGGGCGAAGAGGCTCGATCGAACTGCGAGAGA TC  G
CG                                                                  G  GG

-------ACACAAATTAGAAGAGGAG------------------------------------------------      0.049      0.151      0.151
------AACACAAATTAGAAGAGGAGA-----------------------------------------------      0.194      0.038      0.113
-------ACACAAATTAGAAGAGGAGA-----------------------------------------------      0.631      0.530      0.602
----GTAACACAAATTAGAAGAGGAGAT----------------------------------------------      0.049      0.227      0.113
-------ACACAAATTAGAAGAGGAGAT----------------------------------------------     37.932     72.590     66.040
------AACACAAATTAGAAGAGGAGAT----------------------------------------------      6.265     10.343     11.483
-----TAACACAAATTAGAAGAGGAGAT----------------------------------------------      0.971      1.288      0.339
---------ACAAATTAGAAGAGGAGAT----------------------------------------------      0.826      0.417      1.431
--------CACAAATTAGAAGAGGAGAT----------------------------------------------      0.243      1.250      1.355
----------CAAATTAGAAGAGGAGAT----------------------------------------------      0.049      0.417      0.417
---------ACAAATTAGAAGAGGAGATGG--------------------------------------------      0.049      0.000      0.000
------------AATTAGAAGAGGAGATGGA-------------------------------------------      0.340      0.000      0.000
-----------AAATTAGAAGAGGAGATGGA-------------------------------------------      0.097      0.038      0.038
------------AATTAGAAGAGGAGATGGAG------------------------------------------      3.108      0.644      2.410
-------------ATTAGAAGAGGAGATGGAG------------------------------------------      0.389      0.114      0.151
-----------AAATTAGAAGAGGAGATGGAG------------------------------------------      0.243      0.000      0.000
----------CAAATTAGAAGAGGAGATGGAGT-----------------------------------------      0.243      0.341      0.341
-------------ATTAGAAGAGGAGATGGAGT-----------------------------------------     97.331      7.047     18.110
----------------AGAAGAGGAGATGGAGT-----------------------------------------      0.194      0.606      1.468
-----------------GAAGAGGAGATGGAGT-----------------------------------------      0.049      0.000      0.000
---------------TAGAAGAGGAGATGGAGT-----------------------------------------      0.097      0.303      1.205
-------------------AGAGGAGATGGAGT-----------------------------------------      0.049      0.000      0.000
-----------AAATTAGAAGAGGAGATGGAGT-----------------------------------------     12.191      7.388      4.556
---------ACAAATTAGAAGAGGAGATGGAGT-----------------------------------------      0.097      0.000      0.000
--------------TTAGAAGAGGAGATGGAGT-----------------------------------------      1.943      1.629      1.995
------------AATTAGAAGAGGAGATGGAGT-----------------------------------------    484.807     90.737    238.179
------------AATTAGAAGAGGAGATGGAGTC----------------------------------------      0.097      0.188      0.188
----------------AGAAGAGGAGATGGAGTCGAT-------------------------------------      0.146      0.227      0.527
-----------------GAAGAGGAGATGGAGTCGAT-------------------------------------      0.534      0.189      0.414
-------------------------GATGGAGTCGATGGGCGAAGA----------------------------      0.049      0.114      0.038
-------------------------GATGGAGTCGATGGGCGAAGAG---------------------------      0.097      0.188      0.188
----------------------------GGAGTCGATGGGCGAAGAGG--------------------------      0.680      1.099      0.377
------------------------------AGTCGATGGGCGAAGAGG--------------------------     17.970      8.221     14.571
-----------------------------GAGTCGATGGGCGAAGAGG--------------------------      0.291      0.796      1.205
----------------------------GGAGTCGATGGGCGAAGAGGC-------------------------      0.631      0.265      0.188
-----------------------------GAGTCGATGGGCGAAGAGGC-------------------------      0.777      0.189      0.489
------------------------------AGTCGATGGGCGAAGAGGC-------------------------     14.279      4.849     14.081
----------------------------GGAGTCGATGGGCGAAGAGGCT------------------------    265.571    196.326     85.204
-----------------------------------ATGGGCGAAGAGGCT------------------------      0.146      0.000      0.000
----------------------------------GATGGGCGAAGAGGCT------------------------      0.049      0.000      0.000
---------------------------TGGAGTCGATGGGCGAAGAGGCT------------------------      0.534      0.417      0.417
--------------------------------TCGATGGGCGAAGAGGCT------------------------      0.097      0.076      0.414
-------------------------------GTCGATGGGCGAAGAGGCT------------------------      5.440      2.652      4.857
---------------------------------CGATGGGCGAAGAGGCT------------------------      0.049      0.417      0.753
------------------------------AGTCGATGGGCGAAGAGGCT------------------------   5973.399   3599.216   5830.945 miR_t49
-----------------------------GAGTCGATGGGCGAAGAGGCT------------------------    315.693    159.690    317.735
------------------------------AGTCGATGGGCGAAGAGGCTC-----------------------      8.159     14.548     12.425
-------------------------------GTCGATGGGCGAAGAGGCTC-----------------------      0.146      0.227      0.113
-----------------------------GAGTCGATGGGCGAAGAGGCTC-----------------------      0.194      0.113      0.113
----------------------------GGAGTCGATGGGCGAAGAGGCTC-----------------------      0.194      0.189      0.189
---------------------------------CGATGGGCGAAGAGGCTCGA---------------------      0.243      0.455      0.565
---------------------------------CGATGGGCGAAGAGGCTCGAT--------------------     32.638    138.360    122.666
----------------------------------GATGGGCGAAGAGGCTCGAT--------------------      0.340      1.250      0.452

miRNA: miR_t51,miR_t12 chromosome_5 Start: 935250 End: 935514 Condition: TAP
---------------------------------------------------------------------------------------------------------TCCGCTTCAGGTACACGCCGC--------------      0.049      0.000      0.000
--------------------------------------------------------------------------------------------------CAAGATGTCCGCTTCAGG------------------------      0.049      0.493      1.242
--------------------------------------------------------------------------------------------------CAAGATGTCCGCTTCAGGTAC---------------------      0.680      4.584      4.029
-----------------------------------------------------------------------------------------------TGGCAAGATGTCCGCTTCAGGT-----------------------      0.049      0.000      0.000
-----------------------------------------------------------------------------------------------TGGCAAGATGTCCGCTTCAGG------------------------      0.243      1.099      0.979
----------------------------------------------------------------------------------------------TTGGCAAGATGTCCGCTTCAG-------------------------      0.340      0.076      0.075
----------------------------------------------------------------------------------------------TTGGCAAGATGTCCGCTTCAGGT-----------------------      0.049      0.000      0.000
----------------------------------------------------------------------------------------------TTGGCAAGATGTCCGCTTCAGG------------------------      7.625     18.829     29.744
---------------------------------------------------------------------------------------------TTTGGCAAGATGTCCGCTTCAGG------------------------      1.943      1.781      2.485
---------------------------------------------------------------------------------------------TTTGGCAAGATGTCCGCTTCAG-------------------------      0.194      0.075      0.075
------------------------------------------------------------------------------------AGCATTTCGTTTGG------------------------------------------      0.146      0.000      0.000
-----------------------------------------------------------------------------------CAGCATTTCGTTTGG------------------------------------------      0.146      0.000      0.000
-----------------------------------------------------------------------------------CAGCATTTCGTTTGGCAAG--------------------------------------      0.049      0.000      0.000
----------------------------------------------------------------------------------TCAGCATTTCGTTTGGCAAGA-------------------------------------      0.097      0.000      0.000
----------------------------------------------------------------------------------TCAGCATTTCGTTTGG------------------------------------------      0.097      0.000      0.000
----------------------------------------------------------------------------------TCAGCATTTCGTTTGGCAAG--------------------------------------      0.874      0.379      0.264
---------------------------------------------------------------------------------GTCAGCATTTCGTTTGG------------------------------------------      0.146      0.038      0.038
--------------------------------------------------------------------------------GGTCAGCATTTCGTTTGG------------------------------------------      6.411      1.250      1.205
--------------------------------------------------------------------------------GGTCAGCATTTCGTTTG-------------------------------------------      0.097      0.038      0.075
--------------------------------------------------------------------------------GGTCAGCATTTCGTTTGGC-----------------------------------------      0.146      0.000      0.000
-------------------------------------------------------------------------------CGGTCAGCATTTCGTTTGG------------------------------------------     18.650      3.107      3.125
-------------------------------------------------------------------------------CGGTCAGCATTTCGTTTGGC-----------------------------------------      0.437      0.265      0.264
------------------------------------------------------------------------------TCGGTCAGCATTTCGTTTGGCA----------------------------------------      0.146      0.000      0.000
------------------------------------------------------------------------------TCGGTCAGCATTTCGTTTGG------------------------------------------   4444.765    622.165    427.149 miR_t51
------------------------------------------------------------------------------TCGGTCAGCATTTCGTTTG-------------------------------------------     47.548      5.304      7.041
------------------------------------------------------------------------------TCGGTCAGCATTTCGTTT--------------------------------------------      0.194      0.075      0.075
------------------------------------------------------------------------------TCGGTCAGCATTTCGTTTGGC-----------------------------------------    231.719     21.936     24.435
-----------------------------------------------------------------------------CTCGGTCAGCATTTCGTTTG-------------------------------------------      0.194      0.038      0.038
-----------------------------------------------------------------------------CTCGGTCAGCATTTCGTTTGG------------------------------------------      3.060      0.871      0.565
----------------------------------------------------------------------------TCTCGGTCAGCATTTCGTTTG-------------------------------------------    106.704      3.220      4.631
----------------------------------------------------------------------------TCTCGGTCAGCATTTCGTTTGGC-----------------------------------------      0.389      0.455      0.113
----------------------------------------------------------------------------TCTCGGTCAGCATTTCGTT---------------------------------------------      0.243      0.000      0.000
----------------------------------------------------------------------------TCTCGGTCAGCATTTCGT----------------------------------------------      0.097      0.000      0.000
----------------------------------------------------------------------------TCTCGGTCAGCATTTCGTTTGG------------------------------------------    344.445     55.048     30.309
----------------------------------------------------------------------------TCTCGGTCAGCATTTCGTTT--------------------------------------------      0.049      0.038      0.038
--------------------------------------------------------------------------TATCTCGGTCAGCATTTCG-----------------------------------------------      2.477      0.000      0.000
--------------------------------------------------------------------------TATCTCGGTCAGCATTTCGTTTG-------------------------------------------      0.389      0.000      0.000
--------------------------------------------------------------------------TATCTCGGTCAGCATTTCGT----------------------------------------------     11.025      0.000      0.000
--------------------------------------------------------------------------TATCTCGGTCAGCATTTCGTTT--------------------------------------------      0.243      0.000      0.000
--------------------------------------------------------------------------TATCTCGGTCAGCATTTCGTTTGG------------------------------------------      0.291      0.000      0.000
--------------------------------------------------------------------------TATCTCGGTCAGCATTTCGTT---------------------------------------------     14.813      0.000      0.000
```

```
---------------------------------------------------------AGCAAGCTACTTCAGGGTATC--------------------------------------------------------------     0.049     0.000     0.000
--------------------------------------------------------TAGCAAGCTACTTCAGGGTAT---------------------------------------------------------------     4.031     1.137     1.280
--------------------------------------------------------TAGCAAGCTACTTCAGGGTA----------------------------------------------------------------     0.097     0.000     0.000
--------------------------------------------------------TAGCAAGCTACTTCAGGGTATC--------------------------------------------------------------     0.340     0.038     0.075
-------------------------------------------------------TTAGCAAGCTACTTCAGG-------------------------------------------------------------------     0.146     0.038     0.075
-------------------------------------------------------TTAGCAAGCTACTTCAGGGTATC--------------------------------------------------------------     1.408     1.667     0.264
-------------------------------------------------------TTAGCAAGCTACTTCAGGGT-----------------------------------------------------------------     0.146     0.227     0.038
-------------------------------------------------------TTAGCAAGCTACTTCAGGGTAT---------------------------------------------------------------    16.853    10.722     3.351
-------------------------------------------------------TTAGCAAGCTACTTCAGGGTA----------------------------------------------------------------    10.782    11.783     3.878
-----------------------------------------------------TATTAGCAAGCTACTTCAGGGTAT---------------------------------------------------------------     0.146     0.379     0.379
---------------------------------------------TCG-CCACTATTAGCAAGCTACTTC----------------------------------------------------------------------     0.049     0.000     0.000
--------------------------------------GAGGACATCG-CCACT--------------------------------------------------------------------------------------     0.049     0.000     0.000
------------------------------------AGGAGGACATCG-CCACT--------------------------------------------------------------------------------------     0.049     0.000     0.000
------------------------------------AGGAGGACATCG-CCACTAT------------------------------------------------------------------------------------     0.049     0.000     0.000
----------------------------------TCAGGAGGACATCG-CCACTATT-----------------------------------------------------------------------------------     0.049     0.076     0.038
----------------------------------TCAGGAGGACATCG-CCACTA-------------------------------------------------------------------------------------     0.049     1.061     0.489
----------------------------------TCAGGAGGACATCG-CCACT--------------------------------------------------------------------------------------     0.243     3.523     1.167
----------------------------------TCAGGAGGACATCG-CCACTAT------------------------------------------------------------------------------------     0.146     0.076     0.038
---------------------------------CTCAGGAGGACATCG-CCAC---------------------------------------------------------------------------------------     0.097     0.076     0.075
---------------------------------CTCAGGAGGACATCG-CCACTA-------------------------------------------------------------------------------------     0.291     0.379     0.377
---------------------------------CTCAGGAGGACATCG-CCACTAT------------------------------------------------------------------------------------     0.097     0.189     0.113
---------------------------------CTCAGGAGGACATCG-CCACT--------------------------------------------------------------------------------------     0.971     0.493     0.602
--------------------------------TCTCAGGAGGACATCG-CCACT--------------------------------------------------------------------------------------    90.822   232.848   174.700
--------------------------------TCTCAGGAGGACATCG-CCACTAT------------------------------------------------------------------------------------    14.668    16.973    11.295
--------------------------------TCTCAGGAGGACATCG-CCACTATTAG---------------------------------------------------------------------------------     0.194     0.152     0.152
--------------------------------TCTCAGGAGGACATCG-CCACTA-------------------------------------------------------------------------------------    42.837   105.740    94.466
--------------------------------TCTCAGGAGGACATCG-CC-----------------------------------------------------------------------------------------     0.097     0.493     0.602
--------------------------------TCTCAGGAGGACATCG-CCA----------------------------------------------------------------------------------------     0.194     0.038     0.038
--------------------------------TCTCAGGAGGACATCG-CCACTATT-----------------------------------------------------------------------------------     1.457     2.160     0.339
--------------------------------TCTCAGGAGGACATCG-CCAC---------------------------------------------------------------------------------------     6.022    12.616    11.747
--------------------------------TCTCAGGAGGACATCG-C------------------------------------------------------------------------------------------     0.049     0.152     0.152
------------------------------TGTCTCAGGAGGACATCG-CCAC---------------------------------------------------------------------------------------     0.049     0.000     0.000
------------------------------TGTCTCAGGAGGACATCG-CC-----------------------------------------------------------------------------------------     0.049     0.000     0.000
---------------------CGCCGCACATGTCTCAGGAGG--------------------------------------------------------------------------------------------------     0.049     0.000     0.000
-------------------TGCGCCGCACATGTCTCAGG-----------------------------------------------------------------------------------------------------     0.389     0.341     0.264
-----------------TCTGCGCCGCACATGTCTCA-------------------------------------------------------------------------------------------------------     0.146     0.000     0.000
-----------------TCTGCGCCGCACATGTCTCAGG-----------------------------------------------------------------------------------------------------     0.097     0.000     0.000
-----------------TCTGCGCCGCACATGTCTCAG------------------------------------------------------------------------------------------------------     0.146     0.076     0.075
-----------------TCTGCGCCGCACATGTCTC--------------------------------------------------------------------------------------------------------     0.097     0.000     0.000

-------------                              C    -  AC  T         A  TC                       T                   A     A         C       AG
             GACGTCTGCGCCGCACATGTCTCAGGAGGA ATCG CC  TA TAGCAAGCT CT  AGGGTATCTCGGTCAGCATTTCG TTGGCAAGATGTCCGCTTC GGTAC CGCCGCTCA GCGATGA  C
             CTGCAGACGTGGCGTGTACAGAGTCCTTCT TAGC GG  AT ATCGTTCGA GA  TCCCATAGAGCCAGTCGTAGAGC AACCGTTCTGCAGGTGAGG CCATG GCGGCGAGT CGCTACT  /
GCCCGGTTATGCT                              C    A  C-  C         C  CC                       T                   C     C         A       GG

-------TATGCTCTGCAGACGTGGCGT----------------------------------------------------------------------------------------------------------------     0.680     0.038     0.038
------TTATGCTCTGCAGACGTGGCGT----------------------------------------------------------------------------------------------------------------     1.408     0.114     0.114
---------TGCTCTGCAGACGTGGCGT----------------------------------------------------------------------------------------------------------------     0.291     0.341     0.075
-----GTTATGCTCTGCAGACGTGGCGT----------------------------------------------------------------------------------------------------------------     0.049     0.000     0.000
--------ATGCTCTGCAGACGTGGCGT----------------------------------------------------------------------------------------------------------------     1.943     0.227     0.264
--------ATGCTCTGCAGACGTGGCGTG---------------------------------------------------------------------------------------------------------------     0.097     0.038     0.038
----------GCTCTGCAGACGTGGCGTG---------------------------------------------------------------------------------------------------------------     0.049     0.000     0.000
------TTATGCTCTGCAGACGTGGCGTGT--------------------------------------------------------------------------------------------------------------     1.603     0.227     0.227
--------ATGCTCTGCAGACGTGGCGTGT--------------------------------------------------------------------------------------------------------------    51.142     6.819     9.036
-----------CTCTGCAGACGTGGCGTGT--------------------------------------------------------------------------------------------------------------     0.146     0.076     0.038
-------TATGCTCTGCAGACGTGGCGTGT--------------------------------------------------------------------------------------------------------------     0.583     0.038     0.038
----------GCTCTGCAGACGTGGCGTGT--------------------------------------------------------------------------------------------------------------     1.263     0.758     0.414
--------------TGCAGACGTGGCGTGT--------------------------------------------------------------------------------------------------------------     0.049     0.000     0.000
-----GTTATGCTCTGCAGACGTGGCGTGT--------------------------------------------------------------------------------------------------------------     0.049     0.000     0.000
---------TGCTCTGCAGACGTGGCGTGT--------------------------------------------------------------------------------------------------------------     3.837     2.273     1.318
----GGTTATGCTCTGCAGACGTGGCGTGT--------------------------------------------------------------------------------------------------------------     0.049     0.000     0.000
------------TCTGCAGACGTGGCGTGT--------------------------------------------------------------------------------------------------------------     0.049     0.000     0.000
-----------------AGACGTGGCGTGTACAGAGT-------------------------------------------------------------------------------------------------------     1.020     0.758     0.904
---------------GCAGACGTGGCGTGTACAGAGT-------------------------------------------------------------------------------------------------------     0.826     1.856     0.640
--------------------CGTGGCGTGTACAGAGTCCT----------------------------------------------------------------------------------------------------     0.049     0.000     0.000
----------------------TGGCGTGTACAGAGTCCTTCT-------------------------------------------------------------------------------------------------     2.671     0.758     1.054
--------------------CGTGGCGTGTACAGAGTCCTTCT-------------------------------------------------------------------------------------------------     0.971     0.341     0.038
---------------------GTGGCGTGTACAGAGTCCTTCT-------------------------------------------------------------------------------------------------     0.826     1.099     0.791
-----------------------GGCGTGTACAGAGTCCTTCTCT-----------------------------------------------------------------------------------------------     0.389     0.303     0.339
----------------------------GTACAGAGTCCTTCTCTAGCAGGC----------------------------------------------------------------------------------------     0.146     0.000     0.000
---------------------------------GAGTCCTTCTCTAGCAGGC----------------------------------------------------------------------------------------     0.049     0.038     0.038
------------------------------ACAGAGTCCTTCTCTAGCAGGC----------------------------------------------------------------------------------------     0.971     0.871     0.871
-------------------------------CAGAGTCCTTCTCTAGCAGGC-A--------------------------------------------------------------------------------------     0.194     1.440     0.038
---------------------------------GAGTCCTTCTCTAGCAGGC-A--------------------------------------------------------------------------------------     0.291     1.174     2.523
------------------------------ACAGAGTCCTTCTCTAGCAGGC-A--------------------------------------------------------------------------------------     0.049     1.402     1.402
-----------------------------------GTCCTTCTCTAGCAGGC-AT-------------------------------------------------------------------------------------     0.049     0.076     0.038
--------------------------------------CTTCTCTAGCAGGC-AT-------------------------------------------------------------------------------------     0.049     0.000     0.000
---------------------------------GAGTCCTTCTCTAGCAGGC-AT-------------------------------------------------------------------------------------    29.675   238.000   185.807
-------------------------------------CCTTCTCTAGCAGGC-AT-------------------------------------------------------------------------------------     0.146     0.000     0.000
------------------------------------TCCTTCTCTAGCAGGC-AT-------------------------------------------------------------------------------------     0.049     0.000     0.000
----------------------------------AGTCCTTCTCTAGCAGGC-AT-------------------------------------------------------------------------------------     1.068     0.341     0.113
------------------------------ACAGAGTCCTTCTCTAGCAGGC-AT-------------------------------------------------------------------------------------     5.585   192.196     1.694
--------------------------------AGAGTCCTTCTCTAGCAGGC-AT-------------------------------------------------------------------------------------     7.140    21.595     0.226
-------------------------------CAGAGTCCTTCTCTAGCAGGC-AT-------------------------------------------------------------------------------------     6.945   188.067     1.845
-----------------------------TACAGAGTCCTTCTCTAGCAGGC-AT-------------------------------------------------------------------------------------     0.146     1.288     0.038
-------------------------------------CCTTCTCTAGCAGGC-ATCATCGT--------------------------------------------------------------------------------     0.243     2.728     0.377
---------------------------------------TTCTCTAGCAGGC-ATCATCGT--------------------------------------------------------------------------------     0.049     0.682     0.038
-----------------------------------------CTCTAGCAGGC-ATCATCGTT-------------------------------------------------------------------------------     0.097     0.114     0.038
----------------------------------------TCTCTAGCAGGC-ATCATCGTT-------------------------------------------------------------------------------     1.068     1.553     1.017
-------------------------------------CCTTCTCTAGCAGGC-ATCATCGTT-------------------------------------------------------------------------------     0.049     0.114     0.114
-------------------------------------------------------CATCGTTCGACGACCTC--------------------------------------------------------------------     0.049     0.000     0.000
-------------------------------------------------------CATCGTTCGACGACCTCCCA-----------------------------------------------------------------     0.097     0.075     0.075
------------------------------------------------------TCATCGTTCGACGACCTCCCA-----------------------------------------------------------------     0.049     0.152     0.038
------------------------------------------------------TCATCGTTCGACGACCTCCCAT----------------------------------------------------------------     0.340     0.152     0.188
-------------------------------------------------------CATCGTTCGACGACCTCCCAT----------------------------------------------------------------    30.695     9.585     7.493
---------------------------------------------------C-ATCATCGTTCGACGACCTCCCAT----------------------------------------------------------------     0.049     0.000     0.000
---------------------------------------------------------TCGTTCGACGACCTCCCAT----------------------------------------------------------------     0.049     0.000     0.000
-------------------------------------------------GGC-ATCATCGTTCGACGACCTCCCAT----------------------------------------------------------------     0.049     0.000     0.000
-----------------------------------------------------ATCATCGTTCGACGACCTCCCAT----------------------------------------------------------------     0.049     0.000     0.000
-----------------------------------------------------------------CGACCTCCCATAGAGCCAGT-------------------------------------------------------     0.049     0.038     0.038
--------------------------------------------------------------CGACGACCTCCCATAGAGCCAGT-------------------------------------------------------     0.097     0.038     0.038
---------------------------------------------------------------GACGACCTCCCATAGAGCCAGT-------------------------------------------------------     0.049     0.000     0.000
----------------------------------------------------------------ACGACCTCCCATAGAGCCAGT-------------------------------------------------------     0.146     0.000     0.000
-------------------------------------------------------------------ACCTCCCATAGAGCCAGTCGT----------------------------------------------------     0.146     0.265     0.113
------------------------------------------------------------------GACCTCCCATAGAGCCAGTCGT----------------------------------------------------     0.049     0.038     0.038
----------------------------------------------------------------------TCCCATAGAGCCAGTCGTAGA-------------------------------------------------     0.389     0.417     0.339
-------------------------------------------------------------------------CATAGAGCCAGTCGTAGAGCT----------------------------------------------     0.146     0.303     0.565
-----------------------------------------------------------------------------GAGCCAGTCGTAGAGCTAA--------------------------------------------     0.049     0.000     0.000
----------------------------------------------------------------------------AGAGCCAGTCGTAGAGCTAA--------------------------------------------     0.146     0.493     0.377 miR_t51*
---------------------------------------------------------------------------TAGAGCCAGTCGTAGAGCTAA--------------------------------------------     0.194     0.417     0.151
------------------------------------------------------------------------------AGCCAGTCGTAGAGCTAACCG-----------------------------------------     0.097     0.113     0.113
-------------------------------------------------------------------------------GCCAGTCGTAGAGCTAACCG-----------------------------------------     0.097     0.303     0.339
------------------------------------------------------------------------------AGCCAGTCGTAGAGCTAACCGT----------------------------------------     0.437     0.189     0.188
-------------------------------------------------------------------------------GCCAGTCGTAGAGCTAACCGT----------------------------------------     4.857     7.805     9.977
----------------------------------------------------------------------------------AGTCGTAGAGCTAACCGTTCT-------------------------------------     0.194     0.076     0.076
----------------------------------------------------------------------------------------AGAGCTAACCGTTCTGCAGGT-------------------------------     0.049     0.000     0.000
---------------------------------------------------------------------------------------------TAACCGTTCTGCAGGTGAGGC--------------------------     0.049     0.000     0.000
--------------------------------------------------------------------------------------------CTAACCGTTCTGCAGGTGAGGC--------------------------     0.097     0.038     0.038
-------------------------------------------------------------------------------------------GCTAACCGTTCTGCAGGTGAGGC--------------------------     0.097     0.000     0.000
-------------------------------------------------------------------------------------------------CGTTCTGCAGGTGAGGCC-------------------------     0.049     0.000     0.000
------------------------------------------------------------------------------------------------CCGTTCTGCAGGTGAGGCC-------------------------     0.049     0.000     0.000
-------------------------------------------------------------------------------------------------CGTTCTGCAGGTGAGGCCC------------------------     1.214     0.568     1.958
------------------------------------------------------------------------------------------------CCGTTCTGCAGGTGAGGCCC------------------------     0.923     0.189     0.301
-------------------------------------------------------------------------------------------------CGTTCTGCAGGTGAGGCCCA-----------------------     5.828     5.910    26.694
------------------------------------------------------------------------------------------------CCGTTCTGCAGGTGAGGCCCA-----------------------     5.440    25.687    25.979
--------------------------------------------------------------------------------------------------GTTCTGCAGGTGAGGCCCAT----------------------     3.788     6.857     5.723
----------------------------------------------------------------------------------------------------TCTGCAGGTGAGGCCCAT----------------------     0.049     0.038     0.038
-----------------------------------------------------------------------------------------------ACCGTTCTGCAGGTGAGGCCCAT----------------------     2.137     9.434     0.188
-------------------------------------------------------------------------------------------------------GCAGGTGAGGCCCAT----------------------     0.049     0.000     0.000
------------------------------------------------------------------------------------------------CCGTTCTGCAGGTGAGGCCCAT----------------------   608.802   813.717   501.810
-------------------------------------------------------------------------------------------------CGTTCTGCAGGTGAGGCCCAT----------------------   752.467  1662.972  2815.566 miR_t12
-----------------------------------------------------------------------------------------------------CTGCAGGTGAGGCCCAT----------------------     0.049     0.038     0.075
---------------------------------------------------------------------------------------------------TTCTGCAGGTGAGGCCCAT----------------------     0.194     0.114     0.301
```

miRNA: miR_t88,miR_t53 chromosome_2 Start: 9173113 End: 9173421 Condition: TAP

```
-----------------------------------------------------------------------------------------------------------------------------------------------------------------------------GGAGGGCGCAAGCACGCAAC-------------------------------------------     0.291     0.038     0.339
-----------------------------------------------------------------------------------------------------------------------------------------------------------------------------GGAGGGCGCAAGCACGCAACG------------------------------------------     0.291     0.038     0.452
----------------------------------------------------------------------------------------------------------------------------------------------------------------------------TGGAGGGCGCAAGCACGCAACGA-----------------------------------------     0.049     0.000     0.000
----------------------------------------------------------------------------------------------------------------------------------------------------------------------------TGGAGGGCGCAAGCACGCA---------------------------------------------     0.049     0.188     0.188
----------------------------------------------------------------------------------------------------------------------------------------------------------------------------TGGAGGGCGCAAGCACGCAAC-------------------------------------------    29.772     3.372    13.856
----------------------------------------------------------------------------------------------------------------------------------------------------------------------------TGGAGGGCGCAAGCACGCAA--------------------------------------------     0.097     0.038     0.038
----------------------------------------------------------------------------------------------------------------------------------------------------------------------------TGGAGGGCGCAAGCACGCAACG------------------------------------------    47.305    12.957    23.871 miR_t88
--------------------------------------------------------------------------------------------------------------------------------------------------------------------TAGACGGCTGGAGGGCGCAAG---------------------------------------------------     0.243     0.303     0.565
----TGATGCCCCACCAGCAG-------------------------------------------------------------------------------------------------------------------------------------------------------------------------------------------------------------------------------     0.049     0.000     0.000

TTGGTGATGCCCCACCAGCAGGGCAAGCAGCAGCAAGTGCGGGCGGCCATGTTCCGCGGCCGGGCCCGCCGCCGGGGCCGCAGCAGCATGTATGTATGATGGAGCGGCTAGCTGACGACACGGGTGCTGGCAGCAGCCAGGCAGGCAGCCGCCGCCTGATGA                                                    C             CCT  TC
                                                                                                                                                                  GCTAGACGGCTGGAGGGCGCAAGCACGCAACGAGGGGATGACGAGCACGATG GGGCCTGATACAG   CG  C
                                                                                                                                                                  CGATCTGCCGACCTCCCGCGTTCGTGCGTTGCTCCCCTACTGCTCGTGCTAC CCCGGACTATGTC   GC  /
-----------------------------------------------------------------------------------------------------------------------------------------------------------------C                                                    A             C--  CC

---------------------------------------------------------------------------------------------------------------------------------------------------------------------TCTGCCGACCTCCCGCGTTCGT-------------------------------------------------     0.097     0.038     0.038
-----------------------------------------------------------------------------------------------------------------------------------------------------------------------TGCCGACCTCCCGCGTTCGT-------------------------------------------------     0.049     0.038     0.038
----------------------------------------------------------------------------------------------------------------------------------------------------------------------CTGCCGACCTCCCGCGTTCGT-------------------------------------------------     0.971     0.152     0.678
--------------------------------------------------------------------------------------------------------------------------------------------------------------------------CGACCTCCCGCGTTCGTGCGT---------------------------------------------     2.817     0.871     1.355
--------------------------------------------------------------------------------------------------------------------------------------------------------------------------CGACCTCCCGCGTTCGTGCGTT--------------------------------------------    19.670    15.117    10.354 miR_t88*
----------------------------------------------------------------------------------------------------------------------------------------------------------------------------ACCTCCCGCGTTCGTGCGTT--------------------------------------------     0.583     0.038     0.038
-----------------------------------------------------------------------------------------------------------------------------------------------------------------------------CCTCCCGCGTTCGTGCGTT--------------------------------------------     0.194     0.152     0.075
---------------------------------------------------------------------------------------------------------------------------------------------------------------------------GACCTCCCGCGTTCGTGCGTT--------------------------------------------     0.389     0.152     0.113
-----------------------------------------------------------------------------------------------------------------------------------------------------------------------------------GCGTTCGTGCGTTGCTCCCCT------------------------------------     0.243     0.076     0.151
------------------------------------------------------------------------------------------------------------------------------------------------------------------------------------CGTTCGTGCGTTGCTCCCCT------------------------------------     0.146     0.265     0.264
-------------------------------------------------------------------------------------------------------------------------------------------------------------------------------------GTTCGTGCGTTGCTCCCCTACT---------------------------------     0.097     0.000     0.000
----------------------------------------------------------------------------------------------------------------------------------------------------------------------------------------CGTGCGTTGCTCCCCTACT---------------------------------     0.049     0.038     0.075
---------------------------------------------------------------------------------------------------------------------------------------------------------------------------------------TCGTGCGTTGCTCCCCTACTGCT------------------------------     0.049     0.000     0.000
----------------------------------------------------------------------------------------------------------------------------------------------------------------------------------------CGTGCGTTGCTCCCCTACTGCT------------------------------     0.049     0.000     0.000
-----------------------------------------------------------------------------------------------------------------------------------------------------------------------------------------GTGCGTTGCTCCCCTACTGCT------------------------------     0.291     0.075     0.075
---------------------------------------------------------------------------------------------------------------------------------------------------------------------------------------------GTTGCTCCCCTACTGCTCG----------------------------     0.146     0.114     0.113
```

```
------------------------------------------------------------------------------------------------------------------------------------------------------------------------------------GCGTTGCTCCCCTACTGCTCG---------------------------      0.146      0.227      0.075
-------------------------------------------------------------------------------------------------------------------------------------------------------------------------------------CGTTGCTCCCCTACTGCTCG---------------------------      0.146      0.038      0.113
-------------------------------------------------------------------------------------------------------------------------------------------------------------------------------------CGTTGCTCCCCTACTGCTCGT--------------------------      4.128      1.212      1.619
--------------------------------------------------------------------------------------------------------------------------------------------------------------------------------------GTTGCTCCCCTACTGCTCGT--------------------------      2.186      0.909      1.280
------------------------------------------------------------------------------------------------------------------------------------------------------------------------------------GCGTTGCTCCCCTACTGCTCGT--------------------------      5.585      4.774      1.770
---------------------------------------------------------------------------------------------------------------------------------------------------------------------------------------TTGCTCCCCTACTGCTCGT--------------------------      0.049      0.000      0.000
----------------------------------------------------------------------------------------------------------------------------------------------------------------------------------------TGCTCCCCTACTGCTCGTG-------------------------      0.049      0.000      0.000
-----------------------------------------------------------------------------------------------------------------------------------------------------------------------------------------GCTCCCCTACTGCTCGTG-------------------------      0.971      0.114      0.414
-----------------------------------------------------------------------------------------------------------------------------------------------------------------------------------------GCTCCCCTACTGCTCGTGC------------------------      2.768      0.682      1.431
-----------------------------------------------------------------------------------------------------------------------------------------------------------------------------------------GCTCCCCTACTGCTCGTGCT-----------------------    413.461    144.725    116.303 miR_t53
----------------------------------------------------------------------------------------------------------------------------------------------------------------------------------------TGCTCCCCTACTGCTCGTGCT-----------------------     13.551      1.326      1.167

miRNA: miR_t60,miR_t113 chromosome_13 Start: 2001058 End: 2001210 Condition: TAP
-------TAAGTCAGCAAGCAGTCAGG---------------------------------------------------      0.049      0.265      0.377
---------AGTCAGCAAGCAGTCAGGTAG------------------------------------------------      0.049      0.341      0.452
---------AGTCAGCAAGCAGTCAGGTAGA-----------------------------------------------      0.194      2.993      0.753
-----------TCAGCAAGCAGTCAGGTAGAAGCG-------------------------------------------      0.049      0.114      0.114
-----------TCAGCAAGCAGTCAGGTAGAAG---------------------------------------------      1.991      3.902      2.974
-----------TCAGCAAGCAGTCAGGTAGA-----------------------------------------------     16.610     50.692     76.958
-----------TCAGCAAGCAGTCAGGTAG------------------------------------------------      0.146      0.303      0.038
-----------TCAGCAAGCAGTCAGGTAGAA----------------------------------------------      3.885      5.153      6.099
-------------AGCAAGCAGTCAGGTAGAAGCG-------------------------------------------      0.049      0.341      0.341
-------------AGCAAGCAGTCAGGTAGAA----------------------------------------------      0.049      0.000      0.000
--------------GCAAGCAGTCAGGTAGAAGCG-------------------------------------------      0.243      1.894      0.979
---------------CAAGCAGTCAGGTAGAAGCG-------------------------------------------      0.194      1.326      1.468
----------------AAGCAGTCAGGTAGAAGCGC------------------------------------------      0.049      0.038      0.339
----------------AAGCAGTCAGGTAGAAGCG-------------------------------------------      0.049      0.189      0.188
---------------------------TAGAAGCGCCGAGTAGGAG--------------------------------      0.049      0.000      0.000
---------------------------TAGAAGCGCCGAGTAGGAGTGAG----------------------------      0.049      0.000      0.000
---------------------------TAGAAGCGCCGAGTAGGAGTGA-----------------------------      0.049      0.000      0.000
-----------------------------------CCGAGTAGGAGTGAGAGTCAG----------------------     15.736      3.220      5.346
---------------------------------------GTAGGAGTGAGAGTCAG----------------------      0.049      0.000      0.000
----------------------------------------TAGGAGTGAGAGTCAG----------------------      0.729      0.000      0.000
----------------------------------------TAGGAGTGAGAGTCAGTGGGG-----------------      5.634      3.978      2.937
----------------------------------------TAGGAGTGAGAGTCAGTGGG------------------      0.340      1.440      2.673
----------------------------------------TAGGAGTGAGAGTCAGTGG-------------------      3.740      8.790     15.625
----------------------------------------TAGGAGTGAGAGTCAGT---------------------      0.146      0.758      0.941
----------------------------------------TAGGAGTGAGAGTCAGTGGGGT----------------      0.097      0.000      0.000
----------------------------------------TAGGAGTGAGAGTCAGTGGGGTAT--------------      0.049      0.000      0.000
----------------------------------------TAGGAGTGAGAGTCAGTG--------------------      0.146      0.076      0.113
-----------------------------------------AGGAGTGAGAGTCAGTGGGG-----------------      0.194      0.038      0.038
----------------------------------------------TGAGAGTCAGTGGGGTATG-------------      0.097      0.189      1.355
--------------------------------------------------------TGGGGTATGTAAGCGTCTTG--      0.291      0.038      0.075
--------------------------------------------------------TGGGGTATGTAAGCGTCTTGC-     13.211     46.373     54.707
--------------------------------------------------------TGGGGTATGTAAGCGTCT----      0.146      0.114      0.038
---------------------------------------------------------GGGGTATGTAAGCGTCTTGC-      0.146      0.265      0.753

---CGG T                    A                           T              T  TGC
      C AAGTCAGCAAGCAGTCAGGT GAAGCGCCGAGTAGGAGTGAGAGTCAG GGGGTATGTAAGCG CT   C
      G TTCAGTCGTTCGTCAGTCCA CTTCGCGGCTCATCCTCACTCTCAGTC CCCCATATATTCGC GA   /
ATTGTA C                    C                           T              C  TTT

-------------------------------------------------------CTCCCCATATATTCGCCGATG--      0.049      0.000      0.000
------------------------------------------------------TCTCCCCATATATTCGCCGAT---      0.097      0.075      0.075
------------------------------------------CCTCACTCTCAGTCTCCCCAT---------------      0.049      0.000      0.000
----------------------------------CGGCTCATCCTCACTCTCAGTCT---------------------      0.243      0.303      0.753
-----------------------------------GGCTCATCCTCACTCTCAGTCT---------------------      0.826      0.909      0.527
------------------------------------GCTCATCCTCACTCTCAGTCT---------------------      0.583      0.076      0.301
----------------------------------CGGCTCATCCTCACTCTCAGTC----------------------      2.623      4.887      5.535
--------------------------------CGCGGCTCATCCTCACTCTCAGTC----------------------      0.194      0.000      0.000
--------------------------------CGCGGCTCATCCTCACTCTCAGT-----------------------     24.430     13.222      1.920
-----------------------------------GGCTCATCCTCACTCTCAGT-----------------------      8.548      4.357      3.878
---------------------------------GCGGCTCATCCTCACTCTCAGT-----------------------     13.356      4.887      7.154
----------------------------------CGGCTCATCCTCACTCTCAGT-----------------------   1806.833   2599.440   3533.718 miR_t60
-------------------------------TCGCGGCTCATCCTCACTCTCAGT-----------------------      0.340      0.000      0.000
------------------------------------GCTCATCCTCACTCTCAGT-----------------------      0.097      0.076      0.113
---------------------------------GCGGCTCATCCTCACTCTCAG------------------------      0.777      0.227      0.301
--------------------------------CGCGGCTCATCCTCACTCTCAG------------------------      1.311      0.303      0.301
----------------------------------CGGCTCATCCTCACTCTCAG------------------------     27.781     31.673     72.252
----------------------------------CGGCTCATCCTCACTCTCA-------------------------      5.440      0.076      0.452
--------------------------------CGCGGCTCATCCTCACTCTCA-------------------------      0.194      0.113      0.113
---------------------------------GCGGCTCATCCTCACTCTCA-------------------------      0.049      0.114      0.038
---------------------------------GCGGCTCATCCTCACTCT---------------------------      0.194      0.000      0.000
------------------------------TTCGCGGCTCATCCTCACTCT---------------------------      0.049      0.000      0.000
--------------------------------CGCGGCTCATCCTCACTCT---------------------------      1.214      0.000      0.000
-------------------------------TCGCGGCTCATCCTCACT-----------------------------      0.049      0.000      0.000
----------------------------CCTTCGCGGCTCATCCTCACT-----------------------------      0.049      0.000      0.000
--------------------TCAGTCCACCTTCGCGGCTCAT------------------------------------      0.049      0.000      0.000
---------------------CAGTCCACCTTCGCGGCTCAT------------------------------------      0.437      0.189      0.189
----------------------AGTCCACCTTCGCGGCTCAT------------------------------------      0.243      0.038      0.038
------------------CGTCAGTCCACCTTCGCGGCT---------------------------------------      5.003      3.448      3.727
------------------CGTCAGTCCACCTTCGCGGC----------------------------------------      0.049      0.076      0.076
-----------AGTCGTTCGTCAGTCCACCTTC---------------------------------------------      0.389      0.038      0.038
------------GTCGTTCGTCAGTCCACCTTC---------------------------------------------      2.914      4.357      2.447
--------------CGTTCGTCAGTCCACCTT----------------------------------------------      0.923      3.296      2.523
----------CAGTCGTTCGTCAGTCCACCTT----------------------------------------------      0.340      0.038      0.038
-------------TCGTTCGTCAGTCCACCTT----------------------------------------------      0.340      1.440      0.452
------------GTCGTTCGTCAGTCCACCTT----------------------------------------------     30.355     10.911      6.739
---------------GTTCGTCAGTCCACCTT----------------------------------------------      0.049      0.000      0.000
-----------AGTCGTTCGTCAGTCCACCTT----------------------------------------------    601.274    109.491     38.893 miR_t113
---------TCAGTCGTTCGTCAGTCCACCTT----------------------------------------------      0.049      0.076      0.076
-----------AGTCGTTCGTCAGTCCACCT-----------------------------------------------     10.636      2.046      0.753
----------CAGTCGTTCGTCAGTCCACCT-----------------------------------------------      0.389      0.152      0.038
--------------CGTTCGTCAGTCCACCT-----------------------------------------------      0.049      0.455      0.188
------------GTCGTTCGTCAGTCCACCT-----------------------------------------------      2.088      0.871      0.904
---------TCAGTCGTTCGTCAGTCCACCT-----------------------------------------------      0.097      0.189      0.189
-----------AGTCGTTCGTCAGTCCACC------------------------------------------------      0.097      0.000      0.000
-------CTTCAGTCGTTCGTCAGTCCAC-------------------------------------------------      0.874      2.993      0.941
----------CAGTCGTTCGTCAGTCCAC-------------------------------------------------      0.049      0.000      0.000
-----------AGTCGTTCGTCAGTCCAC-------------------------------------------------      1.020      0.038      0.038
------------GTCGTTCGTCAGTCCAC-------------------------------------------------      0.049      0.038      0.038
-----------AGTCGTTCGTCAGTCC---------------------------------------------------      0.049      0.000      0.000
-----AGCTTCAGTCGTTCGTCAGT-----------------------------------------------------      0.291      0.341      0.341
----TAGCTTCAGTCGTTCGTCAGT-----------------------------------------------------      0.049      0.227      0.227

miRNA: miR_t61 chromosome_17 Start: 3137537 End: 3137719 Condition: TAP
-------------------------------------------------------------------TTCAGTCTTACTTGCTT--GGCTTAC--      0.049      0.075      0.075
----------------------------------------------------------------TGCTTCAGTCTTACTTGCTT--G--------      0.146      0.038      0.038
----------------------------------------------------------------TGCTTCAGTCTTACTTGCTT--GG-------      0.097      0.000      0.000
--------------------------------------------------TGGTCGACGGCAATTGCTTCAGT----------------------      0.049      0.000      0.000
--------------------------------------------------TGGTCGACGGCAATTGCTTCAG-----------------------      0.631      1.174      1.657
--------------------------------------------------TGGTCGACGGCAATTGCTTCA------------------------      0.049      0.076      0.076
-----------------------------------------------TGGTGGTCGACGGCAAT-------------------------------      0.049      0.000      0.000
----------------------------------------------GTGGTGGTCGACGGCAATTGC----------------------------      0.049      0.303      0.452
---------------------------------------------AGTGGTGGTCGACGGCAATTGC----------------------------      0.049      0.227      0.227
--------------------------------------------GAGTGGTGGTCGACGGCAAT-------------------------------      0.146      0.264      0.264
-------------------------------------------TGAGTGGTGGTCGACGGCAA--------------------------------      0.486      0.417      0.414
-------------------------------------------TGAGTGGTGGTCGACGGCAATTG-----------------------------      0.049      0.038      0.151
-------------------------------------------TGAGTGGTGGTCGACGGCAAT-------------------------------    146.336     75.810     72.403 miR_t61
-------------------------------------------TGAGTGGTGGTCGACGGCA---------------------------------      0.194      0.038      0.113
-------------------------------------------TGAGTGGTGGTCGACGG-----------------------------------      0.049      0.530      1.130
-------------------------------------------TGAGTGGTGGTCGACGGCAATT------------------------------      2.914      3.940      7.530
------------------------------------------TTGAGTGGTGGTCGACGGCAAT-------------------------------      0.583      0.720      0.075
------------------------------------------TTGAGTGGTGGTCGACGGCAA--------------------------------      0.194      0.303      0.264
-----------------------------------------TTTGAGTGGTGGTCGACGGCAAT-------------------------------      0.631      0.227      0.038
-----------------------------------------TTTGAGTGGTGGTCGACGGCAA--------------------------------      0.389      0.301      0.301
--------------------------------------TGGTTTGAGTGGTGG------------------------------------------      0.049      0.000      0.000
--------------------------------------TGGTTTGAGTGGTGGTCG---------------------------------------      0.777      0.227      0.377
--------------------------------------TGGTTTGAGTGGTGGTCGACG------------------------------------      0.194      0.075      0.075
--------------------------------------TGGTTTGAGTGGTGGTCGACGGCA---------------------------------      0.049      0.000      0.000
--------------------------------------TGGTTTGAGTGGTGGTCGA--------------------------------------      0.049      0.038      0.038
-----------------------GGTGCAACAGGCCAGTGGTT----------------------------------------------------      0.049      0.000      0.000
----------------------TGGTGCAACAGGCCAGTGGT-----------------------------------------------------      0.049      0.000      0.000
----------------------TGGTGCAACAGGCCAGTGGTT----------------------------------------------------      1.214      0.303      0.301
------TA----GGTGCTCTTCTGGTGCAAC----------------------------------------------------------------      0.049      0.000      0.000

--T   TA----        T                         G         A                           --   T   G
   GCA      GGTGCTCT CTGGTGCAACAGGCCAGTGGTTTGA TGGTGGTCG CGGCAATTGCTTCAGTCTTACTTGCTT  GGC TAC T
   CGT      TCACGAGA GACCACGTTGTCCGGTCACCAAACT GCCACCAGC GTCGTTAACGAAGTCAGAATGAACGAA  CCG ATG C
CGC   CCCAAA        C                         A         C                           CT   C   C

---------AAATCACGAGACGACCACGT------------------------------------------------------------------      0.146      0.000      0.000
-------CCAAATCACGAGACGACCACGT------------------------------------------------------------------      0.097      0.038      0.038
-----TCCCAAATCACGAGACGACCACGT------------------------------------------------------------------      0.049      0.000      0.000
--------CAAATCACGAGACGACCACGT------------------------------------------------------------------      0.291      0.038      0.151
----GTCCCAAATCACGAGACGACCACGT------------------------------------------------------------------      0.049      0.000      0.000
------CCCAAATCACGAGACGACCACGT------------------------------------------------------------------      0.097      0.000      0.000
------CCCAAATCACGAGACGACCACGTT-----------------------------------------------------------------      0.049      0.189      0.189
----GTCCCAAATCACGAGACGACCACGTT-----------------------------------------------------------------      0.049      0.038      0.038
-------CCAAATCACGAGACGACCACGTT-----------------------------------------------------------------      0.097      0.227      0.227
---------------CGAGACGACCACGTTGTCCGGT----------------------------------------------------------      0.146      1.212      0.489
----------------GAGACGACCACGTTGTCCGGT----------------------------------------------------------      0.146      0.038      0.038
---------------------------GTTGTCCGGTCACCAAA---------------------------------------------------      0.923      0.189      0.038
--------------------------CGTTGTCCGGTCACCAAACT-------------------------------------------------      0.194      0.000      0.000
---------------------------GTTGTCCGGTCACCAAACTAG-----------------------------------------------      0.777     10.343      4.819
------------------------------GTCCGGTCACCAAACTAGCCA--------------------------------------------      0.049      0.000      0.000
-----------------------------------------AAACTAGCCACCAGCCGTCGT---------------------------------      0.097      0.000      0.000 miR_t61*
------------------------------------------AACTAGCCACCAGCCGTCGTT--------------------------------      0.049      0.000      0.000
---------------------------------------------------CCAGCCGTCGTTAACGAAGT------------------------      0.049      0.000      0.000
------------------------------------------------------GCCGTCGTTAACGAAGTCAGA--------------------      0.049      0.000      0.000
--------------------------------------------------------CGTCGTTAACGAAGTCAGAAT------------------      0.146      0.076      0.075
-------------------------------------------------------CCGTCGTTAACGAAGTCAGAAT------------------      2.137      1.440      0.226
------------------------------------------------------GCCGTCGTTAACGAAGTCAGAAT------------------      0.437      0.265      0.038

miRNA: miR_t66,miR_t65 chromosome_11 Start: 1442246 End: 1442394 Condition: TAP
----------------CTGTTTACCAACGTGCAGGGGG--------------------------------------------      0.049      0.000      0.000
```

```
----------------CTGTTTACCAACGTGCAGGGG---------------------------------------------    0.097    0.000    0.000
-----------------TGTTTACCAACGTGCAGGGGG--------------------------------------------    5.003    0.455    1.167
-----------------TGTTTACCAACGTGCAGGG----------------------------------------------    0.049    0.038    0.038
-----------------TGTTTACCAACGTGCAGGGG---------------------------------------------    2.428    0.379    0.377
-------------------TTTACCAACGTGCAGGGGG--------------------------------------------    0.097    0.038    0.038
-------------------TTTACCAACGTGCAGGGGGACT-----------------------------------------    0.194    1.364    0.377
-------------------TTTACCAACGTGCAGG-----------------------------------------------    0.049    0.000    0.000
-------------------TTTACCAACGTGCAGGGGGAC------------------------------------------    1.214    0.720    1.280
-------------------TTTACCAACGTGCAGGGGGA-------------------------------------------    0.534    0.114    0.188
--------------------TTACCAACGTGCAGGGGGACTTAG--------------------------------------    0.049    0.000    0.000
--------------------TTACCAACGTGCAGGGGGA-------------------------------------------    0.097    0.000    0.000
--------------------TTACCAACGTGCAGGGGGACT-----------------------------------------    0.291    0.227    0.226
---------------------TACCAACGTGCAGGGGGACT-----------------------------------------    0.146    0.038    0.151
-----------------------CCAACGTGCAGGGGGACTTAG--------------------------------------    0.049    0.227    0.227
-------------------------AACGTGCAGGGGGACTTAGTGG-----------------------------------    0.291    3.978    3.978
-----------------------------TGCAGGGGGACTTAGTGGAG---------------------------------   78.826   57.511   81.627
-----------------------------TGCAGGGGGACTTAGTGGAGATCC-----------------------------    0.049    0.000    0.000
-----------------------------TGCAGGGGGACTTAGTGGAGA--------------------------------  227.833  102.974  106.589 miR_t66
-----------------------------TGCAGGGGGACTTAGTGG-----------------------------------    0.874    4.471    1.920
-----------------------------TGCAGGGGGACTTAG--------------------------------------    0.049    0.000    0.000
-----------------------------TGCAGGGGGACTTAGTGGA----------------------------------    8.014   16.897    8.660
-----------------------------TGCAGGGGGACTTAGTGGAGAT-------------------------------    1.020    0.114    0.678
-----------------------------TGCAGGGGGACTTAGTGGAGATC------------------------------    0.097    0.038    0.038
------------------------------GCAGGGGGACTTAGTGGAG---------------------------------    0.583    0.114    0.565
------------------------------GCAGGGGGACTTAGTGGAGAT-------------------------------    0.049    0.000    0.000
------------------------------GCAGGGGGACTTAGTGGAGA--------------------------------    1.408    0.417    1.318
-------------------------------CAGGGGGACTTAGTGGAGATC------------------------------    0.826    2.046    4.706
-------------------------------CAGGGGGACTTAGTGGAGA--------------------------------    0.049    0.114    0.114
--------------------------------AGGGGGACTTAGTGGAGA--------------------------------    0.049    0.000    0.000
--------------------------------AGGGGGACTTAGTGGAG---------------------------------    0.049    0.000    0.000
----------------------------------GGGGACTTAGTGGAGA--------------------------------    0.097    0.000    0.000
-----------------------------------------TAGTGGAGATCCTCCTGTCCGG-------------------    0.097    0.455    0.339
-----------------------------------------TAGTGGAGATCCTCCTGTCCG--------------------    0.923    0.985    0.188
-----------------------------------------TAGTGGAGATCCTCCTGTCCGGC------------------    0.049    0.038    0.226
--------------------------------------------TGGAGATCCTCCTGTCCGGC------------------    2.234   22.239   10.994
--------------------------------------------TGGAGATCCTCCTGTCCGGCT---ACCTGG--------    0.049    0.076    0.076
--------------------------------------------TGGAGATCCTCCTGTCCGGCT-----------------    1.408    1.212    0.753
--------------------------------------------TGGAGATCCTCCTGTCCGG-------------------    0.049    0.114    0.038
--------------------------------------------------TCCTCCTGTCCGGCT---ACCTGG--------    0.049    0.000    0.000
--------------------------------------------------------TGTCCGGCT---ACCTGGAGCGCAAG    0.000    0.038    0.038

------------TTA                                                  ---    GGA  G AA
               GCTGTTTACCAACGTGCAGGGGGACTTAGTGGAGATCCTCCTGTCCGGCT   ACCT   GC C  C
               TGACAAATGGTTGCACGTCCCCCTGAATCACCTCTAGGAGGACAGGCCGA   TGGA   CG G  /
CGAACGCGAGGTCCC                                                  TTT    GAG  G TT

------------------------------CGTCCCCCTGAATCACCTCT--------------------------------    0.534    0.038    0.226
-----------------------------ACGTCCCCCTGAATCACCTCT--------------------------------    5.585    0.909    1.393
----------------------------CACGTCCCCCTGAATCACCT----------------------------------    0.049    0.038    0.038
---------------------------GCACGTCCCCCTGAATCACCT----------------------------------    2.768    1.819    2.334 miR_t66*
--------------------------TGCACGTCCCCCTGAATCACCT----------------------------------    0.049    0.000    0.000
-------------------------------GTCCCCCTGAATCACCT----------------------------------    0.049    0.000    0.000
---------------------------GCACGTCCCCCTGAATCAC------------------------------------    0.049    0.038    0.075
----------------------TGGTTGCACGTCCCCCTGAAT---------------------------------------    0.389    0.000    0.000
-----------------------GGTTGCACGTCCCCCTGAAT---------------------------------------    0.777    0.606    0.188
----------------GACAAATGGTTGCACGTCCCCCT-------------------------------------------    0.291    1.023    1.023
---------------TGACAAATGGTTGCACGTCCCCCT-------------------------------------------    0.049    0.152    0.152
------------CCCTGACAAATGGTTGCACGTC------------------------------------------------    0.389    0.227    0.226
---------------TGACAAATGGTTGCACGT-------------------------------------------------    0.243    0.265    0.265
-------------CCTGACAAATGGTTGCACGT-------------------------------------------------    7.334   40.803   20.331
-----------TCCCTGACAAATGGTTGCACGT-------------------------------------------------    0.971    1.515    0.941
----------------GACAAATGGTTGCACGT-------------------------------------------------    0.049    0.000    0.000
--------------CTGACAAATGGTTGCACGT-------------------------------------------------    0.437    0.152    0.339
------CGAGGTCCCTGACAAATGGTTGCACGT-------------------------------------------------    0.049    0.000    0.000
----------GTCCCTGACAAATGGTTGCACGT-------------------------------------------------    2.720    5.910    1.242
------------CCCTGACAAATGGTTGCACGT-------------------------------------------------  993.948  593.713  670.674 miR_t65
-------GAGGTCCCTGACAAATGGTTGCACGT-------------------------------------------------    0.146    0.265    0.265
-----------------ACAAATGGTTGCACGT-------------------------------------------------    0.097    0.000    0.000
---------GGTCCCTGACAAATGGTTGCACGT-------------------------------------------------    2.526    5.001    0.452
----------GTCCCTGACAAATGGTTGCACG--------------------------------------------------    0.049    0.000    0.000
------------CCCTGACAAATGGTTGCACG--------------------------------------------------    3.303    0.833    4.518
------------CCCTGACAAATGGTTGCAC---------------------------------------------------    0.049    0.000    0.000
------------CCCTGACAAATGGTTGCA----------------------------------------------------    0.194    0.076    0.301
-------GAGGTCCCTGACAAATGGTTGC-----------------------------------------------------    0.389    0.189    0.189
----CGCGAGGTCCCTGACAAATGGTT-------------------------------------------------------    0.049    0.227    0.075
------CGAGGTCCCTGACAAATGGTT-------------------------------------------------------    4.225    7.767    1.920
-------GAGGTCCCTGACAAATGGTT-------------------------------------------------------    1.554    1.099    0.941
-----GCGAGGTCCCTGACAAATGGTT-------------------------------------------------------    0.437    0.417    0.038
------CGAGGTCCCTGACAAATGGT--------------------------------------------------------    0.049    0.189    0.038
-----GCGAGGTCCCTGACAAAT-----------------------------------------------------------    0.049    0.000    0.000
```

miRNA: miR_t77,miR_t58 chromosome_3 Start: 6573885 End: 6573999 Condition: TAP

```
---------------------------------TCACCGCCGGAACCAACACGA---    0.049    0.000    0.000
---------------------------------TCACCGCCGGAACCAACACGAC--    0.097    0.038    0.038
---------------------------------TCACCGCCGGAACCAACACG----    3.157    1.781    1.431
-------------------------------TGTCACCGCCGGAACCAACACG----    0.049    0.114    0.114
----------------------TTCACCGCATGTCACCGCCGG--------------    0.243    0.000    0.000
-----------------TCACATTCACCGCATGTCACC-------------------    2.234    0.493    0.377 miR_t58*
-----------------TCACATTCACCGCATGTCAC--------------------    0.049    0.114    0.114
----------------TTCACATTCACCGCATGTCAC--------------------    0.194    0.265    0.075
----------------TTCACATTCACCGCATGTCACC-------------------    0.583    0.227    0.038
---------------ATTCACATTCACCGCATGTCA----------------------    0.049    0.038    0.038
---------------ATTCACATTCACCGCATG-------------------------    0.049    0.000    0.000
-------------TCATTCACATTCACCGCATG-------------------------    0.243    0.000    0.000
-------------TCATTCACATTCACCGCATGTC-----------------------    0.049    0.075    0.075
----TACACGCCATCATTCACATT---------------------------------    0.097    0.000    0.000

T                                                      A
 AAGTACACGCCATCATTCACATTCACCGCATGTCACCGCCGGAACCAACACGAC G
 TTCATGTGCGGTAGTAAGTGTAAGTGGCGTACAGTGGCGGCCTTGGTTGTGCTG T
C                                                      T

-------TGCGGTAGTAAGTGTAAGT-------------------------------    0.049    0.000    0.000
-----TGTGCGGTAGTAAGTGTAAGT-------------------------------    8.985    0.417    0.527
------GTGCGGTAGTAAGTGTAAGT-------------------------------    1.700    0.038    0.188
--------GCGGTAGTAAGTGTAAGT-------------------------------    0.097    0.000    0.000
----ATGTGCGGTAGTAAGTGTAAGT-------------------------------    0.146    0.000    0.000
--------GCGGTAGTAAGTGTAAGTGGC----------------------------    0.243    3.258    1.280
----------GGTAGTAAGTGTAAGTGGC----------------------------    0.049    0.038    0.038
----------GGTAGTAAGTGTAAGTGGCG---------------------------    0.534    0.796    0.565
-----------GTAGTAAGTGTAAGTGGCG---------------------------    0.194    0.493    0.904
-----------GTAGTAAGTGTAAGTGGCGT--------------------------   45.508  152.188  124.248
--------GCGGTAGTAAGTGTAAGTGGCGT--------------------------    0.049    0.000    0.000
---------CGGTAGTAAGTGTAAGTGGCGT--------------------------    0.194    0.341    0.075
----------GGTAGTAAGTGTAAGTGGCGT--------------------------  199.955  269.787  202.336 miR_t77
-------------AGTAAGTGTAAGTGGCGT--------------------------    0.049    0.076    0.076
--------------GTAAGTGTAAGTGGCGTA-------------------------    0.049    0.000    0.000
-----------GTAGTAAGTGTAAGTGGCGTAC------------------------    0.049    0.114    0.264
-------------AGTAAGTGTAAGTGGCGTAC------------------------    0.097    0.000    0.000
-------------AGTAAGTGTAAGTGGCGTACA-----------------------    0.049    0.227    0.227
---------------TAAGTGTAAGTGGCGTACAG----------------------    0.340    0.152    0.640
----------------AAGTGTAAGTGGCGTACAG----------------------    0.049    0.152    0.075
-------------------TGTAAGTGGCGTACAGT---------------------    0.146    0.000    0.000
---------------TAAGTGTAAGTGGCGTACAGT---------------------   40.457   14.435   22.402 miR_t58
------------TAGTAAGTGTAAGTGGCGTACAGT---------------------    0.340    0.114    0.114
----------------AAGTGTAAGTGGCGTACAGT---------------------    7.042    8.486   11.145
-------------AGTAAGTGTAAGTGGCGTACAGT---------------------    0.049    0.114    0.075
--------------------GTAAGTGGCGTACAGT---------------------    0.097    0.000    0.000
------------------AGTGTAAGTGGCGTACAGT---------------------    0.534    4.205    4.443
--------------GTAAGTGTAAGTGGCGTACAGT---------------------    0.971    0.379    0.452
--------------------------GGCGTACAGTGGCGGCCTT------------    0.049    0.075    0.075
---------------------------GCGTACAGTGGCGGCCTTGGT---------    0.146    0.189    0.264
--------------------------GGCGTACAGTGGCGGCCTTGGT---------    0.243    0.758    0.038
-----------------------------GTACAGTGGCGGCCTTGGTT--------    0.049    0.000    0.000
----------------------------CGTACAGTGGCGGCCTTGGTT--------    0.049    0.038    0.038
------------------------------TACAGTGGCGGCCTTGGTTGT------    0.097    0.000    0.000
-------------------------------ACAGTGGCGGCCTTGGTTGT------    0.340    0.114    0.226
---------------------------------AGTGGCGGCCTTGGTTGTGCT---    0.291    0.075    0.075
----------------------------------GTGGCGGCCTTGGTTGTGCT---    1.166    0.379    0.226
```

miRNA: miR_t21,miR_t80 chromosome_2 Start: 9129505 End: 9129606 Condition: TAP

```
------------------GAGGGCGCAAGCACGCAAG------------------    0.049    0.076    0.038
-----------------AGAGGGCGCAAGCACGCAAG------------------    0.437    0.114    0.264
-----------------AGAGGGCGCAAGCACGCA--------------------    0.291    0.152    1.355
----------------TAGAGGGCGCAAGCAC-----------------------    0.049    0.000    0.000
----------------TAGAGGGCGCAAGCACGCAAG------------------   31.424   17.693   25.527
----------------TAGAGGGCGCAAGCACGC---------------------    6.897   32.620   30.384
----------------TAGAGGGCGCAAGCACGCA--------------------   38.757   60.163  109.602 miR_t21
----------------TAGAGGGCGCAAGCACGCAAGG-----------------    0.583    0.341    0.452
----------------TAGAGGGCGCAAGCACGCAA-------------------    1.700    2.841    3.502
---------------CTAGAGGGCGCAAGCACGCA--------------------    0.097    0.038    0.113
--------------GCTAGAGGGCGCAAGCACGCA--------------------    0.049    0.000    0.000
------------CGGCTAGAGGGCGCAAGCACG----------------------    0.146    0.568    0.452 miR_t80*
------------CGGCTAGAGGGCGCAAGCACGC---------------------    0.049    2.008    0.904

--------G                                           AC
         AGACGGCTAGAGGGCGCAAGCACGCAAGGAGGGGATGACGAGC  A
         TCTGCCGATCTCCCGCGTTCGTGCGTTCCTCCCCTACTGCTCG  /
GACTGCCGA                                           TT

------CGATCTGCCGATCTCCCGCGT----------------------------    0.049    0.000    0.000
----GCCGATCTGCCGATCTCCCGCGT----------------------------    0.049    0.038    0.038
------------GCCGATCTCCCGCGTTCG-------------------------    0.049    0.000    0.000
----------CTGCCGATCTCCCGCGTTCG-------------------------    6.071    3.031    4.593
----------CTGCCGATCTCCCGCGTTCGT------------------------  434.928  229.211  264.610 miR_t80
---------TCTGCCGATCTCCCGCGTTCGT------------------------    1.068    0.189    0.188
```

```
-------------CCGATCTCCCGCGTTCGT-------------------------      0.194      0.189      0.038
--------------CGATCTCCCGCGTTCGT-------------------------      0.291      0.075      0.075
-----------TGCCGATCTCCCGCGTTCGT-------------------------      2.234      0.227      0.339
---------------GATCTCCCGCGTTCGT-------------------------      0.049      0.000      0.000
------------GCCGATCTCCCGCGTTCGT-------------------------     11.414      2.955      1.958
------------GCCGATCTCCCGCGTTCGTG------------------------      0.631      0.038      0.038
------------GCCGATCTCCCGCGTTCGTGC-----------------------      4.420      1.212      0.301
--------------CGATCTCCCGCGTTCGTGCG----------------------      0.097      0.076      0.076
--------------CGATCTCCCGCGTTCGTGCGT---------------------      2.186      0.985      1.205
--------------CGATCTCCCGCGTTCGTGCGTT--------------------      0.583      0.493      0.151
---------------GATCTCCCGCGTTCGTGCGTT--------------------      0.146      0.076      0.038
-----------------------GCGTTCGTGCGTTCCTCCCCT------------      0.146      0.152      0.152
------------------------CGTTCGTGCGTTCCTCCCCT------------      0.194      0.151      0.151
----------------------------TCGTGCGTTCCTCCCCTACT--------      0.194      0.038      0.038
```

miRNA: miR_t82 chromosome_11 Start: 1713197 End: 1713520 Condition: TAP

```
-----------------------------------------------------------------------------------------------------------------------------------------------TTTCCAGGTGCTCCAGAAC-GG------------      0.049      0.038      0.038
----------------------------------------------------------------------------------------------------------------------------------------------TTTTCCAGGTGCTCCAGAAC-GGC-----------      0.049      0.038      0.038
------------------------------------------------------------------------------------------------------------------------------------------TCGATTTTCCAGGTGCTCCAG------------------      0.049      0.075      0.075
------------------------------------------------------------------------------------------------------------------------------------------TCGATTTTCCAGGTGCTCCAGA-----------------      0.097      0.000      0.000
------------------------------------------------------------------------------------------------------------------------------------------TCGATTTTCCAGGTGCTCC--------------------      0.097      0.038      0.038
-----------------------------------------------------------------------------------------------------------------------TATTGCAGAGCCAGCGGCCTCG------------------------------------      0.049      0.000      0.000
--------------------------------------------------------------------------------------------------------------------TGTTATTGCAGAGCCAGCGG-----------------------------------------      0.049      0.114      0.113
-------------------------------------------------------------------------------------------------------------ACTACACTGTTATTGCAGAGC-----------------------------------------------      0.049      0.000      0.000
----------------------------------------------------------------------------------------------------------AGGACTACACTGTTATTGCAG--------------------------------------------------      0.049      0.038      0.038
---------------------------------------------------------------------------------------------------------AAGGACTACACTGTTATTGCA---------------------------------------------------      0.097      0.000      0.000
--------------------------------------------------------------------------------------------------GGGAACAAGGACTACACTG------------------------------------------------------------      0.146      0.038      0.038
-------------------------------------------------------------------------------------------------CGGGAACAAGGACTACACTGT-----------------------------------------------------------      0.049      0.000      0.000
-------------------------------------------------------------------------------------------------CGGGAACAAGGACTACACTG------------------------------------------------------------      0.146      0.075      0.075
------------------------------------------------------------------------------------------------TCGGGAACAAGGACTACA---------------------------------------------------------------      0.049      0.000      0.000
------------------------------------------------------------------------------------------------TCGGGAACAAGGACTACACTGT-----------------------------------------------------------     10.005      6.251      2.033
------------------------------------------------------------------------------------------------TCGGGAACAAGGACTACACTG------------------------------------------------------------     31.521     23.489     18.825
------------------------------------------------------------------------------------------------TCGGGAACAAGGACTACACT-------------------------------------------------------------      0.631      0.152      0.678
------------------------------------------------------------------------------------------------TCGGGAACAAGGACTACAC--------------------------------------------------------------      0.049      0.113      0.113
--------------------------------------------------------------------------------------TCACGCTTGGTCGGGAACAAGG---------------------------------------------------------------------      0.680      0.152      0.565
--------------------------------------------------------------------------------------TCACGCTTGGTCGGGAACAAG----------------------------------------------------------------------      0.097      0.000      0.000
--------------------------------------------------------------------------------AGCGGCTCACGCTTGGTCGGG----------------------------------------------------------------------------      0.049      0.076      0.076
-------------------------------------------------------------------------GGACTGGAGCGGCTCACGCT------------------------------------------------------------------------------------      0.146      0.264      0.264
------------------------------------------------------------------------TGGACTGGAGCGGCTCACG--------------------------------------------------------------------------------------      0.097      0.075      0.075
------------------------------------------------------------------------TGGACTGGAGCGGCTCACGC-------------------------------------------------------------------------------------      0.243      0.417      0.828
------------------------------------------------------------------------TGGACTGGAGCGGCTCAC---------------------------------------------------------------------------------------      0.049      0.188      0.188
------------------------------------------------------------------------TGGACTGGAGCGGCTCACGCT------------------------------------------------------------------------------------      8.888      2.008      8.133
----------------------------------------------------------------CACACCGGTGGACTGGAGCGG--------------------------------------------------------------------------------------------      0.049      0.000      0.000
-------------------------------------------------------------CGGCACACCGGTGGACTGGAG-----------------------------------------------------------------------------------------------      0.049      0.076      0.076
---------------------------------------------------TTCGGCAGGACGGCACACCGG---------------------------------------------------------------------------------------------------------      0.097      0.075      0.075
-------------------------------------------TACGGAGGTTCGGCAGGACGGC----------------------------------------------------------------------------------------------------------------      0.097      0.114      0.113
-------------------------------------------TACGGAGGTTCGGCAGGACG------------------------------------------------------------------------------------------------------------------      0.049      0.076      0.038
-------------------------------------------TACGGAGGTTCGGCAGGACGG-----------------------------------------------------------------------------------------------------------------      0.146      0.114      0.791
------------------------------------------TTACGGAGGTTCGGCAGGACG------------------------------------------------------------------------------------------------------------------      0.243      0.265      0.489
---------------------------------------TAATTACGGAGGTTCGGCAGG---------------------------------------------------------------------------------------------------------------------      1.360      1.061      2.861
------------------------------------TGGTAATTACGGAGGTTCGG-------------------------------------------------------------------------------------------------------------------------      0.243      0.265      0.113
------------------------------------TGGTAATTACGGAGGTTCGGC------------------------------------------------------------------------------------------------------------------------      1.846      2.766      5.196
-----------------------------------ATGGTAATTACGGAGGTTCGG-------------------------------------------------------------------------------------------------------------------------      0.146      0.000      0.000
------------------------------TCACGATGGTAATTACGGAGG------------------------------------------------------------------------------------------------------------------------------      0.146      0.000      0.000

-------------------------                 T                                                                 C                                                 AC-    AAAG   AG
                         GGCATCACGATGGTAAT ACGGAGGTTCGGCAGGACGGCACACCGGTGGACTGGAGCGGCTCACGCTTGGTCGGGAACAAGGA TACACTGTTATTGCAGAGCCAGCGGCCTCGATTTTCCAGGTGCTCCAGA    GGCA    TGA  C
                         CCGTAGTGCTACCATTA TGCCTCCAAGCCGTCCTGCCGTGTGGCCACCTGACCTCGCCGAGTGCGAACCAGCCCTTGTTCCT ATGTGACAATAACGTCTCGGTCGCCGGAGCTAAAAGGTCCACGAGGTCT    CTGT    GCT  A
CCACCGAAGTCGAACGTTGTCGCGT                 T                                                                 A                                                 GAA    CAA-   AA

-----GAAGTCGAACGTTGTCGCGT--------------------------------------------------------------------------------------------------------------------------------------------------------      0.049      0.000      0.000
----CGAAGTCGAACGTTGTCGCGT--------------------------------------------------------------------------------------------------------------------------------------------------------      0.049      0.493      0.753
-----------GAACGTTGTCGCGTCCGTAGT-------------------------------------------------------------------------------------------------------------------------------------------------      0.049      0.000      0.000
--------------CGTTGTCGCGTCCGTAGTGCT----------------------------------------------------------------------------------------------------------------------------------------------      0.340      0.530      0.527
---------------------GCGTCCGTAGTGCTACCA------------------------------------------------------------------------------------------------------------------------------------------      0.049      0.000      0.000
--------------------CGCGTCCGTAGTGCTACCA------------------------------------------------------------------------------------------------------------------------------------------      0.049      0.075      0.075
------------------GTCGCGTCCGTAGTGCTACCAT-----------------------------------------------------------------------------------------------------------------------------------------      0.049      0.000      0.000
-------------------TCGCGTCCGTAGTGCTACCAT-----------------------------------------------------------------------------------------------------------------------------------------      0.291      0.264      0.264
---------------------GCGTCCGTAGTGCTACCATT----------------------------------------------------------------------------------------------------------------------------------------      0.097      0.189      0.979
-------------------TCGCGTCCGTAGTGCTACCATT----------------------------------------------------------------------------------------------------------------------------------------      0.049      0.000      0.000
--------------------CGCGTCCGTAGTGCTACCATT----------------------------------------------------------------------------------------------------------------------------------------      2.234      4.395     11.709
---------------------GCGTCCGTAGTGCTACCATTA---------------------------------------------------------------------------------------------------------------------------------------      0.291      2.197      4.518
----------------------CGTCCGTAGTGCTACCATTA---------------------------------------------------------------------------------------------------------------------------------------      0.049      0.076      0.188
--------------------CGCGTCCGTAGTGCTACCATTAT--------------------------------------------------------------------------------------------------------------------------------------      0.049      0.000      0.000
----------------------CGTCCGTAGTGCTACCATTAT--------------------------------------------------------------------------------------------------------------------------------------      4.323      9.509     35.957
-----------------------GTCCGTAGTGCTACCATTAT--------------------------------------------------------------------------------------------------------------------------------------      0.049      0.000      0.000
-----------------------GTCCGTAGTGCTACCATTATT-------------------------------------------------------------------------------------------------------------------------------------      0.389      0.265      0.301
----------------------CGTCCGTAGTGCTACCATTATT-------------------------------------------------------------------------------------------------------------------------------------      0.049      0.152      0.226
---------------------------------------------GCCTCCAAGCCGTCCTGCCGT---------------------------------------------------------------------------------------------------------------      0.291      0.152      0.226
----------------------------------------------CCTCCAAGCCGTCCTGCCGT---------------------------------------------------------------------------------------------------------------      0.146      0.038      0.038
----------------------------------------------CCTCCAAGCCGTCCTGCCGTGT-------------------------------------------------------------------------------------------------------------      0.146      0.000      0.000
-----------------------------------------------CTCCAAGCCGTCCTGCCGTGT-------------------------------------------------------------------------------------------------------------      0.049      0.000      0.000
-------------------------------------------------------CGTCCTGCCGTGTGGCCACCT-----------------------------------------------------------------------------------------------------      0.049      0.188      0.188
------------------------------------------------------------TGCCGTGTGGCCACCTGACCT------------------------------------------------------------------------------------------------      0.049      0.000      0.000
--------------------------------------------------------------------GGCCACCTGACCTCGCCGAGT----------------------------------------------------------------------------------------      2.331      0.000      0.000
-------------------------------------------------------------------TGGCCACCTGACCTCGCCGAGT----------------------------------------------------------------------------------------      0.097      0.000      0.000
---------------------------------------------------------------------------------CGCCGAGTGCGAACCAGCCCT---------------------------------------------------------------------------      1.506      0.152      0.339
----------------------------------------------------------------------------------GCCGAGTGCGAACCAGCCCTT--------------------------------------------------------------------------      0.097      0.000      0.000
---------------------------------------------------------------------------------CGCCGAGTGCGAACCAGCCCTT--------------------------------------------------------------------------      0.049      0.000      0.000
-------------------------------------------------------------------------------------GAGTGCGAACCAGCCCTTG-------------------------------------------------------------------------      0.049      0.038      0.038
-------------------------------------------------------------------------------------GAGTGCGAACCAGCCCTTGT------------------------------------------------------------------------      0.146      0.301      0.301
------------------------------------------------------------------------------------CGAGTGCGAACCAGCCCTTGT------------------------------------------------------------------------      0.097      0.075      0.075
--------------------------------------------------------------------------------------AGTGCGAACCAGCCCTTGTT-----------------------------------------------------------------------      0.486      0.379      0.414
-------------------------------------------------------------------------------------GAGTGCGAACCAGCCCTTGTT-----------------------------------------------------------------------     12.968      4.812      8.208
------------------------------------------------------------------------------------CGAGTGCGAACCAGCCCTTGTT-----------------------------------------------------------------------      0.971      4.774      7.794
----------------------------------------------------------------------------------------TGCGAACCAGCCCTTGTTCCT--------------------------------------------------------------------      0.049      0.000      0.000
-------------------------------------------------------------------------------------------GAACCAGCCCTTGTTCCTAAT-----------------------------------------------------------------      0.194      0.075      0.075
-----------------------------------------------------------------------------------------------------------CCTAATGTGACAATAACGTCT-------------------------------------------------      0.340      0.000      0.000
-------------------------------------------------------------------------------------------------------------------------AACGTCTCGGTCGCCGGAGCT-----------------------------------      0.049      0.000      0.000
--------------------------------------------------------------------------------------------------------------------------------CGGTCGCCGGAGCTAAAA-------------------------------      0.049      0.000      0.000
--------------------------------------------------------------------------------------------------------------------------------CGGTCGCCGGAGCTAAAAGG-----------------------------      4.371      3.069     30.347
---------------------------------------------------------------------------------------------------------------------------------GGTCGCCGGAGCTAAAAGG-----------------------------      0.097      0.038      0.038
----------------------------------------------------------------------------------------------------------------------------------GTCGCCGGAGCTAAAAGGT----------------------------      0.291      0.417      0.979
--------------------------------------------------------------------------------------------------------------------------------CGGTCGCCGGAGCTAAAAGGT----------------------------    316.713    556.395   2457.318 miR_t82
---------------------------------------------------------------------------------------------------------------------------------GGTCGCCGGAGCTAAAAGGT----------------------------      6.945      2.197      5.271
-------------------------------------------------------------------------------------------------------------------------------TCGGTCGCCGGAGCTAAAAGGT----------------------------      0.146      0.152      0.151
--------------------------------------------------------------------------------------------------------------------------------CGGTCGCCGGAGCTAAAAGGTC---------------------------      0.631      1.250      2.786
-----------------------------------------------------------------------------------------------------------------------------------------GAGCTAAAAGGTCCACGAGGT-------------------      0.243      0.000      0.000
--------------------------------------------------------------------------------------------------------------------------------------------------------CGAGGTCTGAACTGTCAA-------      0.097      0.000      0.000
```

miRNA: miR_t87 chromosome_11 Start: 2856059 End: 2856444 Condition: TAP

```
-----------TGCCTCTGTTACTTACTGC--------------------------------------------------      0.049      0.000      0.000
-----------TGCCTCTGTTACTTACTGCTG------------------------------------------------      1.797      0.227      1.205
-----------TGCCTCTGTTACTTACTGCTGG-----------------------------------------------      0.049      0.000      0.000
---------------TCTGTTACTTACTGCTGGCCTC-------------------------------------------      0.291      0.038      0.151
---------------TCTGTTACTTACTGCTGGC----------------------------------------------      1.117      0.038      0.038
---------------TCTGTTACTTACTGCTGGCC---------------------------------------------      0.049      0.000      0.000
---------------TCTGTTACTTACTGCTGGCCT--------------------------------------------      0.194      0.000      0.000
---------------TCTGTTACTTACTGCTGGCCTCT------------------------------------------      0.097      0.113      0.113
-----------------TGTTACTTACTGCTGGCCTCT------------------------------------------      0.194      0.038      0.038
-----------------TGTTACTTACTGCTGGCCTC-------------------------------------------      0.049      0.000      0.000
-------------------TTACTTACTGCTGGCCTCTC-----------------------------------------      0.049      0.000      0.000
-------------------TTACTTACTGCTGGCCTCTCG----------------------------------------      0.049      0.000      0.000
--------------------TACTTACTGCTGGCCTCTCG----------------------------------------      1.360      0.493      0.414
--------------------TACTTACTGCTGGCCTCTCGT---------------------------------------      0.097      0.038      0.113
-----------------------TTACTGCTGGCCTCTCGTTCA------------------------------------      0.146      0.000      0.000
------------------------TACTGCTGGCCTCTCGTTCAG-----------------------------------      0.243      0.038      0.038
------------------------------TGGCCTCTCGTTCAGTCTCC------------------------------      0.049      0.076      0.038
------------------------------TGGCCTCTCGTTCAGTCTCCA-----------------------------      0.097      0.000      0.000
--------------------------------------TCGTTCAGTCTCCATCCATCG---------------------      0.583      0.189      0.226
-----------------------------------------TTCAGTCTCCATCCATCGTGC------------------      0.971      0.189      0.151
------------------------------------------------TCCATCCATCGTGCGCA-TCGTCT--------      0.049      0.000      0.000

-                                        T                     A-    CTCCA-   T
 CGGCACGCCCTGCCTCTGTTACTTACTGCTGGCCTCTCGT CAGTCTCCATCCATCGTGCGC  TCGT      AG T
 GCTGTGCGGGACGGAGACAATGAATGACGACCGGAGAGCA GTCAGAGGTAGGTAGCACGCG  GGCA      TC A
T                                        T                     CG    CACAAA   A

---------------------------------------GCATGTCAGAGGTAGGTAGCA--------------------      0.194      0.152      0.152
---------------------------------CGGAGAGCATGTCAGAGGTAGGT------------------------      0.097      0.227      0.227
-----------------------------------GAGAGCATGTCAGAGGTAGGT------------------------      0.243      0.038      0.038
----------------------------------GGAGAGCATGTCAGAGGT----------------------------      0.049      0.114      0.114
---------------------------------CGGAGAGCATGTCAGAGGT----------------------------      6.217      7.577     12.952
--------------------------------CCGGAGAGCATGTCAGAGGT----------------------------      3.205      3.410      5.459
------------------------------GACCGGAGAGCATGTCAGAGGT----------------------------      0.097      0.038      0.113
-------------------------------ACCGGAGAGCATGTCAGAGGT----------------------------     60.079     44.971     81.326 miR_t87
--------------------------------CCGGAGAGCATGTCAGAGG-----------------------------      0.049      0.075      0.075
-------------------------------ACCGGAGAGCATGTCAGAGG-----------------------------      0.631      0.303      1.092
-----------------------GAATGACGACCGGAGAGCATGT-----------------------------------      0.437      0.076      0.075
------GCGGGACGGAGACAATGAATG-----------------------------------------------------      0.049      0.000      0.000
----GTGCGGGACGGAGACAATGAAT------------------------------------------------------      0.049      0.038      0.038
-----TGCGGGACGGAGACAATGAAT------------------------------------------------------      0.097      0.038      0.226
```

miRNA: miR_t91 chromosome_9 Start: 6365819 End: 6366048 Condition: TAP

```
------------------------------------------------------------------------------------TTCTAGTGCCTGGCTGGGATC---------------------------------------------------      0.631      3.372      1.544
-------------------------------------------------------------------------------------TCTAGTGCCTGGCTGGGATC---------------------------------------------------      0.049      0.038      0.038
---------------------------------------------------------------------------------------TAGTGCCTGGCTGGGATCAATGC----------------------------------------------      0.291      1.250      0.565
---------------------------------------------------------------------------------------TAGTGCCTGGCTGGGATCAA-------------------------------------------------      0.146      1.743      1.732
---------------------------------------------------------------------------------------TAGTGCCTGGCTGGGATCAAT------------------------------------------------      1.506      3.296      4.556
---------------------------------------------------------------------------------------TAGTGCCTGGCTGGGATCAATG-----------------------------------------------      0.049      0.038      0.038
---------------------------------------------------------------------------------------TAGTGCCTGGCTGGGATC---------------------------------------------------      0.049      0.833      0.264
---------------------------------------------------------------------------------------TAGTGCCTGGCTGGG------------------------------------------------------      0.049      0.000      0.000
------------------------------------------------------------------------------------------TGCCTGGCTGGGATCAATGCAAG-------------------------------------------      0.243      1.364      0.038
--------------------------------------------------------------------------------------------CCTGGCTGGGATCAATGCAAG-------------------------------------------      0.049      0.455      0.151
--------------------------------------------------------------------------------------------CCTGGCTGGGATCAATGCAAGA------------------------------------------      0.097      0.000      0.000
----------------------------------------------------------------------------------------------TGGCTGGGATCAATGCAAGATGAT--------------------------------------      1.166      1.402      1.402
```

```
---------------------------------------------------------------------------------------------TGGCTGGGATCAATGCAAGAT----------------------------------------      0.243      0.303      0.527
---------------------------------------------------------------------------------------------TGGCTGGGATCAATGCAAGA-----------------------------------------      0.340      1.440      1.581
---------------------------------------------------------------------------------------------TGGCTGGGATCAATGCAAGATGA--------------------------------------      0.534      1.932      0.113
---------------------------------------------------------------------------------------------TGGCTGGGATCAATGCAAG------------------------------------------      0.243      1.061      0.828
---------------------------------------------------------------------------------------------TGGCTGGGATCAATGCAAGATG---------------------------------------      1.846      7.388      2.146
----------------------------------------------------------------------------------------------GGCTGGGATCAATGCAAGATG---------------------------------------      0.049      0.038      0.038
-------------------------------------------------------------------------------------------------TGGGATCAATGCAAGA-----------------------------------------      0.049      0.000      0.000
-------------------------------------------------------------------------------------------------TGGGATCAATGCAAGATGATA------------------------------------      3.205      3.107      1.468
-------------------------------------------------------------------------------------------------TGGGATCAATGCAAGATGAT-------------------------------------      0.971      0.189      0.489
-------------------------------------------------------------------------------------------------TGGGATCAATGCAAGATGATAG-----------------------------------      0.049      0.038      0.038
------------------------------------------------------------------------------------------------------TCAATGCAAGATGATAGTTGG-------------------------------      0.049      0.000      0.000
--------------------------------------------------------------------------------------------------------AATGCAAGATGATAGTTGGATC----------------------------      0.049      0.152      0.152
----------------------------------------------------------------------------------------------------------TGCAAGATGATAGTTGGATCC---------------------------     91.940     66.717     45.746
----------------------------------------------------------------------------------------------------------TGCAAGATGATAGTTGGATC----------------------------      0.680      4.319      2.749
----------------------------------------------------------------------------------------------------------TGCAAGATGATAGTTGGATCCT--------------------------      0.437      0.947      0.979
-----------------------------------------------------------------------------------------------------------GCAAGATGATAGTTGGATCC---------------------------      0.097      0.265      0.038
----------------------------------------------------------------------------------------------------------------ATGATAGTTGGATCCTGGA-----------------------      0.049      0.000      0.000
----------------------------------------------------------------------------------------------------------------ATGATAGTTGGATCCTGGACGC--------------------      0.049      2.311      1.167
----------------------------------------------------------------------------------------------------------------ATGATAGTTGGATCCTGGACG---------------------     85.431     74.219     14.609
-----------------------------------------------------------------------------------------------------------------TGATAGTTGGATCCTGGACG---------------------    151.193     39.970     26.732
-----------------------------------------------------------------------------------------------------------------TGATAGTTGGATCCTGGACGC--------------------      1.554      9.964      5.610
-----------------------------------------------------------------------------------------------------------------TGATAGTTGGATCCTGG------------------------      0.049      0.000      0.000
-----------------------------------------------------------------------------------------------------------------TGATAGTTGGATCCTGGA-----------------------      0.049      0.000      0.000
-----------------------------------------------------------------------------------------------------------------TGATAGTTGGATCCTGGAC----------------------      0.049      0.114      0.114
-----------------------------------------------------------------------------------------------------------------TGATAGTTGGATCCTGGACGCC-------------------      0.243      1.137      1.619
------------------------------------------------------------------------------------------------------------------GATAGTTGGATCCTGGACG---------------------      2.817      0.758      0.301
-------------------------------------------------------------------------------------------------------------------ATAGTTGGATCCTGGACGCCG------------------      0.097      0.303      0.038
-------------------------------------------------------------------------------------------------------------------ATAGTTGGATCCTGGACG---------------------      0.049      0.076      0.076
--------------------------------------------------------------------------------------------------------------------TAGTTGGATCCTGGACG---------------------     19.379      4.357      3.803
--------------------------------------------------------------------------------------------------------------------TAGTTGGATCCTGGAC----------------------      0.049      0.000      0.000
--------------------------------------------------------------------------------------------------------------------TAGTTGGATCCTGGACGCC-------------------      0.097      0.758      0.753
--------------------------------------------------------------------------------------------------------------------TAGTTGGATCCTGGACGCCG------------------      0.049      0.114      0.188
--------------------------------------------------------------------------------------------------------------------TAGTTGGATCCTGGACGC--------------------      0.146      0.114      0.114
---------------------------------------------------------------------------------------------------------------------AGTTGGATCCTGGACG---------------------      0.049      0.000      0.000
-----------------------------------------------------------------------------------------------------------------------TTGGATCCTGGACGCCGAAGGC-------------      0.340      2.425      0.226
------------------------------------------------------------------------------------------------------------------------TGGATCCTGGACGCCGAAGGC-------------      4.274     16.026      9.337
------------------------------------------------------------------------------------------------------------------------TGGATCCTGGACGCCGAAGGCC------------      0.146      0.303      0.303
-------------------------------------------------------------------------------------------------------------------------GGATCCTGGACGCCGAAGGC-------------      0.049      0.189      0.038
-------------------------------------------------------------------------------------------------------------------------------TGGACGCCGAAGGCCATC---------      0.049      0.038      0.038
-------------------------------------------------------------------------------------------------------------------------------TGGACGCCGAAGGCCATCATG------      4.128      0.530      0.301
-------------------------------------------------------------------------------------------------------------------------------TGGACGCCGAAGGCCATCATGCG----      0.049      0.000      0.000
-------------------------------------------------------------------------------------------------------------------------------TGGACGCCGAAGGCCATCATGC-----      0.291      0.000      0.000

-------------------------------------------------------------------------------       T                    G                             A    A         G
                                                                               CATTTTC AGTGCCTGGCTGGGATCAAT CAAGATGATAGTTGGATCCTGGACGCCGA GGCC TCATGCGGT T
                                                                               GTAGAGG TCGCGGACCGACCCTAGTTA GTTCTGCTGTCGATCTAGGACCTGCGGTT CCGG AGTACGCCA A
GGGACCTCGGTTGCTGCCACCACGGTAGCAGCGGCCAGACGAGCCTTGGGTCCGTTGTTCCCTCCCTTCTGGCACCCAG       T                    G                             G    C         A

-----------------------------------------------------------------------------------------------------------------------------GGACCTGCGGTTGCCGGCAGT--------      0.971      0.455      0.075
--------------------------------------------------------------------------------------------------------------------GTCGATCTAGGACCTGCGGTT-----------------      0.049      0.038      0.038
-------------------------------------------------------------------------------------------------------------TTCTGCTGTCGATCTAGGACCT-----------------------      1.311      0.152      0.226
------------------------------------------------------------------------------------------------------------------CTGTCGATCTAGGACCT-----------------------      0.097      0.227      0.226
------------------------------------------------------------------------------------------------------------GTTCTGCTGTCGATCTAGGACCT-----------------------      0.049      0.000      0.000
----------------------------------------------------------------------------------------------------------------TGCTGTCGATCTAGGACCT-----------------------      0.097      0.152      0.152
--------------------------------------------------------------------------------------------------------------TCTGCTGTCGATCTAGGACCT-----------------------    226.133     90.964     60.543
---------------------------------------------------------------------------------------------------------------CTGCTGTCGATCTAGGACCT-----------------------     10.199     10.040      4.744
--------------------------------------------------------------------------------------------------------------------GTCGATCTAGGACCT-----------------------      0.049      0.000      0.000
-----------------------------------------------------------------------------------------------------------------GCTGTCGATCTAGGACCT-----------------------      0.194      0.000      0.000
--------------------------------------------------------------------------------------------------------------TCTGCTGTCGATCTAGGAC-------------------------      0.874      0.493      0.452
--------------------------------------------------------------------------------------------------------------TCTGCTGTCGATCTAGGA--------------------------      0.049      0.000      0.000
--------------------------------------------------------------------------------------------------------------TCTGCTGTCGATCTAGG---------------------------      0.049      0.000      0.000
------------------------------------------------------------------------------------------------------AGTTAGGTTCTGCTGTCGATCT------------------------------      0.340      0.114      0.113
-------------------------------------------------------------------------------------------------ACCCTAGTTAGGTTCTGCTGT------------------------------------      0.049      0.000      0.000
-----------------------------------------------------------------------------------------------CGACCCTAGTTAGGTTCTGCT--------------------------------------      9.471      5.115      2.749
-----------------------------------------------------------------------------------------------CGACCCTAGTTAGGTTCTGC---------------------------------------      0.049      0.000      0.000
--------------------------------------------------------------------------------------------GACCGACCCTAGTTAGGTTCT-----------------------------------------      0.049      0.000      0.000
-------------------------------------------------------------------------------------------GGACCGACCCTAGTTAGGTTCT-----------------------------------------      2.477      1.819      1.995
--------------------------------------------------------------------------------------------GACCGACCCTAGTTAGGTTC------------------------------------------      0.049      0.000      0.000
-------------------------------------------------------------------------------------------GGACCGACCCTAGTTAGGTTC------------------------------------------      0.097      0.076      0.076
------------------------------------------------------------------------------------------CGGACCGACCCTAGTTAGGTT-------------------------------------------      0.097      0.379      0.414
-------------------------------------------------------------------------------------------GGACCGACCCTAGTTAGGTT-------------------------------------------      0.486      2.804      2.447
-----------------------------------------------------------------------------------------GCGGACCGACCCTAGTTAGGTT-------------------------------------------      0.097      0.417      0.417
-----------------------------------------------------------------------------------------------CGACCCTAGTTAGGT--------------------------------------------      0.389      0.000      0.000
------------------------------------------------------------------------------------GGTTCGCGGACCGACCCTAGTTAGGT--------------------------------------------      0.437     11.820      0.715
-----------------------------------------------------------------------------------------GCGGACCGACCCTAGTTAGGT--------------------------------------------   3912.312   6200.058   8357.126 miR_t91
------------------------------------------------------------------------------------------------GACCCTAGTTAGGT--------------------------------------------      0.049      0.000      0.000
------------------------------------------------------------------------------------------CGGACCGACCCTAGTTAGGT--------------------------------------------      0.534      7.160      5.986
--------------------------------------------------------------------------------------------GACCGACCCTAGTTAGGT--------------------------------------------      0.340      0.000      0.000
-------------------------------------------------------------------------------------------GGACCGACCCTAGTTAGGT--------------------------------------------      2.331      6.327      7.380
----------------------------------------------------------------------------------------CGCGGACCGACCCTAGTTAGGT--------------------------------------------      0.486      7.842      3.991
-------------------------------------------------------------------------------------------GGACCGACCCTAGTTAGG---------------------------------------------      0.049      0.265      0.226
-----------------------------------------------------------------------------------------GCGGACCGACCCTAGTTAGG---------------------------------------------     52.065     65.164    172.704
-------------------------------------------------------------------------------------GTTCGCGGACCGACCCTAGTT------------------------------------------------      0.049      0.000      0.000
------------------------------------------------------------CCTCCCTTCTGGCACCCAGGT-------------------------------------------------------------------------      0.049      0.000      0.000
-----------------------------------------------------------CCCTCCCTTCTGGCACCCAGGT-------------------------------------------------------------------------      0.049      0.038      0.038
------------------------------------------------GGTCCGTTGTTCCCTCCCTT--------------------------------------------------------------------------------------      0.049      0.000      0.000
-----------------------------------------------GGGTCCGTTGTTCCCTCCCTT--------------------------------------------------------------------------------------      0.049      0.000      0.000
----CCTCGGTTGCTGCCACCACGGT--------------------------------------------------------------------------------------------------------------------------------      1.894      0.114      0.114
----CCTCGGTTGCTGCCACCACG----------------------------------------------------------------------------------------------------------------------------------      0.097      0.000      0.000

miRNA: miR_t94 chromosome_2 Start: 739300 End: 739394 Condition: TAP
---------AATGGCCGTACTACTATTGTC--------------------      0.049      0.417      0.038
----------ATGGCCGTACTACTATTGTC--------------------      0.049      0.114      0.114
-----------TGGCCGTACTACTATT-----------------------      0.049      0.000      0.000
-----------TGGCCGTACTACTATTGTCAT------------------      0.340      0.227      0.227
-----------TGGCCGTACTACTATTGT---------------------      2.963      4.130      1.581
-----------TGGCCGTACTACTATTGTCATG-----------------      0.194      0.076      0.038
-----------TGGCCGTACTACTATTGTC--------------------    266.834    694.869    427.826 miR_t94
-----------TGGCCGTACTACTATTG----------------------      1.214      0.152      0.075
-----------TGGCCGTACTACTATTGTCA-------------------     22.487      2.463      1.544
-----------TGGCCGTACTACTATTGTCATGT----------------      0.049      0.000      0.000
------------GGCCGTACTACTATTGT---------------------      0.049      0.113      0.113
------------GGCCGTACTACTATTGTC--------------------      6.994     13.450     16.604
------------GGCCGTACTACTATTGTCA-------------------      1.117      0.000      0.000
--------------CCGTACTACTATTGTC--------------------      0.243      0.000      0.000
----------------------------TCATGTCTGACTGCATCATGT-      0.049      0.000      0.000
-------------------------------TGTCTGACTGCATCATGT-      0.049      0.076      0.076

----- GG                                        T
     G  AAATGGCCGTACTACTATTGTCATGTCTGACTGCATCATG T
     C  TTTACCGGCATGATGGTAACAGTACAGACTGACGTAGTAC /
AACTT TA                                        -

----------------------GGTAACAGTACAGACTGACGT-------      0.534      0.720      0.188
-------------------GATGGTAACAGTACAGACT------------      0.049      0.189      0.189
--------TTTACCGGCATGATGGTAACAGT-------------------      0.049      0.000      0.000
---------TTACCGGCATGATGGTAACAGT-------------------      0.146      0.038      0.038
-----------ACCGGCATGATGGTAACAGT-------------------      0.049      0.000      0.000
----TCTATTTACCGGCATGAT----------------------------      0.049      0.000      0.000

miRNA: miR_t97,miR_t26 chromosome_8 Start: 121853 End: 121958 Condition: TAP
------------------------TCGGTCCTAGGCGGCGCGTG------------      0.146      0.076      0.038
------------------------TCGGTCCTAGGCGGCGCGTGT-----------      0.049      0.038      0.038
-------------------TTTCGTCGGTCCTAGGCGGC-----------------      0.340      1.894      0.828
-------------------TTTCGTCGGTCCTAGG---------------------      0.049      0.000      0.000
----------------GGCTTTCGTCGGTCCTAGGC--------------------      0.049      0.038      0.038
----------------GGCTTTCGTCGGTCCTAG----------------------      0.049      0.000      0.000
----------------GGCTTTCGTCGGTCCTAGG---------------------      6.168      0.985      0.715
---------------TGGCTTTCGTCGGTCCTAGG---------------------   1283.074    119.947     89.948 miR_t97
---------------TGGCTTTCGTCGGTCCTAG----------------------     10.734      2.955      2.937
---------------TGGCTTTCGTCGGTCCTAGGC--------------------     19.282     15.533      4.405
-------------AGTGGCTTTCGTCGGTCCTAG----------------------      0.146      0.152      0.152
------------TAGTGGCTTTCGTCGGTCCTAGG---------------------      0.729      0.038      0.226
------------TAGTGGCTTTCGTCGGTCCTA-----------------------      0.146      0.076      0.076
------------TAGTGGCTTTCGTCGGT---------------------------      0.049      0.000      0.000
------------TAGTGGCTTTCGTCGGTCCTAG----------------------      0.049      0.038      0.038
-----------TTAGTGGCTTTCGTCGGTCCTAG----------------------      0.291      0.038      0.038
-----------TTAGTGGCTTTCGTCGGT---------------------------      0.049      0.038      0.038
-----------TTAGTGGCTTTCGTCGGTCCTAGG---------------------      0.194      0.000      0.000
-----------TTAGTGGCTTTCGTCGGTCCTA-----------------------      2.234      1.326      0.113
-----------TTAGTGGCTTTCGTCGGTCCT------------------------      0.631      1.212      0.038

-------                                             GAC
       CAGCTTAGTGGCTTTCGTCGGTCCTAGGCGGCGCGTGTGCGCGCG   G
       GTCGAATCACCGAAAGCAGCCAGGATCCGCCGCGCACACGCGCGC   A
AGCACTG                                             AAA

-------------TCACCGAAAGCAGCCAGG-------------------------      0.291      0.038      0.113
------------ATCACCGAAAGCAGCCAGG-------------------------      0.194      0.076      0.076
--------------CACCGAAAGCAGCCAGGA------------------------      0.049      0.000      0.000
-----------AATCACCGAAAGCAGCCAGGA------------------------      0.097      0.076      0.076
-----------------CGAAAGCAGCCAGGA------------------------      0.097      0.000      0.000
-------------TCACCGAAAGCAGCCAGGA------------------------      5.780      1.250      1.017
------------ATCACCGAAAGCAGCCAGGA------------------------      1.554      0.606      0.606
------GGTCGAATCACCGAAAGCAGCCAGGAT-----------------------      0.146      0.417      0.417
------------ATCACCGAAAGCAGCCAGGAT-----------------------     73.484     30.801     12.274
-------------TCACCGAAAGCAGCCAGGAT-----------------------    416.035    198.409    104.594 miR_t26
----------GAATCACCGAAAGCAGCCAGGAT-----------------------      0.389      0.644      0.301
-----------AATCACCGAAAGCAGCCAGGAT-----------------------      3.351      2.652      0.941
--------TCGAATCACCGAAAGCAGCCAGGAT-----------------------      0.097      0.152      0.075
--------------CACCGAAAGCAGCCAGGAT-----------------------      3.011      5.721      2.071
-----------------CGAAAGCAGCCAGGAT-----------------------      1.603      0.000      0.000
-------GTCGAATCACCGAAAGCAGCCAGGAT-----------------------      0.049      0.076      0.076
-----TGGTCGAATCACCGAAAGCAGCCAGGAT-----------------------      0.097      1.326      0.151
----CTGGTCGAATCACCGAAAGCAGCCAGGAT-----------------------      0.049      0.227      0.075
---------CGAATCACCGAAAGCAGCCAGGAT-----------------------      0.194      1.932      0.151
```

```
-------------------AAAGCAGCCAGGAT-----------------------      0.340      0.000      0.000
----------------CCGAAAGCAGCCAGGAT-----------------------      2.331      3.486      2.372
---------------ACCGAAAGCAGCCAGGAT-----------------------      0.049      0.379      0.038
------------------GAAAGCAGCCAGGAT-----------------------      0.291      0.000      0.000
-----------AATCACCGAAAGCAGCCAGGATC----------------------      0.049      0.000      0.000
-------------TCACCGAAAGCAGCCAGGATC----------------------      0.194      0.038      0.038

miRNA: miR_t99,miR_t104 chromosome_4 Start: 3100617 End: 3100762 Condition: TAP
------------------------------------------------TACAAGGCGGGCATGA------------      0.049      0.000      0.000
------------------------------------------------TACAAGGCGGGCATGAAGGTCG------      0.777      1.364      0.791
--------------------------------------TGCTAGCAACTACAAGG---------------------      0.049      0.076      0.076
------------------------------------GCTGCTAGCAACTACAAG----------------------      0.146      0.000      0.000
------------------------------------GCTGCTAGCAACTACAAGGCG-------------------      0.291      0.606      0.606
------------------------------------GCTGCTAGCAACTACAAGGC--------------------      8.402     17.731     12.877
------------------------------------GCTGCTAGCAACTACAAGG---------------------     10.345      3.258      3.916
-----------------------------------TGCTGCTAGCAACTACAAG----------------------      1.457      0.909      0.565
-----------------------------------TGCTGCTAGCAACTACAAGGCG-------------------     11.996     17.655      0.113
-----------------------------------TGCTGCTAGCAACTACAAGG---------------------    406.418    303.543    142.772
-----------------------------------TGCTGCTAGCAACTACAAGGC--------------------    276.936   1042.322    467.322
----------------------------------GTGCTGCTAGCAACTACAAGG---------------------      0.049      0.038      0.038
---------------------------------AGTGCTGCTAGCAACTACAAG----------------------      0.097      0.076      0.038
---------------------------------AGTGCTGCTAGCAACTACAAGGCG-------------------      0.049      0.000      0.000
---------------------------------AGTGCTGCTAGCAACTACAAGGC--------------------      0.049      0.038      0.038
---------------------------------AGTGCTGCTAGCAACTACAAGG---------------------      0.486      0.493      0.113
--------------------------------CAGTGCTGCTAGCAACTACAAGG---------------------      0.097      0.000      0.000
-------------------------TTACTATCAGTGCTGCTAGC-------------------------------      0.146      0.152      0.075
---------------------CGTATTACTATCAG-----------------------------------------      0.049      0.000      0.000
--------------------TCGTATTACTATCAG-----------------------------------------      0.049      0.000      0.000
--------------------TCGTATTACTATCAGTGCTG------------------------------------      0.049      0.000      0.000
-------------------ATCGTATTACTATCAG-----------------------------------------      0.049      0.000      0.000
------------------CATCGTATTACTATC-------------------------------------------      0.049      0.000      0.000
------------------CATCGTATTACTATCAG-----------------------------------------      0.049      0.038      0.188
-----------------CCATCGTATTACTATCAG-----------------------------------------      0.340      0.038      0.527
---------------GGCCATCGTATTACTATCA------------------------------------------      0.486      0.000      0.000
---------------GGCCATCGTATTACTATC-------------------------------------------      0.826      0.038      0.038
---------------GGCCATCGTATTACTATCAG-----------------------------------------     10.296      0.188      0.188
--------------GGGCCATCGTATTACTATCAGT----------------------------------------      1.020      1.174      1.544
--------------GGGCCATCGTATTACTAT--------------------------------------------      0.243      0.265      0.640
--------------GGGCCATCGTATTACTA---------------------------------------------      0.049      0.038      0.038
--------------GGGCCATCGTATTACTATC-------------------------------------------     49.685     13.677     34.413
--------------GGGCCATCGTATTACTATCAG-----------------------------------------    291.020     65.202    105.046
--------------GGGCCATCGTATTACTATCA------------------------------------------     13.745      1.932      4.744
--------------GGGCCATCGTATTACT----------------------------------------------      0.049      0.000      0.000
-------------TGGGCCATCGTATTACTATCAG-----------------------------------------  18353.426   6066.283   5658.240 miR_t99
-------------TGGGCCATCGTATTACTAT--------------------------------------------     18.553     23.262     36.258
-------------TGGGCCATCGTATTAC-----------------------------------------------      0.340      0.000      0.000
-------------TGGGCCATCGTATTACTA---------------------------------------------      0.874      0.947      0.979
-------------TGGGCCATCGTATTACTATC-------------------------------------------   2499.367   1005.421   1328.283
-------------TGGGCCATCGTATTACTATCAGT----------------------------------------     49.442     93.351     68.788
-------------TGGGCCATCGTATTACTATCA------------------------------------------    693.893    118.356    175.340
-------------TGGGCCATCGTATTACT----------------------------------------------      2.040      1.591      1.581
-------------TGGGCCATCGTATTACTATCAGTGC--------------------------------------      0.049      0.000      0.000
------------ATGGGCCATCGTATTACTATC-------------------------------------------      0.049      0.038      0.038
-----------AATGGGCCATCGTATTACTAT--------------------------------------------      0.049      0.000      0.000

-------T                     T           TA    C          G              TG
        GCAAATGGGCCATCGTATTAC ATCAGTGCTGC  GCAA TACAAGGCGG CATGAAGGTCGATT  A
        CGTTTACTCGGTAGCGTAATG TAGTCACGATG  CGTT ATGTTCCGCC GTACTTCCAGCTAG  C
GGAAAGCG                     T           GC    A          A              CC

----AGCGCGTTTACTCGGTAGCGT---------------------------------------------------      0.049      0.000      0.000
-----------TTACTCGGTAGCGTAATG-----------------------------------------------      0.291      0.076      0.038
-----------TTACTCGGTAGCGTAATGT----------------------------------------------      1.068      0.644      0.904
--------CGTTTACTCGGTAGCGTAATGT----------------------------------------------      0.049      0.000      0.000
------------TACTCGGTAGCGTAATGTT---------------------------------------------      1.408     11.707      6.062
----------TTTACTCGGTAGCGTAATGTT---------------------------------------------      2.671      1.326      1.619
---------GTTTACTCGGTAGCGTAATGTT---------------------------------------------      0.049      0.076      0.076
-----------TTACTCGGTAGCGTAATGTT---------------------------------------------     68.238    117.030     87.764
-----------TTACTCGGTAGCGTAATGTTA--------------------------------------------      0.146      0.000      0.000
-----------TTACTCGGTAGCGTAATGTTAG-------------------------------------------      0.146      0.000      0.000 miR_t99*
-------------ACTCGGTAGCGTAATGTTAG-------------------------------------------      0.146      0.227      0.151
------------TACTCGGTAGCGTAATGTTAG-------------------------------------------      0.049      0.000      0.000
-------------ACTCGGTAGCGTAATGTTAGT------------------------------------------      9.422     16.291     10.203
------------TACTCGGTAGCGTAATGTTAGT------------------------------------------      1.506      3.486      2.937
-----------------GGTAGCGTAATGTTAGT------------------------------------------      0.049      0.000      0.000
--------------CTCGGTAGCGTAATGTTAGT------------------------------------------      0.049      0.796      0.941
-----------TTACTCGGTAGCGTAATGTTAGT------------------------------------------      5.585      5.304      4.066
-------------ACTCGGTAGCGTAATGTTAGTC-----------------------------------------      0.049      0.000      0.000
------------------------TAATGTTAGTCACGATGGCCGT------------------------------      0.097      0.341      0.038
-------------------------AATGTTAGTCACGATGGCCGT------------------------------      1.117      0.682      0.339
---------------------------TGTTAGTCACGATGGCCGTT-----------------------------      0.049      0.152      0.152
-------------------------AATGTTAGTCACGATGGCCGTT-----------------------------      0.146      0.455      0.151
-----------------------------TTAGTCACGATGGCCGTTA----------------------------      0.049      0.000      0.000
-----------------------------TTAGTCACGATGGCCGTTAA---------------------------      0.049      0.000      0.000
---------------------------TGTTAGTCACGATGGCCGTTAAT--------------------------      0.097      0.000      0.000
-----------------------------TTAGTCACGATGGCCGTTAAT--------------------------      0.291      0.455      0.264
-------------------------------AGTCACGATGGCCGTTAAT--------------------------      0.097      0.000      0.000
---------------------------------TCACGATGGCCGTTAAT--------------------------      0.049      0.000      0.000
-----------------------------------ACGATGGCCGTTAATG-------------------------      0.049      0.000      0.000
--------------------------------GTCACGATGGCCGTTAATG-------------------------      0.049      0.114      0.038
------------------------------TAGTCACGATGGCCGTTAATG-------------------------      0.389      0.000      0.000
-----------------------------TTAGTCACGATGGCCGTTAATG-------------------------      0.097      0.227      0.227
-------------------------------AGTCACGATGGCCGTTAATG-------------------------      2.088      0.720      0.715
---------------------------------TCACGATGGCCGTTAATG-------------------------      0.194      0.151      0.151
------------------------------------CGATGGCCGTTAATGT------------------------      0.389      0.000      0.000
---------------------------------TCACGATGGCCGTTAATGT------------------------      3.303      1.705      1.807
-----------------------------TTAGTCACGATGGCCGTTAATGT------------------------      5.100     10.532      0.038
----------------------------------CACGATGGCCGTTAATGT------------------------      0.049      0.152      0.113
--------------------------------GTCACGATGGCCGTTAATGT------------------------      1.991      2.500      1.883
--------------------------------------ATGGCCGTTAATGT------------------------      0.049      0.000      0.000
------------------------------TAGTCACGATGGCCGTTAATGT------------------------     12.045      4.167      3.276
-------------------------------AGTCACGATGGCCGTTAATGT------------------------    140.556     75.886     65.437 miR_t104
-------------------------------------GATGGCCGTTAATGT------------------------      0.049      0.000      0.000
-----------------------------TTAGTCACGATGGCCGTTAATGTT-----------------------      1.166      2.993      2.993
--------------------------------GTCACGATGGCCGTTAATGTT-----------------------     68.918     23.376     28.615
------------------------------------CGATGGCCGTTAATGTT-----------------------      5.440     32.544     28.125
-------------------------------AGTCACGATGGCCGTTAATGTT-----------------------    122.877     45.691     40.173
-------------------------------------GATGGCCGTTAATGTT-----------------------      0.340      0.000      0.000
----------------------------------CACGATGGCCGTTAATGTT-----------------------      3.108      3.523      2.221
------------------------------TAGTCACGATGGCCGTTAATGTT-----------------------      5.585      1.591      0.452
-----------------------------------ACGATGGCCGTTAATGTT-----------------------      0.971      6.251      3.351
---------------------------------TCACGATGGCCGTTAATGTT-----------------------    334.732    160.637    148.984
---------------------------------TCACGATGGCCGTTAATGTTC----------------------      0.923      0.114      0.226
---------------------------------------------TTAATGTTCCGCCAGTACTT-----------      0.049      0.075      0.075
--------------------------------------------GTTAATGTTCCGCCAGTACTT-----------      0.486      0.152      0.377

miRNA: miR_t108 chromosome_6 Start: 3067345 End: 3067479 Condition: HS+N
-------------------------CGGCCTAAATTACTACAACACG----------------------      3.983      1.023      0.527
-------TGACACCACTGCGGCCGCCGG-----------------------------------------      3.837      0.947      0.941
-------TGACACCACTGCGGCCGC--------------------------------------------      0.000      0.038      0.038

---GC      A             C  C                  C  CG  T  A        AG
     CCTGAC CCACTGCGGCCGC GG CTAAATTACTACAACACG CG  CC GG CCCGAGGG  A
     GGACTG GGTGGCGCTGGCG CC GATTTGATGATGTTGTGC GC  GG CC GGGCTCCC  /
TTCCT      G             T  C                  T  AT  C  A        CC

----TGGACTGGGGTGGCGCTGGCGT-------------------------------------------      0.097      0.038      0.038
-----GGACTGGGGTGGCGCTGGCGT-------------------------------------------      3.205      1.174      3.050
------GACTGGGGTGGCGCTGGCGT-------------------------------------------      0.000      0.113      0.113
---------------GGCGCTGGCGTCCCGATTTGAT--------------------------------      1.603      0.303      0.828
----------------GCGCTGGCGTCCCGATTTGAT--------------------------------      0.486      0.076      0.075
-------------------CTGGCGTCCCGATTTGATGAT-----------------------------      0.194      0.227      0.188
-----------------------CGTCCCGATTTGATGATGT---------------------------      0.437      0.152      0.075
------------------------GTCCCGATTTGATGATGTT--------------------------      0.097      0.038      0.038
-----------------------CGTCCCGATTTGATGATGTT--------------------------      1.748      1.402      0.489
------------------------GTCCCGATTTGATGATGTTG-------------------------      2.963      0.568      1.167
-----------------------CGTCCCGATTTGATGATGTTG-------------------------    271.010     67.475    126.921
-------------------------TCCCGATTTGATGATGTTG-------------------------      0.049      0.189      0.301
-------------------------TCCCGATTTGATGATGTTGT------------------------     10.102     18.602     17.621
-----------------------CGTCCCGATTTGATGATGTTGT------------------------  14937.093   7820.560   8621.623 miR_t108
--------------------------CCCGATTTGATGATGTTGT------------------------      3.594      2.387      1.431
---------------------------CCGATTTGATGATGTTGT------------------------      3.400      1.402      1.205
----------------------------CGATTTGATGATGTTGT------------------------      1.263      4.243      5.535
------------------------GTCCCGATTTGATGATGTTGT------------------------    434.345    122.599    156.740
------------------------------------------TGTGCTGCATGGCCCAGGGCT------      0.049      0.038      0.038

miRNA: miR_t109 chromosome_10 Start: 3399870 End: 3400011 Condition: HS+N
--------------------------------CCGGGCGCGGGGAGGGCAGG---------------------      0.000      0.038      0.038
-------------------------------ACCGGGCGCGGGGAGGGCAG----------------------      0.000      0.038      0.038
-------------------------------ACCGGGCGCGGGGAGGGCA-----------------------      0.000      0.075      0.075
-------------------------------ACCGGGCGCGGGGAGGGCAGG---------------------      0.194      0.076      0.904
------------------------------TACCGGGCGCGGGGAGGGC------------------------      0.049      0.038      0.113
------------------------------TACCGGGCGCGGGGAGGGCAGG---------------------     25.207     16.367     27.749
------------------------------TACCGGGCGCGGGGAGGGCAGGG--------------------      0.000      0.038      0.038
------------------------------TACCGGGCGCGGGGAGGG-------------------------      0.049      0.075      0.075
------------------------------TACCGGGCGCGGGGAGGGCAG----------------------      0.826      0.833      2.033
------------------------------TACCGGGCGCGGGGAGGGCA-----------------------      0.097      0.414      0.414
-------------------------TAAGGTACCGGGCGCGGGGAGG--------------------------      0.000      0.075      0.075
-------------AAGAAGGAGCCGTAAGGT------------------------------------------      0.049      0.075      0.075
-------------AAGAAGGAGCCGTAAGG-------------------------------------------      0.000      0.113      0.113
------------TAAGAAGGAGCCGTAAGGT------------------------------------------      9.179      3.448      8.697
------------TAAGAAGGAGCCGTAAGGTA-----------------------------------------      0.923      0.682      1.355
------------TAAGAAGGAGCCGTAAG--------------------------------------------      2.137      7.539     17.432
------------TAAGAAGGAGCCGTAAGG-------------------------------------------      3.837     11.934     36.785
-----------ATAAGAAGGAGCCGTAAGG-------------------------------------------      0.000      0.076      0.151
-----------ATAAGAAGGAGCCGTAAGGTA-----------------------------------------      0.097      0.038      0.038
```

```
-----------ATAAGAAGGAGCCGTAAGGT------------------------------------------      0.389      0.114      0.301
----------GATAAGAAGGAGCCGTAAG--------------------------------------------      0.000      0.264      0.264
---------CGATAAGAAGGAGCCGTAAGG-------------------------------------------      0.534      0.265      0.188 miR_t109*

-----                                  C                              CT
     GTGCCGATAAGAAGGAGCCGTAAGGTACCGGGCG GGGGAGGGCAGGGGCAGGGACGGGGATCAG  G
     TACGGCTATTCTTCCTCGGCATTCCATGGCCCGC CCCCTCCCGTCCCCGTCCCTGCCCCTAGTC  C
GCACC                                  A                              CC

-------CGGCTATTCTTCCTCGG-------------------------------------------------      0.146      0.076      0.038
-------CGGCTATTCTTCCTCGGC------------------------------------------------      0.243      0.076      0.075
--------GGCTATTCTTCCTCGGC------------------------------------------------      0.583      0.682      1.883
--------GGCTATTCTTCCTCGGCA-----------------------------------------------     15.590      1.705      5.271
-----TACGGCTATTCTTCCTCGGCA-----------------------------------------------      0.583      0.076      0.038
---------GCTATTCTTCCTCGGCA-----------------------------------------------      0.437      0.565      0.565
-------CGGCTATTCTTCCTCGGCA-----------------------------------------------     72.318     16.784     78.991
------ACGGCTATTCTTCCTCGGCA-----------------------------------------------      0.534      0.189      0.226
----------CTATTCTTCCTCGGCAT----------------------------------------------      0.049      0.038      0.113
--------GGCTATTCTTCCTCGGCAT----------------------------------------------     22.001     34.022     57.417
---------GCTATTCTTCCTCGGCAT----------------------------------------------      1.797      1.402      4.480
------ACGGCTATTCTTCCTCGGCAT----------------------------------------------     14.910      3.145      2.861
-------CGGCTATTCTTCCTCGGCAT----------------------------------------------    633.912    369.200    671.992
-----TACGGCTATTCTTCCTCGGCAT----------------------------------------------      4.517      1.894      1.544
--------GGCTATTCTTCCTCGGCATT---------------------------------------------   1343.493   2005.841   3034.317
-----TACGGCTATTCTTCCTCGGCATT---------------------------------------------    230.456    104.073     53.088
----------CTATTCTTCCTCGGCATT---------------------------------------------      5.391      5.569     11.069
---------GCTATTCTTCCTCGGCATT---------------------------------------------    188.299    170.260    342.698
----CTACGGCTATTCTTCCTCGGCATT---------------------------------------------      0.194      0.189      0.151
------ACGGCTATTCTTCCTCGGCATT---------------------------------------------    254.691     90.472     67.583
-------CGGCTATTCTTCCTCGGCATT---------------------------------------------  31466.541  27971.867  39879.597 miR_t109
--------GGCTATTCTTCCTCGGCATTC--------------------------------------------      1.068      1.250      2.560
-------CGGCTATTCTTCCTCGGCATTC--------------------------------------------     19.816     11.366     17.206
---------GCTATTCTTCCTCGGCATTC--------------------------------------------      0.146      0.114      0.452
-------CGGCTATTCTTCCTCGGCATTCC-------------------------------------------      0.000      0.113      0.113
------------ATTCTTCCTCGGCATTCCAT-----------------------------------------      4.905      0.417      0.188
-----------TATTCTTCCTCGGCATTCCAT-----------------------------------------      3.011      0.417      0.188
---------------------------------------------CCCGTCCCCGTCCCTGCCCCT-------      0.097      0.114      0.188
```

miRNA: miR_t111 chromosome_12 Start: 6402204 End: 6402332 Condition: HS-N

```
--------TGTTCCAGCTGCAGTAC-----------------------------------------      0.000      0.038      0.038
--------TGTTCCAGCTGCAGTACACC--------------------------------------      0.000      0.076      0.076
-----------------TGCAGTACACCTGGTCCCGC-----------------------------      0.000      0.076      0.076
-------------------------ACCTGGTCCCGCTATTTGAATC-------------------      0.049      0.038      0.038
--------------------------CCTGGTCCCGCTATTTGAATC-------------------      0.000      1.591      0.075
----------------------------TGGTCCCGCTATTTGAATC-------------------      0.534      0.833      0.264
-----------------------------------------------TCGCTGATCGGCACCATG-      0.000      0.038      0.038

----          A                               CT         G      G
    GCAGTGTTCC GCTGCAGTACACCTGGTCCCGCTATTTGAAT  CGCTGATCG CACCAT G
    CGTCACAAGG CGACGTCATGTGGACCAGGGCGATGGACTTA  GCGACTAGT GTGGTG G
GGAC          C                               TC         G      G

--------------------------GGACCAGGGCGATGGACTTAT-------------------      0.680      0.114      0.264
------------------------GTGGACCAGGGCGATGGACTTAT-------------------      0.146      0.152      0.152
------------------------GTGGACCAGGGCGATGGACTTA--------------------      0.000      0.038      0.038
---------------------------GACCAGGGCGATGGACTT---------------------      3.983      0.303      1.092
---------------------CATGTGGACCAGGGCGATGGACTT---------------------      0.000      0.227      0.227
----------------------------ACCAGGGCGATGGACTT---------------------      0.194      0.227      0.188
--------------------TCATGTGGACCAGGGCGATGGACTT---------------------      0.000      0.038      0.038
----------------------ATGTGGACCAGGGCGATGGACTT---------------------      0.146      0.417      0.417
-------------------------TGGACCAGGGCGATGGACTT---------------------      1.117      9.358      6.890
------------------------GTGGACCAGGGCGATGGACTT---------------------   6352.765   3761.937   1514.918 miR_t111
-----------------------TGTGGACCAGGGCGATGGACTT---------------------     41.234     17.579      1.468
--------------------------GGACCAGGGCGATGGACTT---------------------      6.071      8.714      7.417
---------------------------GACCAGGGCGATGGACT----------------------      0.534      0.227      0.113
---------------------CATGTGGACCAGGGCGATGGACT----------------------      0.000      0.076      0.188
-------------------------TGGACCAGGGCGATGGACT----------------------      0.097      0.189      0.339
--------------------------GGACCAGGGCGATGGACT----------------------      1.068      1.061      1.431
----------------------ATGTGGACCAGGGCGATGGACT----------------------      0.583      0.076      0.075
-----------------------TGTGGACCAGGGCGATGGACT----------------------    143.228     40.955     56.476
------------------------GTGGACCAGGGCGATGGACT----------------------    125.306     41.713     36.634
-----------------------TGTGGACCAGGGCGATGGAC-----------------------      1.603      0.038      0.301
-------------------------TGGACCAGGGCGATGGAC-----------------------      0.000      0.038      0.038
------------------------GTGGACCAGGGCGATGGAC-----------------------     11.802      1.553      1.431
---------------------CATGTGGACCAGGGCGATGGA------------------------      0.000      0.038      0.038
------------------------GTGGACCAGGGCGATGGA------------------------     14.182      0.417      0.527
-----------------------TGTGGACCAGGGCGATGGA------------------------      0.729      0.076      0.226
------------------CGTCATGTGGACCAGGGCGAT---------------------------      0.049      0.189      0.414
------TCACAAGGCCGACGTCATGT----------------------------------------      0.000      0.114      0.114
----CGTCACAAGGCCGACGTCAT------------------------------------------      0.049      0.265      0.075
------TCACAAGGCCGACGTCAT------------------------------------------      0.097      0.152      0.264
-----GTCACAAGGCCGACGTCAT------------------------------------------      0.146      0.152      0.038
```

miRNA: miR_t122 chromosome_9 Start: 6518807 End: 6519095 Condition: TAP

```
---------------------------------------------------------------------------------------------------TTAGAGCCTGTGGGGCTGACCTG-------------------------      0.049      0.000      0.000
---------------------------------------------------------------------------------------TGCGCACAGCCCTTAGAGCCT---------------------------------------      0.146      0.000      0.000
---------------------------------------------------------------------------------------TGCGCACAGCCCTTAGAGCCTG--------------------------------------      0.146      0.000      0.000
---------------------------------------------------------------------------------------TGCGCACAGCCCTTAGAGC-----------------------------------------      0.049      0.000      0.000
---------------------------------------------------------------------------------------TGCGCACAGCCCTTAGAGCC----------------------------------------      0.340      0.000      0.000
-------------------------------------------------------GGACGCGGACCCGGCGCA--------------------------------------------------------------------------      0.097      0.000      0.000
-------------------------------------------------------GGACGCGGACCCGGCGCAGCGG----------------------------------------------------------------------      0.049      0.189      0.189
-------------------------------------------------------GGACGCGGACCCGGCGCAGC------------------------------------------------------------------------      0.583      0.606      0.828
-------------------------------------------------------GGACGCGGACCCGGCGCAGCG-----------------------------------------------------------------------      0.146      0.114      0.075
-------------------------------------------------------GGACGCGGACCCGGCGCAG-------------------------------------------------------------------------      1.700      0.985      1.393
------------------------------------------------------TGGACGCGGACCCGGCGCA--------------------------------------------------------------------------      0.437      0.227      0.301
------------------------------------------------------TGGACGCGGACCCGGCGCAGC------------------------------------------------------------------------     64.547     80.394     65.588
------------------------------------------------------TGGACGCGGACCCGGCGCAGCG-----------------------------------------------------------------------     13.356     20.080      6.627
------------------------------------------------------TGGACGCGGACCCGGCGCAGCGGCG--------------------------------------------------------------------      0.146      0.227      0.075
------------------------------------------------------TGGACGCGGACCCGGC-----------------------------------------------------------------------------      0.291      0.000      0.000
------------------------------------------------------TGGACGCGGACCCGGCGC---------------------------------------------------------------------------      0.437      1.856      1.355
------------------------------------------------------TGGACGCGGACCCGGCGCAGCGGC---------------------------------------------------------------------      0.049      3.182      0.038
------------------------------------------------------TGGACGCGGACCCGGCGCAGCGG----------------------------------------------------------------------      7.528     45.766      7.794
------------------------------------------------------TGGACGCGGACCCGGCGCAG-------------------------------------------------------------------------    124.383     68.422     72.779
-----------------------------------------------------TTGGACGCGGACCCGGCGCA--------------------------------------------------------------------------     26.081     13.828     16.190
-----------------------------------------------------TTGGACGCGGACCCG-------------------------------------------------------------------------------      0.194      0.000      0.000
-----------------------------------------------------TTGGACGCGGACCCGGC-----------------------------------------------------------------------------      2.477     10.191     16.303
-----------------------------------------------------TTGGACGCGGACCCGGCGCAG-------------------------------------------------------------------------   1293.516    871.077    922.972 miR_t122
-----------------------------------------------------TTGGACGCGGACCCGGCGCAGCGGC---------------------------------------------------------------------      0.049      0.303      0.113
-----------------------------------------------------TTGGACGCGGACCCGGCG----------------------------------------------------------------------------      1.166      4.357      4.819
-----------------------------------------------------TTGGACGCGGACCCGGCGCAGC------------------------------------------------------------------------     12.968     19.095     15.211
-----------------------------------------------------TTGGACGCGGACCCGG------------------------------------------------------------------------------      0.146      0.000      0.000
-----------------------------------------------------TTGGACGCGGACCCGGCGCAGCG-----------------------------------------------------------------------      0.826      3.334      1.468
-----------------------------------------------------TTGGACGCGGACCCGGCGC---------------------------------------------------------------------------      9.034     51.601     50.753
-----------------------------------------------------TTGGACGCGGACCCGGCGCAGCGG----------------------------------------------------------------------      0.389      3.069      0.452
--------------------------------------------------AAGTTGGACGCGGACCCGGCG----------------------------------------------------------------------------      0.097      0.038      0.038
---------------------------------AGGCACCGCAACGCACCAAGT---------------------------------------------------------------------------------------------      0.049      0.000      0.000
---------------------------------AGGCACCGCAACGCACCAAG----------------------------------------------------------------------------------------------      0.194      0.114      0.301
--------------------------------TAGGCACCGCAACGCACCAAGT---------------------------------------------------------------------------------------------      1.846      0.341      0.113
--------------------------------TAGGCACCGCAACGCACCAAG----------------------------------------------------------------------------------------------     13.696      9.358      9.827
----------------------TAGGCCACGCTAGGCACCGCA--------------------------------------------------------------------------------------------------------      0.194      0.076      0.113
----------------------TAGGCCACGCTAGGCACCGC---------------------------------------------------------------------------------------------------------      0.194      0.758      1.581
-----------TCCAAGCGATGTAGGCCACGC-------------------------------------------------------------------------------------------------------------------      0.049      0.303      0.188
----------TTCCAAGCGATGTAGGCCACG--------------------------------------------------------------------------------------------------------------------      0.097      0.303      0.301
----------TTCCAAGCGATGTAGGCCACGC-------------------------------------------------------------------------------------------------------------------      0.389      7.160      4.029
----CCAGG-TTCCAAGCGATGTAGG-------------------------------------------------------------------------------------------------------------------------      0.049      0.000      0.000

TCA      -    A                                                           C  C                       A         G G    CCT      C  C-      A  T   T
   ACCAGG TTCC AGCGATGTAGGCCACGCTAGGCACCGCAACGCACCAAGTTGGACGCGGACCCGGCGCAG GG GGGAGCAGCTGCGCACAGCCCTT GAGCCTGTG G CTGA   GACGCT TT  GAAGAC TC TCC A
   TGGTCC AGGG TCGCTACATCCGGTGCGATCCGTGGCGTTGCGTGGTTCAACCTGCGCCTGGGCCGCGTC TC TCCTCGTCGACGCGTGTCGGGAA CTCGGACAT C GACT   TTGTGG AG  CTTTTG AG AGG /
TAC      C    C                                                           T  C                       A         - G    T--      C  TA      G  C   -

----GGTCCCAGGGCTCGCTACAT---------------------------------------------------------------------------------------------------------------------------      0.049      0.000      0.000
--------CCAGGGCTCGCTACATCCGG-----------------------------------------------------------------------------------------------------------------------      0.049      0.000      0.000
----------AGGGCTCGCTACATCCGGT----------------------------------------------------------------------------------------------------------------------      0.049      0.000      0.000
------------GGCTCGCTACATCCGGT----------------------------------------------------------------------------------------------------------------------      0.049      0.000      0.000
--------CCAGGGCTCGCTACATCCGGT----------------------------------------------------------------------------------------------------------------------      0.923      0.909      0.038
-------CCCAGGGCTCGCTACATCCGGT----------------------------------------------------------------------------------------------------------------------      1.214      0.265      0.038
---------CAGGGCTCGCTACATCCGGT----------------------------------------------------------------------------------------------------------------------      0.097      0.038      0.038
-----GTCCCAGGGCTCGCTACATCCGGT----------------------------------------------------------------------------------------------------------------------      0.049      0.000      0.000
------TCCCAGGGCTCGCTACATCCGGT----------------------------------------------------------------------------------------------------------------------      0.049      0.000      0.000
------------------------CCGGTGCGATCCGTGGCGTT-------------------------------------------------------------------------------------------------------      0.049      0.075      0.075
-------------------------CGGTGCGATCCGTGGCGTT-------------------------------------------------------------------------------------------------------      0.049      0.152      0.113
------------------------------CGATCCGTGGCGTTGCGT---------------------------------------------------------------------------------------------------      0.049      0.038      0.038
--------------------------GGTGCGATCCGTGGCGTTGCGT---------------------------------------------------------------------------------------------------      0.243      0.000      0.000
-----------------------------GCGATCCGTGGCGTTGCGT---------------------------------------------------------------------------------------------------      0.049      0.000      0.000
-------------------------------GATCCGTGGCGTTGCGTGG-------------------------------------------------------------------------------------------------      0.097      0.000      0.000
------------------------------CGATCCGTGGCGTTGCGTGGT------------------------------------------------------------------------------------------------      1.214      0.114      0.264
-----------------------------GCGATCCGTGGCGTTGCGTGGT------------------------------------------------------------------------------------------------      0.194      0.038      0.038
-------------------------------GATCCGTGGCGTTGCGTGGT------------------------------------------------------------------------------------------------      0.680      0.076      0.038
------------------------------CGATCCGTGGCGTTGCGTGGTT-----------------------------------------------------------------------------------------------      0.049      0.000      0.000
-------------------------------GATCCGTGGCGTTGCGTGGTT-----------------------------------------------------------------------------------------------      0.486      0.114      0.114
--------------------------------------GGCGTTGCGTGGTTCAACCT-----------------------------------------------------------------------------------------      0.340      0.000      0.000
----------------------------------------------------CAACCTGCGCCTGGGCCGCGT--------------------------------------------------------------------------      0.049      0.038      0.038
-------------------------------------------------------------CCTGGGCCGCGTCTTCCTCCT-----------------------------------------------------------------      1.263      0.114      0.114
-----------------------------------------------------------------GGCCGCGTCTTCCTCCT-----------------------------------------------------------------      0.049      0.038      0.038
--------------------------------------------------------------CTGGGCCGCGTCTTCCTCCT-----------------------------------------------------------------      0.049      0.000      0.000
----------------------------------------------------------------GGGCCGCGTCTTCCTCCTCGT--------------------------------------------------------------      2.623      0.682      1.130
-----------------------------------------------------------------GGCCGCGTCTTCCTCCTCGT--------------------------------------------------------------      0.097      0.075      0.075
----------------------------------------------------------------GGGCCGCGTCTTCCTCCTCGTC-------------------------------------------------------------      0.049      0.000      0.000
-----------------------------------------------------------------------GTCTTCCTCCTCGTCGACGCGT------------------------------------------------------      2.234      3.334      3.313
----------------------------------------------------------------------CGTCTTCCTCCTCGTCGACGCGT------------------------------------------------------      0.049      0.038      0.038
--------------------------------------------------------------------------TTCCTCCTCGTCGACGCGTGT----------------------------------------------------      0.049      0.114      0.114
-------------------------------------------------------------------------CTTCCTCCTCGTCGACGCGTGT----------------------------------------------------      0.534      0.227      0.301
------------------------------------------------------------------------TCTTCCTCCTCGTCGACGCGTGT----------------------------------------------------      0.146      0.379      0.640
----------------------------------------------------------------------------------CGTCGACGCGTGTCGGGAAAC--------------------------------------------      0.291      0.265      0.226
-----------------------------------------------------------------------------------GTCGACGCGTGTCGGGAAACT-------------------------------------------      0.097      0.000      0.000
```

```
-------------------------------------------------------------------------------------CGACGCGTGTCGGGAAACT-------------------------------------------      0.049      1.137      0.602
----------------------------------------------------------------------------------CGTCGACGCGTGTCGGGAAACT-------------------------------------------      0.194      0.493      0.113
--------------------------------------------------------------------------------------------TGTCGGGAAACTCGGACAT------------------------------------      0.049      0.000      0.000
---------------------------------------------------------------------------------------------GTCGGGAAACTCGGACAT-CGGA-------------------------------      0.097      0.114      0.226

miRNA: miR_t124 chromosome_6 Start: 2201619 End: 2201697 Condition: TAP
----------------------TAAGATTGCGGTAA---AGAT      0.000      0.000      0.000
--------------------TGTAAGATTGCGGTAA---AGAT      0.000      0.000      0.000
-------------------TTGTAAGATTGCGGTAA---AGA-      0.243      0.000      0.000
-----------GGGCGGCGTTGTAAGATT--------------      3.011      0.417      0.452
----------TGGGCGGCGTTGTAAGATTGC------------      0.049      0.076      0.301
----------TGGGCGGCGTTGTAAGAT---------------      0.097      0.152      0.113
----------TGGGCGGCGTTGTAAGATT--------------     25.887      2.955      4.932
----------TGGGCGGCGTTGTAAGATTG-------------      0.243      0.000      0.000
---------TTGGGCGGCGTTGTAA------------------      0.340      0.000      0.000
---------TTGGGCGGCGTTGTAAGAT---------------     16.367     11.252     12.387
---------TTGGGCGGCGTTGTAAGATTG-------------      5.051      0.265      0.226
---------TTGGGCGGCGTTGTAAGATT--------------   4185.216    623.075    633.588 miR_t124
---------TTGGGCGGCGTTGTAAGA----------------      3.351      3.789      4.556
---------TTGGGCGGCGTTGTAAG-----------------      2.186      3.599      4.932

-----                              A---  A
     CGAGTTGGGCGGCGTTGTAAGATTGCGGTA    AG T
     GCTCAACCCGCCGCAACATTCTAACGCCAT    TC G
CCGTC                              GGTG  G

----CGCTCAACCCGCCGCAA----------------------      0.049      0.000      0.000
----CGCTCAACCCGCCGCAACAT-------------------      0.340      0.076      0.151
-----GCTCAACCCGCCGCAACATT------------------      0.049      0.000      0.000
-------TCAACCCGCCGCAACATT------------------      0.049      0.075      0.075
----CGCTCAACCCGCCGCAACATT------------------      1.117      0.038      0.264
-------TCAACCCGCCGCAACATTC-----------------      0.194      0.226      0.226
--------CAACCCGCCGCAACATTC-----------------      0.049      0.000      0.000
----CGCTCAACCCGCCGCAACATTCT----------------      0.049      0.000      0.000
-----GCTCAACCCGCCGCAACATTCT----------------      0.049      0.114      0.114
-------TCAACCCGCCGCAACATTCT----------------     51.142      5.607     18.524 miR_t124*
--------CAACCCGCCGCAACATTCT----------------      0.826      0.113      0.113
------CTCAACCCGCCGCAACATTCT----------------      0.631      0.414      0.414

miRNAs identified in Chavez-Montes et al.:

miRNA: miR_t2,miR_t38 chromosome_15 Start: 110001 End: 110415 Condition: Nutrient-replete
----------------------------------------------------------------------------------------------------------------------------------------------------------------------------------------------------------TCTTGGGCGGCAGCTGAAGTACAGCACT-      0.073      0.000      0.000
---------------------------------------------------------------------------------------------------------------------------------------------------------------------------------------------------TCTAAGGTCTTGGGCGGCAGC---------------      0.367      0.500      0.163
-------------------------------------------------------------------------------------------------------------------------------------------------------------------------------------------------ACTCTAAGGTCTTGGGCGGCA-----------------      0.073      0.000      0.082
---------------------------------------------------------------------------------------------------------------------------------------------------------------------------------------------ACTAACTCTAAGGTCTTGGGC---------------------      0.220      0.100      0.163
------------------------------------------------------------------------------------------------------------------------------------------------------------------------------------------TACACTAACTCTAAGGTCTTG------------------------      2.717      2.801      4.974
-------------------------------------------------------------------------------------------------------------------------------------------------------------------------------------TCCTGTACACTAACTCTAAGG-----------------------------      0.073      0.000      0.000
----------------------------------------------------------------------------------------------------------------------------------------------------------------TGGTGCTGGGAGTGCTGTAC---------------------------------------------------      0.073      0.100      0.082
------------------------------------------------------------------------------------------------------------------------------------------------------------CACTGGTGCTGGGAGTGCTGT------------------------------------------------------      0.147      0.100      0.000
-----------------------------------------------------------------------------------------------------------------------------------------------------------TCACTGGTGCTGGGAGTGCTG-------------------------------------------------------      0.220      0.300      0.000
---------------------------------------------------------------------------------------------------------------------------------------------------------CGTCACTGGTGCTGGGAGTGC---------------------------------------------------------      0.073      0.000      0.000
-------------------------------------------------------------------------------------------------------------------------------------------------------GGCGTCACTGGTGCTGGGAGT-----------------------------------------------------------      0.073      0.000      0.082
------------------------------------------------------------------------------------------------------------------------------------------------------TGGCGTCACTGGTGCTGGGAGT-----------------------------------------------------------      0.441      0.300      0.408
------------------------------------------------------------------------------------------------------------------------------------------------------TGGCGTCACTGGTGCTGGG--------------------------------------------------------------      1.762      0.900      0.978
------------------------------------------------------------------------------------------------------------------------------------------------------TGGCGTCACTGGTGCTGGGAG------------------------------------------------------------      2.863      2.801      3.017
------------------------------------------------------------------------------------------------------------------------------------------------------TGGCGTCACTGGTGCTGGGA-------------------------------------------------------------      0.147      0.000      0.082
--------------------------------------------------------------------------------------------------------------------------------------TGTCAACCTGACGCCGATGGCG---------------------------------------------------------------------------      0.073      0.000      0.000
----------------------------------------------------------------------------------------------------------------------------------TCGCTGTCAACCTGACGCCGA--------------------------------------------------------------------------------      0.147      0.200      0.163
----------------------------------------------------------------------------------------------------------------------------------TCGCTGTCAACCTGACGCCGAT-------------------------------------------------------------------------------      0.073      0.000      0.000
------------------------------------------------------------------------------------------------------------------------------TTGGTCGCTGTCAACCTGACG------------------------------------------------------------------------------------      0.073      0.000      0.000
-------------------------------------------------------------------------------------------------------------------------AGGCGTTGGTCGCTGTCAACC-----------------------------------------------------------------------------------------      0.073      0.900      0.652
------------------------------------------------------------------------------------------------------------------------AAGGCGTTGGTCGCTGTCAA-------------------------------------------------------------------------------------------      0.073      0.500      0.245
----------------------------------------------------------------------------------------------------------------------ACAAGGCGTTGGTCGCTGTC---------------------------------------------------------------------------------------------      0.073      0.200      0.082
---------------------------------------------------------------------------------------------------------------------AACAAGGCGTTGGTCGCTGTC---------------------------------------------------------------------------------------------      0.294      1.400      2.691
--------------------------------------------------------------------------------------------------------------------GAACAAGGCGTTGGTCGCT------------------------------------------------------------------------------------------------      0.073      0.000      0.000
-------------------------------------------------------------------------------------------------------------------TGAACAAGGCGTTGGTCGCT------------------------------------------------------------------------------------------------      1.175      0.900      1.712
-------------------------------------------------------------------------------------------------------------------TGAACAAGGCGTTGGTCGC-------------------------------------------------------------------------------------------------      0.073      0.000      0.326
-------------------------------------------------------------------------------------------------------------------TGAACAAGGCGTTGGTCGCTGT----------------------------------------------------------------------------------------------      0.073      0.000      0.082
-------------------------------------------------------------------------------------------------------------------TGAACAAGGCGTTGGTCGCTG-----------------------------------------------------------------------------------------------      1.175      3.301      2.772
-----------------------------------------------------------------------------------------------------------------AGTGAACAAGGCGTTGGTCGCT------------------------------------------------------------------------------------------------      0.073      0.000      0.000
-----------------------------------------------------------------------------------------------------------------AGTGAACAAGGCGTTGGTCG--------------------------------------------------------------------------------------------------      0.073      0.200      0.245
-----------------------------------------------------------------------------------------------------------------AGTGAACAAGGCGTTGGTCGC-------------------------------------------------------------------------------------------------      0.367      0.500      0.571
-----------------------------------------------------------------------------------------------------------------AGTGAACAAGGCGTTGGTCGCTG-----------------------------------------------------------------------------------------------      0.073      0.000      0.000
---------------------------------------------------------------------------------------------------------------GCAGTGAACAAGGCGTTGGTC---------------------------------------------------------------------------------------------------      0.514      0.400      0.326
---------------------------------------------------------------------------------------------------------------GCAGTGAACAAGGCGTTGGTCG--------------------------------------------------------------------------------------------------      0.220      0.200      0.245
---------------------------------------------------------------------------------------------------------------GCAGTGAACAAGGCGTTGGT----------------------------------------------------------------------------------------------------      0.073      0.100      0.082
-------------------------------------------------------------------------------------------------------------CGGCAGTGAACAAGGCGTTG------------------------------------------------------------------------------------------------------      0.073      0.600      0.408
-------------------------------------------------------------------------------------------------------------CGGCAGTGAACAAGGCGTT-------------------------------------------------------------------------------------------------------      0.073      0.200      0.163
-------------------------------------------------------------------------------------------------------------CGGCAGTGAACAAGGCGTTGGT----------------------------------------------------------------------------------------------------      0.073      0.000      0.000
-------------------------------------------------------------------------------------------------------------CGGCAGTGAACAAGGCGTTGG-----------------------------------------------------------------------------------------------------      3.157      6.401      5.055
------------------------------------------------------------------------------------------------------------ACGGCAGTGAACAAGGCGTTG------------------------------------------------------------------------------------------------------      3.084      3.101      3.180
------------------------------------------------------------------------------------------------------------ACGGCAGTGAACAAGGCG---------------------------------------------------------------------------------------------------------      5.213      3.401      4.403
------------------------------------------------------------------------------------------------------------ACGGCAGTGAACAAGGCGTT-------------------------------------------------------------------------------------------------------     35.242     21.004     28.049
------------------------------------------------------------------------------------------------------------ACGGCAGTGAACAAGGCGT--------------------------------------------------------------------------------------------------------      9.031      4.501      3.017
-----------------------------------------------------------------------------------------------------------CACGGCAGTGAACAAGGCGTT-------------------------------------------------------------------------------------------------------      0.220      0.200      0.408
-----------------------------------------------------------------------------------------------------------CACGGCAGTGAACAAGGCGT--------------------------------------------------------------------------------------------------------      6.167      5.301      6.768
-----------------------------------------------------------------------------------------------------------CACGGCAGTGAACAAGGCG---------------------------------------------------------------------------------------------------------     12.555      6.001      9.703
----------------------------------------------------------------------------------------------------------ACACGGCAGTGAACAAGGCGTTG------------------------------------------------------------------------------------------------------      0.147      0.200      0.082
----------------------------------------------------------------------------------------------------------ACACGGCAGTGAACAAGGCGT--------------------------------------------------------------------------------------------------------   2523.458   1532.810   2065.860
----------------------------------------------------------------------------------------------------------ACACGGCAGTGAACAAGGCGTT-------------------------------------------------------------------------------------------------------     44.493     17.303     27.886
----------------------------------------------------------------------------------------------------------ACACGGCAGTGAACAAGGC----------------------------------------------------------------------------------------------------------      9.838      7.401     10.518
----------------------------------------------------------------------------------------------------------ACACGGCAGTGAACAAGGCG---------------------------------------------------------------------------------------------------------   4725.554   2420.989   4572.109 miR_t2
----------------------------------------------------------------------------------------------------------ACACGGCAGTGAACAAGG-----------------------------------------------------------------------------------------------------------     17.621     10.802     17.205
---------------------------------------------------------------------------------------------------------GACACGGCAGTGAACAAG------------------------------------------------------------------------------------------------------------      0.294      0.300      0.082
---------------------------------------------------------------------------------------------------------GACACGGCAGTGAACAAGGC----------------------------------------------------------------------------------------------------------      0.661      0.300      0.978
---------------------------------------------------------------------------------------------------------GACACGGCAGTGAACAAGGCGT--------------------------------------------------------------------------------------------------------      0.808      0.500      0.652
---------------------------------------------------------------------------------------------------------GACACGGCAGTGAACAAGGCG---------------------------------------------------------------------------------------------------------     51.174     28.106     49.575
---------------------------------------------------------------------------------------------------------GACACGGCAGTGAACAAGG-----------------------------------------------------------------------------------------------------------      0.294      0.400      0.245
--------------------------------------------------------------------------------------------------------GGACACGGCAGTGAACAAGG-----------------------------------------------------------------------------------------------------------      0.220      1.600      0.734
--------------------------------------------------------------------------------------------------------GGACACGGCAGTGAACAAGGCG---------------------------------------------------------------------------------------------------------      0.073      0.100      0.000
--------------------------------------------------------------------------------------------------------GGACACGGCAGTGAACAAG------------------------------------------------------------------------------------------------------------      0.220      1.000      0.897
-------------------------------------------------------------------------------------------------------AGGACACGGCAGTGAACAAGGCG---------------------------------------------------------------------------------------------------------      1.615      1.800      0.245
-------------------------------------------------------------------------------------------------------AGGACACGGCAGTGAACA--------------------------------------------------------------------------------------------------------------      0.954      5.701      3.588
-------------------------------------------------------------------------------------------------------AGGACACGGCAGTGAACAAGGC----------------------------------------------------------------------------------------------------------      0.587      1.600      0.652
-------------------------------------------------------------------------------------------------------AGGACACGGCAGTGAACAAG------------------------------------------------------------------------------------------------------------     81.129    196.540    141.306
-------------------------------------------------------------------------------------------------------AGGACACGGCAGTGAACAA-------------------------------------------------------------------------------------------------------------      1.762      6.701      2.935
-------------------------------------------------------------------------------------------------------AGGACACGGCAGTGAACAAGGCGT--------------------------------------------------------------------------------------------------------      0.073      0.000      0.082
-------------------------------------------------------------------------------------------------------AGGACACGGCAGTGAACAAGG-----------------------------------------------------------------------------------------------------------     76.063    379.477    171.557
------------------------------------------------------------------------------------------------------CAGGACACGGCAGTGAACA--------------------------------------------------------------------------------------------------------------      0.073      1.700      0.734
------------------------------------------------------------------------------------------------------CAGGACACGGCAGTGAACAAG------------------------------------------------------------------------------------------------------------     26.578     68.014     48.189
------------------------------------------------------------------------------------------------------CAGGACACGGCAGTGAACAA-------------------------------------------------------------------------------------------------------------      0.147      2.300      0.978
------------------------------------------------------------------------------------------------------CAGGACACGGCAGTGAACAAGG-----------------------------------------------------------------------------------------------------------      0.514      2.200      0.571
-----------------------------------------------------------------------------------------------------GCAGGACACGGCAGTGAACA--------------------------------------------------------------------------------------------------------------      0.073      0.000      0.082
----------------------------------------------------------------------------------------------------AGCAGGACACGGCAGTGAA----------------------------------------------------------------------------------------------------------------      0.073      0.100      0.000
----------------------------------------------------------------------------------------------------AGCAGGACACGGCAGTGAACAA-------------------------------------------------------------------------------------------------------------      0.367      1.200      1.223
----------------------------------------------------------------------------------------------------AGCAGGACACGGCAGTGAACA--------------------------------------------------------------------------------------------------------------     10.866     52.211     49.086
----------------------------------------------------------------------------------------------------AGCAGGACACGGCAGTGAAC---------------------------------------------------------------------------------------------------------------      0.587      1.300      2.120
----------------------------------------------------------------------------------------------------AGCAGGACACGGCAGTGAACAAGG-----------------------------------------------------------------------------------------------------------      0.073      0.000      0.082
----------------------------------------------------------------------------------------------------AGCAGGACACGGCAGTGAACAAG------------------------------------------------------------------------------------------------------------      0.220      0.600      0.408
---------------------------------------------------------------------------------------------------AAGCAGGACACGGCAGTGAACA--------------------------------------------------------------------------------------------------------------      0.441      0.600      1.386
---------------------------------------------------------------------------------------------------AAGCAGGACACGGCAGTGAAC---------------------------------------------------------------------------------------------------------------      0.147      0.800      0.978
--------------------------------------------------------------------------------------------------GAAGCAGGACACGGCAGTGA-----------------------------------------------------------------------------------------------------------------      0.073      0.100      0.163
-------------------------------------------------------------------------------------------------TGAAGCAGGACACGGCAGTGAAC---------------------------------------------------------------------------------------------------------------      0.220      0.400      1.468
-------------------------------------------------------------------------------------------------TGAAGCAGGACACGGCAGTGAA----------------------------------------------------------------------------------------------------------------      0.073      0.300      1.142
-------------------------------------------------------------------------------------------------TGAAGCAGGACACGGCAGTG------------------------------------------------------------------------------------------------------------------      0.294      0.400      0.489
-------------------------------------------------------------------------------------------------TGAAGCAGGACACGGCAGTGA-----------------------------------------------------------------------------------------------------------------      7.489     15.903     17.857
----------------------------------------------------------------------------------------------AGCTGAAGCAGGACACGGCAG--------------------------------------------------------------------------------------------------------------------      1.175      0.800      2.038
---------------------------------------------------------------------------------------------CAGCTGAAGCAGGACACGGCAG--------------------------------------------------------------------------------------------------------------------      0.147      0.100      0.245
--------------------------------------------------------------------------------------------ACAGCTGAAGCAGGACACGGCA---------------------------------------------------------------------------------------------------------------------      0.073      0.000      0.000
------------------------------------------------------------------------------------------TCACAGCTGAAGCAGGACACG------------------------------------------------------------------------------------------------------------------------      0.514      1.500      0.815
----------------------------------------------------------------------------------------TCTCACAGCTGAAGCAGGACA--------------------------------------------------------------------------------------------------------------------------     14.904     12.403     19.651
--------------------------------------------------------------------------------------CATCTCACAGCTGAAGCAGGACA--------------------------------------------------------------------------------------------------------------------------      0.147      0.100      0.000
-----------------------------------------------------------------------------------AGGACATCTCACAGCTGAAG--------------------------------------------------------------------------------------------------------------------------------      0.441      0.700      0.652
-----------------------------------------------------------------------------------AGGACATCTCACAGCTGAAGC-------------------------------------------------------------------------------------------------------------------------------      0.367      1.100      0.489
----------------------------------------------------------------------------------AAGGACATCTCACAGCTGAAG--------------------------------------------------------------------------------------------------------------------------------      0.073      0.500      0.245
--------------------------------------------------------------------------------AGAAGGACATCTCACAGCTGA----------------------------------------------------------------------------------------------------------------------------------      1.542      0.300      3.751
--------------------------------------------------------------------------------AGAAGGACATCTCACAGC-------------------------------------------------------------------------------------------------------------------------------------      0.147      0.000      0.000
-------------------------------------------------------------------------------CAGAAGGACATCTCACAGCTG-----------------------------------------------------------------------------------------------------------------------------------      0.073      0.100      0.326
------------------------------------------------------------------------------GCAGAAGGACATCTCACAGC-------------------------------------------------------------------------------------------------------------------------------------      0.220      0.000      0.245
-----------------------------------------------------------------------------TGCAGAAGGACATCTCACA---------------------------------------------------------------------------------------------------------------------------------------      0.073      0.100      0.082
-----------------------------------------------------------------------------TGCAGAAGGACATCTCACAG--------------------------------------------------------------------------------------------------------------------------------------      0.587      0.600      1.794
-----------------------------------------------------------------------------TGCAGAAGGACATCTCACAGC-------------------------------------------------------------------------------------------------------------------------------------     53.450     49.010    124.020
-----------------------------------------------------------------------------TGCAGAAGGACATCTCACAGCT------------------------------------------------------------------------------------------------------------------------------------      0.881      0.300      1.060
--------------------------------------------------------------------------ACATGCAGAAGGACATCTCAC----------------------------------------------------------------------------------------------------------------------------------------      0.073      0.000      0.163
--------------------------------------------------------------------TCGGCGACATGCAGAAGGACAT---------------------------------------------------------------------------------------------------------------------------------------------      0.073      0.000      0.000
--------------------------------------------------------------------TCGGCGACATGCAGAAGGACA----------------------------------------------------------------------------------------------------------------------------------------------      4.112      2.300      3.669
--------------------------------------------------------------------TCGGCGACATGCAGAAGGAC-----------------------------------------------------------------------------------------------------------------------------------------------      0.220      0.100      0.163
-------------------------------------------------------------------ATCGGCGACATGCAGAAGGACA----------------------------------------------------------------------------------------------------------------------------------------------      0.073      0.000      0.000
-----------------------------------------------------------------ACATCGGCGACATGCAGAAGG-------------------------------------------------------------------------------------------------------------------------------------------------      0.073      1.100      0.652
-----------------------------------------------------------------ACATCGGCGACATGCAGAAGGAC-----------------------------------------------------------------------------------------------------------------------------------------------      0.220      0.300      0.408
----------------------------------------------------------------GACATCGGCGACATGCAGAAG--------------------------------------------------------------------------------------------------------------------------------------------------      0.073      0.000      0.082
--------------------------------------------------------------AGGACATCGGCGACATGCAGA----------------------------------------------------------------------------------------------------------------------------------------------------      0.220      1.800      1.549
--------------------------------------------------------------AGGACATCGGCGACATGCAG-----------------------------------------------------------------------------------------------------------------------------------------------------      1.175      1.900      2.935
-------------------------------------------------------------AAGGACATCGGCGACATGCAGA----------------------------------------------------------------------------------------------------------------------------------------------------      0.220      0.200      0.000
-------------------------------------------------------------AAGGACATCGGCGACATGCAG-----------------------------------------------------------------------------------------------------------------------------------------------------      1.982      3.901      5.463
-----------------------------------------------------------AGAAGGACATCGGCGACATGCA------------------------------------------------------------------------------------------------------------------------------------------------------      0.147      0.000      1.549
-----------------------------------------------------------AGAAGGACATCGGCGACATG--------------------------------------------------------------------------------------------------------------------------------------------------------      1.836      2.801     15.166
-----------------------------------------------------------AGAAGGACATCGGCGACA----------------------------------------------------------------------------------------------------------------------------------------------------------      0.147      0.300      0.245
-----------------------------------------------------------AGAAGGACATCGGCGACATGC-------------------------------------------------------------------------------------------------------------------------------------------------------     16.446     18.704     68.492
----------------------------------------------------------CAGAAGGACATCGGCGACATGC-------------------------------------------------------------------------------------------------------------------------------------------------------      0.367      0.500      2.609
----------------------------------------------------------CAGAAGGACATCGGCGACA----------------------------------------------------------------------------------------------------------------------------------------------------------      0.073      0.000      0.163
----------------------------------------------------------CAGAAGGACATCGGCGACATG--------------------------------------------------------------------------------------------------------------------------------------------------------      0.808      1.300      6.523
--------------------------------------------------------TGCAGAAGGACATCGGCGACA----------------------------------------------------------------------------------------------------------------------------------------------------------      8.884     23.405     41.748
-------------------------------------------------------ATGCAGAAGGACATCGGCGACA----------------------------------------------------------------------------------------------------------------------------------------------------------      0.220      0.800      0.571
-------------------------------------------------------ATGCAGAAGGACATCGGCGA------------------------------------------------------------------------------------------------------------------------------------------------------------      0.073      0.200      0.082
```

```
-------------------------------------AATGCAGAAGGACATCGGCG--------------------------------------------------------------------------------------------------------------------------------------------------------------     2.129      4.501      6.360
-------------------------------------AATGCAGAAGGACATCGGCGA-------------------------------------------------------------------------------------------------------------------------------------------------------------     0.220      0.500      1.386
------------------------------------AAATGCAGAAGGACATCGGCG--------------------------------------------------------------------------------------------------------------------------------------------------------------     0.514      1.300      1.712
-----------------------------------AAAATGCAGAAGGACATCGGCG--------------------------------------------------------------------------------------------------------------------------------------------------------------     0.220      0.200      0.082
------------------------------AGGGCAAAATGCAGAAGGAC---------------------------------------------------------------------------------------------------------------------------------------------------------------------     0.073      0.100      0.082
------------------------------AGGGCAAAATGCAGAAGGACA--------------------------------------------------------------------------------------------------------------------------------------------------------------------     1.689      7.802      2.935
--------------------------AACCAGGGCAAAATGCAGAAGG-----------------------------------------------------------------------------------------------------------------------------------------------------------------------     0.147      1.700      1.223
--------------------------AACCAGGGCAAAATGCAGAAG------------------------------------------------------------------------------------------------------------------------------------------------------------------------     0.073      1.300      0.489
-----------------------ACGAACCAGGGCAAAATGCAG---------------------------------------------------------------------------------------------------------------------------------------------------------------------------     0.073      0.200      0.326
---------------------TCACGAACCAGGGCAAAAT-------------------------------------------------------------------------------------------------------------------------------------------------------------------------------     0.073      0.000      0.000
---------------------TCACGAACCAGGGCAAAATGC-----------------------------------------------------------------------------------------------------------------------------------------------------------------------------     0.808      0.800      2.691 miR_t38
---------------------TCACGAACCAGGGCAAAATG------------------------------------------------------------------------------------------------------------------------------------------------------------------------------     0.147      0.100      0.163
--------------------CTCACGAACCAGGGCAAAATG------------------------------------------------------------------------------------------------------------------------------------------------------------------------------     0.073      0.100      0.000
---------------AGGTGCTCACGAACCAGGGCA-----------------------------------------------------------------------------------------------------------------------------------------------------------------------------------     0.073      0.100      0.163

-----------                                               C        G  C     A                 G     G                               C A            T                  CC            C                A       TACA  ACT
           AACAAGGTGCTCACGAACCAGGGCAAAATGCAGAAGGACATCGGCGA ATGCAGAA GA ATCTC CAGCTGAAGCAGGACAC GCAGT AACAAGGCGTTGGTCGCTGTCAACCTGACGC G TGGCGTCACTGG GCTGGGAGTGCTGTACTT  TGTACACTAACT TAAGGTCTTGGGCGGC GCTGAAG    GC   C
           TTGTTCCACGAGTGCTTGGTCCCGTTTTACGTCTTCCTGTAGCCGCT TACGTCTT TT TAGAG GTCGACTTCGTCCTGTG CGTCA TTGTTCCGCAACCAGCGACAGTTGGACTGCG C ACCGCAGTGACC CGACCCTCGCGACATGAA  ACATGTGGTTGA GTTCCAGAGCCCGCCG CGACTTC    CG   /
GGGTGTTCGAG                                               A        A  A     C                 A     G                               A C            T                  AC            A                C       ----  AAA

-----GTTCGAGTTGTTCCACGAGT----------------------------------------------------------------------------------------------------------------------------------------------------------------------------------------------     0.073      0.000      0.000
-----------------CCACGAGTGCTTGGTCCCGTT---------------------------------------------------------------------------------------------------------------------------------------------------------------------------------     0.147      0.300      0.326
-------------------ACGAGTGCTTGGTCCCGTTTT-------------------------------------------------------------------------------------------------------------------------------------------------------------------------------     0.294      0.300      0.571
--------------------CGAGTGCTTGGTCCCGTTTTA------------------------------------------------------------------------------------------------------------------------------------------------------------------------------     0.073      0.100      0.163 miR_t38*
-----------------------GTGCTTGGTCCCGTTTTACGT---------------------------------------------------------------------------------------------------------------------------------------------------------------------------     0.073      0.000      0.000
----------------------------------------ACGTCTTCCTGTAGCCGCT------------------------------------------------------------------------------------------------------------------------------------------------------------     0.073      0.000      0.000
--------------------------------------TTACGTCTTCCTGTAGCCGCT------------------------------------------------------------------------------------------------------------------------------------------------------------     0.294      0.300      0.000
----------------------------------------ACGTCTTCCTGTAGCCGCTATA---------------------------------------------------------------------------------------------------------------------------------------------------------     0.073      0.000      0.000
--------------------------------------------CTTCCTGTAGCCGCTATACGT------------------------------------------------------------------------------------------------------------------------------------------------------     0.147      1.200      0.897
--------------------------------------------------GTAGCCGCTATACGTCTTATT------------------------------------------------------------------------------------------------------------------------------------------------     0.147      0.200      0.326
-----------------------------------------------------------ATACGTCTTATTATAGAGCGT---------------------------------------------------------------------------------------------------------------------------------------     0.073      0.000      0.082
-----------------------------------------------------------------------------------CTTCGTCCTGTGACGTCAGT----------------------------------------------------------------------------------------------------------------     0.073      0.000      0.000
----------------------------------------------------------------------------------ACTTCGTCCTGTGACGTCAGT----------------------------------------------------------------------------------------------------------------     0.073      0.100      0.000
-----------------------------------------------------------------------------------CTTCGTCCTGTGACGTCAGTT---------------------------------------------------------------------------------------------------------------     0.367      0.600      1.223
-----------------------------------------------------------------------------------------CCTGTGACGTCAGTTGTTCCG---------------------------------------------------------------------------------------------------------     0.073      0.000      0.000
-------------------------------------------------------------------------------------------TGTGACGTCAGTTGTTCCGCA-------------------------------------------------------------------------------------------------------     0.147      0.000      0.082
--------------------------------------------------------------------------------------------GTGACGTCAGTTGTTCCGCAA------------------------------------------------------------------------------------------------------     3.304      4.001      5.463
-------------------------------------------------------------------------------------------TGTGACGTCAGTTGTTCCGCAA------------------------------------------------------------------------------------------------------     0.220      0.100      0.571
-----------------------------------------------------------------------------------------------------GTTGTTCCGCAACCAGCGAC----------------------------------------------------------------------------------------------     0.073      0.000      0.000
-----------------------------------------------------------------------------------------------------GTTGTTCCGCAACCAGCGACA---------------------------------------------------------------------------------------------     0.661      3.801      1.957
--------------------------------------------------------------------------------------------------TCAGTTGTTCCGCAACCAGCGACA---------------------------------------------------------------------------------------------     0.147      0.000      0.000
-------------------------------------------------------------------------------------------------------TGTTCCGCAACCAGCGACA---------------------------------------------------------------------------------------------     0.073      0.000      0.000
-----------------------------------------------------------------------------------------------------------CCGCAACCAGCGACAGTT------------------------------------------------------------------------------------------     0.073      0.000      0.000
--------------------------------------------------------------------------------------------------------GTTCCGCAACCAGCGACAGTT------------------------------------------------------------------------------------------     0.073      0.300      0.815
-----------------------------------------------------------------------------------------------------------CCGCAACCAGCGACAGTTGGA---------------------------------------------------------------------------------------     0.073      0.200      0.082
----------------------------------------------------------------------------------------------------------------------------------------ACCGCAGTGACCTCGACCCT-----------------------------------------------------------     0.073      0.000      0.163
---------------------------------------------------------------------------------------------------------------------------------------CACCGCAGTGACCTCGACCCT-----------------------------------------------------------     0.073      0.000      0.000
------------------------------------------------------------------------------------------------------------------------------------------------GACCTCGACCCTCGCGACAT---------------------------------------------------     0.147      0.700      0.082
-----------------------------------------------------------------------------------------------------------------------------------------------TGACCTCGACCCTCGCGACAT---------------------------------------------------     0.147      1.300      0.163
-------------------------------------------------------------------------------------------------------------------------------------------------ACCTCGACCCTCGCGACATGAA------------------------------------------------     0.073      0.000      0.245
--------------------------------------------------------------------------------------------------------------------------------------------------CCTCGACCCTCGCGACATGAA------------------------------------------------     1.909      4.001      7.338
------------------------------------------------------------------------------------------------------------------------------------------------------------------ATGAAACACATGTGGTTGAAGTT------------------------------     0.073      0.000      0.000
-------------------------------------------------------------------------------------------------------------------------------------------------------------------TGAAACACATGTGGTTGAAGTT------------------------------     0.147      0.200      0.000
--------------------------------------------------------------------------------------------------------------------------------------------------------------------GAAACACATGTGGTTGAAGTT------------------------------     0.220      0.100      0.245
----------------------------------------------------------------------------------------------------------------------------------------------------------------------------------CATGTGGTTGAAGTTCCAGA-------------------------     0.073      0.000      0.000
-----------------------------------------------------------------------------------------------------------------------------------------------------------------------------------ACATGTGGTTGAAGTTCCAGA-------------------------     0.147      0.000      0.000

miRNA: miR_t112,miR_t56,miR_t4 chromosome_5 Start: 3227662 End: 3227766 Condition: Nutrient-replete
------------------ACACTTTTCACGCCTCTCG--------------------     0.220      0.000      0.000
-----------------CACACTTTTCACGCCTCTC---------------------     0.073      0.000      0.000
----------------CCACACTTTTCACGCCTCTC---------------------     0.147      0.000      0.000
----------------CCACACTTTTCACGCCTCTCG--------------------     0.147      0.000      0.000
---------------TCCACACTTTTCACGCCTC-----------------------     0.147      0.000      0.000
---------------TCCACACTTTTCACGCCTCTC---------------------     1.909      0.200      0.489
--------------TTCCACACTTTTCACGCCTCTCG--------------------     0.734      0.000      0.082
--------------TTCCACACTTTTCACGCCTC-----------------------     1.175      0.300      1.060
--------------TTCCACACTTTTCACGCCTCTC---------------------     1.468      0.600      0.652
--------------TTCCACACTTTTCACGCCTCT----------------------    16.740      8.602      4.403 miR_t112
--------------TTCCACACTTTTCACGCCT------------------------     0.147      0.000      0.000
--------------TTCCACACTTTTCACGCC-------------------------     0.514      0.100      0.082
-------------ATTCCACACTTTTCACGCCT------------------------     0.147      0.100      0.000
-------------ATTCCACACTTTTCACGCCTC-----------------------     3.010      0.400      1.223
-------------ATTCCACACTTTTCACGCCTCT----------------------     0.220      0.400      0.245
-------------ATTCCACACTTTTCACGCC-------------------------     0.073      0.000      0.000

---------                                            AGT
         GCCCATTCCACACTTTTCACGCCTCTCGCCCAGAAGGCAGCAGC   C
         CGGGTAAGGTGTGAAAAGTGCGGAGAGCGGGTCTTCCGTCGTCG   /
TCGCCACGG                                            CCC

------ACGGCGGGTAAGGTGTGAA--------------------------------     0.073      0.100      0.000
-----CACGGCGGGTAAGGTGTGAAA-------------------------------     1.322      0.200      0.489
------ACGGCGGGTAAGGTGTGAAA-------------------------------     0.954      0.200      0.815
-------CGGCGGGTAAGGTGTGAAA-------------------------------     0.220      0.200      0.082
-------CGGCGGGTAAGGTGTGAAAA------------------------------     1.909      0.300      0.815
------ACGGCGGGTAAGGTGTGAAAA------------------------------     1.615      0.700      0.978
--------GGCGGGTAAGGTGTGAAAA------------------------------     0.147      0.100      0.326
-------CGGCGGGTAAGGTGTGAAAAG-----------------------------     0.147      0.000      0.082
------ACGGCGGGTAAGGTGTGAAAAG-----------------------------     0.367      0.000      0.082
--------GGCGGGTAAGGTGTGAAAAG-----------------------------     0.220      0.100      0.000
---------GCGGGTAAGGTGTGAAAAGT----------------------------     0.147      0.100      0.163
--------GGCGGGTAAGGTGTGAAAAGT----------------------------    15.712     11.302     15.085 miR_t56
-------CGGCGGGTAAGGTGTGAAAAGT----------------------------     0.367      0.500      0.489
-----------GGGTAAGGTGTGAAAAGT----------------------------     0.220      0.000      0.082
------------GGTAAGGTGTGAAAAGTGC--------------------------     0.073      0.000      0.000
------------GGTAAGGTGTGAAAAGTGCG-------------------------     0.367      0.400      0.245
--------------TAAGGTGTGAAAAGTGCGG------------------------     0.367      0.700      0.571
---------------AAGGTGTGAAAAGTGCGG------------------------     1.542      0.900      0.408
-------------GTAAGGTGTGAAAAGTGCGG------------------------     0.220      0.100      0.082
------------GGTAAGGTGTGAAAAGTGCGG------------------------     7.709      3.801      1.794
---------------AAGGTGTGAAAAGTGCGGA-----------------------    18.135     12.603     11.578
----------------AGGTGTGAAAAGTGCGGA-----------------------     0.954      2.000      1.549
------------GGTAAGGTGTGAAAAGTGCGGA-----------------------     8.443      2.100      4.566
-----------GGGTAAGGTGTGAAAAGTGCGGA-----------------------     0.073      0.000      0.000
-------------GTAAGGTGTGAAAAGTGCGGA-----------------------   151.026    266.654    244.942 miR_t112*
--------------TAAGGTGTGAAAAGTGCGGA-----------------------    41.923     80.616     48.108
---------------AAGGTGTGAAAAGTGCGGAG----------------------     3.230      1.200      1.957
--------------TAAGGTGTGAAAAGTGCGGAG----------------------     6.461      3.801      3.180
----------------AGGTGTGAAAAGTGCGGAG----------------------     0.367      0.900      0.163
-------------GTAAGGTGTGAAAAGTGCGGAGA---------------------     0.073      0.000      0.408
---------------AAGGTGTGAAAAGTGCGGAGA---------------------  1669.873    770.556   1259.199 miR_t4
----------------AGGTGTGAAAAGTGCGGAGA---------------------    16.813      7.401      7.991
--------------TAAGGTGTGAAAAGTGCGGAGA---------------------   125.475     66.313    109.914
-----------------GGTGTGAAAAGTGCGGAGA---------------------    16.373      4.301      5.218
------------------GTGTGAAAAGTGCGGAGA---------------------     1.615      0.200      0.652
-------------------TGTGAAAAGTGCGGAGAGCGGGT---------------     0.294      0.000      0.000
------------------GTGTGAAAAGTGCGGAGAGCGGGT---------------     0.073      0.000      0.000
------------------------AAAGTGCGGAGAGCGGGT---------------     0.073      0.000      0.000
--------------------GTGAAAAGTGCGGAGAGCGGGT---------------     0.661      0.300      0.978
---------------------TGAAAAGTGCGGAGAGCGGGT---------------     1.689      0.800      1.468
----------------------GAAAAGTGCGGAGAGCGGGT---------------     1.248      0.300      0.652
----------------------GAAAAGTGCGGAGAGCGGGTCTT------------     0.220      0.100      0.000
-----------------------AAAAGTGCGGAGAGCGGGTCTT------------     0.073      0.000      0.000
--------------------------AGTGCGGAGAGCGGGTCTT------------     0.073      0.000      0.000
------------------------AAAGTGCGGAGAGCGGGTCTT------------     0.441      0.000      0.082

miRNA: miR_t18,miR_t40,miR_t72,miR_t42 chromosome_14 Start: 1950055 End: 1950359 Condition: Nutrient-replete
---------------------------------------------------------------------------------------------------------------------------------------TAGGCGCGGAGCTCATCAAGC----------     0.147      0.200      0.571
-------------------------------------------------------------------------------------------------------------------------------TCGCCTCGTAGGCGCGGAGC-------------------     1.101      0.600      0.082
-------------------------------------------------------------------------------------------------------------------------------TCGCCTCGTAGGCGCGGAGCT------------------     0.220      0.000      0.082
-------------------------------------------------------------------------------------------------------------------------------TCGCCTCGTAGGCGCGGAG--------------------     0.147      0.100      0.163
------------------------------------------------------------------------------------------------------------------------------ATCGCCTCGTAGGCGCGGAG--------------------     0.954      0.500      1.386
------------------------------------------------------------------------------------------------------------------------------ATCGCCTCGTAGGCGCGGAGC-------------------     0.294      0.900      0.489
------------------------------------------------------------------------------------------------------------------------------ATCGCCTCGTAGGCGCGGAGCT------------------     0.073      0.000      0.000
-----------------------------------------------------------------------------------------------------------------------------CATCGCCTCGTAGGCGCGG----------------------     0.073      0.000      0.000
-----------------------------------------------------------------------------------------------------------------------------CATCGCCTCGTAGGCGCGGAG--------------------     0.073      0.000      0.082
----------------------------------------------------------------------------------------------------------------------------CCATCGCCTCGTAGGCGCGGA---------------------     0.147      0.000      0.000
---------------------------------------------------------------------------------------------------------------------------TCCATCGCCTCGTAGGCGCGGA---------------------     0.073      0.000      0.082
---------------------------------------------------------------------------------------------------------------------------TCCATCGCCTCGTAGGCGCGG----------------------     2.717      3.101      1.468
---------------------------------------------------------------------------------------------------------------------------TCCATCGCCTCGTAGGCGCG-----------------------     0.220      0.100      0.245
-------------------------------------------------------------------------------------------------------------------------TGTCCATCGCCTCGTAGGCGCGG----------------------     0.073      0.000      0.000
-------------------------------------------------------------------------------------------------------------------------TGTCCATCGCCTCGTAGGCGCG-----------------------     0.073      0.000      0.000
-------------------------------------------------------------------------------------------------------------------------TGTCCATCGCCTCGTAGGCGC------------------------     0.514      0.700      0.815
------------------------------------------------------------------------------------------------------------------------ATGTCCATCGCCTCGTAGGCGCG-----------------------     0.220      0.000      0.000
------------------------------------------------------------------------------------------------------------------------ATGTCCATCGCCTCGTAGGCGC------------------------     1.248      1.000      0.978
---------------------------------------------------------------------------------------------------------------------TCGATGTCCATCGCCTCGTAGG---------------------------     0.147      0.400      0.082
---------------------------------------------------------------------------------------------------------------------TCGATGTCCATCGCCTCGTAG----------------------------     0.073      0.100      0.000
-------------------------------------------------------------------------------------------------------------------TCTCGATGTCCATCGCCTCGT------------------------------     0.073      0.000      0.000
------------------------------------------------------------------------------------------------------------------ATCTCGATGTCCATCGCCTCG-------------------------------     0.147      0.200      0.000
---------------------------------------------------------------------------------------------------------TGCAGGGGCATCTCGATG-------------------------------------------     0.073      0.100      0.245
---------------------------------------------------------------------------------------------------------TGCAGGGGCATCTCGATGTCC----------------------------------------     0.147      0.100      0.082
--------------------------------------------------------------------------------------------------------CTGCAGGGGCATCTCGATG-------------------------------------------     0.808      0.900      0.082
-------------------------------------------------------------------------------------------------------ACTGCAGGGGCATCTCGATG-------------------------------------------     8.590      9.002     10.111
-------------------------------------------------------------------------------------------------------ACTGCAGGGGCATCTCGA---------------------------------------------     0.367      0.800      0.326
-------------------------------------------------------------------------------------------------------ACTGCAGGGGCATCTCGATGT------------------------------------------     0.294      0.200      0.000 miR_t42*
------------------------------------------------------------------------------------------------------TACTGCAGGGGCATCTCGATG-------------------------------------------  1673.176   2392.583   2450.640 miR_t18
------------------------------------------------------------------------------------------------------TACTGCAGGGGCATCTCGAT--------------------------------------------    11.454     20.604     48.108
------------------------------------------------------------------------------------------------------TACTGCAGGGGCATCTCGA---------------------------------------------    92.289    183.837    156.880
------------------------------------------------------------------------------------------------------TACTGCAGGGGCATCTCGATGT------------------------------------------    36.930     36.007     45.825
------------------------------------------------------------------------------------------------------TACTGCAGGGGCATCTCG----------------------------------------------     2.790      6.001      4.729
------------------------------------------------------------------------------------------------------TACTGCAGGGGCATCTCGATGTC-----------------------------------------     0.073      0.500      0.571
-----------------------------------------------------------------------------------------------------GTACTGCAGGGGCATCTCGA---------------------------------------------     0.073      0.000      0.163
-----------------------------------------------------------------------------------------------------GTACTGCAGGGGCATCTCGAT--------------------------------------------     0.220      0.200      0.082
----------------------------------------------------------------------------------------------------GGTACTGCAGGGGCATCTCGA---------------------------------------------     0.734      0.500      1.468
---------------------------------------------------------------------------------------------------AGGTACTGCAGGGGCATCTCGA---------------------------------------------     1.248      1.400      2.772
---------------------------------------------------------------------------------------------------AGGTACTGCAGGGGCATCTC-----------------------------------------------     0.073      0.100      0.163
```

```
---------------------------------------------------------------------------------------------------AGGTACTGCAGGGGCATCTCGAT--------------------------------------------      0.147      0.000      0.000
---------------------------------------------------------------------------------------------------AGGTACTGCAGGGGCATCTCG----------------------------------------------      3.304      5.601      4.974
---------------------------------------------------------------------------------------------------AGGTACTGCAGGGGCATC-------------------------------------------------      0.073      0.200      0.245
-----------------------------------------------------------------------------------------------GCGCAGGTACTGCAGGGGCAT--------------------------------------------------      0.073      0.000      0.000
-----------------------------------------------------------------------------------------------GCGCAGGTACTGCAGGGGCA---------------------------------------------------      0.073      0.000      0.000
---------------------------------------------------------------------------------------------CCGCGCAGGTACTGCAGGGGC----------------------------------------------------      0.147      0.000      0.082
---------------------------------------------------------------------------------------TTCTCGCCGCGCAGGTACTGC----------------------------------------------------------      0.147      0.400      0.245
------------------------------------------------------------------------------------ACGTTCTCGCCGCGCAGG----------------------------------------------------------------      0.073      0.200      0.163
------------------------------------------------------------------------------------ACGTTCTCGCCGCGCAGGTAC-------------------------------------------------------------      0.147      0.000      0.082
---------------------------------------------------------------------------------TCCACGTTCTCGCCGCGCA------------------------------------------------------------------      0.073      0.000      0.000
---------------------------------------------------------------------------------TCCACGTTCTCGCCGCGCAGGT---------------------------------------------------------------      0.514      0.100      0.082
---------------------------------------------------------------------------------TCCACGTTCTCGCCGCGCAGG----------------------------------------------------------------     15.125     15.203      6.768 miR_t40
------------------------------------------------------------------------------TTCTCCACGTTCTCGCCGCGCA------------------------------------------------------------------      0.073      0.100      0.082
------------------------------------------------------------------------------TTCTCCACGTTCTCGCCGCGC-------------------------------------------------------------------      0.073      0.000      0.163
---------------------------------------------------------------------------ACCTTCTCCACGTTCTCGCCG----------------------------------------------------------------------      0.147      0.100      0.163
--------------------------------------------------------------TACATTCTCCGTCACCTTCTCC----------------------------------------------------------------------------------      0.367      0.000      0.408
--------------------------------------------------------------TACATTCTCCGTCACCTTCT------------------------------------------------------------------------------------      1.689      0.700      0.897
--------------------------------------------------------------TACATTCTCCGTCACCTTC-------------------------------------------------------------------------------------      0.441      0.300      0.571
--------------------------------------------------------------TACATTCTCCGTCACCTTCTCCA---------------------------------------------------------------------------------      0.220      0.000      0.000
-------------------------------------------------------------TTACATTCTCCGTCACCT---------------------------------------------------------------------------------------      0.073      0.000      0.000
-------------------------------------------------------------TTACATTCTCCGTCACCTTCT------------------------------------------------------------------------------------      0.073      0.200      0.163
-------------------------------------------------------------TTACATTCTCCGTCACCTTC-------------------------------------------------------------------------------------      0.220      0.400      0.408
---------------------------------------------------------TACCTTACATTCTCCGTCACCTTC-------------------------------------------------------------------------------------      0.073      0.000      0.000
-------------------------------------------------TACCCACTTACCTTACATTCTCC----------------------------------------------------------------------------------------------      0.147      0.000      0.245
----------------------------------------AACGGGAAGTACCCACTTACCTT-------------------------------------------------------------------------------------------------------      0.073      0.000      0.000
----------------------------------------AACGGGAAGTACCCACTTACC---------------------------------------------------------------------------------------------------------      0.367      0.100      0.326
----------------------------------------AACGGGAAGTACCCACTTACCT--------------------------------------------------------------------------------------------------------      2.570      0.900      2.935
----------------------------------------AACGGGAAGTACCCACTTAC----------------------------------------------------------------------------------------------------------      0.073      0.000      0.000
-----------------------------AAGTTATCAGGAACGGGAAG---------------------------------------------------------------------------------------------------------------------      0.147      0.100      0.245
-----------------------------AAGTTATCAGGAACGGGAAGT--------------------------------------------------------------------------------------------------------------------      0.147      0.000      0.245
----------------------------GAAGTTATCAGGAACGGGAAG---------------------------------------------------------------------------------------------------------------------      0.073      0.300      0.082
---------------------------CGAAGTTATCAGGAACGGGAAG---------------------------------------------------------------------------------------------------------------------      0.294      0.300      0.245
----ACGACGGGAAAAGGCCCGCCCTT-------------------------------------------------------------------------------------------------------------------------------------------      0.073      0.000      0.082

TGTAACGACGGGAAAAGGCCCGCCCTTC      A                   A  TACC                      A                                                                             C TC
                            GAAGTT TCAGGAACGGGAAGTACCC CT    TTACATTCTCCGTCACCTTCTCC CGTTCTCGCCGCGCAGGTACTGCAGGGGCATCTCGATGTCCATCGCCTCGTAGGCGCGGAGCTCATCAAGCGCCTG G  T
                            CTTCAA GGTCCTTGCCCTTCATGGG GG    AATGTAAGAGGCGGTGGAAGAGG GCAAGAGCGGCGCGTCCATGACGTCCCCGTAGAGCTACAGGTAGCGGAGCATCCGCGCCTCGAGTAGTTCGCGGAC C  T
--------------------------AT      C                   C  CGC-                      C                                                                             - TT

-------------------------------TCAACGGTCCTTGCCCTTCAT------------------------------------------------------------------------------------------------------------------      0.073      0.000      0.000
--------------------------------------------CCCTTCATGGGCGGCG-CAAT-----------------------------------------------------------------------------------------------------      0.073      0.000      0.000
-------------------------------------------------CATGGGCGGCG-CAATGTAA-------------------------------------------------------------------------------------------------      0.073      0.000      0.000
------------------------------------------------TCATGGGCGGCG-CAATGTAAGA-----------------------------------------------------------------------------------------------      1.909      3.501      3.180
-------------------------------------------------CATGGGCGGCG-CAATGTAAGA-----------------------------------------------------------------------------------------------      0.587      0.000      0.245
--------------------------------------------------------GGCG-CAATGTAAGAGGCGGT-----------------------------------------------------------------------------------------      0.147      0.200      0.000
-----------------------------------------------------------G-CAATGTAAGAGGCGGTGGA--------------------------------------------------------------------------------------      0.881      4.801      1.875
---------------------------------------------------------GCG-CAATGTAAGAGGCGGTGGA--------------------------------------------------------------------------------------      0.073      0.000      0.082
----------------------------------------------------------CG-CAATGTAAGAGGCGGTGGA--------------------------------------------------------------------------------------      1.322      4.401      3.588
-------------------------------------------------------------CAATGTAAGAGGCGGTGGA--------------------------------------------------------------------------------------      1.322      0.900      0.978
--------------------------------------------------------------AATGTAAGAGGCGGTGGA--------------------------------------------------------------------------------------      0.514      1.000      0.326
-------------------------------------------------------------CAATGTAAGAGGCGGTGGAA-------------------------------------------------------------------------------------      2.056     12.202      5.055
--------------------------------------------------------------AATGTAAGAGGCGGTGGAA-------------------------------------------------------------------------------------      0.073      0.200      0.326
-----------------------------------------------------------G-CAATGTAAGAGGCGGTGGAA-------------------------------------------------------------------------------------      0.073      0.400      0.245
--------------------------------------------------------------AATGTAAGAGGCGGTGGAAG------------------------------------------------------------------------------------      0.294      0.000      0.245
-------------------------------------------------------------CAATGTAAGAGGCGGTGGAAG------------------------------------------------------------------------------------      0.073      0.200      0.082
---------------------------------------------------------------ATGTAAGAGGCGGTGGAAGA-----------------------------------------------------------------------------------      0.514      1.900      0.326
----------------------------------------------------------------TGTAAGAGGCGGTGGAAGA-----------------------------------------------------------------------------------      0.147      0.600      0.489
--------------------------------------------------------------AATGTAAGAGGCGGTGGAAGA-----------------------------------------------------------------------------------     88.545    104.921    118.965
-----------------------------------------------------------------GTAAGAGGCGGTGGAAGA-----------------------------------------------------------------------------------      0.147      0.200      0.489
-------------------------------------------------------------CAATGTAAGAGGCGGTGGAAGA-----------------------------------------------------------------------------------     55.653     57.712     55.283
-------------------------------------------------------------------AAGAGGCGGTGGAAGAGGCGC------------------------------------------------------------------------------      0.073      0.000      0.000
---------------------------------------------------------------------GAGGCGGTGGAAGAGGCGCA-----------------------------------------------------------------------------      0.073      0.200      0.245
--------------------------------------------------------------------AGAGGCGGTGGAAGAGGCGCA-----------------------------------------------------------------------------      0.220      0.000      0.000
----------------------------------------------------------------------AGGCGGTGGAAGAGGCGCAA----------------------------------------------------------------------------      0.220      0.200      0.245
---------------------------------------------------------------------GAGGCGGTGGAAGAGGCGCAA----------------------------------------------------------------------------      0.073      0.400      0.163
-----------------------------------------------------------------------GGCGGTGGAAGAGGCGCAA----------------------------------------------------------------------------      0.073      0.000      0.082
----------------------------------------------------------------------AGGCGGTGGAAGAGGCGCAAG---------------------------------------------------------------------------      0.073      0.000      0.245
------------------------------------------------------------------------GCGGTGGAAGAGGCGCAAGA--------------------------------------------------------------------------      0.073      0.000      0.163
-----------------------------------------------------------------------GGCGGTGGAAGAGGCGCAAGA--------------------------------------------------------------------------     35.242     23.805     29.517
-------------------------------------------------------------------------CGGTGGAAGAGGCGCAAGA--------------------------------------------------------------------------      0.147      0.000      0.000
----------------------------------------------------------------------AGGCGGTGGAAGAGGCGCAAGA--------------------------------------------------------------------------      9.838      4.801     10.600
-------------------------------------------------------------------------CGGTGGAAGAGGCGCAAGAGC------------------------------------------------------------------------      0.073      0.000      0.000
---------------------------------------------------------------------------GTGGAAGAGGCGCAAGAGCG-----------------------------------------------------------------------      0.073      0.100      0.000
--------------------------------------------------------------------------GGTGGAAGAGGCGCAAGAGCG-----------------------------------------------------------------------      0.441      0.200      0.245
-----------------------------------------------------------------------------GGAAGAGGCGCAAGAGCGGC---------------------------------------------------------------------      0.073      0.600      0.000
------------------------------------------------------------------------------GAAGAGGCGCAAGAGCGGCG--------------------------------------------------------------------      0.147      0.400      0.082
--------------------------------------------------------------------------------AGAGGCGCAAGAGCGGCG--------------------------------------------------------------------      4.552     11.602      4.158
-----------------------------------------------------------------------------GGAAGAGGCGCAAGAGCGGCG--------------------------------------------------------------------      4.479     13.903      5.789
----------------------------------------------------------------------------TGGAAGAGGCGCAAGAGCGGCG--------------------------------------------------------------------      0.881      2.200      0.978
-------------------------------------------------------------------------------AAGAGGCGCAAGAGCGGCG--------------------------------------------------------------------      0.367      0.500      0.082
---------------------------------------------------------------------------------GAGGCGCAAGAGCGGCGC-------------------------------------------------------------------      0.147      0.100      0.000
--------------------------------------------------------------------------------AGAGGCGCAAGAGCGGCGC-------------------------------------------------------------------      1.175      1.400      0.815
-----------------------------------------------------------------------------GGAAGAGGCGCAAGAGCGGCGC-------------------------------------------------------------------      0.367      1.200      0.326
----------------------------------------------------------------------------TGGAAGAGGCGCAAGAGCGGCGC-------------------------------------------------------------------      0.073      0.100      0.082
--------------------------------------------------------------------------------AGAGGCGCAAGAGCGGCGCG------------------------------------------------------------------      7.636     10.802      7.257
---------------------------------------------------------------------------------GAGGCGCAAGAGCGGCGCG------------------------------------------------------------------      0.441      0.200      0.163
----------------------------------------------------------------------------------AGGCGCAAGAGCGGCGCGT-----------------------------------------------------------------      4.185     25.605      7.012
-------------------------------------------------------------------------------AAGAGGCGCAAGAGCGGCGCGT-----------------------------------------------------------------      3.451      1.800      2.202
--------------------------------------------------------------------------------AGAGGCGCAAGAGCGGCGCGT-----------------------------------------------------------------   2509.801   2028.510   2230.649 miR_t72
---------------------------------------------------------------------------------GAGGCGCAAGAGCGGCGCGT-----------------------------------------------------------------     56.754     53.011     64.742
-----------------------------------------------------------------------------------GGCGCAAGAGCGGCGCGT-----------------------------------------------------------------      3.965      4.101      1.875
---------------------------------------------------------------------------------GAGGCGCAAGAGCGGCGCGTC----------------------------------------------------------------      0.147      0.000      0.000
--------------------------------------------------------------------------------AGAGGCGCAAGAGCGGCGCGTC----------------------------------------------------------------      2.643      1.200      1.060
-----------------------------------------------------------------------------------GGCGCAAGAGCGGCGCGTC----------------------------------------------------------------      0.220      0.100      0.082
----------------------------------------------------------------------------------AGGCGCAAGAGCGGCGCGTCC---------------------------------------------------------------      0.734      0.500      0.652
-----------------------------------------------------------------------------------GGCGCAAGAGCGGCGCGTCC---------------------------------------------------------------      0.808      0.600      0.082
-----------------------------------------------------------------------------------GGCGCAAGAGCGGCGCGTCCA--------------------------------------------------------------     77.165     18.904     15.737
----------------------------------------------------------------------------------AGGCGCAAGAGCGGCGCGTCCA--------------------------------------------------------------      3.744      0.500      0.245
--------------------------------------------------------------------------------------GCAAGAGCGGCGCGTCCA--------------------------------------------------------------      0.147      0.100      0.000
-----------------------------------------------------------------------------------GGCGCAAGAGCGGCGCGTCCAT-------------------------------------------------------------     17.694     18.704     13.698
----------------------------------------------------------------------------------AGGCGCAAGAGCGGCGCGTCCAT-------------------------------------------------------------      0.220      0.200      0.000
------------------------------------------------------------------------------------GCGCAAGAGCGGCGCGTCCAT-------------------------------------------------------------      0.661      1.100      0.978
-------------------------------------------------------------------------------------CGCAAGAGCGGCGCGTCCATG------------------------------------------------------------      0.073      0.000      0.000
---------------------------------------------------------------------------------------CAAGAGCGGCGCGTCCATGA-----------------------------------------------------------      0.073      0.100      0.000
------------------------------------------------------------------------------------------GAGCGGCGCGTCCATGACGT--------------------------------------------------------      0.073      0.000      0.000
-----------------------------------------------------------------------------------------AGAGCGGCGCGTCCATGACGT--------------------------------------------------------      0.441      0.400      0.000
-------------------------------------------------------------------------------------------AGCGGCGCGTCCATGACGTCCC-----------------------------------------------------      0.073      0.000      0.000
--------------------------------------------------------------------------------------------GCGGCGCGTCCATGACGTCCC-----------------------------------------------------      1.689      1.900      1.142
--------------------------------------------------------------------------------------------GCGGCGCGTCCATGACGTCCCC----------------------------------------------------      0.073      0.000      0.000
----------------------------------------------------------------------------------------------GGCGCGTCCATGACGTCCCCGT--------------------------------------------------      0.073      0.000      0.000
-----------------------------------------------------------------------------------------------GCGCGTCCATGACGTCCCCGT--------------------------------------------------      0.808      0.200      0.245
------------------------------------------------------------------------------------------------CGCGTCCATGACGTCCCCGTA-------------------------------------------------      0.147      0.000      0.082
---------------------------------------------------------------------------------------------------GTCCATGACGTCCCCGTAGA-----------------------------------------------      1.395      0.400      0.000
--------------------------------------------------------------------------------------------------CGTCCATGACGTCCCCGTAGA-----------------------------------------------      0.734      1.000      0.163
--------------------------------------------------------------------------------------------------CGTCCATGACGTCCCCGTAGAG----------------------------------------------      0.147      0.000      0.000
---------------------------------------------------------------------------------------------------GTCCATGACGTCCCCGTAGAG----------------------------------------------      0.587      0.100      0.245
-----------------------------------------------------------------------------------------------------CCATGACGTCCCCGTAGAG----------------------------------------------      0.220      0.400      0.000
----------------------------------------------------------------------------------------------------TCCATGACGTCCCCGTAGAGC---------------------------------------------      0.073      0.000      0.000
-----------------------------------------------------------------------------------------------------CCATGACGTCCCCGTAGAGC---------------------------------------------      0.073      0.000      0.000
----------------------------------------------------------------------------------------------------TCCATGACGTCCCCGTAGAGCT--------------------------------------------      0.734      0.200      0.163
-----------------------------------------------------------------------------------------------------CCATGACGTCCCCGTAGAGCT--------------------------------------------     17.254     16.303     11.089 miR_t42
-------------------------------------------------------------------------------------------------------ATGACGTCCCCGTAGAGCT--------------------------------------------      0.147      0.100      0.000
------------------------------------------------------------------------------------------------------CATGACGTCCCCGTAGAGCT--------------------------------------------      0.220      0.200      0.245
---------------------------------------------------------------------------------------------------------GACGTCCCCGTAGAGCTA-------------------------------------------      0.073      0.000      0.000
-------------------------------------------------------------------------------------------------------ATGACGTCCCCGTAGAGCTAC------------------------------------------      0.073      0.000      0.000
--------------------------------------------------------------------------------------------------------TGACGTCCCCGTAGAGCTACA-----------------------------------------      0.661      2.100      0.897
---------------------------------------------------------------------------------------------------------GACGTCCCCGTAGAGCTACA-----------------------------------------      0.661      0.700      0.326
----------------------------------------------------------------------------------------------------------ACGTCCCCGTAGAGCTACAGGT--------------------------------------      0.073      0.000      0.082
------------------------------------------------------------------------------------------------------------GTCCCCGTAGAGCTACAGGT--------------------------------------      0.147      0.200      0.163
-----------------------------------------------------------------------------------------------------------CGTCCCCGTAGAGCTACAGGT--------------------------------------      1.689      2.400      1.549
------------------------------------------------------------------------------------------------------------GTCCCCGTAGAGCTACAGGTA-------------------------------------      0.073      0.000      0.000
--------------------------------------------------------------------------------------------------------------CCCCGTAGAGCTACAGGTAG------------------------------------      0.073      0.000      0.000
---------------------------------------------------------------------------------------------------------------CCCGTAGAGCTACAGGTAGC-----------------------------------      0.073      0.000      0.000
--------------------------------------------------------------------------------------------------------------CCCCGTAGAGCTACAGGTAGC-----------------------------------      0.073      0.000      0.082
-----------------------------------------------------------------------------------------------------------------CGTAGAGCTACAGGTAGCG----------------------------------      0.073      0.000      0.082
-----------------------------------------------------------------------------------------------------------------CGTAGAGCTACAGGTAGCGG---------------------------------      0.073      0.000      0.082
-----------------------------------------------------------------------------------------------------------------CGTAGAGCTACAGGTAGCGGA--------------------------------      6.755      8.102      9.948
--------------------------------------------------------------------------------------------------------------------AGAGCTACAGGTAGCGGA--------------------------------      0.073      0.200      0.000
------------------------------------------------------------------------------------------------------------------GTAGAGCTACAGGTAGCGGA--------------------------------      0.441      0.100      0.326
--------------------------------------------------------------------------------------------------------------------AGAGCTACAGGTAGCGGAGCAT----------------------------      0.220      1.200      1.712
---------------------------------------------------------------------------------------------------------------------GAGCTACAGGTAGCGGAGCAT----------------------------      0.073      0.000      0.326
--------------------------------------------------------------------------------------------------------------------------ACAGGTAGCGGAGCATCC--------------------------      0.073      1.000      0.245
------------------------------------------------------------------------------------------------------------------------CTACAGGTAGCGGAGCATCCG-------------------------      0.073      0.200      0.082
--------------------------------------------------------------------------------------------------------------------------ACAGGTAGCGGAGCATCCG-------------------------      0.220      6.601      0.652
--------------------------------------------------------------------------------------------------------------------------ACAGGTAGCGGAGCATCCGCG-----------------------     15.932     73.415     27.397
---------------------------------------------------------------------------------------------------------------------------CAGGTAGCGGAGCATCCGCG-----------------------      0.147      2.000      0.978
----------------------------------------------------------------------------------------------------------------------------AGGTAGCGGAGCATCCGCGC----------------------      0.073      0.000      0.082
--------------------------------------------------------------------------------------------------------------------------ACAGGTAGCGGAGCATCCGCGC----------------------      0.220      0.100      0.408
-----------------------------------------------------------------------------------------------------------------------------------------------CCTCGAGTAGTTCGCGGA-----      0.073      0.000      0.000

miRNA: miR_t99,miR_t104 chromosome_4 Start: 3100616 End: 3100763 Condition: Nutrient-replete
--------------------------------------------------------CGGGCATGAAGGTCGATTTG-      0.367      0.200      0.571
--------------------------------------------------------CGGGCATGAAGGTCGATTT--      0.147      0.100      1.142
--------------------------------------------------------CGGGCATGAAGGTCGATT---      0.220      0.200      0.245
-------------------------------------------------------GCGGGCATGAAGGTCGATTT--      0.073      0.600      1.223
-------------------------------------------------------GCGGGCATGAAGGTCGATT---      0.220      0.200      0.163
-------------------------------------------------------GCGGGCATGAAGGTCGATTTG-      0.073      0.100      0.815
-------------------------------------------------------GCGGGCATGAAGGTCGAT----      0.294      0.100      0.571
--------------------------------------------------ACAAGGCGGGCATGAAGGTCG------      0.587      0.600      0.489
--------------------------------------------------ACAAGGCGGGCATGAAGG---------      0.147      0.000      0.000
-------------------------------------------------TACAAGGCGGGCATGAAGGT--------      0.147      0.000      0.000
-------------------------------------------------TACAAGGCGGGCATGAAGG---------      0.587      0.500      0.489
-------------------------------------------------TACAAGGCGGGCATGAAGGTC-------      0.220      0.100      0.489
-------------------------------------------------TACAAGGCGGGCATGAAGGTCG------      8.370      7.602      7.420
-----------------------------------------------ACTACAAGGCGGGCATGAAGG---------      0.220      0.400      0.163
----------------------------------------------AACTACAAGGCGGGCATGAAG----------      0.147      0.000      0.000
```

```
---------------------------------------TGCTAGCAACTACAAGGC--------------------      0.073      0.000      0.000
--------------------------------------CTGCTAGCAACTACAAGG---------------------      0.073      0.100      0.082
-------------------------------------GCTGCTAGCAACTACAAGGCG-------------------      0.147      0.100      0.245
-------------------------------------GCTGCTAGCAACTACAAG----------------------      0.073      0.000      0.000
-------------------------------------GCTGCTAGCAACTACAAGGC--------------------      0.220      0.100      0.082
-------------------------------------GCTGCTAGCAACTACAAGG---------------------      0.367      0.300      0.326
------------------------------------TGCTGCTAGCAACTACAAGGCG-------------------      9.324      7.401     13.780
------------------------------------TGCTGCTAGCAACTACAAGGC--------------------     34.214     17.103     39.872
------------------------------------TGCTGCTAGCAACTACAAGG---------------------     15.345      9.902     23.402
------------------------------------TGCTGCTAGCAACTACAAG----------------------      0.514      0.300      0.734
-----------------------------------GTGCTGCTAGCAACTACAAGG---------------------      0.073      0.000      0.163
-----------------------------------GTGCTGCTAGCAACTACAAG----------------------      0.073      0.000      0.000
----------------------------------AGTGCTGCTAGCAACTAC-------------------------      0.073      0.200      0.163
----------------------------------AGTGCTGCTAGCAACTACAAG----------------------     17.327     30.906     23.809
----------------------------------AGTGCTGCTAGCAACTACAAGGC--------------------      0.073      0.200      0.163
----------------------------------AGTGCTGCTAGCAACTACAAGG---------------------      6.094      8.502      8.480
----------------GGCCATCGTATTACTATCAG-----------------------------------------      0.073      0.100      0.163
----------------GGCCATCGTATTACTATC-------------------------------------------      0.147      0.000      0.000
---------------GGGCCATCGTATTACTATC-------------------------------------------      0.294      0.500      0.734
---------------GGGCCATCGTATTACTATCAG-----------------------------------------      2.496      1.600      4.077
---------------GGGCCATCGTATTACTATCA------------------------------------------      0.147      0.400      0.082
---------------GGGCCATCGTATTACTATCAGT----------------------------------------      0.220      0.000      0.000
--------------TGGGCCATCGTATTACTA---------------------------------------------     11.233      5.901     15.003
--------------TGGGCCATCGTATTACTATCAG-----------------------------------------    795.950    757.953   1295.973 miR_t99
--------------TGGGCCATCGTATTACTATC-------------------------------------------    121.364     60.012    160.549
--------------TGGGCCATCGTATTACTATCAGT----------------------------------------      5.580      2.601      7.420
--------------TGGGCCATCGTATTACTATCA------------------------------------------     34.140     17.103     52.592
--------------TGGGCCATCGTATTACTAT--------------------------------------------      5.580      8.402     15.329
-------------ATGGGCCATCGTATTACTATC-------------------------------------------      0.220      1.200      0.734
------------AATGGGCCATCGTATTACTATCAG-----------------------------------------      0.073      0.100      0.082
------------AATGGGCCATCGTATTACTA---------------------------------------------      0.294      0.200      0.326
-----------AAATGGGCCATCGTATTACTA---------------------------------------------      0.147      0.100      0.082

-------                       T            A    C          G              TG
       GTGCAAATGGGCCATCGTATTAC ATCAGTGCTGCT GCAA TACAAGGCGG CATGAAGGTCGATT  A
       CGCGTTTACTCGGTAGCGTAATG TAGTCACGATGG CGTT ATGTTCCGCC GTACTTCCAGCTAG  C
GGGAAAG                       T            C    A          A              CC

-----AAGCGCGTTTACTCGGTAGCGT--------------------------------------------------      0.073      0.000      0.000
------AGCGCGTTTACTCGGTAGCGT--------------------------------------------------      0.587      0.400      0.815
---------GCGTTTACTCGGTAGCGTAA------------------------------------------------      0.147      0.000      0.000
----------CGTTTACTCGGTAGCGTAAT-----------------------------------------------      0.073      0.000      0.000
-------------TTACTCGGTAGCGTAATG----------------------------------------------      0.147      0.000      0.163
------------TTTACTCGGTAGCGTAATG----------------------------------------------      0.294      0.100      0.245
-------------TTACTCGGTAGCGTAATGT---------------------------------------------      0.294      0.100      0.408
------------TTTACTCGGTAGCGTAATGT---------------------------------------------      1.248      0.500      1.305
--------------TACTCGGTAGCGTAATGT---------------------------------------------      0.073      0.300      0.082
-----------GTTTACTCGGTAGCGTAATGT---------------------------------------------      1.909      1.500      4.158
-----------GTTTACTCGGTAGCGTAATGTT--------------------------------------------      0.734      0.800      2.365
--------------TACTCGGTAGCGTAATGTT--------------------------------------------      3.084      3.501      3.914
------------TTTACTCGGTAGCGTAATGTT--------------------------------------------    149.043     73.815     87.817
---------------ACTCGGTAGCGTAATGTT--------------------------------------------      1.028      1.500      1.223
-------------TTACTCGGTAGCGTAATGTT--------------------------------------------     48.678     36.907     67.840
---------------ACTCGGTAGCGTAATGTTA-------------------------------------------      0.073      0.000      0.000
--------------TACTCGGTAGCGTAATGTTA-------------------------------------------      0.073      0.200      0.000
-------------TTACTCGGTAGCGTAATGTTA-------------------------------------------      0.441      0.400      0.652
---------------ACTCGGTAGCGTAATGTTAG------------------------------------------      0.661      2.200      1.875
----------------CTCGGTAGCGTAATGTTAG------------------------------------------      0.147      0.200      0.245
-------------TTACTCGGTAGCGTAATGTTAG------------------------------------------      0.734      2.801      1.060 miR_t99*
--------------TACTCGGTAGCGTAATGTTAG------------------------------------------     11.013      8.402      6.931
--------------TACTCGGTAGCGTAATGTTAGT-----------------------------------------      2.203      3.401      4.158
------------------CGGTAGCGTAATGTTAGT-----------------------------------------      0.073      0.200      0.489
------------TTTACTCGGTAGCGTAATGTTAGT-----------------------------------------      0.294      0.000      0.000
---------------ACTCGGTAGCGTAATGTTAGT-----------------------------------------      2.643      5.001      3.995
-------------TTACTCGGTAGCGTAATGTTAGT-----------------------------------------      2.937      2.801      5.789
----------------CTCGGTAGCGTAATGTTAGT-----------------------------------------      0.147      0.300      0.163
---------------------------AATGTTAGTCACGATGGCCG------------------------------      0.073      0.100      0.163
---------------------------AATGTTAGTCACGATGGCCGT-----------------------------      4.625      2.000      5.708
--------------------------TAATGTTAGTCACGATGGCCGT-----------------------------      0.147      0.100      0.163
---------------------------AATGTTAGTCACGATGGCCGTTA---------------------------      0.073      0.000      0.000
---------------------------------AGTCACGATGGCCGTTAA--------------------------      0.147      0.000      0.245
-------------------------------TTAGTCACGATGGCCGTTAAT-------------------------      0.073      0.000      0.163
---------------------------------AGTCACGATGGCCGTTAAT-------------------------      0.073      0.300      0.489
---------------------------------AGTCACGATGGCCGTTAATG------------------------     20.778     13.303     29.925
--------------------------------TAGTCACGATGGCCGTTAATG------------------------      2.203      0.100      2.202
----------------------------------GTCACGATGGCCGTTAATG------------------------      0.514      0.200      0.571
-----------------------------------TCACGATGGCCGTTAATG------------------------      0.661      0.400      0.571
-------------------------------TTAGTCACGATGGCCGTTAATG------------------------      0.147      0.100      0.163
-------------------------------TTAGTCACGATGGCCGTTAATGT-----------------------      8.297      3.701     15.166
----------------------------------GTCACGATGGCCGTTAATGT-----------------------      6.461      6.101     29.435
--------------------------------TAGTCACGATGGCCGTTAATGT-----------------------    132.230     67.514    151.254
-----------------------------------TCACGATGGCCGTTAATGT-----------------------      3.451      2.200      6.115
---------------------------------AGTCACGATGGCCGTTAATGT-----------------------   1820.164   1283.459   2982.516 miR_t104
------------------------------------CACGATGGCCGTTAATGT-----------------------      3.230      2.300      4.566
--------------------------------TAGTCACGATGGCCGTTAATGTT----------------------      3.744      0.700      2.528
---------------------------------AGTCACGATGGCCGTTAATGTT----------------------     84.654     53.411    106.245
-------------------------------------ACGATGGCCGTTAATGTT----------------------      0.808      0.400      1.549
----------------------------------GTCACGATGGCCGTTAATGTT----------------------     73.127     66.513    100.292
-----------------------------------TCACGATGGCCGTTAATGTT----------------------    199.116    148.130    255.460
------------------------------------CACGATGGCCGTTAATGTT----------------------      6.755      5.401      9.540
-------------------------------TTAGTCACGATGGCCGTTAATGTT----------------------      0.147      0.000      0.082
----------------------------------GTCACGATGGCCGTTAATGTTC---------------------      0.073      0.000      0.000
-----------------------------------TCACGATGGCCGTTAATGTTC---------------------      0.954      0.700      0.326
-------------------------------------ACGATGGCCGTTAATGTTC---------------------      0.073      0.000      0.082
-------------------------------------ACGATGGCCGTTAATGTTCC--------------------      0.073      0.000      0.000
------------------------------------CACGATGGCCGTTAATGTTCC--------------------      0.147      0.400      0.408
------------------------------------------------------TCCGCCAGTACTTCCAGC-----      0.073      0.000      0.000
------------------------------------------------------TCCGCCAGTACTTCCAGCTA---      0.073      0.000      0.000

miRNA: miR_t124 chromosome_6 Start: 2201613 End: 2201694 Condition: Nutrient-replete
---------------CGGCGTTGTAAGATTGCGGT--------      0.073      0.000      0.000
--------------GCGGCGTTGTAAGATTGCGG---------      0.954      0.000      0.000
--------------GCGGCGTTGTAAGATTGCGGT--------      1.248      0.000      0.000
-------------GGCGGCGTTGTAAGATTGCGGT--------      0.073      0.000      0.000
------------GGGCGGCGTTGTAAGATT-------------     10.132      0.000      0.000
------------GGGCGGCGTTGTAAGATTG------------      0.073      0.000      0.000
-----------TGGGCGGCGTTGTAAGATT-------------      7.636      0.000      0.000
-----------TGGGCGGCGTTGTAAGATTGC-----------      1.101      0.000      0.000
-----------TGGGCGGCGTTGTAAGATTG------------      1.836      0.000      0.000
-----------TGGGCGGCGTTGTAAGAT--------------      0.294      0.000      0.000
----------TTGGGCGGCGTTGTAAGATTGC-----------      0.073      0.000      0.000
----------TTGGGCGGCGTTGTAAGATTG------------     12.188      0.000      0.000
----------TTGGGCGGCGTTGTAAGAT--------------     42.584      0.000      0.082
----------TTGGGCGGCGTTGTAAGA---------------     22.613      0.000      0.000
----------TTGGGCGGCGTTGTAAGATT-------------   2384.473      0.000      0.163 miR_t124
---------GTTGGGCGGCGTTGTAAGATT-------------      0.147      0.000      0.000
---------GTTGGGCGGCGTTGTAAGAT--------------      0.073      0.000      0.000
--------AGTTGGGCGGCGTTGTAAGAT--------------     20.117      0.000      0.000
--------AGTTGGGCGGCGTTGTAAGA---------------      0.294      0.000      0.000
--------AGTTGGGCGGCGTTGTAAGATT-------------      6.681      0.000      0.000
-------GAGTTGGGCGGCGTTGTAAGAT--------------      0.881      0.000      0.000
-------GAGTTGGGCGGCGTTGTAAGATT-------------      0.661      0.000      0.000
-------GAGTTGGGCGGCGTTGTAAGA---------------      0.073      0.000      0.000
----AGCGAGTTGGGCGGCGTTGTA------------------      0.073      0.000      0.000

TGGC                                AAGATG
    AGCGAGTTGGGCGGCGTTGTAAGATTGCGGTA      C
    TCGCTCAACCCGCCGCAACATTCTAACGCCAT      /
----                                GGGGGG

---------TCAACCCGCCGCAACATTC---------------      0.073      0.000      0.000
---------TCAACCCGCCGCAACATTCT--------------      0.661      0.000      0.000 miR_t124*

miRNA: miR_t98,miR_t20 chromosome_6 Start: 6775868 End: 6776242 Condition: Phosphate-deprivation
-----------------------------------------------------------------------------------------------------------------------------------------------------------------------------------------------------------------------------------------------------------------------------------TACCCGAACGTCGAGTTCAAGT---      0.000      0.000      0.082
-----------------------------------------------------------------------------------------------------------------------------------------------------------------------------------------------------------------------------------------------------------------------------------TACCCGAACGTCGAGTTCAAG----      0.808      0.800      0.897
-----------------------------------------------------------------------------------------------------------------------------------------------------------------------------------------------------------------------------------------------------------------------------------TACCCGAACGTCGAGTTCAA-----      0.000      0.000      0.082
--------------------------------------------------------------------------------------------------------------------------------------------------------------------------------------------------------------------------------------------------------------------------------AGCTCTACCCGAACGTCGAG----------      0.000      0.000      0.082
-----------------------------------------------------------------------------------------------------------------------------------------------------------------------------------------------------------------------------------------------------------------------------TCGAGCTCTACCCGAACGTCG------------      0.147      0.200      0.082
----------------------------------------------------------------------------------------------------------------------------------------------------------------------------------------------------------------------------------------------------------------------AAGACCTTCGAGCTCTACC---------------------      0.220      0.000      0.163
----------------------------------------------------------------------------------------------------------------------------------------------------------------------------------------------------------------------------------------------------------------------AAGACCTTCGAGCTCTACCCG-------------------      0.808      0.800      0.571
----------------------------------------------------------------------------------------------------------------------------------------------------------------------------------------------------------------------------------------------------------------------AAGACCTTCGAGCTCTACCCGA------------------      0.073      0.000      0.082
----------------------------------------------------------------------------------------------------------------------------------------------------------------------------------------------------------------------------------------------------------------------AAGACCTTCGAGCTCTACCC--------------------      0.000      0.200      0.082
--------------------------------------------------------------------------------------------------------------------------------------------------------------------------------------------------------------------------------------------------------------------CCAAGACCTTCGAGCTCTACC---------------------      0.514      3.101      0.652
--------------------------------------------------------------------------------------------------------------------------------------------------------------------------------------------------------------------------------------------------------------------CCAAGACCTTCGAGCTCTAC----------------------      0.073      0.300      0.082
--------------------------------------------------------------------------------------------------------------------------------------------------------------------------------------------------------------------------------------------------------------------CCAAGACCTTCGAGCTCTA-----------------------      0.000      0.100      0.082
-------------------------------------------------------------------------------------------------------------------------------------------------------------------------------------------------------------------------------------------------------------------CCCAAGACCTTCGAGCTCTACC---------------------      0.147      0.000      0.163
------------------------------------------------------------------------------------------------------------------------------------------------------------------------------------------------------------------------------------------------------------------TCCCAAGACCTTCGAGCTCTACC---------------------      0.147      0.400      0.326
------------------------------------------------------------------------------------------------------------------------------------------------------------------------------------------------------------------------------------------------------------------TCCCAAGACCTTCGAGCTCTAC----------------------      0.220      0.100      0.326
------------------------------------------------------------------------------------------------------------------------------------------------------------------------------------------------------------------------------------------------------------------TCCCAAGACCTTCGAGCTC-------------------------      0.000      0.000      0.082
-----------------------------------------------------------------------------------------------------------------------------------------------------------------------------------------------------------------------------------------------------------------TTCCCAAGACCTTCGAGCTCT------------------------      0.661      1.900      1.712
-----------------------------------------------------------------------------------------------------------------------------------------------------------------------------------------------------------------------------------------------------------------TTCCCAAGACCTTCGAGCTC-------------------------      1.689      2.200      2.038
-----------------------------------------------------------------------------------------------------------------------------------------------------------------------------------------------------------------------------------------------------------------TTCCCAAGACCTTCGAGCTCTAC----------------------      1.028      1.000      2.772
-----------------------------------------------------------------------------------------------------------------------------------------------------------------------------------------------------------------------------------------------------------------TTCCCAAGACCTTCGAGCTCTA-----------------------      0.220      0.500      0.734
-----------------------------------------------------------------------------------------------------------------------------------------------------------------------------------------------------------------------------------------------------------------TTCCCAAGACCTTCGAGC---------------------------      0.073      0.100      0.245
-----------------------------------------------------------------------------------------------------------------------------------------------------------------------------------------------------------------------------------------------------------------TTCCCAAGACCTTCGAGCTCTACC---------------------      0.661      1.000      0.652
-----------------------------------------------------------------------------------------------------------------------------------------------------------------------------------------------------------------------------------------------------TGACCGTCCTCCTTCCCAAG-------------------------------------      0.000      0.200      0.082
--------------------------------------------------------------------------------------------------------------------------------------------------------------------------------------------------------------------------------------------------GCGTGACCGTCCTCCTTCCC----------------------------------------      0.587      0.700      1.060
--------------------------------------------------------------------------------------------------------------------------------------------------------------------------------------------------------------------------------------------------GCGTGACCGTCCTCCTTCCCA---------------------------------------      1.028      0.500      0.408
--------------------------------------------------------------------------------------------------------------------------------------------------------------------------------------------------------------------------------------------------GCGTGACCGTCCTCCTTCC-----------------------------------------      0.294      1.000      0.082
-------------------------------------------------------------------------------------------------------------------------------------------------------------------------------------------------------------------------------------------------GGCGTGACCGTCCTCCTTCCCAA--------------------------------------      0.000      0.000      0.082
-------------------------------------------------------------------------------------------------------------------------------------------------------------------------------------------------------------------------------------------------GGCGTGACCGTCCTCCTTC------------------------------------------      0.220      0.300      0.326
-------------------------------------------------------------------------------------------------------------------------------------------------------------------------------------------------------------------------------------------------GGCGTGACCGTCCTCCTT-------------------------------------------      0.073      0.300      0.163
-------------------------------------------------------------------------------------------------------------------------------------------------------------------------------------------------------------------------------------------------GGCGTGACCGTCCTCCTTCC-----------------------------------------      0.294      4.201      1.223
-------------------------------------------------------------------------------------------------------------------------------------------------------------------------------------------------------------------------------------------------GGCGTGACCGTCCTCCTTCCC----------------------------------------      0.808      1.900      0.978
------------------------------------------------------------------------------------------------------------------------------------------------------------------------------------------------------------------------------------------------GGGCGTGACCGTCCTCCTTC------------------------------------------      0.000      0.000      0.163
```

```
---TGGGCGTGACCGTCCTCCT---      0.441     0.200     0.163
---TGGGCGTGACCGTCCTCCTT---     0.000     0.300     0.489
---TGGGCGTGACCGTCCTCCTTC---    4.112     7.201     3.098
---GTGGGCGTGACCGTCCTCCT---     0.073     0.000     0.082
---TGTGGGCGTGACCGTCCTC---      0.000     0.000     0.082
---TGTGGGCGTGACCGTCCTCC---     0.000     0.700     0.245
---TGTGGGCGTGACCGTCCT---       0.000     0.000     0.082
---TGTGGGCGTGACCGTCCTCCT---    2.056     2.501     2.528
---TTGTGGGCGTGACCGTCCT---      0.294     0.200     0.082
---TTGTGGGCGTGACCGTCCTCC---   41.923    67.114    55.120
---TTGTGGGCGTGACCGTCCTC---     1.028     1.700     0.408
---TTGTGGGCGTGACCGTCCTCCT---   0.514     1.100     0.734
---TTGTGGGCGTGACCGTCCTCCTTC--- 0.000     0.000     0.082
---TTGTGGGCGTGACCGTCC---       0.367     0.600     0.734
---TGTTGTGGGCGTGACCGTCCTCC---  0.000     0.000     0.082
---GGTGTTGTGGGCGTGACCG---      0.000     0.000     0.082
---ACCGACCCCGGTGTTGTGG---      0.000     0.000     0.082
---ACCGACCCCGGTGTTGTGGGCG---   0.881     1.800     1.223
---ACCGACCCCGGTGTTGTGGGC---    0.367     0.100     0.245
---TGACCGACCCCGGTGTTGTG---     0.147     0.700     0.082
---TGACCGACCCCGGTGTTGTGG---    0.441     0.700     0.489
---ATGACCGACCCCGGTGTT---       0.073     0.200     0.163
---ATGACCGACCCCGGTGTTGT---     1.028     1.200     0.652
---ATGACCGACCCCGGTGTTGTG---    8.297    15.903     7.746
---ATGACCGACCCCGGTGTTG---      0.734     1.100     0.489
---CATGACCGACCCCGGTGTTG---     0.073     0.100     0.245
---CATGACCGACCCCGGTGTT---      0.000     0.000     0.082
---ACATGACCGACCCCGGTG---       0.294     0.200     0.082
---ACATGACCGACCCCGGTGTTG---   14.244    28.306    19.080
---ACATGACCGACCCCGGTGTT---     0.220     0.100     0.652
---ACATGACCGACCCCGGTGT---      0.073     0.200     0.082
---ACATGACCGACCCCGGTGTTGT---   0.073     0.100     0.163
---AACATGACCGACCCCGGTGT---     0.294     0.100     0.326
---AACATGACCGACCCCGGTGTT---    0.073     0.000     0.245
----CGGTGGATGTAGACGGTCCAGA---  0.000     0.000     0.082

TGCGCGGTGGATGTAGACGGTCCAGACCGCTCCTGGCGCCGGTGATTTTAGACCGTTTGTTCGTGGTGGTGTTGAGGAACACCGGTTTTTGCAGTTGTAGCTGTTGTCGTCGTGGTGAATCAGGGCTTGTCAGTGGACAGGTAGCAGCTCTTGCCGTCCGTGTGGCCGTAGTAGGTCCCCTTGAGGTCCCCCGCCTT                                                   AC                              C
      GAACATGACCGACCCCGGTGTTGTGGGCGTGACCGTCCTCCTTCCCAAG  CTTCGAGCTCTACCCGAACGTCGAGTTCAAGTA G
      CTTGTACTGGCTGGGGCCACAACACCCGCACTGGCAGGAGGAAGGGTTC  GAAGCTCGAGATGGGCTTGCAGCTCAAGTTCAT /
---ATACCT                                                AA                              -

---CTTGTACTGGCTGGGGCCA---            0.000     0.000     0.082
---CTTGTACTGGCTGGGGCCAC---           0.000     0.000     0.082
---TCTTGTACTGGCTGGGGCCACA---         1.762     1.000     0.897
---GTACTGGCTGGGGCCACA---             0.441     0.400     0.326
---CTTGTACTGGCTGGGGCCACA---         16.152    19.004    15.085
---CTCTTGTACTGGCTGGGGCCACA---        0.073     0.000     0.326
---TTGTACTGGCTGGGGCCACA---           1.909     0.800     2.202
---TGTACTGGCTGGGGCCACA---            4.772     4.601     2.609
---CTTGTACTGGCTGGGGCCACAA---         0.000     0.100     0.163
---GTACTGGCTGGGGCCACAA---            0.220     0.100     0.326
---TGTACTGGCTGGGGCCACAA---          10.866    11.702    12.312
---TTGTACTGGCTGGGGCCACAA---          1.322     0.900     3.262
---TCTTGTACTGGCTGGGGCCACAA---        0.073     0.000     0.082
---TACTGGCTGGGGCCACAA---             0.073     0.000     0.082
---ACTGGCTGGGGCCACAAC---             0.000     0.000     0.082
---TGTACTGGCTGGGGCCACAAC---          0.734     1.100     0.571
---TACTGGCTGGGGCCACAAC---            0.000     0.000     0.082
---GTACTGGCTGGGGCCACAAC---           0.367     0.700     0.815
---ACTGGCTGGGGCCACAACA---            0.367     0.100     0.245
---GTACTGGCTGGGGCCACAACA---          2.349    10.702     7.094
---ACTGGCTGGGGCCACAACAC---           0.367     0.300     0.326
---ACTGGCTGGGGCCACAACACC---          6.314     6.301     3.506
---CTGGCTGGGGCCACAACACC---           0.073     0.000     0.082
---ACTGGCTGGGGCCACAACACCC---         4.699     5.201     3.506
---CTGGCTGGGGCCACAACACCC---          0.000     0.100     0.082
---GGCTGGGGCCACAACACCCGC---          0.220     0.700     0.408
---GCCACAACACCCGCACTGGCA---          0.000     0.100     0.163
---ACAACACCCGCACTGGCAG---            0.000     0.100     0.082
---ACAACACCCGCACTGGCAGG---           0.147     2.100     0.571
---CACAACACCCGCACTGGCAGGA---         0.000     0.700     0.082
---AACACCCGCACTGGCAGGA---            0.000     0.700     0.245
---ACAACACCCGCACTGGCAGGA---         64.683   779.057   171.231
---CAACACCCGCACTGGCAGGA---           0.514    15.203     2.038
---ACACCCGCACTGGCAGGA---             0.220     0.600     0.245
---ACAACACCCGCACTGGCAGGAG---         0.000     0.000     0.082
---ACACCCGCACTGGCAGGAG---            0.441     5.201     0.978
---CACCCGCACTGGCAGGAG---             0.000     1.400     0.489
---CAACACCCGCACTGGCAGGAG---          0.220     3.201     0.326
---AACACCCGCACTGGCAGGAG---           0.147     0.400     0.326
---ACACCCGCACTGGCAGGAGG---           1.101     9.402     2.935
---CACCCGCACTGGCAGGAGG---            0.000     0.500     0.326
---AACACCCGCACTGGCAGGAGG---          0.000     0.100     0.082
---CACCCGCACTGGCAGGAGGA---           1.909    11.902     5.055
---ACACCCGCACTGGCAGGAGGA---        440.596  3364.180  1122.867
---ACCCGCACTGGCAGGAGGA---            1.542     4.201     1.712
---CCCGCACTGGCAGGAGGA---             0.147     3.001     0.489
---AACACCCGCACTGGCAGGAGGA---         1.028     6.301     1.468
---ACACCCGCACTGGCAGGAGGAA---         3.377    18.604     7.828
---ACCCGCACTGGCAGGAGGAA---          17.621    35.707    12.394
---CACCCGCACTGGCAGGAGGAA---         18.135    42.109    20.140
---CCCGCACTGGCAGGAGGAA---            1.615     3.001     1.386
---CGCACTGGCAGGAGGAAG---             0.220     0.600     1.223
---CCCGCACTGGCAGGAGGAAG---           0.808     0.600     1.957
---CCGCACTGGCAGGAGGAAG---            0.514     2.100     1.223
---CACCCGCACTGGCAGGAGGAAG---         0.294     0.200     0.245
---ACACCCGCACTGGCAGGAGGAAG---        0.000     0.000     0.082
---ACCCGCACTGGCAGGAGGAAG---         13.803    10.202     5.789
---CGCACTGGCAGGAGGAAGG---            0.147     0.200     0.571
---CCCGCACTGGCAGGAGGAAGG---          3.818     3.401     3.588
---CCGCACTGGCAGGAGGAAGG---           0.954     2.400     0.734
---GCACTGGCAGGAGGAAGG---             0.000     0.700     0.326
---GCACTGGCAGGAGGAAGGG---            0.000     0.100     0.245
---CCGCACTGGCAGGAGGAAGGG---          0.000     0.000     0.163
---CGCACTGGCAGGAGGAAGGG---           0.881     0.900     1.386
---CACTGGCAGGAGGAAGGG---             0.000     0.000     0.163
---ACTGGCAGGAGGAAGGGT---             0.000     0.300     0.163
---CGCACTGGCAGGAGGAAGGGT---        198.602   170.034   229.205 miR_t98
---CCGCACTGGCAGGAGGAAGGGT---         4.405     6.201     7.665
---CCCGCACTGGCAGGAGGAAGGGT---        0.000     0.000     0.082
---CACTGGCAGGAGGAAGGGT---            0.220     0.600     0.815
---GCACTGGCAGGAGGAAGGGT---           4.332     6.401     7.094
---ACTGGCAGGAGGAAGGGTT---            0.147     0.100     0.163
---GCACTGGCAGGAGGAAGGGTT---          6.388     8.002     8.154
---ACTGGCAGGAGGAAGGGTTCA---          0.147     0.000     0.082
---ACTGGCAGGAGGAAGGGTTCAA---         4.405     2.100     3.588
---CTGGCAGGAGGAAGGGTTCAA---          0.073     0.100     0.571
---GCAGGAGGAAGGGTTCAAGAA---          3.304     1.200     1.631
---GGCAGGAGGAAGGGTTCAAGAA---         0.514     0.100     0.489
---GCAGGAGGAAGGGTTCAAGAAG---         0.000     0.000     0.082
---GGAGGAAGGGTTCAAGAAG---            0.147     0.000     0.082
---AGGAGGAAGGGTTCAAGAAGC---          0.073     0.100     0.163
---CAGGAGGAAGGGTTCAAGAAGCT---        0.000     0.000     0.163
---GGAAGGGTTCAAGAAGCT---             0.147     0.000     0.163
---GAGGAAGGGTTCAAGAAGCT---           0.000     0.000     0.082
---GGAGGAAGGGTTCAAGAAGCT---         14.244     8.402    12.312
---AAGGGTTCAAGAAGCTCG---             0.000     0.000     0.082
---GGAAGGGTTCAAGAAGCTCG---           0.220     0.500     0.489
---AAGGGTTCAAGAAGCTCGA---            0.000     0.100     0.245
---AGGGTTCAAGAAGCTCGA---             0.147     0.000     0.326
---GAAGGGTTCAAGAAGCTCGA---           1.028     3.101     2.120
---GGAAGGGTTCAAGAAGCTCGA---          8.737    28.906    18.509
---AGGAAGGGTTCAAGAAGCTCGA---         0.073     0.900     0.408
---AAGGGTTCAAGAAGCTCGAG---           0.367     0.400     0.489
---AGGGTTCAAGAAGCTCGAG---            4.919     3.901     6.197
---GAAGGGTTCAAGAAGCTCGAG---         17.107    13.903    33.675
---GGAAGGGTTCAAGAAGCTCGAG---         9.398     2.400    18.509
---GGTTCAAGAAGCTCGAGA---             0.073     0.200     0.082
---GGAAGGGTTCAAGAAGCTCGAGA---     1114.668   368.374  2007.315
---GGGTTCAAGAAGCTCGAGA---            0.514     0.400     0.978
---AAGGGTTCAAGAAGCTCGAGA---         21.806    18.404    50.309
---AGGAAGGGTTCAAGAAGCTCGAGA---       2.496     0.100     2.854
---GAAGGGTTCAAGAAGCTCGAGA---      1779.342  1995.703  3762.513
---AGGGTTCAAGAAGCTCGAGA---          25.624    34.007    31.963
---AGGAAGGGTTCAAGAAGCTCGAGAT---     13.730     0.200     6.523
---AGGGTTCAAGAAGCTCGAGAT---       4836.346  4693.248  7237.277
---GGTTCAAGAAGCTCGAGAT---            3.744     2.400     6.197
---GGGTTCAAGAAGCTCGAGAT---          36.563    25.005    57.403
---GAAGGGTTCAAGAAGCTCGAGAT---     6860.031  4146.738 13298.850
---GTTCAAGAAGCTCGAGAT---             0.220     0.200     0.897
---GGAGGAAGGGTTCAAGAAGCTCGAGAT---    0.000     0.000     0.082
---GGAAGGGTTCAAGAAGCTCGAGAT---    5499.478   318.264  7215.995 miR_t20
---GAGGAAGGGTTCAAGAAGCTCGAGAT---     0.000     0.000     0.082
---AAGGGTTCAAGAAGCTCGAGAT---       289.717   394.080   623.851
---GAAGGGTTCAAGAAGCTCGAGATG---       0.000     0.000     0.082
---AAGGGTTCAAGAAGCTCGAGATG---        0.000     0.000     0.163
---GGTTCAAGAAGCTCGAGATGG---          0.147     0.100     0.082
---AGAAGCTCGAGATGGGCTT---            0.147     0.000     0.082
---AAGAAGCTCGAGATGGGCTT---           0.147     0.200     0.163
---TCAAGAAGCTCGAGATGGGCTT---         0.220     0.500     0.326
---CAAGAAGCTCGAGATGGGCTT---          0.294     0.200     0.326
---CGAGATGGGCTTGCAGCTCA---           0.000     0.100     0.082
---CGAGATGGGCTTGCAGCTCAA---          0.073     0.300     0.489
---AGATGGGCTTGCAGCTCAAGT---          0.294     0.700     0.652
---GATGGGCTTGCAGCTCAAGT---           0.000     0.200     0.163
---GGGCTTGCAGCTCAAGTTCA---           0.000     0.000     0.082
```

```
----------------------------------------------------------------------------------------------------------------------------------------------------------------------------------------------------------------------------------------------------------------------------GGGCTTGCAGCTCAAGTTCAT--      0.073      0.100      0.245
-----------------------------------------------------------------------------------------------------------------------------------------------------------------------------------------------------------------------------------------------------------------------------GGCTTGCAGCTCAAGTTCAT--      0.000      0.000      0.163

miRNA: miR_t3 chromosome_4 Start: 3694784 End: 3694900 Condition: Sulfur-deprivation
----TAATGCGGGAAAATGGTAAC---------------------------------------      6.461      1.200      3.262
------ATGCGGGAAAATGGTAACTTAG-----------------------------------      1.322      0.900      0.734
-------TGCGGGAAAATGGTAACTTAGT----------------------------------      0.073      0.100      0.082
-------TGCGGGAAAATGGTAACTTAG-----------------------------------      0.367      0.400      0.489
---------CGGGAAAATGGTAACTTAG-----------------------------------      0.000      0.100      0.000
---------------AATGGTAACTTAGTCATCCCA---------------------------      0.073      0.100      0.082
----------------ATGGTAACTTAGTCATCCCAA--------------------------      0.000      0.100      0.000
-----------------TGGTAACTTAGTCATCCCAAG-------------------------      0.073      0.100      0.082
--------------------TAACTTAGTCATCCCAAGGCGTG--------------------      1.322      3.101      0.978
--------------------TAACTTAGTCATCCCAAGG------------------------      0.073      0.100      0.163
--------------------TAACTTAGTCATCCCAAGGCG----------------------      8.223     27.205     12.638
--------------------TAACTTAGTCATCCCAAGGCGT---------------------      1.762      6.901      2.691
--------------------TAACTTAGTCATCCCAAG-------------------------      0.000      0.300      0.163
---------------------AACTTAGTCATCCCAAGGCG----------------------      0.147      0.400      0.245
---------------------AACTTAGTCATCCCAAGGCGTG--------------------      2.717      7.201      4.566
---------------------AACTTAGTCATCCCAAGGCGT---------------------      3.891      8.802      4.240
----------------------ACTTAGTCATCCCAAGGCG----------------------      1.322      3.901      0.978
----------------------ACTTAGTCATCCCAAGGCGTG--------------------    426.646   1111.425    426.202 miR_t3
----------------------ACTTAGTCATCCCAAGGCGTGT-------------------      0.147      0.200      0.489
----------------------ACTTAGTCATCCCAAGGC-----------------------      0.441      1.200      0.978
----------------------ACTTAGTCATCCCAAGGCGT---------------------      1.101      4.401      1.060
-----------------------CTTAGTCATCCCAAGGCGTG--------------------      0.661      0.600      0.326
------------------------TTAGTCATCCCAAGGCGTGTA------------------      6.241      7.001      7.828
------------------------TTAGTCATCCCAAGGCGT---------------------      0.000      0.300      0.000
------------------------TTAGTCATCCCAAGGCGTGTAT-----------------      0.220      0.700      0.245
------------------------TTAGTCATCCCAAGGCGTGT-------------------      3.598      4.801      1.712
------------------------TTAGTCATCCCAAGGCGTG--------------------      4.332      9.302      2.935
-------------------------TAGTCATCCCAAGGCGTG--------------------      0.147      0.900      0.408
-------------------------TAGTCATCCCAAGGCGTGT-------------------      0.000      0.200      0.082
-------------------------TAGTCATCCCAAGGCGTGTA------------------      0.147      0.500      0.082
--------------------------AGTCATCCCAAGGCGTGTATC----------------      0.000      0.100      0.000
----------------------------TCATCCCAAGGCGTGTATCA---------------      0.147      0.100      0.000
----------------------------TCATCCCAAGGCGTGTATCACC-------------      0.147      0.100      0.163

CATGTAATGC                    A  C                 A        C
          GGGAAAATGGTAACTTAGTC TC CAAGGCGTGTATCACCC TGTTACGGG A
          CCCTTTTACCATTGAATCAG AG GTTCCGCACATAGTGGG ACAATGTCC /
---------T                    C  T                 C        -

----------------------------------GTTCCGCACATAGTGGGCACA--------      0.147      0.200      0.082
--------------------------TCAGCAGTGTTCCGCACATAG----------------      0.000      0.100      0.000
-------------------------ATCAGCAGTGTTCCGCACAT------------------      0.073      1.100      0.571
------------------------AATCAGCAGTGTTCCGCACAT------------------      0.147      1.300      0.897
-----------------------GAATCAGCAGTGTTCCGCACAT------------------      0.000      0.100      0.000
----------------------TGAATCAGCAGTGTTCCGCA---------------------      0.073      0.400      0.000
---------------------TTGAATCAGCAGTGTTCCGCA---------------------      0.294      0.100      0.245
----------------------TGAATCAGCAGTGTTCCGC----------------------      0.073      0.400      0.489
-----------------------GAATCAGCAGTGTTCCGC----------------------      0.073      0.100      0.000
--------------------ATTGAATCAGCAGTGTTCCGC----------------------      1.322      4.801      4.240 miR_t3*
---------------------TTGAATCAGCAGTGTTCCGC----------------------      0.147      0.600      0.163
--------------------ATTGAATCAGCAGTGTTCCG-----------------------      0.147      0.300      0.163
--------------TTTACCATTGAATCAGCAGT-----------------------------      0.147      0.100      0.245
-------------TTTTACCATTGAATCAGCAGT-----------------------------      0.073      0.300      0.163

miRNA: miR_t61 chromosome_17 Start: 3137408 End: 3137970 Condition: Sulfur-deprivation
---------------------------------------------------------------------------------------------------------------------------------------------------------------------------------------------------------------------------------------------------------------------------------------------------------------------------------------TGCTTCAGTCTTACTTGCTT--G--------      0.073      0.600      0.652
---------------------------------------------------------------------------------------------------------------------------------------------------------------------------------------------------------------------------------------------------------------------------------------------------------------------------------------TGCTTCAGTCTTACTTGCTT-----------      0.000      0.200      0.163
----------------------------------------------------------------------------------------------------------------------------------------------------------------------------------------------------------------------------------------------------------------------------------------------------------------------------------GACGGCAATTGCTTCAGTC---------------------      0.000      0.100      0.000
-----------------------------------------------------------------------------------------------------------------------------------------------------------------------------------------------------------------------------------------------------------------------------------------------------------------------------------CGACGGCAATTGCTTCAGTC---------------------      0.000      0.100      0.000
----------------------------------------------------------------------------------------------------------------------------------------------------------------------------------------------------------------------------------------------------------------------------------------------------------------------------TCGACGGCAATTGCTTCAGTC---------------------      0.734      3.101      1.957
----------------------------------------------------------------------------------------------------------------------------------------------------------------------------------------------------------------------------------------------------------------------------------------------------------------------------TCGACGGCAATTGCTTCAG-----------------------      0.147      0.300      0.082
----------------------------------------------------------------------------------------------------------------------------------------------------------------------------------------------------------------------------------------------------------------------------------------------------------------------------TCGACGGCAATTGCTTCAGT----------------------      1.322      2.100      2.772
----------------------------------------------------------------------------------------------------------------------------------------------------------------------------------------------------------------------------------------------------------------------------------------------------------------------------TCGACGGCAATTGCTTCAGTCT--------------------      0.294      3.301      1.794
--------------------------------------------------------------------------------------------------------------------------------------------------------------------------------------------------------------------------------------------------------------------------------------------------------------------------GGTCGACGGCAATTGCTTCAG-----------------------      0.000      0.100      0.000
--------------------------------------------------------------------------------------------------------------------------------------------------------------------------------------------------------------------------------------------------------------------------------------------------------------------------GGTCGACGGCAATTGCTTCAGT----------------------      0.000      0.100      0.000
-------------------------------------------------------------------------------------------------------------------------------------------------------------------------------------------------------------------------------------------------------------------------------------------------------------------------TGGTCGACGGCAATTGCTT--------------------------      0.000      0.300      0.000
-------------------------------------------------------------------------------------------------------------------------------------------------------------------------------------------------------------------------------------------------------------------------------------------------------------------------TGGTCGACGGCAATTGCTTCA------------------------      0.000      4.901      1.468
-------------------------------------------------------------------------------------------------------------------------------------------------------------------------------------------------------------------------------------------------------------------------------------------------------------------------TGGTCGACGGCAATTGCTTC-------------------------      0.367      2.400      1.712
-------------------------------------------------------------------------------------------------------------------------------------------------------------------------------------------------------------------------------------------------------------------------------------------------------------------------TGGTCGACGGCAATTGCTTCAGT----------------------      1.175      3.901      2.365
-------------------------------------------------------------------------------------------------------------------------------------------------------------------------------------------------------------------------------------------------------------------------------------------------------------------------TGGTCGACGGCAATTGCTTCAG-----------------------     10.866     41.708     53.897
-------------------------------------------------------------------------------------------------------------------------------------------------------------------------------------------------------------------------------------------------------------------------------------------------------------------------TGGTCGACGGCAATTGCT---------------------------      0.073      0.800      0.815
-----------------------------------------------------------------------------------------------------------------------------------------------------------------------------------------------------------------------------------------------------------------------------------------------------------------------GGTGGTCGACGGCAATTGCT---------------------------      0.000      0.200      0.000
----------------------------------------------------------------------------------------------------------------------------------------------------------------------------------------------------------------------------------------------------------------------------------------------------------------------TGGTGGTCGACGGCAATTGCT---------------------------      0.147      0.700      1.223
----------------------------------------------------------------------------------------------------------------------------------------------------------------------------------------------------------------------------------------------------------------------------------------------------------------------TGGTGGTCGACGGCAATTGC----------------------------      0.073      0.200      0.571
----------------------------------------------------------------------------------------------------------------------------------------------------------------------------------------------------------------------------------------------------------------------------------------------------------------------TGGTGGTCGACGGCAATTGCTT--------------------------      0.000      0.200      0.082
---------------------------------------------------------------------------------------------------------------------------------------------------------------------------------------------------------------------------------------------------------------------------------------------------------------------GTGGTGGTCGACGGCAATTGC----------------------------      0.294      0.400      0.815
---------------------------------------------------------------------------------------------------------------------------------------------------------------------------------------------------------------------------------------------------------------------------------------------------------------------GTGGTGGTCGACGGCAATTGCT---------------------------      0.147      0.200      0.489
--------------------------------------------------------------------------------------------------------------------------------------------------------------------------------------------------------------------------------------------------------------------------------------------------------------------AGTGGTGGTCGACGGCAATTG-----------------------------      0.000      0.100      0.163
--------------------------------------------------------------------------------------------------------------------------------------------------------------------------------------------------------------------------------------------------------------------------------------------------------------------AGTGGTGGTCGACGGCAATTGCT---------------------------      0.073      0.100      0.245
--------------------------------------------------------------------------------------------------------------------------------------------------------------------------------------------------------------------------------------------------------------------------------------------------------------------AGTGGTGGTCGACGGCAATTGC----------------------------      0.441      0.800      0.571
--------------------------------------------------------------------------------------------------------------------------------------------------------------------------------------------------------------------------------------------------------------------------------------------------------------------AGTGGTGGTCGACGGCAATT------------------------------      0.073      0.200      0.082
-------------------------------------------------------------------------------------------------------------------------------------------------------------------------------------------------------------------------------------------------------------------------------------------------------------------GAGTGGTGGTCGACGGCAAT-------------------------------      0.147      0.200      0.978
-------------------------------------------------------------------------------------------------------------------------------------------------------------------------------------------------------------------------------------------------------------------------------------------------------------------GAGTGGTGGTCGACGGCAATT------------------------------      0.000      0.200      0.000
------------------------------------------------------------------------------------------------------------------------------------------------------------------------------------------------------------------------------------------------------------------------------------------------------------------TGAGTGGTGGTCGACGGCAA--------------------------------      1.689      1.600      5.137
------------------------------------------------------------------------------------------------------------------------------------------------------------------------------------------------------------------------------------------------------------------------------------------------------------------TGAGTGGTGGTCGACGGCAAT-------------------------------    212.772    199.140    651.493 miR_t61
------------------------------------------------------------------------------------------------------------------------------------------------------------------------------------------------------------------------------------------------------------------------------------------------------------------TGAGTGGTGGTCGACGGCA---------------------------------      0.220      0.100      0.978
------------------------------------------------------------------------------------------------------------------------------------------------------------------------------------------------------------------------------------------------------------------------------------------------------------------TGAGTGGTGGTCGACGGCAATT------------------------------      2.863      1.900      6.686
-----------------------------------------------------------------------------------------------------------------------------------------------------------------------------------------------------------------------------------------------------------------------------------------------------------------TTGAGTGGTGGTCGACGGCAAT-------------------------------      1.175      1.100      2.446
-----------------------------------------------------------------------------------------------------------------------------------------------------------------------------------------------------------------------------------------------------------------------------------------------------------------TTGAGTGGTGGTCGACGGCAA--------------------------------      1.175      0.300      1.957
----------------------------------------------------------------------------------------------------------------------------------------------------------------------------------------------------------------------------------------------------------------------------------------------------------------TTTGAGTGGTGGTCGACGGCAAT-------------------------------      0.514      0.100      0.652
-----------------------------------------------------------------------------------------------------------------------------------------------------------------------------------------------------------------------------------------------------------------------------------------------------------AGTGGTTTGAGTGGTGGTCG---------------------------------------      0.147      0.200      0.163
-------------------------------------------------------------------------------------------------------------------------------------------------------------------------------------------------------------------------------------------------------------------------------------------------------GGCCAGTGGTTTGAGTGGTGG------------------------------------------      0.073      0.100      0.000
------------------------------------------------------------------------------------------------------------------------------------------------------------------------------------------------------------------------------------------------------------------------------------------------------AGGCCAGTGGTTTGAGTGGTGG------------------------------------------      0.220      0.300      0.408
------------------------------------------------------------------------------------------------------------------------------------------------------------------------------------------------------------------------------------------------------------------------------------------------------AGGCCAGTGGTTTGAGTGGT--------------------------------------------      0.000      0.200      0.163
------------------------------------------------------------------------------------------------------------------------------------------------------------------------------------------------------------------------------------------------------------------------------------------------------AGGCCAGTGGTTTGAGTGGTG-------------------------------------------      0.808      0.300      4.322
----------------------------------------------------------------------------------------------------------------------------------------------------------------------------------------------------------------------------------------------------------------------------------------------------ACAGGCCAGTGGTTTGAGTG----------------------------------------------      0.073      0.300      0.978
----------------------------------------------------------------------------------------------------------------------------------------------------------------------------------------------------------------------------------------------------------------------------------------------------ACAGGCCAGTGGTTTGAGT-----------------------------------------------      0.000      0.100      0.000
----------------------------------------------------------------------------------------------------------------------------------------------------------------------------------------------------------------------------------------------------------------------------------------------------ACAGGCCAGTGGTTTGAGTGGT--------------------------------------------      0.000      0.100      0.000
----------------------------------------------------------------------------------------------------------------------------------------------------------------------------------------------------------------------------------------------------------------------------------------------------ACAGGCCAGTGGTTTGAGTGG---------------------------------------------      0.367      0.200      0.408
---------------------------------------------------------------------------------------------------------------------------------------------------------------------------------------------------------------------------------------------------------------------------------------------------AACAGGCCAGTGGTTTGAGTG----------------------------------------------      0.147      0.600      0.734
---------------------------------------------------------------------------------------------------------------------------------------------------------------------------------------------------------------------------------------------------------------------------------------------------AACAGGCCAGTGGTTTGAGTGG---------------------------------------------      0.073      0.300      0.163
-----------------------------------------------------------------------------------------------------------------------------------------------------------------------------------------------------------------------------------------------------------------------------------------------------GCAACAGGCCAGTGGTTT---------------------------------------------------      0.000      0.100      0.000
----------------------------------------------------------------------------------------------------------------------------------------------------------------------------------------------------------------------------------------------------------------------------------------------GGTGCAACAGGCCAGTGGTT----------------------------------------------------      0.000      0.100      0.000
---------------------------------------------------------------------------------------------------------------------------------------------------------------------------------------------------------------------------------------------------------------------------------------------TGGTGCAACAGGCCAGTGGTT----------------------------------------------------      2.570      6.901     11.334
-------------------------------------------------------------------------------------------------------------------------------------------------------------------------------------------------------------------------------------------------------------------------------------------TCTGGTGCAACAGGCCAGTGGT-----------------------------------------------------      0.000      0.100      0.000
-----------------------------------------------------------------------------------------------------------------------------------------------------------------------------------------------------------------------------------------------------------------------------------------TAGGTGCTCTTCTGGTGCAACA---------------------------------------------------------------      0.073      0.200      0.408
-----------------------------------------------------------------------------------------------------------------------------------------------------------------------------------------------------------------------------------------------------------------------------------------TAGGTGCTCTTCTGGTGCAAC----------------------------------------------------------------      0.000      0.100      0.245
-----------------------------------------------------------------------------------------------------------------------------------------------------------------TCGCATCGTCCGCGGTTGAGTTGC-----------------------------------------------------------------------------------------------------------------------------------------------------------------      0.000      0.100      0.000
------------------------------------------------------------------------------------------------------------------------------------GTTGGCATGGAGGTTATGGCT-------------------------------------------------------------------------------------------------------------------------------------------------------------------------------------------------      0.000      0.100      0.000

--------------------------------------------------------------------------------------------------------------------------------ACTTGTTGGCATGGAGGTTATGGCTGACCCGGTTCGCATCGTCCGCGGTTGAGTTGCGAAGAAGATGGTACAAGAGGATAGGCAAGGCCACCCGAGGGCATACTGGAAGCCGCAGAAATGCCAATGAGATGCATA        T                         G         A                           --   T   G
                                                                                                                                                                                                                                                                       GGTGCTCT CTGGTGCAACAGGCCAGTGGTTTGA TGGTGGTCG CGGCAATTGCTTCAGTCTTACTTGCTT  GGC TAC T
                                                                                                                                                                                                                                                                       TCACGAGA GACCACGTTGTCCGGTCACCAAACT GCCACCAGC GTCGTTAACGAAGTCAGAATGAACGAA  CCG ATG C
CCCAAGGTGGCTAGTTGGGACGACGGTGTGTCGTAGGCCCCGAAAGCCTAGGTCGTTGTCTGACTTCGGGAGTCACTAGCTATCGACGAACCTCGGCGTGGTCGGGCTGCAGAGGTGATTTATAGTATGTACGTTCGTGGTGCTTCAGGGGCGGGACTGCTCGAAAGAACTCGGGTACGGAATGGGGAACTTTGTTGTACTATAAACTGTGCTGCCGGGCGTCTTCCACTCGTGACCACCGTTTTGTCGGCCGCCGTCCCAAA        C                         A         C                           CT   C   C

----AGGTGGCTAGTTGGGACG------------------------------------------------------------------------------------------------------------------------------------------------------------------------------------------------------------------------------------------------------------------------------------------------------------------------------------      0.000      0.100      0.000
--------------------------------------------------------------------------------------CGAACCTCGGCGTGGTCGGG------------------------------------------------------------------------------------------------------------------------------------------------------------------------------------------------------------------------------------------------      0.000      0.100      0.000
------------------------------------------------------------------------------------------------------------------------------------------------------------------------TCGGGTACGGAATGGGGAACT-----------------------------------------------------------------------------------------------------------------------------------------------------------      0.000      0.100      0.000
----------------------------------------------------------------------------------------------------------------------------------------------------------------------------------------------------------------------------------------------------------------------------------CCAAATCACGAGACGACCACGT------------------------------------------------------------------      0.147      0.200      2.202
--------------------------------------------------------------------------------------------------------------------------------------------------------------------------------------------------------------------------------------------------------------------------------TCCCAAATCACGAGACGACCACGT------------------------------------------------------------------      0.294      0.100      0.897
-----------------------------------------------------------------------------------------------------------------------------------------------------------------------------------------------------------------------------------------------------------------------------------CAAATCACGAGACGACCACGT------------------------------------------------------------------      0.000      0.100      0.082
---------------------------------------------------------------------------------------------------------------------------------------------------------------------------------------------------------------------------------------------------------------------------------CCCAAATCACGAGACGACCACGT------------------------------------------------------------------      0.441      0.700      5.300
-------------------------------------------------------------------------------------------------------------------------------------------------------------------------------------------------------------------------------------------------------------------------------GTCCCAAATCACGAGACGACCACGT------------------------------------------------------------------      0.000      0.100      0.000
----------------------------------------------------------------------------------------------------------------------------------------------------------------------------------------------------------------------------------------------------------------------------------CCAAATCACGAGACGACCACGTT-----------------------------------------------------------------      0.073      0.200      0.489
---------------------------------------------------------------------------------------------------------------------------------------------------------------------------------------------------------------------------------------------------------------------------------CCCAAATCACGAGACGACCACGTT-----------------------------------------------------------------      0.367      0.200      2.528
--------------------------------------------------------------------------------------------------------------------------------------------------------------------------------------------------------------------------------------------------------------------------------TCCCAAATCACGAGACGACCACGTT-----------------------------------------------------------------      0.000      0.100      0.163
-------------------------------------------------------------------------------------------------------------------------------------------------------------------------------------------------------------------------------------------------------------------------------------------GAGACGACCACGTTGTCCGGT----------------------------------------------------------      0.073      0.300      0.163
--------------------------------------------------------------------------------------------------------------------------------------------------------------------------------------------------------------------------------------------------------------------------------------------AGACGACCACGTTGTCCGGT----------------------------------------------------------      0.000      0.100      0.163
---------------------------------------------------------------------------------------------------------------------------------------------------------------------------------------------------------------------------------------------------------------------------------------------GACGACCACGTTGTCCGGTCA--------------------------------------------------------      0.000      0.200      0.082
-----------------------------------------------------------------------------------------------------------------------------------------------------------------------------------------------------------------------------------------------------------------------------------------------CGACCACGTTGTCCGGTCACC------------------------------------------------------      0.073      1.300      0.163
-------------------------------------------------------------------------------------------------------------------------------------------------------------------------------------------------------------------------------------------------------------------------------------------------------CACGTTGTCCGGTCACCAA----------------------------------------------------      0.000      0.200      0.000
--------------------------------------------------------------------------------------------------------------------------------------------------------------------------------------------------------------------------------------------------------------------------------------------------------ACGTTGTCCGGTCACCAA----------------------------------------------------      0.000      0.100      0.082
--------------------------------------------------------------------------------------------------------------------------------------------------------------------------------------------------------------------------------------------------------------------------------------------------------TGTCCGGTCACCAAACTAGC----------------------------------------------      0.000      0.200      0.000
--------------------------------------------------------------------------------------------------------------------------------------------------------------------------------------------------------------------------------------------------------------------------------------------------------TGTCCGGTCACCAAACTAGCCA--------------------------------------------      0.073      0.200      0.163
----------------------------------------------------------------------------------------------------------------------------------------------------------------------------------------------------------------------------------------------------------------------------------------------------------TCCGGTCACCAAACTAGCCA--------------------------------------------      0.073      0.100      0.082
---------------------------------------------------------------------------------------------------------------------------------------------------------------------------------------------------------------------------------------------------------------------------------------------------------GTCCGGTCACCAAACTAGCCA--------------------------------------------      0.881      0.800      1.468
-----------------------------------------------------------------------------------------------------------------------------------------------------------------------------------------------------------------------------------------------------------------------------------------------------------------------------AACTAGCCACCAGCCGTCGT---------------------------------      0.000      0.100      0.000
-----------------------------------------------------------------------------------------------------------------------------------------------------------------------------------------------------------------------------------------------------------------------------------------------------------------------------AACTAGCCACCAGCCGTCGTT--------------------------------      0.147      0.100      0.082
-------------------------------------------------------------------------------------------------------------------------------------------------------------------------------------------------------------------------------------------------------------------------------------------------------------------------------CTAGCCACCAGCCGTCGTT--------------------------------      0.000      0.100      0.000
-----------------------------------------------------------------------------------------------------------------------------------------------------------------------------------------------------------------------------------------------------------------------------------------------------------------------------------CCACCAGCCGTCGTTAACGAA--------------------------      0.000      0.100      0.245
-------------------------------------------------------------------------------------------------------------------------------------------------------------------------------------------------------------------------------------------------------------------------------------------------------------------------------------ACCAGCCGTCGTTAACGAAGT------------------------      0.000      0.100      0.489
---------------------------------------------------------------------------------------------------------------------------------------------------------------------------------------------------------------------------------------------------------------------------------------------------------------------------------------CAGCCGTCGTTAACGAAGTCA----------------------      0.000      0.100      0.000
-----------------------------------------------------------------------------------------------------------------------------------------------------------------------------------------------------------------------------------------------------------------------------------------------------------------------------------------TAACGAAGTCAGAATGAA---------------      0.000      0.100      0.000
-----------------------------------------------------------------------------------------------------------------------------------------------------------------------------------------------------------------------------------------------------------------------------------------------------------------------------------------TAACGAAGTCAGAATGAAC--------------      0.073      0.400      0.326
------------------------------------------------------------------------------------------------------------------------------------------------------------------------------------------------------------------------------------------------------------------------------------------------------------------------------------------AACGAAGTCAGAATGAAC--------------      0.000      0.200      0.082
-------------------------------------------------------------------------------------------------------------------------------------------------------------------------------------------------------------------------------------------------------------------------------------------------------------------------------------------ACGAAGTCAGAATGAACG-------------      0.000      0.300      0.163
-----------------------------------------------------------------------------------------------------------------------------------------------------------------------------------------------------------------------------------------------------------------------------------------------------------------------------------------TAACGAAGTCAGAATGAACG-------------      0.147      0.300      1.060
-----------------------------------------------------------------------------------------------------------------------------------------------------------------------------------------------------------------------------------------------------------------------------------------------------------------------------------------TAACGAAGTCAGAATGAACGA------------     27.679     34.807    217.871
-------------------------------------------------------------------------------------------------------------------------------------------------------------------------------------------------------------------------------------------------------------------------------------------------------------------------------------------ACGAAGTCAGAATGAACGA------------      0.073      1.200      1.631
----------------------------------------------------------------------------------------------------------------------------------------------------------------------------------------------------------------------------------------------------------------------------------------------------------------------------------------TTAACGAAGTCAGAATGAACGA------------      0.147      0.500      0.571
------------------------------------------------------------------------------------------------------------------------------------------------------------------------------------------------------------------------------------------------------------------------------------------------------------------------------------------AACGAAGTCAGAATGAACGA------------      0.367      1.900      3.995
------------------------------------------------------------------------------------------------------------------------------------------------------------------------------------------------------------------------------------------------------------------------------------------------------------------------------------------AACGAAGTCAGAATGAACGAA-----------      0.000      0.300      0.082

miRNA: miR_t91 chromosome_9 Start: 6365819 End: 6366106 Condition: Sulfur-deprivation
-------------------TGCCATCGTCACCGGTCTAAT--------------------------------------------------------------------------------------------------      0.000      0.100      0.000
---------------------------------------------------C----AGGGAAGACACAGGATCCATT-------------------------------------------------------------      0.073      0.100      0.082
--------------------------------------------------------AGGGAAGACACAGGATCCATT-------------------------------------------------------------      0.000      0.200      0.245
--------------------------------------------------------------ACACAGGATCCATTTTCTAGTG------------------------------------------------------      0.073      0.100      0.000
---------------------------------------------------------------------------------GCCTGGCTGGGATCAATGCAAG-----------------------------------      0.000      0.100      0.000
----------------------------------------------------------------------------------CCTGGCTGGGATCAATGCAA------------------------------------      1.762     10.602      2.935
----------------------------------------------------------------------------------CCTGGCTGGGATCAATGCA-------------------------------------      0.881      6.401      1.468
----------------------------------------------------------------------------------CCTGGCTGGGATCAATGCAAG-----------------------------------     27.753    130.726     33.105
```

```
-------------------------------------------------------------------------------------------CCTGGCTGGGATCAATGC---------------------------------------------      0.073      1.700      0.326
-------------------------------------------------------------------------------------------CCTGGCTGGGATCAATGCAAGAT----------------------------------------      0.220      0.100      0.000
-------------------------------------------------------------------------------------------CCTGGCTGGGATCAATGCAAGA-----------------------------------------      1.615     11.402      2.772
--------------------------------------------------------------------------------------------CTGGCTGGGATCAATGCAAG------------------------------------------      0.000      0.200      0.000
---------------------------------------------------------------------------------------------TGGCTGGGATCAATGCAAGATGAT-------------------------------------      0.367      0.100      0.734
---------------------------------------------------------------------------------------------TGGCTGGGATCAATGCAAGATG---------------------------------------      5.066      6.501     13.209
---------------------------------------------------------------------------------------------TGGCTGGGATCAATGCAAGAT----------------------------------------      0.000      0.100      0.082
---------------------------------------------------------------------------------------------TGGCTGGGATCAATGCAAG------------------------------------------      0.147      0.400      0.245
---------------------------------------------------------------------------------------------TGGCTGGGATCAATGCAAGATGA--------------------------------------      0.441      0.400      1.631
---------------------------------------------------------------------------------------------TGGCTGGGATCAATGCAAGA-----------------------------------------      0.294      0.200      0.408
------------------------------------------------------------------------------------------------CTGGGATCAATGCAAGATGAT-------------------------------------      0.000      0.100      0.000
-------------------------------------------------------------------------------------------------TGGGATCAATGCAAGATGAT-------------------------------------      1.909      1.200      7.420
-------------------------------------------------------------------------------------------------TGGGATCAATGCAAGATGA--------------------------------------      0.000      0.100      0.163
-------------------------------------------------------------------------------------------------TGGGATCAATGCAAGATGATA------------------------------------      2.863      1.800      8.317
--------------------------------------------------------------------------------------------------------AATGCAAGATGATAGTTGGAT-----------------------------      1.762      1.300      1.712
--------------------------------------------------------------------------------------------------------AATGCAAGATGATAGTTGGATC----------------------------      0.367      0.800      1.549
---------------------------------------------------------------------------------------------------------ATGCAAGATGATAGTTGGATC----------------------------      0.000      0.200      0.082
----------------------------------------------------------------------------------------------------------TGCAAGATGATAGTTGGATCC---------------------------     13.216      5.401     18.102
------------------------------------------------------------------------------------------------------------CAAGATGATAGTTGGATCCTGG------------------------      0.147      0.100      0.245
-------------------------------------------------------------------------------------------------------------AAGATGATAGTTGGATCC---------------------------      0.000      0.100      0.000
----------------------------------------------------------------------------------------------------------------ATGATAGTTGGATCCTGGACGC--------------------      0.073      0.100      0.000
----------------------------------------------------------------------------------------------------------------ATGATAGTTGGATCCTGG------------------------      0.587      1.200      0.815
----------------------------------------------------------------------------------------------------------------ATGATAGTTGGATCCTGGACG---------------------     19.970     17.404     19.732
----------------------------------------------------------------------------------------------------------------ATGATAGTTGGATCCTGGAC----------------------      0.514      1.000      0.408
----------------------------------------------------------------------------------------------------------------ATGATAGTTGGATCCTGGA-----------------------      0.881      4.101      3.262
-----------------------------------------------------------------------------------------------------------------TGATAGTTGGATCCTGGACG---------------------      1.395      1.100      2.528
------------------------------------------------------------------------------------------------------------------GATAGTTGGATCCTGGACG---------------------      0.000      0.100      0.082
--------------------------------------------------------------------------------------------------------------------TAGTTGGATCCTGGACGCCG------------------      0.000      0.100      0.082
---------------------------------------------------------------------------------------------------------------------AGTTGGATCCTGGACGCCGAAG---------------      0.000      0.200      0.245
----------------------------------------------------------------------------------------------------------------------GTTGGATCCTGGACGCCGAAG---------------      0.294      0.100      0.326
------------------------------------------------------------------------------------------------------------------------TGGATCCTGGACGCCGAAGG--------------      0.147      0.100      0.082
------------------------------------------------------------------------------------------------------------------------TGGATCCTGGACGCCGAAGGCC------------      0.073      0.100      0.163
------------------------------------------------------------------------------------------------------------------------TGGATCCTGGACGCCGAAGGC-------------      0.881      1.000      0.652
-------------------------------------------------------------------------------------------------------------------------------TGGACGCCGAAGGCCATCATG------      5.433     10.902     13.535
-------------------------------------------------------------------------------------------------------------------------------TGGACGCCGAAGGCCATCA--------      0.000      0.100      0.326

-----------------AG            A       AA  TAATG         C----         ACA  A         T                    G                             A    A         G
                   GGTGCCATCGTC CCGGTCT  TC     CCAGGCAAC     AGGGAAGAC   GG TCCATTTTC AGTGCCTGGCTGGGATCAAT CAAGATGATAGTTGGATCCTGGACGCCGA GGCC TCATGCGGT T
                   CCACGGTAGCAG GGCCAGA  AG     GGTCCGTTG     TCCCTTCTG   CC AGGTAGAGG TCGCGGACCGACCCTAGTTA GTTCTGCTGTCGATCTAGGACCTGCGGTT CCGG AGTACGCCA A
GGGACCTCGGTTGCTGCCA            C       CG  CCTTG         TTCCC         GCA  C         T                    G                             G    C         A

----------------------------------------------------------------------------------------------------------------------------AGGACCTGCGGTTGCCGGCAGT--------      0.220      0.400      0.082
-----------------------------------------------------------------------------------------------------------------------------GGACCTGCGGTTGCCGGCAGT--------      1.982      2.400      2.528
---------------------------------------------------------------------------------------------------------------------------TAGGACCTGCGGTTGCCGGCA----------      0.000      0.100      0.082
-----------------------------------------------------------------------------------------------------------------GCTGTCGATCTAGGACCT-----------------------      2.349      0.600      3.425
----------------------------------------------------------------------------------------------------------------TGCTGTCGATCTAGGACCT-----------------------      0.220      0.300      0.571
--------------------------------------------------------------------------------------------------------------TCTGCTGTCGATCTAGGACCT-----------------------    295.664     73.115    278.372
-------------------------------------------------------------------------------------------------------------TTCTGCTGTCGATCTAGGACCT-----------------------      0.734      0.100      0.326
---------------------------------------------------------------------------------------------------------------CTGCTGTCGATCTAGGACCT-----------------------      6.975      2.200     10.029
--------------------------------------------------------------------------------------------------------------TCTGCTGTCGATCTAGGAC-------------------------      9.765      4.201     12.638
----------------------------------------------------------------------------------------------------------AGGTTCTGCTGTCGATCTAGGA--------------------------      0.294      0.400      1.223
-----------------------------------------------------------------------------------------------------------GGTTCTGCTGTCGATCTAGGA--------------------------      0.000      0.100      0.000
--------------------------------------------------------------------------------------------------------------TCTGCTGTCGATCTAGGA--------------------------      2.863      0.600      2.283
---------------------------------------------------------------------------------------------------------TAGGTTCTGCTGTCGATCT------------------------------      0.000      0.100      0.000
------------------------------------------------------------------------------------------------------AGTTAGGTTCTGCTGTCGATCT------------------------------      4.699      1.500      3.751
-------------------------------------------------------------------------------------------------------GTTAGGTTCTGCTGTCGATCT------------------------------      0.147      0.100      0.326
------------------------------------------------------------------------------------------------------AGTTAGGTTCTGCTGTCGAT--------------------------------      0.000      0.200      0.000
-------------------------------------------------------------------------------------------------ACCCTAGTTAGGTTCTGCTGT------------------------------------      0.000      0.200      0.082
-----------------------------------------------------------------------------------------------CGACCCTAGTTAGGTTCTGCT--------------------------------------      0.147      0.800      1.142
------------------------------------------------------------------------------------------------GACCCTAGTTAGGTTCTGCT--------------------------------------      0.000      0.100      0.082
----------------------------------------------------------------------------------------------CCGACCCTAGTTAGGTTCTGC---------------------------------------      0.073      0.600      0.245
---------------------------------------------------------------------------------------------ACCGACCCTAGTTAGGTTCTGC---------------------------------------      0.000      0.100      0.082
-------------------------------------------------------------------------------------------GGACCGACCCTAGTTAGGTTCT-----------------------------------------      0.000      0.300      0.082
--------------------------------------------------------------------------------------------GACCGACCCTAGTTAGGTTCT-----------------------------------------      0.000      0.100      0.000
---------------------------------------------------------------------------------------------ACCGACCCTAGTTAGGTTCT-----------------------------------------      0.073      0.100      0.000
-----------------------------------------------------------------------------------------GCGGACCGACCCTAGTTAGGTT-------------------------------------------      0.147      0.200      0.082
---------------------------------------------------------------------------------------TCGCGGACCGACCCTAGTTAGGT--------------------------------------------      0.220      0.900      0.815
------------------------------------------------------------------------------------------CGGACCGACCCTAGTTAGGT--------------------------------------------      2.790      8.302      3.343
--------------------------------------------------------------------------------------------GACCGACCCTAGTTAGGT--------------------------------------------      5.580      7.802      8.806
-------------------------------------------------------------------------------------------GGACCGACCCTAGTTAGGT--------------------------------------------      8.297     13.703     11.905
-----------------------------------------------------------------------------------------GCGGACCGACCCTAGTTAGGT--------------------------------------------   1480.595   2683.942   2318.548 miR_t91
----------------------------------------------------------------------------------------CGCGGACCGACCCTAGTTAGGT--------------------------------------------      2.496      8.402      5.382
------------------------------------------------------------------------------------------CGGACCGACCCTAGTTAGG---------------------------------------------      0.073      0.400      0.082
-----------------------------------------------------------------------------------------GCGGACCGACCCTAGTTAGG---------------------------------------------     41.115     71.314     56.995
-------------------------------------------------------------------------------------------GGACCGACCCTAGTTAGG---------------------------------------------      0.000      0.200      0.245
----------------------------------------------------------------------------------------CGCGGACCGACCCTAGTTAGG---------------------------------------------      0.073      0.100      0.082
-----------------------------------------------------------------------------------------GCGGACCGACCCTAGTTAG----------------------------------------------      0.734      1.900      1.305
-----------------------------------------------------------------------------------------GCGGACCGACCCTAGTTA-----------------------------------------------      0.147      1.600      0.245
--------------------------------------------------------------------------CCCAGGTAGAGGTTCGCGGAC-----------------------------------------------------------      0.073      0.200      0.163
--------------------------------------------------------------------------CCCAGGTAGAGGTTCGCGGA------------------------------------------------------------      0.000      0.100      0.000
----------------------------------------------------------------------GGCACCCAGGTAGAGGTTCGC---------------------------------------------------------------      0.000      0.100      0.082
-------------------------------------------------------------------TCTGGCACCCAGGTAGAGGTTC-----------------------------------------------------------------      0.661      1.700      0.734
--------------------------------------------------------------------CTGGCACCCAGGTAGAGGTTC-----------------------------------------------------------------      0.000      0.300      0.082
----------------------------------------------------------------------GGCACCCAGGTAGAGGTTC-----------------------------------------------------------------      0.000      0.100      0.082
---------------------ACGGTAGCAGCGGCCAGACGA----------------------------------------------------------------------------------------------------------------      0.441      1.000      0.489
--------------------CACGGTAGCAGCGGCCAGACGA----------------------------------------------------------------------------------------------------------------      0.220      0.200      0.408
----------------CCACCACGGTAGCAGCGGCCA---------------------------------------------------------------------------------------------------------------------      0.000      0.100      0.000
---------------GCCACCACGGTAGCAGCGGCCA---------------------------------------------------------------------------------------------------------------------      0.367      0.300      0.408
---------------GCCACCACGGTAGCAGCGGCC----------------------------------------------------------------------------------------------------------------------      1.762      0.400      0.652
----------------CCACCACGGTAGCAGCGGCC----------------------------------------------------------------------------------------------------------------------      0.000      0.100      0.000
------------------ACCACGGTAGCAGCGGCC----------------------------------------------------------------------------------------------------------------------      0.000      0.100      0.082
----CCTCGGTTGCTGCCACCACGGT--------------------------------------------------------------------------------------------------------------------------------      0.294      0.300      0.245

miRNA: miR_t77,miR_t58 chromosome_3 Start: 6573885 End: 6574012 Condition: Nutrient-replete
---------------------------------------------------GCCGGAACCAACACGACA-      0.661      6.501      0.326
----------------------------------------------TCACCGCCGGAACCAACACGAC--      0.073      0.000      0.000
----------------------------------------------TCACCGCCGGAACCAACA------      0.073      0.000      0.000
----------------------------------------------TCACCGCCGGAACCAACACGA---      0.073      0.100      0.000
----------------------------------------------TCACCGCCGGAACCAACACG----      1.248      0.100      0.000
--------------------------------------------TGTCACCGCCGGAACCAAC-------      0.073      0.000      0.000
--------------------------------------------TGTCACCGCCGGAACCAACA------      0.147      0.000      0.000
-----------------------------------------GCATGTCACCGCCGGAACCAAC-------      0.073      0.000      0.000
-----------------------------------------GCATGTCACCGCCGGAACC----------      0.073      0.000      0.000
-------------------------------------CACCGCATGTCACCGCCGGAA------------      0.220      0.000      0.000
-----------------------------------TTCACCGCATGTCACCGCCGGA-------------      0.073      0.000      0.000
-----------------------------------TTCACCGCATGTCACCGCCGG--------------      0.734      0.800      0.000
--------------------------------ACATTCACCGCATGTCAC--------------------      0.147      0.000      0.000
-------------------------------CACATTCACCGCATGTCA---------------------      0.147      0.100      0.000
-------------------------------CACATTCACCGCATGTCACC-------------------      2.717      2.901      0.245
------------------------------TCACATTCACCGCATGTCAC--------------------      1.248      0.500      0.082
------------------------------TCACATTCACCGCATGTCA---------------------      0.661      1.500      0.000
------------------------------TCACATTCACCGCATGTCACC-------------------      8.884      6.801      0.082
-----------------------------TTCACATTCACCGCATGTCACC-------------------      1.982      2.601      0.408
-----------------------------TTCACATTCACCGCATGTCAC--------------------      5.653      9.102      0.326
-----------------------------TTCACATTCACCGCATGTCA---------------------      8.150     18.104      0.163
-----------------------------TTCACATTCACCGCATGT-----------------------      0.073      0.000      0.000
----------------------------ATTCACATTCACCGCATGTC----------------------      4.038      3.101      0.326
----------------------------ATTCACATTCACCGCATGTCA---------------------    421.359    299.360     27.560
----------------------------ATTCACATTCACCGCATGTCACC-------------------      2.790      2.400      0.326
----------------------------ATTCACATTCACCGCATGTCAC--------------------      4.699      2.901      0.245
----------------------------ATTCACATTCACCGCATG------------------------      6.681      6.901      0.652
----------------------------ATTCACATTCACCGCATGT-----------------------      0.514      0.300      0.000
--------------------------TCATTCACATTCACCGCATG------------------------      0.147      0.100      0.000
--------------------------TCATTCACATTCACCGCATGTC----------------------      0.073      0.100      0.000
-------------------------ATCATTCACATTCACCGCAT-------------------------      0.073      0.000      0.000
-------------------------ATCATTCACATTCACCGCATG------------------------      0.441      1.000      0.163
-----------------TACACGCCATCATTCACATTCA-------------------------------      0.073      0.000      0.000
-----------------TACACGCCATCATTCACATT---------------------------------      0.147      0.300      0.000
-----------------TACACGCCATCATTCACA-----------------------------------      0.073      0.000      0.000

-------------T                                                      A
              AAGTACACGCCATCATTCACATTCACCGCATGTCACCGCCGGAACCAACACGAC G
              TTCATGTGCGGTAGTAAGTGTAAGTGGCGTACAGTGGCGGCCTTGGTTGTGCTG T
GCACGTTGCAATGC                                                      T

-----GTTGCAATGCTTCATGTGCGGTAG-----------------------------------------      0.220      0.000      0.000
------------TGCTTCATGTGCGGTAGT----------------------------------------      0.147      0.000      0.000
---------CAATGCTTCATGTGCGGTAGT----------------------------------------      0.073      0.000      0.000
----------AATGCTTCATGTGCGGTAGT----------------------------------------      0.147      0.000      0.000
-----------ATGCTTCATGTGCGGTAGT----------------------------------------      0.073      0.000      0.000
------------TGCTTCATGTGCGGTAGTAA--------------------------------------      0.073      0.000      0.000
-------------GCTTCATGTGCGGTAGTAAGT------------------------------------      0.220      0.200      0.000
---------------TTCATGTGCGGTAGTAAGT------------------------------------      0.294      0.100      0.000
-----------ATGCTTCATGTGCGGTAGTAAGT------------------------------------      0.073      0.100      0.000
----------------TCATGTGCGGTAGTAAGT------------------------------------      0.441      0.100      0.000
------------TGCTTCATGTGCGGTAGTAAGT------------------------------------      0.073      0.100      0.000
----------------TCATGTGCGGTAGTAAGTGT----------------------------------      4.332      2.501      0.408
-----------------CATGTGCGGTAGTAAGTGT----------------------------------      1.028      0.200      0.000
---------------TTCATGTGCGGTAGTAAGTGT----------------------------------      0.367      0.000      0.082
------------------ATGTGCGGTAGTAAGTGTA---------------------------------      0.073      0.200      0.000
----------------TCATGTGCGGTAGTAAGTGTA---------------------------------      3.891      2.801      0.408
---------------TTCATGTGCGGTAGTAAGTGTA---------------------------------      0.367      0.000      0.082
-----------------CATGTGCGGTAGTAAGTGTA---------------------------------     10.426      3.001      0.408
------------------ATGTGCGGTAGTAAGTGTAA--------------------------------      0.147      0.200      0.000
-----------------CATGTGCGGTAGTAAGTGTAA--------------------------------     15.271      5.801      2.365
----------------TCATGTGCGGTAGTAAGTGTAA--------------------------------      0.587      0.200      0.000
-------------------TGTGCGGTAGTAAGTGTAAG-------------------------------      0.147      0.000      0.000
-------------------TGTGCGGTAGTAAGTGTAAGT------------------------------     44.566     25.105      3.343
---------------------TGCGGTAGTAAGTGTAAGT------------------------------      0.734      0.200      0.000
------------------ATGTGCGGTAGTAAGTGTAAGT------------------------------      0.441      0.100      0.000
----------------------GCGGTAGTAAGTGTAAGT------------------------------      1.322      0.800      0.163
--------------------GTGCGGTAGTAAGTGTAAGT------------------------------     20.337      8.302      1.468
--------------------GTGCGGTAGTAAGTGTAAGTG-----------------------------      0.367      0.000      0.082
----------------------GCGGTAGTAAGTGTAAGTG-----------------------------      0.073      0.100      0.000
----------------------GCGGTAGTAAGTGTAAGTGG----------------------------      0.147      0.000      0.000
------------------------GGTAGTAAGTGTAAGTGG----------------------------      0.147      0.000      0.000
```

```
---------------------TGCGGTAGTAAGTGTAAGTGG----------------------------      0.147      0.000      0.000
------------------------GGTAGTAAGTGTAAGTGGC---------------------------      0.073      0.000      0.000
-----------------------CGGTAGTAAGTGTAAGTGGC---------------------------      0.073      0.000      0.000
----------------------GCGGTAGTAAGTGTAAGTGGC---------------------------      3.084      0.700      0.163
---------------------TGCGGTAGTAAGTGTAAGTGGC---------------------------      0.073      0.000      0.000
------------------------GGTAGTAAGTGTAAGTGGCG--------------------------      1.542      0.500      0.000
------------------------GGTAGTAAGTGTAAGTGGCGT-------------------------    267.764     85.917     15.085 miR_t77
---------------------------AGTAAGTGTAAGTGGCGT-------------------------      0.441      0.500      0.163
-------------------------GTAGTAAGTGTAAGTGGCGT-------------------------      1.175      0.500      0.082
-----------------------CGGTAGTAAGTGTAAGTGGCGT-------------------------      0.220      0.200      0.000
---------------------------AGTAAGTGTAAGTGGCGTA------------------------      0.881      0.200      0.082
------------------------GGTAGTAAGTGTAAGTGGCGTA------------------------      0.073      0.100      0.000
---------------------------AGTAAGTGTAAGTGGCGTAC-----------------------      3.084      1.800      0.245
-------------------------GTAGTAAGTGTAAGTGGCGTAC-----------------------      0.220      0.100      0.000
-----------------------------TAAGTGTAAGTGGCGTAC-----------------------      0.147      0.000      0.000
----------------------------GTAAGTGTAAGTGGCGTACA----------------------     11.821     13.903      1.468
---------------------------AGTAAGTGTAAGTGGCGTACA----------------------   2386.896    758.653    158.429
----------------------GCGGTAGTAAGTGTAAGTGGCGTACA----------------------      0.073      0.000      0.000
------------------------------AAGTGTAAGTGGCGTACA----------------------     12.114      4.701      1.223
-----------------------------TAAGTGTAAGTGGCGTACA----------------------     10.426      8.202      0.978
--------------------------TAGTAAGTGTAAGTGGCGTACA----------------------     13.730      2.200      0.326
-----------------------------TAAGTGTAAGTGGCGTACAG---------------------     63.655     22.405      5.218
----------------------------GTAAGTGTAAGTGGCGTACAG---------------------      1.101      1.000      0.000
------------------------------AAGTGTAAGTGGCGTACAG---------------------     20.484      6.101      1.468
---------------------------AGTAAGTGTAAGTGGCGTACAG---------------------      0.661      0.200      0.163
--------------------------TAGTAAGTGTAAGTGGCGTACAG---------------------      0.073      0.000      0.000
--------------------------TAGTAAGTGTAAGTGGCGTACAGT--------------------      7.856      0.000      0.245
---------------------------AGTAAGTGTAAGTGGCGTACAGT--------------------     20.044      2.200      2.038
-------------------------------AGTGTAAGTGGCGTACAGT--------------------     33.627     20.504      2.935
-----------------------------TAAGTGTAAGTGGCGTACAGT--------------------  14646.553   5386.188    970.227 miR_t58
--------------------------------GTGTAAGTGGCGTACAGT--------------------     10.132      4.001      1.223
----------------------------GTAAGTGTAAGTGGCGTACAGT--------------------    202.714     65.813     28.783
------------------------------AAGTGTAAGTGGCGTACAGT--------------------   3336.882    901.582    238.826
-----------------------------TAAGTGTAAGTGGCGTACAGTGGC-----------------      0.073      0.000      0.000
--------------------------------GTGTAAGTGGCGTACAGTGGC-----------------      0.294      0.000      0.000
----------------------------------GTAAGTGGCGTACAGTGGCGG---------------      0.073      0.000      0.000
---------------------------------TGTAAGTGGCGTACAGTGGCGG---------------      0.073      0.000      0.000
------------------------------------AAGTGGCGTACAGTGGCGGCC-------------      0.073      0.000      0.000
-------------------------------------AGTGGCGTACAGTGGCGGCC-------------      0.073      0.000      0.000
-------------------------------------AGTGGCGTACAGTGGCGGCCT------------      0.367      0.000      0.000
---------------------------------------TGGCGTACAGTGGCGGCCTT-----------      0.954      0.200      0.000
----------------------------------------GGCGTACAGTGGCGGCCTT-----------      0.073      0.000      0.000
-------------------------------------AGTGGCGTACAGTGGCGGCCTT-----------      0.147      0.000      0.000
--------------------------------------GTGGCGTACAGTGGCGGCCTT-----------      0.734      0.200      0.000
----------------------------------------GGCGTACAGTGGCGGCCTTGGT--------      0.147      0.000      0.082
---------------------------------------TGGCGTACAGTGGCGGCCTTGGT--------      0.073      0.000      0.000
------------------------------------------CGTACAGTGGCGGCCTTGGT--------      0.147      0.000      0.000
-----------------------------------------GCGTACAGTGGCGGCCTTGGT--------      1.101      0.800      0.000
---------------------------------------------ACAGTGGCGGCCTTGGTT-------      0.147      0.000      0.000
-------------------------------------------GTACAGTGGCGGCCTTGGTT-------      0.294      0.500      0.000
------------------------------------------CGTACAGTGGCGGCCTTGGTT-------      0.294      0.100      0.000
--------------------------------------------TACAGTGGCGGCCTTGGTTG------      0.073      0.000      0.000
-------------------------------------------GTACAGTGGCGGCCTTGGTTGT-----      0.220      0.000      0.000
---------------------------------------------ACAGTGGCGGCCTTGGTTGT-----      7.489      4.701      1.142
-----------------------------------------------AGTGGCGGCCTTGGTTGT-----      0.073      0.000      0.000
--------------------------------------------TACAGTGGCGGCCTTGGTTGT-----     13.656      2.400      0.408
----------------------------------------------CAGTGGCGGCCTTGGTTGT-----      0.147      0.100      0.000
-----------------------------------------------AGTGGCGGCCTTGGTTGTGC---      0.073      0.000      0.000
-----------------------------------------------AGTGGCGGCCTTGGTTGTGCT--      0.587      0.100      0.000
------------------------------------------------GTGGCGGCCTTGGTTGTGCT--      0.220      0.200      0.000

miRNA: miR_t10,miR_t90 chromosome_16 Start: 185024 End: 185180 Condition: Sulfur-deprivation
------------------------------------------------------------------TATCCAGCAAGCAACATCCACAT--------------------      0.441      2.801      0.326
------------------------------------------------------------------TATCCAGCAAGCAACATCCAC----------------------      0.661     10.902      3.588
------------------------------------------------------------------TATCCAGCAAGCAACATCCACA---------------------     18.208    140.028     32.942
------------------------------------------------------------------TATCCAGCAAGCAACATCCA-----------------------      0.147      1.900      0.408
-------------------------------------------------------------------ATCCAGCAAGCAACATCCACAT--------------------      0.367      4.501      0.408
-------------------------------------------------------------------ATCCAGCAAGCAACATCCACA---------------------      1.689     56.411      3.995 miR_t90*
-------------------------------------------------------------------ATCCAGCAAGCAACATCCAC----------------------      0.073      0.700      0.163
--------------------------------------------------------------------TCCAGCAAGCAACATCCACA---------------------      6.241     96.820     11.171
--------------------------------------------------------------------TCCAGCAAGCAACATCCACATA-------------------      0.220      8.502      1.386
--------------------------------------------------------------------TCCAGCAAGCAACATCCAC----------------------      0.000      1.400      0.163
--------------------------------------------------------------------TCCAGCAAGCAACATCCA-----------------------      0.000      1.100      0.082
--------------------------------------------------------------------TCCAGCAAGCAACATCCACAT--------------------      2.349     33.707      2.691
---------------------------------------------------------------------CCAGCAAGCAACATCCACATA-------------------      0.000      2.000      0.082
----------------------------------------------------------------------CAGCAAGCAACATCCACAT--------------------      0.000      1.000      0.082
----------------------------------------------------------------------CAGCAAGCAACATCCACATA-------------------      0.220      4.501      0.489
----------------------------------------------------------------------CAGCAAGCAACATCCACA---------------------      0.220      3.801      0.326
-----------------------------------------------------------------------AGCAAGCAACATCCACATAAG-----------------      0.000      0.000      0.082
--------------------------------------------------------------------------AAGCAACATCCACATAAGCCG--------------      0.000      0.000      0.082
---------------------------------------------------------------------------------ATCCACATAAGCCGGGGTTGG-------      0.000      0.100      0.163
----------------------------------------------------------------------------------TCCACATAAGCCGGGGTTGG-------      0.000      0.000      0.245
----------------------------------------------------------------------------------------TAAGCCGGGGTTGGTAGCAA-      0.441      1.400      1.386
----------------------------------------------------------------------------------------TAAGCCGGGGTTGGTAGCA--      0.220      0.400      0.571
----------------------------------------------------------------------------------------TAAGCCGGGGTTGGTAGC---      0.147      0.500      0.652
----------------------------------------------------------------------------------------TAAGCCGGGGTTGGTAGCAAC     10.279      9.202     21.445 miR_t10

--------------------------------------------------------------                                           CAA
                                                              ACGGTATCCAGCAAGCAACATCCACATAAGCCGGGGTTGGTAG   C
                                                              TGCCATAGGTCGTTCGTTGTAGGTGTATTCGGCCCCAACCATC   T
TTTTTTGGTAGGTGGCGGGGTTGTGCCCGGGTGGCCGGTTCCGGCGGGGTTGGTTGGTGTAG                                           TTT

--------------------------------------------------------------------------------GTAGGTGTATTCGGCCCCAA---------      0.147      0.200      0.245
----------------------------------------------------------------------------------AGGTGTATTCGGCCCCAA---------      0.000      0.000      0.082
-------------------------------------------------------------------------------TGTAGGTGTATTCGGCCCCAA---------      0.734      0.500      0.489
--------------------------------------------------------------------AGGTCGTTCGTTGTAGGTGTAT-------------------      0.073      0.000      0.082
---------------------------------------------------------------------GGTCGTTCGTTGTAGGTGTAT-------------------      0.220      0.200      0.326
-------------------------------------------------------------------TAGGTCGTTCGTTGTAGGTGTA--------------------      0.073      0.000      0.082
-------------------------------------------------------------------TAGGTCGTTCGTTGTAGGTGT---------------------     49.338     74.915    115.295
----------------------------------------------------------------------GTCGTTCGTTGTAGGTGT---------------------      0.661      0.900      1.794
-----------------------------------------------------------------CATAGGTCGTTCGTTGTAGGTGT---------------------      0.073      0.000      0.082
------------------------------------------------------------------ATAGGTCGTTCGTTGTAGGTGT---------------------      4.405      4.701     11.823
--------------------------------------------------------------------AGGTCGTTCGTTGTAGGTGT---------------------    115.857    147.630    237.196
---------------------------------------------------------------------GGTCGTTCGTTGTAGGTGT---------------------      1.836      2.601      4.892
--------------------------------------------------------------------AGGTCGTTCGTTGTAGGTG----------------------      0.294      0.500      0.489
-------------------------------------------------------------------TAGGTCGTTCGTTGTAGGTG----------------------      0.073      0.100      0.245
------------------------------------------------------------------ATAGGTCGTTCGTTGTAGGTG----------------------      0.000      0.000      0.082
-----------------------------------------------------------------CATAGGTCGTTCGTTGTAGGT-----------------------     17.694     20.104     38.323 miR_t90
----------------------------------------------------------------CCATAGGTCGTTCGTTGTAGGT-----------------------      1.395      1.700      2.283
--------------------------------------------------------------TGCCATAGGTCGTTCGTTGTAGGT-----------------------      0.073      0.100      0.082
--------------------------------------------------------------------AGGTCGTTCGTTGTAGGT-----------------------      0.587      0.900      1.060
------------------------------------------------------------------ATAGGTCGTTCGTTGTAGGT-----------------------     24.963     50.410     73.792
-------------------------------------------------------------------TAGGTCGTTCGTTGTAGGT-----------------------      0.587      1.700      2.365
---------------------------------------------------------------GCCATAGGTCGTTCGTTGTAGGT-----------------------      0.073      0.000      0.082
-----------------------------------------------------------------CATAGGTCGTTCGTTGTAGG------------------------      0.000      0.100      0.082
------------------------------------------------------------------ATAGGTCGTTCGTTGTAGG------------------------      0.000      0.100      0.245
-------------------------------------------------------------------TAGGTCGTTCGTTGTAGG------------------------      0.000      0.100      0.082
------------------------------------------------------------------ATAGGTCGTTCGTTGTAG-------------------------      0.000      0.200      0.245
-----------------------------------------------------------------CATAGGTCGTTCGTTGTAG-------------------------      0.000      0.000      0.163
---------------------------------------------------------TGTAGTGCCATAGGTCGTTCGTT-----------------------------      0.000      0.000      0.082
----------------------------------------------------------GTAGTGCCATAGGTCGTTCGT------------------------------      0.000      0.000      0.082
----TTGGTAGGTGGCGGGGTTGTGC-----------------------------------------------------------------------------------      0.000      0.000      0.082

miRNA: miR_t76,miR_t5 chromosome_14 Start: 3218700 End: 3218921 Condition: Phosphate-deficient
---------------------------------CCCGG--AGGTGC---CGG-AGCGGTTGGGGTT--------------------------------------------------------------      0.073      0.000      0.000
-----------------------------------------------------------------TGCGGGGCCCGCAGGTAGCTGC-----------------------------------------      2.570      1.500      3.262 miR_t76
------------------------------------------------------------------GCGGGGCCCGCAGGTAGCTGC-----------------------------------------      3.230     11.102      8.480
------------------------------------------------------------------GCGGGGCCCGCAGGTAGCT-------------------------------------------      0.073      0.600      0.326
------------------------------------------------------------------GCGGGGCCCGCAGGTAGCTG------------------------------------------      0.147      1.600      1.142
------------------------------------------------------------------GCGGGGCCCGCAGGTAGCTGCC----------------------------------------      0.073      0.300      0.082
-------------------------------------------------------------------CGGGGCCCGCAGGTAGCTG------------------------------------------      0.073      0.100      0.163
-------------------------------------------------------------------CGGGGCCCGCAGGTAGCTGC-----------------------------------------      2.790      4.501      2.038
-----------------------------------------------------------------------------AGGTAGCTGCCGTTGAGCCCG------------------------------      0.367      0.300      0.408
------------------------------------------------------------------------------------TGCCGTTGAGCCCGACGCTGC-----------------------      0.147      0.000      0.082
---------------------------------------------------------------------------------------CGTTGAGCCCGACGCTGCTG---------------------      0.587      2.300      0.652
---------------------------------------------------------------------------------------CGTTGAGCCCGACGCTGCTGC--------------------      3.451      8.902      3.017
---------------------------------------------------------------------------------------CGTTGAGCCCGACGCTGCTGCC-------------------      0.294      0.800      0.082
---------------------------------------------------------------------------------------CGTTGAGCCCGACGCTGCT----------------------      0.073      1.400      0.652
-----------------------------------------------------------------------------------------TTGAGCCCGACGCTGCTGCC-------------------      0.220      0.200      0.000
-----------------------------------------------------------------------------------------TTGAGCCCGACGCTGCTGC--------------------      0.220      0.000      0.082
------------------------------------------------------------------------------------------TGAGCCCGACGCTGCTGC--------------------      0.073      0.000      0.000

-----------------------------    C    --      ---   -   G                        A     C                    C    CG C         G
                             TGCA CCGG  AGGTGC   CGG AGC GTTGGGGTTGCGGGGCCCGCAGGT GCTGC GTTGAGCCCGACGCTGCTGC GCCG  C GGTGCTGCT C
                             ACGT GGCC  TCCACG   GCC TCG CGACCCCGACGCCCTGGGCGTCCA CGACG CAGCTCGGGCTGCGACGACG CGGC  G CCACGACGG C
TGGGCAGGGGCAGGGACGTCGGCCAGTCC    C    AG      TCA   A   A                        C     C                    T    CA T         C

----------------------------------------------------------------------------------------------------GCGACGACGTCGGCCAGTCCA-------      0.073      0.100      0.000
-------------------------------------------------------------------------------------------------GCTGCGACGACGTCGGCCAGT----------      0.073      0.000      0.000
----------------------------------------------------------------------------------------CCAGCTCGGGCTGCGACGACG-------------------      0.147      0.300      0.815
---------------------------------------------------------------------------------------GCCAGCTCGGGCTGCGACGAC--------------------      0.073      0.000      0.000
----------------------------------------------------------------------------------------CCAGCTCGGGCTGCGACGAC--------------------      0.073      0.100      0.163
--------------------------------------------------------------------------------------CGCCAGCTCGGGCTGCGACGAC--------------------      0.661      1.200      1.060
-------------------------------------------------------------------------------------ACGCCAGCTCGGGCTGCGACGAC--------------------      0.147      0.300      0.163
--------------------------------------------------------------------------------------CGCCAGCTCGGGCTGCGACGA---------------------    795.730    983.699   1318.070 miR_t5
-------------------------------------------------------------------------------------ACGCCAGCTCGGGCTGCGACGA---------------------    205.944    134.227    231.325
------------------------------------------------------------------------------------GACGCCAGCTCGGGCTGCGACGA---------------------      0.073      0.000      0.000
-----------------------------------------------------------------------------------------CAGCTCGGGCTGCGACGA---------------------      0.441      1.000      0.815
----------------------------------------------------------------------------------------CCAGCTCGGGCTGCGACGA---------------------      3.304      8.802      8.725
---------------------------------------------------------------------------------------GCCAGCTCGGGCTGCGACGA---------------------      8.370     11.102     16.634
--------------------------------------------------------------------------------------CGCCAGCTCGGGCTGCGACG----------------------      3.744      4.101      6.034
-------------------------------------------------------------------------------------ACGCCAGCTCGGGCTGCGACG----------------------      2.570      3.101      5.137
----------------------------------------------------------------------------------------CCAGCTCGGGCTGCGACG----------------------      0.073      0.000      0.082
------------------------------------------------------------------------------------GACGCCAGCTCGGGCTGCGAC-----------------------      0.073      0.200      0.408
--------------------------------------------------------------------------------------CGCCAGCTCGGGCTGCGAC-----------------------      0.073      0.100      0.163
-------------------------------------------------------------------------------------ACGCCAGCTCGGGCTGCGA------------------------      0.147      0.200      0.082
```

```
-----------------------------------------------------------------------------------CGACGCCAGCTCGGGCTGCGA------------------------      0.661      1.100      1.305
--------------------------------------------------------------------------------------CGCCAGCTCGGGCTGCGA------------------------      0.220      0.400      0.734
---------------------------------------------------------------------------------ACCGACGCCAGCTCGGGCT----------------------------      0.073      0.000      0.000
----------------------------------------------------------------CGACGCCCTGGGCGTCCACCGA------------------------------------------      1.028      1.700      1.631 miR_t76*
-----------------------------------------------------------------GACGCCCTGGGCGTCCACCGA------------------------------------------      2.937      3.801      3.425
----------------------------------------------------------CGACCCCGACGCCCTGGGCGT-------------------------------------------------      0.073      0.000      0.000
-----CAGGGGCAGGGACGTCGGCCAGT----------------------------------------------------------------------------------------------------      0.147      0.000      0.408

miRNA: miR_t8 chromosome_4 Start: 3694773 End: 3695093 Condition: Phosphate-deficient
-----------------------------------------------------------------------------------------------------------------------------------------------------------------------------------------------------------------------CTTAGTCGTCACAAGGCGTG--------------------      0.220      0.100      0.082
----------------------------------------------------------------------------------------------------------------------------------------------------------------------------------------------------------------------ACTTAGTCGTCACAAGGCGTGT-------------------      0.000      0.000      0.082
----------------------------------------------------------------------------------------------------------------------------------------------------------------------------------------------------------------------ACTTAGTCGTCACAAGGCGTG--------------------     24.376     27.706     63.111
----------------------------------------------------------------------------------------------------------------------------------------------------------------------------------------------------------------------ACTTAGTCGTCACAAGGCG----------------------      0.220      0.000      0.245
---------------------------------------------------------------------------------------------------------------------------------------------------------------------------------------------------------------------AACTTAGTCGTCACAAGGCG----------------------      0.073      0.100      0.245
---------------------------------------------------------------------------------------------------------------------------------------------------------------------------------------------------------------------AACTTAGTCGTCACAAGGCGTG--------------------      0.147      0.300      0.978
---------------------------------------------------------------------------------------------------------------------------------------------------------------------------------------------------------------------AACTTAGTCGTCACAAGGCGT---------------------      0.220      0.300      0.245
--------------------------------------------------------------------------------------------------------------------------------------------------------------------------------------------------------------------TAACTTAGTCGTCACAAGGCG----------------------      5.066     10.602     23.157 miR_t8
--------------------------------------------------------------------------------------------------------------------------------------------------------------------------------------------------------------------TAACTTAGTCGTCACAAGGCGTG--------------------      0.294      0.100      0.571
--------------------------------------------------------------------------------------------------------------------------------------------------------------------------------------------------------------------TAACTTAGTCGTCACAAGG------------------------      0.147      0.000      0.245
--------------------------------------------------------------------------------------------------------------------------------------------------------------------------------------------------------------------TAACTTAGTCGTCACAAG-------------------------      0.147      0.100      0.082
--------------------------------------------------------------------------------------------------------------------------------------------------------------------------------------------------------------------TAACTTAGTCGTCACAAGGCGT---------------------      0.367      0.600      0.734
-----------------------------------------------------------------------------------------------------------------------------------------------------------------------------------------------------------------TGGTAACTTAGTCGTCACAAG-------------------------      0.073      0.100      0.408
-------------------------------------------------------------------------------------------------------------------------------------------------------------------------------------------------------CGAGGGAAAATGGTAACTTAG-----------------------------------      0.220      0.200      0.326
--------------------------------------------------------------------------------------------------------------------------------------------------------------------------------------------------TGTAACGAGGGAAAATGGTAA----------------------------------------      0.073      0.000      0.163
--------------------------------------------------------------------------------------------------------------------------------------------------------------------------------------------------TGTAACGAGGGAAAATGGTA-----------------------------------------      0.147      0.100      0.489

----------------------------------------------------------------------------------------------------------------------------------------------------------------------------------------------GTCC     C A                       A                        A  T
                                                                                                                                                                                                  TGTAA G GGGAAAATGGTAACTTAGTCGTC CAAGGCGTGTATCACCCGTGTTAC GG G
                                                                                                                                                                                                  ACATT C CCCTTTTACCATTGAATCAGTAG GTTCCGCACATAGTGGGTACAATG CC /
AGGTCGGTCGAGAAGGGCCCGGCGTCGAAGCTGGGGTCCCAAGGCCCGCCTCACGGAGGAAGGAACCCCTCGCGTACGGACCCGCAGCACGACGTCCCCGACACCCGCCGGCGCGTCGTCGGCACTTCGACCACCTGGACACCGCCCCCGGCCTCCCGTTCCTCCGCGACGTCCACGCCAACCCAGAAGCCTGT     A G                       G                        C  -

----CGGTCGAGAAGGGCCCGGCGT------------------------------------------------------------------------------------------------------------------------------------------------------------------------------------------------------------------------------      0.000      0.000      0.082

miRNA: miR_t13,miR_t109 chromosome_10 Start: 3399866 End: 3400007 Condition: Nutrient-replete
-------CCGATAAGAAGGAGCCGTAAGG-------------------------------------------      0.367      2.400      0.326
-------CCGATAAGAAGGAGCCGTAAGGT------------------------------------------      0.073      0.000      0.000
--------CGATAAGAAGGAGCCGTAAGG-------------------------------------------      5.947     50.710     13.372
--------CGATAAGAAGGAGCCGTAAG--------------------------------------------      0.073      4.401      1.142
--------CGATAAGAAGGAGCCGTAAGGT------------------------------------------      0.587      5.501      1.386
---------GATAAGAAGGAGCCGTAAGGT------------------------------------------      3.157      5.101      5.382 miR_t109*
---------GATAAGAAGGAGCCGTAAGGTA-----------------------------------------      0.220      0.000      0.163
---------GATAAGAAGGAGCCGTAAGG-------------------------------------------      4.185     12.903      5.789
---------GATAAGAAGGAGCCGTAAG--------------------------------------------      0.661      0.200      0.082
---------GATAAGAAGGAGCCGTAA---------------------------------------------      0.367      0.800      0.571
----------ATAAGAAGGAGCCGTAAGGT------------------------------------------     83.993    116.123    114.317
----------ATAAGAAGGAGCCGTAAG--------------------------------------------      0.147      0.200      0.163
----------ATAAGAAGGAGCCGTAAGG-------------------------------------------      3.524      8.602      4.240
----------ATAAGAAGGAGCCGTAAGGTA-----------------------------------------      4.919      9.302      5.952
-----------TAAGAAGGAGCCGTAAGGTA-----------------------------------------      2.203      4.901      5.545
-----------TAAGAAGGAGCCGTAAGGT------------------------------------------     17.254     38.908     26.092
-----------TAAGAAGGAGCCGTAAGGTAC----------------------------------------      2.863      3.701      3.180
-----------TAAGAAGGAGCCGTAAGG-------------------------------------------      2.717      7.602      2.691
-----------TAAGAAGGAGCCGTAAGGTACC---------------------------------------      0.073      0.100      0.000
------------AAGAAGGAGCCGTAAGGTAC----------------------------------------      0.220      0.400      0.163
------------AAGAAGGAGCCGTAAGGT------------------------------------------      0.220      0.100      0.245
-------------AGAAGGAGCCGTAAGGTACCG--------------------------------------      0.147      0.000      0.000
-------------AGAAGGAGCCGTAAGGTACC---------------------------------------      0.147      0.100      0.326
---------------AAGGAGCCGTAAGGTACCGGG------------------------------------      0.147      0.100      0.000
------------------GAGCCGTAAGGTACCGGGCG----------------------------------      0.073      0.000      0.082
-------------------AGCCGTAAGGTACCGGGCGTG--------------------------------      3.524      3.501      4.485
----------------------CGTAAGGTACCGGGCGTGGGG-----------------------------      0.147      0.100      0.000
-----------------------GTAAGGTACCGGGCGTGGGG-----------------------------      0.073      0.000      0.000
------------------------TAAGGTACCGGGCGTGGGGA----------------------------      0.073      0.000      0.000
------------------------TAAGGTACCGGGCGTGGGGAGGGC------------------------      0.073      0.000      0.000
------------------------TAAGGTACCGGGCGTGGGGAG---------------------------      2.496      1.700      2.120
------------------------TAAGGTACCGGGCGTGGGGAGG--------------------------     10.426      8.402     13.780
-------------------------AAGGTACCGGGCGTGGGG-----------------------------      0.073      0.000      0.000
-------------------------AAGGTACCGGGCGTGGGGAGG--------------------------      0.881      0.500      0.652
-------------------------AAGGTACCGGGCGTGGGGA----------------------------      0.073      0.000      0.000
--------------------------AGGTACCGGGCGTGGGGAGGG-------------------------      3.377      2.100      2.854
--------------------------AGGTACCGGGCGTGGGGAGG--------------------------      0.514      0.300      0.571
--------------------------AGGTACCGGGCGTGGGGAGGGCAG----------------------      0.220      0.000      0.082
--------------------------AGGTACCGGGCGTGGGGAGGGCA-----------------------      0.147      0.000      0.163
--------------------------AGGTACCGGGCGTGGGGAGGGC------------------------      1.615      0.300      0.897
---------------------------GGTACCGGGCGTGGGGAGGGC------------------------      2.790      0.400      1.142
---------------------------GGTACCGGGCGTGGGGAGGGCAG----------------------      0.073      0.000      0.000
---------------------------GGTACCGGGCGTGGGGAGGGCA-----------------------      0.294      0.000      0.000
----------------------------GTACCGGGCGTGGGGAGGGCA-----------------------      5.947      1.000      2.446
----------------------------GTACCGGGCGTGGGGAGGGCAG----------------------      2.570      0.900      1.468
----------------------------GTACCGGGCGTGGGGAGGGCAGGG--------------------      0.073      0.000      0.000
----------------------------GTACCGGGCGTGGGGAGGGC------------------------      1.762      0.700      0.897
----------------------------GTACCGGGCGTGGGGAGGG-------------------------      0.073      0.000      0.000
----------------------------GTACCGGGCGTGGGGAGGGCAGG---------------------      3.818      0.200      1.957
-----------------------------TACCGGGCGTGGGGAGGGCAG----------------------    958.576   1065.515   1124.742
-----------------------------TACCGGGCGTGGGGAGGG-------------------------      5.653      4.301      4.240
-----------------------------TACCGGGCGTGGGGAGGGCA-----------------------     29.515     51.610     47.211
-----------------------------TACCGGGCGTGGGGAGGGCAGGGG-------------------      0.514      0.100      0.082
-----------------------------TACCGGGCGTGGGGAGGGCAGGGGC------------------      0.220      0.100      0.000
-----------------------------TACCGGGCGTGGGGAGGGCAGG---------------------  11636.759   5413.093   9096.437 miR_t13
-----------------------------TACCGGGCGTGGGGAGGGC------------------------     13.509     14.903     14.025
-----------------------------TACCGGGCGTGGGGAGGGCAGGG--------------------     26.138      4.701     21.771
------------------------------ACCGGGCGTGGGGAGGGCAGG---------------------    821.721    203.741    282.123
------------------------------ACCGGGCGTGGGGAGGGCAGGGG-------------------      0.147      0.000      0.000
------------------------------ACCGGGCGTGGGGAGGGCAG----------------------     37.004     15.503     13.862
------------------------------ACCGGGCGTGGGGAGGGCAGGG--------------------      2.129      0.300      0.571
------------------------------ACCGGGCGTGGGGAGGGC------------------------      7.048      3.201      4.403
------------------------------ACCGGGCGTGGGGAGGGCA-----------------------     32.452     17.404     20.385
-------------------------------CCGGGCGTGGGGAGGGCAG----------------------      4.038      0.400      1.223
-------------------------------CCGGGCGTGGGGAGGGCA-----------------------      2.056      4.201      1.875
-------------------------------CCGGGCGTGGGGAGGGCAGGG--------------------      2.717      0.600      0.978
-------------------------------CCGGGCGTGGGGAGGGCAGG---------------------     21.659      9.702      6.115
--------------------------------CGGGCGTGGGGAGGGCAGGG--------------------      0.147      0.000      0.245
--------------------------------CGGGCGTGGGGAGGGCAGG---------------------      2.496      2.200      1.549
--------------------------------CGGGCGTGGGGAGGGCAGGGG-------------------      2.423      0.700      1.060
--------------------------------CGGGCGTGGGGAGGGCAG----------------------      0.367      0.200      0.326
---------------------------------GGGCGTGGGGAGGGCAGGGGC------------------      0.734      0.500      0.815
---------------------------------GGGCGTGGGGAGGGCAGGGGCA-----------------      0.073      0.000      0.000
---------------------------------GGGCGTGGGGAGGGCAGG---------------------      1.248      1.100      0.897
---------------------------------GGGCGTGGGGAGGGCAGGGG-------------------      0.220      0.000      0.000
-----------------------------------GCGTGGGGAGGGCAGGGGC------------------      0.073      0.000      0.000
------------------------------------CGTGGGGAGGGCAGGGGCAGG---------------      0.073      0.000      0.000
---------------------------------------------GGCAGGGGCAGGGACGGGGAT------      0.147      0.000      0.000
----------------------------------------------GCAGGGGCAGGGACGGGGATC-----      0.808      1.100      0.815
--------------------------------------------------GGGCAGGGACGGGGATCA----      0.073      3.301      0.408
---------------------------------------------------GGCAGGGACGGGGATCAGGG-      0.294      0.500      0.163
---------------------------------------------------GGCAGGGACGGGGATCAGG--      0.220      3.301      0.734
---------------------------------------------------GGCAGGGACGGGGATCAG---      0.441      0.800      0.815
----------------------------------------------------GCAGGGACGGGGATCAGG--      0.147      1.200      0.489

---GA                                                                GG
     TGCCGATAAGAAGGAGCCGTAAGGTACCGGGCGTGGGGAGGGCAGGGGCAGGGACGGGGATCAG  G
     ACGGCTATTCTTCCTCGGCATTCCATGGCCCGCGCCCCTCCCGTCCCCGTCCCTGCCCCTAGTC  G
GGGAC                                                                GG

----------------------------CCATGGCCCGCGCCCCTCCCGT----------------------      5.213      3.301      1.631 miR_t13*
-------------------------------TGGCCCGCGCCCCTCCCGT----------------------      0.073      0.000      0.000
---------------------------TCCATGGCCCGCGCCCCTCCCGT----------------------      0.514      0.300      0.082
------------------------------ATGGCCCGCGCCCCTCCCGT----------------------      0.073      0.100      0.163
-----------------------------CATGGCCCGCGCCCCTCCCGT----------------------      6.094      2.701      1.712
----------------------------CCATGGCCCGCGCCCCTCCCG-----------------------      0.367      0.100      0.245
--------------------------TTCCATGGCCCGCGCCCCTCC-------------------------      0.073      0.000      0.000
------------------CCTCGGCATTCCATGGCCCGC---------------------------------      0.073      0.000      0.163
----------CTATTCTTCCTCGGCATTCCAT----------------------------------------      0.073      0.100      0.163
-----------TATTCTTCCTCGGCATTCCAT----------------------------------------      4.038      8.702      3.180
-------------TTCTTCCTCGGCATTCCAT----------------------------------------      0.073      0.000      0.000
------------ATTCTTCCTCGGCATTCCAT----------------------------------------      9.838     12.603      8.317
----------CTATTCTTCCTCGGCATTCC------------------------------------------      0.073      0.000      0.000
--------GGCTATTCTTCCTCGGCATTC-------------------------------------------      1.836      1.200      0.897
---------GCTATTCTTCCTCGGCATTC-------------------------------------------      0.220      0.400      0.326
-----------TATTCTTCCTCGGCATTC-------------------------------------------      0.073      0.000      0.000
---------GCTATTCTTCCTCGGCATT--------------------------------------------     12.775      7.702     10.682
-----CACGGCTATTCTTCCTCGGCATT--------------------------------------------      0.367      1.000      0.571
--------GGCTATTCTTCCTCGGCATT--------------------------------------------    216.223    243.649    223.171
-------CGGCTATTCTTCCTCGGCATT--------------------------------------------     18.942     28.506     20.629 miR_t109
----------CTATTCTTCCTCGGCATT--------------------------------------------      1.101      1.500      0.489
------ACGGCTATTCTTCCTCGGCATT--------------------------------------------     75.036     97.320     96.623
---------GCTATTCTTCCTCGGCAT---------------------------------------------      0.294      0.600      0.082
------ACGGCTATTCTTCCTCGGCAT---------------------------------------------     16.666     40.508     22.423
--------GGCTATTCTTCCTCGGCAT---------------------------------------------      6.681     11.902     10.437
-------CGGCTATTCTTCCTCGGCAT---------------------------------------------      1.762      8.602      1.223
--------GGCTATTCTTCCTCGGCA----------------------------------------------      0.441      0.600      0.163
------ACGGCTATTCTTCCTCGGCA----------------------------------------------      0.073      1.300      0.163
------ACGGCTATTCTTCCTCGGC-----------------------------------------------      0.147      0.000      0.000
------ACGGCTATTCTTCCTCGG------------------------------------------------      0.147      0.000      0.163

miRNA: miR_t122,miR_t70 chromosome_9 Start: 6518812 End: 6519093 Condition: Nutrient-replete
-------------------------------------------------------------------------------------TGCGCACAGCCCTTAGAGCCTG--------------------------------------      0.073      0.100      0.000
-------------------------------------------------------------------------------------TGCGCACAGCCCTTAGAGCCT---------------------------------------      0.661      0.100      0.245
-------------------------------------------------------------------------------------TGCGCACAGCCCTTAGAGCC----------------------------------------      0.367      0.100      0.000
------------------------------------------------------------------------------------CTGCGCACAGCCCTTAGAGCC----------------------------------------      0.073      0.000      0.000
-----------------------------------------------------------------------------------GCTGCGCACAGCCCTTAGAG------------------------------------------      0.073      0.000      0.000
----------------------------------------------------------------------------------AGCTGCGCACAGCCCTTAGAG------------------------------------------      0.954      0.800      0.245
------------------------------------------------------------------------------GAGCAGCTGCGCACAGCCCTT----------------------------------------------      0.147      0.000      0.000
------------------------------------------------------------------------------GAGCAGCTGCGCACAGCCCT-----------------------------------------------      0.073      0.000      0.000
-----------------------------------------------------------------------------GGAGCAGCTGCGCACAGCCCT-----------------------------------------------      0.073      0.000      0.000
---------------------------------------------------------------------------CGGGAGCAGCTGCGCACAGC--------------------------------------------------      0.073      0.000      0.082
```

```
---------------------------------------------------------------------------CGGGAGCAGCTGCGCACAGCCC------------------------------------------------      0.147      0.300      0.000
---------------------------------------------------------------------------CGGGAGCAGCTGCGCACAGCCCT-----------------------------------------------      0.220      0.200      0.000
--------------------------------------------------------------------------GCGGGAGCAGCTGCGCACAGC--------------------------------------------------      0.073      0.000      0.000
------------------------------------------------------------------------CGGCGGGAGCAGCTGCGCACA----------------------------------------------------      1.175      1.100      0.245
------------------------------------------------------------------------CGGCGGGAGCAGCTGCGCAC-----------------------------------------------------      0.514      0.400      0.082
------------------------------------------------------------------------CGGCGGGAGCAGCTGCGCACAG---------------------------------------------------      0.073      0.000      0.000
------------------------------------------------------------------------CGGCGGGAGCAGCTGCGCACAGC--------------------------------------------------      0.073      0.000      0.000
-------------------------------------------------------ACGCGGACCCGGCGCAGCGGCG--------------------------------------------------------------------      2.570      0.800      0.815
------------------------------------------------------GACGCGGACCCGGCGCAG-------------------------------------------------------------------------      1.322      0.200      0.652
-----------------------------------------------------GGACGCGGACCCGGCGCAG-------------------------------------------------------------------------     14.464     10.702      6.360
-----------------------------------------------------GGACGCGGACCCGGCGCA--------------------------------------------------------------------------      0.147      0.100      0.000
-----------------------------------------------------GGACGCGGACCCGGCGCAGC------------------------------------------------------------------------      0.220      0.100      0.163
----------------------------------------------------TGGACGCGGACCCGGCGCAGCGG----------------------------------------------------------------------      2.276      1.100      1.223
----------------------------------------------------TGGACGCGGACCCGGCGCAG-------------------------------------------------------------------------    543.604    371.975    233.282
----------------------------------------------------TGGACGCGGACCCGGCGCAGCG-----------------------------------------------------------------------      4.625      3.801      1.794
----------------------------------------------------TGGACGCGGACCCGGCGCA--------------------------------------------------------------------------      0.734      0.600      0.408
----------------------------------------------------TGGACGCGGACCCGGCGC---------------------------------------------------------------------------      0.294      0.500      0.163
----------------------------------------------------TGGACGCGGACCCGGCGCAGC------------------------------------------------------------------------     40.675     26.005     21.363
---------------------------------------------------TTGGACGCGGACCCGGCGC---------------------------------------------------------------------------      3.818      4.701      1.875
---------------------------------------------------TTGGACGCGGACCCGGCGCAGC------------------------------------------------------------------------      4.112      6.501      2.038
---------------------------------------------------TTGGACGCGGACCCGGCGCAGCGG----------------------------------------------------------------------      0.073      0.300      0.000
---------------------------------------------------TTGGACGCGGACCCGGCGCAG-------------------------------------------------------------------------   6052.187   4847.579   2113.723 miR_t122
---------------------------------------------------TTGGACGCGGACCCGGCGCAGCGGCG--------------------------------------------------------------------      0.073      0.000      0.000
---------------------------------------------------TTGGACGCGGACCCGGCG----------------------------------------------------------------------------     17.841     15.303      6.034
---------------------------------------------------TTGGACGCGGACCCGGCGCAGCG-----------------------------------------------------------------------      0.147      0.400      0.000
---------------------------------------------------TTGGACGCGGACCCGGCGCA--------------------------------------------------------------------------     29.148     20.404     15.492
-------------------------------------------------AGTTGGACGCGGACCCGGCGCAG-------------------------------------------------------------------------      0.147      0.300      0.082
-------------------------------------------------AGTTGGACGCGGACCCGGC-----------------------------------------------------------------------------      0.073      0.000      0.000
-------------------------------------------------AGTTGGACGCGGACCCGGCGC---------------------------------------------------------------------------      0.661      0.400      0.489
------------------------------------------------AAGTTGGACGCGGACCCGGCGC---------------------------------------------------------------------------      2.203      1.000      0.734
------------------------------------------------AAGTTGGACGCGGACCCGGCG----------------------------------------------------------------------------      8.150      4.701      4.158
------------------------------------------------AAGTTGGACGCGGACCCGGCGCAG-------------------------------------------------------------------------      0.220      0.300      0.163
------------------------------------------------AAGTTGGACGCGGACCCGGC-----------------------------------------------------------------------------      0.881      1.100      0.408
-----------------------------------------------CAAGTTGGACGCGGACCCGGC-----------------------------------------------------------------------------      0.073      0.100      0.000
-------------------------------------------GCACCAAGTTGGACGCGGA-----------------------------------------------------------------------------------      0.073      0.600      0.082
-------------------------------------------GCACCAAGTTGGACGCGGACC---------------------------------------------------------------------------------      0.147      1.300      0.734
-------------------------------------------GCACCAAGTTGGACGCGG------------------------------------------------------------------------------------      0.441      0.300      0.000
-------------------------------------------GCACCAAGTTGGACGCGGAC----------------------------------------------------------------------------------      0.147      0.100      0.000
------------------------------------------CGCACCAAGTTGGACGCGGA-----------------------------------------------------------------------------------      0.147      0.100      0.000
------------------------------------------CGCACCAAGTTGGACGCGG------------------------------------------------------------------------------------      0.147      0.100      0.000
-----------------------------------------ACGCACCAAGTTGGACGCGGA-----------------------------------------------------------------------------------     24.890     13.903     18.672
-----------------------------------------ACGCACCAAGTTGGACGCGG------------------------------------------------------------------------------------     79.955     39.208     49.902
-----------------------------------------ACGCACCAAGTTGGACGCGGACC---------------------------------------------------------------------------------      0.073      0.000      0.000
-----------------------------------------ACGCACCAAGTTGGACGCG-------------------------------------------------------------------------------------      0.220      0.200      0.489
-----------------------------------------ACGCACCAAGTTGGACGCGGAC----------------------------------------------------------------------------------      1.101      1.200      0.652
----------------------------------------AACGCACCAAGTTGGACGCGG------------------------------------------------------------------------------------     23.421     11.002     15.655
----------------------------------------AACGCACCAAGTTGGACG---------------------------------------------------------------------------------------      0.073      0.000      0.000
----------------------------------------AACGCACCAAGTTGGACGCGGA-----------------------------------------------------------------------------------      0.441      0.000      0.082
----------------------------------------AACGCACCAAGTTGGACGC--------------------------------------------------------------------------------------      0.073      0.000      0.000
----------------------------------------AACGCACCAAGTTGGACGCG-------------------------------------------------------------------------------------      0.294      0.100      0.408
--------------------------------------GCAACGCACCAAGTTGGACG---------------------------------------------------------------------------------------      0.220      0.400      0.326
-------------------------------------CGCAACGCACCAAGTTGGACG---------------------------------------------------------------------------------------      0.073      0.100      0.000
--------------------------------GGCACCGCAACGCACCAAG----------------------------------------------------------------------------------------------      0.073      0.200      0.000
-------------------------------AGGCACCGCAACGCACCAAG----------------------------------------------------------------------------------------------      1.175      1.600      0.652
------------------------------TAGGCACCGCAACGCACCAA-----------------------------------------------------------------------------------------------      0.147      0.400      0.082
------------------------------TAGGCACCGCAACGCACCAAG----------------------------------------------------------------------------------------------    106.019     99.020     52.266
------------------------------TAGGCACCGCAACGCACCAAGT---------------------------------------------------------------------------------------------      6.167      4.701      1.875
------------------------------TAGGCACCGCAACGCACCA------------------------------------------------------------------------------------------------      0.073      0.100      0.082
---------------------AGGCCACGCTAGGCACCGCA--------------------------------------------------------------------------------------------------------      0.073      0.000      0.000
--------------------TAGGCCACGCTAGGCACCGCA--------------------------------------------------------------------------------------------------------      6.094      5.901      3.343
--------------------TAGGCCACGCTAGGCACCGC---------------------------------------------------------------------------------------------------------      0.587      0.100      0.245
--------------------TAGGCCACGCTAGGCACCGCAA-------------------------------------------------------------------------------------------------------      0.220      0.000      0.082
-----------------ATGTAGGCCACGCTAGGCACC-----------------------------------------------------------------------------------------------------------      0.073      0.100      0.000
-----------------ATGTAGGCCACGCTAGGCAC------------------------------------------------------------------------------------------------------------      0.073      0.000      0.000
-------------AGCGATGTAGGCCACGCTAGG---------------------------------------------------------------------------------------------------------------      0.147      0.600      0.734
------------AAGCGATGTAGGCCACGCTAG----------------------------------------------------------------------------------------------------------------      0.954      0.200      0.163
--------TTCCAAGCGATGTAGGCCACGC-------------------------------------------------------------------------------------------------------------------      0.661      0.600      0.326
--------TTCCAAGCGATGTAGGCCACG--------------------------------------------------------------------------------------------------------------------      1.689      1.600      1.468
--------TTCCAAGCGATGTAGGCCACGCT------------------------------------------------------------------------------------------------------------------      0.147      0.000      0.000

---    -    A                                                           C  C                       A         GG     CCT      C  C-      A  T   T
   CAGG TTCC AGCGATGTAGGCCACGCTAGGCACCGCAACGCACCAAGTTGGACGCGGACCCGGCGCAG GG GGGAGCAGCTGCGCACAGCCCTT GAGCCTGTGG  CTGA   GACGCT TT  GAAGAC TC TCC A
   GTCC AGGG TCGCTACATCCGGTGCGATCCGTGGCGTTGCGTGGTTCAACCTGCGCCTGGGCCGCGTC TC TCCTCGTCGACGCGTGTCGGGAA CTCGGACATC  GACT   TTGTGG AG  CTTTTG AG AGG /
CTG    C    C                                                           T  C                       A         G-     T--      C  TA      G  C   -

------CCCAGGGCTCGCTACATCCG-----------------------------------------------------------------------------------------------------------------------      0.073      0.000      0.000
------CCCAGGGCTCGCTACATCCGG----------------------------------------------------------------------------------------------------------------------      0.587      0.000      0.489
--------CAGGGCTCGCTACATCCGGT---------------------------------------------------------------------------------------------------------------------      0.073      0.200      0.000
-------CCAGGGCTCGCTACATCCGGT---------------------------------------------------------------------------------------------------------------------      0.808      2.400      0.978
-----TCCCAGGGCTCGCTACATCCGGT---------------------------------------------------------------------------------------------------------------------      0.073      0.100      0.082
------CCCAGGGCTCGCTACATCCGGT---------------------------------------------------------------------------------------------------------------------     43.098     39.208     30.985
-------CCAGGGCTCGCTACATCCGGTG--------------------------------------------------------------------------------------------------------------------      0.147      0.300      0.082
------CCCAGGGCTCGCTACATCCGGTG--------------------------------------------------------------------------------------------------------------------      0.147      0.400      0.000
-----------GGCTCGCTACATCCGGTGCGA-----------------------------------------------------------------------------------------------------------------      0.073      0.000      0.082
-----------GGCTCGCTACATCCGGTGCGAT----------------------------------------------------------------------------------------------------------------      0.073      0.000      0.000
------------GCTCGCTACATCCGGTGCGAT----------------------------------------------------------------------------------------------------------------      0.220      0.300      0.082
----------------------TCCGGTGCGATCCGTGGCGT-------------------------------------------------------------------------------------------------------      0.073      0.100      0.000
---------------------ATCCGGTGCGATCCGTGGCGT-------------------------------------------------------------------------------------------------------      0.073      0.100      0.082
----------------------TCCGGTGCGATCCGTGGCGTT------------------------------------------------------------------------------------------------------      0.073      0.000      0.082
---------------------------TGCGATCCGTGGCGTTGCGT--------------------------------------------------------------------------------------------------      0.147      0.000      0.000
-------------------------GGTGCGATCCGTGGCGTTGCGT--------------------------------------------------------------------------------------------------      0.073      0.100      0.000
--------------------------GTGCGATCCGTGGCGTTGCGT--------------------------------------------------------------------------------------------------      0.147      0.000      0.163
------------------------------GATCCGTGGCGTTGCGTG-------------------------------------------------------------------------------------------------      0.073      0.000      0.000
----------------------------GCGATCCGTGGCGTTGCGTGG------------------------------------------------------------------------------------------------      0.073      0.000      0.000
----------------------------GCGATCCGTGGCGTTGCGTGGT-----------------------------------------------------------------------------------------------      0.294      0.500      0.163
-----------------------------CGATCCGTGGCGTTGCGTGGT-----------------------------------------------------------------------------------------------      4.772      4.201      3.506
------------------------------GATCCGTGGCGTTGCGTGGT-----------------------------------------------------------------------------------------------      1.542      0.900      0.897
------------------------------GATCCGTGGCGTTGCGTGGTT----------------------------------------------------------------------------------------------      0.734      0.500      0.489
---------------------------------CCGTGGCGTTGCGTGGTTCAA-------------------------------------------------------------------------------------------      0.294      0.100      0.245
-------------------------------------GGCGTTGCGTGGTTCAACCT----------------------------------------------------------------------------------------      0.220      0.100      0.082
--------------------------------------------------TCAACCTGCGCCTGGGCCG----------------------------------------------------------------------------      0.073      0.000      0.000
--------------------------------------------------TCAACCTGCGCCTGGGCCGC---------------------------------------------------------------------------      0.073      0.100      0.000
--------------------------------------------------TCAACCTGCGCCTGGGCCGCG--------------------------------------------------------------------------      0.073      0.000      0.000 miR_t122*
---------------------------------------------------CAACCTGCGCCTGGGCCGCGT-------------------------------------------------------------------------      0.147      0.000      0.000
------------------------------------------------------------CCTGGGCCGCGTCTTCCTCCT----------------------------------------------------------------      0.441      0.000      0.000
---------------------------------------------------------------GGGCCGCGTCTTCCTCCTCGT-------------------------------------------------------------      0.514      0.000      0.408
-----------------------------------------------------------------------TCTTCCTCCTCGTCGACGCG------------------------------------------------------      0.073      0.000      0.082
------------------------------------------------------------------------CTTCCTCCTCGTCGACGCG------------------------------------------------------      0.073      0.000      0.000
-------------------------------------------------------------------------TTCCTCCTCGTCGACGCGT-----------------------------------------------------      0.073      0.000      0.000
-----------------------------------------------------------------------TCTTCCTCCTCGTCGACGCGT-----------------------------------------------------     32.599      7.502     11.497 miR_t70
----------------------------------------------------------------------GTCTTCCTCCTCGTCGACGCGT-----------------------------------------------------      2.203      0.300      1.223
------------------------------------------------------------------------CTTCCTCCTCGTCGACGCGT-----------------------------------------------------      2.643      1.500      2.528
-------------------------------------------------------------------------TTCCTCCTCGTCGACGCGTGT---------------------------------------------------      0.073      0.200      0.082
------------------------------------------------------------------------CTTCCTCCTCGTCGACGCGTGT---------------------------------------------------      0.514      0.100      0.326
-----------------------------------------------------------------------TCTTCCTCCTCGTCGACGCGTGT---------------------------------------------------      0.147      0.100      0.000
---------------------------------------------------------------------------------CGTCGACGCGTGTCGGGAAA--------------------------------------------      0.147      0.200      0.000
--------------------------------------------------------------------------------TCGTCGACGCGTGTCGGGAAA--------------------------------------------      0.073      0.000      0.000
------------------------------------------------------------------------------------CGACGCGTGTCGGGAAACT------------------------------------------      0.073      0.000      0.000
--------------------------------------------------------------------------------------------GTCGGGAAACTCGGACATC-GG-------------------------------      0.441      0.400      0.082
----------------------------------------------------------------------------------------------CGGGAAACTCGGACATC-GGA------------------------------      0.073      0.000      0.000
--------------------------------------------------------------------------------------------GTCGGGAAACTCGGACATC-GGA------------------------------      4.038      0.500      1.386
---------------------------------------------------------------------------------------------TCGGGAAACTCGGACATC-GGA------------------------------      0.147      0.100      0.000
```

Figure S1. Secondary structure plots of predicted miRNA precursor loci and frequency of reads from the Chlamydomonas AGO3-associated sRNA libraries or from the total sRNA libraries prepared by Chávez Montes *et al.*[25]. For AGO3-associated sRNAs, the first column indicates read abundance (Counts Per Million mapped reads) in libraries prepared from cells grown mixotrophically (TAP), the second column indicates read abundance in libraries prepared from cells grown photoautotrophically in nitrogen depleted minimal medium (HS-N) and the third column indicates read abundance in libraries prepared from cells grown photoautotrophically in nutrient replete minimal medium (HS+N). For the miRNAs identified in the libraries prepared by Chávez Montes *et al.*[25], the first column indicates read abundance (CPM) in the libraries from cells grown in nutrient replete medium, the second column indicates read abundance in the libraries from cells grown in sulfur deprived medium and the third column indicates read abundance in the libraries from cells grown in phosphate deprived medium. Mature miRNAs are indicated in red and passenger strands (miRNA*), when detected, are indicated in blue. Each plot is annotated with the locus ID (miR_t#), the chromosome, the chromosomal position (start and end), and the trophic condition(s) under which the miRNA was preferentially identified. Loci with long hairpins may code for more than one candidate miRNA. Additionally, some loci code for more than one hairpin in a tandem arrangement (clustered miRNA precursor loci) and, in these cases, the folding of each hairpin is shown separately.

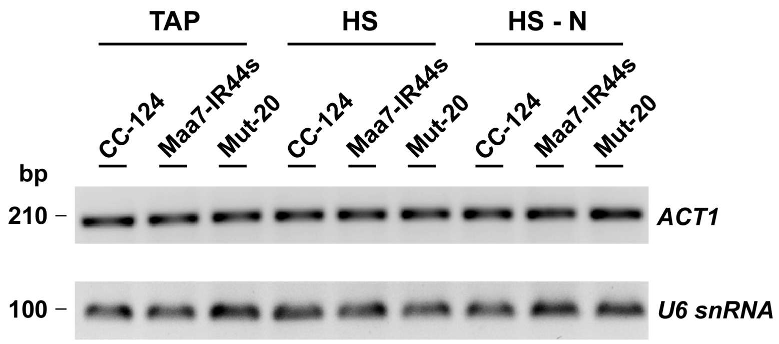

Figure S2. U6 small nuclear RNA abundance in Chlamydomonas cells grown under the denoted trophic conditions. Transcript abundance was examined by semi-quantitative RT-PCR. The panels show representative reverse images of agarose resolved RT-PCR products stained with ethidium bromide. CC-124, wild type strain (mt$^-$); Maa7-IR44s, CC-124 containing a transgene expressing FLAG-tagged AGO3; Mut-20, *TSN1* deletion mutant, in the Maa7-IR44s background, defective in sRNA biogenesis[14]. Amplification of the mRNA corresponding to *ACT1* (encoding actin) was used as a control for equivalent amounts of input RNA and for the efficiency of the RT-PCRs [Msanne, J. *et al. Phytochemistry* **75**, 50-59 (2012)].

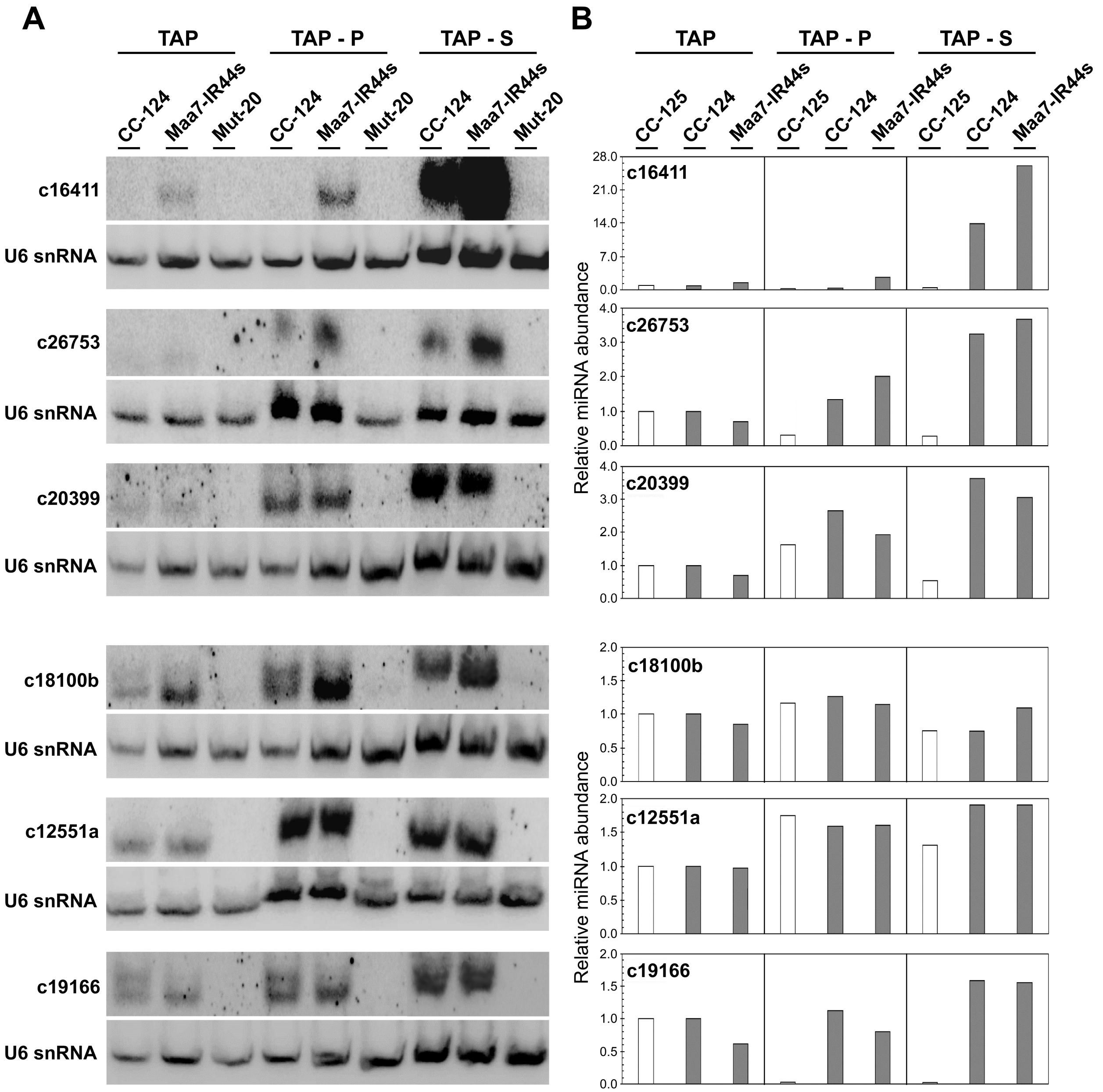

Figure S3. Northern blot analysis of miRNA expression in Chlamydomonas cells grown under the denoted trophic conditions. (**A**) Small RNAs were detected with probes specific for the indicated miRNAs. The same filters were reprobed with the U6 small nuclear RNA sequence as a control for lane loading. CC-124, wild type strain (mt$^-$); CC-125, wild type strain (mt$^+$); Maa7-IR44s, CC-124 containing a transgene expressing FLAG-tagged AGO3; Mut-20, *TSN1* deletion mutant, in the Maa7-IR44s background, defective in sRNA biogenesis[14]. (**B**) Relative miRNA levels in the indicated strains under the different trophic conditions. Values shown are the average of two independent experiments and are normalized to those of the CC-124 strain (the CC-125 strain for read counts) grown mixotrophically in nutrient replete medium (TAP). The relative standard deviation, as percentage of the mean, was in no case higher than 34.7%. Data corresponds to phosphorimager measurements of sRNA signals on northern blots (gray bars) or normalized read counts from the total sRNA libraries prepared by Chávez Montes *et al.*[25] (white bars).

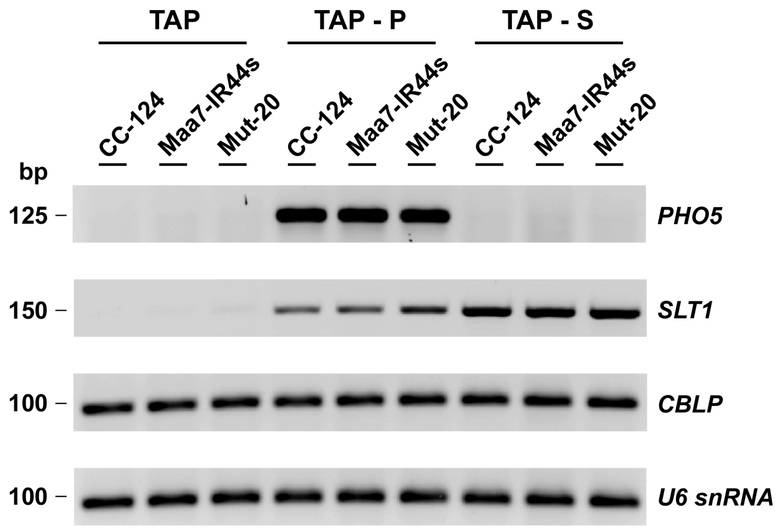

Figure S4. Gene expression in Chlamydomonas cells grown under the denoted trophic conditions. Transcript abundance was examined by semi-quantitative RT-PCR. The panels show representative reverse images of agarose resolved RT-PCR products stained with ethidium bromide. CC-124, wild type strain (mt$^-$); Maa7-IR44s, CC-124 containing a transgene expressing FLAG-tagged AGO3; Mut-20, *TSN1* deletion mutant, in the Maa7-IR44s background, defective in sRNA biogenesis[14]. Examined genes included *PHO5*, encoding a phosphate-repressible alkaline phosphatase, *SLT1* (SAC1-*LIKE TRANSPORTER1*), encoding a sodium/sulfate cotransporter, and the *U6* small nuclear RNA. Amplification of the mRNA corresponding to *CBLP* (also known as *RECEPTOR OF ACTIVATED PROTEIN KINASE C1, RCK1*) was used as a control for equivalent amounts of input RNA and for the efficiency of the RT-PCRs [Aksoy, M. *et al. Plant Physiol* **162**, 195-211 (2013)].

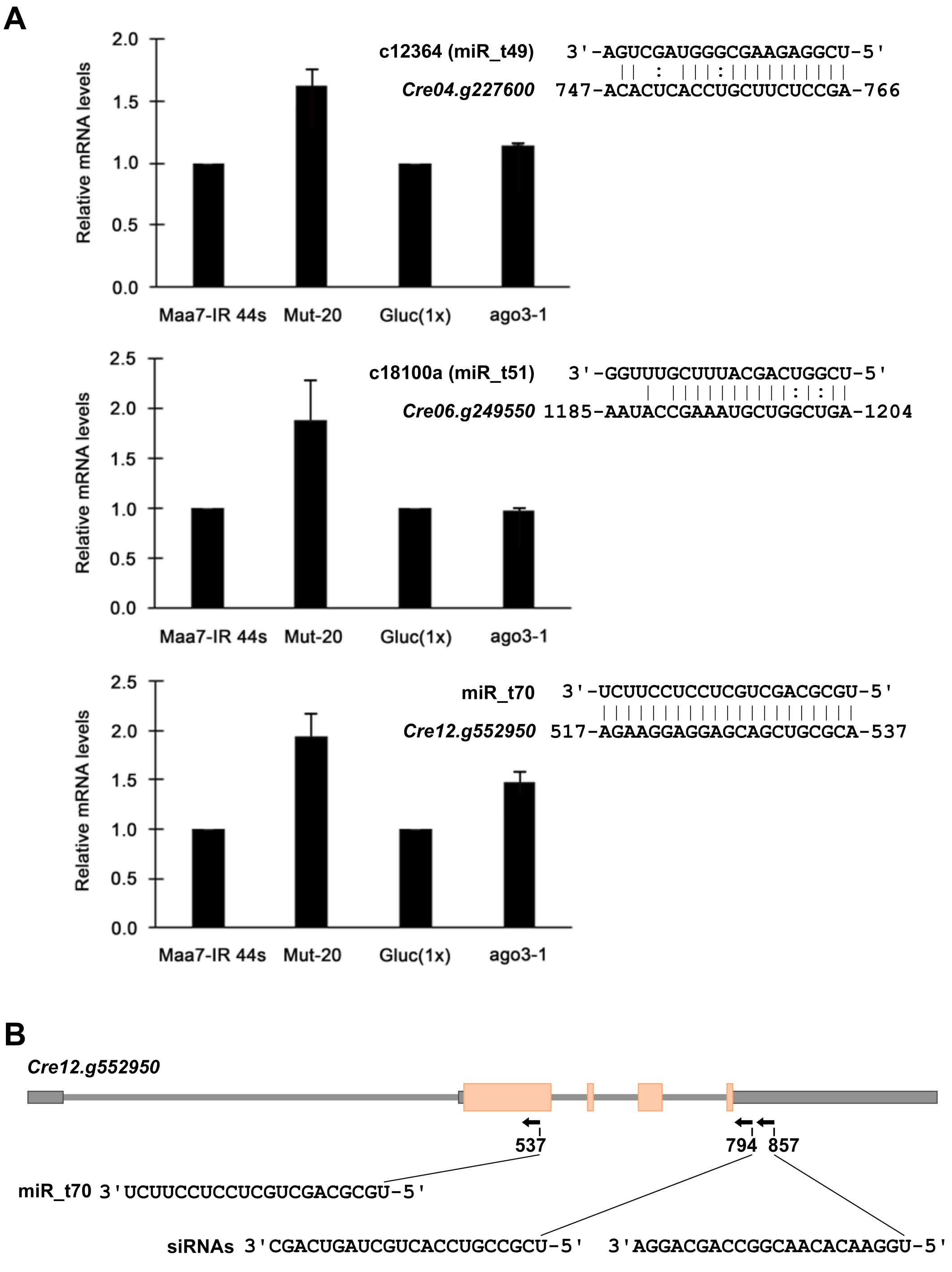

Figure S5. Quantitative RT-PCR analysis of predicted miRNA-target transcript abundance. (**A**) Relative transcript levels of *Cre04.g227600* (target of c12364), *Cre06.g249550* (target of c18100a) and *Cre12.g552950* (target of miR_t70) in the indicated strains. Maa7-IR44s, CC-124 strain containing a transgene expressing FLAG-tagged AGO3; Mut-20, *TSN1* deletion mutant, in the Maa7-IR44s background, defective in sRNA biogenesis[14]; Gluc(1x), wild type strain derived from CC-124; ago3-1, *AGO3* disrupted mutant, in the Gluc(1x) background, defective in RNAi[28]. Values, normalized to those in the wild type strains, are means ± SD of three independent experiments. (**B**) Diagram of the *Cre12.g552950* gene (from Phytozome v11, coding region shown in orange), indicating the binding sites for miR_t70 and two putative, perfectly complementary, siRNAs.

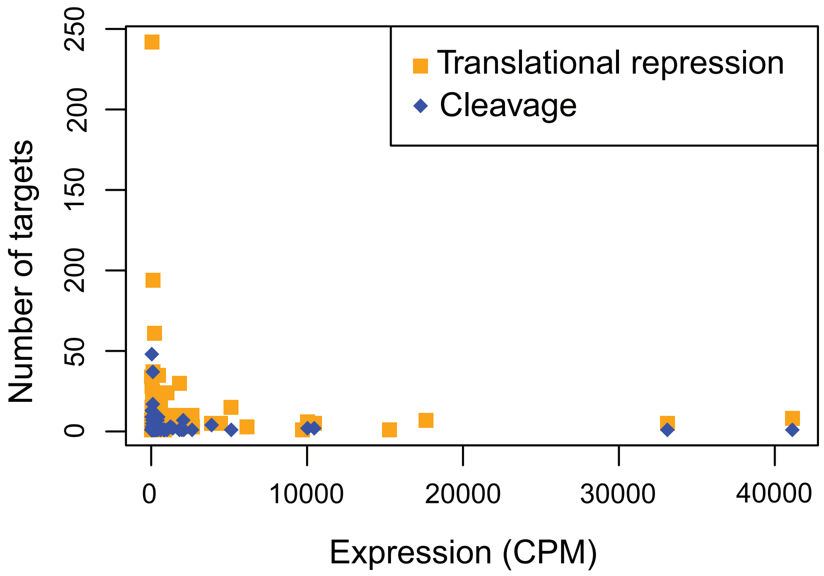

Figure S6. Relationship between miRNA expression level and number of predicted targets in *C. reinhardtii.* The expression level and the number of predicted targets (blue diamonds for cleavage targets and orange squares for translational repression targets) are plotted for each miRNA identified in the AGO3-pulldown libraries from *C. reinhardtii*.
